# Discovery of new VEGFR-2 inhibitors based on bis([1, 2, 4]triazolo)[4,3-*a*:3',4'-*c*]quinoxaline derivatives as anticancer agents and apoptosis inducers

**DOI:** 10.1080/14756366.2021.1915303

**Published:** 2021-05-31

**Authors:** Nawaf A. Alsaif, Mohammed S. Taghour, Mohammed M. Alanazi, Ahmad J. Obaidullah, Abdulrahman A. Al-Mehizia, Manal M. Alanazi, Saleh Aldawas, Alaa Elwan, Hazem Elkady

**Affiliations:** a Department of Pharmaceutical Chemistry, College of Pharmacy, King Saud University, Riyadh, Saudi Arabia; b Pharmaceutical Medicinal Chemistry & Drug Design Department, Faculty of Pharmacy (Boys), Al-Azhar University, Cairo, Egypt

**Keywords:** Anticancer, apoptosis, bis([1,2,4]triazolo)[4,3-*a*:3',4'-*c*]quinoxaline, molecular docking, VEGFR-2

## Abstract

Herein, a new wave of bis([1, 2, 4]triazolo)[4,3-*a*:3',4'-*c*]quinoxaline derivatives have been successfully designed and synthesised. The synthesised derivatives were biologically investigated for their cytotoxic activities against HepG2 and MCF-7. Also, the tested compounds were further examined *in vitro* for their VEGFR-2 inhibitory activity. The most promising derivative **23j** was further investigated for its apoptotic behaviour in HepG2 cell lines using flow cytometric and western-plot analyses. Additional *in-silico* studies were performed to predict how the synthesised compounds can bind to VEGFR-2 and to determine the drug-likeness profiling of these derivatives. The results revealed that compounds **23a**, **23i**, **23j**, **23l**, and **23n** displayed the highest antiproliferative activities against the two cell lines with IC_50_ values ranging from 6.4 to 19.4 µM. Furthermore, compounds **23a**, **23d**, **23h**, **23i**, **23j**, **23l**, **23 m**, and **23n** showed the highest VEGFR-2 inhibitory activities with IC_50_ values ranging from 3.7 to 11.8 nM, comparing to sorafenib (IC_50_ = 3.12 nM). Moreover, compound **23j** arrested the HepG2 cell growth at the G2/M phase and induced apoptosis by 40.12% compared to the control cells (7.07%). As well, such compound showed a significant increase in the level of caspase-3 (1.36-fold), caspase-9 (2.80-fold), and BAX (1.65-fold), and exhibited a significant decrease in Bcl-2 level (2.63-fold).

## 1. Introduction

Cancer is a rebound system represented by unrestricted cell growth^1^. Cancer originates in the humanoid body from the buildup of genetic and epigenetic variations in the normal cells^2^. In spite of the huge efforts directed towards cancer treatment and prevention, cancer remains one of the foremost public health problems all over the world^3^. Consequently; increasing interest in the current medicinal chemistry has been dedicated to the design and synthesis of more effective anticancer agents with low side effects^4^.

Angiogenesis is a multi-stage process to produce new vessels from quiescent pre-existing ones^5–7^. It is interrelated to several physiological functions and also, it is a fundamental step in numerous diseases including cancer^8^. Furthermore, it has a significant role in tumour progression and development^9^^,^^10^.

At present, there are main approaches in targeting angiogenesis that has been verified in clinical trials and officially approved in clinical practice^11^: i) monoclonal antibodies which bind to vascular endothelial growth factor-A (VEGF-A) e.g. bevacizumab^12^; ii) VEGF-trap e.g. aflibercept which binds to VEGF-A, VEGF-B, and placental growth factor (PGF)^13^; iii) monoclonal antibodies targeting VEGF receptors and block the binding of natural VEGFR ligands e.g. ramucirumab, iv) tyrosine kinase inhibitors (TKIs) which are drugs that inhibit the kinase activity of VEGF receptors through binding to the ATP binding site. Hence, TKIs hinder the phosphorylation of the tyrosine residue and subsequent transmission of signalling down the intercellular pathway. Among this class of agents, sorafenib **1** and sunitinib **2** are the prototypes^14^. There have been numerous known tyrosine kinase receptors such as vascular endothelial growth factor receptors (VEGFRs) and endothelial growth factor receptors (EGFRs)^15^.

It has been stated that the vascular endothelial growth factor (VEGF) can stimulate the production and movement of vascular endothelial cells and regulate the formation of blood vessels^12^^,^^16^. There are three main vascular endothelial growth factor receptors (VEGFR-1, VEGFR-2, and VEGFR-3), which are strategic intermediates in angiogenesis and in the construction of new networks of blood vessels required to hoard oxygen and nourishment for cancer growth^17^.

Vascular endothelial growth factor receptor-2 (VEGFR-2) is the most critical regulator of angiogenic factors that plays a significant role in tumour survival, angiogenesis, and migration^18^. Binding of VEGFR-2 to VEGF leads to inspiration of downstream signalling pathway and certain endothelial reactions, such as improved permeability of vascular cells and increased endothelial cell multiplying and propagation, consequently, lead to angiogenesis^19^. Hence, blockage of VEGF/VEGFR-2 system is considered a favourable approach for anti-angiogenic therapy in retarding cancer growth^20^^,^^21^. Additionally, VEGFR-2 has been confirmed to motivate apoptosis in cancer cells which synergistically enhances the antitumor effect^22–25^.

Apoptosis has two major pathways; the intrinsic pathway which is controlled by the Bcl-2 family (involve BAX and Bcl-2) and the extrinsic pathway which is controlled by a subgroup of tumour necrosis factor receptors superfamily (TNFR)^26^. Immediately as the apoptosis process is instigated, caspases, a family of protease enzymes, are activated. Caspases are classified into initiator caspases as caspases 8 and 9, effector caspases as caspases 3, 6, and, 7 and inflammatory caspases as caspases 1, 4, 5, 11, and 12^26^. Caspase 9 is the initiator of intrinsic apoptosis while caspase 3 plays a central role in the execution phase of apoptosis^27^. Cancer cells can avoid apoptosis by hindering caspase function, decreasing BAX expression level or increasing the gene expression level of anti-apoptotic Bcl-2^27^^,^^28^.

**Figure 1.**
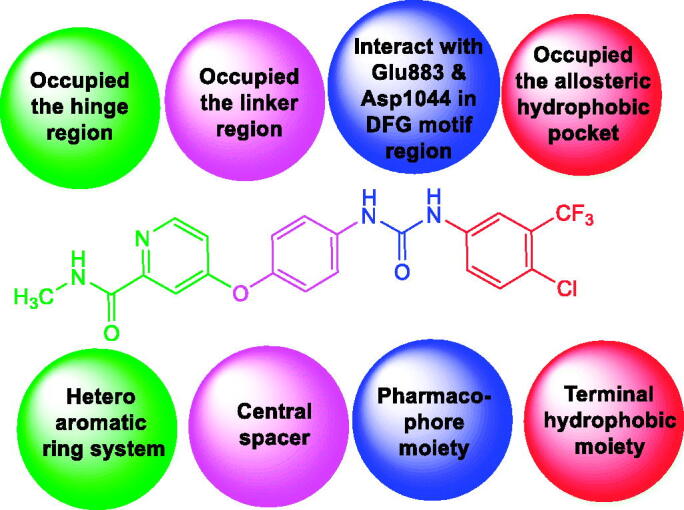
The essential structural requirements of reported VEGFR-2 inhibitors.

A literature study revealed that VEGFR-2 inhibitors have main pharmacophoric features^29–31^. (i) A flat heteroaromatic ring system that can accommodate the hinge region^29^. (ii) A central spacer moiety that can occupy the linker region between the hinge region and DFG domain of the enzyme^32^. (iii) A pharmacophore moiety consists of an H-bond acceptor (HBA) and an H-bond donor (HBD). Such pharmacophore moiety can interact with the two vital amino acid residues (Asp1044 and Glu883) in the DFG motif region^33^. (iv) A terminal hydrophobic moiety that occupies the allosteric hydrophobic pocket via numerous hydrophobic interactions^34^ (Figure 1).

**Figure 2.**
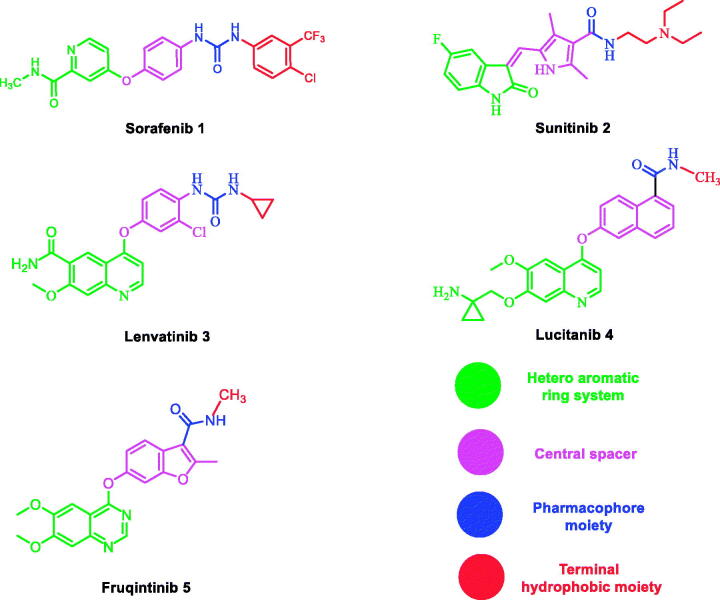
Structures of some representative VEGFR-2 inhibitors.

Also, a literature review revealed that many VEGFR-2 inhibitors have been approved as potent anticancer agents such as sorafenib **1**^35^, Sunitinib **2**^36^, lenvatinib **3**^37^^,^^38^, lucitanib **4**^37^^,^^38^, and fruquinitinib **5**^39^. All of them fulfilled the main pharmacophoric features of VEGFR-2 inhibitors (Figure 2).

### 1.1. Rationale of molecular design

Based on facts mentioned above and in the extension of our efforts to discover novel molecules with potential anticancer activity^5^^,^^40–48^, we design and synthesise a new wave of bis([1, 2, 4]triazolo)[4,3-*a*:3′,4′-*c*]quinoxaline derivatives having the basic crucial pharmacophoric features of VEGFR-2 inhibitors.

**Figure 3.**
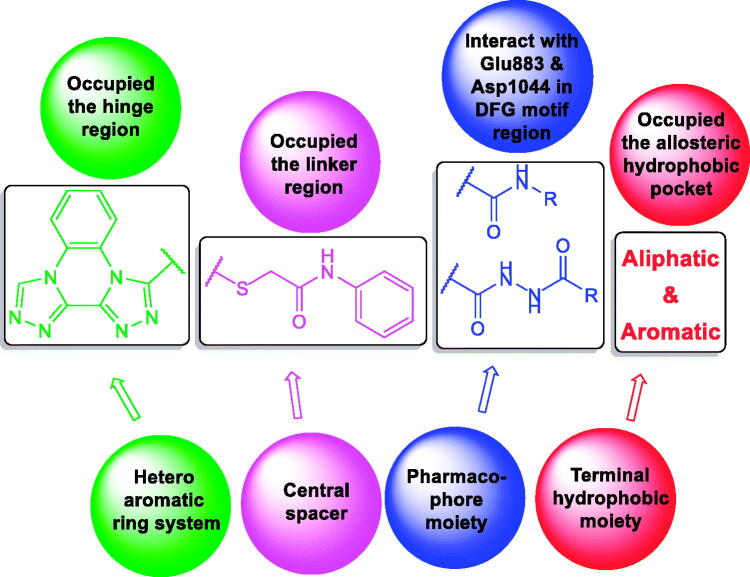
Schematic representation showing the designing strategy.

**Figure 4.**
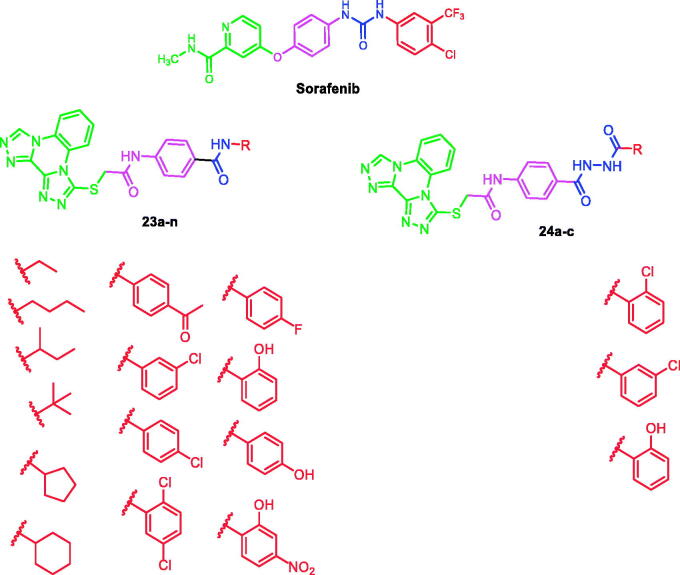
The target compounds fulfilled the pharmacophoric features of VEGFR-2 inhibitors.

At first, bis([1, 2, 4]triazolo)[4,3-*a*:3′,4′-*c*]quinoxaline moiety was selected as a heteroaromatic ring system to occupy the hinge region in the ATP binding site. Such moiety was accomplished to attain more rigid structures that could increase binding affinity against the active site. Then, sulfanyl-*N*-phenylacetamide moiety was utilised as a central spacer to occupy the linker region. Such spacer was expected to increase the flexibility of the designed compounds. Regarding the DFG-motif region, we used two different moieties having the essential HBA/HBD features to play the role of the pharmacophore. The pharmacophores were designed to be amide moiety (compounds **23a**–**n**), or diamide moiety (compounds **24a**–**c**). Finally, the allosteric hydrophobic region can be occupied by different terminal aliphatic moieties (**23a**–**f**) or terminal aromatic moieties (**23 g**–**n** and **24a**–**c**). These wide varieties of modifications helped us to study the SAR of these compounds as efficient anticancer agents with potential VEGFR-2 inhibitory activities. All modification pathways and molecular design rationale were illustrated and summarised in Figure 3. As shown in Figure 4, the target compounds achieved the essential pharmacophoric requirements of VEGFR-2 inhibitors.

To attain our aim of this work, cytotoxicity and VEGFR-2 inhibitory effects of the synthesised compounds were assessed. Besides, flow cytometric analyses were carried out for the most active candidate to estimate its potential against apoptosis induction and cell cycle arrest. Furthermore, such a compound was examined against several apoptotic markers include caspase 3/9 and BAX, and Bcl-2.

## 2. Results and discussion

### 2.1. Chemistry

**Scheme 1.**
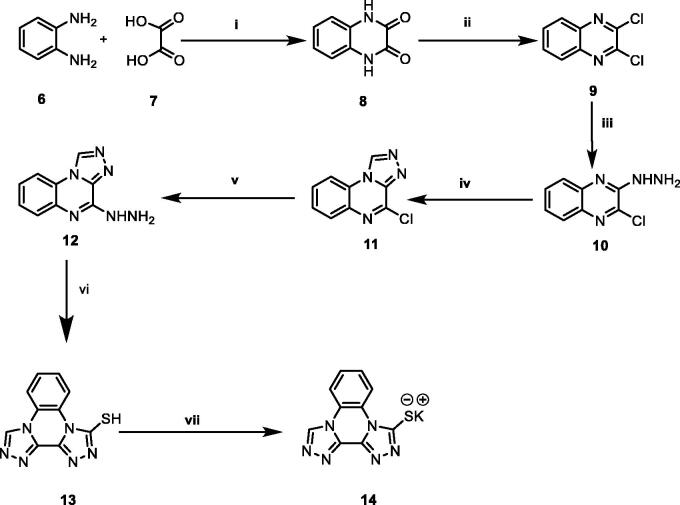
Synthetic pathway for compound **14**; Reagents and conditions: (i) 4 N conc. HCl/reflux/6 h, (ii) SOCl_2_/DCE/reflux/4 h, (iii) NH_2_NH_2_.H_2_O/ethanol/r.t., (iv) triethyl orthoformate/reflux/4 h, (v) NH_2_NH_2_.H_2_O/ethanol/reflux/4 h, (vi) absolute ethanol/KOH/CS_2_/reflux/6 h, (vii) absolute ethanol/KOH/heating/10 min.

**Scheme 2.**
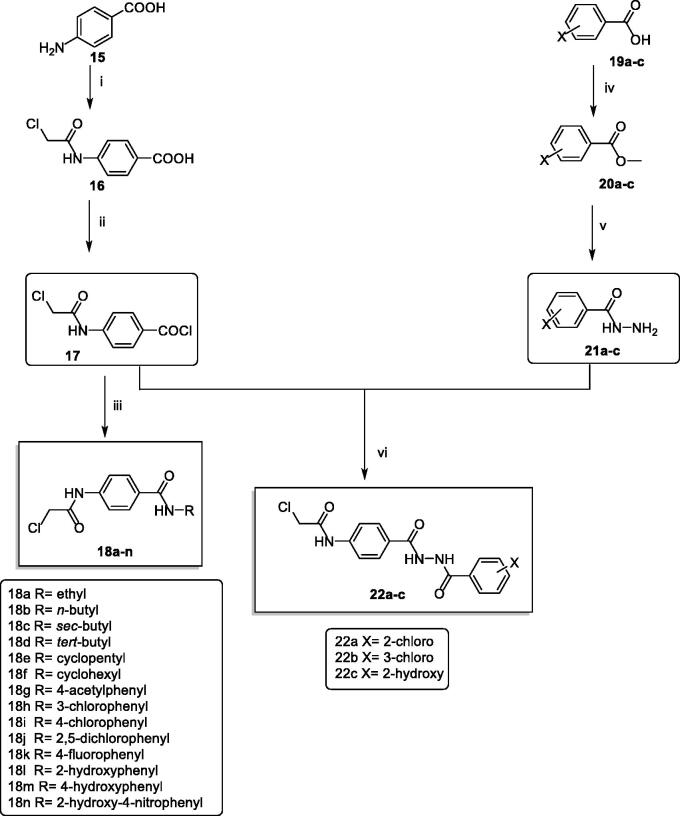
Synthetic pathway for compounds **18a**–**n** and **22a**–**c**; Reagents and conditions: (i) ClCH_2_COCl/DMF/NaHCO_3_/r.t./1 h, (ii) SOCl_2_/DCE/DMF/reflux/4 h, (iii) RNH_2_/acetonitrile/TEA/r.t./8 h (iv) methanol/conc.H_2_SO_4_/reflux/8 h, (v) NH_2_NH_2_.H_2_O/ethanol/reflux/8 h, (vi) acetonitrile/TEA/r.t./8 h.

**Scheme 3.**
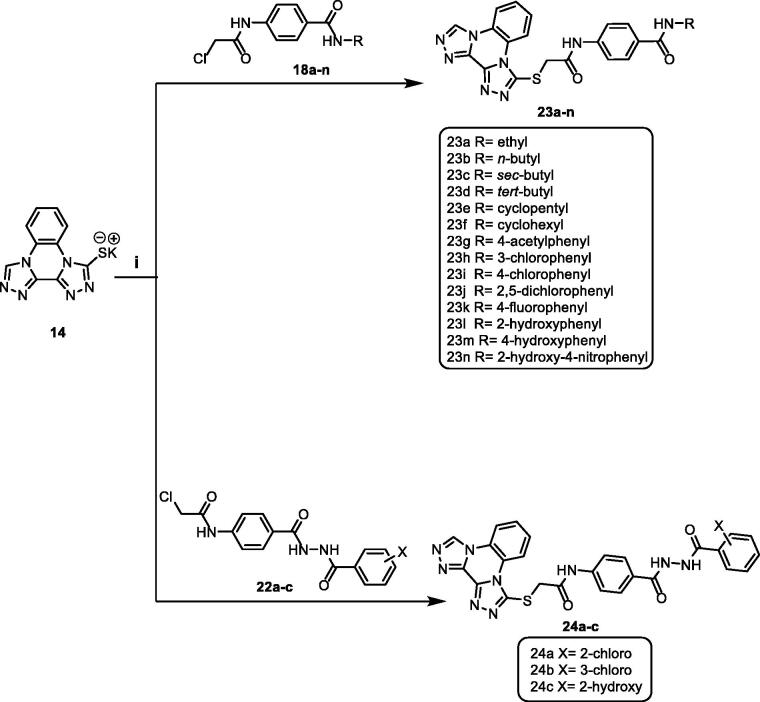
Synthetic pathway for compounds **23a**–**n** and **24a**–**c**; Reagents and conditions: (i) DMF/KI/reflux/6 h.

The general synthetic pathways adopted for the synthesis of the designed compounds are illustrated in Schemes 1–3. Scheme 1 depicts the synthesis of potassium bis[1, 2, 4]triazolo[4,3-*a*]quinoxaline-4-thiolate **14**. Initially, *o*-phenylenediamine **6** was refluxed with oxalic acid **7** in the presence of 4 N HCl to afford 2,3-(1*H*,4*H*)-quinoxalinedione **8**^49^. Chlorination of compound **8** was done by refluxing with thionyl chloride yielding 2,3- dichloroquinoxaline **9**^49^. Subsequent treatment of the latter with hydrazine hydrate in absolute ethanol afforded 2-chloro-3-hydrazinylquinoxaline **10**^50^. Heating of compound **10** with triethyl orthoformate gave 4-chloro[1, 2, 4]triazolo[4,3-*a*]quinoxaline **11**^50^. The obtained compound **11** was heated with hydrazine hydrate to afford 4-hydrazinyl-[1, 2, 4] triazolo[4,3-*a*]quinoxaline **12**^51^. Moreover, reflux of **12** in an alcoholic mixture of carbon disulphide and potassium hydroxide afforded bis[1, 2, 4]triazolo[4,3-*a*:3′,4′-*c*]quinoxaline-3-thiol **13**^44^. Heating compound **13** with an alcoholic solution of potassium hydroxide gave the corresponding potassium salt **14**^44^. (Scheme 1)

Scheme 2 was carried out to prepare the key intermediates **(18a**–**n** and **22a**–**c)**. At first, acetylation of *p*-amino benzoic acid **15** with chloroacetyl chloride in DMF in the presence of NaHCO_3_ provided the key product 4–(2-chloroacetamido)benzoic acid **16**. Compound **16** was acylated using thionyl chloride to produce the benzoyl chloride derivative **17**^52^. Furthermore, stirring of compound **17** with commercially available amines namely, ethylamine, *n*-butylamine, *sec*-butylamine, *tert*- butylamine, cyclopentylamine, cyclohexylamine, 4-aminoacetophenone, 3-chloroaniline, 4-chloroaniline, 2,5-dichloroaniline, 4-fluoroaniline, 2-hydroxyaniline, 4-hydroxyaniline, and 2-hydroxy-4-nitroaniline at room temperature in acetonitrile/TEA mixture afforded the corresponding intermediates **18a**–**n**, respectively. On the other hand, to prepare the ester derivatives **20a**–**c,** appropriate acid derivatives **19a**–**c** namely, 2-chlorobenzoic acid, 3-chlorobenzoic, and 2-hydroxybenzoic acid were refluxed in methanol in the presence of sulphuric acid according to the reported procedures^42^^,^^53^^,^^54^. In addition, reflux of **20a**–**c** with hydrazine hydrate afforded the corresponding acid hydrazides **21a**–**c**^42^. In the end, in acetonitrile and TEA mixture, compound **17** was stirred with the acid hydrazides **21a**–**c** to produce the corresponding diamide derivatives **22a**–c (Scheme 2).

The structures of compounds **18a**–**n** and **22a**–**c** were confirmed by spectral and elemental data. IR spectra of such compounds showed strong bands around 3370 − 3100 cm^−1^ corresponding to NHs. Also, IR spectra showed strong C=O absorption bands at a range of 1770 − 1624 cm^−1^. Moreover, ^1^H NMR spectra showed singlet signals around *δ* 10.50 and 8.35 ppm corresponding to the two amidic NHs. Additionally, CH_2_ protons appeared at around *δ* 4.30 ppm. Matching with such results, ^13^C NMR spectra also confirmed the validity of suggested structures where characteristic peaks were displayed around *δ* 165.60, 165.05, and 44.00 ppm corresponding to the two C=O and CH_2_ groups, respectively.

Scheme 3 demonstrated the synthetic pathway of the final target compounds (**23a**–**n** and **24a**–**c)**. Compound **14** was heated with the formerly synthesised intermediates (**18a**–**n** and **22a**–**c)** in dry DMF using KI to furnish the titled compounds **23a**–**n** and **24a**–**c**, respectively.

The spectral and elemental data supported the structures of the obtained derivatives, where the ^1^H NMR spectra of compounds **23a**–**n** and **24a**–**c** displayed characteristic downfield singlet signals around *δ* 10.75 ppm. The mass spectra were also consistent with the expected structures. Taking compound **23d** as a representative example, the IR spectrum demonstrated stretching bands at 2968 and 2929 cm^−1^ corresponding to aliphatic CH bonds. The ^1^H NMR spectrum of this compound showed an up-field singlet signal at *δ* 1.38 ppm corresponding to tertiary butyl moiety. Furthermore, ^13^C NMR spectrum showed the presence of two peaks at *δ* 51.16 and 29.11 corresponding to CH and three CH_3_ of *tert*-butyl moiety.

### 2.2. Biological evaluation

#### 2.2.1. *In vitro* anti-proliferative activity

All newly prepared compounds were assessed for their *in vitro* cytotoxic efficiencies via standard MTT method^55–57^, against breast cancer (MCF-7) and hepatocellular carcinoma (HepG2) cell lines. Sorafenib was applied as a standard anticancer drug. The growth inhibitory concentration (IC_50_) values were concluded for each final compound and depicted in Table 1.

**Table 1.** *In vitro* anti-proliferative activities of the tested compounds against MCF-7 and HepG2 cell lines, and *in vitro* enzymatic inhibitory activities against VEGFR-2.

	IC_50_ (µM)^a^		IC_50_ (nM)^a^
Comp.	MCF-7	HepG2	VEGFR-2
**23a**	17.7 ± 1.5	11.3 ± 0.9	7.1 ± 0.4
**23b**	78.9 ± 6.1	69.1 ± 6.1	71.6 ± 5.2
**23c**	76.2 ± 6.9	69.8 ± 5.9	47.1 ± 4.2
**23d**	21.6 ± 1.8	17.5 ± 1.2	11.8 ± 0.8
**23e**	78.3 ± 6.5	74.8 ± 6.9	39.8 ± 3.1
**23f**	74.1 ± 6.7	70.4 ± 5.8	39.5 ± 2.9
**23g**	81.3 ± 7.4	80.7 ± 7.1	62.7 ± 5.7
**23h**	37.2 ± 3.2	22.3 ± 1.8	11.7 ± 0.9
**23i**	18.3 ± 1.2	10.8 ± 0.9	9.4 ± 0.7
**23j**	10.3 ± 0.8	6.4 ± 0.5	3.7 ± 0.2
**23k**	68.2 ± 5.2	71.8 ± 6.3	49.6 ± 4.1
**23l**	19.4 ± 1.1	11.3 ± 0.7	5.8 ± 0.3
**23m**	21.6 ± 1.7	15.2 ± 1.2	9.7 ± 0.7
**23n**	16.5 ± 1.3	12.7 ± 0.9	7.4 ± 0.5
**24a**	48.3 ± 3.9	34.5 ± 2.8	23.9 ± 1.9
**24b**	42.7 ± 3.7	30.3 ± 2.7	22.3 ± 1.8
**24c**	40.7 ± 3.7	29.8 ± 2.1	20.7 ± 1.6
**Sorafenib**	3.51 ± 0.22	2.17 ± 0.14	3.12 ± 0.1

^a^IC_50_ values are the mean ± SD of three separate experiments.

Overall, HepG2 cells were more sensitive to the tested compounds than MCF-7 except for compound **23k**. Among the series, compound **23j** showed the highest anti-proliferative activities against MCF-7 and HepG2 cell lines with IC_50_ values of 10.3 and 6.4 µM, respectively. In addition, compounds **23a**, **23i**, **23 l**, and **23n** exhibited good anti-proliferative activities against the tested cell lines with IC_50_ values ranging from 7.1 to 19.4 µM, comparing to sorafenib (IC_50_ = 3.51 and 2.17 µM against MCF-7 and HepG2, respectively). Moreover, compounds **23d**, **23h**, **23m**, **24a**, **24b**, and **24c** displayed moderate anti-proliferative activities against the tested cell lines with IC_50_ values ranging from 15.2 to 48.3 µM. On the other hand, the rest of the compounds displayed weak anti-proliferative activities against the tested cell lines.

#### 2.2.2. *In vitro* VEGFR-2 enzyme assay inhibition

All the synthesised compounds were subjected to further assay for their ability to inhibit VEGFR-2 using sorafenib as a positive control. The results were stated as growth inhibitory concentration (IC_50_) values and illuminated in Table 1.

Compound **23j** was the most potent VEGFR-2 inhibitor with an IC_50_ value of 3.7 nM, nearly equal to that of sorafenib (IC_50_ = 3.12 nM). Moreover, compounds **23a**, **23d**, **23h**, **23i**, **23l**, **23m**, and **23n** showed promising activities with IC_50_ values ranging from 5.8 to 11.8 nM. On the other hand, compounds **23c**, **23e**, **23f**, **23k**, and **24a**–**c** exhibited moderate to weak activity with IC_50_ values ranging from 20.7 to 49.6 nM. Finally, compounds **23b** and **23g** exhibited the lowest anti VEGFR-2 activities with IC_50_ values of 71.6 and 62.7 nM, respectively.

#### 2.2.3. Structure–activity relationship (SAR)

Inspecting the results of different biological analyses (anti-proliferative activity and VEGFR-2 assay); we concluded a valuable SAR.

At First, the effect of pharmacophore moiety on the activity was explored. It was noticed that the amide derivatives **23h** (IC_50_ = 37.2 and 22.3 µM against MCF-7 and HepG2, respectively & 11.7 nM against VEGFR-2) and **23 l** (IC_50_ = 19.4 and 11.3 µM against MCF-7 and HepG2, respectively & 5.8 nM against VEGFR-2) displayed higher activities than the corresponding diamide derivatives **24b** (IC_50_ = 42.7 and 30.3 µM against MCF-7 and HepG2, respectively & 22.3 nM against VEGFR-2) and **24c** (IC_50_ = 40.7 and 29.8 µM against MCF-7 and HepG2, respectively & 20.7 nM against VEGFR-2).

Next, we investigated the effect of the terminal hydrophobic moiety. With respect to the terminal aliphatic hydrophobic moieties, it was found that the VEGFR-2 inhibitory activities decreased in the order of ethyl (**23a**, IC_50_ = 7.1 nM) > *tert*-butyl (**23d**, IC_50_ = 11.8 nM) > cyclohexyl (**23f**, IC_50_ = 39.5 nM) > cyclopentyl (**23e**, IC_50_ = 39.8 nM) > *sec*-butyl (**23c**, IC_50_ = 47.1 nM) > *n*-butyl (**23b**, IC_50_ = 71.6 nM). In addition, the effect of the substitution on the aromatic hydrophobic moieties has been examined. For the amide derivatives, it was found that the VEGFR-2 inhibitory activities decreased in the order of 2,5-dichloro (**23j**, IC_50_ = 3.7 nM) >2-hydroxy (**23l**, IC_50_ = 5.8 nM) >2-hydroxy −4-nitro (**23n**, IC_50_ = 7.4 nM) >4-chloro (**23i**, IC_50_ = 9.4 nM) >4-hydroxy (**23m**, IC_50_ = 9.7 nM) >4-fluoro (**23k**, IC_50_ = 49.6 nM). While the diamide derivatives revealed decrease in VEGFR-2 inhibitory activities in the order of 2- hydroxy (**24c**, IC_50_ = 20.7 nM) >3-chloro (**24b**, IC_50_ = 22.3 nM) > 2-chloro (**24a**, IC_50_ = 23.9 nM).

#### 2.2.4. Correlation of cytotoxicity with VEGFR-2 inhibition

**Figure 5.**
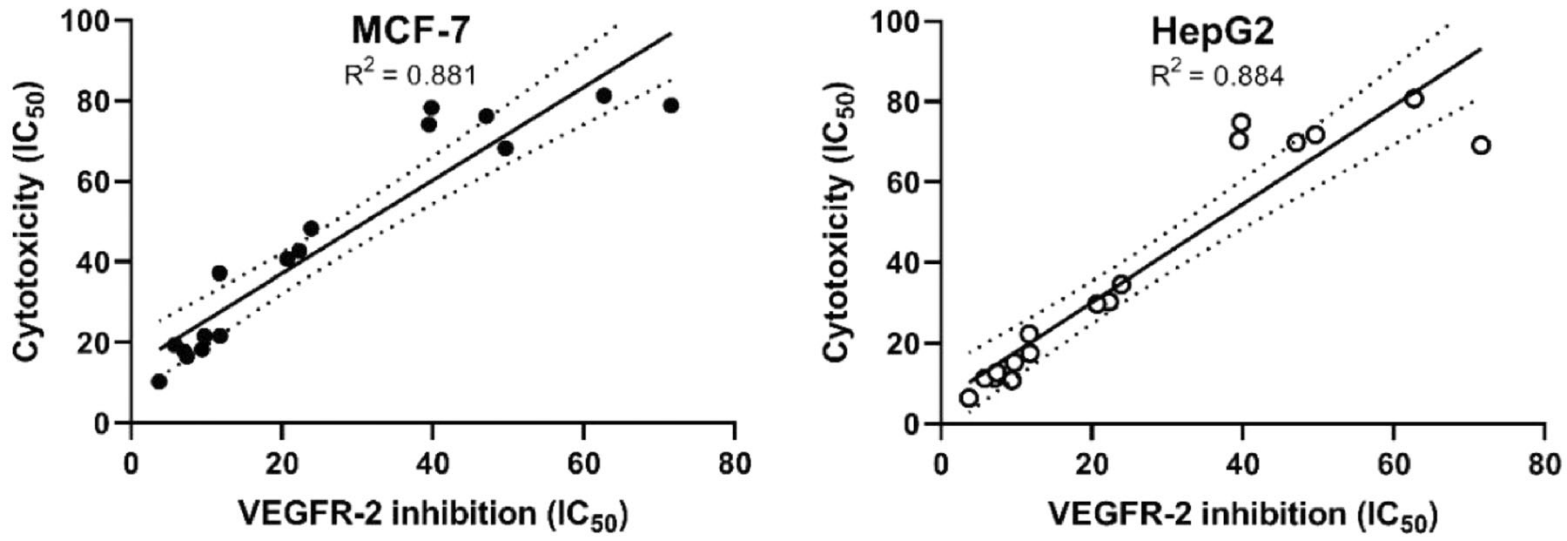
Correlation of cytotoxicity with VEGFR2 inhibition on two cell line models MCF-7and HepG2. MCF-7 (*p* value >.0001) & HepG2 (*p* value >.0001).

From the aforementioned results, we can conclude that our tested compounds can inhibit VEGFR-2 in the tested cell lines. To prove that inhibition of VEGFR-2 is the major prominent cause of cell mortality, we compared the cytotoxicity results of the synthesised candidates with their corresponding VEGFR-2 inhibitory activities utilising a simple linear regression analysis. The coefficients of determination (*R*^2^) were determined. The *R*^2^ values for MCF-7 and HepG2 were 0.881 (*p* values >.0001) and 0.800 (*p* values > .0001), respectively (Figure 5). Such high values of R^2^ indicate the high correlation between the dependent variable (VEGFR-2 inhibition) and the independent one (cytotoxicity).

#### 2.2.5. Cellular mechanistic study

Compound **23j** which demonstrated remarkable cytotoxic potency and significant inhibitory activity against VEGFR-2 was nominated for further cellular mechanistic study. This involved study of its influence on cell cycle progression and induction of apoptosis in HepG2 cells.

##### 2.2.5.1. Effect on cell cycle progression

**Figure 6.**
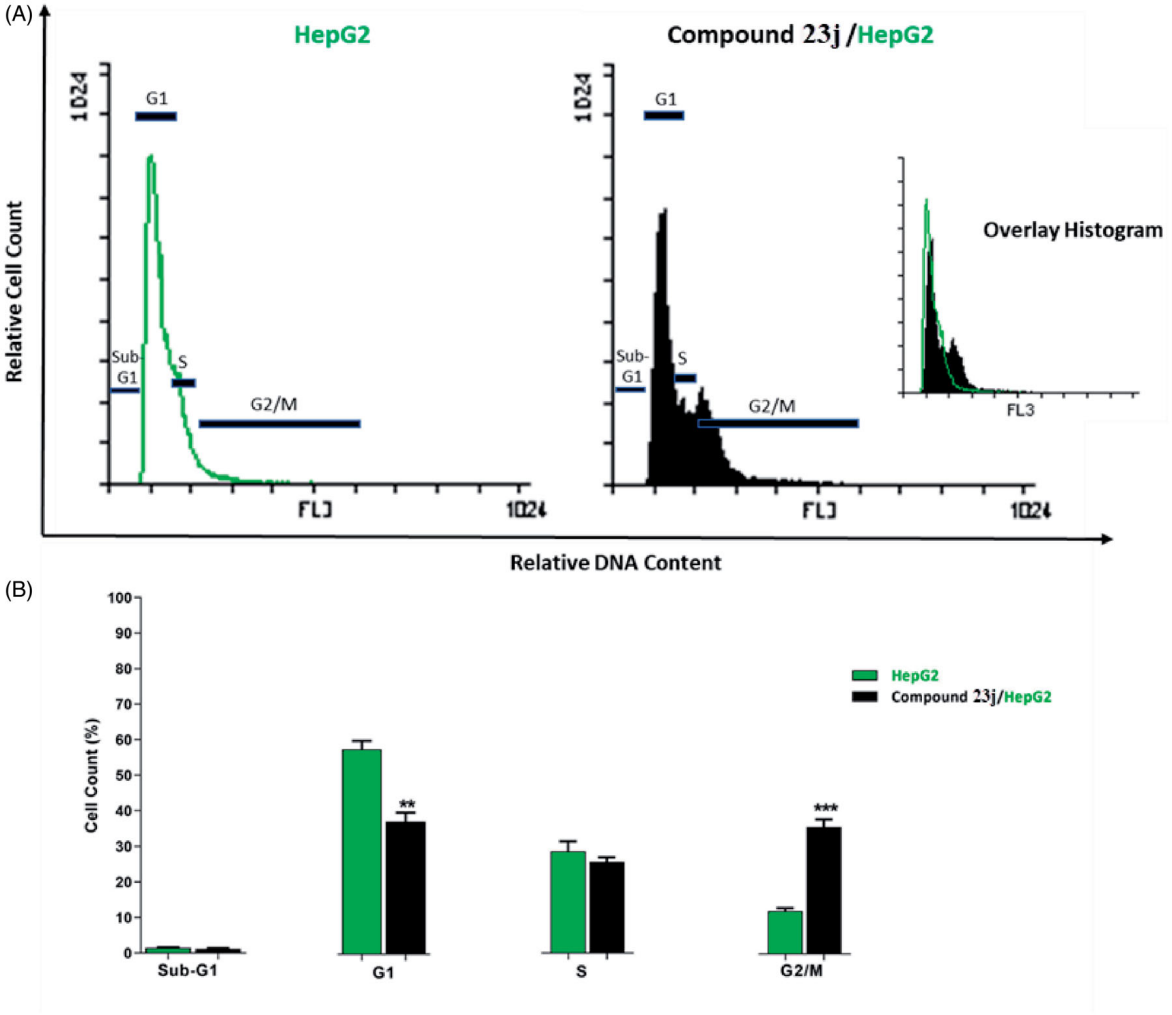
Cell cycle analysis of HepG2 cells treated with compound **23j**. **p < .01 and ***p < .001.

In this work, HepG2 cell line was treated with compound **23j** at a concentration of 6.4 μM (the IC_50_ value of compound **23j**) and incubated for 24 h. Then, the cells were stained with propidium iodide and analysed for cell distribution during the various phases of the cell cycle against untreated HepG2 cells. Flow cytometry results exhibited that the percent of HepG2 cells decreased at the Sub-G1, G1 and S phases. For Sub-G1 phase, it decreased from 1.46% to 1.21%, for G1 phase it decreased from 57.75 to 37.34% while for S phase it decreased from 28.65% to 25.79%. On the other hand, the percentage of HepG2 cells significantly increased at G2/M phase from 12.13% to 35.64%. Such results indicated that compound **23j** inhibited proliferation of HepG2 cells via cessation of the growth of the cell cycle at G2/M phase (Table 2 and Figure 6).

**Table 2.** Effect of compound **23j** on cell cycle progression in HepG2 cells after 24 h treatment.

Sample	Cell cycle distribution (%)^a^			
	%Sub-G1	%G1	%S	%G2 / M
HepG2	1.46 ± 0.17	57.75 ± 2.34	28.65 ± 2.74	12.13 ± 0.80
Compound **23j/HepG2**	1.21 ± 0.15	37.34 ± 2.50**	25.79 ± 1.21	35.64 ± 2.16***

^a^Values are given as mean ± SEM of three independent experiments. **p < .01 and ***p < .001.

##### 2.2.5.2. Apoptosis analysis

**Figure 7.**
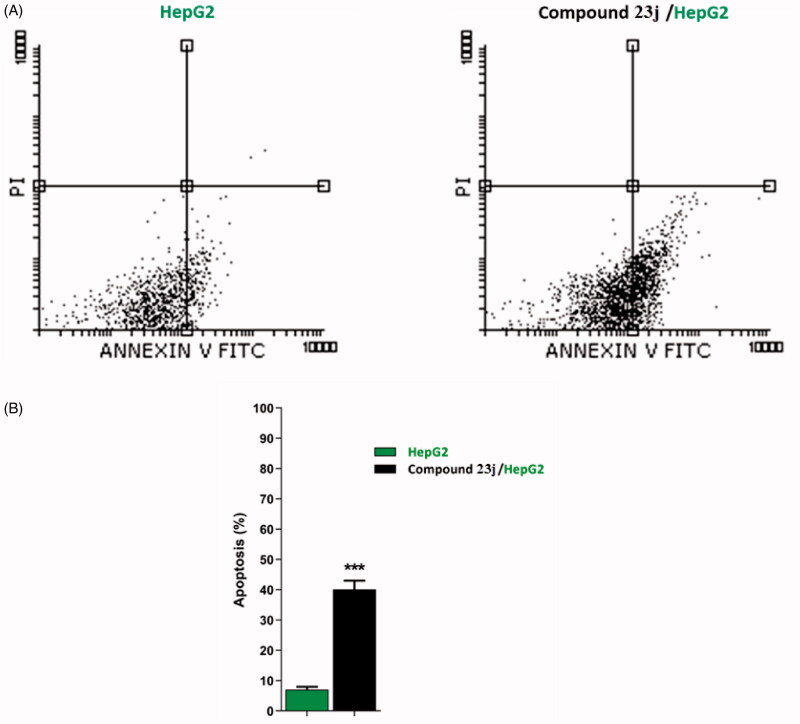
Flow cytometric analysis of apoptosis in HepG2 cells exposed to compound **23j**. ***p < .001.

To confirm the apoptotic ability of compound **23j**, a flow cytometry technique was performed. In such technique, HepG2 cells were stained with annexin V/propidium iodide (PI) and incubated for 24 h with compound **23j** (6.4 μM). It was revealed that compound **23j** triggered more apoptotic cells comparing to untreated control cells. In details, compound **23j** induced apoptosis by 40.12% (early apoptosis = 39.97% & late apoptosis = 0.15%), compared to 7.07% in the control cells (early apoptosis = 6.88% & late apoptosis = 0.19%). Such findings revealed that compound **23j** could induce apoptosis in HepG2 cells (Table 3 & Figure 7).

**Table 3.** apoptotic effect of compound **23j** against HepG2 cells.

Sample	Viable^a^ (left bottom)	Apoptosis^a^		Necrosis^a^ (left top)
		Early (right bottom)	Late (right top)	
HepG2	92.80 ± 1.91	6.88 ± 0.90	0.19 ± 0.01	0.14 ± 0.01
Compound **23j/HepG2**	59.61 ± 2.88	39.97 ± 2.93***	0.15 ± 0.01	0.24 ± 0.07

^a^Values are given as mean ± SEM of three independent experiments. ***p < .001.

##### 2.2.5.3. Caspase 3/9 assay

**Figure 8.**
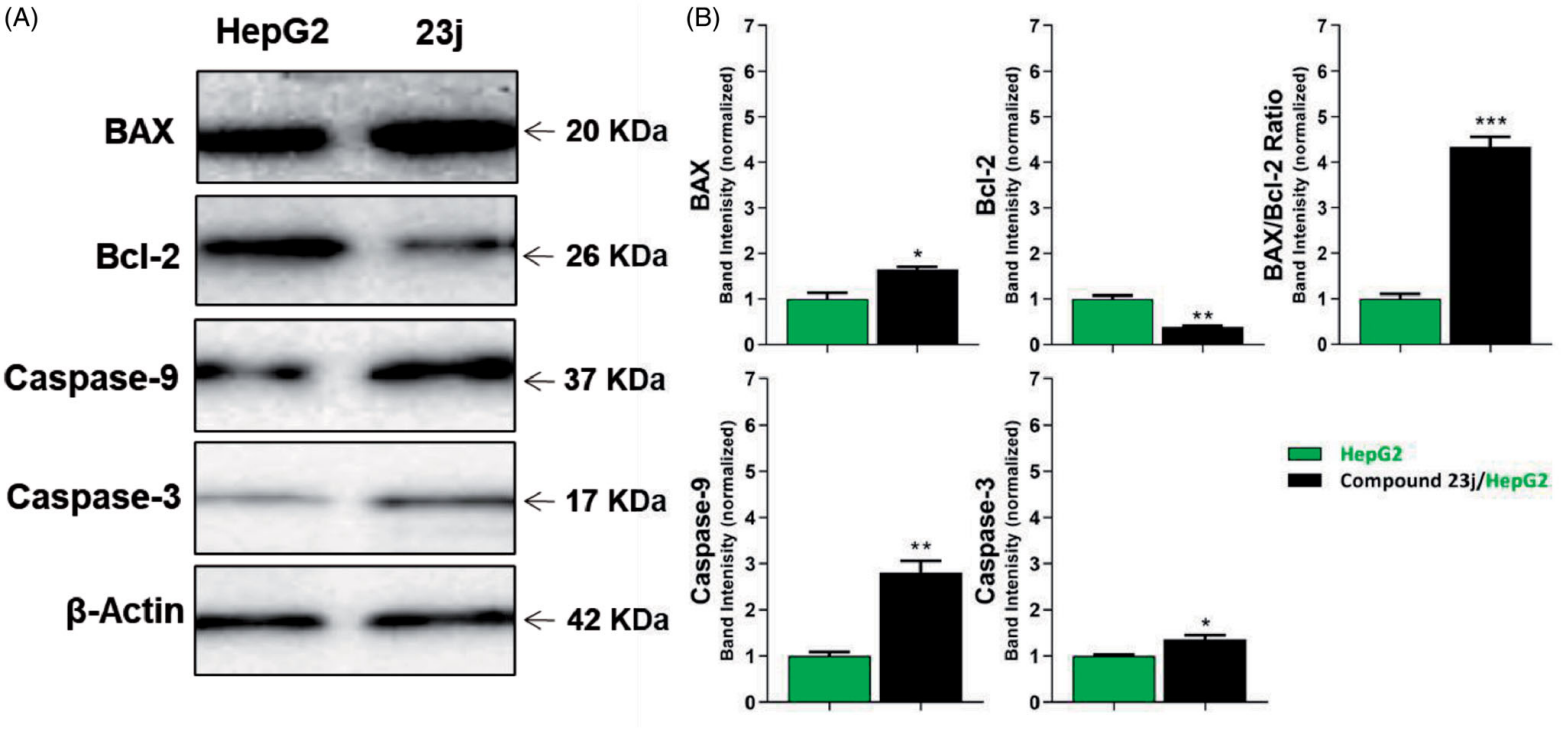
The immunoblotting of BAX, Bcl-2, Caspase-9, and Caspase-3 (Normalized to β-actin). *p < .05, **p < .01, ***p < .001.

To study the effect of compound **23j**, the most promising member, on caspase-3 and caspase-9 levels, western blot technique was carried out. HepG2 cells were treated with **23j** (6.4 µM) for 24 h. The results revealed that compound **23j** produced a significant increase in the cellular levels of caspase-3 (1.36-fold) and caspase-9 (2.80-fold) compared to the control cells (Table 4 and Figure 8).

**Table 4.** Effect of compound **23j** on levels of BAX, Bcl-2, active caspase-9, and active caspase-3 proteins expression

Sample	Protein expression (normalized to β-actin)^a^				
	BAX	Bcl-2	BAX/Bcl-2 ratio	Caspases-9	Caspases-3
HepG2	1.00 ± 0.13	1.00 ± 0.07	1.00 ± 0.10	1.00 ± 0.08	1.00 ± 0.03
23j/HepG2	1.65 ± 0.06*	0.38 ± 0.03**	4.34 ± 0.22***	2.80 ± 0.25**	1.36 ± 0.10*

^a^Values are given as mean ± SEM of three independent experiments. *p < .05, **p < .01 and ***p < .001.

##### 2.2.5.4. Evaluation of BAX and Bcl-2 expressions

BAX and Bcl-2 cellular levels were assessed for compound **23j** after 24 h of its application on HepG2 cells using western blot technique. The results showed that compound **23j** produced an increase of the pro-apoptotic factor BAX by 1.65-fold while anti-apoptotic protein Bcl-2 demonstrated a concentration decreased by 2.63-fold. Moreover, compound **23j** increased BAX/Bcl-2 ratio to be 4.34 which indicates the efficiency of compound **23j** on apoptosis cascade (Table 4 and Figure 8).

### 2.3. Molecular modelling study

#### 2.3.1. Docking study

**Figure 9.**
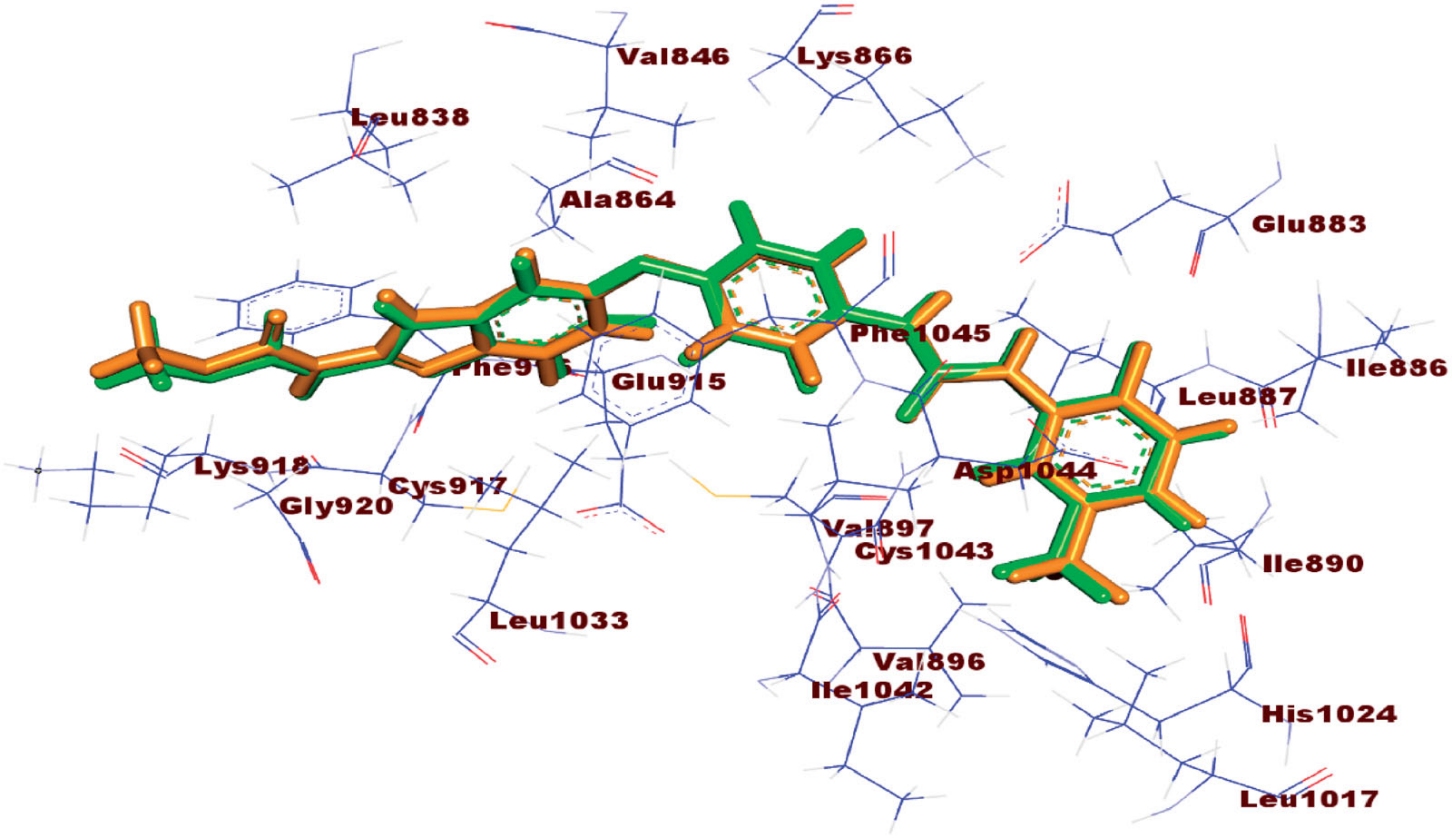
Alignment of the co-crystallised pose (green) and the redocked pose (Orange) of the same ligand inside the protein.

Molecular docking study was accomplished to get further insight into the binding modes and orientations of the newly synthesised compounds into the ATP binding site of VEGFR-2 enzyme. Sorafenib was used as a reference ligand. Validation of the docking procedure was achieved via re-docking of the co-crystallised ligand against the active pocket of VEGFR-2. The results revealed that the RMSD value between the re-docked pose and the co-crystallised one was 1.02. This value revealed the validity of the docking process (Figure 9). The calculated ΔG (binding free energies) of the synthesised compounds and the reference drug against VEGFR-2 were summarised in Table 5.

**Table 5.** The calculated Δ*G* of the synthesised candidates and sorafenib (Δ*G* in kcal/mole).

Comp. No.	ΔG [kcal mol^-1^]	Compound	Δ*G* [kcal mol^–1^]
**23a**	–22.15	**23j**	–23.87
**23b**	–22.86	**23k**	–22.98
**23c**	–23.25	**23l**	–23.26
**23d**	–21.65	**23m**	–22.88
**23e**	–22.97	**23n**	–22.98
**23f**	–21.47	**24a**	–21.30
**23g**	–23.00	**24b**	–21.71
**23h**	–22.37	**24c**	–22.76
**23i**	–23.63	**Sorafenib**	–22.48

**Figure 10.**
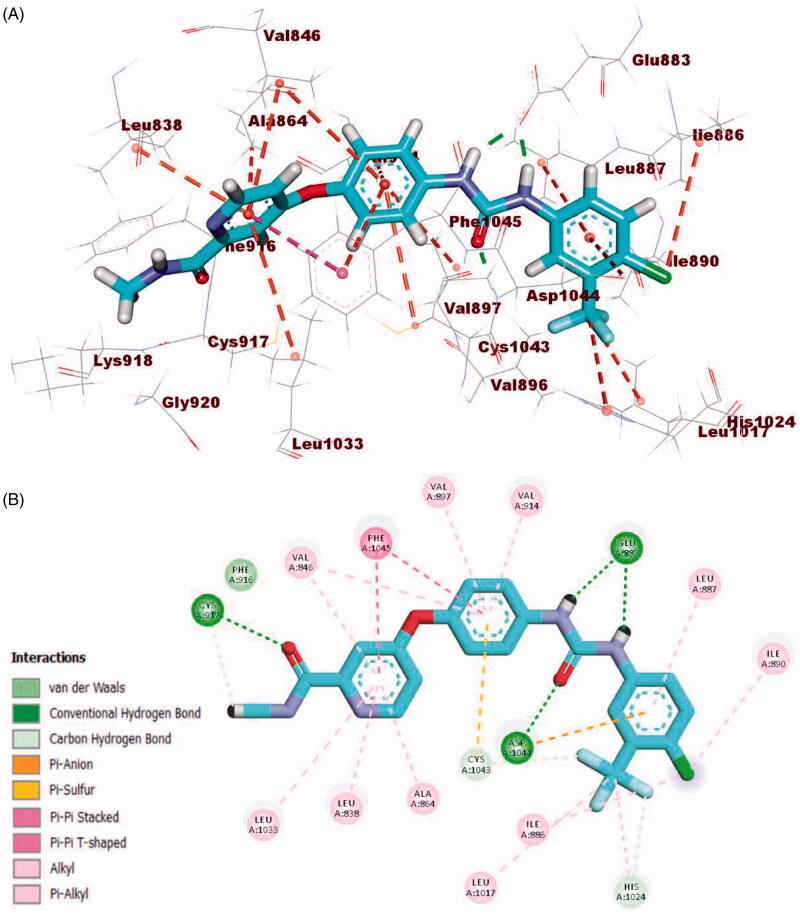
(A**)** 3D binding mode of sorafenib into VEGFR-2. (B) 2D binding mode sorafenib into VEGFR-2.

Sorafenib interactions with the amino acids of the active site have been studied and displayed in 2 D and 3 D style in Figure 10. The proposed binding mode of sorafenib revealed an affinity value of −22.48 kcal/mol. The *N*-methylpicolinamide moiety formed five hydrophobic interactions with Val846, Ala864, Phe1045, Leu1033, and Leu838. Likewise, it formed one hydrogen bond with Cys917. Moreover, the phenyl moiety was buried in the linker region forming five hydrophobic interactions with Phe1045, Cys1043, Val846, Val914, and Val897. Furthermore, the pharmacophore moiety (urea group) formed three hydrogen bonds with the two crucial amino acids Glu883 and Asp1044. Finally, the 1-chloro-2-(trifluoromethyl)benzene moiety conquered the allosteric hydrophobic region forming many hydrophobic and electrostatic interactions with Ile886, Leu887 Leu1017, His1024, Ile890, and Asp1044^34^^,^^58^.

**Figure 11.**
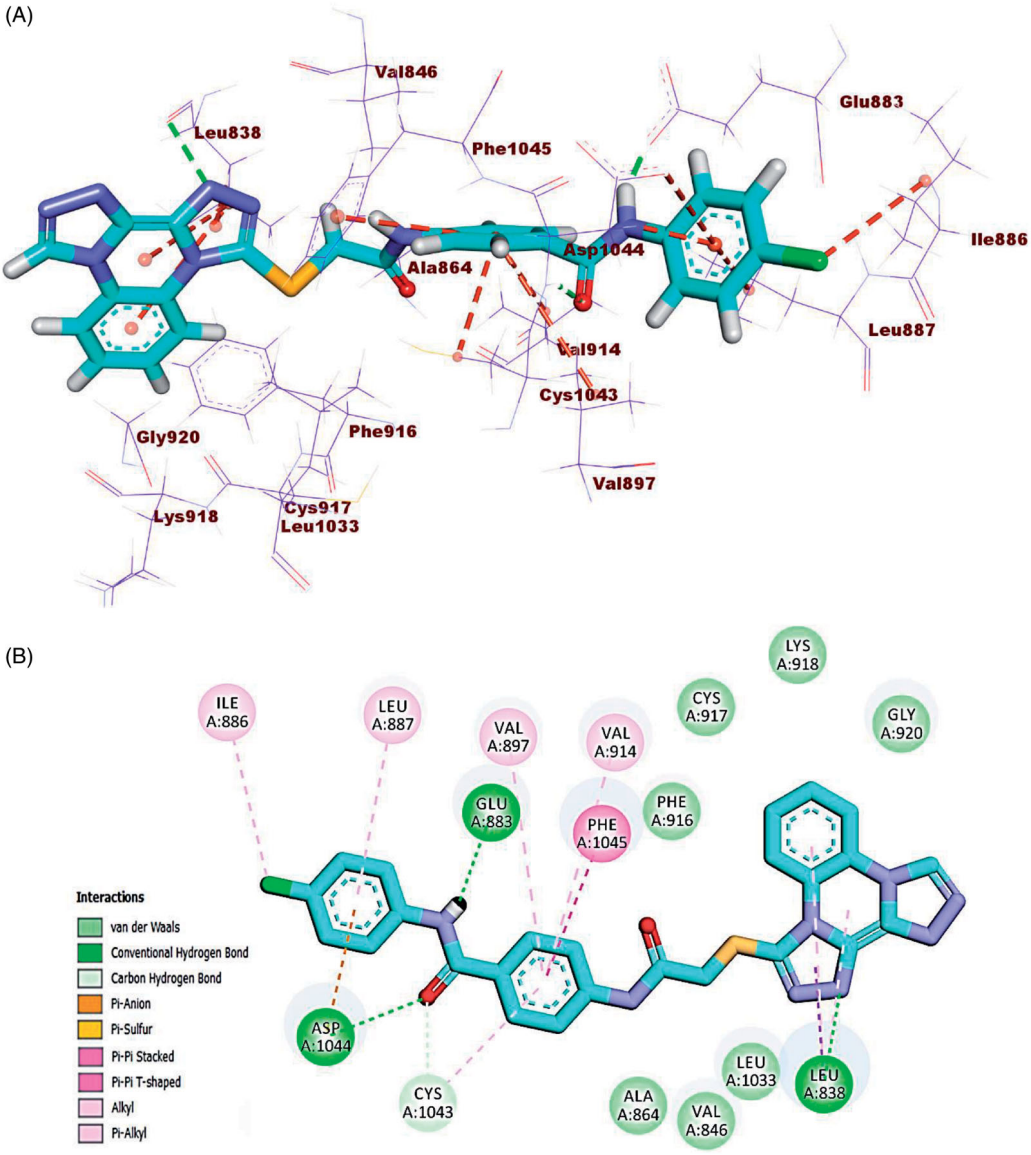
(A) 3D binding mode of compound **23i** into VEGFR-2. (B) 2D binding mode of compound **23i** into VEGFR-2.

Docking simulation of compound **23i** revealed that it has a good fitting into the enzyme active sites with docking score of −23.63 kcal/mol. In DFG region, the amide moiety (pharmacophore) formed two hydrogen bonds with carboxylate moiety of Glu883 (1.50 Å) and NH group of Asp1044 (1.82 Å). Furthermore, the phenyl ring (central spacer) occupied the linker region forming five hydrophobic interactions with Cys1043, Phe1045, Val897, and Val914. Also, the bis([1, 2, 4]triazolo)[4,3-*a*:3′,4′-*c*]quinoxaline moiety occupied the hinge region forming two hydrophobic and one hydrogen bond interactions with Leu838. Additionally, the terminal hydrophobic (4-chlorophenyl) moiety formed three hydrophobic interactions with Ile886 and Leu887 and one electrostatic interaction with Asp1044 (Figure 11).

**Figure 12.**
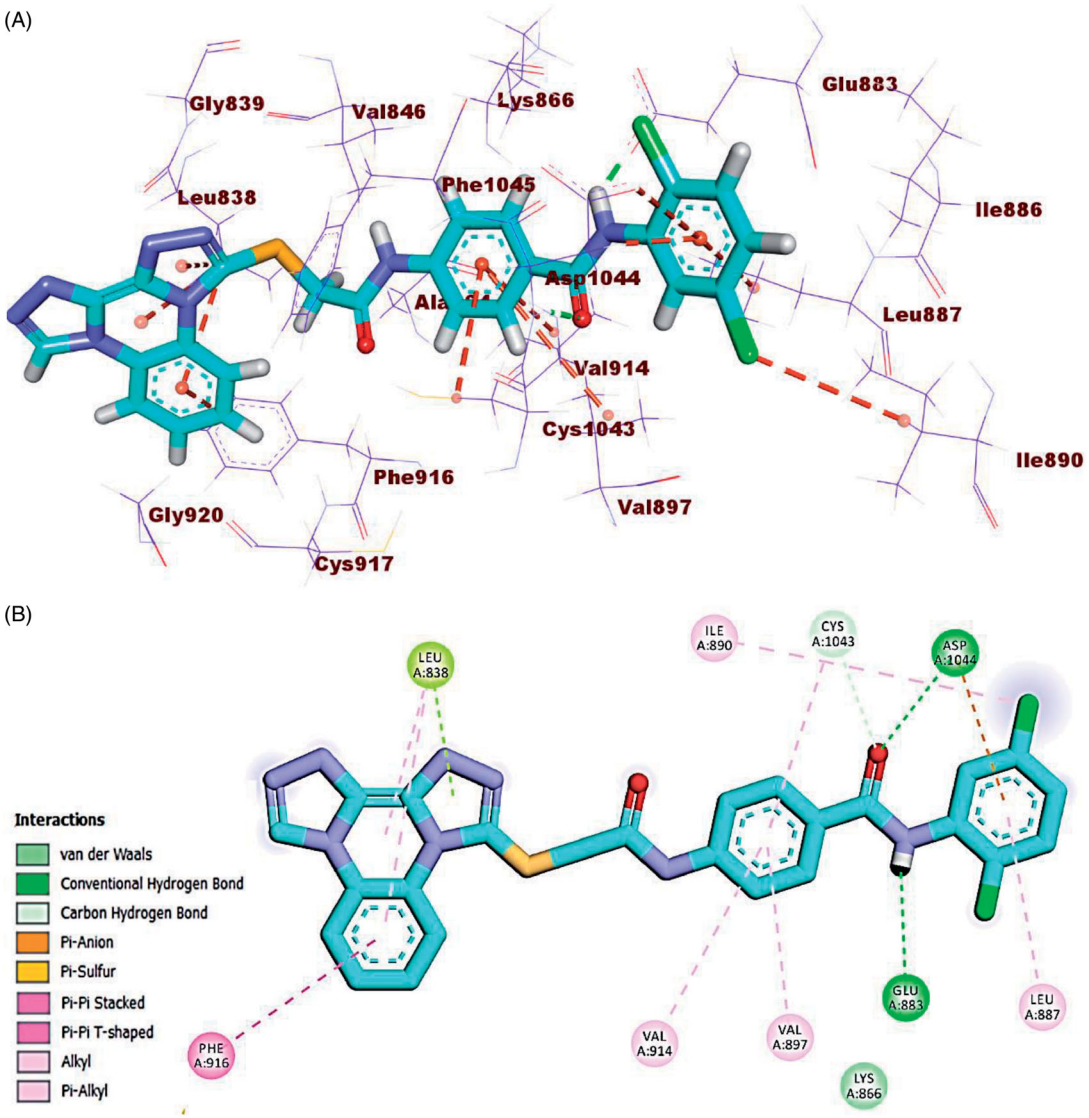
(A) 3D binding mode of compound **23j** into VEGFR-2. (B) 2D binding mode of compound **23j** into VEGFR-2.

The docking findings of compound **23j** revealed affinity value of −23.87 kcal/mol. The amide moiety formed two hydrogen bonds with Glu883 (COO^-^, 1.75 Å) and Asp1044 (NH, 2.20 Å). Additionally, in the hinge region, the docked compound formed four hydrophobic interactions via its bis([1, 2, 4]triazolo)[4,3-*a*:3′,4′-*c*]quinoxaline moiety with Leu838 and Phe916. Also, the central phenyl ring moiety formed three hydrophobic interactions with Val914, Val897, and Cys1043. Finally, the 2,5-dichlorophenyl moiety formed electrostatic and hydrophobic interactions with Asp1044, Ile890, and Leu 887 (Figure 12).

**Figure 13.**
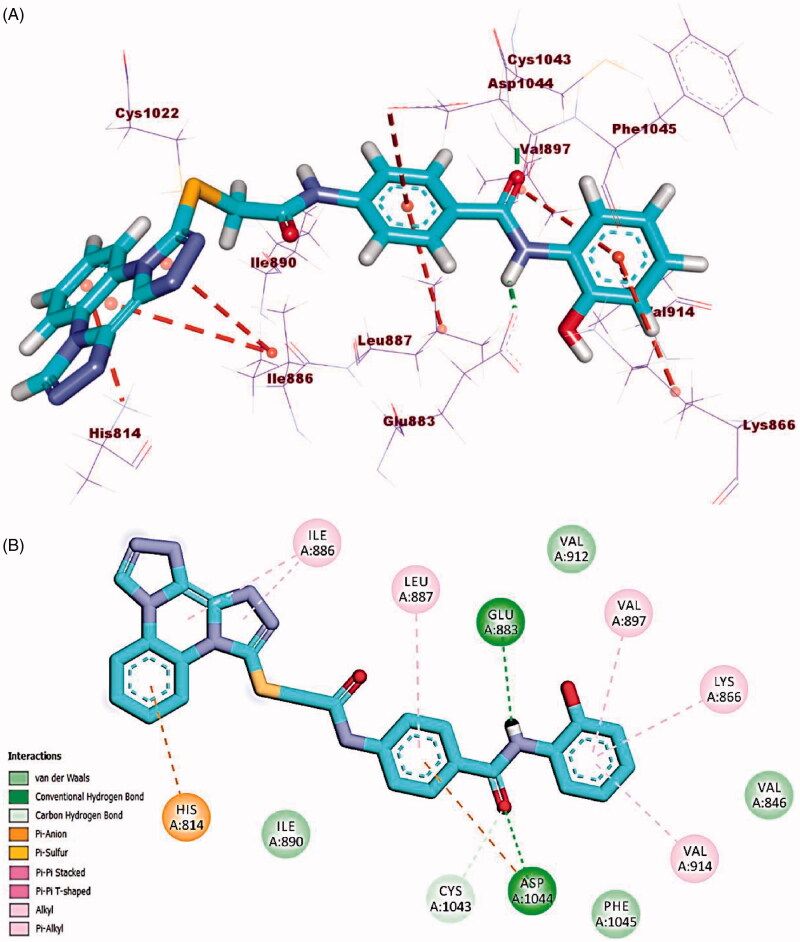
(A) 3D binding mode of compound **23 l** into VEGFR-2. (B) 2D binding mode of compound **23 l** into VEGFR-2.

Compound **23l** (affinity value of −23.26 kcal/mol) combined with the receptor protein as follows; In DFG domain, the amide moiety formed two hydrogen bonds with Glu883 (distance: 2.28 Å) and Asp1044 (distance: 1.76 Å). Moreover, in the hinge region, bis([1, 2, 4]triazolo)[4,3-*a*:3′,4′-*c*]quinoxaline moiety formed three hydrophobic interactions with His814 and Ile886. Also, in the linker region, the central phenyl ring moiety formed two hydrophobic interactions with Asp1044 and Leu887. Additionally, the terminal hydrophobic (2-hydroxyphenyl moiety) formed three hydrophobic interactions with Val897, Val914, and Lys866 (Figure 13).

#### 2.3.2. *In silico* ADME analysis

To investigate pharmacokinetics properties of the prepared compounds, computer aided ADME studies were accomplished using Accelrys Discovery Studio 4.0 software. Sorafenib was used as a reference molecule. These studies include the estimation of certain parameters. 1) Blood brain barrier penetration which measures the ability of molecule to diffuse through blood brain barrier. 2) Absorption level which determines human intestinal absorption (HIA) of a chemical after its oral administration. A well-absorbed compound is one that is absorbed at least 90% into the blood stream^59^^,^^60^. iii) Solubility level in which the solubility of a chemical in water was predicted at 25 °C. iv) CYP2D6 binding which analyzes cytochrome P450 2D6 enzyme inhibition^61^^,^^62^. v) Plasma protein binding which predicts the fraction of drug bound to plasma proteins in the blood^63^. The results were predicted and listed in Table 6.

**Table 6.** ADME screening of the designed compounds

Comp.	BBB level^a^	Solubility level^b^	Absorption level^c^	CYP2D6 prediction^d^	PPB prediction^e^
**23a**	4	2	0	FALSE	FALSE
**23b**	4	2	1	FALSE	FALSE
**23c**	4	2	1	FALSE	FALSE
**23d**	4	2	1	FALSE	FALSE
**23e**	4	2	1	FALSE	FALSE
**23f**	4	1	2	FALSE	FALSE
**23g**	4	2	2	FALSE	FALSE
**23h**	4	1	2	FALSE	FALSE
**23ai**	4	1	2	FALSE	FALSE
**23j**	4	1	2	FALSE	FALSE
**23k**	4	1	1	FALSE	FALSE
**23l**	4	2	2	FALSE	FALSE
**23m**	4	2	2	FALSE	FALSE
**23n**	4	2	3	FALSE	FALSE
**24a**	4	1	2	FALSE	FALSE
**24b**	4	1	2	FALSE	FALSE
**24c**	4	2	3	FALSE	FALSE
**Sorafenib**	4	1	0	FALSE	TRUE

^a^BBB: very high (0), high (1), medium (2), low (3), or very low (4).

^b^Solubility level: very low (1), low (2), good (3), or optimal (4).

^c^Absorption level: good (0), moderate (1), poor (2), or very poor (3).

^d^CYP2D6: inhibitor (TRUE) or non-inhibitor (FALSE).

^e^PBB: less than 90% (FALSE) or more than 90% (TRUE).

The results revealed that the estimated compounds have very low BBB penetration levels.

**Figure 14.**
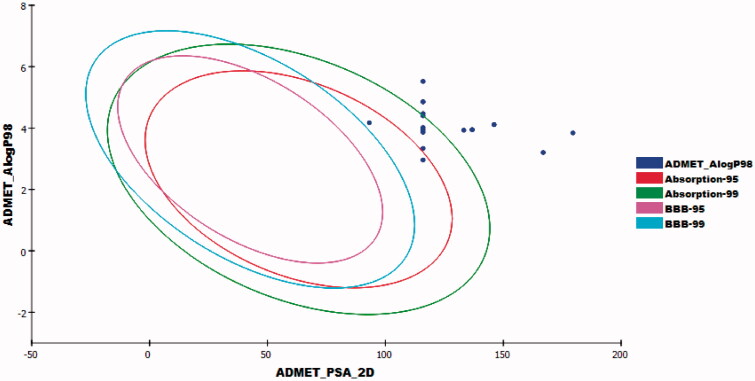
The ADME plot of the synthesised compounds.

Accordingly, the CNS side effects of all compounds were expected to be low. Regarding absorption levels, compound **23a** demonstrated good absorption level, compounds **23b**–**e** and **23k** exhibited moderate absorption levels. On the other hand, compounds **23f**–**j**, **23l**–**n**, and **24a**–**c** showed poor and very poor intestinal absorption. With respect to aqueous solubility, all compounds demonstrated low and very low levels of aqueous solubility. Moreover, the effect on cytochrome P450 2D6 was investigated. The results showed that all examined members were non-inhibitors of CYP2D6. Consequently, liver side effect is not expected upon their administration. The plasma protein binding model exhibited that all compounds were anticipated to bind plasma protein less than 90% (Figure 14).

#### 2.3.3. Toxicity studies

Toxicity profile of the prepared candidates was assessed as stated by the validated and constructed models in Discovery studio software version 4.0^64^^,^^65^. These models involve: 1) FDA rodent carcinogenicity. 2) Carcinogenic potency TD_50_^66^_._ 3) Rat maximum tolerated dose^67^^,^^68^. 4) Developmental toxicity potential^69^^,^^70^. 5) Rat oral LD_50_^71^_._ 6) Rat chronic LOAEL^72^^,^^73^.

Regarding FDA rodent carcinogenicity model, all the tested candidates were forecasted to be non-carcinogenic. For carcinogenic potency TD_50_ rat model, compounds **23a**,**b**, **23 l**,**m**, and **24c** showed TD_50_ values ranging from 33.907 to 44.001 g/kg body weight, which are higher than sorafenib (14.244). With respect to rat maximum tolerated dose model, compounds **23a**–**c**, **23 h**–**n**, and **24a**–**c** demonstrated maximum tolerated dose with a range of 0.099 to 0.391 g/kg body weight which are higher than that of sorafenib **(**0.089 g/kg body weight). On the other hand, compounds **23d**–**g** showed a maximum tolerated dose lower than that of sorafenib with a range of 0.083 to 0.088 g/kg body weight. Additionally, all compounds were predicted to be non-toxic against developmental toxicity potential model. For rat oral LD_50_ model, compounds **23a**–**d**, **23g**, **23i**, **23m**, and **24a**–**c** revealed oral LD_50_ values in a range of 0.864 to 2.477 g/kg body weight which are higher than that of sorafenib **(**0.823 g/kg body weight). For rat chronic LOAEL model, the tested compounds exhibited LOAEL values ranging from 0.064 to 0.329 g/kg body weight. These values are higher than sorafenib (0.005 g/kg body weight) Table 7.

**Table 7.** Toxicity study of the synthesised compounds.

Comp.	FDA rodent carcinogenicity (mouse- female)	Carcinogenic potency TD_50_ (rat)^a^	Rat maximum tolerated dose (feed)^b^	Developmental toxicity potential	Rat oral LD_50_^b^	Rat chronic LOAEL^b^
**23a**	Non-carcinogen	35.802	0.099	Non-toxic	2.209	0.153
**23b**	Non-carcinogen	33.907	0.145	Non-toxic	1.900	0.329
**23c**	Non-carcinogen	0.543	0.114	Non-toxic	1.899	0.206
**23d**	Non-carcinogen	10.185	0.075	Non-toxic	1.978	0.084
**23e**	Non-carcinogen	0.857	0.088	Non-toxic	0.729	0.221
**23f**	Non-carcinogen	0.794	0.083	Non-toxic	0.619	0.192
**23g**	Non-carcinogen	7.641	0.087	Non-toxic	1.071	0.094
**23h**	Non-carcinogen	4.519	0.133	Non-toxic	0.720	0.072
**23i**	Non-carcinogen	4.519	0.133	Non-toxic	1.140	0.067
**23j**	Non-carcinogen	4.056	0.105	Non-toxic	0.546	0.064
**23k**	Non-carcinogen	4.882	0.144	Non-toxic	0.592	0.073
**23l**	Non-carcinogen	37.552	0.362	Non-toxic	0.663	0.134
**23m**	Non-carcinogen	37.552	0.362	Non-toxic	0.864	0.115
**23n**	Non-carcinogen	13.503	0.262	Non-toxic	0.769	0.094
**24a**	Non-carcinogen	5.280	0.143	Non-toxic	2.477	0.155
**24b**	Non-carcinogen	5.080	0.143	Non-toxic	1.754	0.089
**24c**	Non-carcinogen	44.001	0.391	Non-toxic	1.350	0.211
**Sorafenib**	Single-carcinogen	14.244	0.089	Toxic	0.823	0.005

^a^Unit: mg/kg body weight/day.

^b^Unit: g/kg body weight.

## 3. Conclusion

In the present study, a new series of bis([1, 2, 4]triazolo)[4,3-*a*:3′,4′-*c*]quinoxaline derivatives were synthesised as potential cytotoxic agents and VEGFR-2 inhibitors. The anticancer efficiency of these derivatives was evaluated against MCF-7 and HepG2 cancer cell lines. Four compounds **23a** (IC_50_ = 17.7 and 11.3 µM), **23i** (IC_50_ = 18.3 and 10.8 µM), **23j** (IC_50_ = 10.3 and 6.4 µM), **23l** (IC_50_ = 19.4 and 11.3 µM), and **23n** (IC_50_ =16.5 and 12.7 µM) were the most active antiproliferative members against MCF-7 and HepG2, respectively. The *in vitro* VEGFR-2 assay revealed that compounds **23a**, **23d**, **23h**, **23i**, **23j**, **23l**, **23m**, and **23n** exhibited the highest inhibitory activity versus VEGFR-2 with IC_50_ values of 7.1, 11.8, 11.7, 9.4, 3.7, 5.8, 9.7, and 7.4 nM, respectively. SAR revealed that the amide derivatives (compounds **23h** and **23l**) exhibited higher activities than the corresponding diamide derivatives (compounds **24b** and **24c**). Furthermore, compound **23j**, the most potent member, arrested the HepG2 cell growth at G2/M phase and induced apoptosis by 40.12% compared to the control cells (7.07%). Moreover, caspase activation assay was performed for **23j** in HepG2 cell lines. The results showed significant increase of caspase 3 (1.36-fold) and caspase 9 (2.80-fold). BAX and Bcl-2 concentration levels were also evaluated and showed a titre increase of the pro-apoptotic protein BAX (1.65-fold) and a decrease of Bcl-2 (2.63-fold). Additionally, compound **23j** increased BAX/Bcl-2 ratio to be 4.34. Finally, computational physicochemical assessment of the synthesised compounds showed that they have favourable properties with satisfactory drug-like profiles.

## 4. Experimental

### 4.1. Chemistry

All the reagents, chemicals, and apparatus were presented in Supplementary data. Compounds **8**, **9**, **10**, **11**, **12**, **13**, **14**, **16**, **17**, **20a**–**c**, and **21a**–**c** were prepared in accordance with the reported procedures^41^^,^^42^^,^^44^^,^^49^^,^^50^^,^^52^.

#### 4.1.1. General procedure for synthesis of compounds 18a–n

A mixture of **17** (2 mmol) and appropriate amines namely, ethylamine, *n*-butylamine, *sec*-butylamine, *tert*-butylamine, cyclopentylamine, cyclohexylamine, 4-aminoacetophenone, 3-chloroaniline, 4-chloroaniline, 2,5-dichloroaniline, 4-flouroaniline, 2-hydroxyaniline, 4-hydroxyaniline, and 2-hydroxy-4-nitroaniline (2 mmol) was stirred in acetonitrile (50 ml) in the presence of trimethylamine (1 ml) for 8 h. The obtained precipitates were filtered and crystallised from ethanol to give the corresponding derivatives, **18a**–**n**, respectively.

##### 4.1.1.1. 4-(2-Chloroacetamido)-*N*-ethylbenzamide (18a)

Yellow crystal **(**yield, 80%); m.p. = 190 − 192 °C; FT-IR (*ν*_max_, cm^−1^): 3376, 3273 (NH), 2971(C–H aliphatic), 1709, 1636 (C=O), 1605 (C=N); ^1^H NMR (400 MHz, DMSO-d_6_) *δ* 10.49 (s, 1H), 8.36 (s, 1H), 7.80 (d, *J* = 9.7 Hz, 2H), 7.63 (d, *J* = 8.3 Hz, 2H), 4.26 (s, 2H), 3.29 − 3.21 (q, 2H), 1.14 − 1.05 (t, 3H).; ^13^C NMR (101 MHz, DMSO-d_6_) *δ* 165.73 (2C), 165.40 (2C), 141.29, 130.24, 128.37, 44.04, 34.42, 15.43, 15.20; Anal. Calcd. for C_11_H_13_ClN_2_O_2_ (240.68): C, 54.89; H, 5.44; N, 11.64. Found: C, 54.72; H, 5.20; N, 11.46%.

##### 4.1.1.2. *N*-Butyl-4–(2-chloroacetamido)benzamide (18b)

White crystal **(**yield, 85%); m.p. = 170 − 172 °C; FT-IR (*ν*_max_, cm^−1^): 3369, 3263 (NH), 3185 (NH), 3099 (C–H aromatic), 2960 (C–H aliphatic), 1706, 1633 (C=O), 1606 (C=N); ^1^H NMR (400 MHz, DMSO-d_6_) *δ* 10.57 (s, 1H), 8.33 (s, 1H), 7.80 (dd, *J* = 8.8, 1.8 Hz, 2H), 7.66 − 7.61 (m, 2H), 4.27 (s, 2H), 3.25 − 3.18 (m, 2H), 1.51 − 1.43 (m, 2H), 1.33 − 1.26 (m, 2H), 0.89 − 0.84 (m, 3H); ^13^C NMR (101 MHz, DMSO-d_6_) *δ* 165.93, 165.41, 141.30, 130.27, 128.67, 128.31, 119.43, 118.44, 44.44, 44.01, 31.74, 20.12, 14.18; MS (*m*/*z*): 269 (M^+^ + 1, 100%). Anal. Calcd. for C_13_H_17_ClN_2_O_2_ (268.74): C, 58.10; H, 6.38; N, 10.42. Found: C, 58.38; H, 6.61; N, 10.32%.

##### 4.1.1.3. *N*-(Sec-butyl)-4-(2-chloroacetamido)benzamide (18c)

White crystals (yield 78%); m.p. = 180–182 °C; IR (KBr) *ν* cm^−1^: 3307, 3113 (NH), 2969, 2933 (CH aliphatic), 1684, 1625 (C=O); ^1^H NMR (400 MHz, DMSO-d_6_) *δ* 10.54 (s, 1H), 8.06 (d, *J* = 6.1 Hz, 1H), 7.82 (d, *J* = 6.3 Hz, 2H), 7.65 (d, *J* = 7.9 Hz, 2H), 4.28 (s, 2H), 3.89 (dq, *J* = 11.8, 6.8 Hz, 1H), 1.56 − 1.41 (m, 2H), 1.11 (d, *J* = 5.1 Hz, 3H), 0.84 (d, *J* = 4.7 Hz, 3H); ^13^C NMR (101 MHz, DMSO-d_6_) *δ* 165.46, 165.38, 141.25, 130.45, 128.64, 119.33, 118.74, 118.44, 46.76, 44.05, 29.31, 20.69, 11.29; Anal. Calcd. for C_13_H_17_ClN_2_O_2_ (268.74): C, 58.10; H, 6.38; N, 10.42. Found: C, 58.06; H, 6.65; N, 10.29%.

##### 4.1.1.4. *N*-(Tert-butyl)-4-(2-chloroacetamido)benzamide (18d)

Yellow crystals (yield 68%); m.p. = 190–192 °C; IR (KBr) *ν* cm^−1^: 3314 (NH), 2975 (CH aliphatic), 1666, 1628 (C=O), 1606 (C=N); Anal. Calcd. for C_13_H_17_ClN_2_O_2_ (268.74): C, 58.10; H, 6.38; N, 10.42. Found: C, 58.31; H, 6.55; N, 10.59%.

##### 4.1.1.5. 4-(2-Chloroacetamido)-*N*-cyclopentylbenzamide (18e)

White crystals (yield 82%); m.p. = 230–232 °C; IR (KBr) *ν* cm^−1^: 3290 (NH), 2961 (CH aliphatic), 1680, 1624 (C=O); ^1^H NMR (400 MHz, DMSO-d_6_) *δ* 10.77 (s, 1H), 8.20 (d, *J* = 7.3 Hz, 1H), 7.81 (d, *J* = 8.7 Hz, 2H), 7.66 (d, *J* = 8.7 Hz, 2H), 4.31 (s, 2H), 3.03 (qd, *J* = 7.2, 4.2 Hz, 1H), 1.87 − 1.82 (m, 2H), 1.66 (tt, *J* = 4.5, 2.5 Hz, 2H), 1.50 (qd, *J* = 7.7, 6.9, 3.9 Hz, 4H); ^13^C NMR (101 MHz, DMSO-d_6_) *δ* 165.75, 165.42, 141.35, 130.26, 128.76, 119.28, 118.81, 118.36, 51.46, 44.00, 32.54, 31.05, 24.05, 8.93; MS (*m/z*): 281 (M^+^ + 1, 100%); Anal. Calcd. for C_14_H_17_ClN_2_O_2_ (280.75): C, 59.89; H, 6.10; N, 9.98. Found: C, 59.50; H, 6.04; N, 10.25%.

##### 4.1.1.6. 4-(2-Chloroacetamido)-*N*-cyclohexylbenzamide (18f)

White crystals (yield 78%); m.p. = 220–222 °C; FT-IR ((*ν*_max_, cm^−1^): 3286 (NH), 3042 (C–H aromatic), 2938, 2854 (C–H aliphatic), 1681, 1624 (C=O), 1610 (C=N); ^1^H NMR (400 MHz, DMSO-d_6_) *δ* 10.52 (s, 1H), 8.08 (d, *J* = 7.9 Hz, 1H), 7.83 − 7.77 (m, 2H), 7.65 − 7.59 (m, 2H), 4.26 (s, 2H), 3.72 (s, 1H), 1.78 (d, *J* = 7.1 Hz, 2H), 1.71 (d, *J* = 8.4 Hz, 2H), 1.58 (d, *J* = 12.8 Hz, 1H), 1.27 (dd, *J* = 11.4, 8.6 Hz, 4H), 1.12 (q, *J* = 10.8, 9.3 Hz, 1H); ^13^C NMR (101 MHz, DMSO-d_6_) *δ* 165.38, 165.15, 141.24, 130.41, 129.07, 128.80, 128.47, 119.35, 118.86, 118.41, 48.79, 48.66, 44.42, 44.02, 25.63; Anal. Calcd. for C_15_H_19_ClN_2_O_2_ (294.77): C, 61.12; H, 6.50; N, 9.50. Found: C, 60.88; H, 6.39; N, 9.25%.

##### 4.1.1.7. *N*-(4-Acetylphenyl)-4-(2-chloroacetamido)benzamide (18g)

White crystals (yield 80%); m.p. = 250–252 °C; FT-IR (*ν*_max_, cm^−1^): 3326 (NH), 3062 (C–H aromatic), 1674, 1655 (C=O), 1596 (C=N); ^1^H NMR (400 MHz, DMSO-d_6_) *δ* 10.61 (s, 1H), 10.46 (s, 1H), 7.98 − 7.90 (m, 6H), 7.72 (s, 2H), 4.29 (s, 2H), 2.53 (s, 3H); ^13^C NMR (101 MHz, DMSO-d_6_) *δ* 197.08, 165.69(2 C), 165.57(2 C), 144.14, 142.15, 132.33, 129.90, 129.85, 129.60, 129.26, 119.97, 119.66, 118.65, 44.05, 27.16; Anal. Calcd. for C_17_H_15_ClN_2_O_3_ (330.76): C, 61.73; H, 4.57; N, 8.47. Found: C, 61.44; H, 4.39; N, 8.29%.

##### 4.1.1.8. 4-(2-Chloroacetamido)-*N*-(3-chlorophenyl)benzamide (18h)

Yellow crystal **(**yield, 75%); m.p. = 190 − 192 °C; FT-IR (v max, cm^−1^): 3291 (NH), 1676, 1644 (C=O), 1593 (C=N); ^1^H NMR (400 MHz, DMSO-d_6_) *δ* 10.60 (s, 1H), 10.30 (s, 1H), 7.95 − 7.94 (m, 2H), 7.93 (d, *J* = 1.9 Hz, 1H), 7.74 − 7.68 (m, 3H), 7.36 (t, *J* = 8.1 Hz, 1H), 7.14 − 7.12 (m, 1H), 4.29 (s, 2H); ^13^C NMR (101 MHz, DMSO-d_6_) *δ* 165.56, 165.53, 142.07, 141.20, 141.14, 133.33, 129.86, 129.41, 129.12, 120.24, 119.93, 119.03, 118.66, 118.59, 44.05; Anal. Calcd. for C_15_H_12_Cl_2_N_2_O_2_ (323.17): C, 55.75; H, 3.74; N, 8.67. Found: C, 55.64; H, 3.63; N, 8.45%.

##### 4.1.1.9. 4-(2-Chloroacetamido)-*N*-(4-chlorophenyl)benzamide (18i)

Yellow crystal **(**yield, 83%); m.p. = 228 − 230 °C; FT-IR (*ν*_max_, cm^−1^): 3324 (NH), 1666 (C=O amide), 1595 (C=N); ^1^H NMR (400 MHz, DMSO-d_6_) *δ* 10.59 (s, 1H), 10.27 (s, 1H), 7.93 (d, *J* = 8.6 Hz, 2H), 7.79 (d, *J* = 8.9 Hz, 2H), 7.72 (d, *J* = 8.7 Hz, 2H), 7.38 (d, *J* = 8.8 Hz, 2H), 4.29 (s, 2H); ^13^C NMR (101 MHz, DMSO-d_6_) *δ* 165.53 (2C), 165.36 (2C), 141.98, 138.67, 130.01, 129.36 (2C), 129.08, 128.58, 128.30, 127.56, 118.63, 44.05; Anal. Calcd. for C_15_H_12_Cl_2_N_2_O_2_ (322.17): C, 55.75; H, 3.74; N, 8.67. Found: C, 55.59; H, 3.57; N, 8.40%.

##### 4.1.1.10. 4-(2-Chloroacetamido)-*N*-(2,5-dichlorophenyl)benzamide (18j)

Yellow crystal **(**yield, 77%); m.p. = 195 − 197 °C; FT-IR (*ν*_max_, cm^−1^): 3277, 3104 (NH), 3033 (C–H aromatic), 1770, 1679 (C=O), 1606 (C=N); ^1^H NMR (400 MHz, DMSO-d_6_) *δ* 10.63 (s, 1H), 10.03 (s, 1H), 7.96 (d, *J* = 8.7 Hz, 2H), 7.73 (dd, *J* = 5.6, 3.0 Hz, 3H), 7.57 (d, *J* = 8.6 Hz, 1H), 7.34 (dd, *J* = 8.6, 2.5 Hz, 1H), 4.29 (s, 2H); ^13^C NMR (101 MHz, DMSO-d_6_) *δ* 165.60 (2 C), 165.26, 142.26, 136.86, 131.87, 131.81, 130.80, 129.48 (2C), 129.19, 128.97 (2C), 118.75, 44.02; Anal. Calcd. for C_15_H_11_Cl_3_N_2_O_2_ (357.61): C, 50.38; H, 3.10; N, 7.83. Found: C, 50.42; H, 3.18; N, 7.64%.

##### 4.1.1.11. 4-(2-Chloroacetamido)-*N*-(4-fluorophenyl)benzamide (18k)

White crystal **(**yield, 70%); m.p. = 238–240 °C; FT-IR (*ν*_max_, cm^−1^)**;** 3325 (NH), 1666, 1643 (C=O), 1611 (C=N); ^1^H NMR (400 MHz, DMSO-d_6_) *δ* 10.66 (s, 1H), 10.23 (s, 1H), 7.95 (d, *J* = 7.0 Hz, 2H), 7.82 − 7.70 (m, 4H), 7.17 (s, 2H), 4.31 (t, *J* = 2.8 Hz, 2H); ^13^C NMR (101 MHz, DMSO-d_6_) *δ* 165.54, 165.21, 141.91, 136.06, 136.02, 130.12, 129.28, 129.04, 122.15, 119.45, 119.02, 118.63, 116.11, 115.88, 44.06; Anal. Calcd. for C_15_H_12_ClFN_2_O_2_ (306.72): C, 58.74; H, 3.94; N, 9.13. Found: C, 58.55; H, 3.89; N, 9.05%.

##### 4.1.1.12. 4-(2-Chloroacetamido)-*N*-(2-hydroxyphenyl)benzamide (18l)

Brown crystal **(**yield, 65%); m.p. = 192 − 194 °C; FT-IR (*ν*_max_, cm^−1^): 3262, 3190 (NH), 3064 (C–H aromatic), 2952 (C–H aliphatic), 1735, 1683 (C=O), 1600 (C=N); ^1^H NMR (400 MHz, DMSO-d_6_) *δ* 10.70 (s, 1H), 10.00 (s, 1H), 9.49 (s, 1H), 8.11–8.02 (m, 2H), 8.02–7.91 (m, 2H), 7.12 − 6.73 (m, 4H), 4.32 (s, 2H); ^13^C NMR (101 MHz, DMSO-d_6_) *δ* 165.77, 165.24, 165.07, 163.82 (2C), 149.77, 144.89, 143.80, 141.98, 131.61, 129.69 (2C), 126.39, 124.39, 44.07; Anal. Calcd. for C_15_H_13_ClN_2_O_3_ (304.72): C, 59.12; H, 4.30; N, 9.19. Found: C, 59.01; H, 4.14; N, 8.92%.

##### 4.1.1.13. 4-(2-Chloroacetamido)-*N*-(4-hydroxyphenyl)benzamide (18m)

Brown crystal **(**yield, 85%); m.p. >300 °C; FT-IR (*ν*_max_, cm^−1^): 3309, 3104 (NH), 3033 (C–H aromatic), 1770, 1671 (C=O), 1607 (C=N); ^1^H NMR (400 MHz, DMSO-d_6_) *δ* 10.81 (s, 1H), 10.29 (s, 1H), 9.95 (s, 1H), 8.11 (d, *J* = 9.2 Hz, 2H), 7.85 − 7.80 (m, 2H), 7.50 (d, *J* = 8.4 Hz, 2H), 6.72 (d, *J* = 8.3 Hz, 2H), 4.30 (d, *J* = 4.1 Hz, 2H); Anal. Calcd. for C_15_H_13_ClN_2_O_3_ (304.73): C, 59.12; H, 4.30; N, 9.19. Found: C, 59.32; H, 4.15; N, 8.95%.

##### 4.1.1.14. 4-(2-Chloroacetamido)-*N*-(2-hydroxy-4-nitrophenyl)benzamide (18n)

Greenish yellow crystal **(**yield, 60%); m.p. = 198–200 °C; FT-IR (*ν*_max_, cm^−1^): 3596, 3409 (NH), 3074 (C–H aromatic), 1700, 1652 (C=O), 1602 (C=N); ^1^H NMR (400 MHz, DMSO-d_6_) *δ* 11.15 (s, 1H), 10.66 (s, 1H), 9.51 (s, 1H), 8.22 (d, *J* = 8.3 Hz, 1H), 7.96 (d, *J* = 8.3 Hz, 2H), 7.85 − 7.67 (m, 4H), 4.30 (s, 2H); ^13^C NMR (101 MHz, DMSO-d_6_) *δ* 165.61, 165.05 (2C), 148.61 (2C), 143.65, 142.42, 133.57, 129.12 (2 C), 122.04, 118.83, 115.16, 109.46, 44.07; Anal. Calcd. for C_15_H_12_ClN_3_O_5_ (349.72): C, 51.52; H, 3.46; N, 12.02. Found: C, 51.33; H, 3.57; N, 11.87%.

#### 4.1.2. General procedure for the synthesis of compounds 22a–c

A mixture of **17** (2 mmol) and the acid hydrazides **21a**–**c** namely, 2-chlorobenzohydrazide, 3-chlorobenzohydrazide, and 2-hydroxybenzohyazide (2 mmol) was allowed to stir in acetonitrile (50 ml) in the presence of trimethylamine (1 ml) for 8 h. The precipitated products were filtered, dried, and crystalised from ethanol to give the diamide intermediated, **22a**–**c**, respectively.

##### 4.1.2.1. 2-Chloro-*N*-(4-(2-(2-chlorobenzoyl)hydrazine-1-carbonyl)phenyl)acetamide (22a)

White crystal (yield, 85%); m.p. = 230 − 232 °C; FT-IR (*ν*_max_, cm^−1^): 3409, 3249 (NH), 3074 (C–H aromatic), 1673, 1650 (C=O), 1603 (C=N); ^1^H NMR (400 MHz, DMSO-d_6_) *δ* 10.65 (s, 1H), 10.55 (s, 1H), 10.38 (s, 1H), 7.92 (d, *J* = 7.5 Hz, 2H), 7.72 (d, *J* = 7.6 Hz, 2H), 7.56 − 7.46 (m, 4H), 4.33 − 4.28 (m, 2H); Anal. Calcd. for C_16_H_13_Cl_2_N_3_O_3_ (366.19): C, 52.48; H, 3.58; N, 11.47. Found: C, 52.72; H, 3.48; N, 11.34%.

##### 4.1.2.2. 2-Chloro-*N*-(4-(2-(3-chlorobenzoyl)hydrazine-1-carbonyl)phenyl)acetamide (22b)

Yellow crystal **(**yield, 80%); m.p. = 235 − 237 °C; FT-IR (*ν*_max_, cm^−1^): 3409, 3283 (NH), 3074 (C–H aromatic), 1676, 1645 (C=O), 1604 (C=N); ^1^H NMR (400 MHz, DMSO-d_6_) *δ* 10.63 (s, 2H), 10.51 (s, 1H), 7.91 (dd, *J* = 19.0, 9.7 Hz, 4H), 7.74 − 7.66 (m, 3H), 7.58 (d, *J* = 6.4 Hz, 1H), 4.30 (t, *J* = 2.4 Hz, 2H); ^13^C NMR (101 MHz, DMSO-d_6_) *δ* 165.67, 165.58 (2C), 165.00, 142.13, 134.92, 133.83, 129.11, 128.88, 127.83 (2C), 127.59, 119.58, 119.13, 118.75, 44.06; Anal. Calcd. for C_16_H_13_Cl_2_N_3_O_3_ (366.19): C, 52.48; H, 3.58; N, 11.47. Found: C, 52.71; H, 3.62; N, 11.60%.

##### 4.1.2.3. 2-Chloro-*N*-(4–(2-(2-hydroxybenzoyl)hydrazine-1-carbonyl)phenyl)acetamide (22c)

Yellow crystal **(**yield, 80%); m.p. = 233 − 235 °C; FT-IR (*ν*_max_, cm^−1^): 3272 (NH), 1687, 1661 (C=O), 1600 (C=N); ^1^H NMR (400 MHz, DMSO-d_6_) *δ* 11.97 (s, 1H), 10.69 (d, *J* = 9.7 Hz, 2H), 10.63 (s, 1H), 7.93 (d, *J* = 8.0 Hz, 3H), 7.75 (d, *J* = 8.1 Hz, 2H), 7.44 (d, *J* = 8.6 Hz, 1H), 6.96 (q, *J* = 8.4, 7.4 Hz, 2H), 4.32 (s, 2H); ^13^C NMR (101 MHz, DMSO-d_6_) *δ* 168.25, 165.62, 165.47, 159.76, 159.72, 142.21, 129.14, 128.87, 128.65, 127.68, 119.58, 119.16, 118.79, 118.12, 114.98, 44.07; Anal. Calcd. for C_16_H_14_ClN_3_O_4_ (347.76): C, 55.26; H, 4.06; N, 12.08. Found: C, 55.45; H, 3.83; N, 11.90%.

#### 4.1.3. General procedure for the synthesis of compounds 23a–n

A mixture of potassium salt of bis([1, 2, 4]triazolo)[4,3-*a*:3′,4′-*c*]quinoxaline-3-thiol **14** (0.5 g, 0.001 mol) and 4-(2-chloroacetamido)-*N*-(substituted)benzamide **18a**–**n** (0.001 mol) in DMF (50 ml) was heated on a water bath for 6 h. After cooling to room temperature, the reaction mixture was poured on crushed ice. The obtained precipitates were collected by filtration, dried, and crystalised from ethanol to give the target compounds **23a**–**n**.

##### 4.1.3.1. 4-(2-(Bis([1, 2, 4]triazolo)[4,3-*a*:3',4'-*c*]quinoxalin-3-ylthio)acetamido)-*N*-ethylbenzamide (23a)

White crystal (yield, 70%); m.p. = 260–262 °C. FT-IR (*ν*_max_, cm^−1^): 3267 (NH), 1700, 1655 (C=O), 1596 (C=N); ^1^H NMR (700 MHz, DMSO-d_6_) δ 10.69 (s, 1H), 10.12 (s, 1H), 8.35 (dq, *J* = 11.5, 3.7, 3.1 Hz, 2H), 7.86 (dd, *J* = 8.1, 3.1 Hz, 1H), 7.85 − 7.80 (m, 2H), 7.75 − 7.69 (m, 2H), 7.69 − 7.65 (m, 1H), 7.63 (dt, *J* = 10.1, 5.1 Hz, 1H), 4.41 (d, *J* = 3.4 Hz, 2H), 3.32 − 3.25 (m, 2H), 1.16 − 1.09 (m, 3H); ^13^C NMR (176 MHz, DMSO-d_6_) *δ* 166.67, 165.83, 151.65, 141.85 (2C), 138.29, 135.59, 129.91, 128.73, 128.52 (2C), 128.34, 128.32 (2C), 124.07, 118.76 (2C), 117.13, 34.45 (2C), 15.33; Anal. Calcd. for C_21_H_18_N_8_O_2_S (446.49): C, 56.49; H, 4.06; N, 25.10. Found: C, 56.21; H, 4.15; N, 24.95%.

##### 4.1.3.2. 4-(2-(Bis([1, 2, 4]triazolo)[4,3-*a*:3',4'-*c*]quinoxalin-3-ylthio)acetamido)-*N*-butylbenzamide (23b)

Yellowish white crystal (yield, 70%); m.p. = 252–254 °C. FT-IR (*ν*_max_, cm^−1^): 3299 (NH), 2928 (CH aliphatic), 1676, 1631 (C=O); ^1^H NMR (700 MHz, DMSO-d_6_) *δ* 10.68 (s, 1H), 10.02 (s, 1H), 8.65 − 8.58 (m, 1H), 8.50 − 8.45 (m, 1H), 8.33 (q, *J* = 4.9 Hz, 1H), 7.86 − 7.79 (m, 2H), 7.75 (p, *J* = 7.6, 7.2 Hz, 2H), 7.65 (d, *J* = 8.4 Hz, 2H), 4.57 (s, 2H), 3.25 (p, *J* = 6.6, 5.4 Hz, 2H), 1.50 (q, *J* = 7.5 Hz, 2H), 1.33 (q, *J* = 8.0 Hz, 2H), 0.94 − 0.88 (m, 3H); ^13^C NMR (176 MHz, DMSO-d_6_) *δ* 165.93, 165.90, 147.72, 142.11, 141.56, 139.35, 138.78, 130.08, 128.54, 128.37, 128.35, 124.09, 123.20, 118.76, 118.58, 118.07, 39.29, 38.88, 31.78, 20.15, 14.22; MS (*m/z*): 475 (M^+^ + 1, 50%); Anal. Calcd. for C_23_H_22_N_8_O_2_S (474.54): C, 58.21; H, 4.67; N, 23.61. Found: C, 58.05; H, 4.60; N, 23.55%.

##### 4.1.3.3. 4-(2-(Bis([1, 2, 4]triazolo)[4,3-*a*:3',4'-*c*]quinoxalin-3-ylthio)acetamido)-*N*-(sec-butyl)benzamide (23c)

White crystal (yield, 72%); m.p. = 245–247 °C. FT-IR (*ν*_max_, cm^−1^): 3266, 3127 (NH), 2965, 2910 (CH aliphatic), 1701, 1636 (C=O), 1605 (C=N); ^1^H NMR (700 MHz, DMSO-d_6_) *δ* 10.68 (s, 1H), 10.01 (s, 1H), 8.58 (d, *J* = 8.1 Hz, 1H), 8.45 (d, *J* = 7.9 Hz, 1H), 8.04 (d, *J* = 8.4 Hz, 1H), 7.83 (d, *J* = 8.3 Hz, 2H), 7.73 (p, *J* = 7.6 Hz, 2H), 7.65 (d, *J* = 8.3 Hz, 2H), 4.57 (s, 2H), 3.90 (dd, *J* = 14.5, 7.5 Hz, 1H), 1.51 (ddq, *J* = 29.9, 15.4, 8.3, 7.7 Hz, 2H), 1.13 (d, *J* = 6.6 Hz, 3H), 0.87 (d, *J* = 7.5 Hz, 3H); ^13^C NMR (176 MHz, DMSO-d_6_) *δ* 165.88, 165.48, 147.74, 142.07, 141.52, 139.32, 138.77, 130.27, 128.63 (2C), 128.34, 128.32, 124.04, 123.15, 118.69 (2C), 118.56, 118.03, 46.82, 38.89, 29.34, 20.77, 11.25; MS (*m/z*): 475 (M^+^ + 1, 60%); Anal. Calcd. for C_23_H_22_ClN_8_O_2_S (474.54): C, 58.21; H, 4.67; N, 23.61. Found: C, 57.98; H, 4.62; N, 23.57%.

##### 4.1.3.4. 4-(2-(Bis([1, 2, 4]triazolo)[4,3-*a*:3',4'-*c*]quinoxalin-3-ylthio)acetamido)-*N*-(tert-butyl)benzamide (23d)

Faint yellow crystal (yield, 75%); m.p. = 248–250 °C. FT-IR (*ν*_max_, cm^−1^): 3439, 3104 (NH), 3051 (CH aromatic), 2968, 2929 (CH aliphatic), 1651 (C=O), 1600 (C=N); ^1^H NMR (700 MHz, DMSO-d_6_) *δ* 10.67 (s, 1H), 10.03 (s, 1H), 8.61 (d, *J* = 8.0 Hz, 1H), 8.50 − 8.47 (m, 1H), 7.79 (t, *J* = 6.9 Hz, 3H), 7.76 (d, *J* = 8.1 Hz, 2H), 7.63 (d, *J* = 8.1 Hz, 2H), 4.57 (s, 2H), 1.38 (s, 9H); ^13^C NMR (176 MHz, DMSO-d_6_) *δ* 166.06, 165.85, 147.74, 142.13, 141.39, 139.37, 138.78, 131.16, 128.74 (2C), 128.38, 128.37, 124.12, 123.23, 118.60, 118.57 (2C), 118.08, 51.16, 38.87, 29.11 (3C); MS (*m/z*): 475 (M^+^ + 1, 100%); Anal. Calcd. for C_23_H_22_N_8_O_2_S (474.54): C, 58.21; H, 4.67; N, 23.61. Found: C, 58.05; H, 4.55; N, 23.44%.

##### 4.1.3.5. 4-(2-(Bis([1, 2, 4]triazolo)[4,3-*a*:3',4'-*c*]quinoxalin-3-ylthio)acetamido)-*N*-cyclopentylbenzamide (23e)

Yellow crystal (yield, 77%); m.p. = 244–246 °C. FT-IR (*ν*_max_, cm^−1^): 3262, 3124 (NH), 2950, 2905 (CH aliphatic), 1699, 1636 (C=O), 1607 (C=N); ^1^H NMR (700 MHz, DMSO-d_6_) *δ* 10.68 (s, 1H), 10.02 (s, 1H), 8.61 (dd, *J* = 8.0, 1.6 Hz, 1H), 8.47 (dd, *J* = 7.8, 1.8 Hz, 1H), 8.17 (d, *J* = 7.3 Hz, 1H), 7.83 (d, *J* = 8.6 Hz, 2H), 7.76 (ddd, *J* = 8.9, 7.6, 1.6 Hz, 2H), 7.65 (d, *J* = 8.6 Hz, 2H), 4.57 (s, 2H), 4.21 (q, *J* = 7.1 Hz, 1H), 1.88 (dtd, *J* = 12.0, 8.8, 7.9, 4.4 Hz, 2H), 1.70 (dt, *J* = 9.6, 5.3 Hz, 2H), 1.56 − 1.51 (m, 4H); ^13^C NMR (176 MHz, DMSO-d_6_) *δ* 165.88, 165.73, 147.73, 142.12, 141.53, 139.36, 138.78, 130.15, 128.70 (2C), 128.37, 128.35, 124.10, 123.21, 118.66 (2C), 118.59, 118.06, 51.34, 38.87, 32.61 (2C), 24.09 (2C); MS (*m/z*): 487 (M^+^ + 1, 65%); Anal. Calcd. for C_24_H_22_N_8_O_2_S (486.55): C, 59.25; H, 4.56; N, 23.03. Found: C, 59.08; H, 4.47; N, 22.89%.

##### 4.1.3.6. 4-(2-(Bis([1, 2, 4]triazolo)[4,3-*a*:3',4'-*c*]quinoxalin-3-ylthio)acetamido)-*N*-cyclohexylbenzamide (23f)

White crystal (yield, 70%); m.p. = 270–272 °C. FT-IR (*ν*_max_, cm^−1^): 3269, 3131 (NH), 2930 (CH aliphatic), 1700, 1633 (C=O), 1607 (C=N); ^1^H NMR (700 MHz, DMSO-d_6_) *δ* 10.68 (s, 1H), 10.02 (s, 1H), 8.61 (dd, *J* = 7.9, 1.6 Hz, 1H), 8.47 (dd, *J* = 7.7, 1.8 Hz, 1H), 8.09 (d, *J* = 7.9 Hz, 1H), 7.82 (d, *J* = 8.6 Hz, 2H), 7.76 (dtd, *J* = 16.4, 7.5, 1.5 Hz, 2H), 7.65 (d, *J* = 8.7 Hz, 2H), 4.57 (s, 2H), 3.75 (ddt, *J* = 15.1, 11.0, 5.3 Hz, 1H), 1.81 (dt, *J* = 8.6, 4.1 Hz, 2H), 1.74 (dt, *J* = 11.4, 4.1 Hz, 2H), 1.64 − 1.59 (m, 1H), 1.30 (dd, *J* = 11.3, 8.6 Hz, 4H), 1.13 (qt, *J* = 8.4, 4.3 Hz, 1H); ^13^C NMR (176 MHz, DMSO-d_6_) *δ* 165.88, 165.14, 147.73, 142.12, 141.54, 139.36, 138.78, 130.21, 128.69 (2C), 128.37, 128.35, 124.10, 123.22, 118.67 (2C), 118.59, 118.07, 48.73, 38.87, 32.95 (2C), 25.76, 25.45 (2C); MS (*m/z*): 501 (M^+^ + 1, 100%); Anal. Calcd. for C_25_H_24_N_8_O_2_S (500.58): C, 59.99; H, 4.83; N, 22.39. Found: C, 59.85; H, 4.76; N, 22.11%.

##### 4.1.3.7. *N*-(4-Acetylphenyl)-4-(2-(bis([1, 2, 4]triazolo)[4,3-*a*:3',4'-*c*]quinoxalin-3-ylthio)acetamido)benzamide (23g)

Pale white crystal (yield, 80%); m.p. = 275–277 °C. FT-IR (*ν*_max_, cm^−1^): 3300 (NH), 1655 (C=O), 1593 (C=N); ^1^H NMR (700 MHz, DMSO-d_6_) *δ* 10.77 (s, 1H), 10.14 (s, 1H), 7.97 (d, *J* = 8.6 Hz, 2H), 7.81 (dd, *J* = 8.0, 1.5 Hz, 1H), 7.78 − 7.76 (m, 2H), 7.72 (d, *J* = 8.7 Hz, 2H), 7.59 − 7.57 (m, 1H), 7.54 (dd, *J* = 8.5, 1.3 Hz, 1H), 7.40 − 7.37 (m, 1H), 7.36 − 7.34 (m, 2H), 7.10 (t, *J* = 7.4 Hz, 1H), 5.19 (s, 2H), 2.49 (s, 3H); ^13^C NMR (176 MHz, DMSO-d_6_) *δ* 165.80, 165.23, 157.97, 154.85, 142.00, 139.71, 133.48, 132.47 (2C), 130.20 (2C), 130.04, 129.29, 129.23 (2C), 129.05 (2C), 123.99, 123.94 (2C), 120.80 (2C), 118.83 (2C), 115.21, 45.80, 21.59; MS (*m/z*): 537 (M^+^ + 1, 40%), 354 (40%); Anal. Calcd. for C_27_H_20_N_8_O_3_S (536.57): C, 60.44; H, 3.76; N, 20.88. Found: C, 60.10; H, 3.66; N, 20.59%.

##### 4.1.3.8. 4-(2-(Bis([1, 2, 4]triazolo)[4,3-*a*:3',4'-*c*]quinoxalin-3-ylthio)acetamido)-*N*-(3-chlorophenyl)benzamide (23h)

Yellowish white crystal (yield, 63%); m.p. = 280–282 °C. FT-IR (*ν*_max_, cm^−1^): 3267, 3109 (NH), 1701, 1647 (C=O), 1593 (C=N); ^1^H NMR (700 MHz, DMSO-d_6_) *δ* 10.79 (s, 1H), 10.30 (s, 1H), 10.02 (s, 1H), 8.61 (dd, *J* = 8.1, 1.5 Hz, 1H), 8.47 (dd, *J* = 7.9, 1.7 Hz, 1H), 7.98 − 7.95 (m, 3H), 7.78 − 7.73 (m, 4H), 7.70 (ddd, *J* = 8.3, 2.1, 1.0 Hz, 1H), 7.38 (t, *J* = 8.1 Hz, 1H), 7.15 (ddd, *J* = 8.0, 2.1, 0.9 Hz, 1H), 4.59 (s, 2H); ^13^C NMR (176 MHz, DMSO-d_6_) *δ* 166.08, 165.52, 147.71, 142.38, 142.12, 141.23, 139.34, 138.78, 133.38, 130.76, 129.63, 129.30 (2C), 128.37, 124.08, 123.65, 123.20, 120.10 (2C), 119.03, 118.86 (2C), 118.59, 118.06, 38.89; Anal. Calcd. for C_25_H_17_ClN_8_O_2_S (528.98): C, 56.77; H, 3.24; N, 21.18. Found: C, 56.37; H, 3.11; N, 20.99%.

##### 4.1.3.9. 4-(2-(Bis([1, 2, 4]triazolo)[4,3-*a*:3',4'-*c*]quinoxalin-3-ylthio)acetamido)-*N*-(4-chlorophenyl)benzamide (23i)

Yellow crystal (yield, 65%); m.p. = 277–279 °C. FT-IR (*ν*_max_, cm^−1^): 3382, 3111 (NH), 1674 (C=O), 1595 (C=N); ^1^H NMR (700 MHz, DMSO-d_6_) *δ* 10.78 (s, 1H), 10.28 (s, 1H), 10.03 (s, 1H), 8.62 (d, *J* = 8.0 Hz, 1H), 8.53 − 8.44 (m, 1H), 7.96 (d, *J* = 8.5 Hz, 3H), 7.82 (d, *J* = 8.5 Hz, 2H), 7.78 (d, *J* = 9.1 Hz, 1H), 7.74 (d, *J* = 8.4 Hz, 2H), 7.41 (d, *J* = 8.6 Hz, 2H), 4.59 (s, 2H); ^13^C NMR (176 MHz, DMSO-d_6_) *δ* 166.07, 165.39, 147.71, 142.29, 142.15, 139.37, 138.79, 138.72, 129.80, 129.26 (2C), 128.98 (2C), 128.38 (2C), 127.57, 124.12, 123.24, 122.27 (2C), 118.86 (2C), 118.61, 118.08, 38.88; Anal. Calcd. for C_25_H_17_ClN_8_O_2_S (528.98): C, 56.77; H, 3.24; N, 21.18. Found: C, 56.44; H, 3.19; N, 20.85%.

##### 4.1.3.10. 4-(2-(Bis([1, 2, 4]triazolo)[4,3-*a*:3′,4′-*c*]uinoxaline-3-ylthio)acetamido)-*N*-(2,5-dichlorophenyl)benzamide (23j)

White crystal (yield, 78%); m.p. = 260–262 °C. FT-IR (*ν*_max_, cm^−1^): 3429 (NH), 1672 (C=O), 1511 (C=N); ^1^H NMR (700 MHz, DMSO-d_6_) *δ* 10.80 (s, 1H), 10.03 (s, 2H), 8.62 (dd, *J* = 8.0, 1.5 Hz, 1H), 8.48 (dd, *J* = 7.9, 1.7 Hz, 1H), 7.99 − 7.97 (m, 2H), 7.77 (dt, *J* = 5.0, 2.2 Hz, 2H), 7.76 (m, 2H), 7.75 (s, 1H), 7.61 (d, *J* = 8.6 Hz, 1H), 7.38 (dd, *J* = 8.6, 2.6 Hz, 1H), 4.60 (s, 2H); ^13^C NMR (176 MHz, DMSO-d_6_) *δ* 166.11, 165.23, 147.70, 142.59, 142.15, 139.37, 138.79, 136.95, 131.89, 131.35, 129.39 (2C), 128.76, 128.39, 128.37, 128.20, 127.90, 127.39, 124.11, 123.23, 118.94 (2C), 118.60, 118.08, 38.89; MS (*m/z*): 563 (M^+^, 30%), 420 (65%); Anal. Calcd. For C_25_H_16_Cl_2_N_8_O_2_S (563.42): C, 53.30; H, 2.86; N, 19.89. Found: C, 53.01; H, 2.77; N, 19.66%.

##### 4.1.3.11. 4-(2-(Bis([1, 2, 4]triazolo)[4,3-*a*:3′,4′-*c*]uinoxaline-3-ylthio)acetamido)-*N*-(4-fluorophenyl)benzamide (23k)

White powder (yield, 80%); m.p. = 250–252 °C. FT-IR (*ν*_max_, cm^−1^): 3289, 3108 (NH), 1645 (C=O), 1604 (C=N); ^1^H NMR (700 MHz, DMSO-d_6_) *δ* 10.77 (s, 1H), 10.21 (s, 1H), 10.02 (s, 1H), 8.79 − 8.57 (m, 1H), 8.55 − 8.41 (m, 1H), 7.96 (t, *J* = 9.5 Hz, 2H), 7.78 (dt, *J* = 35.0, 12.1 Hz, 5H), 7.26 − 7.14 (m, 3H), 4.59 (s, 2H); ^13^C NMR (176 MHz, DMSO-d_6_) *δ* 166.04, 165.20, 147.72, 142.11, 139.34, 138.78, 129.17 (2C), 128.36 (2C), 124.08, 123.19, 122.61 (2C), 122.57 (2C), 118.84 (2C), 118.58, 118.06, 115.68 (2C), 115.55 (2C), 38.90; MS (*m/z*): 513 (M^+^ + 1, 25%), 372 (100%); Anal. Calcd. For C_25_H_17_FN_8_O_2_S (512.52): C, 58.59; H, 3.34; N, 21.86. Found: C, 58.34; H, 3.22; N, 21.69%.

##### 4.1.3.12. 4-(2-(Bis([1, 2, 4]triazolo)[4,3-*a*:3′,4′-*c*]uinoxaline-3-ylthio)acetamido)-*N*-(2-hydroxyphenyl)benzamide (23l)

Off white crystal (yield, 71%); m.p. = 282–284 °C. FT-IR (*ν*_max_, cm^−1^): 3263 (NH), 1646 (C=O), 1600 (C=N); ^1^H NMR (700 MHz, DMSO-d_6_) *δ* 10.78 (s, 1H), 10.02 (s, 1H), 9.76 (s, 1H), 9.46 (s, 1H), 8.60 (dd, *J* = 8.1, 1.4 Hz, 1H), 8.46 (dd, *J* = 8.0, 1.7 Hz, 1H), 7.97 − 7.96 (m, 2H), 7.75 − 7.73 (m, 3H), 7.69 (dt, *J* = 8.1, 2.1 Hz, 2H), 7.04 − 7.03 (m, 1H), 6.93 − 6.92 (m, 1H), 6.84 − 6.83 (m, 1H), 4.60 (s, 2H); ^13^C NMR (176 MHz, DMSO-d_6_) *δ* 166.05, 165.10, 149.68, 147.72, 142.24, 142.10, 139.33, 138.77, 129.49, 129.08 (2C), 128.36, 128.34, 126.46, 126.03, 124.43, 124.06, 123.17, 119.53 (2C), 118.95, 118.57, 118.06, 116.50, 38.93; MS (*m/z*): 511 (M^+^ + 1, 70%), 293 (100%); Anal. Calcd. For C_25_H_18_N_8_O_3_S (510.53): C, 58.82; H, 3.55; N, 21.95. Found: C, 58.78; H, 3.50; N, 21.88%.

##### 4.1.3.13. 4-(2-(Bis([1, 2, 4]triazolo)[4,3-*a*:3′,4′-*c*]uinoxaline-3-ylthio)acetamido)-*N*-(4-hydroxyphenyl)benzamide (23m)

Pale yellow crystal (yield, 75%); m.p. = 285–287 °C. FT-IR (*ν*_max_, cm^−1^): 3414 (NH), 1601 (C=N); ^1^H NMR (700 MHz, DMSO-d_6_) *δ* 10.75 (s, 1H), 10.01 (s, 1H), 9.93 (s, 1H), 9.26 (s, 1H), 8.63 − 8.54 (m, 1H), 8.51 − 8.39 (m, 1H), 7.93 (d, *J* = 8.7 Hz, 2H), 7.72 (dq, *J* = 14.7, 7.1, 6.6 Hz, 4H), 7.55 − 7.49 (m, 2H), 6.79 − 6.70 (m, 2H), 4.59 (s, 2H); ^13^C NMR (176 MHz, DMSO-d_6_) *δ* 165.99, 164.72, 154.09 (2C), 147.74, 142.05, 139.30, 138.77, 131.21, 130.33, 128.98 (2C), 128.33 (2C), 124.01, 123.12, 122.74 (2C), 118.81(2C), 118.55, 118.02, 115.42 (2C), 38.92; MS (*m/z*): 511(M^+^ + 1, 30%); Anal. Calcd. For C_25_H_18_N_8_O_3_S (510.53): C, 58.82; H, 3.55; N, 21.95. Found: C, 58.63; H, 3.48; N, 21.73%.

##### 4.1.3.14. 4-(2-(Bis([1, 2, 4]triazolo)[4,3-*a*:3′,4′-*c*]uinoxaline-3-ylthio)acetamido)-*N*-(2-hydroxy-4-nitrophenyl)benzamide (23n)

Red crystal (yield, 68%); m.p. = 265–267 °C. FT-IR (*ν*_max_, cm^−1^): 3294, 3101 (NH), 2927 (CH aliphatic), 1653 (C=O), 1603 (C=N); ^1^H NMR (700 MHz, DMSO-d_6_) *δ* 11.14 (s, 1H), 10.82 (s, 1H), 10.02 (s, 1H), 9.49 (s, 1H), 8.61 (dd, *J* = 8.1, 1.5 Hz, 1H), 8.47 (dd, *J* = 7.8, 1.8 Hz, 1H), 8.25 (d, *J* = 8.9 Hz, 1H), 7.98 − 7.96 (m, 2H), 7.79 (dd, *J* = 8.9, 2.6 Hz, 1H), 7.78 − 7.75 (m, 3H), 7.74 (dd, *J* = 6.7, 2.1 Hz, 2H), 4.60 (s, 2H); ^13^C NMR (176 MHz, DMSO-d_6_) *δ* 166.14, 165.06, 148.55, 147.70, 143.66, 142.72, 142.13, 139.35, 138.78, 133.63, 129.27 (2C), 128.90, 128.38, 128.36, 124.08, 123.20, 121.83, 119.05 (2C), 118.59, 118.07, 115.57, 109.89, 38.91; MS (*m/z*): 554 (M^+^ − 1, 70%); Anal. Calcd. For C_25_H_17_N_9_O_5_S (555.53): C, 54.05; H, 3.08; N, 22.69. Found: C, 53.85; H, 2.97; N, 22.43%.

#### 4.1.4. General procedure for the synthesis of compounds 24a–c

A mixture of potassium salt of bis([1, 2, 4]triazolo)[4,3-*a*:3′,4′-*c*]quinoxaline-3-thiol **14** (0.5 g, 0.001 mol) and 2-chloro-*N*-(4–(2-(substituted)hydrazine-1-carbonyl)phenyl)acetamide **22a**–**c** (0.001 mol) in DMF (50 ml) was heated on a water bath for 6 h. After cooling to room temperature, the reaction mixture was poured on crushed ice. The obtained precipitates were collected by filtration, dried, and crystalised from ethanol to give the target compounds **24a**–**c.**

##### 4.1.4.1. 2-(Bis([1, 2, 4]triazolo)[4,3-*a*:3',4'-*c*]quinoxalin-3-ylthio)-*N*-(4–(2-(2-chlorobenzoyl)hydrazine-1-carbonyl)phenyl)acetamide (24a)

White crystal (yield, 65%); m.p. = 220–222 °C. FT-IR (*ν*_max_, cm^−1^): 3185 (NH), 1698 (C=O), 1603 (C=N); ^1^H NMR (700 MHz, DMSO-d_6_) *δ* 10.77 (s, 1H), 10.54 (s, 1H), 10.37 (s, 1H), 10.03 (s, 1H), 8.63 (dd, *J* = 8.1, 1.5 Hz, 1H), 8.48 (dd, *J* = 7.9, 1.7 Hz, 1H), 7.93 (d, *J* = 8.7 Hz, 2H), 7.77 (dtd, *J* = 17.1, 7.5, 1.5 Hz, 2H), 7.72 (d, *J* = 8.7 Hz, 2H), 7.57 (ddd, *J* = 8.0, 4.0, 1.5 Hz, 2H), 7.53 (td, *J* = 7.6, 1.7 Hz, 1H), 7.48 (td, *J* = 7.4, 1.3 Hz, 1H), 4.60 (s, 2H); ^13^C NMR (176 MHz, DMSO-d_6_) *δ* 166.25, 166.08, 165.40, 147.70, 142.36, 142.15, 139.37, 138.79, 135.18, 131.98, 130.93, 130.38, 129.90, 129.06 (2C), 128.37 (2C), 127.64 (2C), 124.12, 123.23, 118.91(2C), 118.60, 118.10, 38.94. MS (*m/z*): 572 (M^+^, 100%); Anal. Calcd. for C_26_H_18_ClN_9_O_3_S (572): C, 54.60; H, 3.17; N, 22.04. Found: C, 54.39; H, 3.09; N, 21.88%.

##### 4.1.4.2. 2-(Bis([1, 2, 4]triazolo)[4,3-*a*:3',4'-*c*]quinoxalin-3-ylthio)-*N*-(4–(2-(3-chlorobenzoyl)hydrazine-1-carbonyl)phenyl)acetamide (24b)

White crystal (yield, 63%); m.p. = 232–234 °C. FT-IR (*ν*_max_, cm^−1^): 3260, 3108 (NH), 1639 (C=O), 1603 (C=N); ^1^H NMR (700 MHz, DMSO-d_6_) *δ* 10.78 (s, 1H), 10.62 (s, 1H), 10.49 (s, 1H), 10.02 (s, 1H), 8.62 (dd, *J* = 8.0, 1.5 Hz, 1H), 8.47 (dd, *J* = 7.8, 1.7 Hz, 1H), 7.96 (t, *J* = 1.9 Hz, 1H), 7.92 (d, *J* = 8.5 Hz, 2H), 7.91 − 7.88 (m, 1H), 7.78 − 7.76 (m, 1H), 7.75 − 7.71 (m, 3H), 7.70 − 7.69 (m, 1H), 7.59 (t, *J* = 7.9 Hz, 1H), 4.60 (s, 2H); ^13^C NMR (176 MHz, DMSO-d_6_) *δ* 166.09, 165.67, 164.99, 147.71, 142.40, 142.13, 139.35, 138.78, 135.00, 133.84, 132.23, 131.11, 129.03 (2C), 128.38, 128.36, 127.72 (2C), 127.66, 126.66, 124.09, 123.21, 118.96, 118.59, 118.07, 38.92; MS (*m/z*): 572 (M^+^, 100%); Anal. Calcd. for C_26_H_18_ClN_9_O_3_S (572): C, 54.60; H, 3.17; N, 22.04. Found: C, 54.45; H, 3.11; N, 21.95%.

##### 4.1.4.3. 2-(Bis([1, 2, 4]triazolo)[4,3-*a*:3',4'-*c*]quinoxalin-3-ylthio)-*N*-(4–(2-(2-hydroxybenzoyl)hydrazine-1-carbonyl)phenyl)acetamide (24c)

Yellow crystal (yield, 60%); m.p. = 270–272 °C. FT-IR (*ν*_max_, cm^−1^): 3282 (NH), 1648, 1619 (C=O), 1599 (C=N); ^1^H NMR (700 MHz, DMSO-d_6_) *δ* 11.96 (s, 1H), 10.78 (s, 1H), 10.67 (s, 1H), 10.59 (s, 1H), 10.02 (s, 1H), 8.62 (dd, *J* = 8.0, 1.5 Hz, 1H), 8.47 (dd, *J* = 7.8, 1.7 Hz, 1H), 7.93 (dd, *J* = 10.4, 7.6 Hz, 3H), 7.77 (ddt, *J* = 10.8, 8.3, 3.9 Hz, 2H), 7.74 (dd, *J* = 7.3, 4.8 Hz, 2H), 7.47 (ddd, *J* = 8.7, 7.2, 1.7 Hz, 1H), 7.00 − 6.95 (m, 2H), 4.59 (s, 2H); ^13^C NMR (176 MHz, DMSO-d_6_) *δ* 168.26, 166.09, 165.44, 159.79, 147.71, 142.45, 142.13, 139.35, 138.78, 134.65, 129.07 (2 C), 128.73, 128.38, 128.36, 127.52, 124.09, 123.21, 119.52, 118.96 (2C), 118.59, 118.07, 117.88, 115.01, 38.91; MS (*m/z*): 554 (M^+^ + 1, 100%); Anal. Calcd. for C_26_H_19_N_9_O_4_S (553.56): C, 56.41; H, 3.46; N, 22.77. Found: C, 56.20; H, 3.39; N, 22.58%.

### 4.2. Biological testing

#### 4.2.1. *In vitro* anti-proliferative activity

MTT assay protocol^55–57^^,^^74^ was applied as described in Supplementary data.

#### 4.2.2. *In vitro* VEGFR-2 kinase assay

All the synthesised compounds were tested for their inhibitory activity against VEGFR-2 as described in Supplementary data^75^.

#### 4.2.3. Flow cytometry analysis for cell cycle

Cell cycle analysis was performed using propidium iodide (PI) staining and flow cytometry analysis for compound **23j** as described in Supplementary data^76^^,^^77^.

#### 4.2.4. Flow cytometry analysis for apoptosis

Apoptotic effect was assessed for compound **23j** as described in Supplementary data^78^^,^^79^.

#### 4.2.5. Western blot analysis

Western blot technique was performed for compound **23j** to determine its effect against caspase3, caspase9, BAX, and Bcl-2 as described in Supplementary data^80–82^.

### 4.3. *In silico* studies

#### 4.3.1. Docking studies

Docking studies were carried out against VEEGFR-2 (PDB ID: 2OH4) using Discovery Studio 4.0 as described in Supplementary data^83–85^^,^^86^^,^^87^.

#### 4.3.2. Admet studies

ADMET studies were carried out as described in Supplementary data^85^^,^^87^.

#### 4.3.3. Toxicity studies

Toxicity studies were performed as described in Supplementary data.

## Supplementary Material

Supplemental Material
