# Supplementary material for: Discovery of new VEGFR-2 inhibitors based on bis([1, 2, 4]triazolo)[4,3-a:3',4'-c]quinoxaline derivatives as anticancer agents and apoptosis inducers
Source: J Enzyme Inhib Med Chem. 2021 May 31;36(1):1093–114. doi: 10.1080/14756366.2021.1915303 (PMC8168755; doi:10.1080/14756366.2021.1915303)
Supplement: Supplemental Material [file IENZ_A_1915303_SM9751.pdf]

Discovery of new VEGFR-2 inhibitors based on bis([1,2,4]triazolo)[4,3-*a*:3',4'-*c*]quinoxaline derivatives as anticancer agents and apoptosis inducers

Nawaf A. Alsaif[a*], Mohammed S. Taghour[b], Mohammed M. Alanazi[a], Ahmad J. Obaidullah[a], Abdulrahman A. Al-Mehizia[a], Manal M. Alanazi[a], Saleh Aldawas[a], Alaa Elwan[b*], Hazem Elkady[b*].

[a] Department of Pharmaceutical Chemistry, College of Pharmacy, King Saud University, Riyadh, Saudi Arabia

[b] Pharmaceutical Medicinal Chemistry & Drug Design Department, Faculty of Pharmacy (Boys), Al-Azhar University, Cairo 11884, Egypt.

*** Corresponding authors:**

Hazem Elkady:

Pharmaceutical Medicinal Chemistry & Drug Design Department, Faculty of Pharmacy (Boys), Al-Azhar University, Cairo 11884, Egypt.

Email: Hazemelkady@azhar.edu.eg

Nawaf A. Alsaif

Department of Pharmaceutical Chemistry, College of Pharmacy, King Saud University, Riyadh, Saudi Arabia

Email: nalsaif@KSU.EDU.SA

Alaa Elwan

Pharmaceutical Medicinal Chemistry & Drug Design Department, Faculty of Pharmacy (Boys), Al-Azhar University, Cairo 11884, Egypt.

Email: alaaelwan34@azhar.edu.eg

Content

**1**	Highlights
**2**	Experimental chemistry
**3**	Experimental of biological testing
**4**	*In silico* studies procedures
**5**	Spectral data of the intermediate compounds **18a-n** & **22a-c** and final targe compounds **23a-n** & **24a-c**
**6**	*In silico* toxicity data

Highlights

- Seventeen compounds of novel quinoxaline derivatives were designed and synthesized.
- The synthesized compounds were assessed for their *in vitro* antiproliferative and VEGFR-2 inhibitory activities.
- Apoptotic activities were evaluated against BAX, Bcl-2, caspase3, and caspase9.
- *In silico* studies were carried.

Experimental chemistry

All solvents and reagents were commercially available and used without further purification. Progress of reactions were monitored by TLC using TLC sheets coated with UV fluorescent silica gel (Kieselgel 0.25mm, 60 F254, Merck Germany) with a developing solvent system of DCM/methanol (95:5) and were visualized using UV lamp. The melting points were determined using a Gallen lamp melting point apparatus. Elemental analyses were accomplished using a CHN analyzer. The infrared spectra were recorded on FT/IR-6600typeA spectrophotometer. ^{1}H NMR spectra were recorded at 400 and 700 MHz, while ^{13}C NMR spectra were run at 100 and 176 MHz, on a Bruker Avance NEO-600 equipped with a 1.7 mm TCI CryoProbe. Chemical shifts were expressed in δ (ppm) with reference to TMS and coupling constant (J) in Hertz using DMSO-d_6 and $CDCl_3$-d_6 as solvents. The mass spectra were recorded on an Agilent 6410 triple-quadrupole mass spectrometer equipped with an ESI source.

Experimental of biological testing

1. *In vitro* anti-proliferative activity

MTT assay protocol was applied as described previously to assess the anti-proliferative activity of the synthesized compounds. Two human cancer cell lines (MCF-7 and HepG-2) were used in this test. At first, the cell lines were cultured in RPMI-1640 medium with 10% fetal bovine serum. Antibiotics (100 units/ml penicillin and100 µg/ml streptomycin) were added at 37°C in a 5% CO_2 incubator. The cell lines were seeded in a 96-well plate at a density of 1.0 x 104 cells / well at 37 ^{o}C for 48 h under 5% CO_2. After incubation, the cells were treated with different concentration of the synthesized compounds and incubated for 24 h. After 24 h of drug treatment, 20 µl of MTT solution at 5mg/ml was added and incubated for 4 h. Dimethyl sulfoxide (DMSO) in volume of 100 µl was added into each well to dissolve the purple formazan formed. The colorimetric assay was measured and recorded at absorbance of 570 nm using a plate reader (EXL 800, USA). The relative cell viability in percentage was calculated as (A570 of treated samples/A570 of untreated sample) X 100.

2. *In vitro* VEGFR-2 kinase assay

All the synthesized compounds were tested for its inhibitory activity against VEGFR-2. Human VEGFR-2 ELISA kit (Enzyme-Linked Immunosorbent Assay) was utilized in this test. At first, specific antibody for VEGFR-2 was seeded on a 96-well plate and 100 µL of the standard solution or the tested compound was added, all were incubated at room temperature for 2.5 h. Then washed, 100 µL of the prepared biotin antibody was added, then incubated at room temperature for additional 1 h. and washed. Then, 100 µL of streptavidin solution was added and incubated for 45 min. at room temperature. Washed again, 100 µL of TMB Substrate reagent was added and incubated for 30 min. at room temperature. 50 µL of the stop solution was added, then read at 450 nm immediately. The standard curve was drawn, concentrations on the X-axis and the absorbance on the Y-axis.

3. Flow cytometry analysis for cell cycle

To determine the role of the synthesized compounds in cell cycle distribution, cell cycle analysis was performed using propidium iodide (PI) staining and flow cytometry analysis for compound **11e**. Flow Cytometry Kit for Cell Cycle Analysis (ab139418_Propidium Iodide Flow Cytometry Kit/BD) was used in this test. HepG2 cells were treated with compound **23j** (6.4 µM) for 24 h.

Then, the cells were fixed in 70% ethanol at 4 °C for 12 h. After that, the cells were washed with cold PBS, incubated with 100 µl RNase A at 37 °C for 30 min, and stained with 400 µl PI in the dark at room temperature for further 30 min. The stained cells were measured using Epics XL-MCL™ Flow Cytometer (Beckman Coulter), and the data were analyzed using Flowing software (version 2.5.1, Turku Centre for Biotechnology, Turku, Finland).

4. Flow cytometry analysis for apoptosis

Flow cytometry cell apoptosis analysis was used to investigate the apoptotic effect of the synthesized compounds. HepG2 cells were treated with compound **23j** (6.4 µM) for 24 h, collected by trypsin, centrifuged, washed two successive times with PBS, suspended in 500 µl binding buffer, and double stained with 5 µl Annexin V-FITC and 5 µl PI in the dark at room temperature for 15 min. The stained cells were measured using Epics XL-MCL™ Flow Cytometer and analyzed using Flowing software.

5. Western blot analysis

The proteins expression of cleaved caspase-3, caspase-9, BAX, and Bcl-2 were determined using Western blot analysis. In brief, HepG2 cells were treated with control, or with the synthesized compounds. Then cells were lysed in 250 µL precold lysis buffer (pH 7.4: Tris-Base [10 mM], NaCl [100 mM], ethylenediaminetetraacetic acid [EDTA, 25 mM], ethylene glycol bis (2-aminoethyl) tetraacetic acid [EGTA, 25 mM], 1% [v/v] NP-40, and 1% [v/v] Triton X-100) supplemented with 1:350 protease:phosphatase inhibitors cocktail (Sigma). The cells were immediately frozen at −20°C for 1.5 hours for further lysis, collected by cell scraper, sonicated 3 × 10 seconds, and centrifuged (13000 rpm, 15 minutes). Total protein concentrations in the supernatant were determined colorimetrically using the Pierce 660 nm Protein Assay method (Thermo Fisher Scientific, Rockford, IL), with BSA as the standard. Equal amounts of protein (25 µg) samples were mixed with SDS-loading buffer (pH 6.8: Tris-HCl [700 mM], dithiothreitol [DTT, 600 mM], sodium dodecyl sulfate [SDS, 12%], glycerol [60%], and bromophenol blue [0.012%]), denatured by boiling at 95°C for 10 minutes, allowed to cool on ice for 15 minutes, vortexed vigorously for 30 seconds and loaded into SDS-polyacrylamide gel and separated by an electrophoresis unit (Cleaver Scientific Ltd, UK), transferred onto polyvinylidene fluoride membranes (Bio-Rad) for 35 minutes using a Trans-Blot SD semi-dry transfer cell (Bio-Rad) at

250 mA and 22 V. Membranes were blocked with 5% (w/v) blotting grade dry milk (Bio-Rad) in Tris-buffered saline/Tween-20 (TBS-T) (pH 7.5: Tris-base [20 mM], NaCl [150 mM], and 0.05% [v/v] Tween- 20) while shaking for 1.5 hour at RT, and then incubated with the corresponding primary antibody against cleaved caspase-3 antibody (1:000, #9661; Cell Signaling Technology), caspase-9 antibody (1:1000, #9662; Cell Signaling Technology), β-actin (1:2000, #A5060; Sigma), BAX (Biovision, USA) and Bcl-2(Bioimaging, system, syngene, UK) for 9-10 hours at 4°C in a humidified chamber. The blots were washed with TBS-T three times for 15 minutes and incubated with matched horseradish peroxidase (HRP)-linked secondary antibodies (Dako, Denmark) for another 1 hour at RT, followed by washing 3 × 15 min with TBS-T. After membranes were incubated at RT with 1:1 reagent mixture of chemiluminescence Western Lightning ECL (Perkin Elmer, Waltham, MA) for 1 minute, the bands were visualized in Chemi-Doc imager (Bio-Rad). Means of the detected blot intensities were then quantified, analyzed by the combined Bio-Rad Image Lab software and their corresponding background subtracted, with normalization to the corresponding bands density of β-actin as the sampling loading control. Data were collected from three separate experiments.

6. Statistical analysis

Graph Pad Prism 6 software was used for statistical evaluation of the grouped data. Values are expressed as the mean ± SD of the triplicates of each experiment. Two - way analysis of variance with multiple comparisons post hoc test were used for normally distributed quantitative variables. P value of less than 0.05 was accepted as statistically significant.

***In silico* studies procedures**

1. Docking studies

The crystal structure of VEGFR-2 was downloaded from the Protein Data Bank, http://www.rcsb.org/pdb (PDB ID: 2OH4, resolution: 2.05 Å). Discovery Studio 4.0 software was used in the docking studies. At first, the target molecule was prepared by removal of the water molecules. Any crystallographic disorders and unfilled valence atoms were corrected using alternate conformations and valence monitor options. Then, the protein structure was subjected to energy minimization by applying CHARMm force fields for charge, and MMFF94 force field for partial charge. Inflexibility of structure was obtained by creating fixed atom

constraint. The binding site of the protein was defined and prepared for docking. Sorafenib and the designed compounds 2D structures were sketched using ChemBioDraw Ultra 14.0 and saved in MDL-SD file format. Next, the SD file was opened, 3D structures were protonated, and energy was minimized by applying CHARMm force fields for charge andMMFF94 force field for partial charge and then prepared for docking by optimization of the parameters. Docking process was accomplished using CDOCKER-CHARMm-based technique in the interface of Accelry's Discovery Studio 4.0. A maximum of 10 conformers was considered for each molecule in the docking analysis. After that the docking scores (CDOCKER interaction energy) of the best -fitted conformation of each of the docked molecules with the amino acids at the VEGFR-2 binding pocket were recorded

2. ADMET studies

ADMET descriptors (absorption, distribution, metabolism, excretion and toxicity) of the compounds were determined using Discovery studio 4.0. At first, the CHARMM force field was applied then the tested compounds were prepared and minimized according to the preparation of small molecule protocol. Then ADMET descriptors protocol was applied to carry out these studies.

3. Toxicity studies

The toxicity parameters of the synthesized compounds were calculated using Discovery studio 4.0. Sorafenib was used as a reference drug. At first, the CHARMM force field was applied then the compounds were prepared and minimized according to the preparation of small molecule protocol. Then different parameters were calculated from toxicity prediction (extensible) protocol.

IR of compound 18a

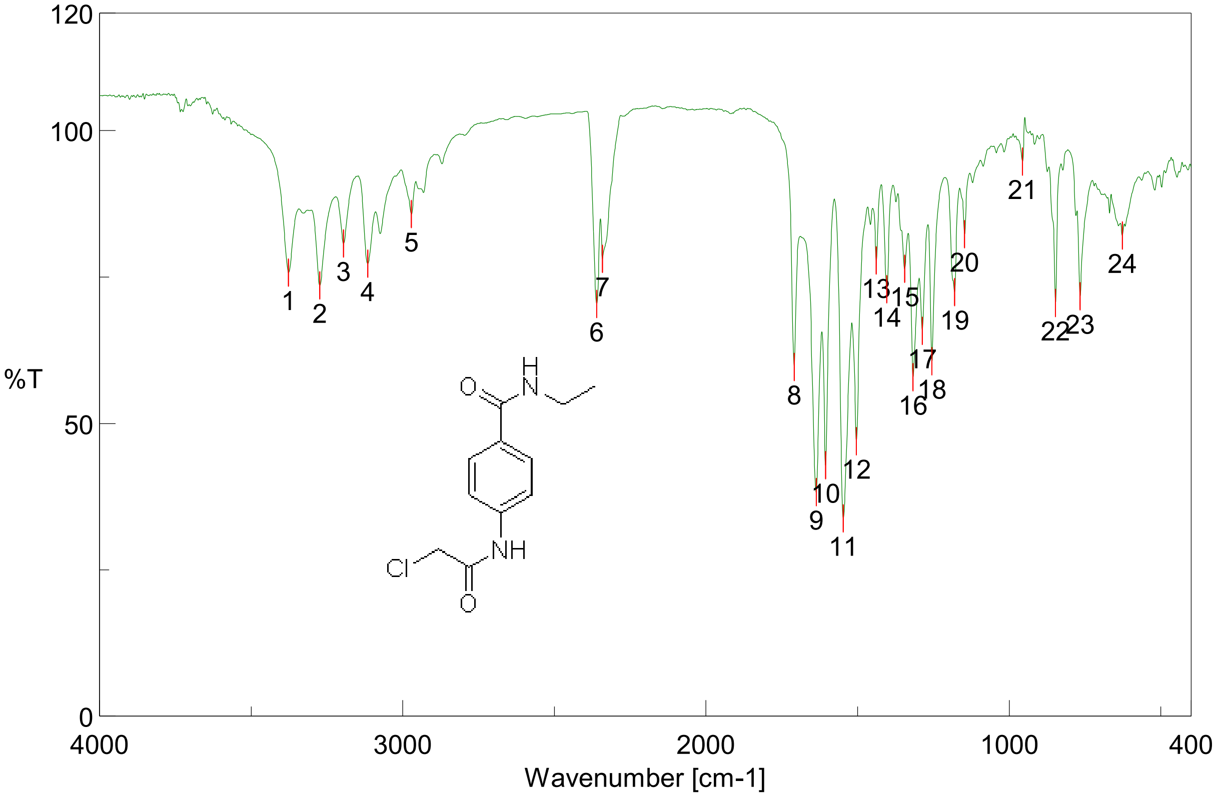

[Comments]

Sample name	A22
Comment	
User	
Division	
Company	KSU

[Detailed Information]

Creation date	9/15/2020 4:35 AM
Data array type	Linear data array
Horizontal axis	Wavenumber [cm-1]
Vertical axis	%T
Start	399.193 cm-1
End	4000.6 cm-1
Data interval	0.964233 cm-1
Data points	3736

[Measurement Information]

Model Name	FT/IR-6600typeA
Serial Number	A014661790
Measurement Date	9/15/2020 4:34 AM
Light Source	Standard
Detector	TGS
Accumulation	Auto (14)
Resolution	4 cm-1
Zero Filling	On
Apodization	Cosine
Gain	Auto (1)
Aperture	Auto (7.1 mm)
Scanning Speed	Auto (2 mm/sec)
Filter	Auto (10000 Hz)

[Result of Peak Picking]

No.	Position	Intensity	No.	Position	Intensity
1	3376.75	75.6799	2	3273.57	73.5203

[Result of Peak Picking]

No.	Position	Intensity	No.	Position	Intensity
3	3195.47	80.6549	4	3115.44	77.2436
5	2971.77	85.6717	6	2360.44	70.3551
7	2341.16	78.0707	8	1709.59	59.6132
9	1636.3	38.2102	10	1605.45	42.7991
11	1547.59	33.7802	12	1504.2	46.9011
13	1438.64	77.7571	14	1403.92	72.8882
15	1345.11	76.3499	16	1317.14	57.8195
17	1287.25	65.7832	18	1255.43	60.5531
19	1180.22	72.3574	20	1147.44	82.2692
21	956.52	94.671	22	847.561	70.4898
23	766.566	71.6772	24	626.752	82.0664

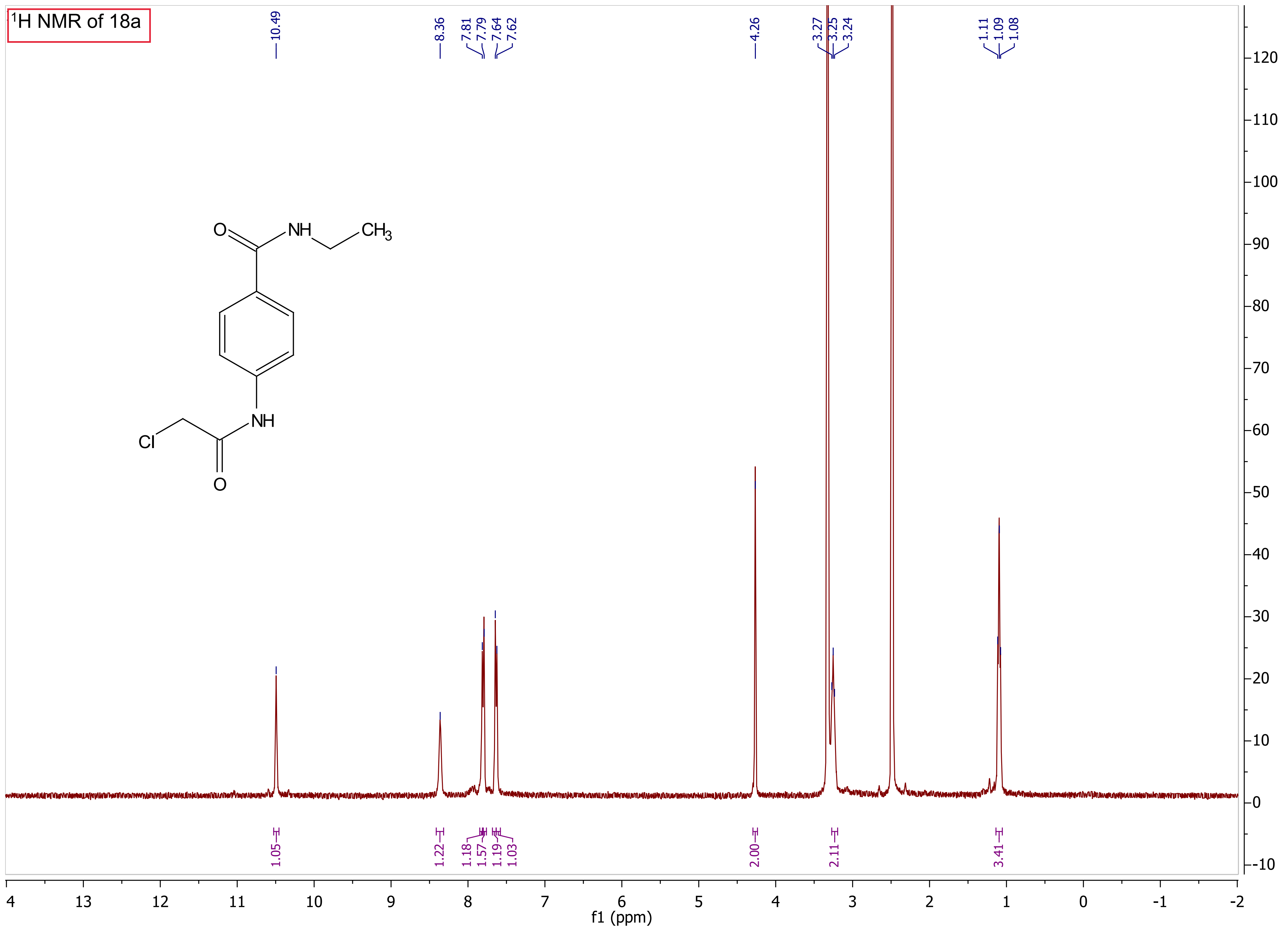

1H NMR of 18a
10.49
8.36
7.81
7.79
7.64
7.62
4.26
3.27
3.25
3.24
1.11
1.09
1.08
1.05
1.22
1.18
1.57
1.19
1.03
2.00
2.11
3.41
f1 (ppm)

^{1}H NMR of 18a

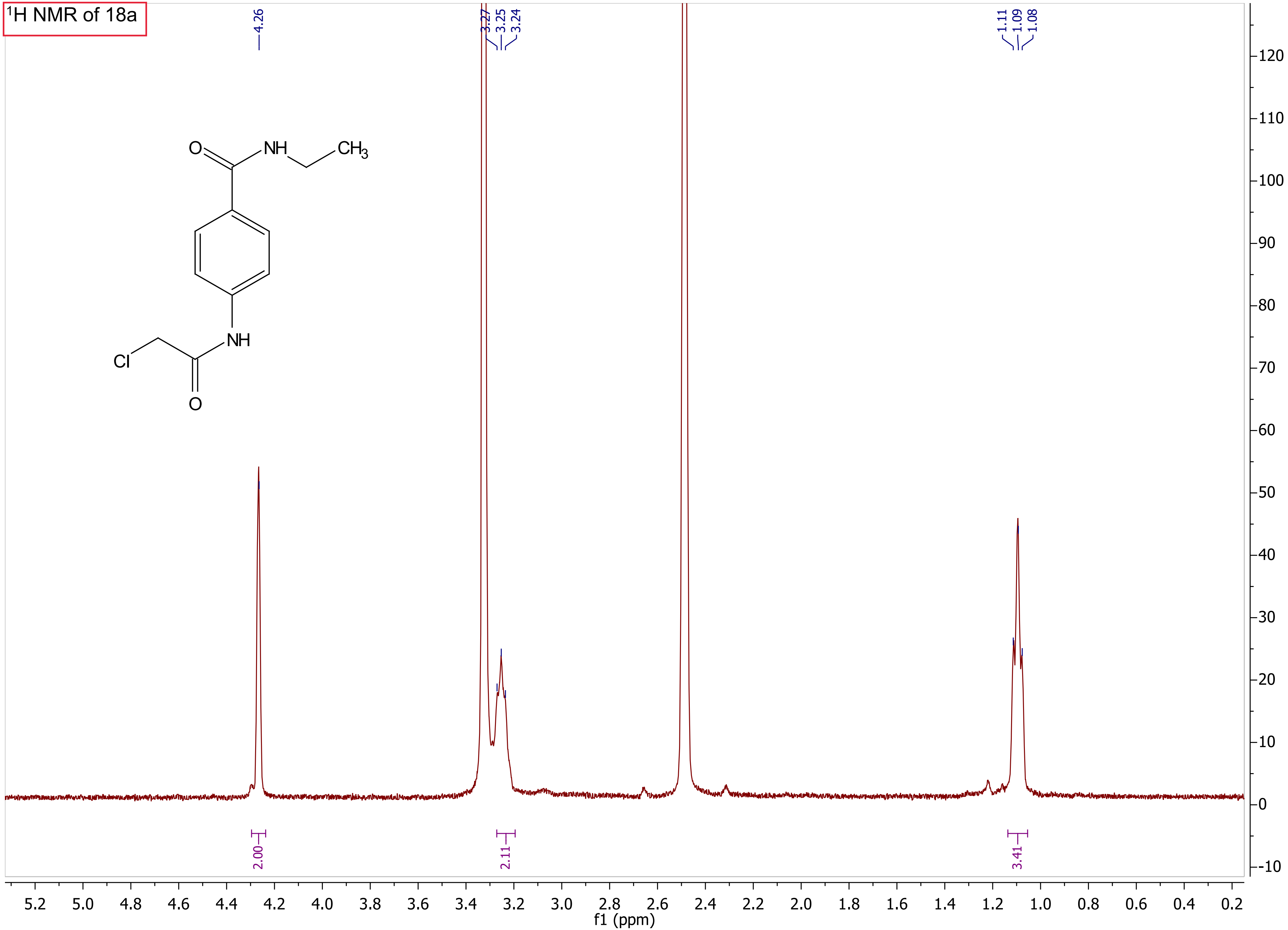

O
NH
CH3
NH
Cl
O
4.26
3.27
3.25
3.24
1.11
1.09
1.08
2.00
2.11
3.41
5.2 5.0 4.8 4.6 4.4 4.2 4.0 3.8 3.6 3.4 3.2 3.0 2.8 2.6 2.4 2.2 2.0 1.8 1.6 1.4 1.2 1.0 0.8 0.6 0.4 0.2
f1 (ppm)
120 110 100 90 80 70 60 50 40 30 20 10 0 -10

^{1}H NMR of 18a

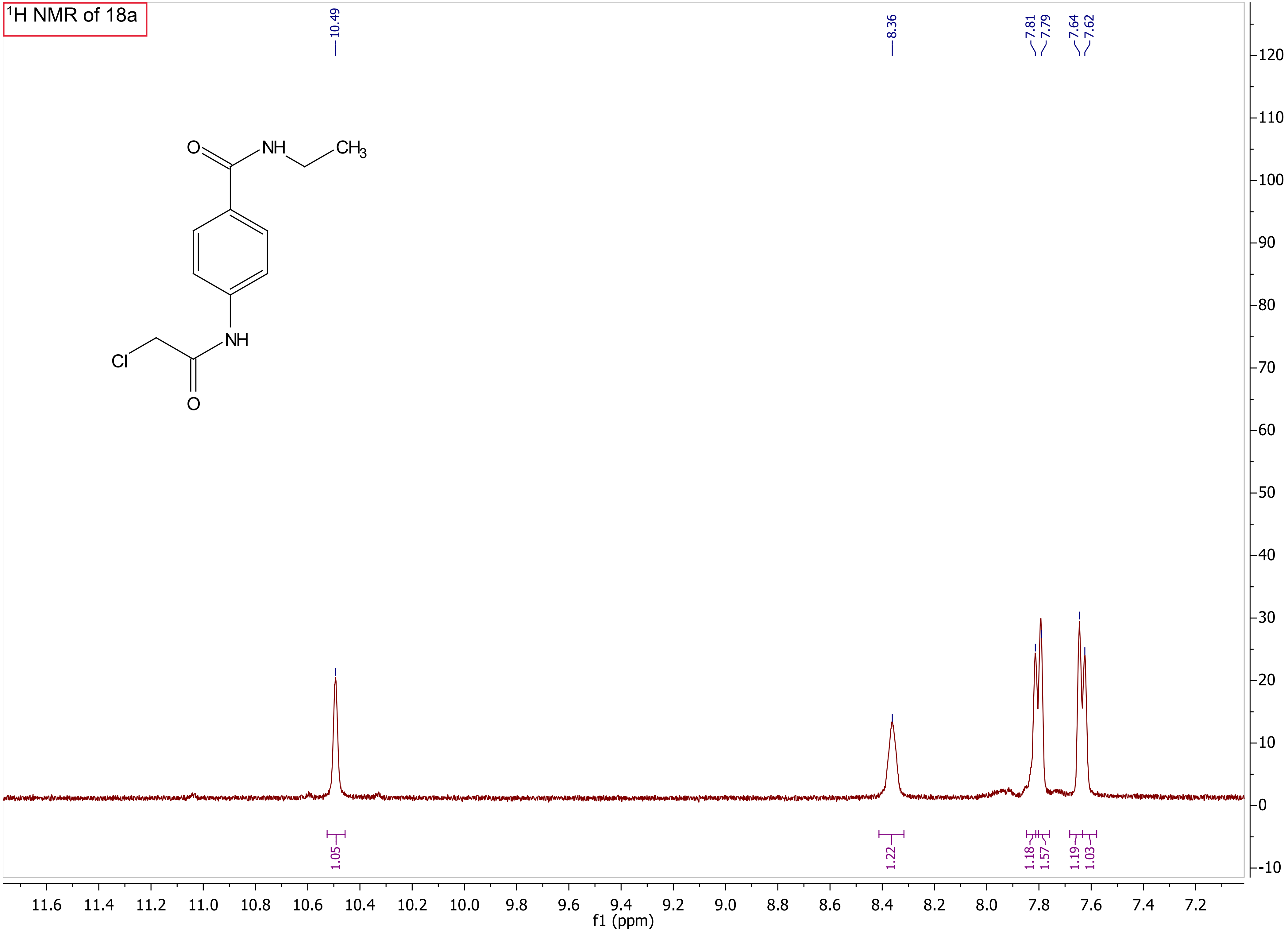

10.49
8.36
7.81
7.79
7.64
7.62
1.05
1.22
1.18
1.57
1.19
1.03
O
NH
CH3
NH
Cl
O
11.6 11.4 11.2 11.0 10.8 10.6 10.4 10.2 10.0 9.8 9.6 9.4 9.2 9.0 8.8 8.6 8.4 8.2 8.0 7.8 7.6 7.4 7.2
f1 (ppm)
120 110 100 90 80 70 60 50 40 30 20 10 0 -10

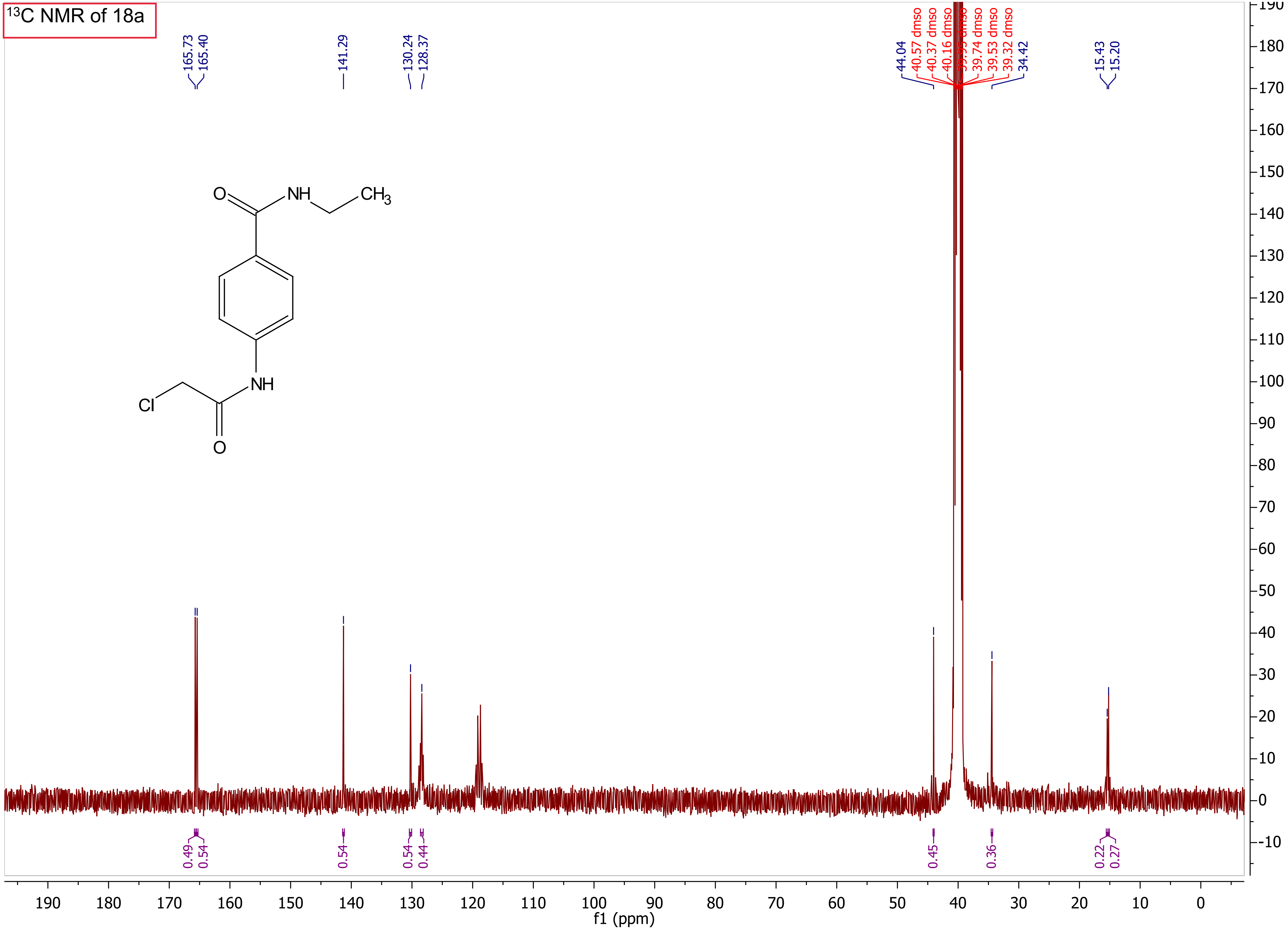

^{13}C NMR of 18a
165.73
165.40
141.29
130.24
128.37
44.04
40.57 dmso
40.37 dmso
40.16 dmso
39.74 dmso
39.53 dmso
39.32 dmso
34.42
15.43
15.20
O
NH
CH3
Cl
NH
O
0.49
0.54
0.54
0.54
0.44
0.45
0.36
0.22
0.27
f1 (ppm)

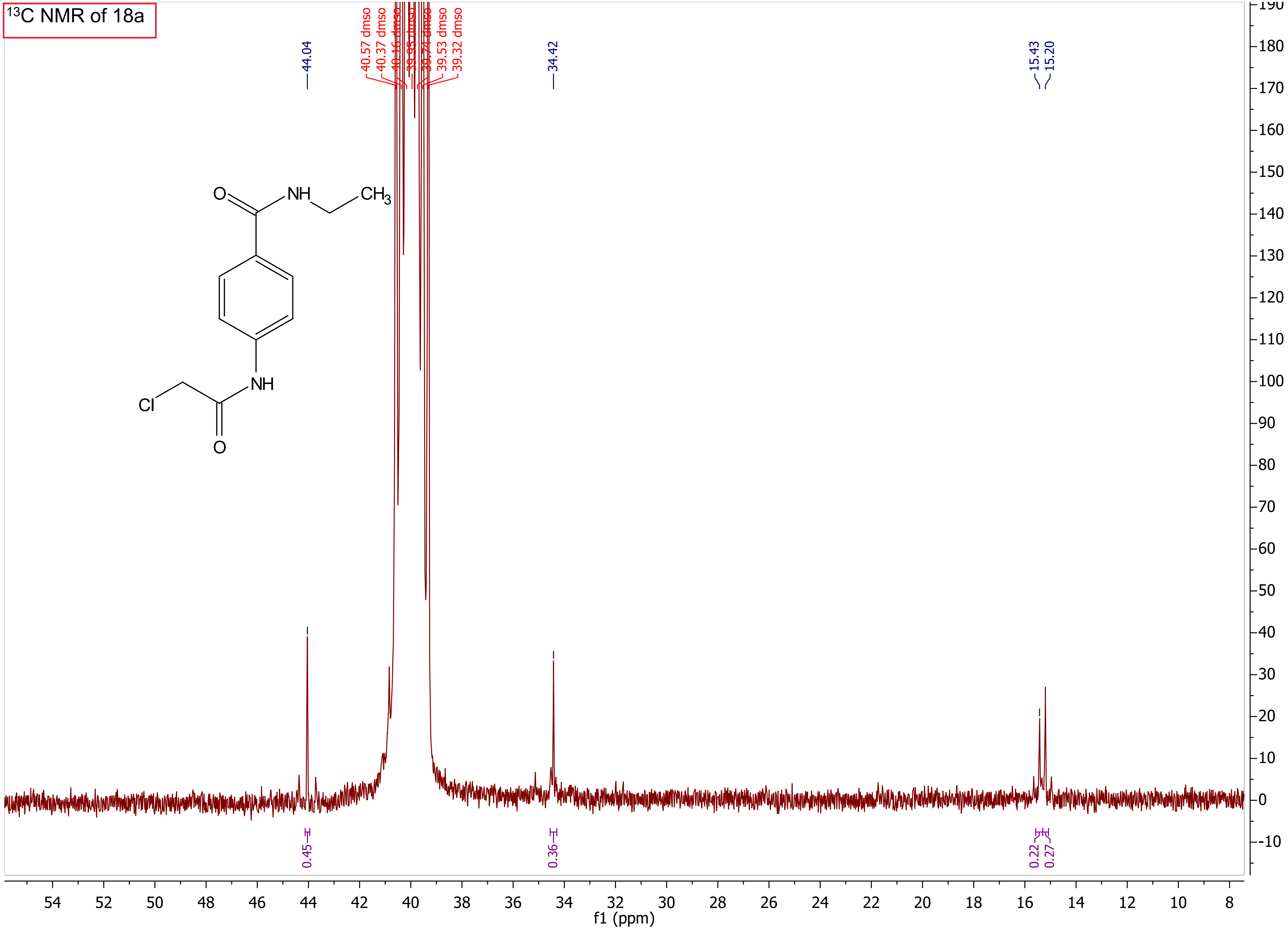

^{13}C NMR of 18a
44.04
40.57 dmso
40.37 dmso
40.16 dmso
39.95 dmso
39.74 dmso
39.53 dmso
39.32 dmso
34.42
15.43
15.20
0.45
0.36
0.22
0.27
f1 (ppm)

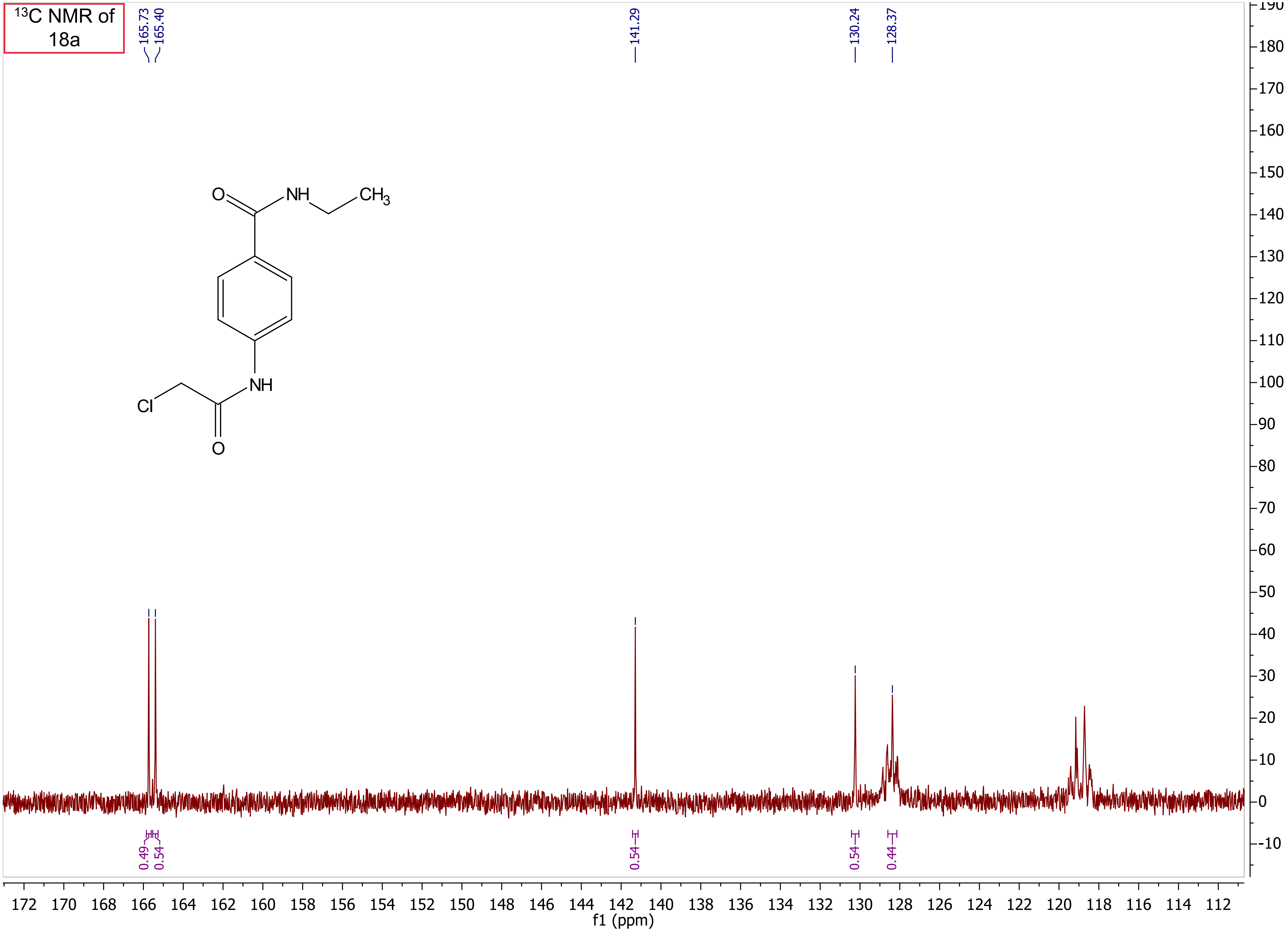

^{13}C NMR of 18a
165.73
165.40
141.29
130.24
128.37
O
NH
CH3
NH
Cl
O
0.49
0.54
0.54
0.54
0.44
f1 (ppm)

IR of compound 18b

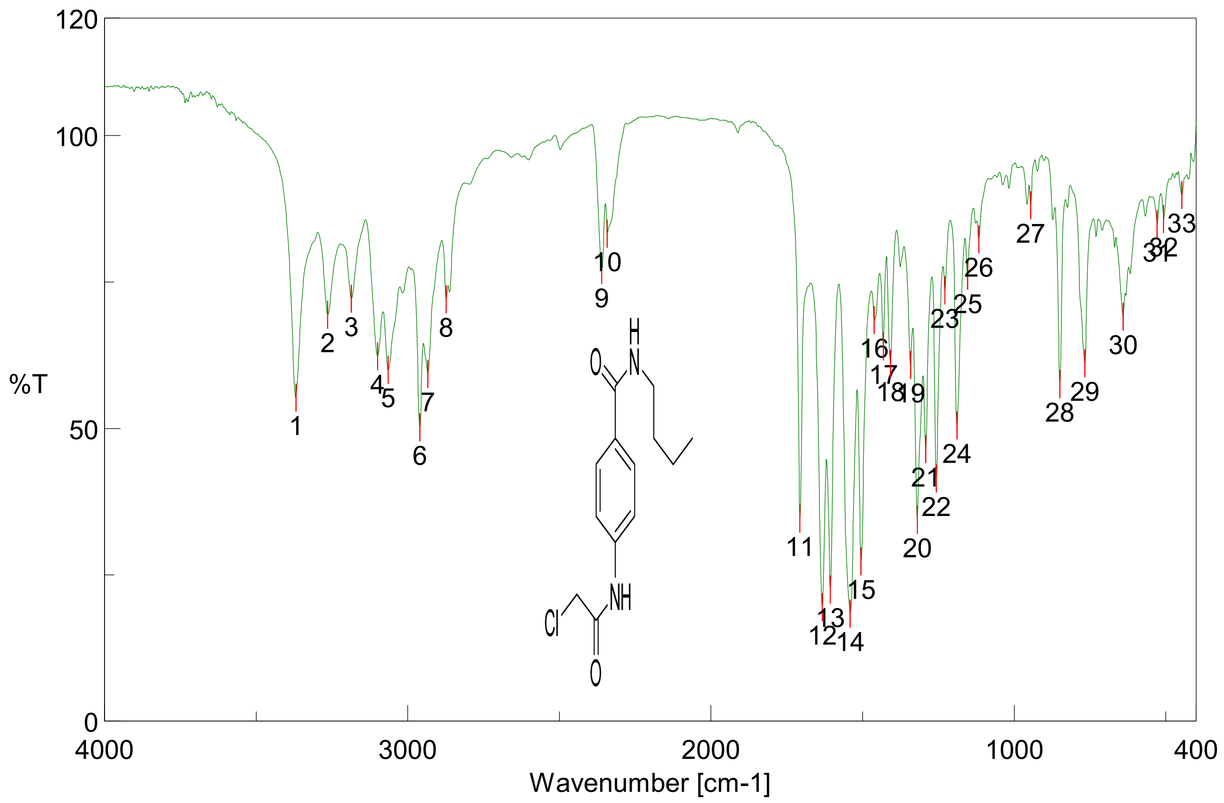

[Comments]

Sample name	A10
Comment	
User	
Division	
Company	KSU

[Detailed Information]

Creation date	9/15/2020 4:03 AM
Data array type	Linear data array
Horizontal axis	Wavenumber [cm-1]
Vertical axis	%T
Start	399.193 cm-1
End	4000.6 cm-1
Data interval	0.964233 cm-1
Data points	3736

[Measurement Information]

Model Name	FT/IR-6600typeA
Serial Number	A014661790
Measurement Date	9/15/2020 4:00 AM
Light Source	Standard
Detector	TGS
Accumulation	Auto (15)
Resolution	4 cm-1
Zero Filling	On
Apodization	Cosine
Gain	Auto (1)
Aperture	Auto (7.1 mm)
Scanning Speed	Auto (2 mm/sec)
Filter	Auto (10000 Hz)

[Result of Peak Picking]

No.	Position	Intensity	No.	Position	Intensity
1	3369.03	55.2078	2	3263.93	69.3748

[Result of Peak Picking]

No.	Position	Intensity	No.	Position	Intensity
3	3185.83	72.1139	4	3099.05	62.2523
5	3064.33	59.9531	6	2960.2	50.158
7	2933.2	59.2398	8	2873.42	72.0007
9	2360.44	77.0263	10	2342.12	83.2085
11	1706.69	34.5857	12	1633.41	19.4538
13	1606.41	22.4255	14	1540.85	18.3533
15	1505.17	27.247	16	1461.78	68.4541
17	1431.89	63.9774	18	1407.78	60.9897
19	1342.21	60.8167	20	1320.04	34.3468
21	1292.07	46.4101	22	1257.36	41.4131
23	1228.43	73.5231	24	1188.9	50.4431
25	1154.19	76.0187	26	1116.58	82.2429
27	945.913	88.0454	28	849.49	57.5179
29	767.53	61.0637	30	641.215	69.056
31	528.4	84.8269	32	507.187	85.6843
33	447.404	89.769			

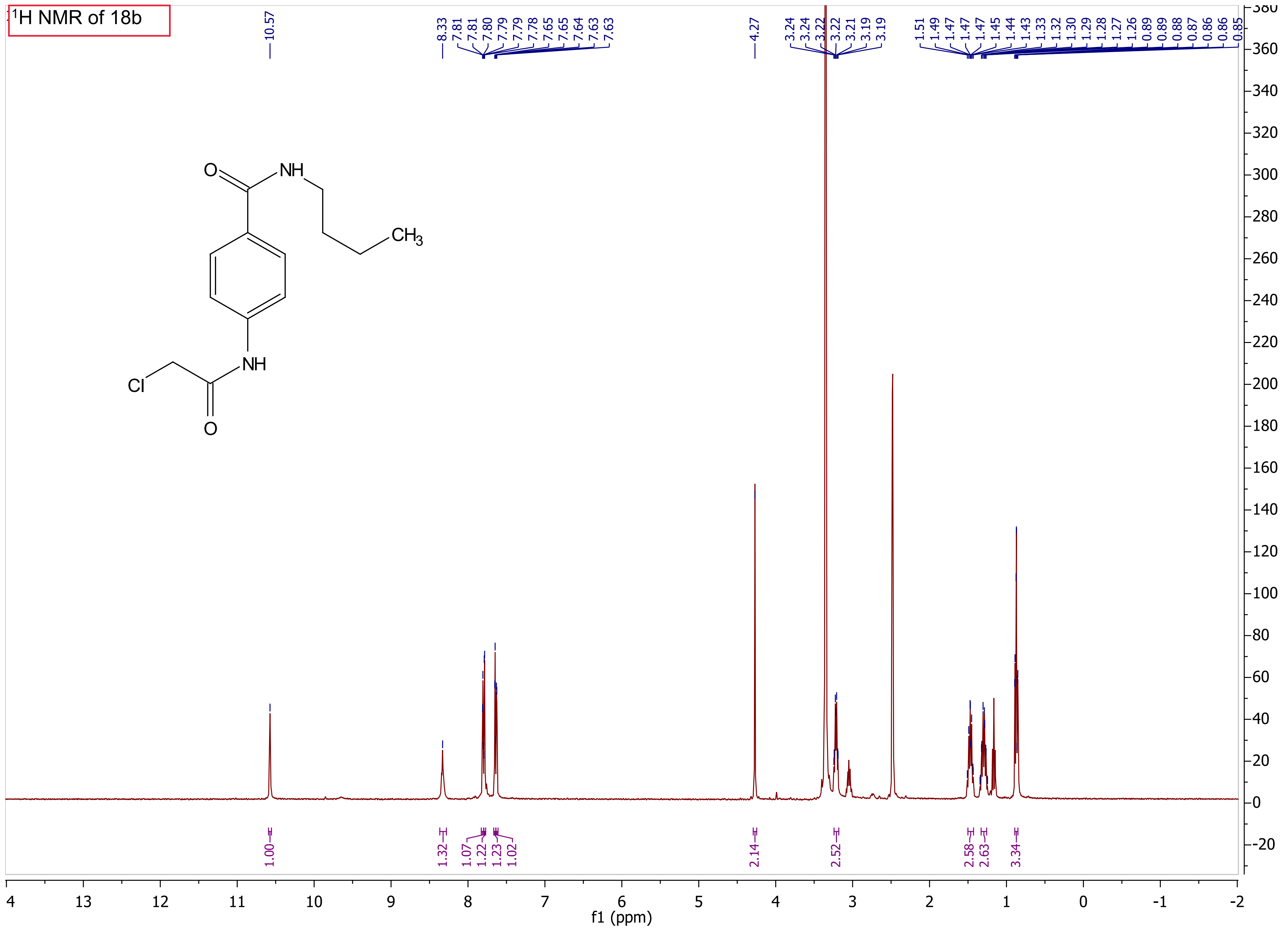

1H NMR of 18b
10.57
8.33 7.81 7.81 7.80 7.79 7.79 7.78 7.65 7.65 7.64 7.63 7.63
4.27
3.24 3.24 3.22 3.22 3.21 3.19 3.19
1.51 1.49 1.47 1.47 1.47 1.45 1.44 1.43 1.33 1.32 1.30 1.29 1.28 1.27 1.26 0.89 0.89 0.88 0.87 0.86 0.86 0.85
O
NH
CH3
NH
Cl
O
1.00
1.32
1.07
1.22
1.23
1.02
2.14
2.52
2.58
2.63
3.34
f1 (ppm)

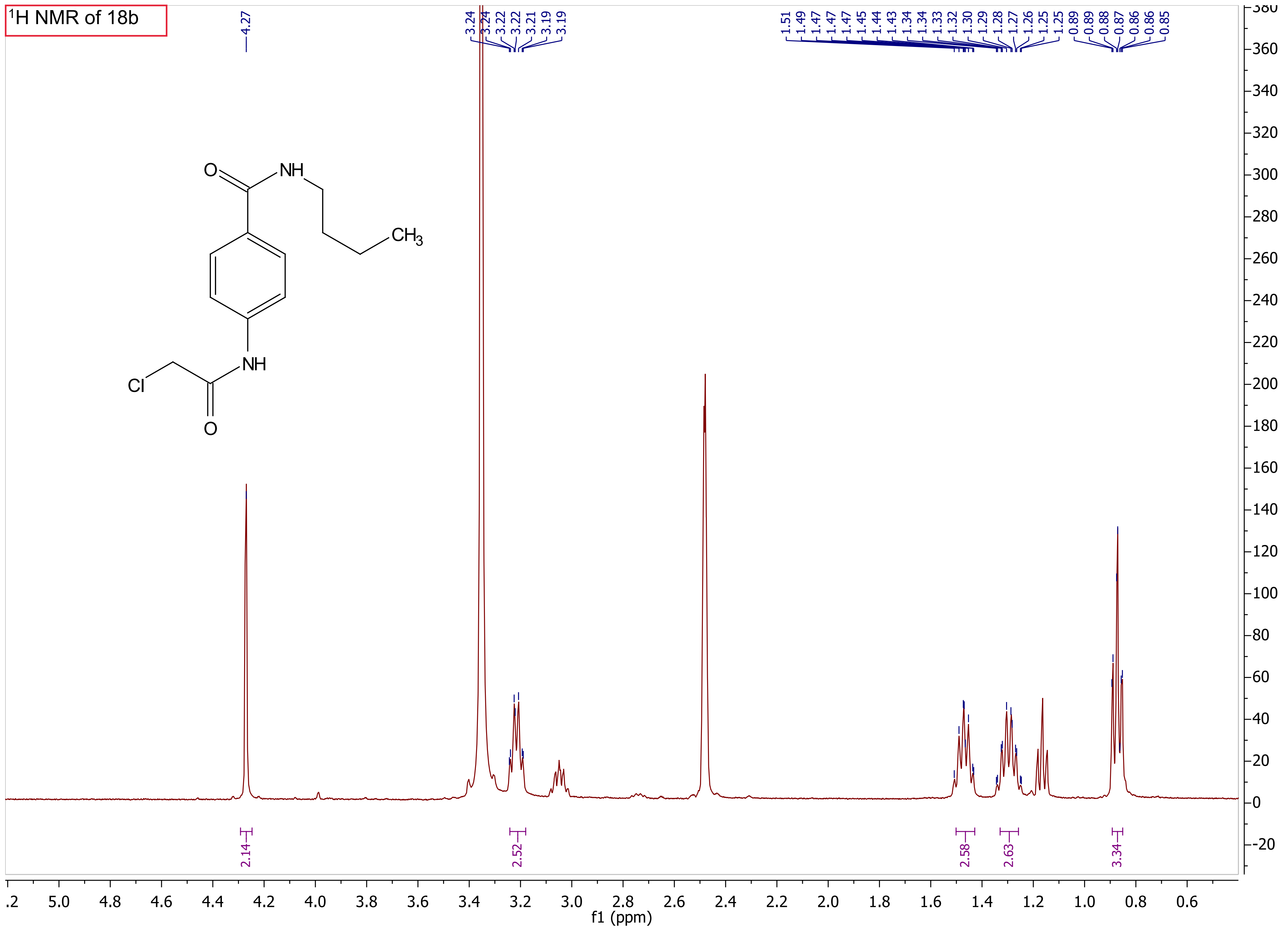

1H NMR of 18b
4.27
3.24
3.24
3.22
3.22
3.21
3.19
3.19
1.51
1.49
1.47
1.47
1.47
1.45
1.44
1.43
1.34
1.34
1.33
1.32
1.30
1.29
1.28
1.27
1.26
1.25
1.25
0.89
0.89
0.88
0.87
0.86
0.86
0.85
O
NH
CH3
NH
Cl
O
2.14
2.52
2.58
2.63
3.34
f1 (ppm)

^{1}H NMR of 18b

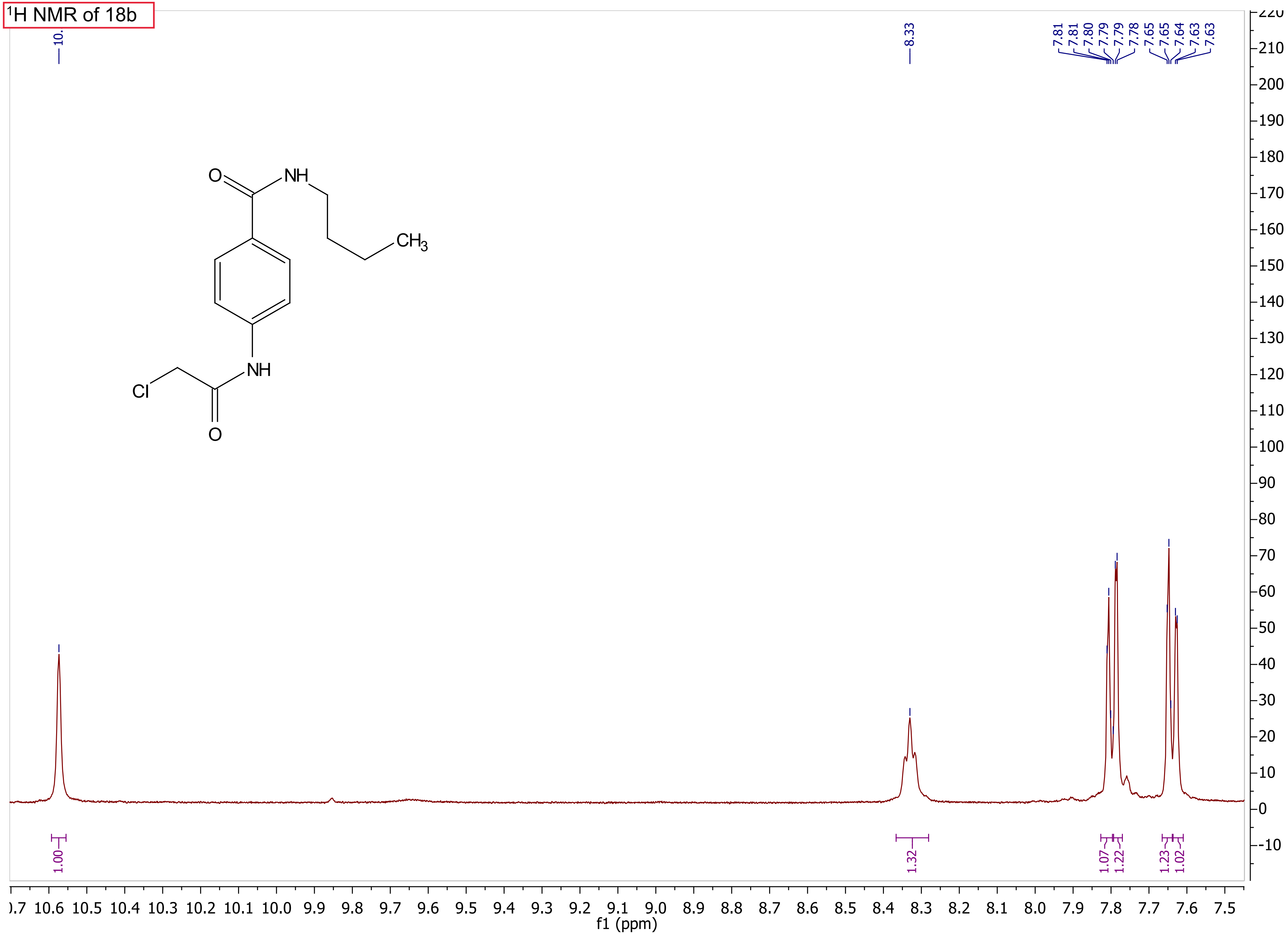

10.
8.33
7.81
7.81
7.80
7.79
7.79
7.78
7.65
7.65
7.64
7.63
7.63
O
NH
CH3
NH
Cl
O
1.00
1.32
1.07
1.22
1.23
1.02
f1 (ppm)

^{13}C NMR of 18b

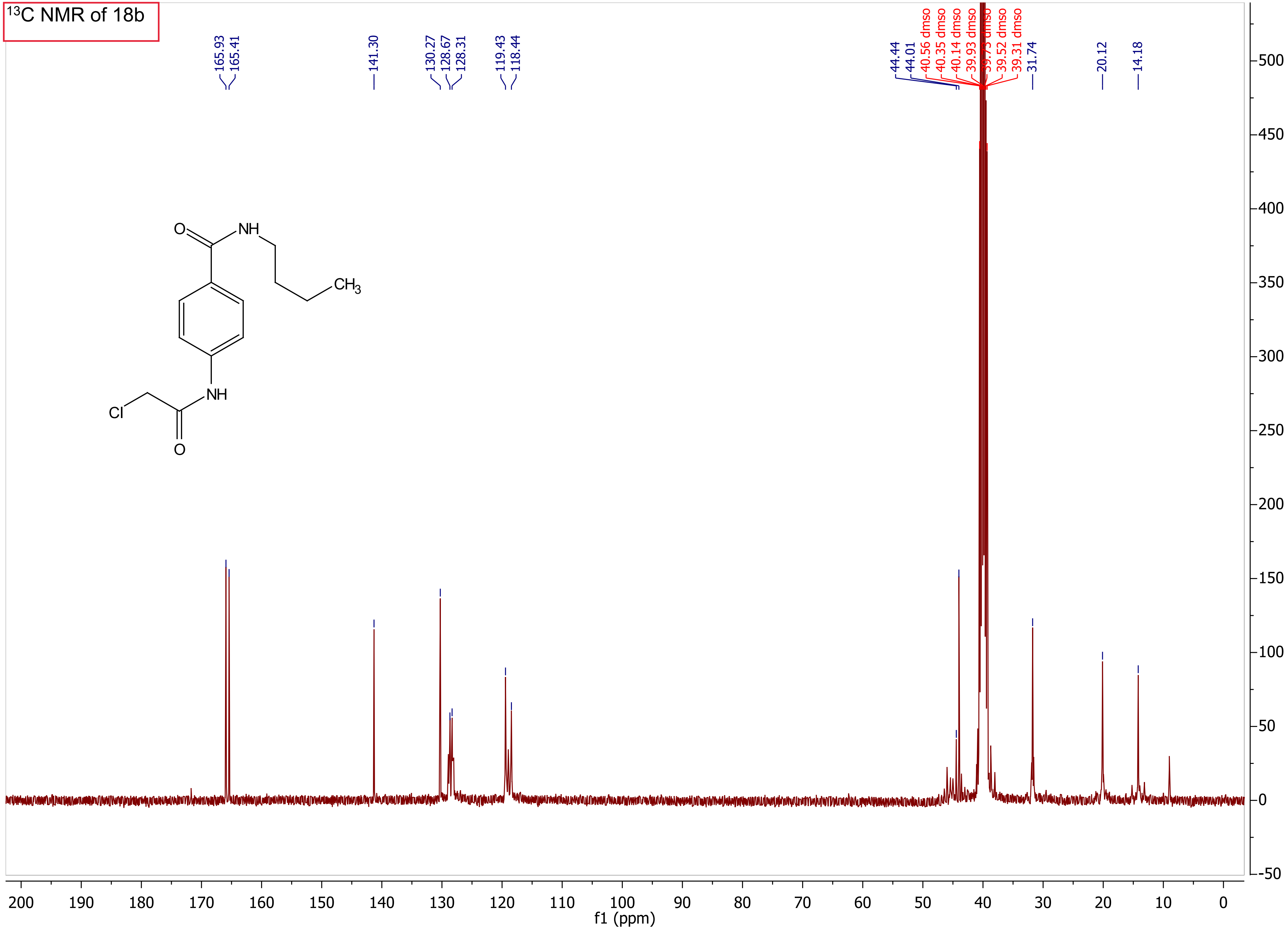

13C NMR of 18b

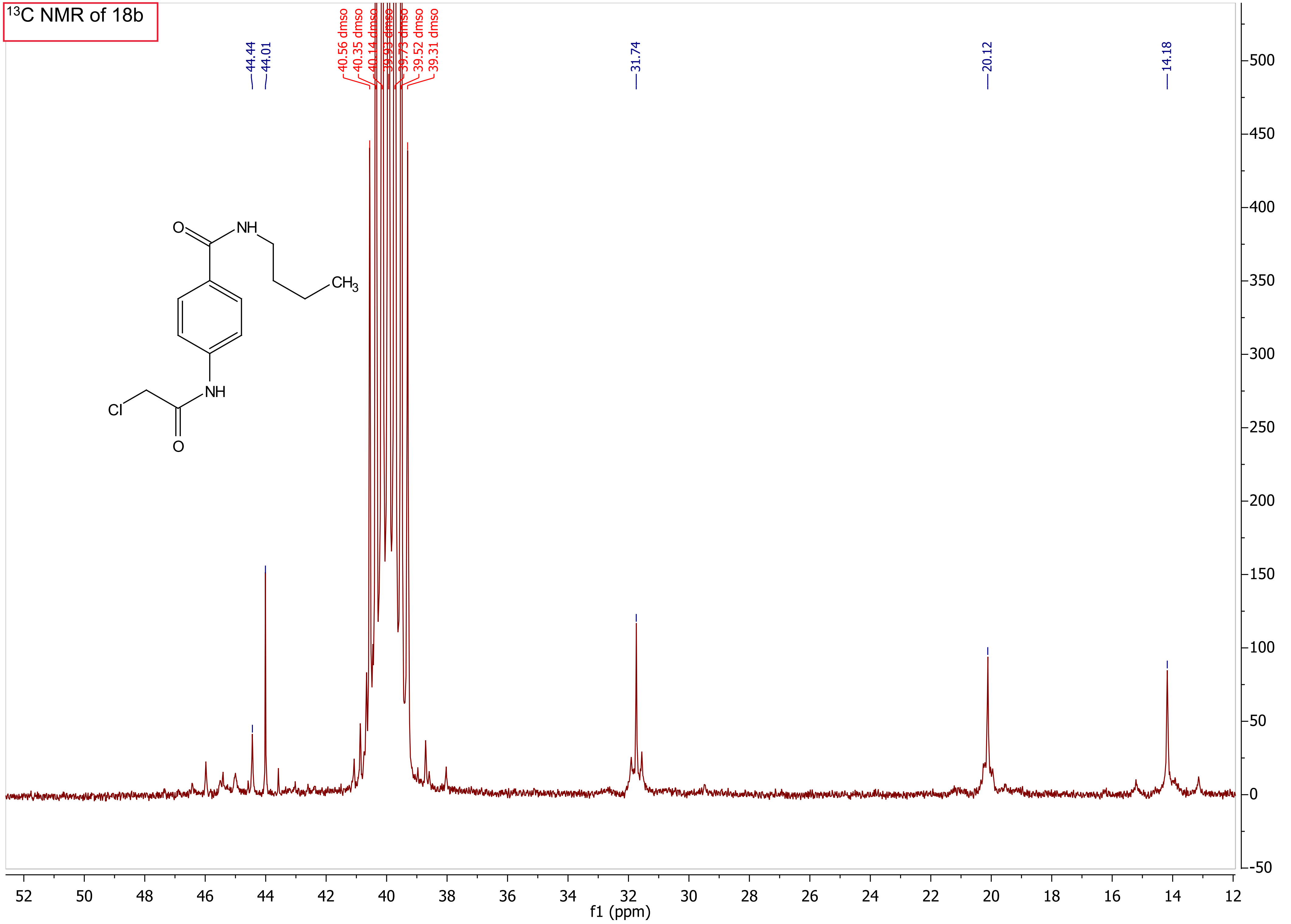

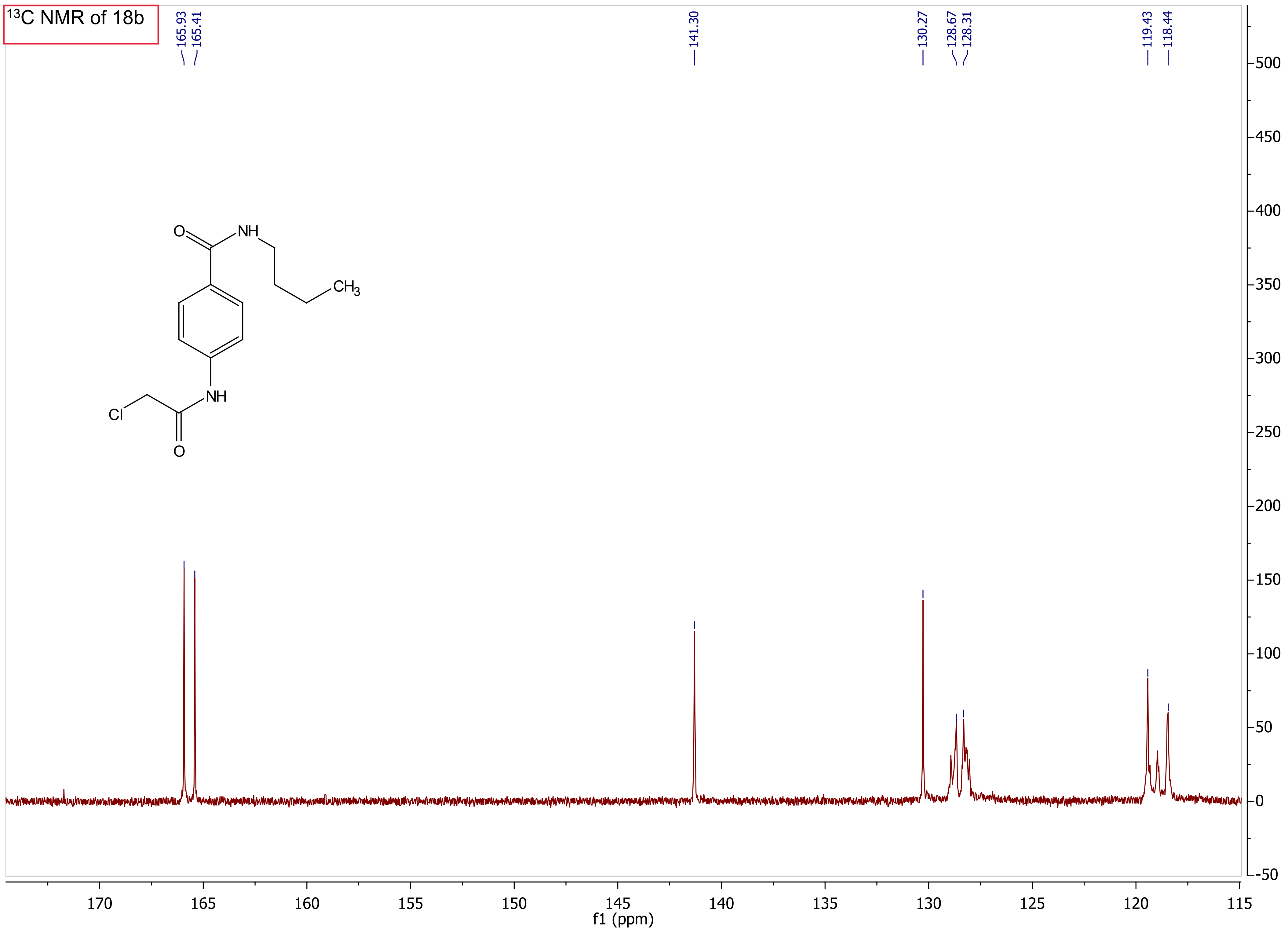

13C NMR of 18b
165.93
165.41
141.30
130.27
128.67
128.31
119.43
118.44
O
NH
CH3
NH
Cl
O
f1 (ppm)
170
165
160
155
150
145
140
135
130
125
120
115
500
450
400
350
300
250
200
150
100
50
0
-50

IR of compound 18c

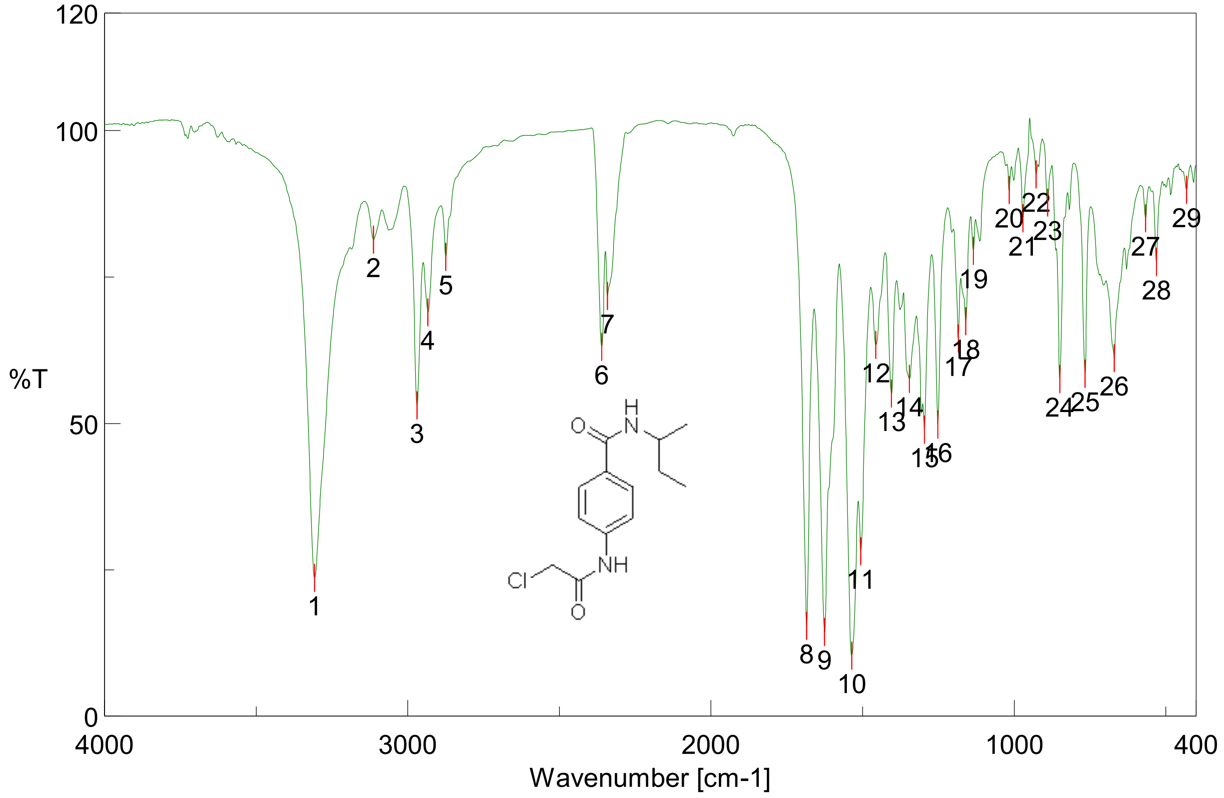

[Comments]

Sample name	A28
Comment	
User	
Division	
Company	KSU

[Detailed Information]

Creation date	9/15/2020 5:02 AM
Data array type	Linear data array
Horizontal axis	Wavenumber [cm-1]
Vertical axis	%T
Start	399.193 cm-1
End	4000.6 cm-1
Data interval	0.964233 cm-1
Data points	3736

[Measurement Information]

Model Name	FT/IR-6600typeA
Serial Number	A014661790
Measurement Date	9/15/2020 5:00 AM
Light Source	Standard
Detector	TGS
Accumulation	Auto (15)
Resolution	4 cm-1
Zero Filling	On
Apodization	Cosine
Gain	Auto (1)
Aperture	Auto (7.1 mm)
Scanning Speed	Auto (2 mm/sec)
Filter	Auto (10000 Hz)

[Result of Peak Picking]

No.	Position	Intensity	No.	Position	Intensity
1	3307.32	23.591	2	3113.51	81.3502

[Result of Peak Picking]

No.	Position	Intensity	No.	Position	Intensity
3	2969.84	53.0596	4	2933.2	68.924
5	2874.38	78.3655	6	2360.44	63.0057
7	2341.16	71.7134	8	1684.52	15.4076
9	1625.7	14.3589	10	1536.02	10.3462
11	1506.13	28.1022	12	1456.96	63.3594
13	1404.89	55.0481	14	1346.07	57.5744
15	1295.93	48.8647	16	1251.58	49.7345
17	1184.08	64.45	18	1159.97	67.3783
19	1134.9	79.4402	20	1016.3	89.7969
21	970.983	84.969	22	927.593	92.4879
23	889.987	87.6732	24	849.49	57.542
25	766.566	58.4487	26	670.142	61.1021
27	566.969	85.008	28	531.293	77.4934
29	431.977	89.8718			

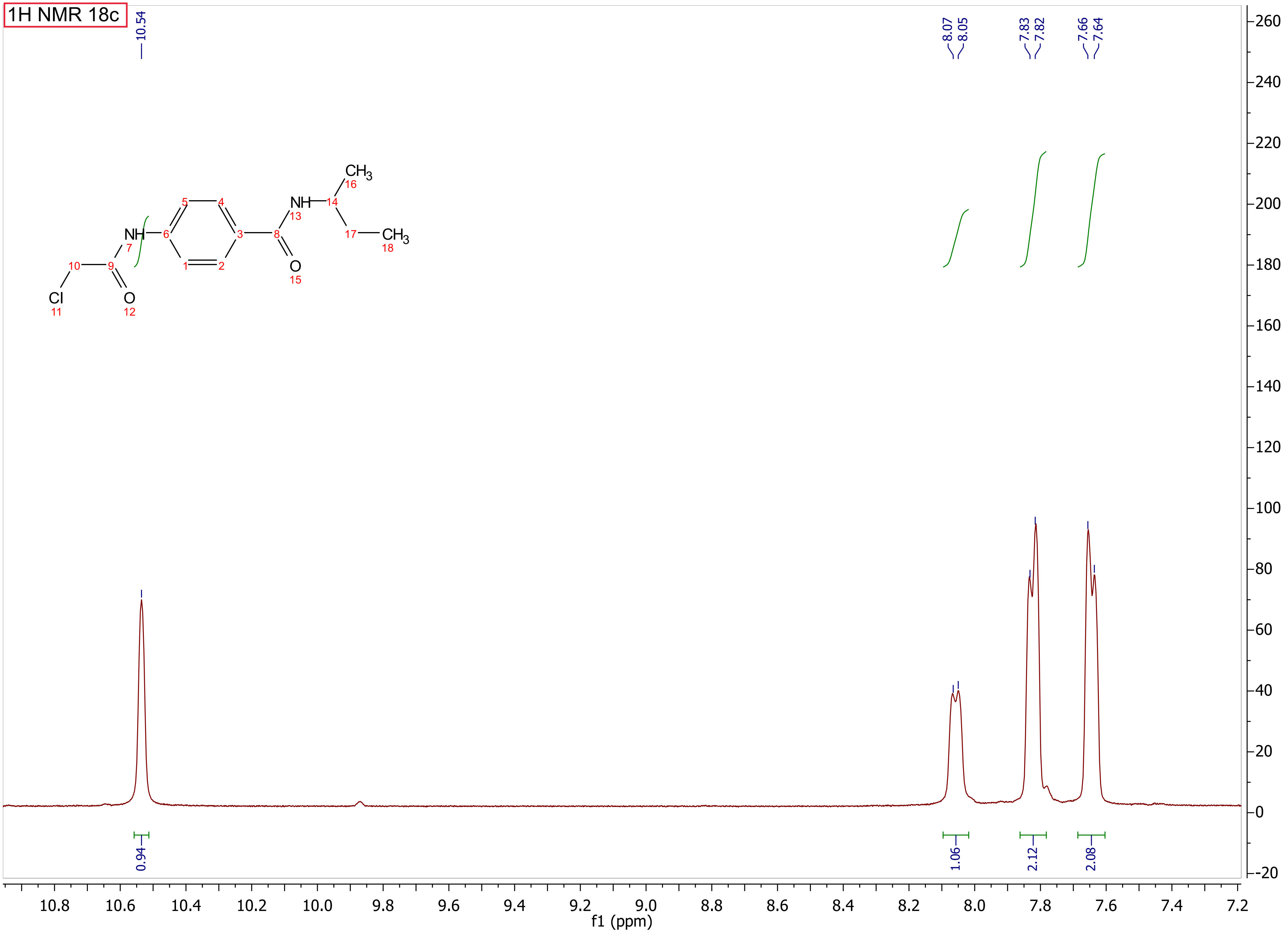
1H NMR 18c
10.54
8.07
8.05
7.83
7.82
7.66
7.64
0.94
1.06
2.12
2.08
CH3
NH
NH
CH3
Cl
O
O
f1 (ppm)
10.8
10.6
10.4
10.2
10.0
9.8
9.6
9.4
9.2
9.0
8.8
8.6
8.4
8.2
8.0
7.8
7.6
7.4
7.2
260
240
220
200
180
160
140
120
100
80
60
40
20
0
-20

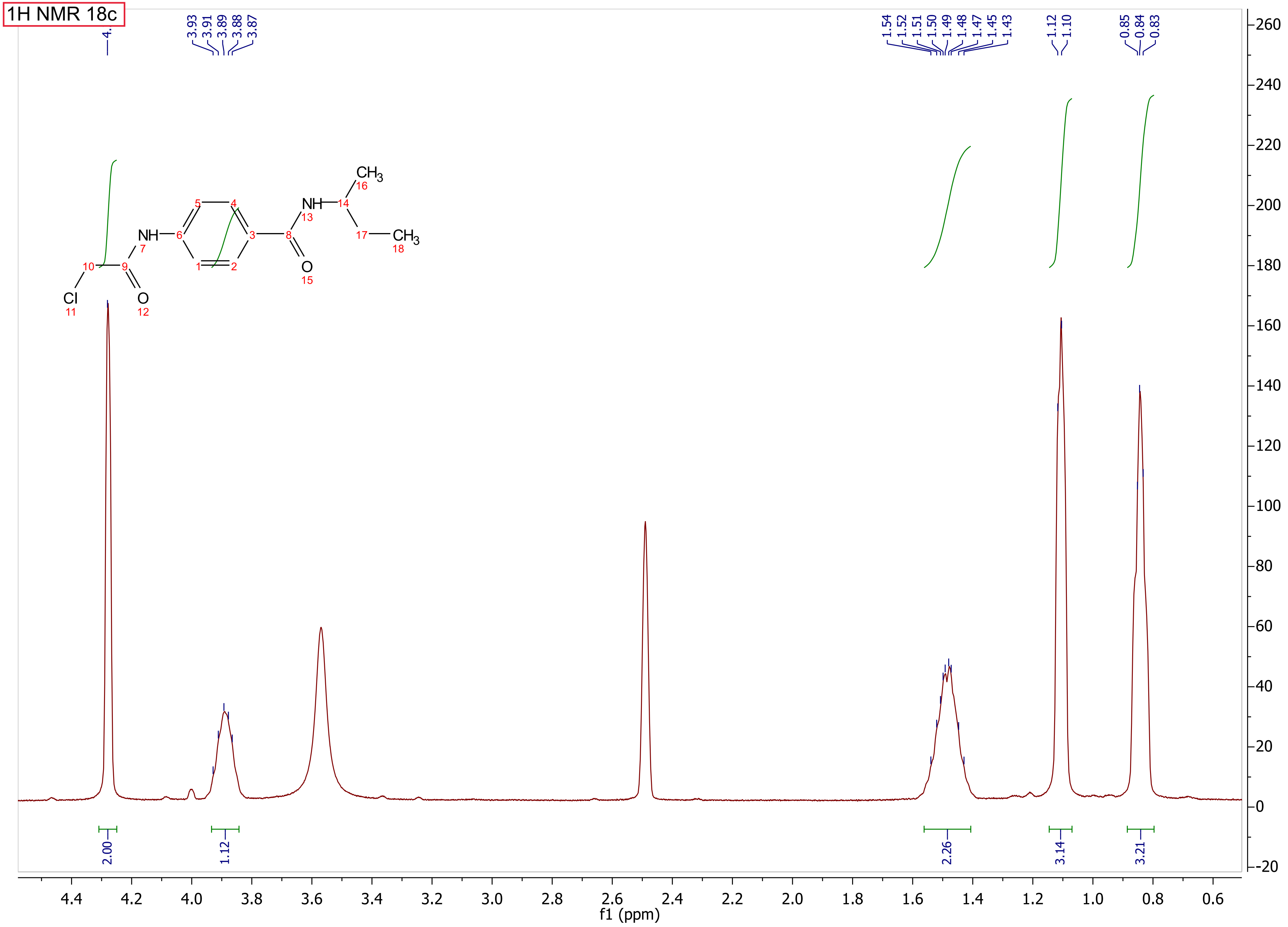
1H NMR 18c
4.
3.93
3.91
3.89
3.88
3.87
1.54
1.52
1.51
1.50
1.49
1.48
1.47
1.45
1.43
1.12
1.10
0.85
0.84
0.83
Cl
O
NH
NH
O
CH3
CH3
2.00
1.12
2.26
3.14
3.21
f1 (ppm)

1H NMR 18c

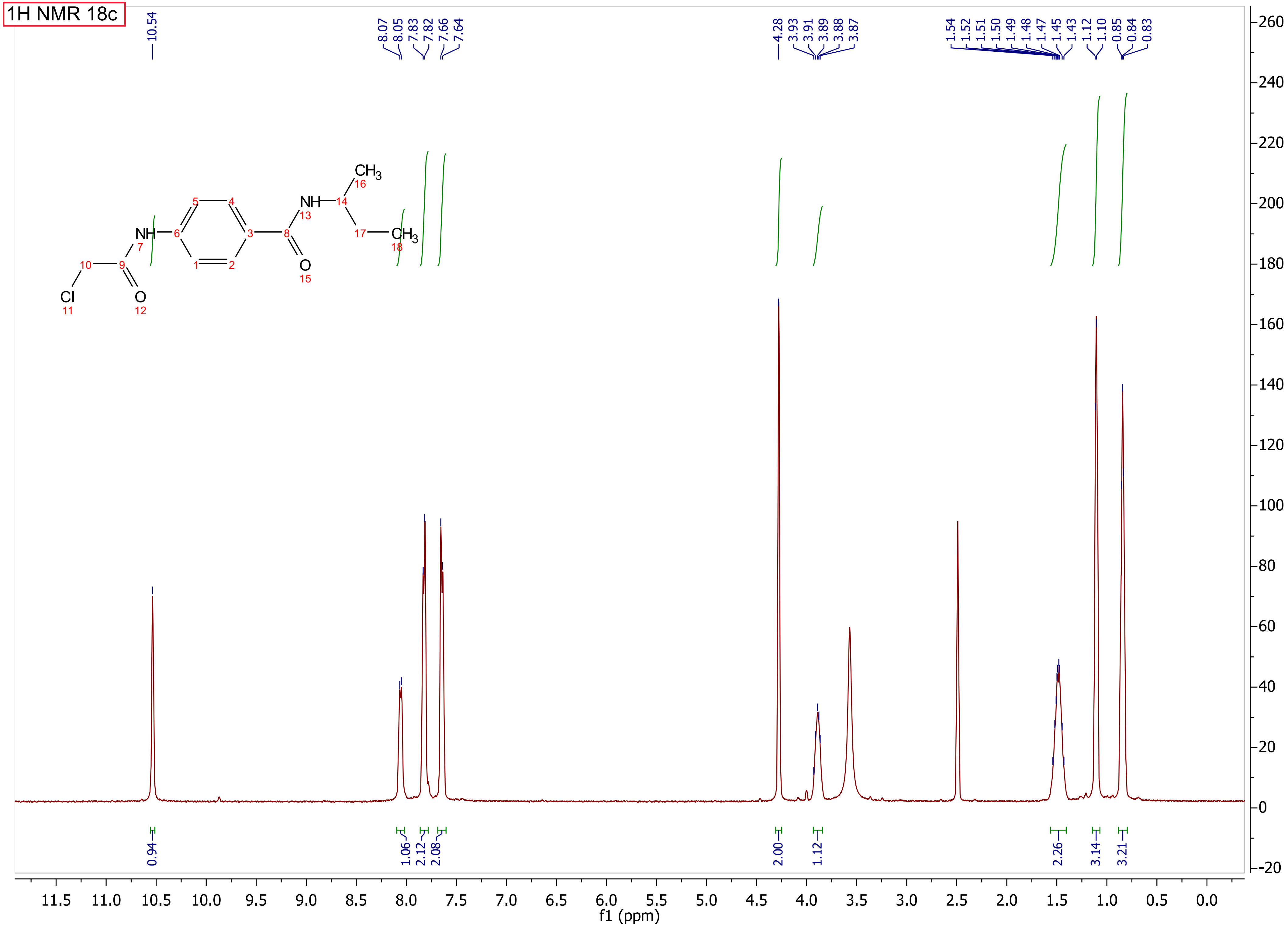
10.54
8.07
8.05
7.83
7.82
7.66
7.64
4.28
3.93
3.91
3.89
3.88
3.87
1.54
1.52
1.51
1.50
1.49
1.48
1.47
1.45
1.43
1.12
1.10
0.85
0.84
0.83
0.94
1.06
2.12
2.08
2.00
1.12
2.26
3.14
3.21
f1 (ppm)

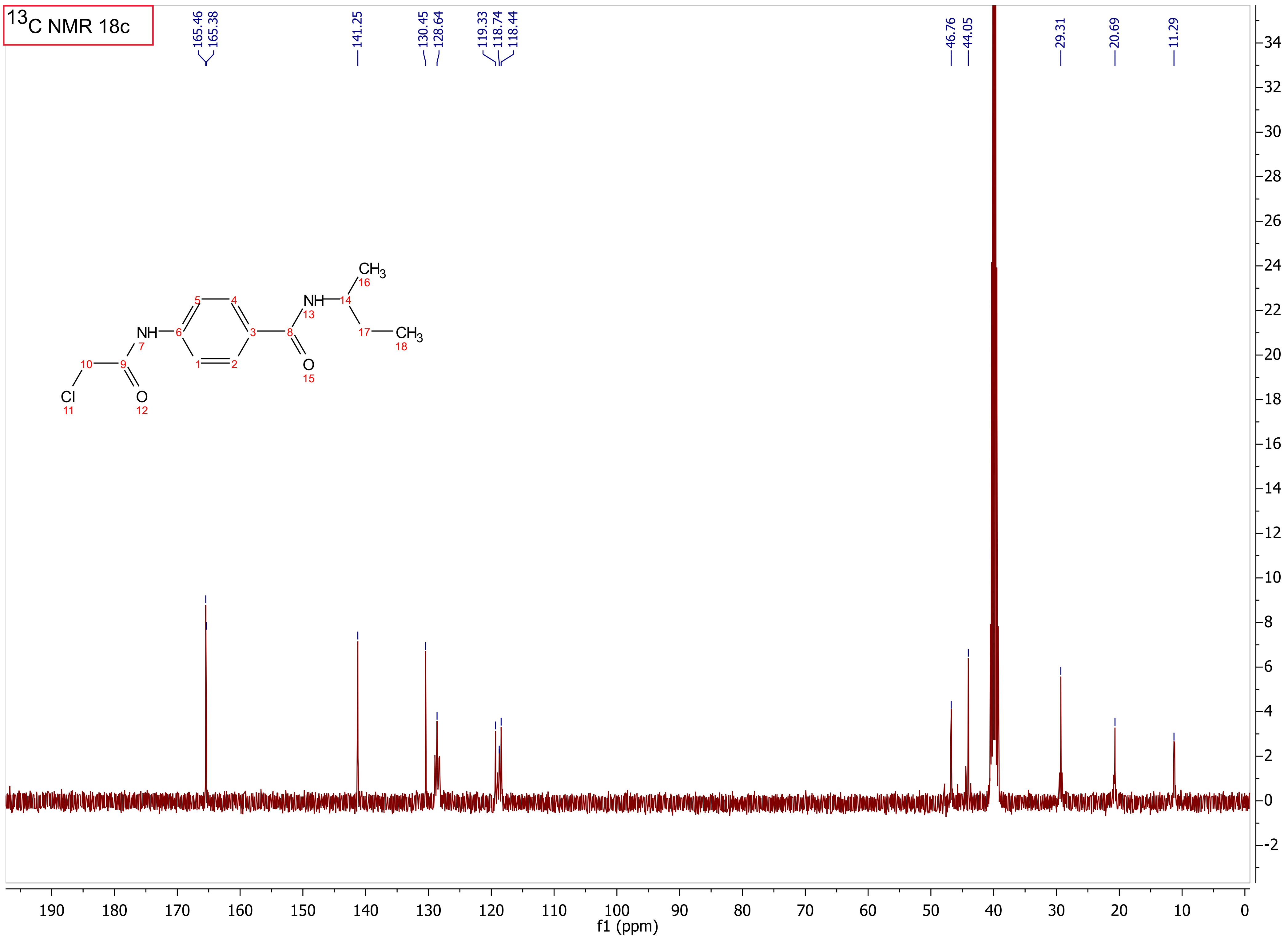

13C NMR 18c
165.46
165.38
141.25
130.45
128.64
119.33
118.74
118.44
46.76
44.05
29.31
20.69
11.29
NH
CH3
Cl
O
f1 (ppm)

IR of compound 18d

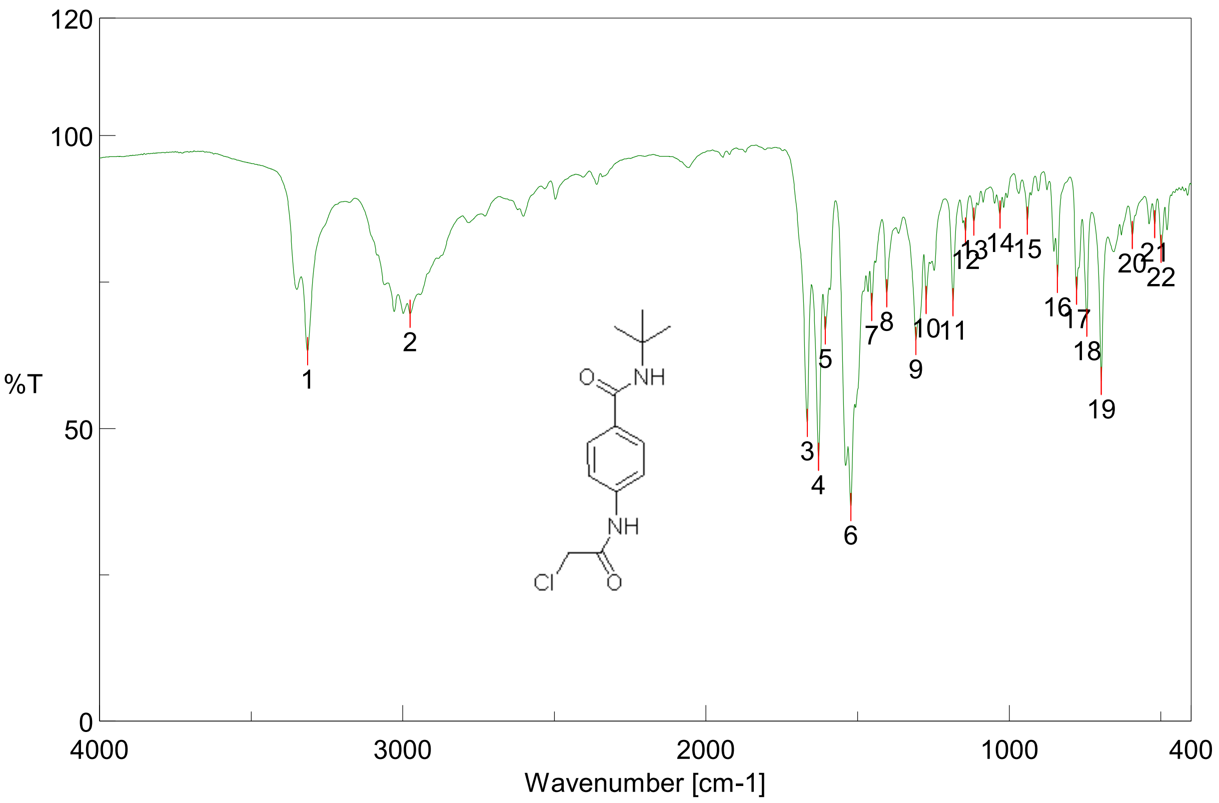

[Comments]

Sample name	A2
Comment	
User	
Division	
Company	KSU

[Detailed Information]

Creation date	9/15/2020 2:31 AM
Date modified	9/15/2020 6:22 AM
Data array type	Linear data array
Horizontal axis	Wavenumber [cm-1]
Vertical axis	%T
Start	399.193 cm-1
End	4000.6 cm-1
Data interval	0.964233 cm-1
Data points	3736

[Measurement Information]

Model Name	FT/IR-6600typeA
Serial Number	A014661790
Measurement Date	9/15/2020 2:30 AM
Light Source	Standard
Detector	TGS
Accumulation	Auto (15)
Resolution	4 cm-1
Zero Filling	On
Apodization	Cosine
Gain	Auto (1)
Aperture	Auto (7.1 mm)
Scanning Speed	Auto (2 mm/sec)
Filter	Auto (10000 Hz)

[Result of Peak Picking]

No.	Position	Intensity	No.	Position	Intensity
1	3314.07	63.145	2	2975.62	69.5193

[Result of Peak Picking]

No.	Position	Intensity	No.	Position	Intensity
3	1666.2	50.9043	4	1628.59	45.0956
5	1606.41	66.7172	6	1522.52	36.5181
7	1454.06	70.6511	8	1403.92	73.0256
9	1307.5	64.8905	10	1273.75	71.8558
11	1185.04	71.4908	12	1144.55	83.5863
13	1116.58	85.2765	14	1030.77	86.4388
15	940.128	85.4109	16	840.812	75.4662
17	778.136	73.4809	18	744.388	67.9765
19	696.177	58.0609	20	593.968	82.9874
21	520.686	84.8388	22	499.473	80.6324

IR of compound 18e

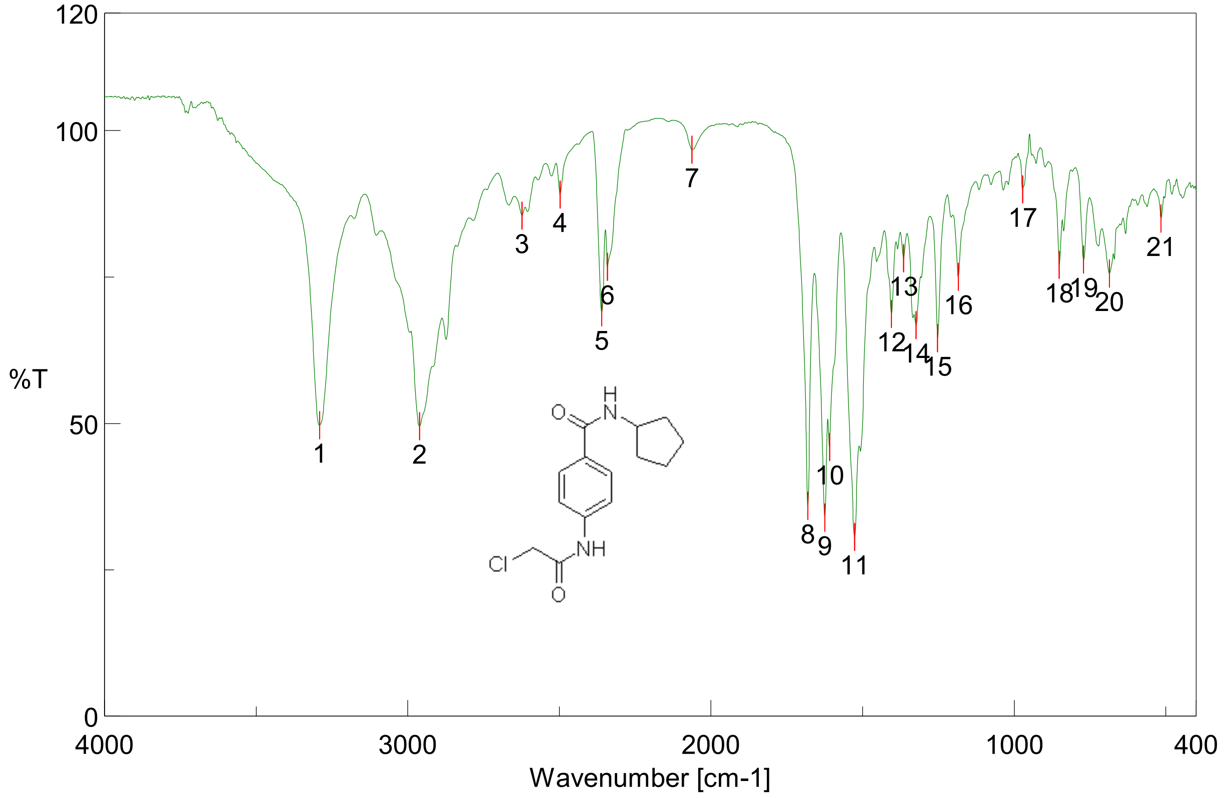

[Comments]

Sample name	A20
Comment	
User	
Division	
Company	KSU

[Detailed Information]

Creation date	9/15/2020 4:33 AM
Data array type	Linear data array
Horizontal axis	Wavenumber [cm-1]
Vertical axis	%T
Start	399.193 cm-1
End	4000.6 cm-1
Data interval	0.964233 cm-1
Data points	3736

[Measurement Information]

Model Name	FT/IR-6600typeA
Serial Number	A014661790
Measurement Date	9/15/2020 4:31 AM
Light Source	Standard
Detector	TGS
Accumulation	Auto (15)
Resolution	4 cm-1
Zero Filling	On
Apodization	Cosine
Gain	Auto (1)
Aperture	Auto (7.1 mm)
Scanning Speed	Auto (2 mm/sec)
Filter	Auto (10000 Hz)

[Result of Peak Picking]

No.	Position	Intensity	No.	Position	Intensity
1	3290.93	49.6376	2	2961.16	49.5065

[Result of Peak Picking]

No.	Position	Intensity	No.	Position	Intensity
3	2623.68	85.4441	4	2497.36	89.0162
5	2360.44	68.9204	6	2341.16	76.6904
7	2062.5	96.6795	8	1680.66	35.8597
9	1624.73	33.8893	10	1609.31	45.9185
11	1526.38	30.6143	12	1404.89	68.6751
13	1365.35	78.1339	14	1323.89	66.7715
15	1252.54	64.5185	16	1184.08	74.9668
17	971.947	89.9258	18	851.418	77.0115
19	771.387	77.9373	20	686.534	75.5368
21	515.865	84.9357			

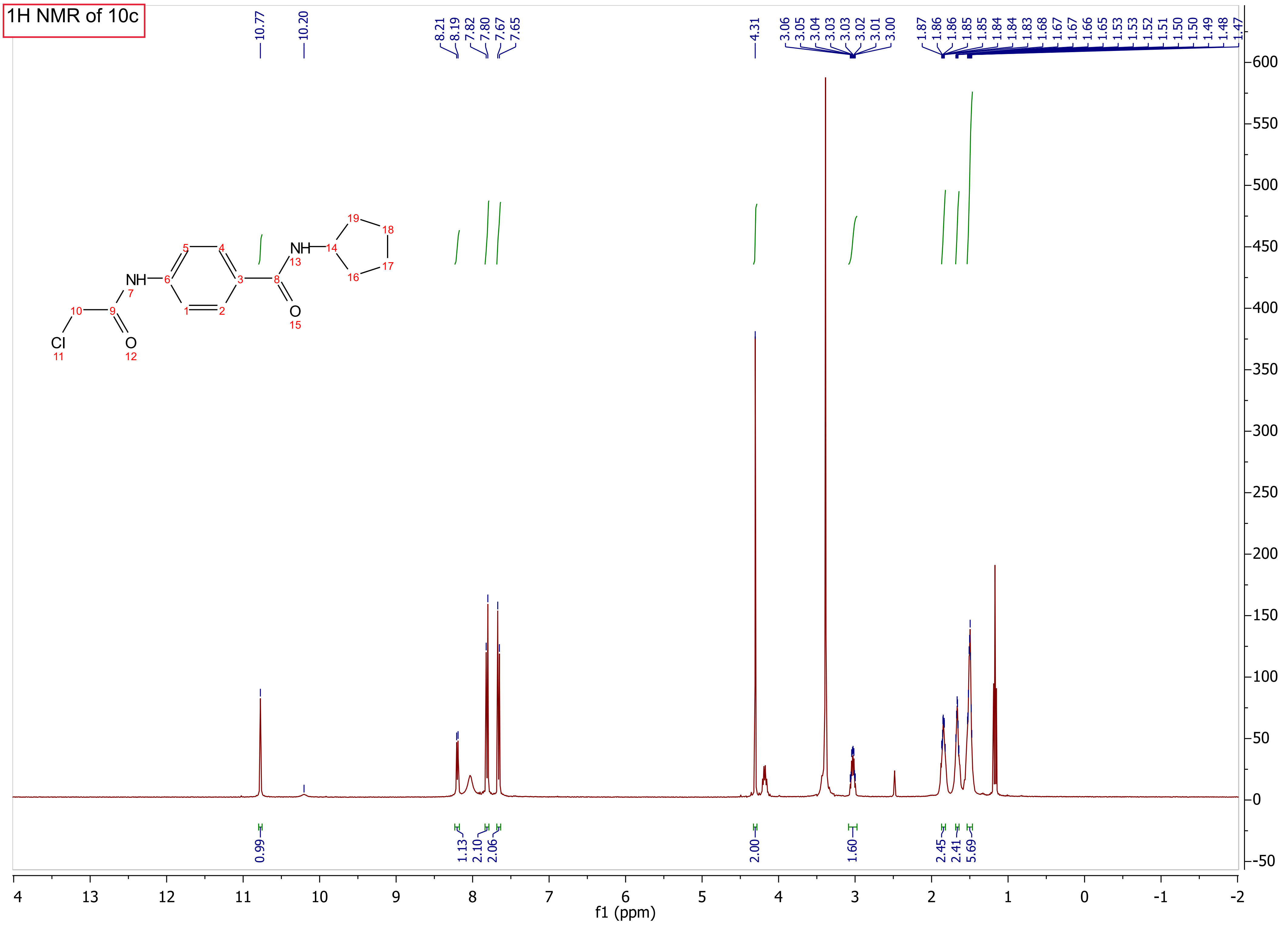
1H NMR of 10c
10.77
10.20
8.21
8.19
7.82
7.80
7.67
7.65
4.31
3.06
3.05
3.04
3.03
3.03
3.02
3.01
3.00
1.87
1.86
1.86
1.85
1.85
1.84
1.84
1.83
1.68
1.67
1.67
1.66
1.65
1.53
1.53
1.52
1.51
1.50
1.50
1.49
1.48
1.47
0.99
1.13
2.10
2.06
2.00
1.60
2.45
2.41
5.69
NH
NH
O
O
Cl
f1 (ppm)

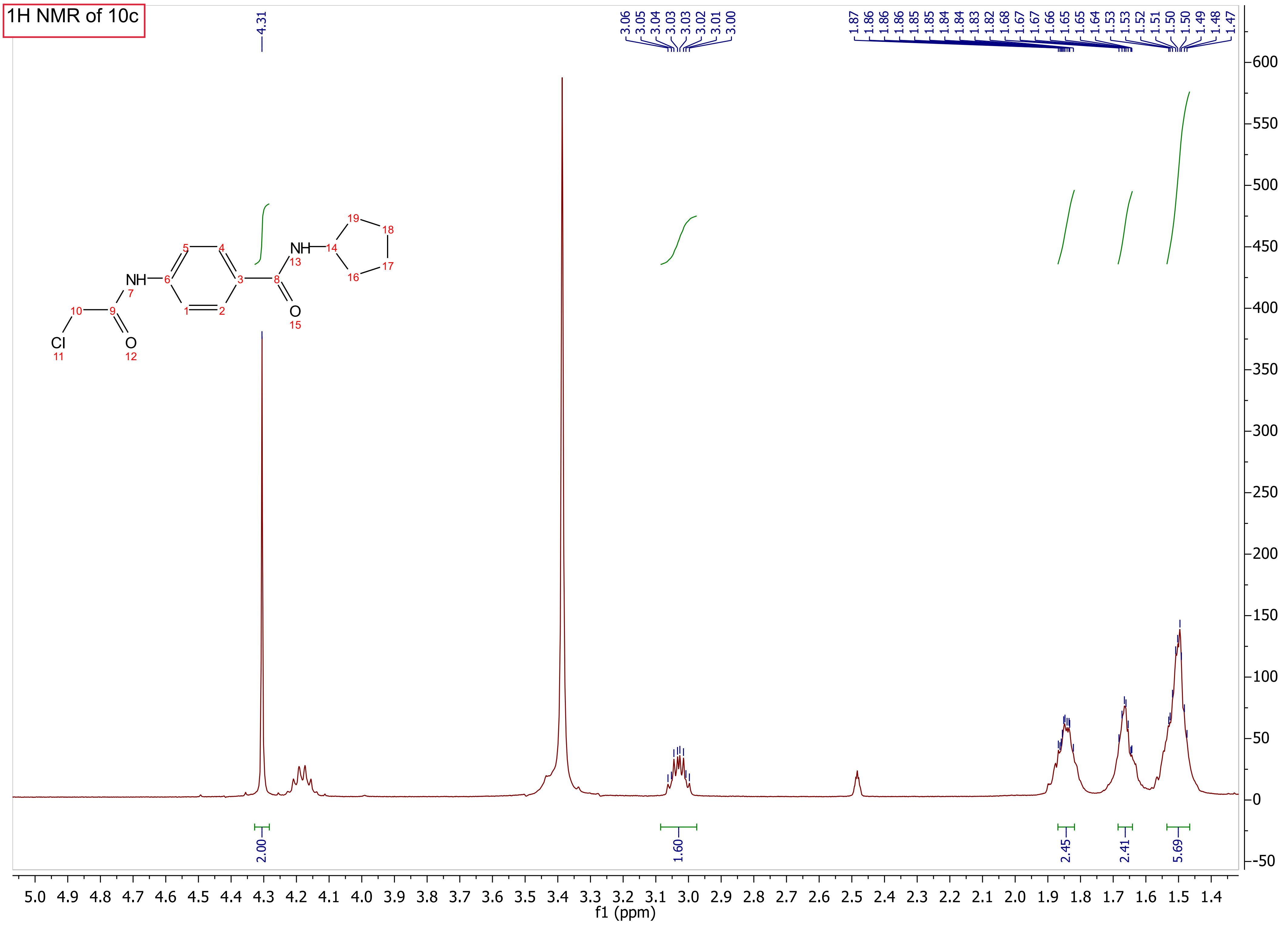
1H NMR of 10c
4.31
3.06 3.05 3.04 3.03 3.03 3.02 3.01 3.00
1.87 1.86 1.86 1.86 1.85 1.85 1.84 1.84 1.83 1.82 1.68 1.67 1.67 1.66 1.65 1.65 1.64 1.53 1.53 1.52 1.51 1.50 1.50 1.49 1.48 1.47
NH
NH
Cl
O
O
2.00
1.60
2.45
2.41
5.69
f1 (ppm)

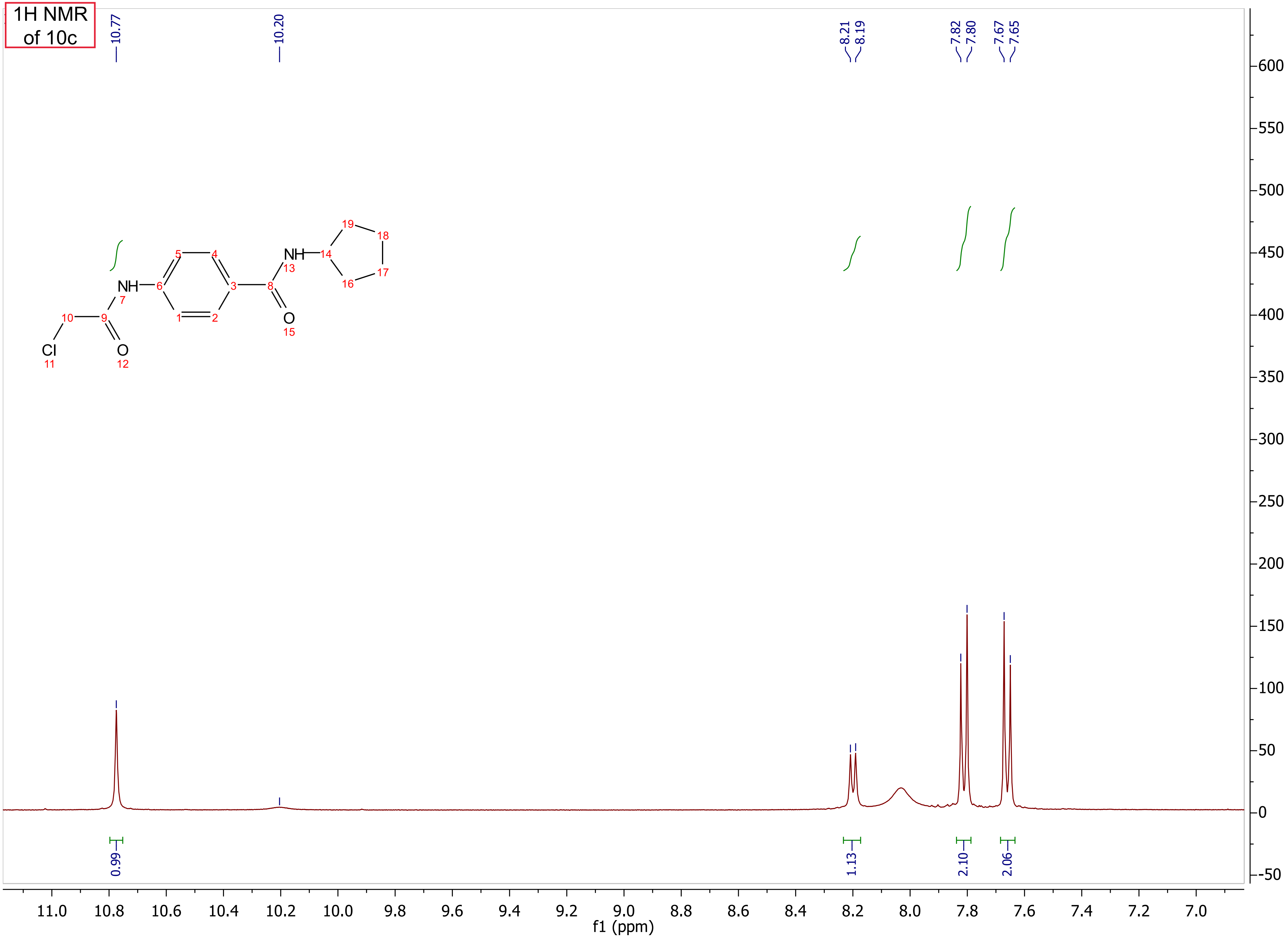
1H NMR of 10c
10.77
10.20
8.21
8.19
7.82
7.80
7.67
7.65
NH
Cl
O
NH
O
0.99
1.13
2.10
2.06
f1 (ppm)

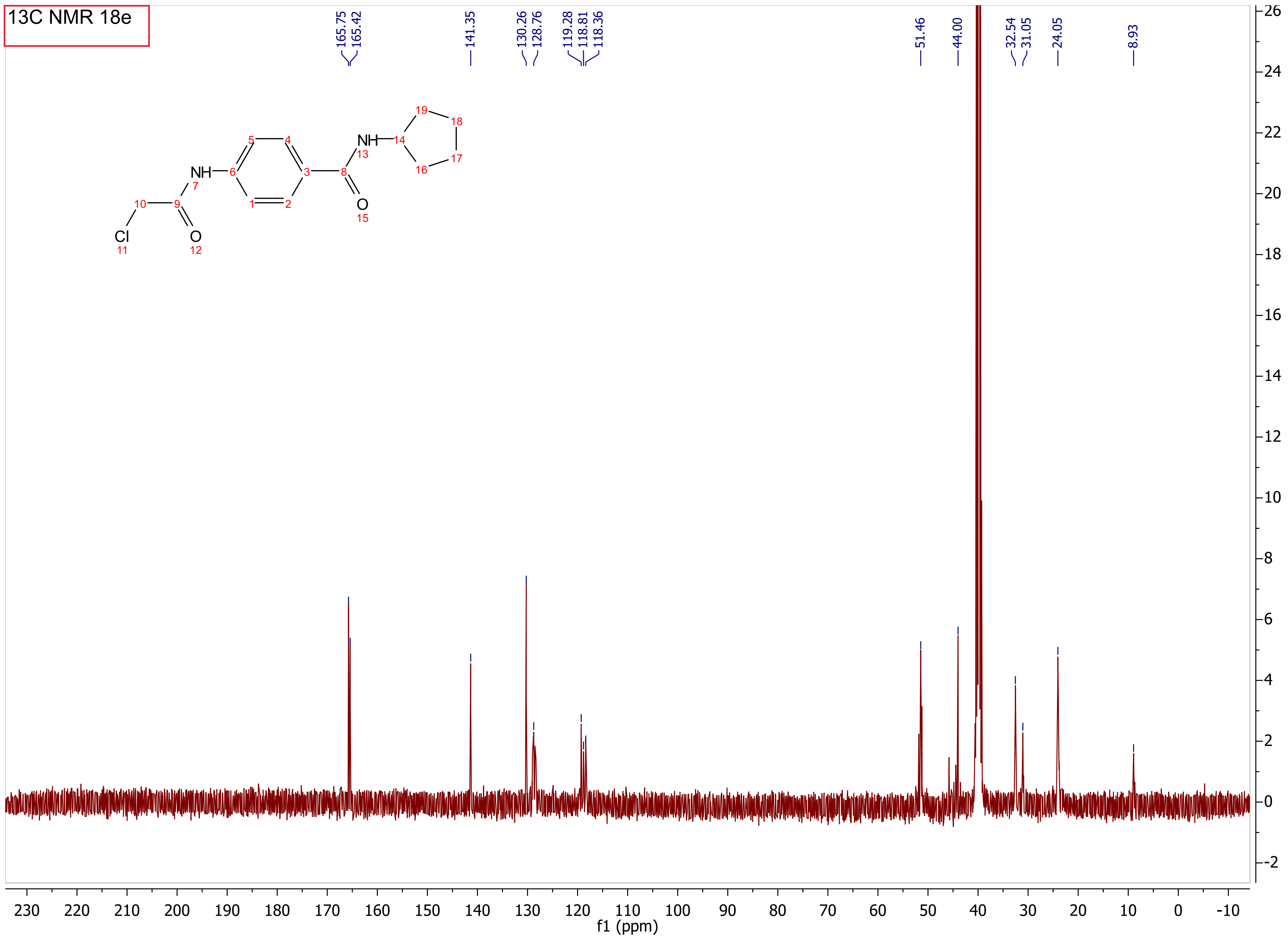
13C NMR 18e
165.75
165.42
141.35
130.26
128.76
119.28
118.81
118.36
51.46
44.00
32.54
31.05
24.05
8.93
NH
7
10
9
Cl
11
O
12
6
5
4
3
1
2
8
O
15
NH
13
14
19
18
17
16
f1 (ppm)

IR of compound 18f

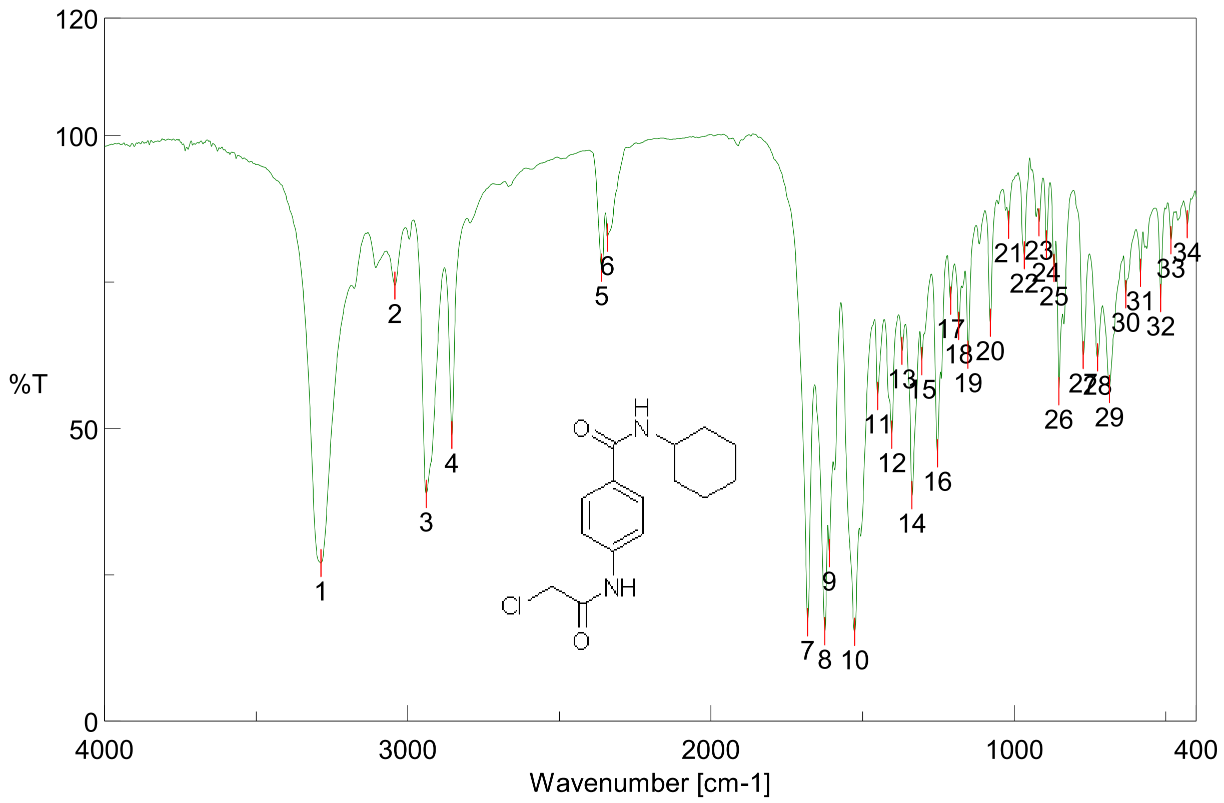

[Comments]

Sample name	A8
Comment	
User	
Division	
Company	KSU

[Detailed Information]

Creation date	9/15/2020 3:54 AM
Data array type	Linear data array
Horizontal axis	Wavenumber [cm-1]
Vertical axis	%T
Start	399.193 cm-1
End	4000.6 cm-1
Data interval	0.964233 cm-1
Data points	3736

[Measurement Information]

Model Name	FT/IR-6600typeA
Serial Number	A014661790
Measurement Date	9/15/2020 3:52 AM
Light Source	Standard
Detector	TGS
Accumulation	Auto (16)
Resolution	4 cm-1
Zero Filling	On
Apodization	Cosine
Gain	Auto (1)
Aperture	Auto (7.1 mm)
Scanning Speed	Auto (2 mm/sec)
Filter	Auto (10000 Hz)

[Result of Peak Picking]

No.	Position	Intensity	No.	Position	Intensity
1	3286.11	26.9925	2	3042.16	74.3627

[Result of Peak Picking]

No.	Position	Intensity	No.	Position	Intensity
3	2938.98	38.8065	4	2854.13	48.8058
5	2360.44	77.41	6	2341.16	82.5525
7	1681.62	16.8924	8	1624.73	15.3373
9	1610.27	28.6916	10	1526.38	15.2244
11	1450.21	55.5012	12	1403.92	48.8665
13	1370.18	63.1973	14	1337.39	38.56
15	1304.61	61.4592	16	1253.5	45.7121
17	1210.11	71.7831	18	1183.11	67.4674
19	1152.26	62.5633	20	1078.98	68.0212
21	1018.23	84.6918	22	967.126	79.5256
23	917.95	85.1677	24	893.844	81.3728
25	868.774	77.3204	26	852.382	56.3147
27	772.351	62.5105	28	725.104	62.0812
29	686.534	56.7105	30	632.537	72.8492
31	583.361	76.5368	32	516.829	72.2319
33	483.081	82.0703	34	429.084	84.8125

^{1}H NMR of 18f

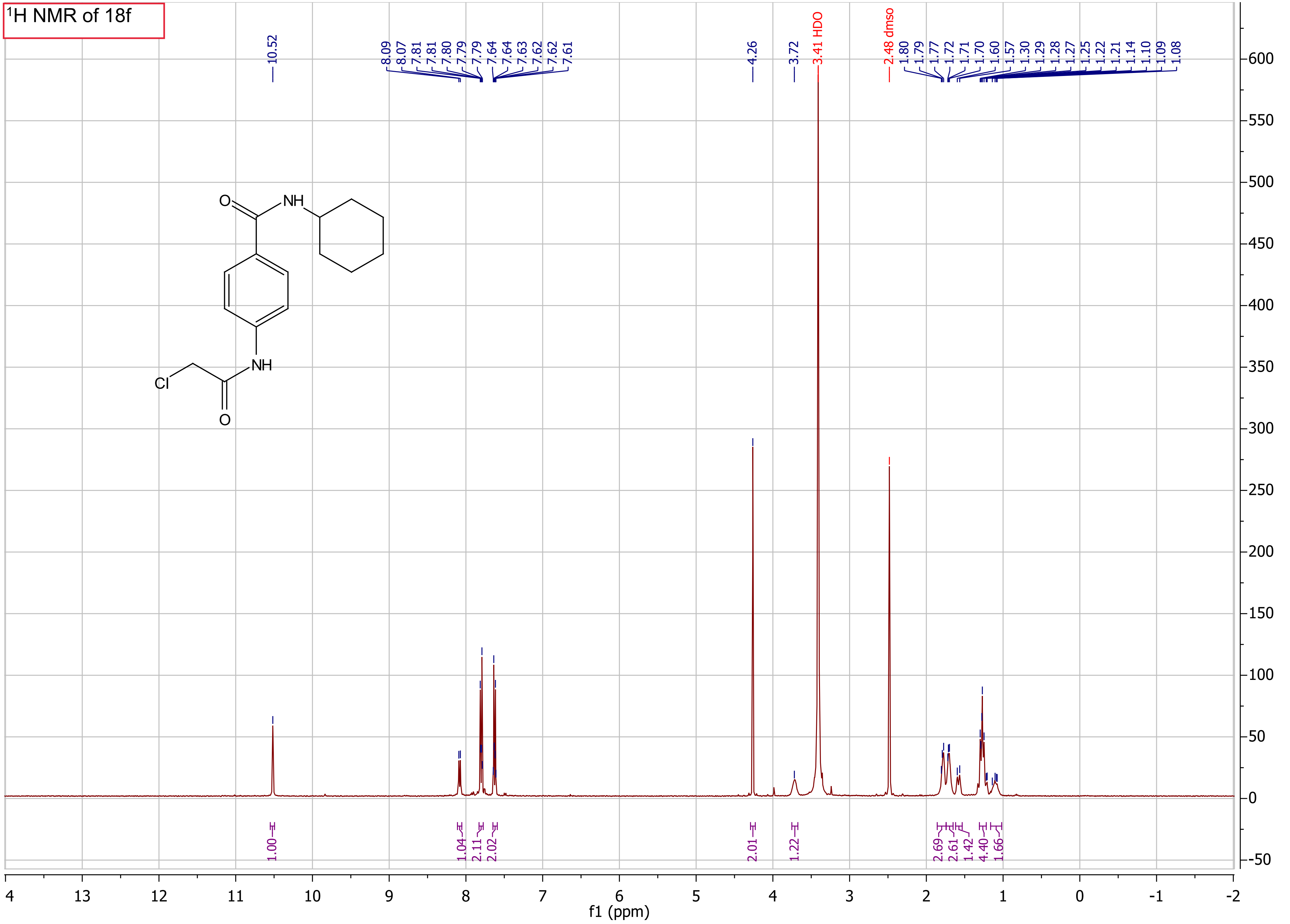

10.52
8.09
8.07
7.81
7.81
7.80
7.79
7.79
7.64
7.64
7.63
7.62
7.62
7.61
4.26
3.72
3.41 HDO
2.48 dmso
1.80
1.79
1.77
1.72
1.71
1.70
1.60
1.57
1.30
1.29
1.28
1.27
1.25
1.22
1.21
1.14
1.10
1.09
1.08
O
NH
NH
Cl
O
1.00
1.04
2.11
2.02
2.01
1.22
2.69
2.61
1.42
4.40
1.66
f1 (ppm)

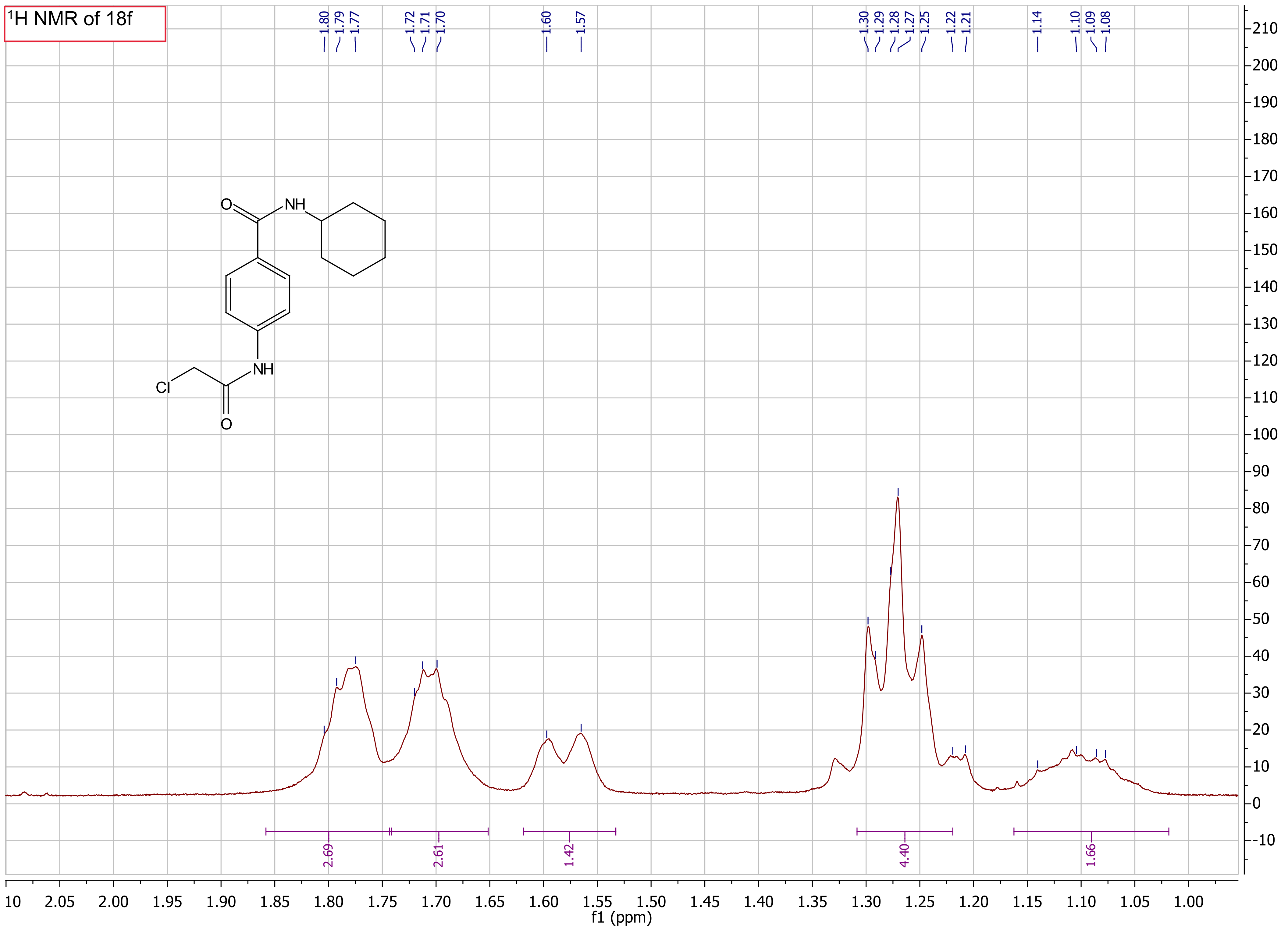

1H NMR of 18f
1.80
1.79
1.77
1.72
1.71
1.70
1.60
1.57
1.30
1.29
1.28
1.27
1.25
1.22
1.21
1.14
1.10
1.09
1.08
O
NH
NH
Cl
O
2.69
2.61
1.42
4.40
1.66
f1 (ppm)

^1H NMR of 18f

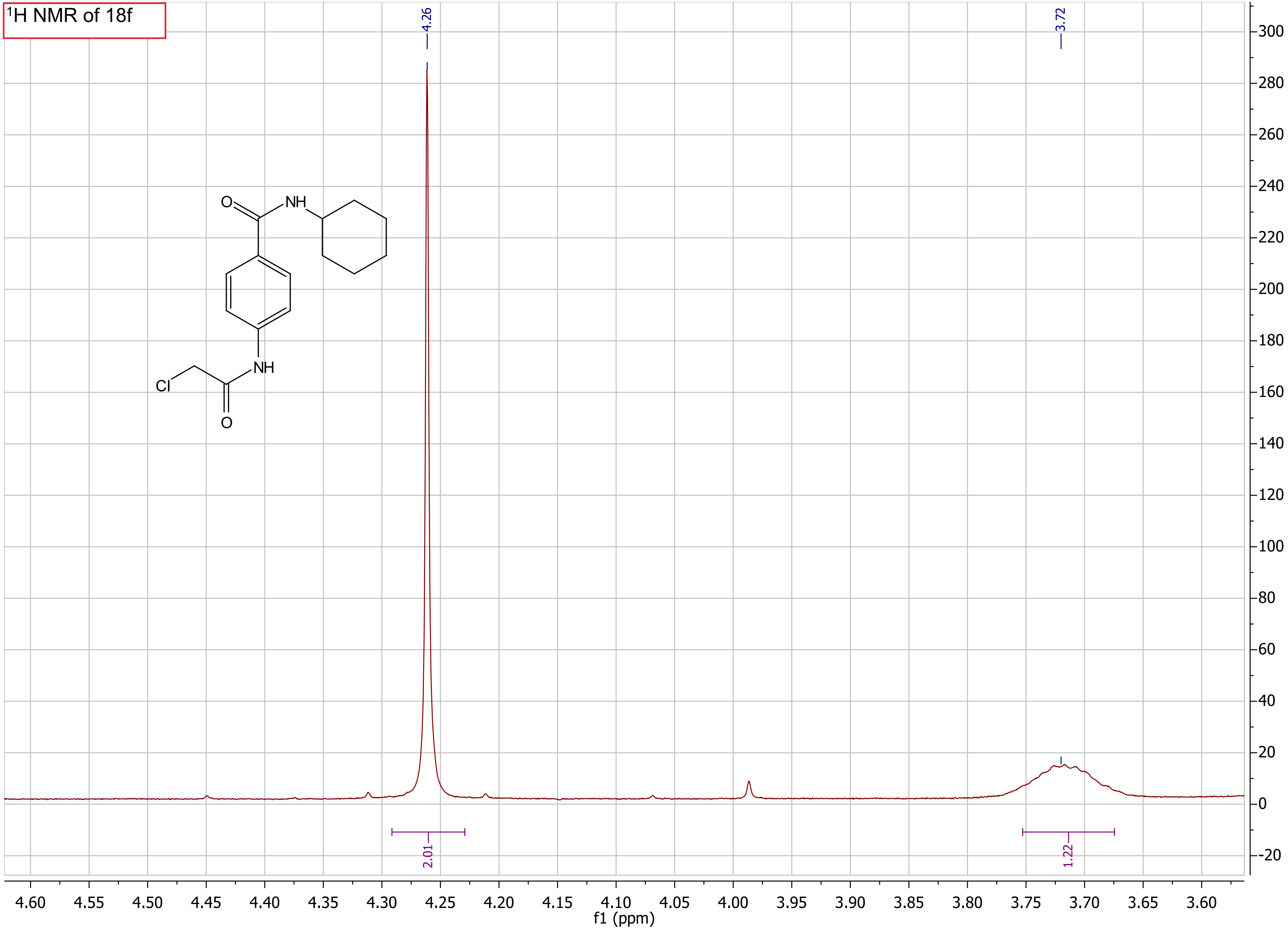

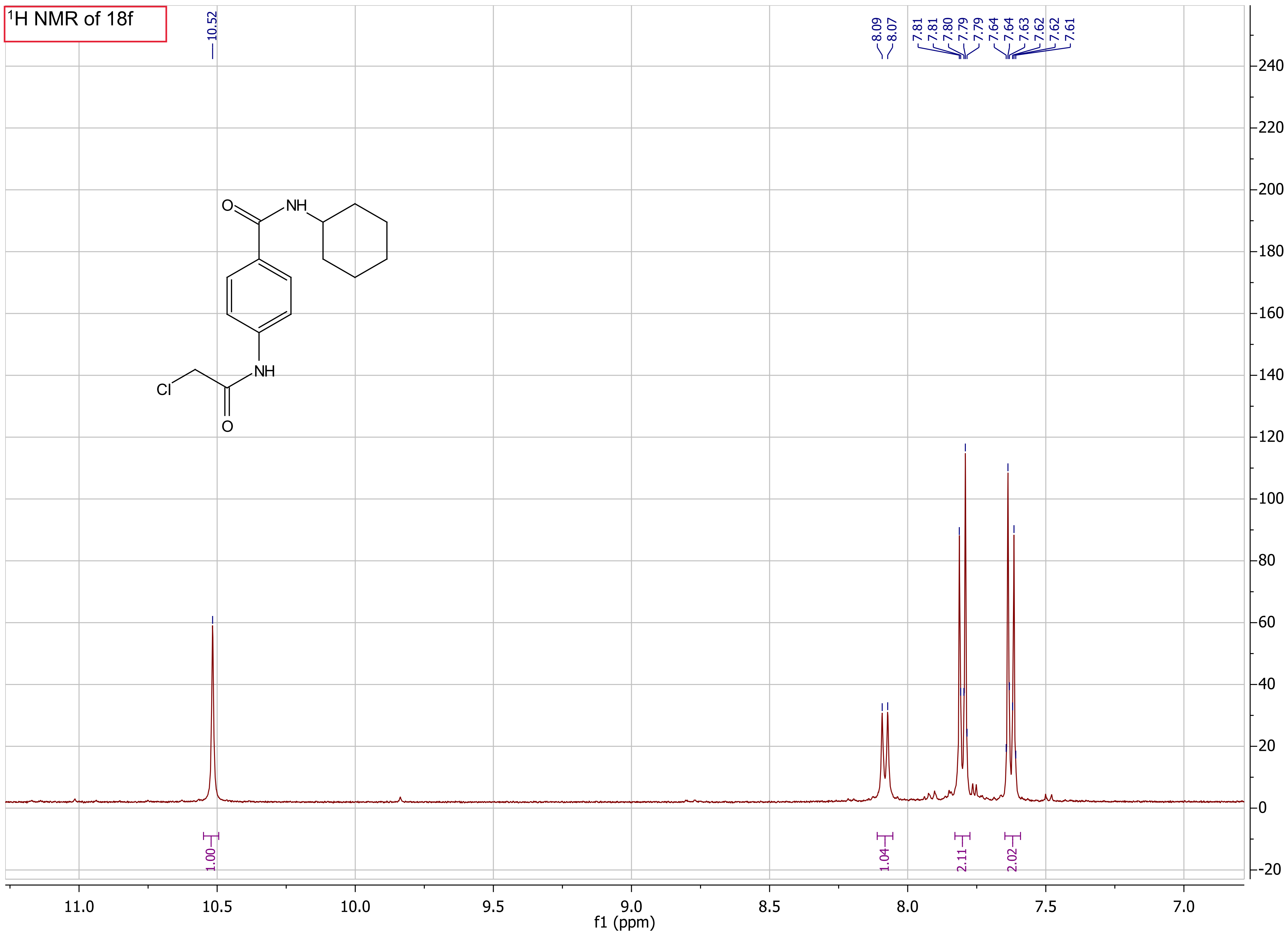

1H NMR of 18f
10.52
8.09
8.07
7.81
7.81
7.80
7.79
7.79
7.64
7.64
7.63
7.62
7.62
7.61
O
NH
NH
Cl
O
1.00
1.04
2.11
2.02
f1 (ppm)

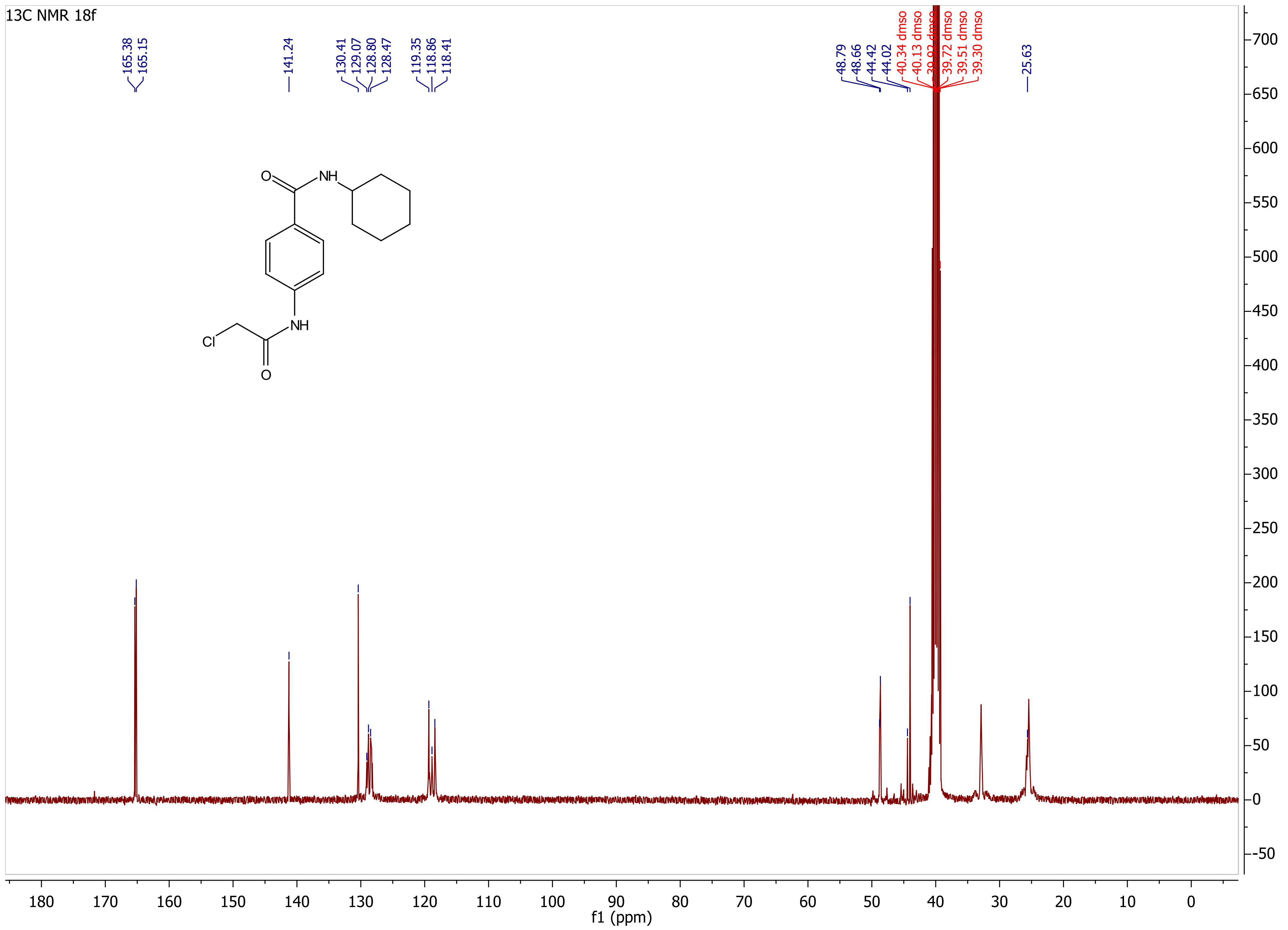
13C NMR 18f
165.38
165.15
141.24
130.41
129.07
128.80
128.47
119.35
118.86
118.41
48.79
48.66
44.42
44.02
40.34 dmso
40.13 dmso
39.72 dmso
39.51 dmso
39.30 dmso
25.63
O
NH
NH
Cl
O
f1 (ppm)

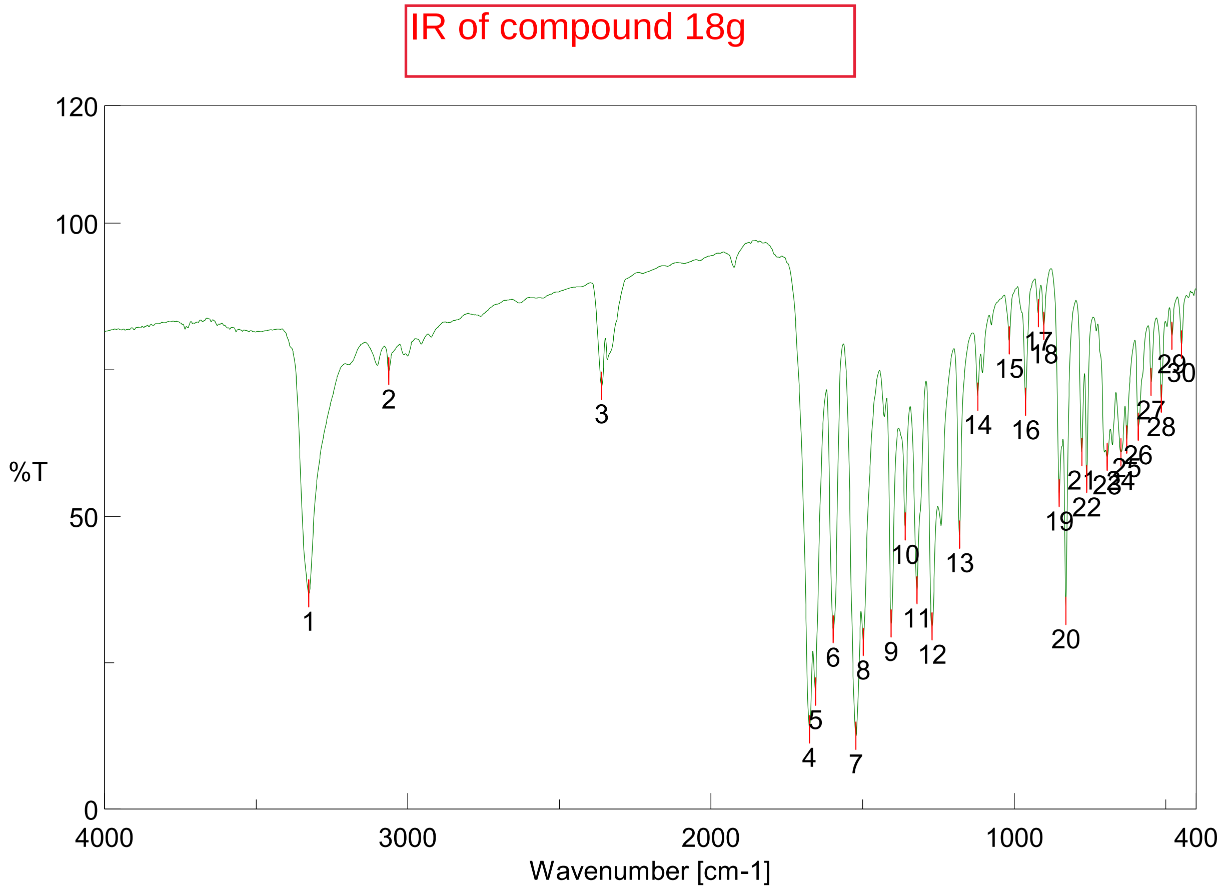

[Comments]

Sample name	A7
Comment	
User	
Division	
Company	KSU

[Detailed Information]

Creation date	9/15/2020 3:46 AM
Data array type	Linear data array
Horizontal axis	Wavenumber [cm-1]
Vertical axis	%T
Start	399.193 cm-1
End	4000.6 cm-1
Data interval	0.964233 cm-1
Data points	3736

[Measurement Information]

Model Name	FT/IR-6600typeA
Serial Number	A014661790
Measurement Date	9/15/2020 3:46 AM
Light Source	Standard
Detector	TGS
Accumulation	Auto (17)
Resolution	4 cm-1
Zero Filling	On
Apodization	Cosine
Gain	Auto (2)
Aperture	Auto (7.1 mm)
Scanning Speed	Auto (2 mm/sec)
Filter	Auto (10000 Hz)

[Result of Peak Picking]

No.	Position	Intensity	No.	Position	Intensity
1	3326.61	36.8318	2	3062.41	74.7154

[Result of Peak Picking]

No.	Position	Intensity	No.	Position	Intensity
3	2360.44	72.2141	4	1674.87	13.6063
5	1655.59	20.0351	6	1596.77	30.672
7	1522.52	12.5101	8	1497.45	28.4849
9	1405.85	31.7046	10	1359.57	48.2199
11	1321	37.377	12	1270.86	31.2026
13	1180.22	46.8069	14	1120.44	70.3419
15	1016.3	79.9368	16	962.305	69.4854
17	920.843	84.5846	18	902.523	82.422
19	851.418	53.9174	20	830.205	33.8264
21	777.172	60.9204	22	760.78	56.3145
23	693.284	60.0687	24	647.965	60.8317
25	628.68	63.0577	26	591.075	65.2002
27	548.649	72.8851	28	514.901	70.0164
29	480.188	80.709	30	448.369	79.2647

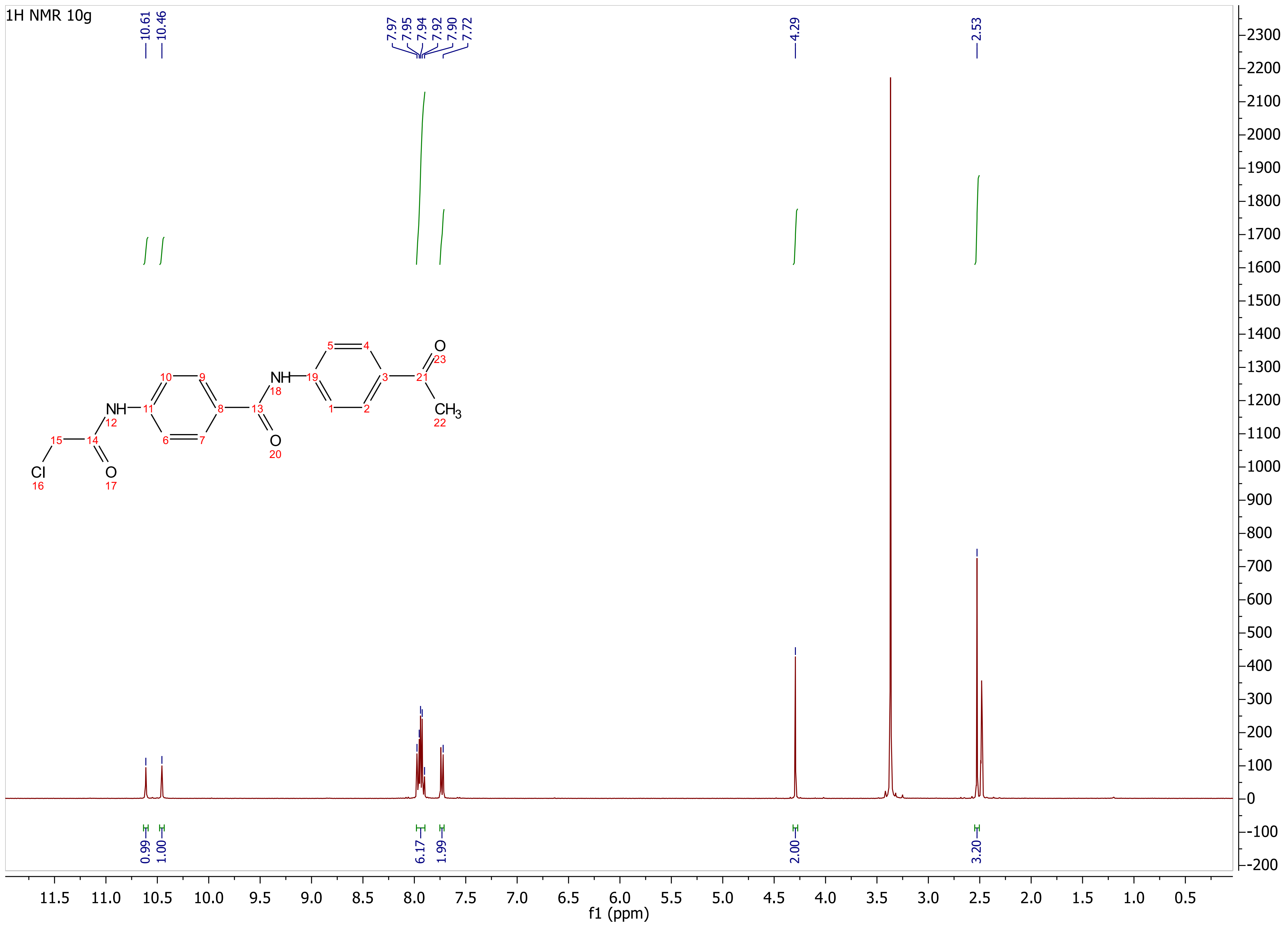

1H NMR 10g
10.61
10.46
7.97
7.95
7.94
7.92
7.90
7.72
4.29
2.53
0.99
1.00
6.17
1.99
2.00
3.20
f1 (ppm)

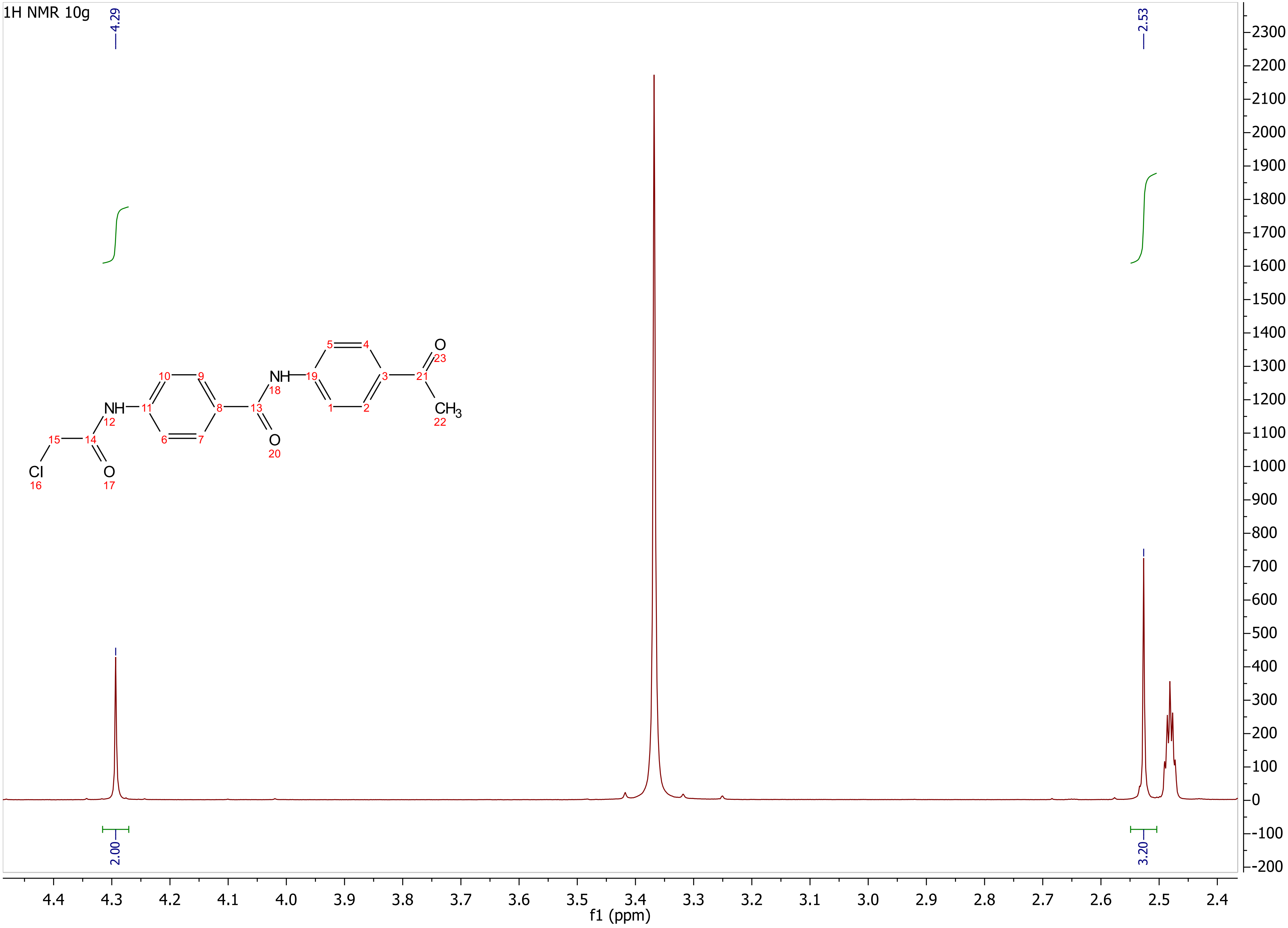

1H NMR 10g
4.29
2.53
2.00
3.20
NH
NH
CH3
Cl
O
O
O
f1 (ppm)
4.4
4.3
4.2
4.1
4.0
3.9
3.8
3.7
3.6
3.5
3.4
3.3
3.2
3.1
3.0
2.9
2.8
2.7
2.6
2.5
2.4

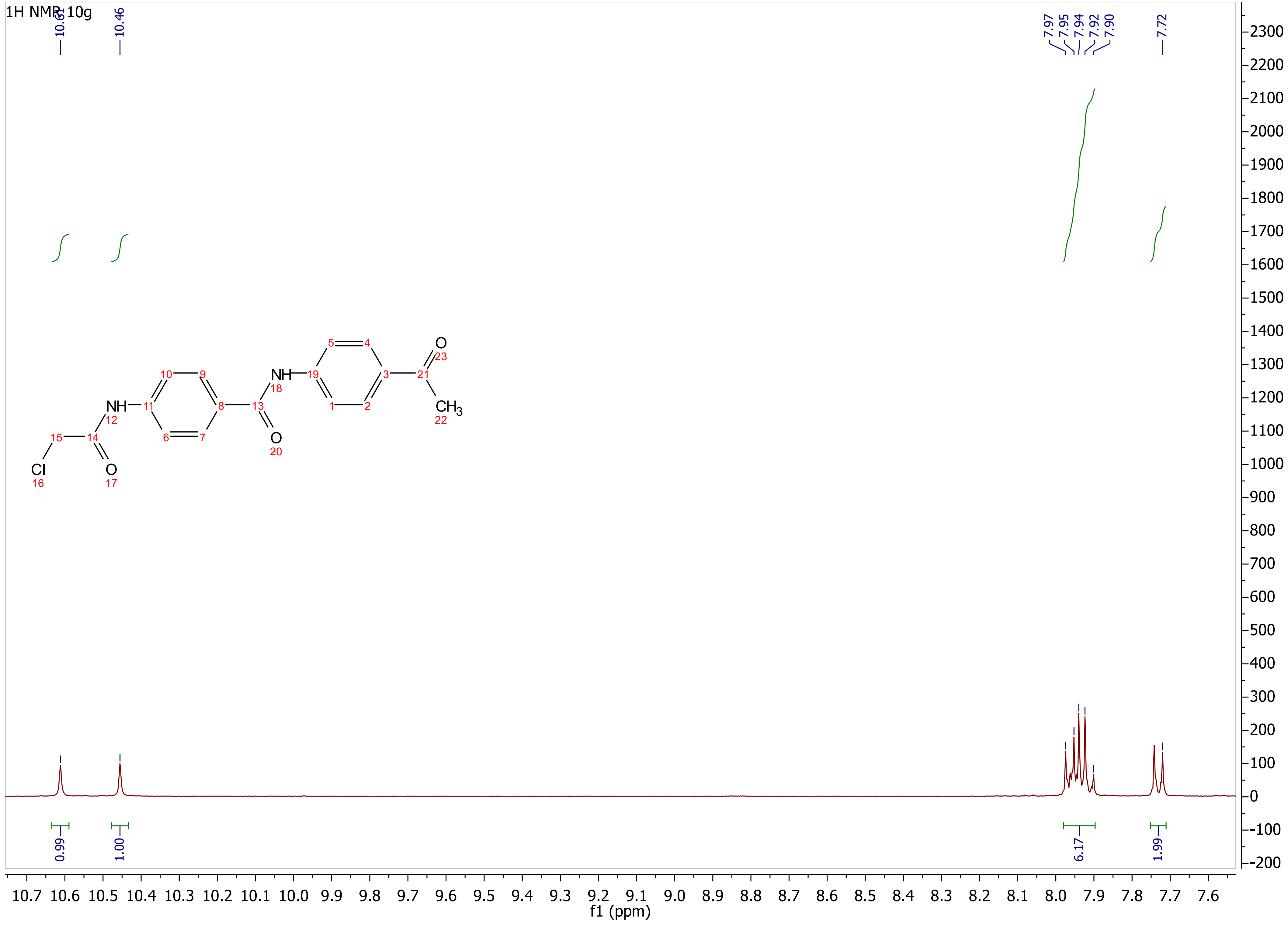
1H NMR 10g
10.61
10.46
7.97
7.95
7.94
7.92
7.90
7.72
0.99
1.00
6.17
1.99
NH
12
NH
18
O
17
O
20
O
23
Cl
16
CH3
22
f1 (ppm)

13C NMR 18g

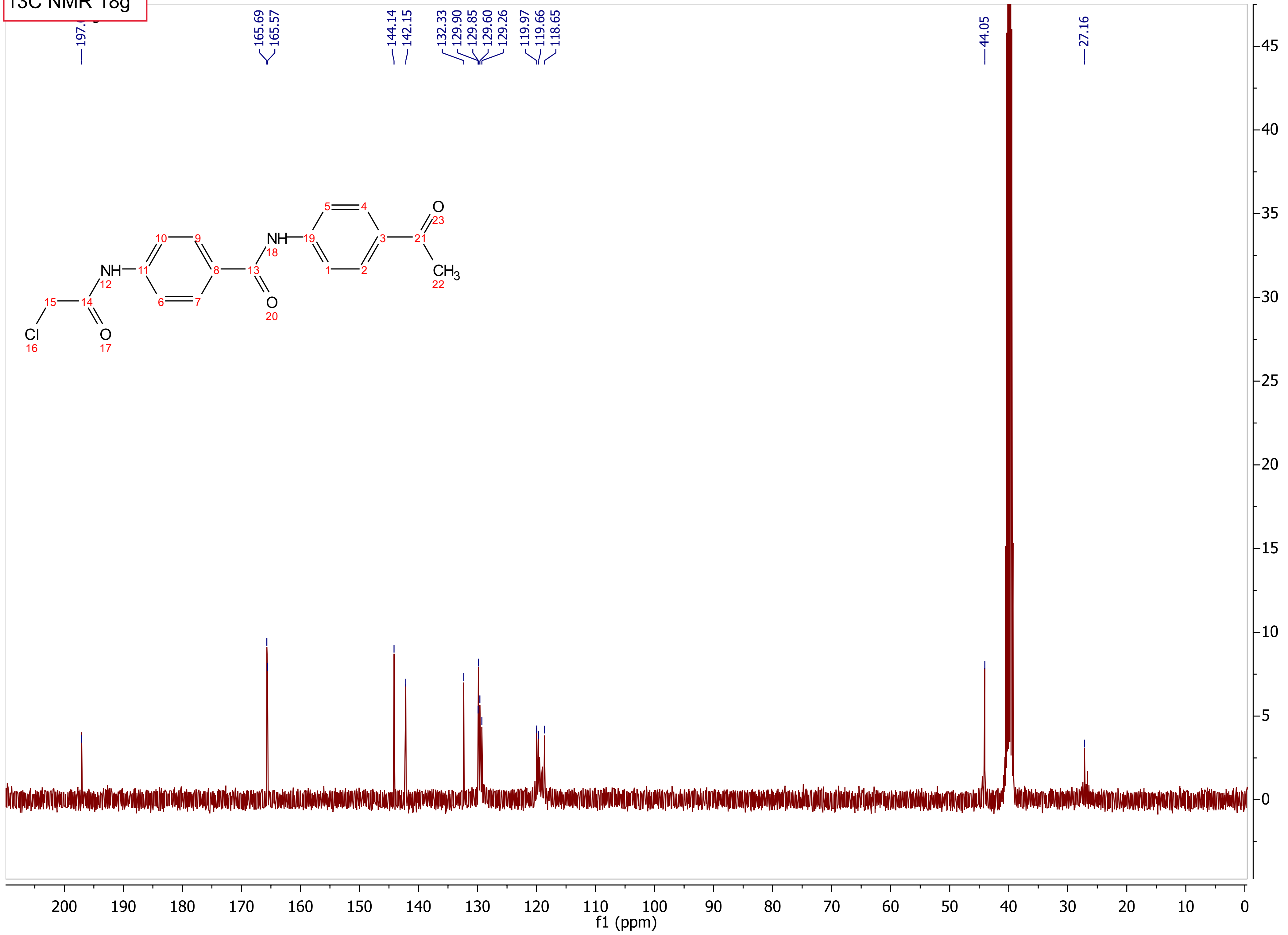
197.
165.69
165.57
144.14
142.15
132.33
129.90
129.85
129.60
129.26
119.97
119.66
118.65
44.05
27.16
Cl
NH
O
NH
O
O
CH3
f1 (ppm)
200 190 180 170 160 150 140 130 120 110 100 90 80 70 60 50 40 30 20 10 0
45 40 35 30 25 20 15 10 5 0

IR of compound 18h

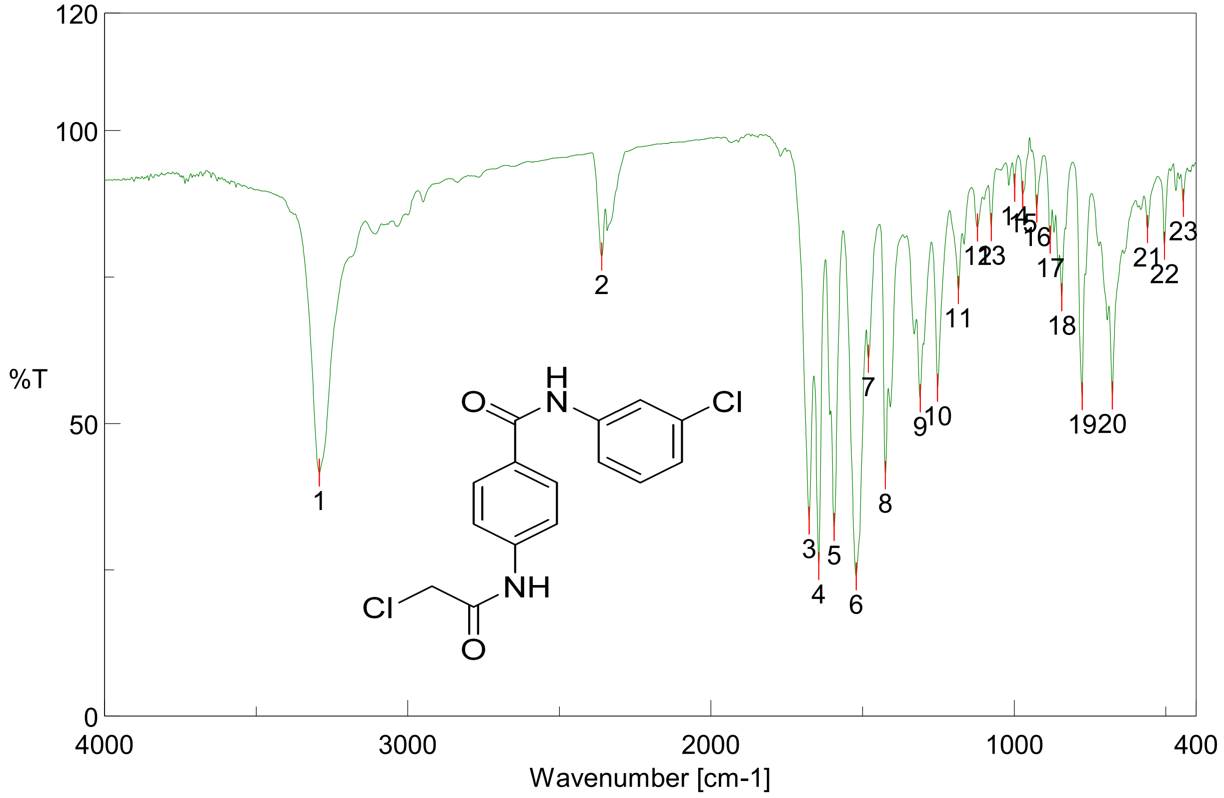

[Comments]

Sample name	A6
Comment	
User	
Division	
Company	KSU

[Detailed Information]

Creation date	9/15/2020 3:41 AM
Data array type	Linear data array
Horizontal axis	Wavenumber [cm-1]
Vertical axis	%T
Start	399.193 cm-1
End	4000.6 cm-1
Data interval	0.964233 cm-1
Data points	3736

[Measurement Information]

Model Name	FT/IR-6600typeA
Serial Number	A014661790
Measurement Date	9/15/2020 3:39 AM
Light Source	Standard
Detector	TGS
Accumulation	Auto (15)
Resolution	4 cm-1
Zero Filling	On
Apodization	Cosine
Gain	Auto (1)
Aperture	Auto (7.1 mm)
Scanning Speed	Auto (2 mm/sec)
Filter	Auto (10000 Hz)

[Result of Peak Picking]

No.	Position	Intensity	No.	Position	Intensity
1	3291.89	41.5463	2	2360.44	78.4557

[Result of Peak Picking]

No.	Position	Intensity	No.	Position	Intensity
3	1676.8	33.3698	4	1644.98	25.6188
5	1593.88	32.3065	6	1521.56	23.895
7	1481.06	61.0278	8	1425.14	41.1238
9	1310.39	54.263	10	1252.54	56.0434
11	1184.08	72.7278	12	1121.4	83.4221
13	1076.08	83.4536	14	998.946	90.1875
15	971.947	88.9998	16	925.664	86.6087
17	881.309	81.3381	18	843.704	71.5175
19	776.208	54.623	20	676.892	54.7116
21	560.22	83.1423	22	504.294	80.2718
23	442.583	87.6048			

^{1}H NMR of 18h

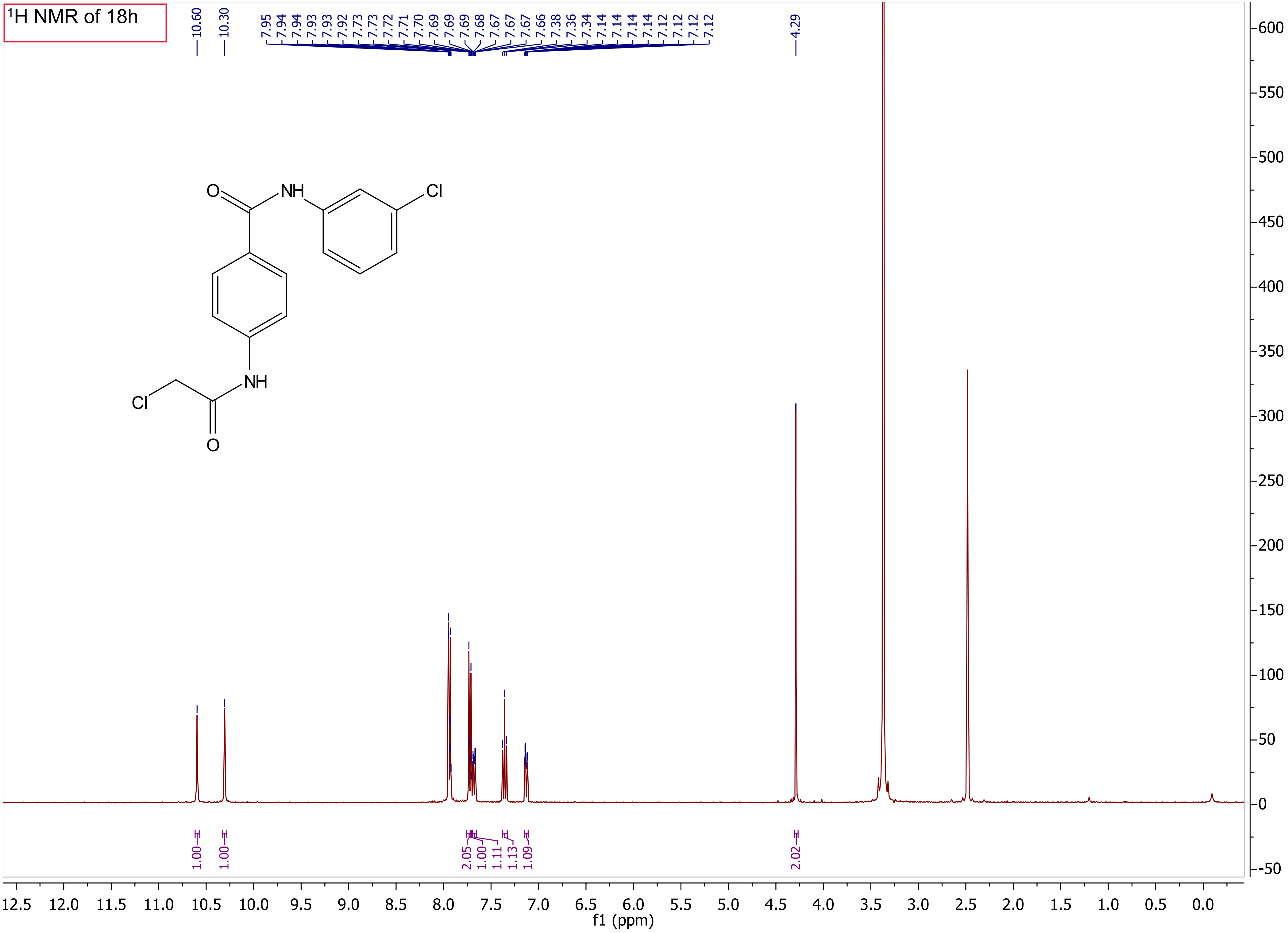

10.60
10.30
7.95
7.94
7.94
7.93
7.93
7.92
7.73
7.73
7.72
7.71
7.70
7.69
7.69
7.69
7.68
7.67
7.67
7.67
7.66
7.38
7.36
7.34
7.14
7.14
7.14
7.14
7.12
7.12
7.12
7.12
4.29
O
NH
Cl
NH
Cl
O
1.00
1.00
2.05
1.00
1.11
1.13
1.09
2.02
f1 (ppm)

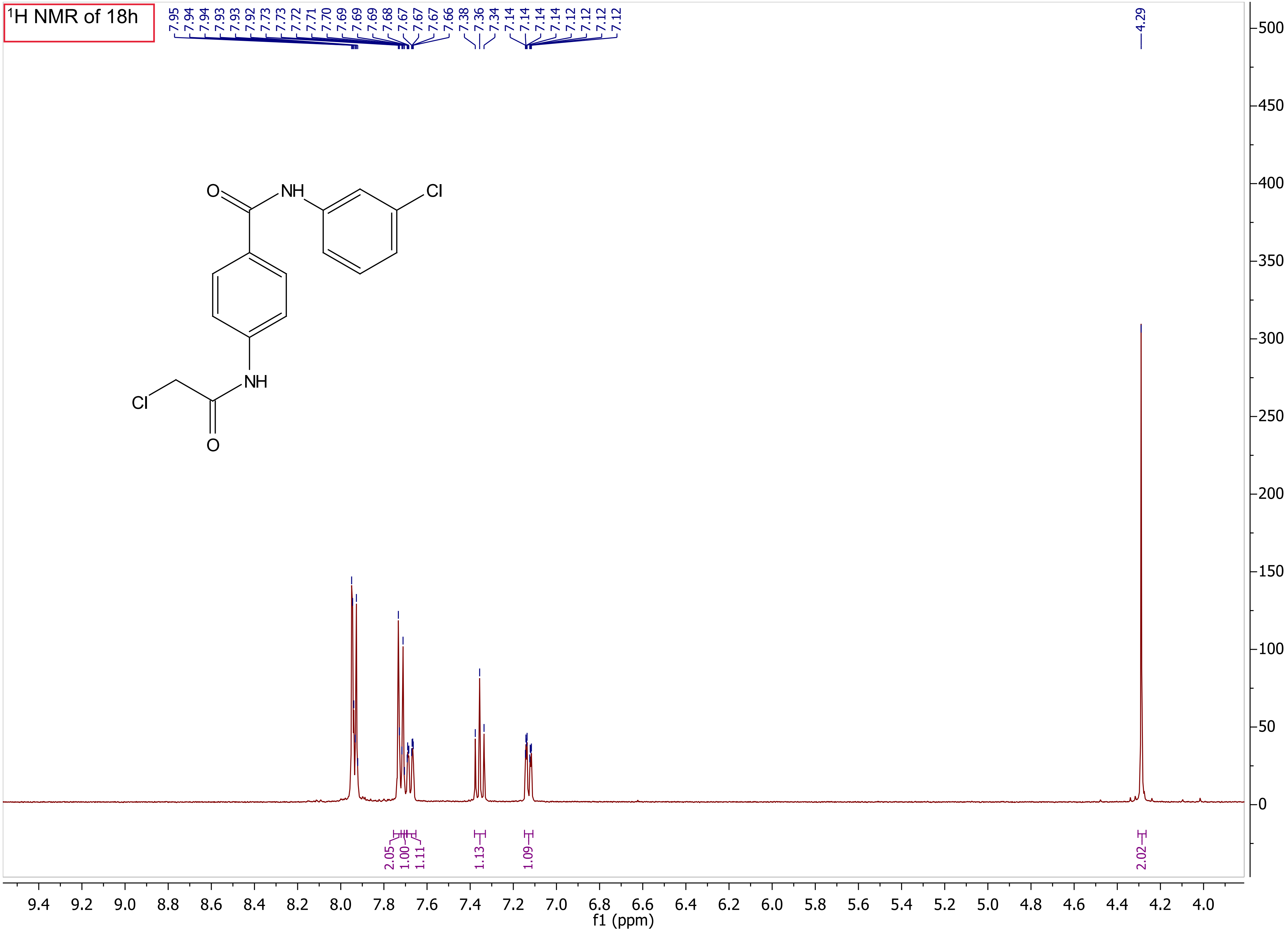

1H NMR of 18h
7.95 7.94 7.94 7.93 7.93 7.92 7.73 7.73 7.72 7.71 7.70 7.69 7.69 7.69 7.68 7.67 7.67 7.67 7.66 7.38 7.36 7.34 7.14 7.14 7.14 7.14 7.12 7.12 7.12 7.12
4.29
O
NH
Cl
NH
Cl
O
2.05
1.00
1.11
1.13
1.09
2.02
500
450
400
350
300
250
200
150
100
50
0
9.4 9.2 9.0 8.8 8.6 8.4 8.2 8.0 7.8 7.6 7.4 7.2 7.0 6.8 6.6 6.4 6.2 6.0 5.8 5.6 5.4 5.2 5.0 4.8 4.6 4.4 4.2 4.0
f1 (ppm)

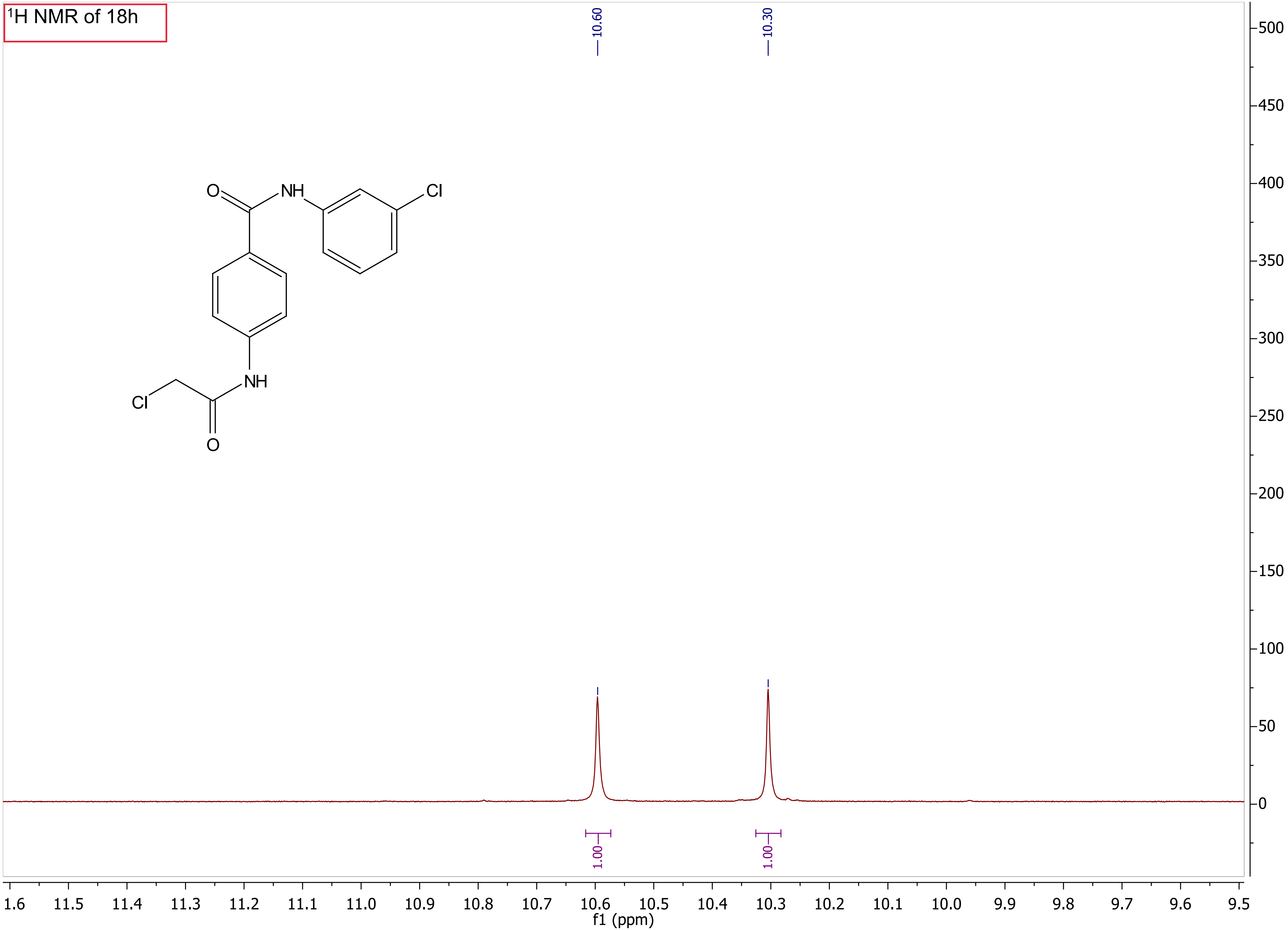

¹H NMR of 18h
10.60
10.30
O
NH
Cl
NH
Cl
O
1.00
1.00
1.6
11.5
11.4
11.3
11.2
11.1
11.0
10.9
10.8
10.7
10.6
10.5
10.4
10.3
10.2
10.1
10.0
9.9
9.8
9.7
9.6
9.5
f1 (ppm)
500
450
400
350
300
250
200
150
100
50
0

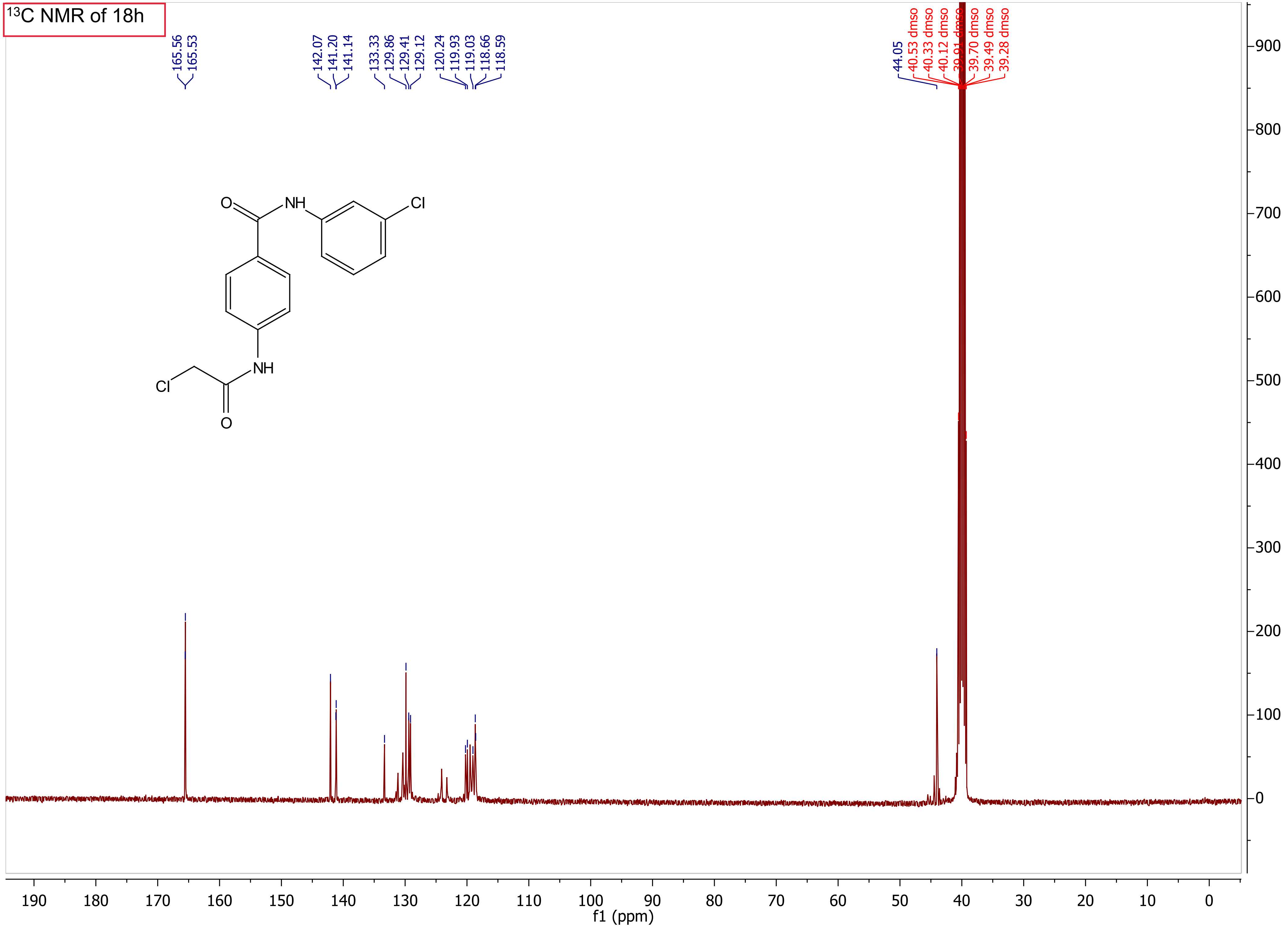

^{13}C NMR of 18h
165.56
165.53
142.07
141.20
141.14
133.33
129.86
129.41
129.12
120.24
119.93
119.03
118.66
118.59
44.05
40.53 dmso
40.33 dmso
40.12 dmso
39.91 dmso
39.70 dmso
39.49 dmso
39.28 dmso
O
NH
Cl
NH
Cl
O
f1 (ppm)

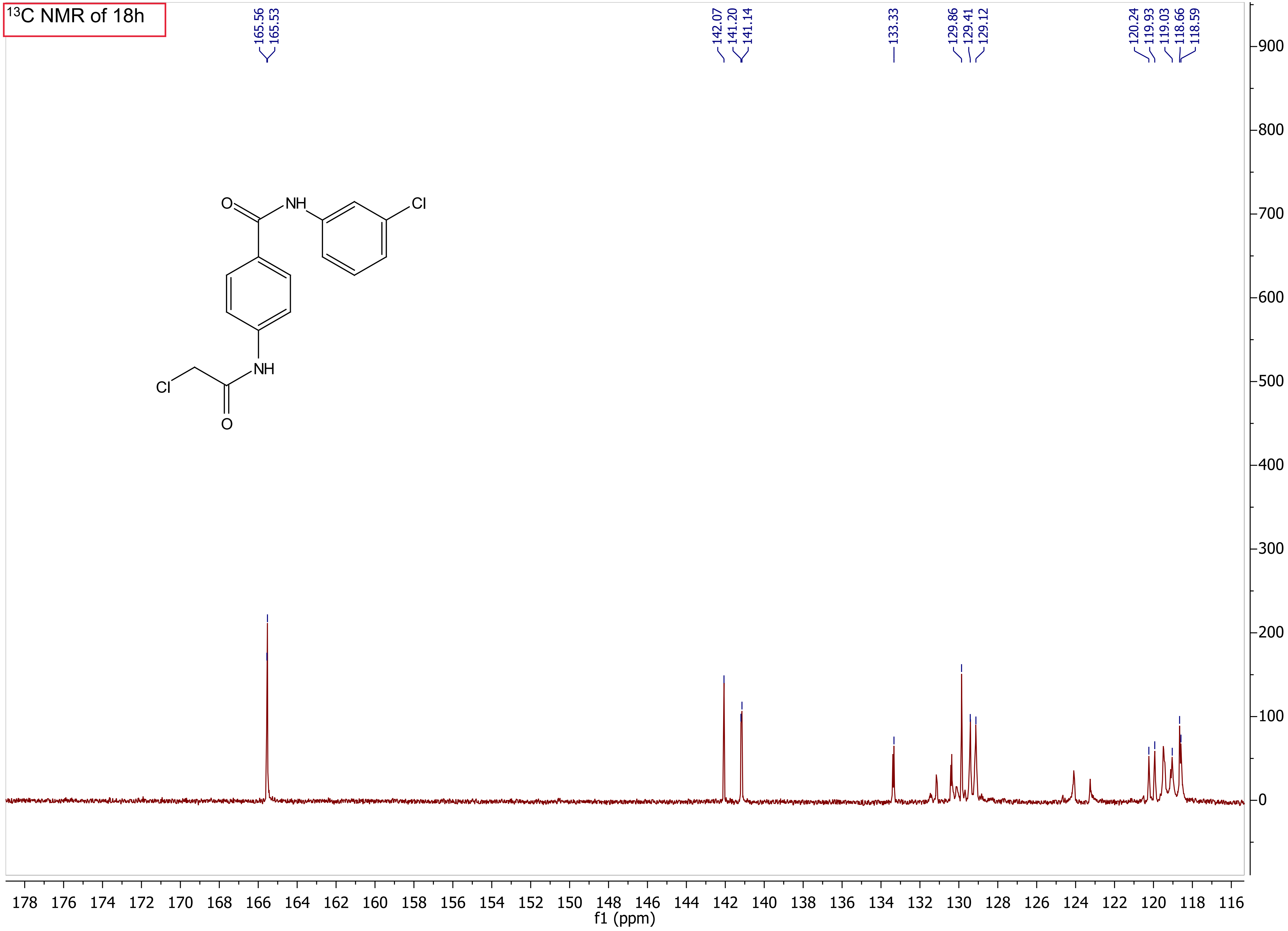

13C NMR of 18h
165.56
165.53
142.07
141.20
141.14
133.33
129.86
129.41
129.12
120.24
119.93
119.03
118.66
118.59
O
NH
Cl
NH
Cl
O
f1 (ppm)

IR of compound 18i

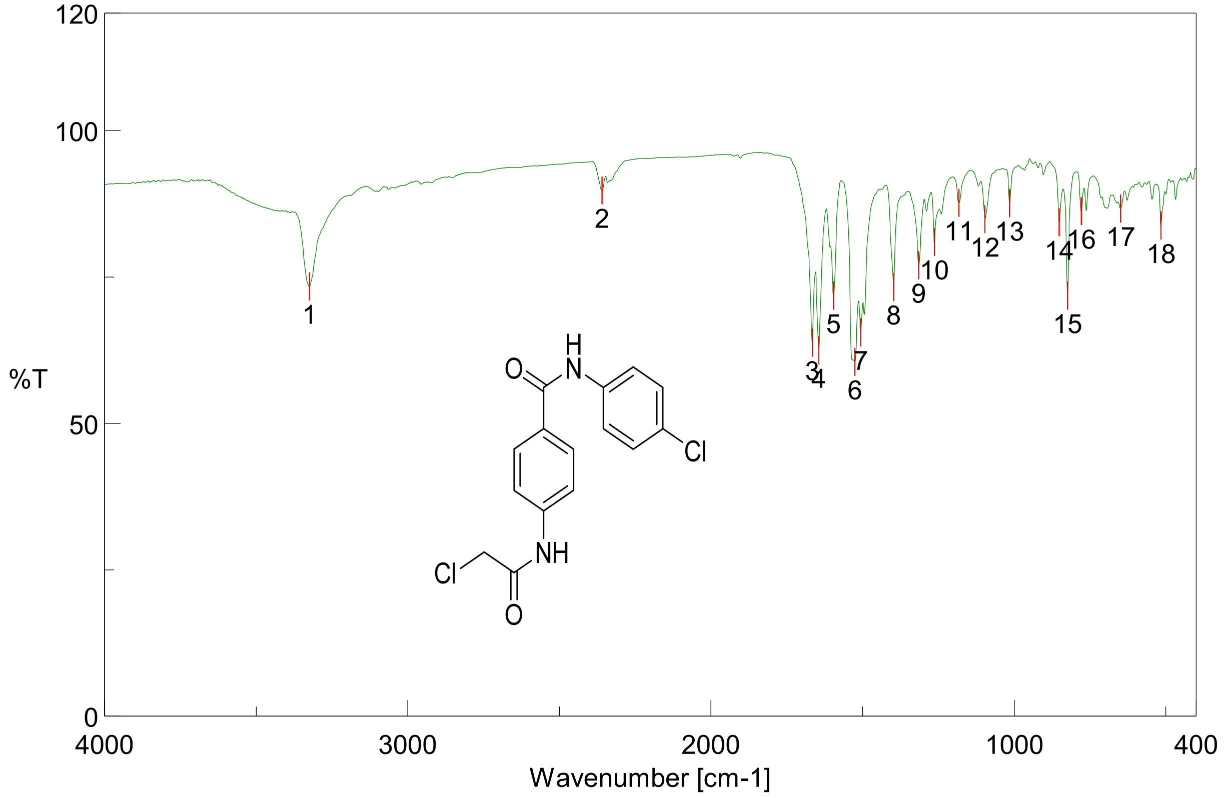

[Comments]

Sample name	A3
Comment	
User	
Division	
Company	KSU

[Detailed Information]

Creation date	9/15/2020 2:41 AM
Data array type	Linear data array
Horizontal axis	Wavenumber [cm-1]
Vertical axis	%T
Start	399.193 cm-1
End	4000.6 cm-1
Data interval	0.964233 cm-1
Data points	3736

[Measurement Information]

Model Name	FT/IR-6600typeA
Serial Number	A014661790
Measurement Date	9/15/2020 2:38 AM
Light Source	Standard
Detector	TGS
Accumulation	Auto (15)
Resolution	4 cm-1
Zero Filling	On
Apodization	Cosine
Gain	Auto (1)
Aperture	Auto (7.1 mm)
Scanning Speed	Auto (2 mm/sec)
Filter	Auto (10000 Hz)

[Result of Peak Picking]

No.	Position	Intensity	No.	Position	Intensity
1	3324.68	73.3275	2	2359.48	89.7429

[Result of Peak Picking]

No.	Position	Intensity	No.	Position	Intensity
3	1666.2	63.7518	4	1644.98	62.4412
5	1595.81	71.7871	6	1525.42	60.4584
7	1506.13	65.4744	8	1397.17	73.2605
9	1315.21	76.9601	10	1263.15	80.9292
11	1182.15	87.6272	12	1096.33	84.8233
13	1015.34	87.53	14	851.418	84.2545
15	824.42	71.757	16	779.101	86.1967
17	648.929	86.619	18	515.865	83.751

^{1}H NMR of 18i

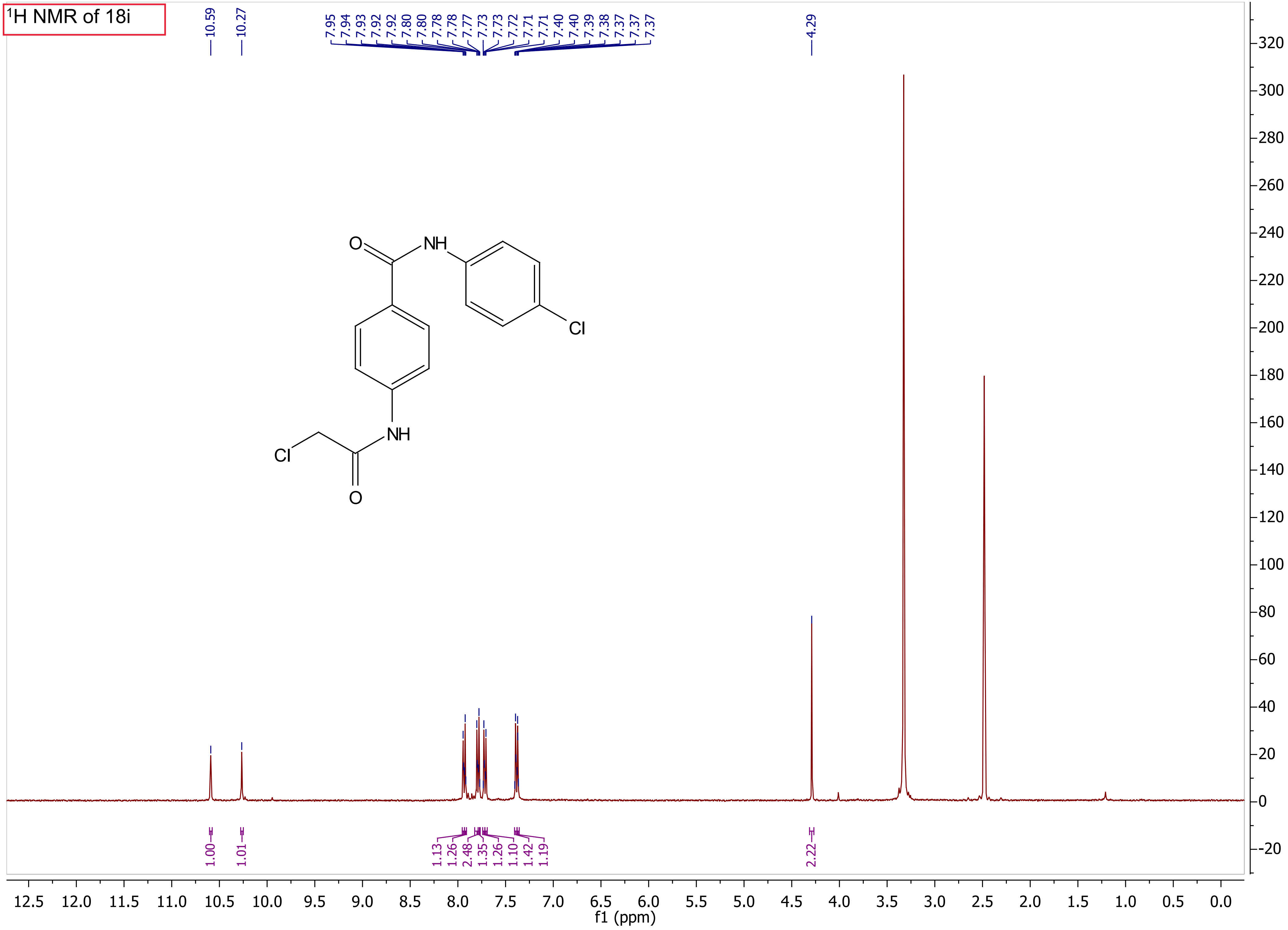

10.59
10.27
7.95
7.94
7.93
7.92
7.92
7.80
7.80
7.78
7.78
7.77
7.73
7.73
7.72
7.71
7.71
7.40
7.40
7.39
7.38
7.37
7.37
7.37
4.29
O
NH
Cl
NH
Cl
O
1.00
1.01
1.13
1.26
2.48
1.35
1.26
1.10
1.42
1.19
2.22
f1 (ppm)

^{1}H NMR of 18i

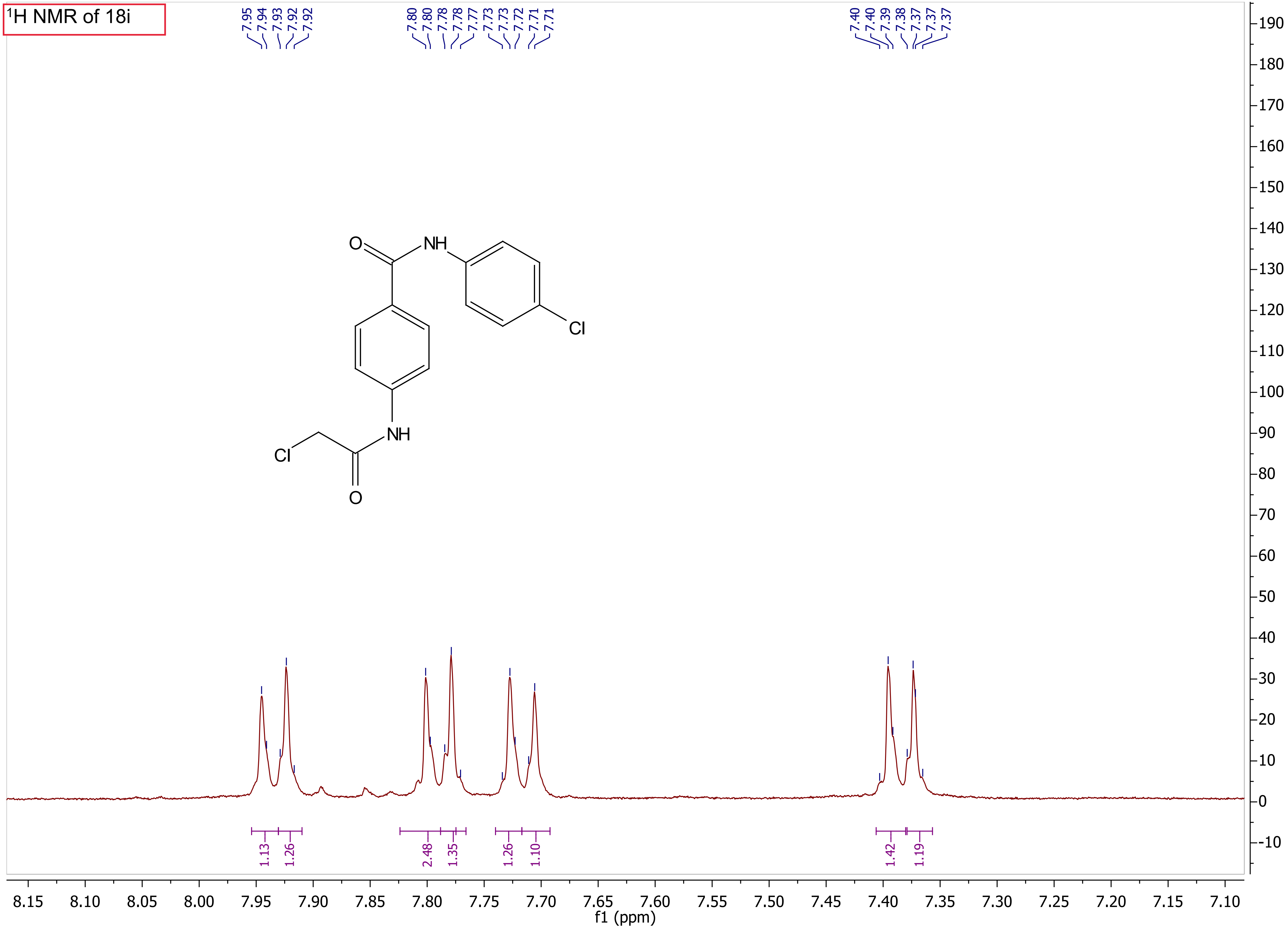

7.95
7.94
7.93
7.92
7.92
7.80
7.80
7.78
7.78
7.77
7.73
7.73
7.72
7.71
7.71
7.40
7.40
7.39
7.38
7.37
7.37
7.37
O
NH
Cl
NH
Cl
O
1.13
1.26
2.48
1.35
1.26
1.10
1.42
1.19
f1 (ppm)

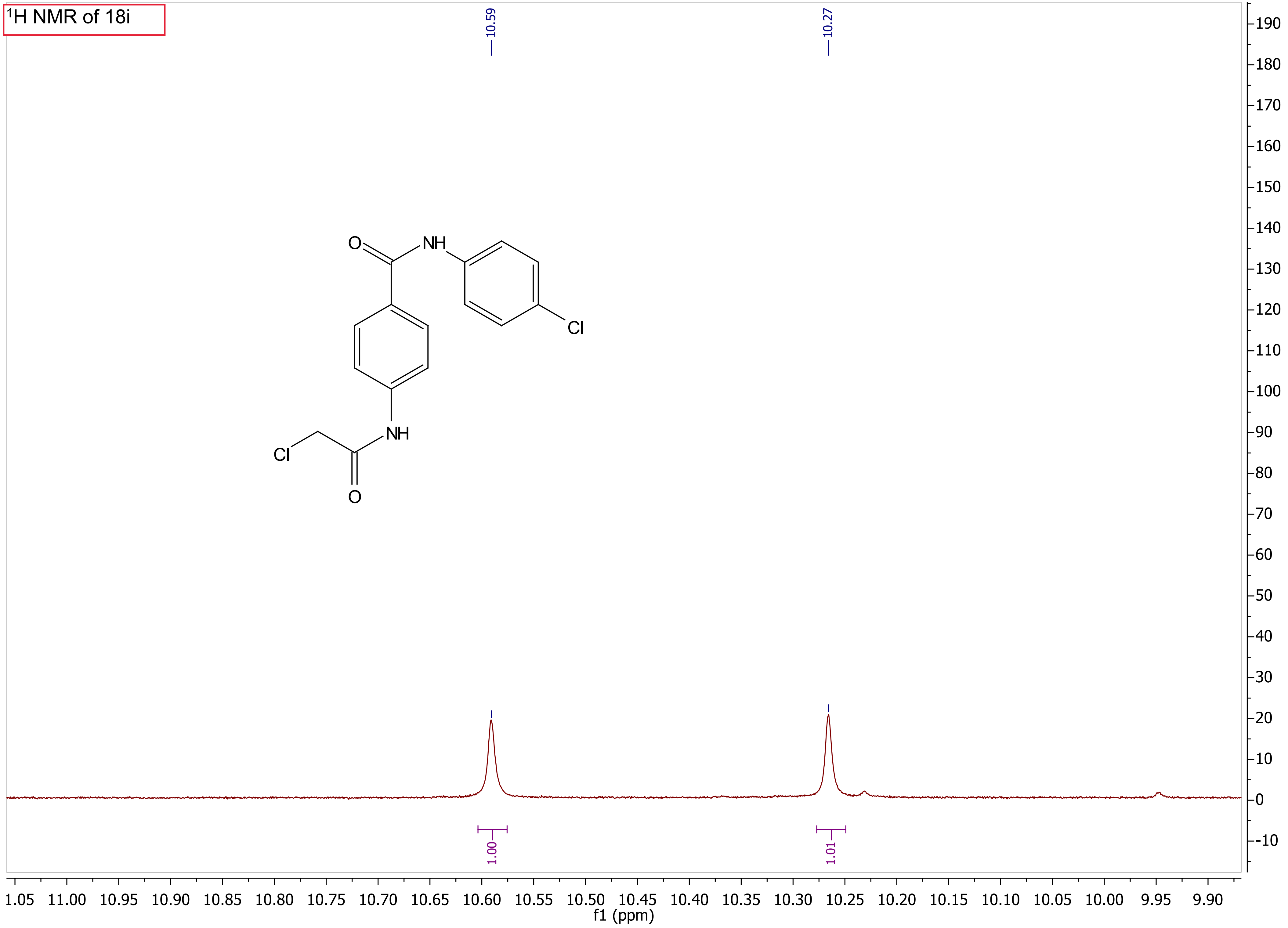

1H NMR of 18i
10.59
10.27
O
NH
Cl
NH
Cl
O
1.00
1.01
f1 (ppm)

^{13}C NMR of 18i

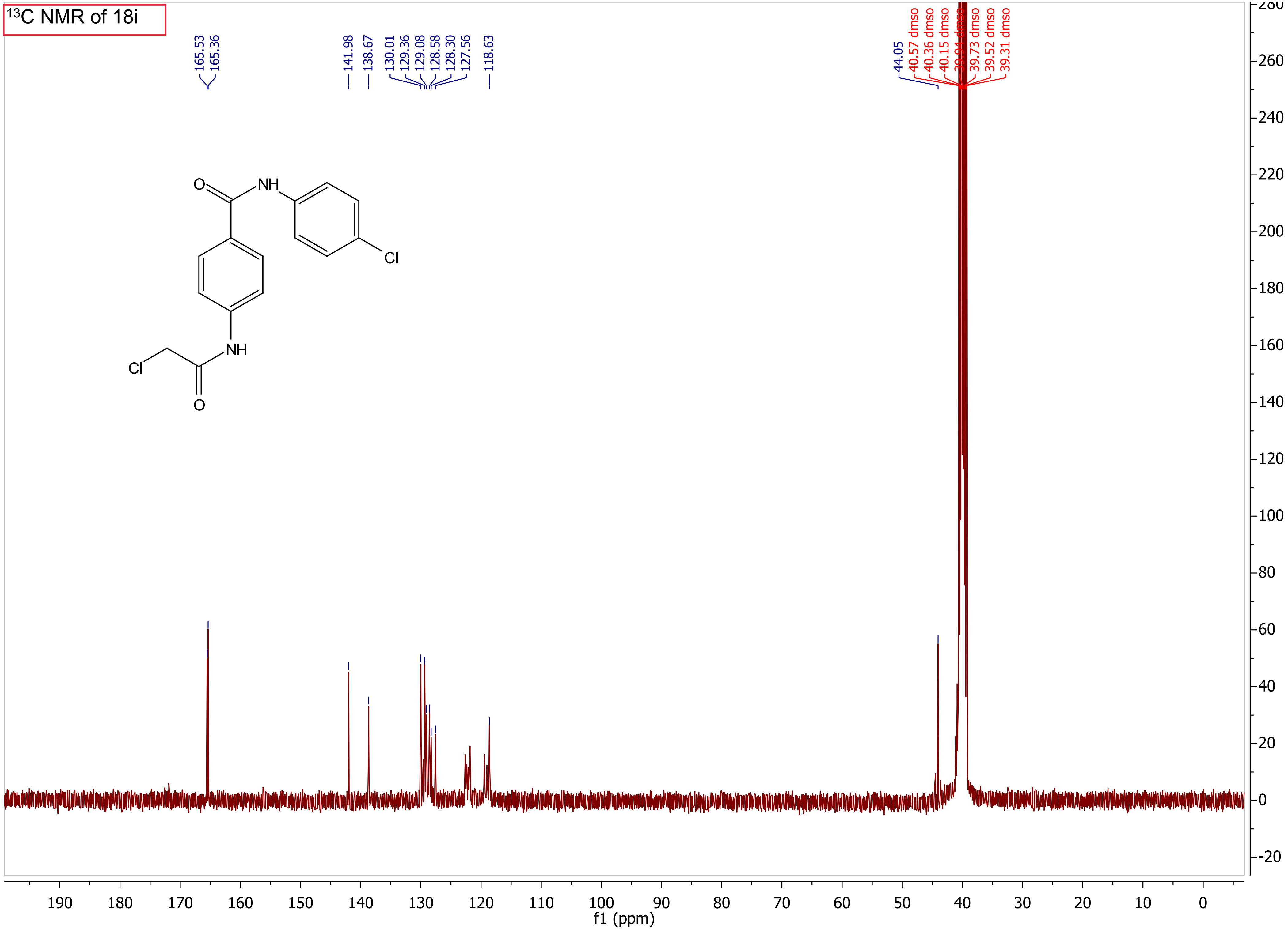

^{13}C NMR of 18i

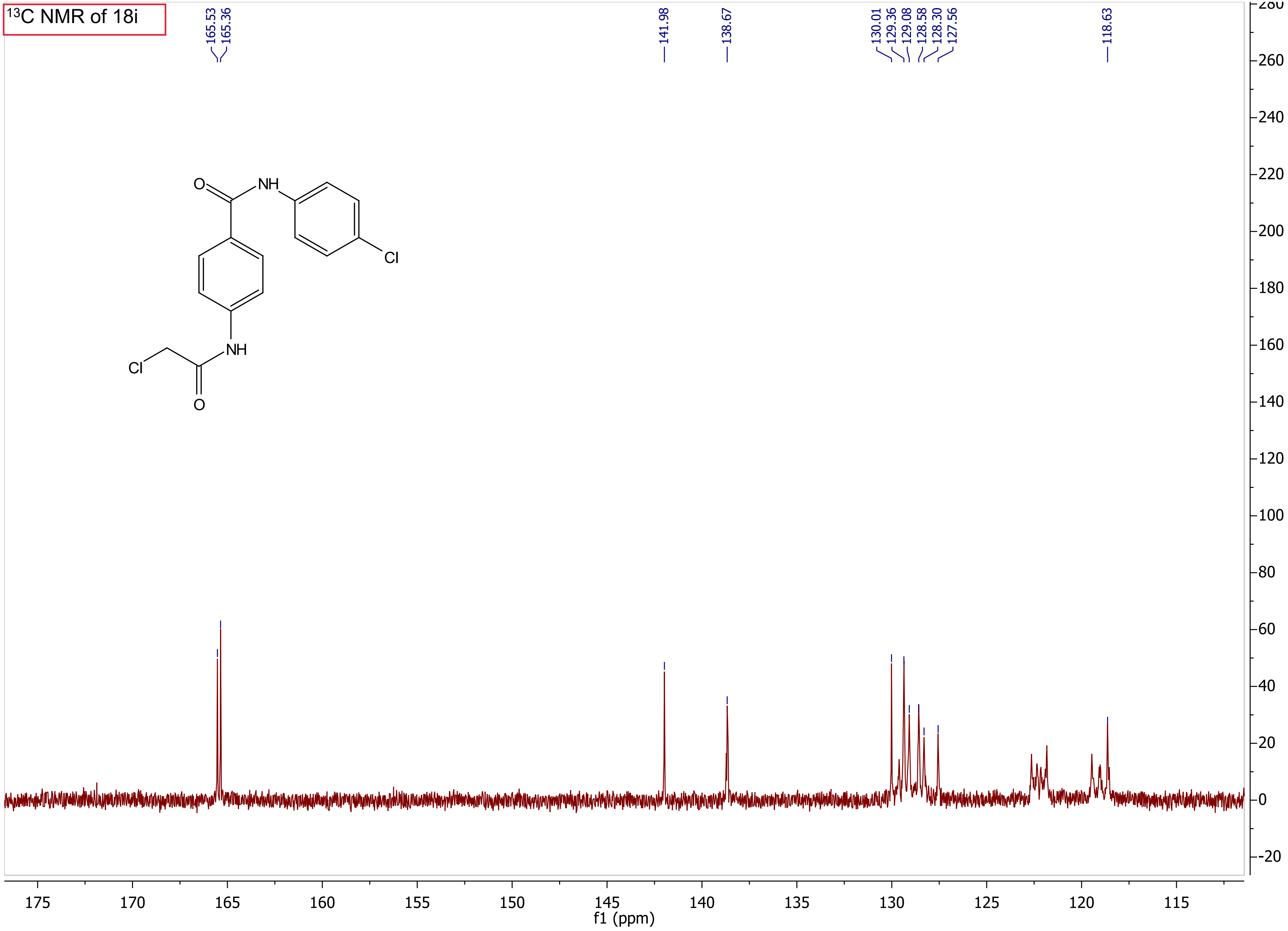

IR of compound 18j

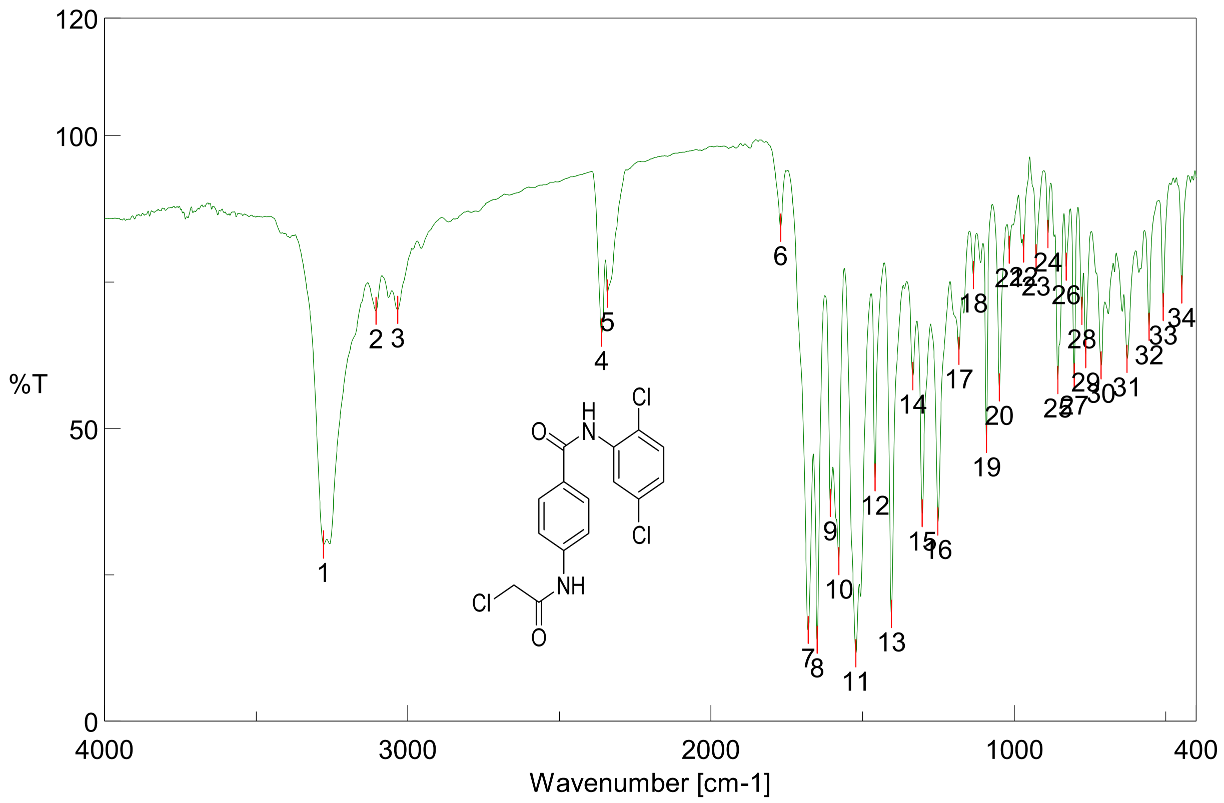

[Comments]

Sample name	A15
Comment	
User	
Division	
Company	KSU

[Detailed Information]

Creation date	9/15/2020 4:15 AM
Data array type	Linear data array
Horizontal axis	Wavenumber [cm-1]
Vertical axis	%T
Start	399.193 cm-1
End	4000.6 cm-1
Data interval	0.964233 cm-1
Data points	3736

[Measurement Information]

Model Name	FT/IR-6600typeA
Serial Number	A014661790
Measurement Date	9/15/2020 4:13 AM
Light Source	Standard
Detector	TGS
Accumulation	Auto (17)
Resolution	4 cm-1
Zero Filling	On
Apodization	Cosine
Gain	Auto (2)
Aperture	Auto (7.1 mm)
Scanning Speed	Auto (2 mm/sec)
Filter	Auto (10000 Hz)

[Result of Peak Picking]

No.	Position	Intensity	No.	Position	Intensity
1	3277.43	30.1286	2	3104.83	70.0312

[Result of Peak Picking]

No.	Position	Intensity	No.	Position	Intensity
3	3033.48	70.1937	4	2360.44	66.2868
5	2341.16	72.9835	6	1770.33	84.2388
7	1679.69	15.5628	8	1649.8	13.8904
9	1606.41	37.2757	10	1578.45	27.3199
11	1522.52	11.5882	12	1458.89	41.6317
13	1404.89	18.3215	14	1334.5	58.8806
15	1303.64	35.4924	16	1251.58	34.0586
17	1182.15	63.1864	18	1134.9	76.1617
19	1091.51	48.1523	20	1049.09	56.946
21	1016.3	80.4343	22	969.055	80.666
23	927.593	79.0402	24	889.023	83.1287
25	856.239	58.2183	26	828.277	77.5758
27	802.242	58.7372	28	777.172	69.9991
29	764.637	62.6708	30	713.533	60.7541
31	627.716	61.8313	32	555.398	67.2966
33	509.115	70.6571	34	447.404	73.6997

^{1}H NMR of 18j

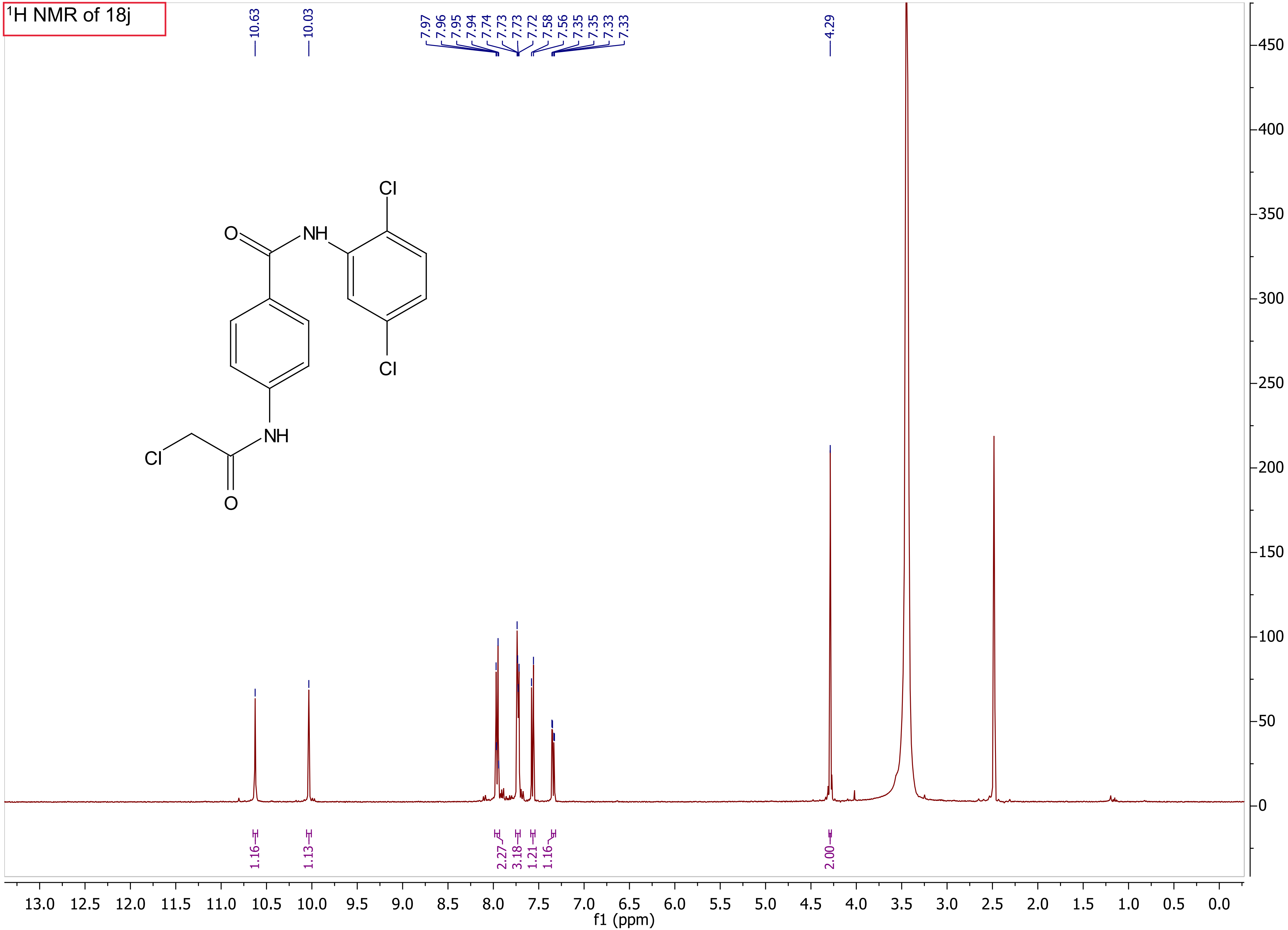

10.63
10.03
7.97
7.96
7.95
7.94
7.74
7.73
7.73
7.72
7.58
7.56
7.35
7.35
7.33
7.33
4.29
Cl
O
NH
Cl
NH
Cl
O
1.16
1.13
2.27
3.18
1.21
1.16
2.00
13.0 12.5 12.0 11.5 11.0 10.5 10.0 9.5 9.0 8.5 8.0 7.5 7.0 6.5 6.0 5.5 5.0 4.5 4.0 3.5 3.0 2.5 2.0 1.5 1.0 0.5 0.0
f1 (ppm)
450
400
350
300
250
200
150
100
50
0

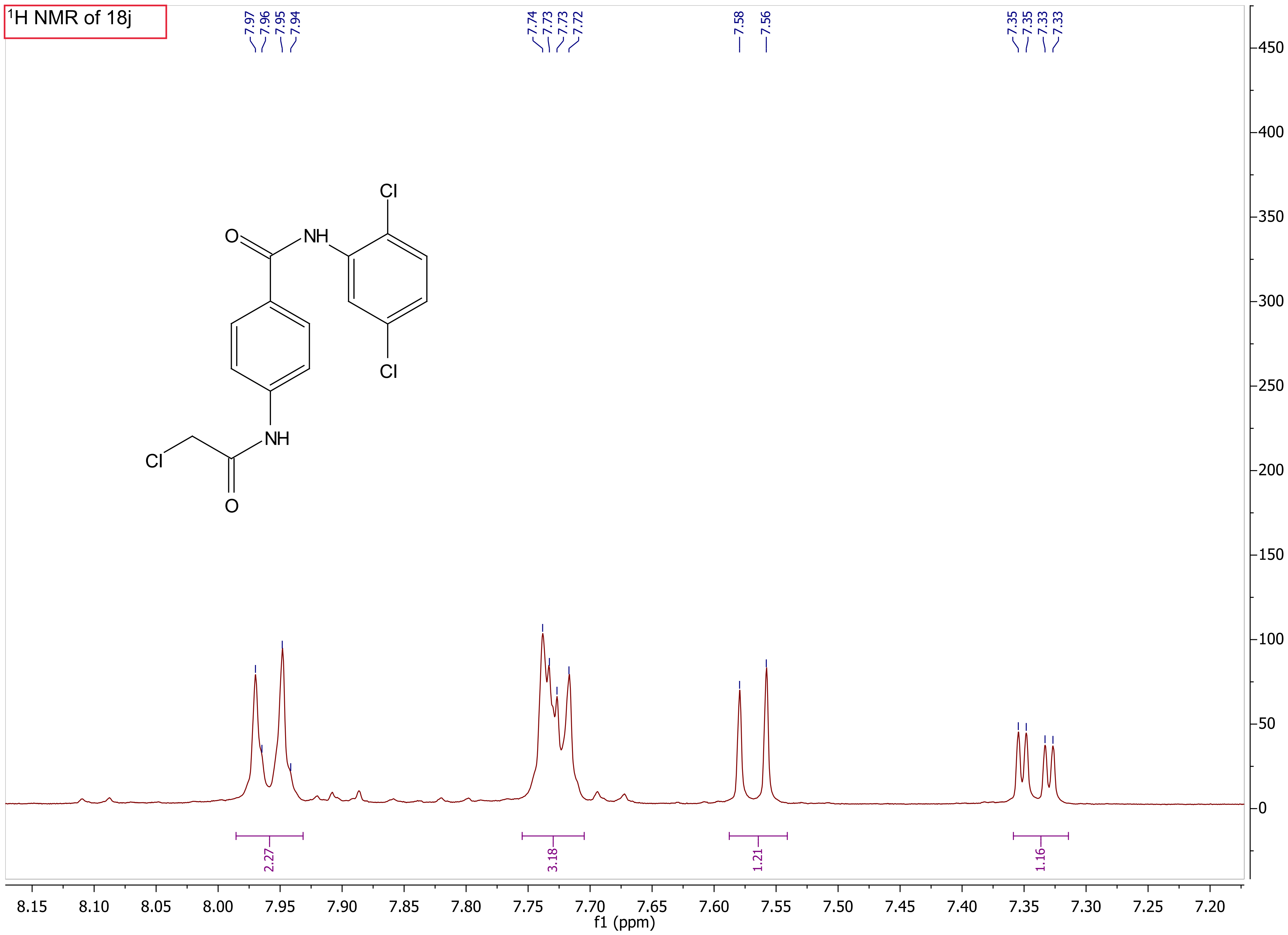

^{1}H NMR of 18j
7.97
7.96
7.95
7.94
7.74
7.73
7.73
7.72
7.58
7.56
7.35
7.35
7.33
7.33
Cl
O
NH
Cl
NH
Cl
O
2.27
3.18
1.21
1.16
f1 (ppm)

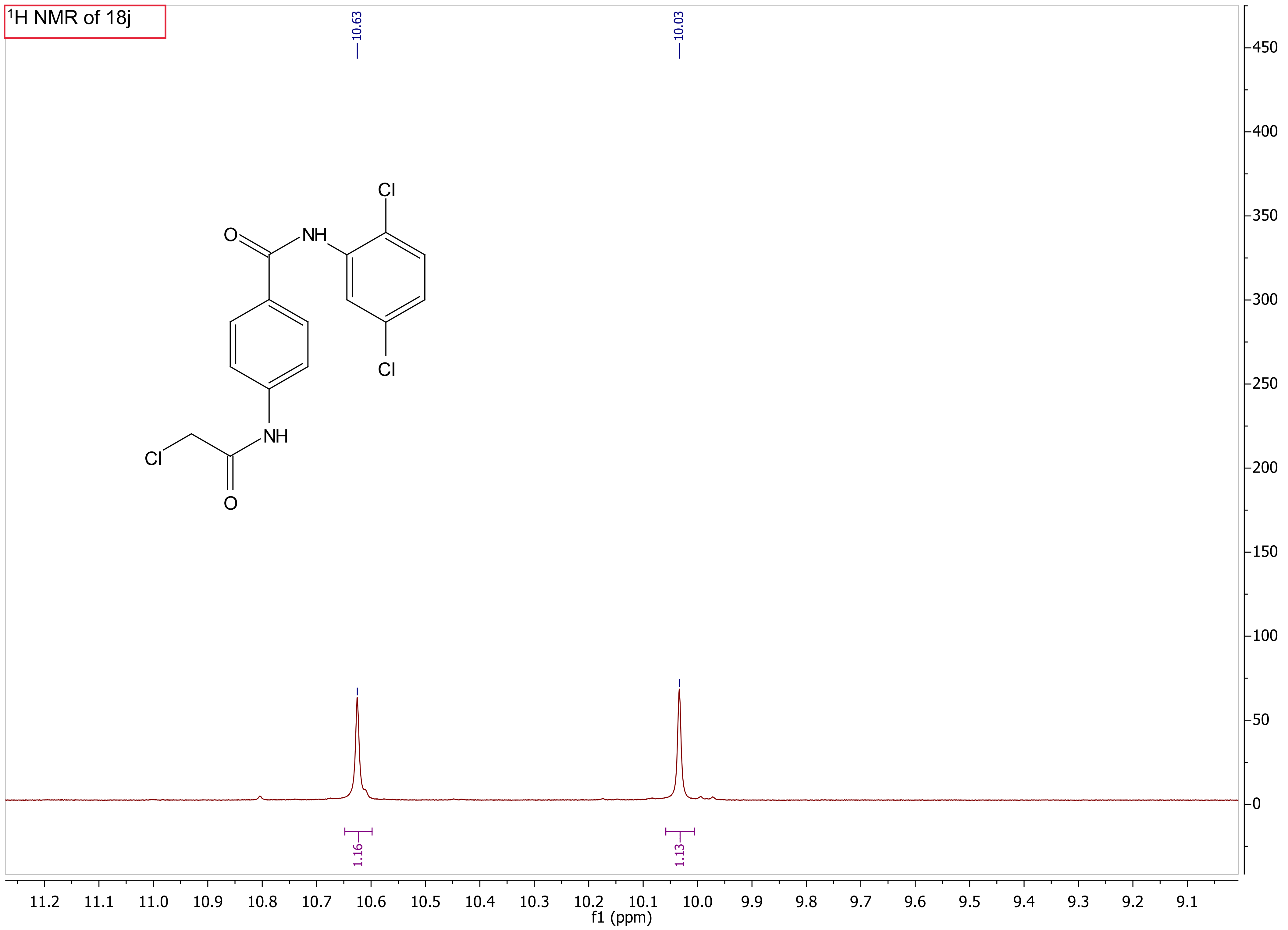
1H NMR of 18j
10.63
10.03
Cl
O
NH
Cl
NH
Cl
O
1.16
1.13
450
400
350
300
250
200
150
100
50
0
11.2
11.1
11.0
10.9
10.8
10.7
10.6
10.5
10.4
10.3
10.2
10.1
10.0
9.9
9.8
9.7
9.6
9.5
9.4
9.3
9.2
9.1
f1 (ppm)

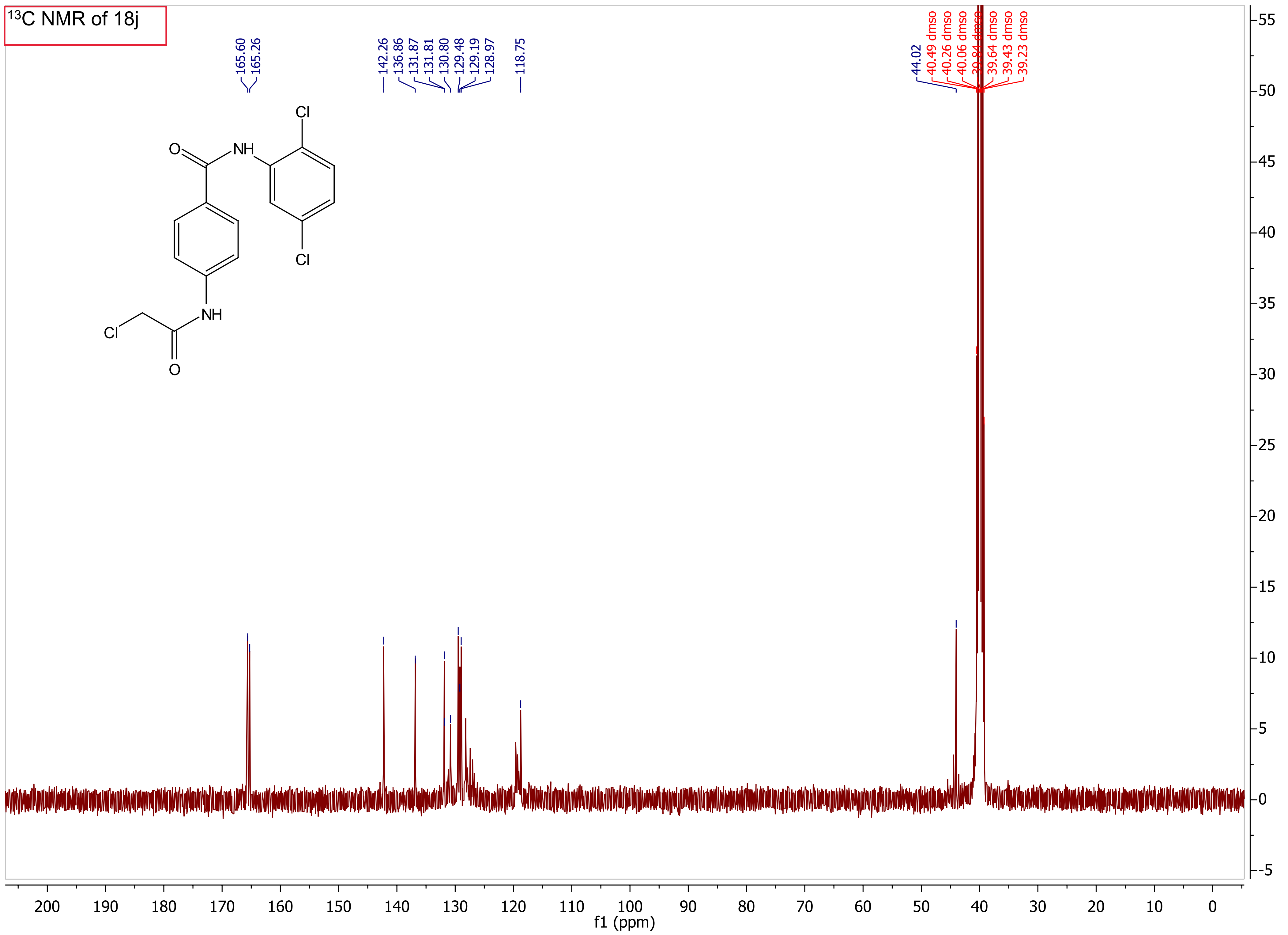

13C NMR of 18j
165.60
165.26
142.26
136.86
131.87
131.81
130.80
129.48
129.19
128.97
118.75
44.02
40.49 dmso
40.26 dmso
40.06 dmso
39.84 dmso
39.64 dmso
39.43 dmso
39.23 dmso
Cl
O
NH
Cl
NH
Cl
O
f1 (ppm)

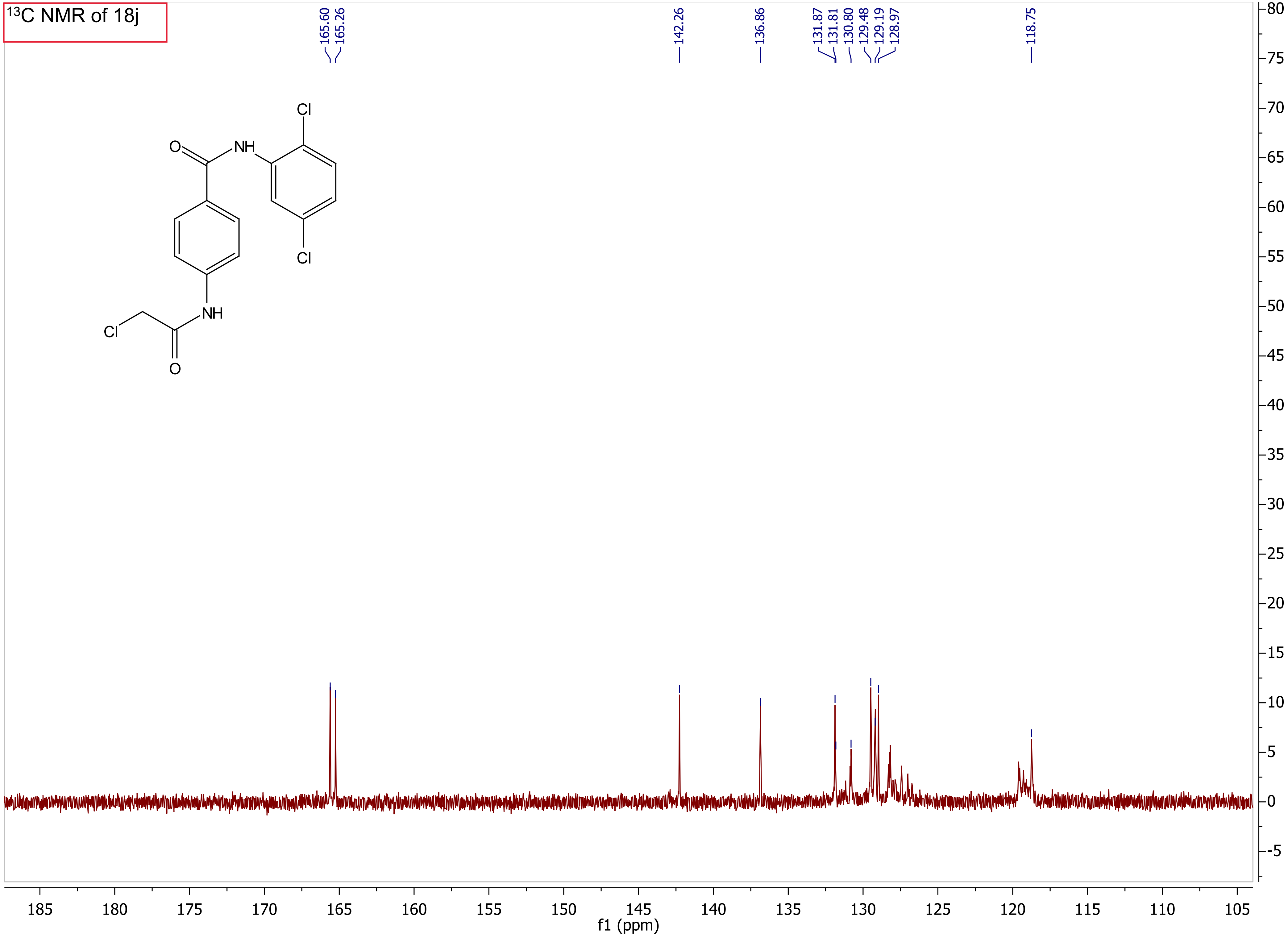

^{13}C NMR of 18j
165.60
165.26
142.26
136.86
131.87
131.81
130.80
129.48
129.19
128.97
118.75
Cl
O
NH
Cl
NH
Cl
O
f1 (ppm)

IR of compound 18k

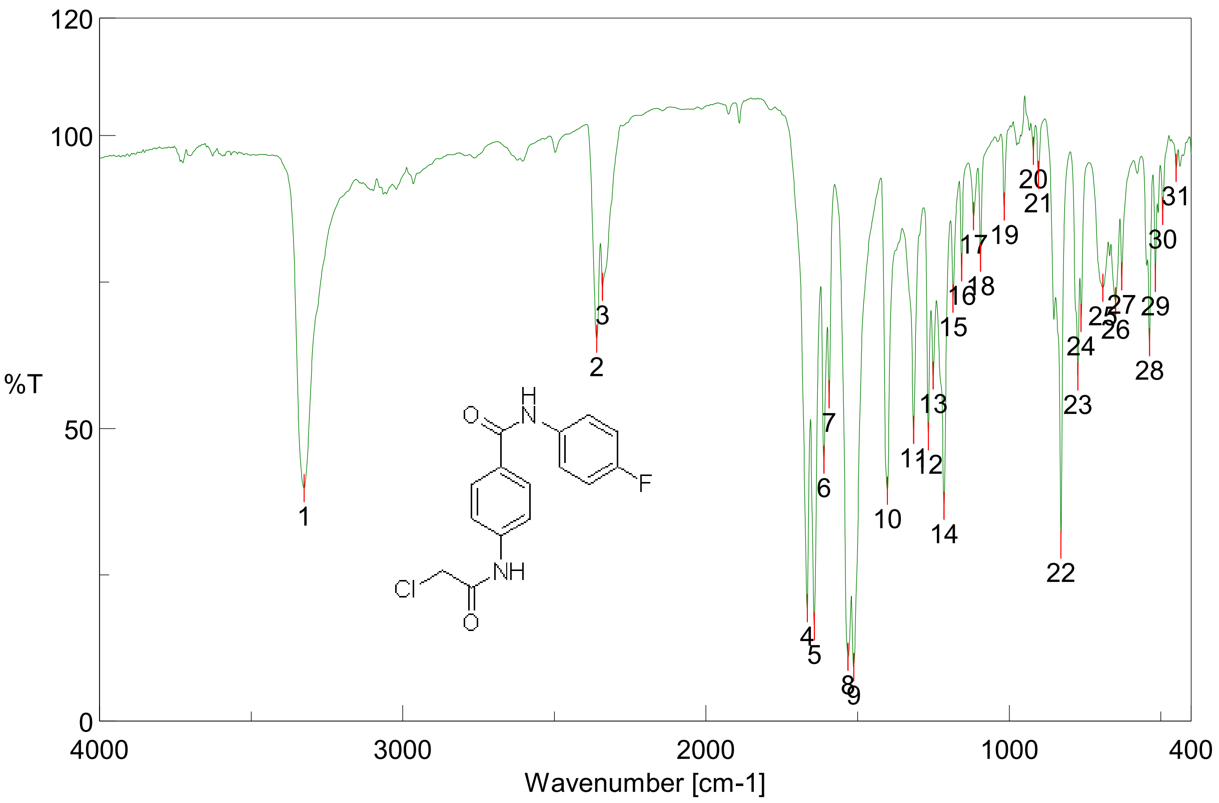

[Comments]

Sample name	A27
Comment	
User	
Division	
Company	KSU

[Detailed Information]

Creation date	9/15/2020 4:58 AM
Data array type	Linear data array
Horizontal axis	Wavenumber [cm-1]
Vertical axis	%T
Start	399.193 cm-1
End	4000.6 cm-1
Data interval	0.964233 cm-1
Data points	3736

[Measurement Information]

Model Name	FT/IR-6600typeA
Serial Number	A014661790
Measurement Date	9/15/2020 4:57 AM
Light Source	Standard
Detector	TGS
Accumulation	Auto (15)
Resolution	4 cm-1
Zero Filling	On
Apodization	Cosine
Gain	Auto (1)
Aperture	Auto (7.1 mm)
Scanning Speed	Auto (2 mm/sec)
Filter	Auto (10000 Hz)

[Result of Peak Picking]

No.	Position	Intensity	No.	Position	Intensity
1	3325.64	39.7597	2	2360.44	65.2491

[Result of Peak Picking]

No.	Position	Intensity	No.	Position	Intensity
3	2341.16	74.1223	4	1666.2	19.2991
5	1643.05	16.1865	6	1611.23	44.6329
7	1593.88	55.7822	8	1531.2	10.959
9	1512.88	9.25398	10	1402	39.3057
11	1315.21	49.7137	12	1267	48.6261
13	1250.61	58.9645	14	1214.93	36.7777
15	1185.04	72.0892	16	1157.08	77.4682
17	1117.55	86.1791	18	1094.4	79.0945
19	1016.3	87.8543	20	920.843	97.3507
21	903.487	93.2202	22	829.241	30.1686
23	773.315	58.8499	24	762.709	68.846
25	691.355	73.9718	26	648.929	71.6788
27	628.68	75.9033	28	537.078	64.6761
29	517.793	75.6658	30	493.688	87.0821
31	449.333	94.4481			

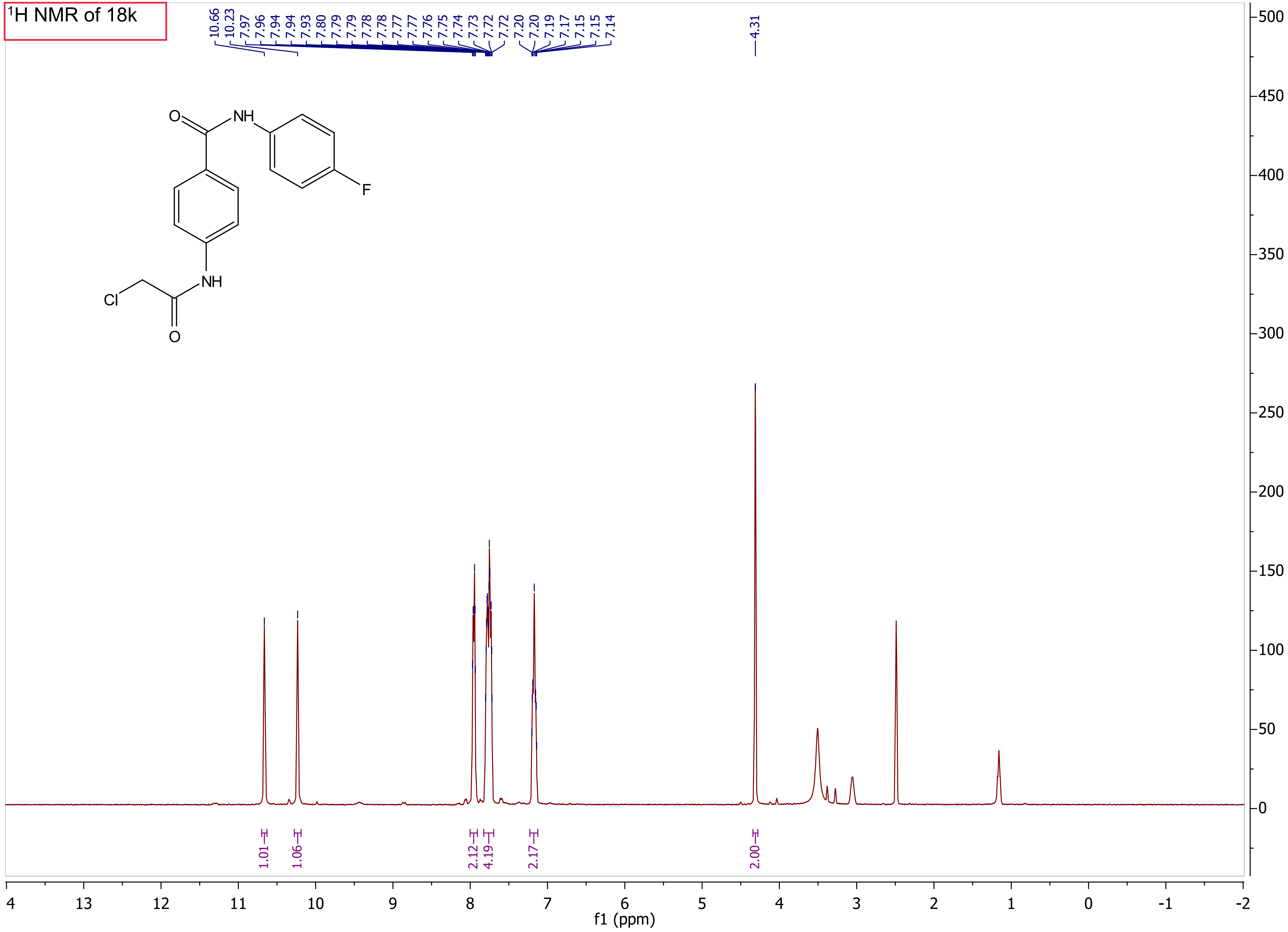

1H NMR of 18k
10.66
10.23
7.97
7.96
7.94
7.94
7.93
7.80
7.79
7.79
7.78
7.78
7.77
7.77
7.76
7.75
7.74
7.73
7.72
7.72
7.20
7.20
7.19
7.17
7.15
7.15
7.14
4.31
O
NH
F
NH
Cl
O
1.01
1.06
2.12
4.19
2.17
2.00
f1 (ppm)

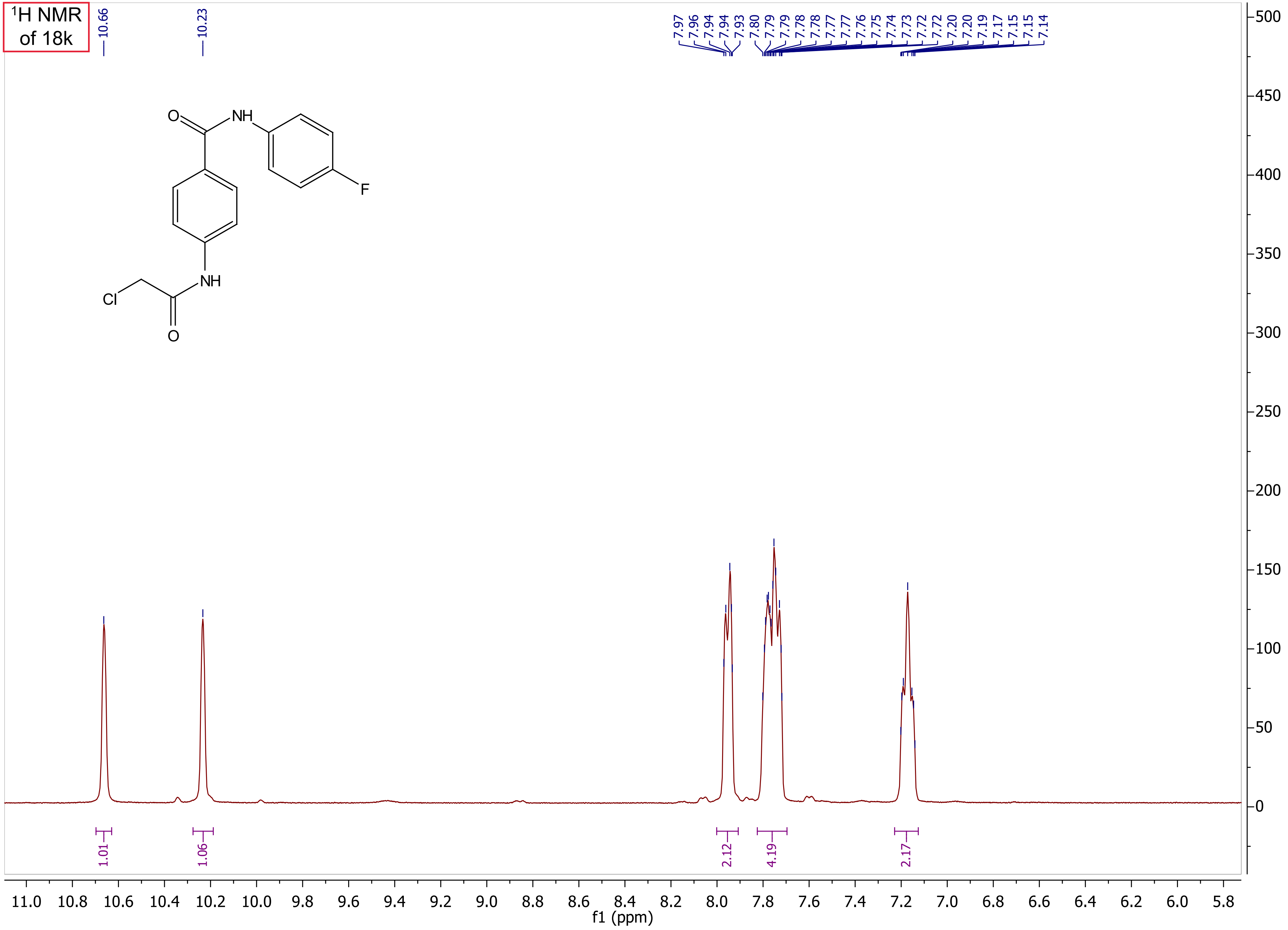

1H NMR
of 18k
10.66
10.23
7.97
7.96
7.94
7.94
7.93
7.80
7.79
7.79
7.78
7.78
7.77
7.77
7.76
7.75
7.74
7.73
7.72
7.72
7.20
7.20
7.19
7.17
7.15
7.15
7.14
O
NH
F
NH
Cl
O
1.01
1.06
2.12
4.19
2.17
f1 (ppm)

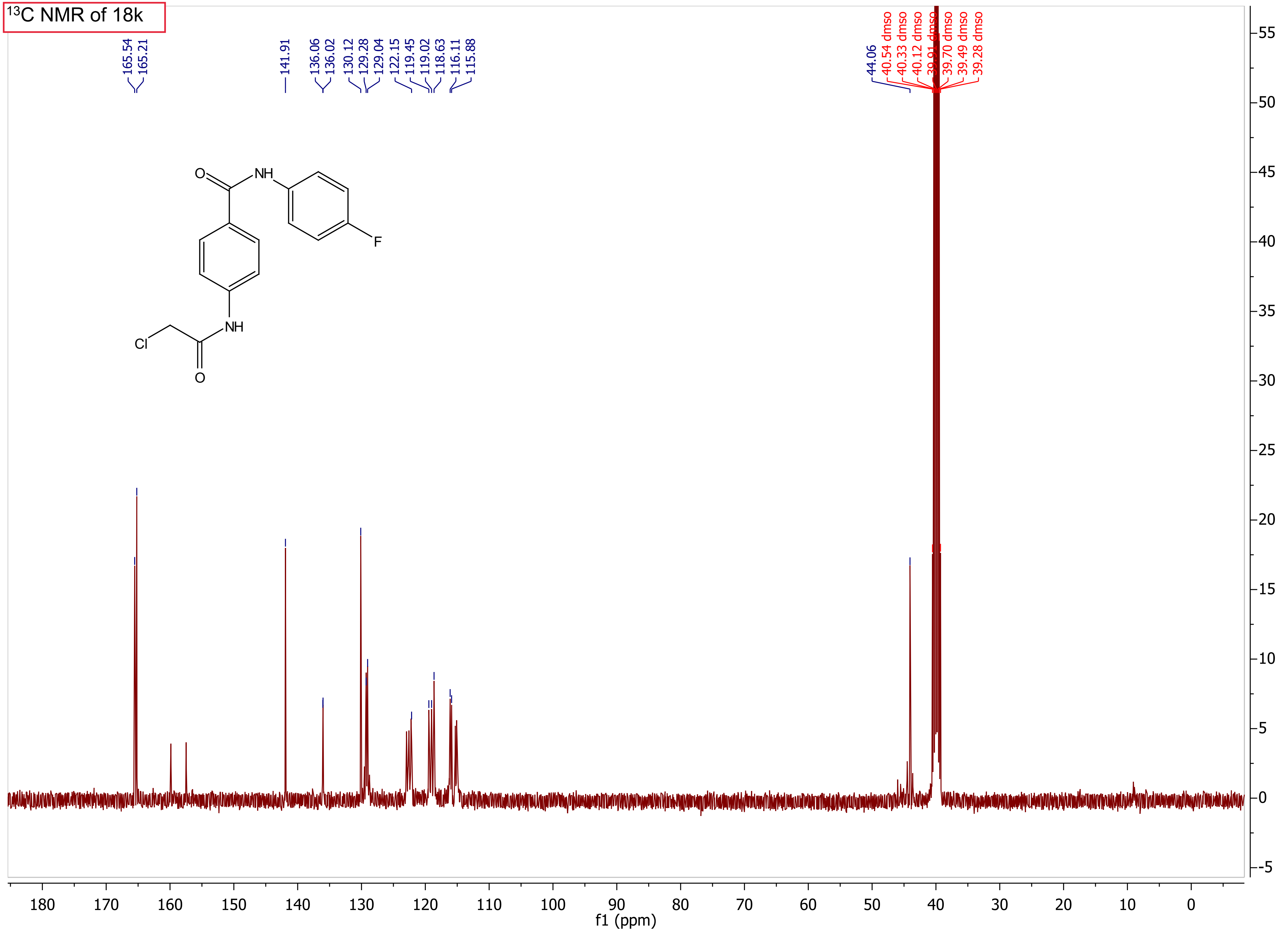
13C NMR of 18k
165.54
165.21
141.91
136.06
136.02
130.12
129.28
129.04
122.15
119.45
119.02
118.63
116.11
115.88
44.06
40.54 dmso
40.33 dmso
40.12 dmso
39.91 dmso
39.70 dmso
39.49 dmso
39.28 dmso
O
NH
F
NH
Cl
O
f1 (ppm)

IR of compound 18l

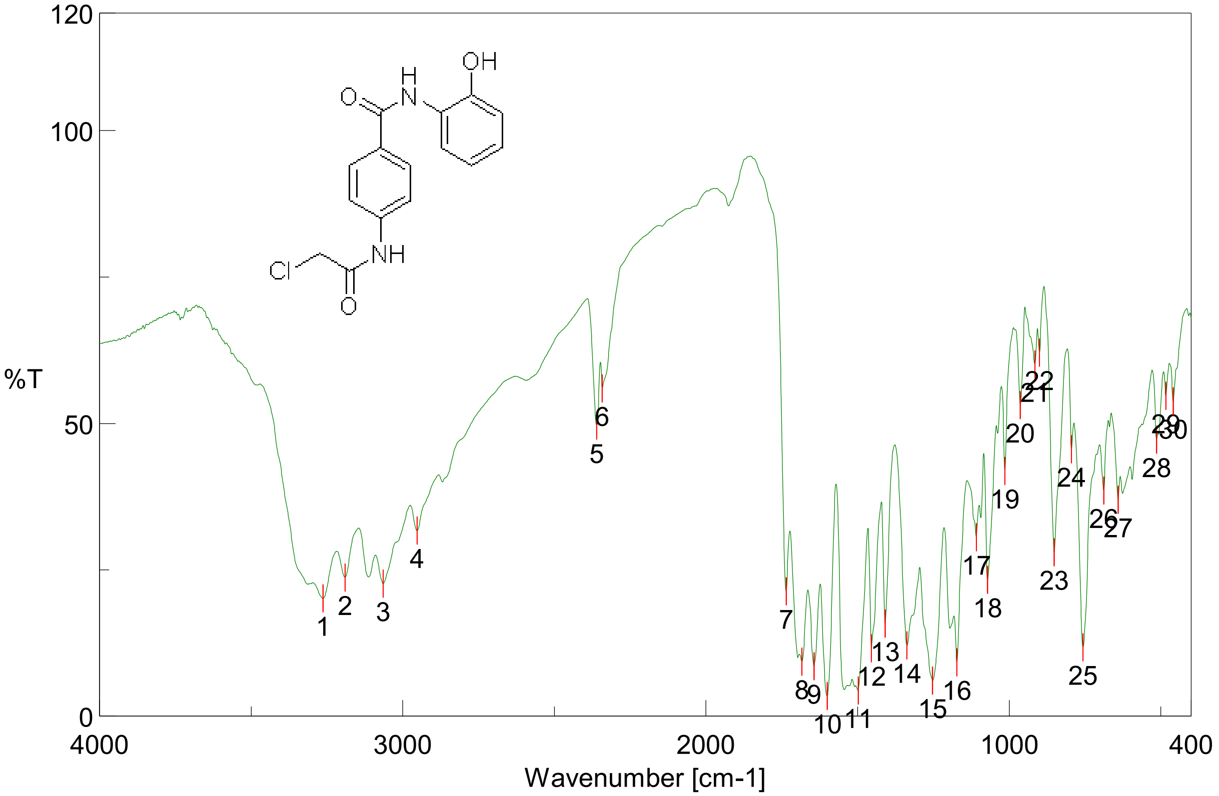

[Comments]

Sample name	A24
Comment	
User	
Division	
Company	KSU

[Detailed Information]

Creation date	9/15/2020 4:51 AM
Data array type	Linear data array
Horizontal axis	Wavenumber [cm-1]
Vertical axis	%T
Start	399.193 cm-1
End	4000.6 cm-1
Data interval	0.964233 cm-1
Data points	3736

[Measurement Information]

Model Name	FT/IR-6600typeA
Serial Number	A014661790
Measurement Date	9/15/2020 4:49 AM
Light Source	Standard
Detector	TGS
Accumulation	Auto (25)
Resolution	4 cm-1
Zero Filling	On
Apodization	Cosine
Gain	Auto (2)
Aperture	Auto (7.1 mm)
Scanning Speed	Auto (2 mm/sec)
Filter	Auto (10000 Hz)

[Result of Peak Picking]

No.	Position	Intensity	No.	Position	Intensity
1	3262.97	20.0971	2	3190.65	23.676

[Result of Peak Picking]

No.	Position	Intensity	No.	Position	Intensity
3	3064.33	22.6098	4	2952.48	31.6703
5	2360.44	49.5947	6	2342.12	55.9356
7	1735.62	21.3042	8	1683.55	9.29712
9	1644.02	8.53021	10	1600.63	3.42178
11	1498.42	4.34892	12	1455.03	11.5598
13	1409.71	15.7758	14	1337.39	12.0519
15	1252.54	6.06166	16	1172.51	9.16555
17	1108.87	30.5309	18	1071.26	23.2483
19	1014.37	41.8532	20	963.269	53.0687
21	915.058	60.0507	22	900.594	62.0425
23	851.418	27.9871	24	794.528	45.5427
25	756.923	11.7358	26	688.463	38.515
27	641.215	36.9235	28	513.936	47.2129
29	483.081	54.6877	30	458.975	53.7331

IR of compound 18m

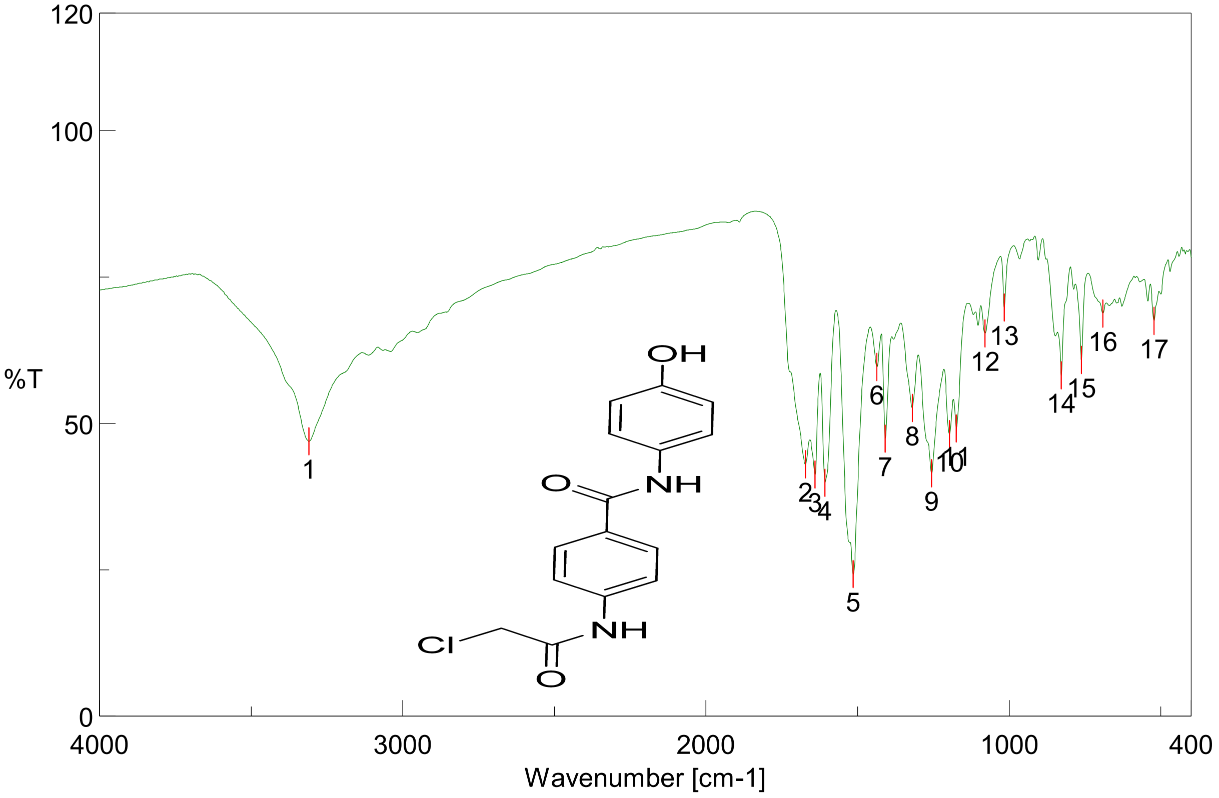

[Comments]

Sample name	A40
Comment	
User	
Division	
Company	KSU

[Detailed Information]

Creation date	9/15/2020 5:58 AM
Data array type	Linear data array
Horizontal axis	Wavenumber [cm-1]
Vertical axis	%T
Start	399.193 cm-1
End	4000.6 cm-1
Data interval	0.964233 cm-1
Data points	3736

[Measurement Information]

Model Name	FT/IR-6600typeA
Serial Number	A014661790
Measurement Date	9/15/2020 5:57 AM
Light Source	Standard
Detector	TGS
Accumulation	Auto (16)
Resolution	4 cm-1
Zero Filling	On
Apodization	Cosine
Gain	Auto (1)
Aperture	Auto (7.1 mm)
Scanning Speed	Auto (2 mm/sec)
Filter	Auto (10000 Hz)

[Result of Peak Picking]

No.	Position	Intensity	No.	Position	Intensity
1	3309.25	46.9297	2	1671.98	42.981

[Result of Peak Picking]

No.	Position	Intensity	No.	Position	Intensity
3	1640.16	41.2246	4	1607.38	39.8214
5	1514.81	24.2343	6	1436.71	59.579
7	1408.75	47.3328	8	1320.04	52.6115
9	1256.4	41.4319	10	1197.58	48.113
11	1174.44	49.1201	12	1079.94	65.3151
13	1016.3	69.7695	14	828.277	58.1768
15	761.744	60.7663	16	691.355	68.7259
17	522.615	67.4649			

IR of compound 18n

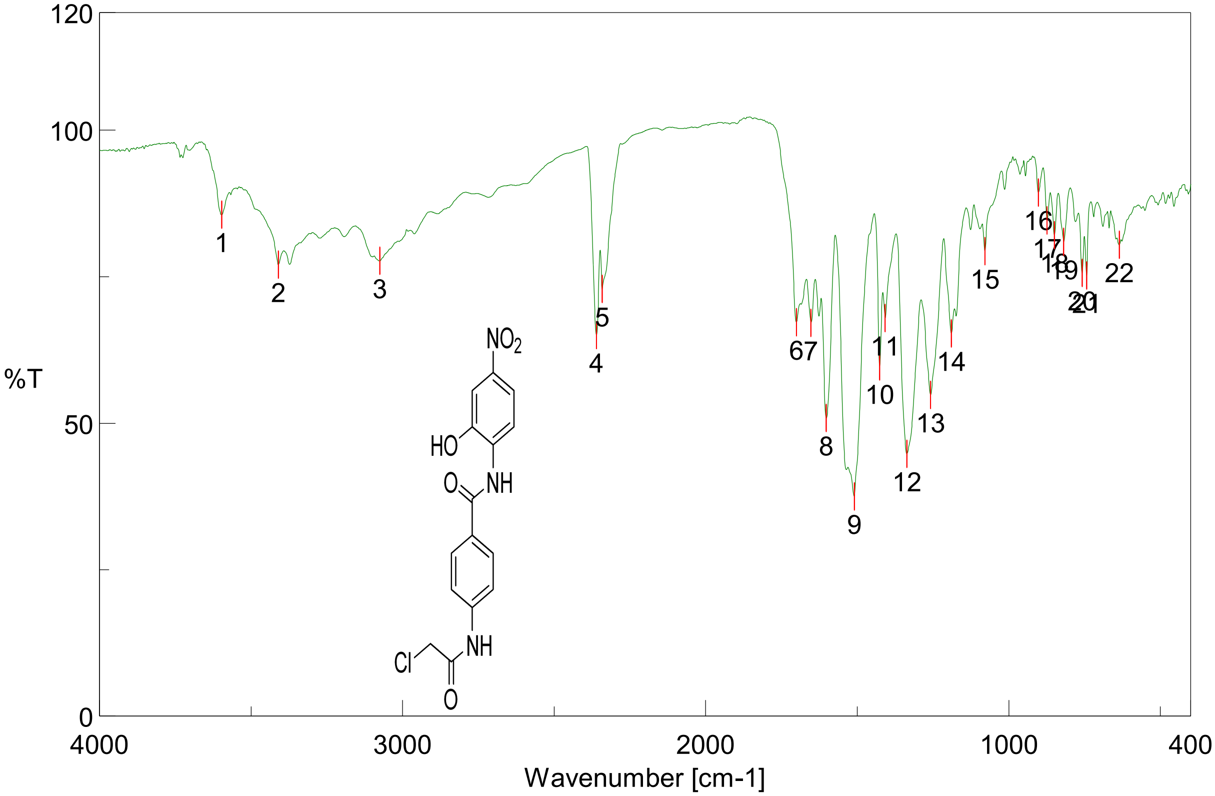

[Comments]

Sample name	A26
Comment	
User	
Division	
Company	KSU

[Detailed Information]

Creation date	9/15/2020 4:53 AM
Data array type	Linear data array
Horizontal axis	Wavenumber [cm-1]
Vertical axis	%T
Start	399.193 cm-1
End	4000.6 cm-1
Data interval	0.964233 cm-1
Data points	3736

[Measurement Information]

Model Name	FT/IR-6600typeA
Serial Number	A014661790
Measurement Date	9/15/2020 4:52 AM
Light Source	Standard
Detector	TGS
Accumulation	Auto (15)
Resolution	4 cm-1
Zero Filling	On
Apodization	Cosine
Gain	Auto (1)
Aperture	Auto (7.1 mm)
Scanning Speed	Auto (2 mm/sec)
Filter	Auto (10000 Hz)

[Result of Peak Picking]

No.	Position	Intensity	No.	Position	Intensity
1	3596.59	85.5098	2	3409.53	76.9988

[Result of Peak Picking]

No.	Position	Intensity	No.	Position	Intensity
3	3074.94	77.6589	4	2360.44	64.976
5	2341.16	72.8543	6	1700.91	67.2092
7	1652.7	67.057	8	1602.56	50.835
9	1509.99	37.4719	10	1426.1	59.6212
11	1407.78	67.8722	12	1336.43	44.7375
13	1258.32	54.7933	14	1189.86	65.2957
15	1078.98	79.3275	16	902.523	89.3054
17	873.596	84.5353	18	849.49	82.0578
19	818.634	80.9059	20	757.888	75.6095
21	743.424	75.1804	22	635.43	80.3884

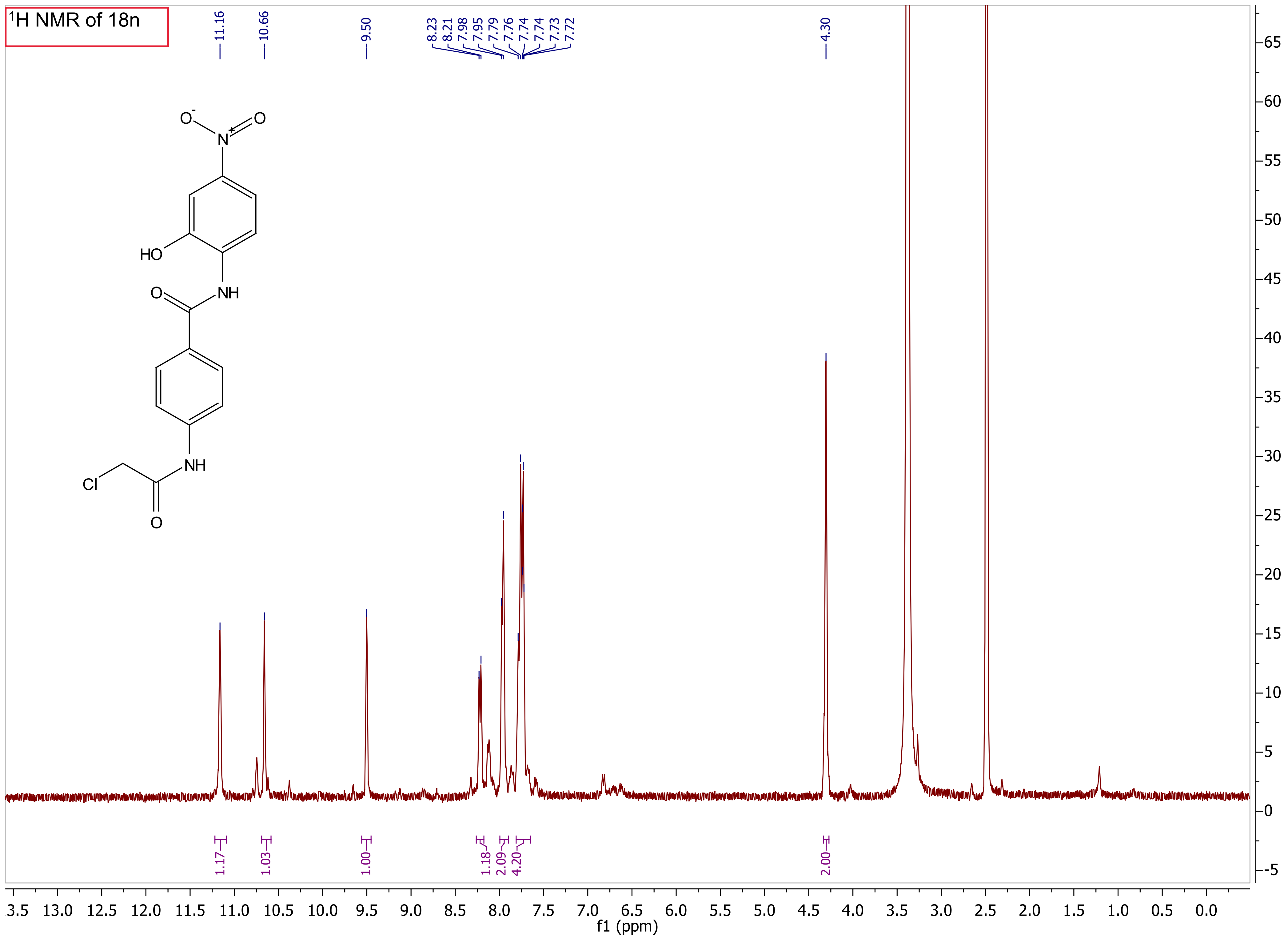

^{1}H NMR of 18n
11.16
10.66
9.50
8.23
8.21
7.98
7.95
7.79
7.76
7.74
7.74
7.73
7.72
4.30
1.17
1.03
1.00
1.18
2.09
4.20
2.00
f1 (ppm)
HO
NH
O
Cl

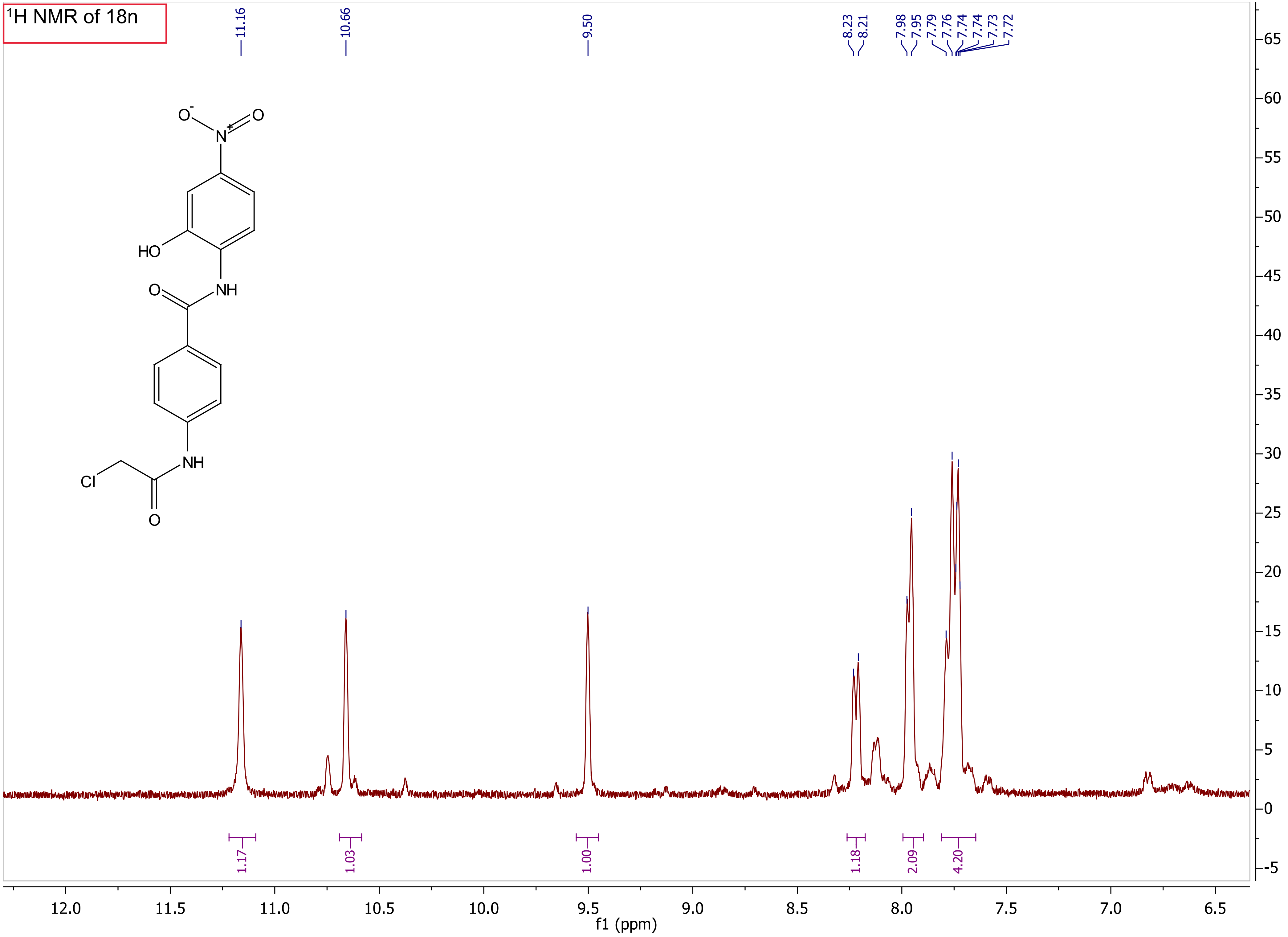

1H NMR of 18n
11.16
10.66
9.50
8.23
8.21
7.98
7.95
7.79
7.76
7.74
7.74
7.73
7.72
O-
N+
O
HO
NH
O
NH
Cl
O
1.17
1.03
1.00
1.18
2.09
4.20
12.0
11.5
11.0
10.5
10.0
9.5
9.0
8.5
8.0
7.5
7.0
6.5
f1 (ppm)
65
60
55
50
45
40
35
30
25
20
15
10
5
0
-5

13C NMR of 18n

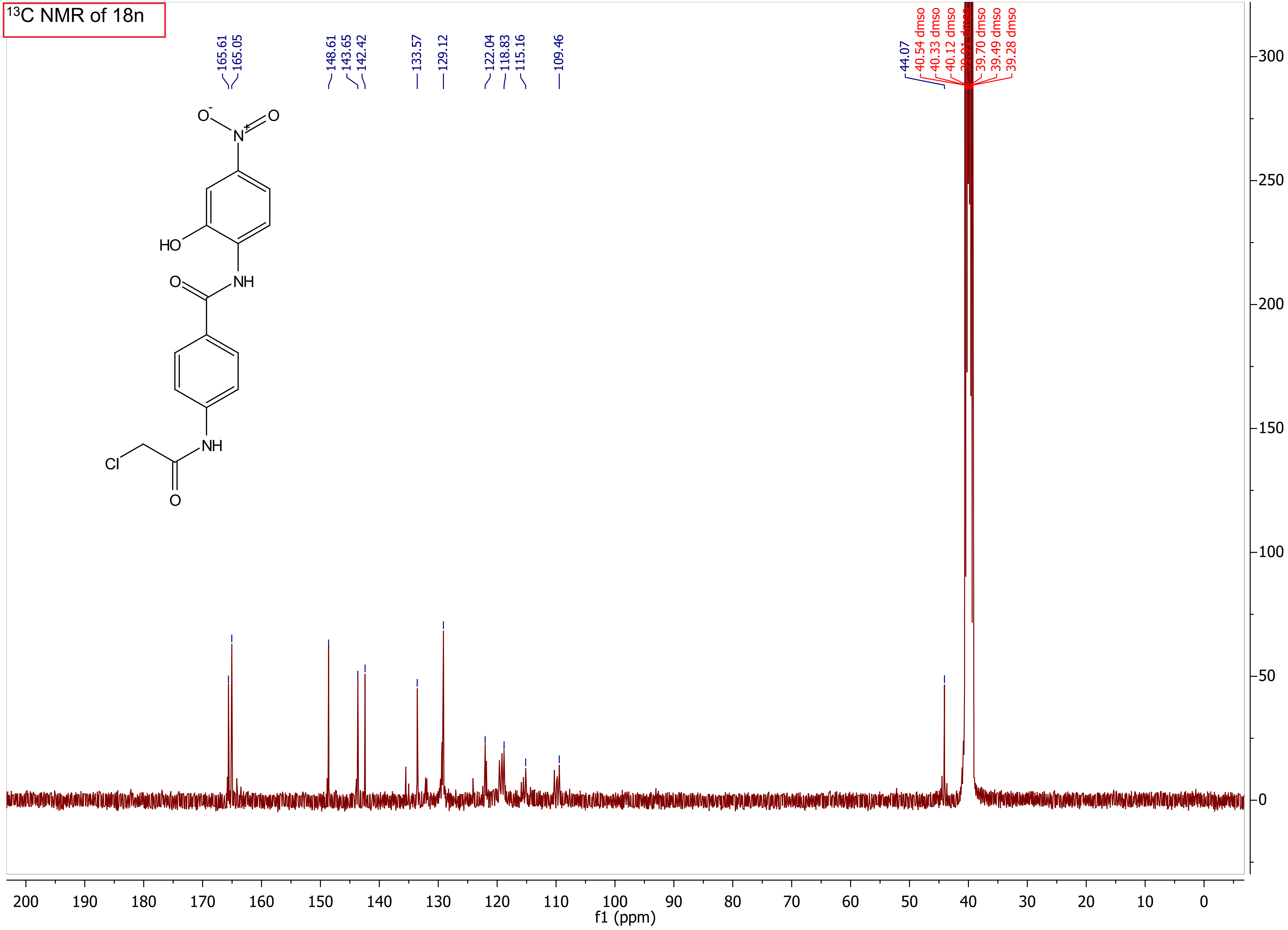
165.61
165.05
148.61
143.65
142.42
133.57
129.12
122.04
118.83
115.16
109.46
44.07
40.54 dmso
40.33 dmso
40.12 dmso
39.70 dmso
39.49 dmso
39.28 dmso
O-
N+
O
HO
NH
O
NH
Cl
O
f1 (ppm)

IR of compound 22a

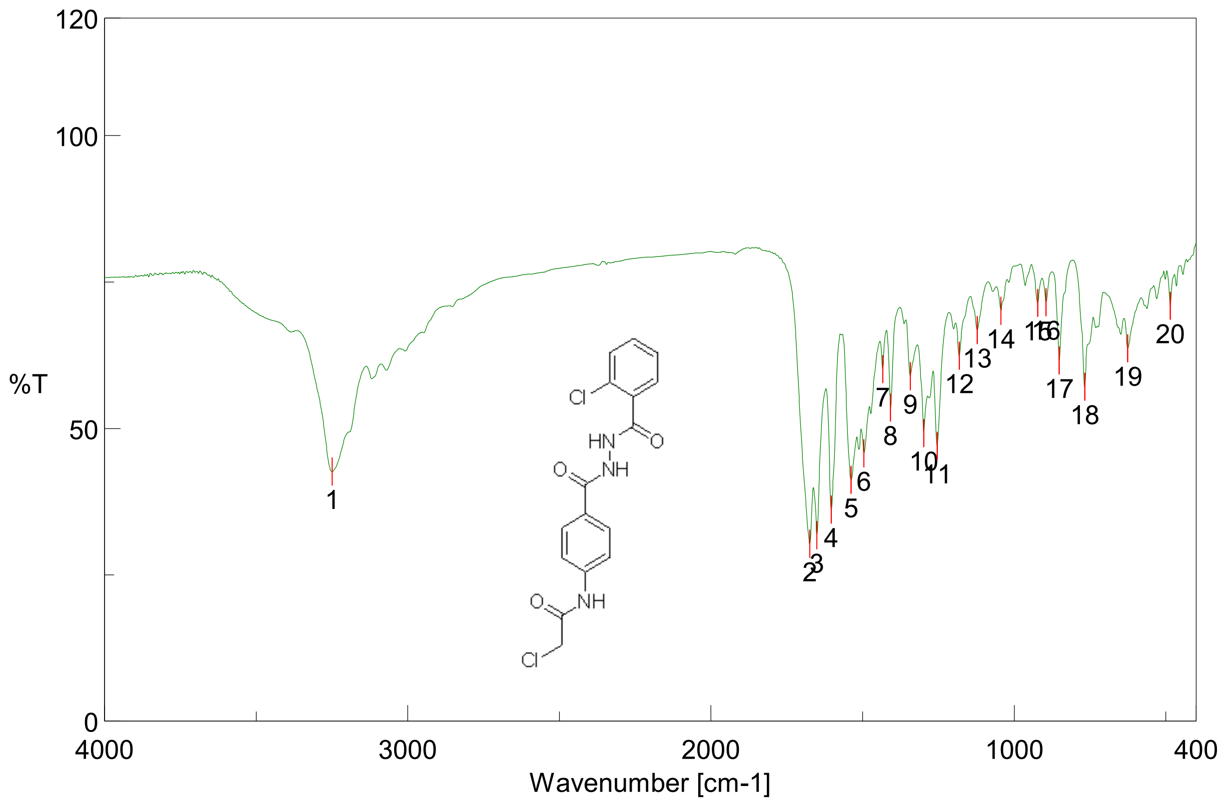

[Comments]

Sample name	A56
Comment	
User	
Division	
Company	KSU

[Detailed Information]

Creation date	9/15/2020 6:15 AM
Data array type	Linear data array
Horizontal axis	Wavenumber [cm-1]
Vertical axis	%T
Start	399.193 cm-1
End	4000.6 cm-1
Data interval	0.964233 cm-1
Data points	3736

[Measurement Information]

Model Name	FT/IR-6600typeA
Serial Number	A014661790
Measurement Date	9/15/2020 6:13 AM
Light Source	Standard
Detector	TGS
Accumulation	Auto (15)
Resolution	4 cm-1
Zero Filling	On
Apodization	Cosine
Gain	Auto (1)
Aperture	Auto (7.1 mm)
Scanning Speed	Auto (2 mm/sec)
Filter	Auto (10000 Hz)

[Result of Peak Picking]

No.	Position	Intensity	No.	Position	Intensity
1	3249.47	42.5828	2	1673.91	30.2488

[Result of Peak Picking]

No.	Position	Intensity	No.	Position	Intensity
3	1650.77	31.7571	4	1603.52	36.1213
5	1537.95	41.1991	6	1495.53	45.738
7	1433.82	60.056	8	1407.78	53.5274
9	1343.18	58.8881	10	1298.82	49.1653
11	1254.47	46.9571	12	1181.19	62.3724
13	1122.37	66.76	14	1044.26	70.0384
15	922.771	71.39	16	894.809	71.4799
17	851.418	61.5648	18	767.53	57.0609
19	625.788	63.6589	20	485.009	70.9238

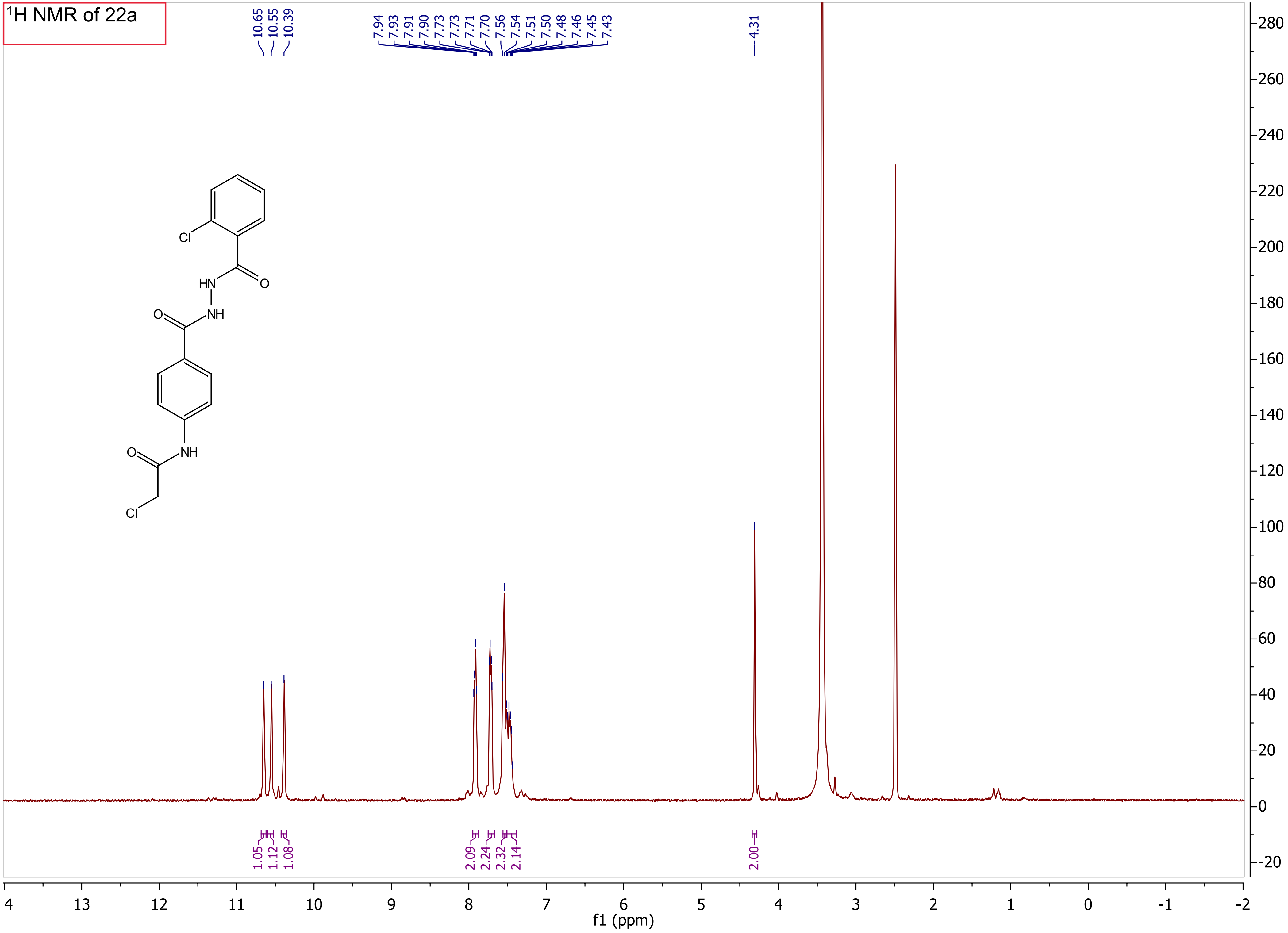

1H NMR of 22a
10.65
10.55
10.39
7.94
7.93
7.91
7.90
7.73
7.73
7.71
7.70
7.56
7.54
7.51
7.50
7.48
7.46
7.45
7.43
4.31
1.05
1.12
1.08
2.09
2.24
2.32
2.14
2.00
Cl
HN
O
O
NH
NH
O
Cl
f1 (ppm)

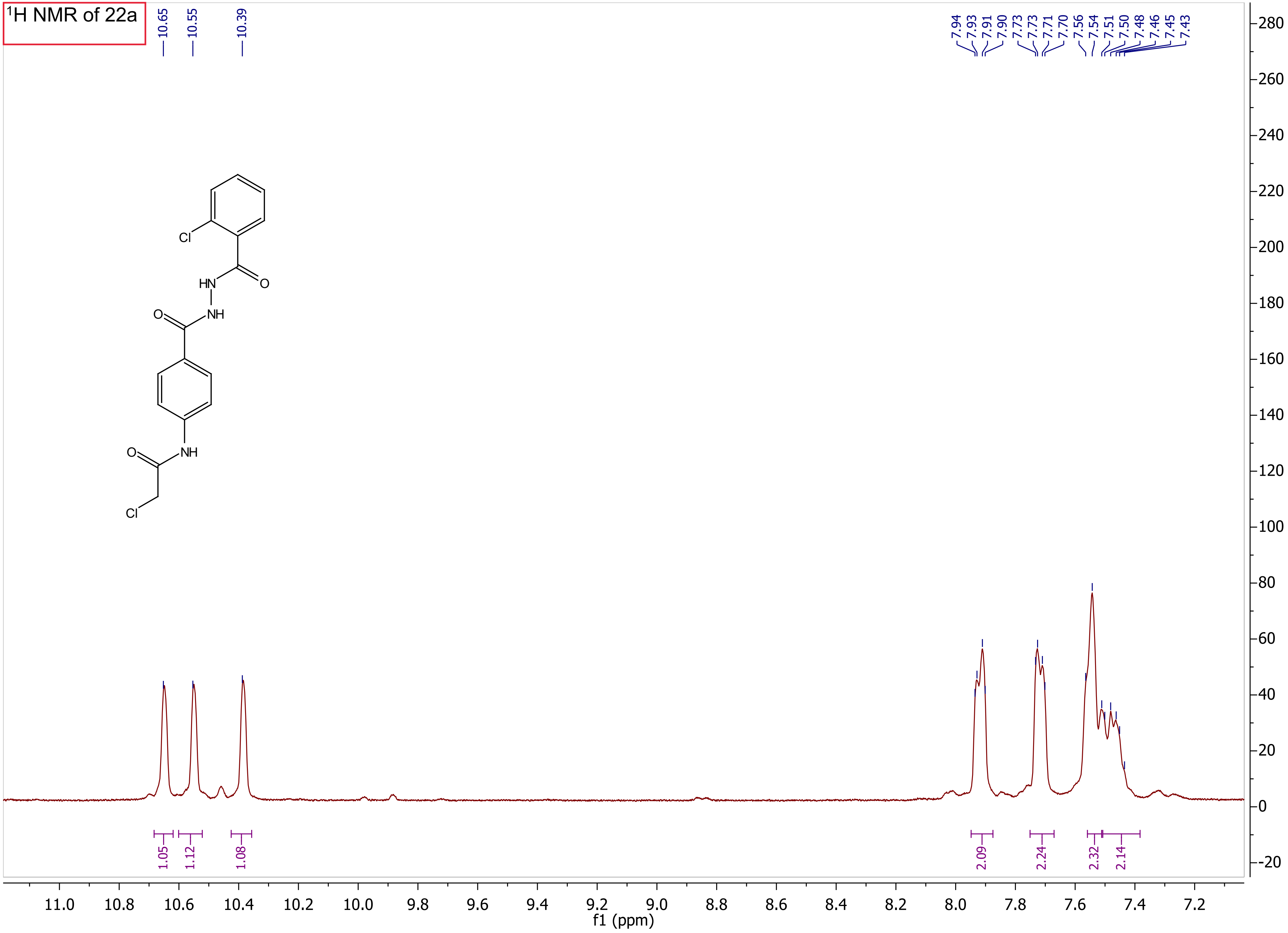

1H NMR of 22a
10.65
10.55
10.39
7.94
7.93
7.91
7.90
7.73
7.73
7.71
7.70
7.56
7.54
7.51
7.50
7.48
7.46
7.45
7.43
Cl
HN
O
NH
O
NH
O
Cl
1.05
1.12
1.08
2.09
2.24
2.32
2.14
f1 (ppm)

IR of compound 22b

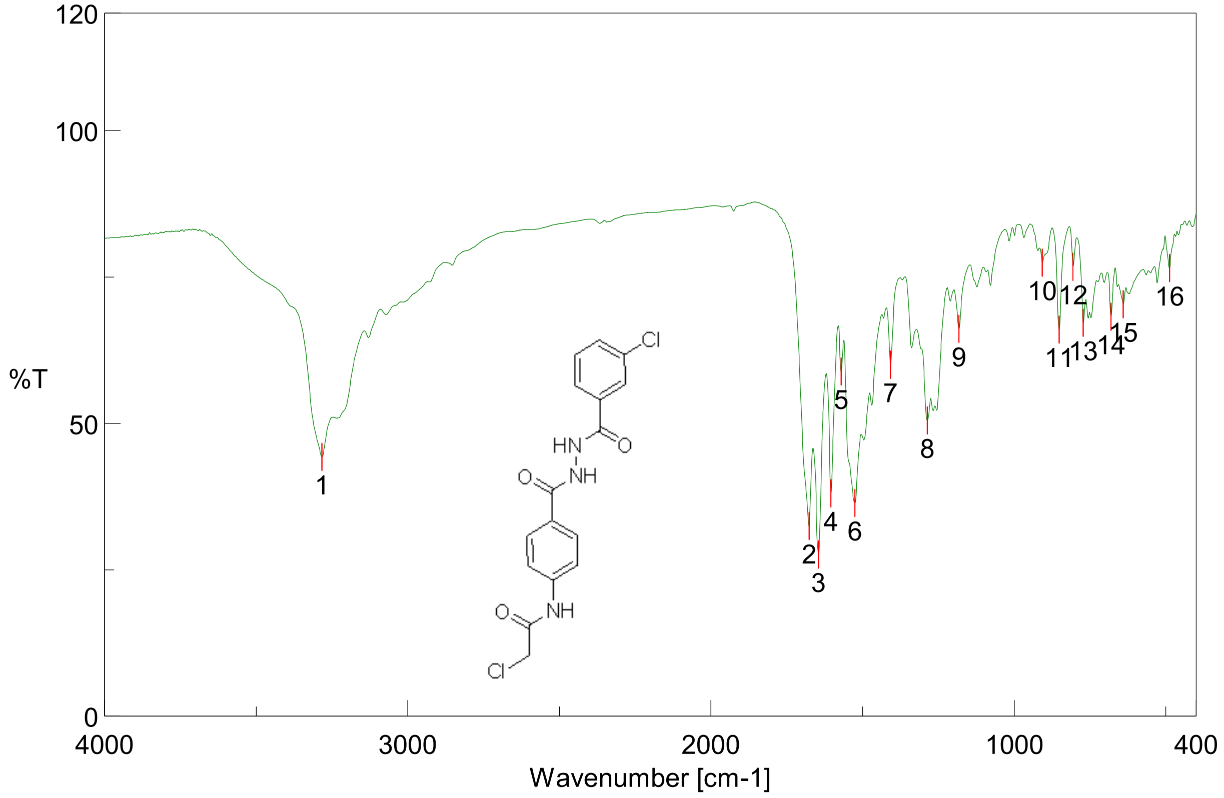

[Comments]

Sample name	A54
Comment	
User	
Division	
Company	KSU

[Detailed Information]

Creation date	9/15/2020 6:09 AM
Data array type	Linear data array
Horizontal axis	Wavenumber [cm-1]
Vertical axis	%T
Start	399.193 cm-1
End	4000.6 cm-1
Data interval	0.964233 cm-1
Data points	3736

[Measurement Information]

Model Name	FT/IR-6600typeA
Serial Number	A014661790
Measurement Date	9/15/2020 6:07 AM
Light Source	Standard
Detector	TGS
Accumulation	Auto (14)
Resolution	4 cm-1
Zero Filling	On
Apodization	Cosine
Gain	Auto (1)
Aperture	Auto (7.1 mm)
Scanning Speed	Auto (2 mm/sec)
Filter	Auto (10000 Hz)

[Result of Peak Picking]

No.	Position	Intensity	No.	Position	Intensity
1	3283.21	44.2527	2	1676.8	32.4174

[Result of Peak Picking]

No.	Position	Intensity	No.	Position	Intensity
3	1645.95	27.5931	4	1604.48	37.9989
5	1570.74	58.8117	6	1525.42	36.3556
7	1407.78	59.9981	8	1287.25	50.4587
9	1182.15	66.0943	10	907.344	77.3705
11	851.418	65.981	12	806.099	76.7063
13	772.351	67.1082	14	680.749	68.1672
15	640.251	70.2956	16	487.902	76.4425

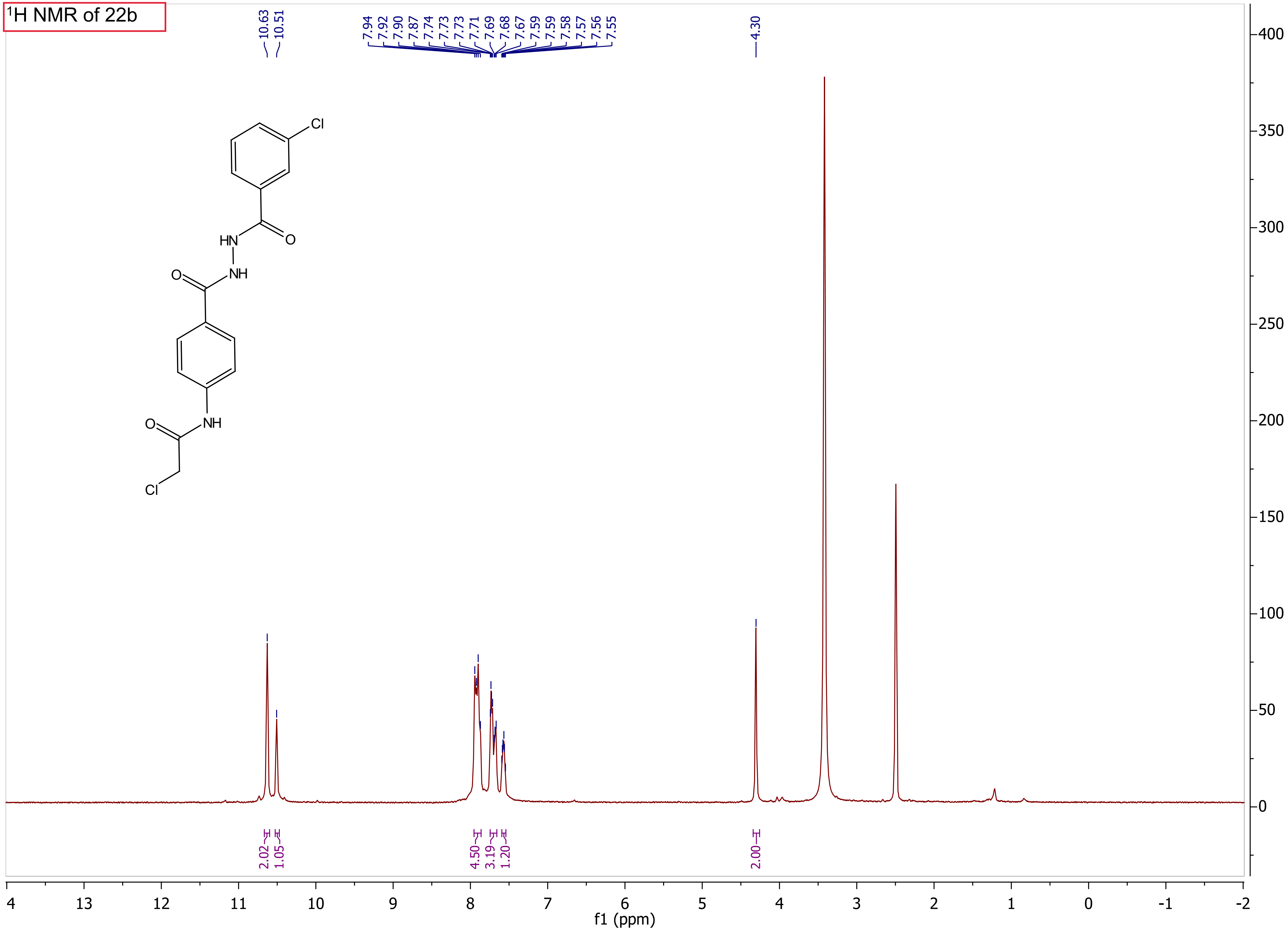

1H NMR of 22b
10.63
10.51
7.94
7.92
7.90
7.87
7.74
7.73
7.73
7.71
7.69
7.68
7.67
7.59
7.59
7.58
7.57
7.56
7.55
4.30
Cl
HN
O
O
NH
NH
O
Cl
2.02
1.05
4.50
3.19
1.20
2.00
f1 (ppm)

^{1}H NMR of 22b

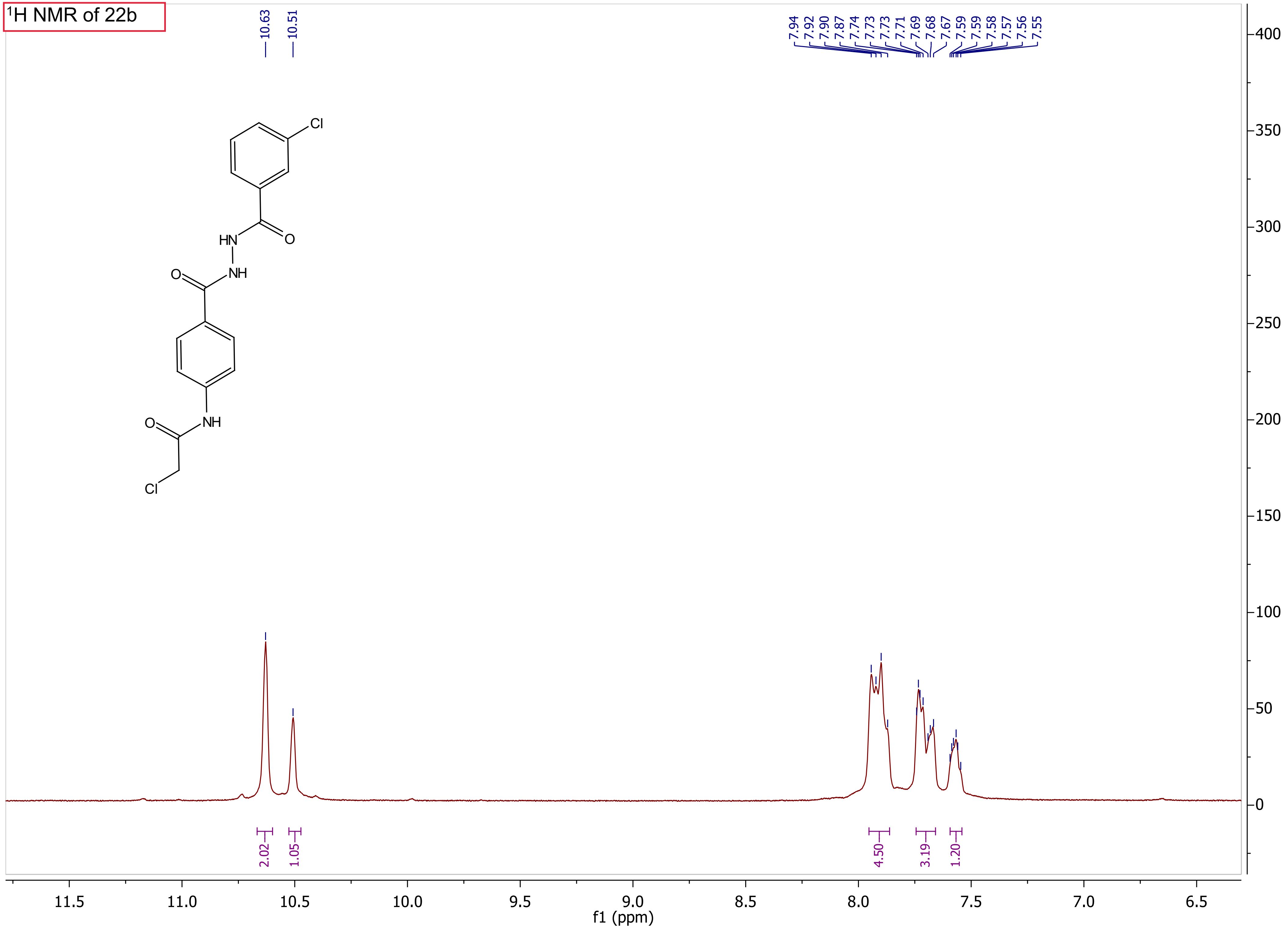

10.63
10.51
7.94
7.92
7.90
7.87
7.74
7.73
7.73
7.71
7.69
7.68
7.67
7.59
7.59
7.58
7.57
7.56
7.55
Cl
HN
O
NH
O
NH
O
Cl
2.02
1.05
4.50
3.19
1.20
400
350
300
250
200
150
100
50
0
11.5
11.0
10.5
10.0
9.5
9.0
8.5
8.0
7.5
7.0
6.5
f1 (ppm)

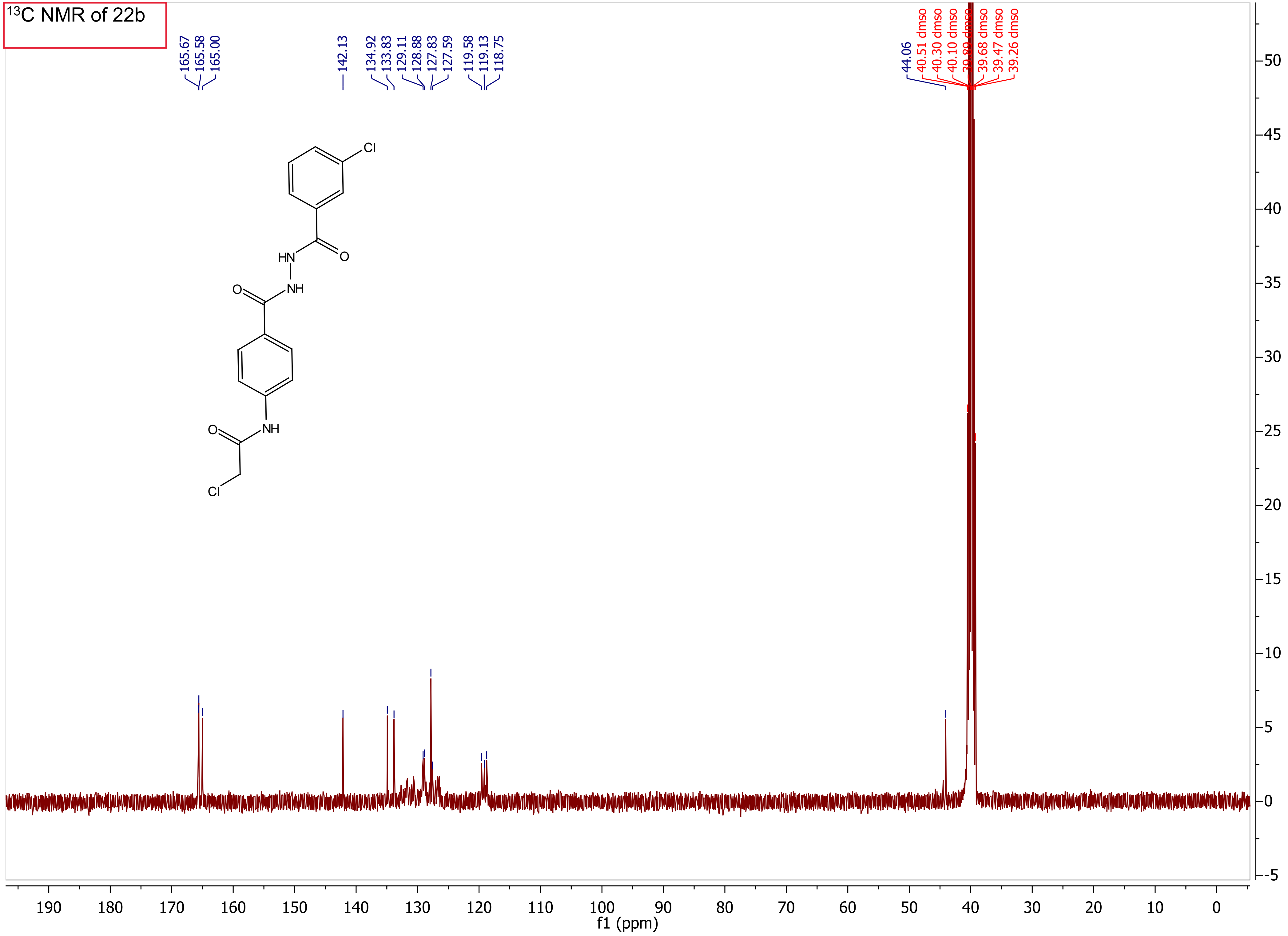

13C NMR of 22b
165.67
165.58
165.00
142.13
134.92
133.83
129.11
128.88
127.83
127.59
119.58
119.13
118.75
44.06
40.51 dmso
40.30 dmso
40.10 dmso
39.89 dmso
39.68 dmso
39.47 dmso
39.26 dmso
Cl
HN
O
NH
O
NH
O
Cl
f1 (ppm)

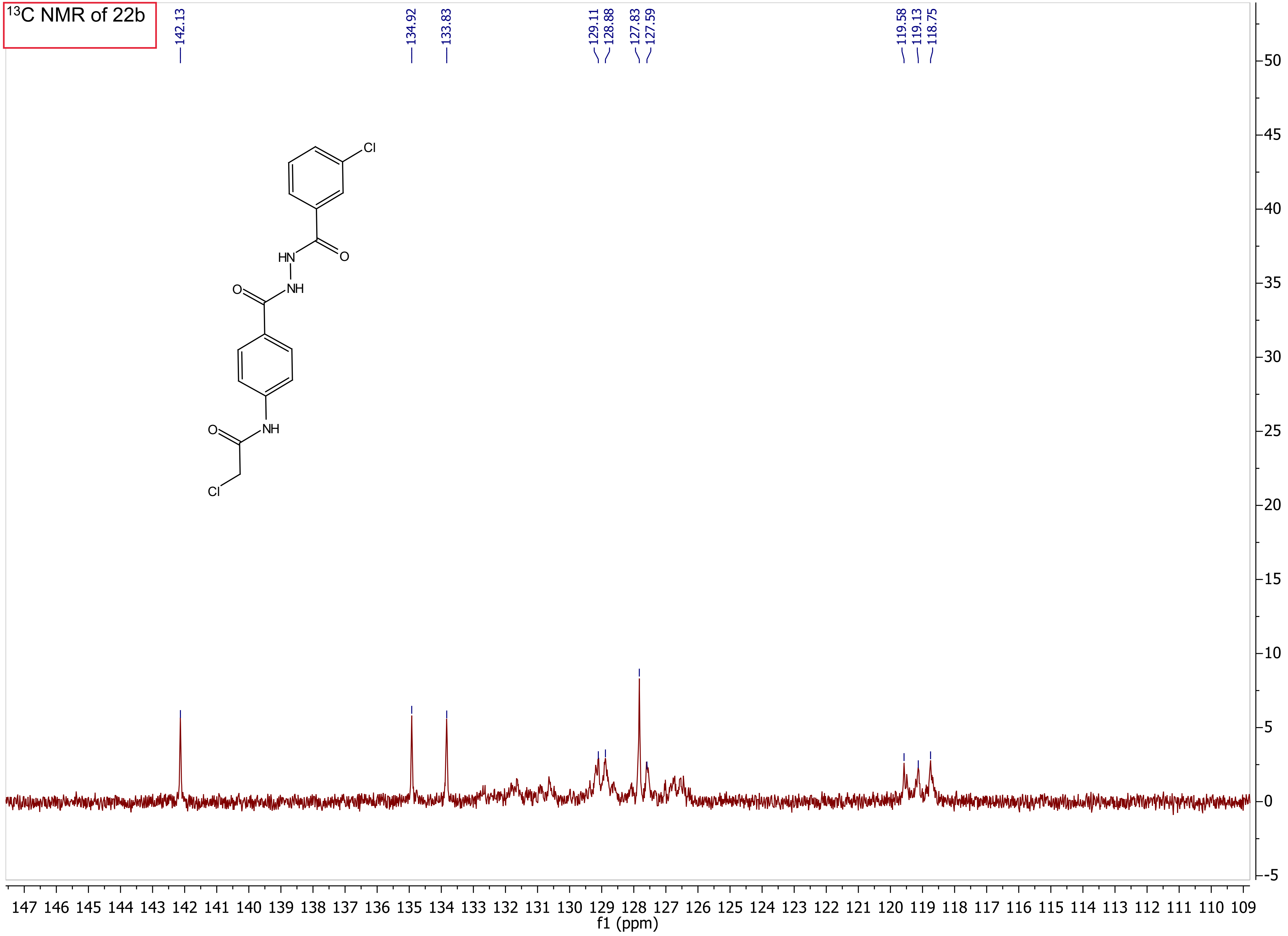

13C NMR of 22b
142.13
134.92
133.83
129.11
128.88
127.83
127.59
119.58
119.13
118.75
Cl
HN
O
NH
O
NH
O
Cl
f1 (ppm)

IR of compound 22c

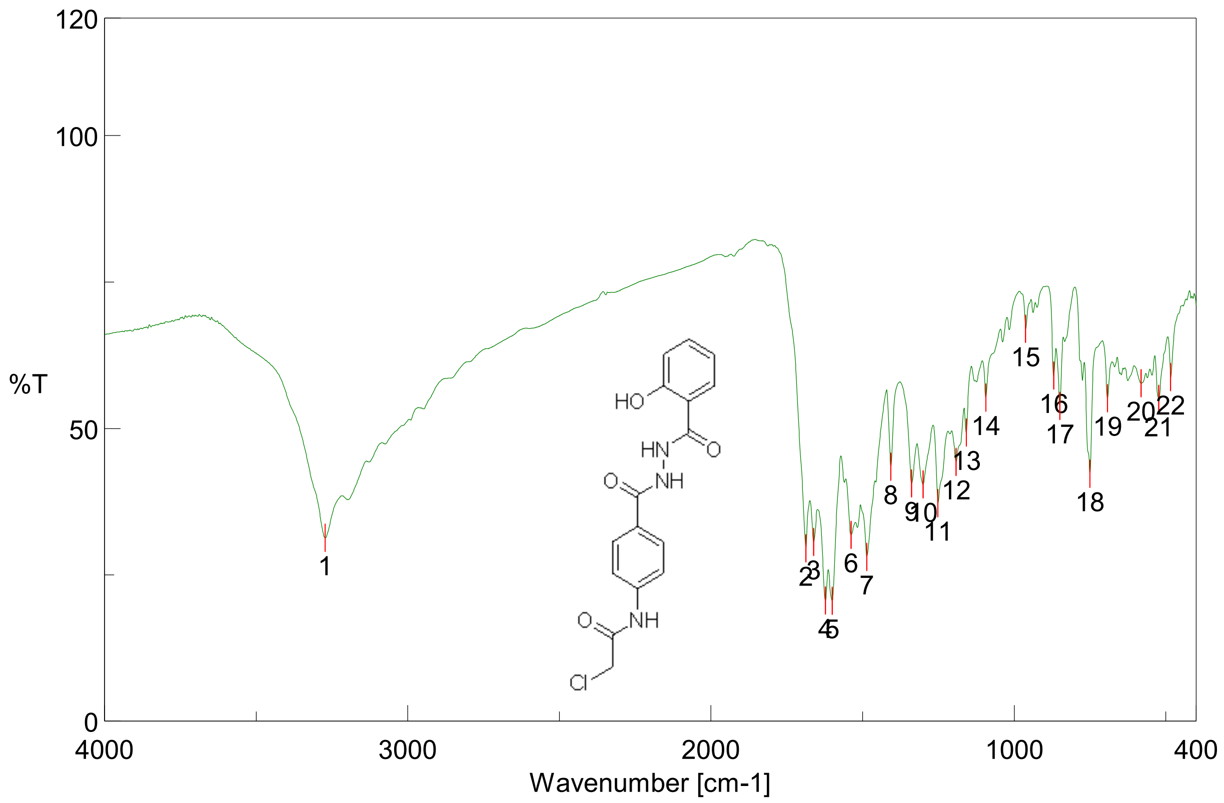

[Comments]

Sample name	A57
Comment	
User	
Division	
Company	KSU

[Detailed Information]

Creation date	9/15/2020 6:20 AM
Data array type	Linear data array
Horizontal axis	Wavenumber [cm-1]
Vertical axis	%T
Start	399.193 cm-1
End	4000.6 cm-1
Data interval	0.964233 cm-1
Data points	3736

[Measurement Information]

Model Name	FT/IR-6600typeA
Serial Number	A014661790
Measurement Date	9/15/2020 6:17 AM
Light Source	Standard
Detector	TGS
Accumulation	Auto (18)
Resolution	4 cm-1
Zero Filling	On
Apodization	Cosine
Gain	Auto (2)
Aperture	Auto (7.1 mm)
Scanning Speed	Auto (2 mm/sec)
Filter	Auto (10000 Hz)

[Result of Peak Picking]

No.	Position	Intensity	No.	Position	Intensity
1	3272.61	31.2826	2	1687.41	29.49

[Result of Peak Picking]

No.	Position	Intensity	No.	Position	Intensity
3	1661.37	30.5599	4	1622.8	20.5842
5	1600.63	20.556	6	1537.95	31.8091
7	1485.88	28.0318	8	1406.82	43.4196
9	1338.36	40.59	10	1300.75	40.3916
11	1251.58	37.2018	12	1191.79	44.259
13	1158.04	49.2871	14	1093.44	55.2116
15	962.305	66.9626	16	869.739	58.9669
17	849.49	53.7754	18	750.174	42.2172
19	692.32	55.0973	20	581.433	57.6606
21	523.579	55.0202	22	484.045	58.7324

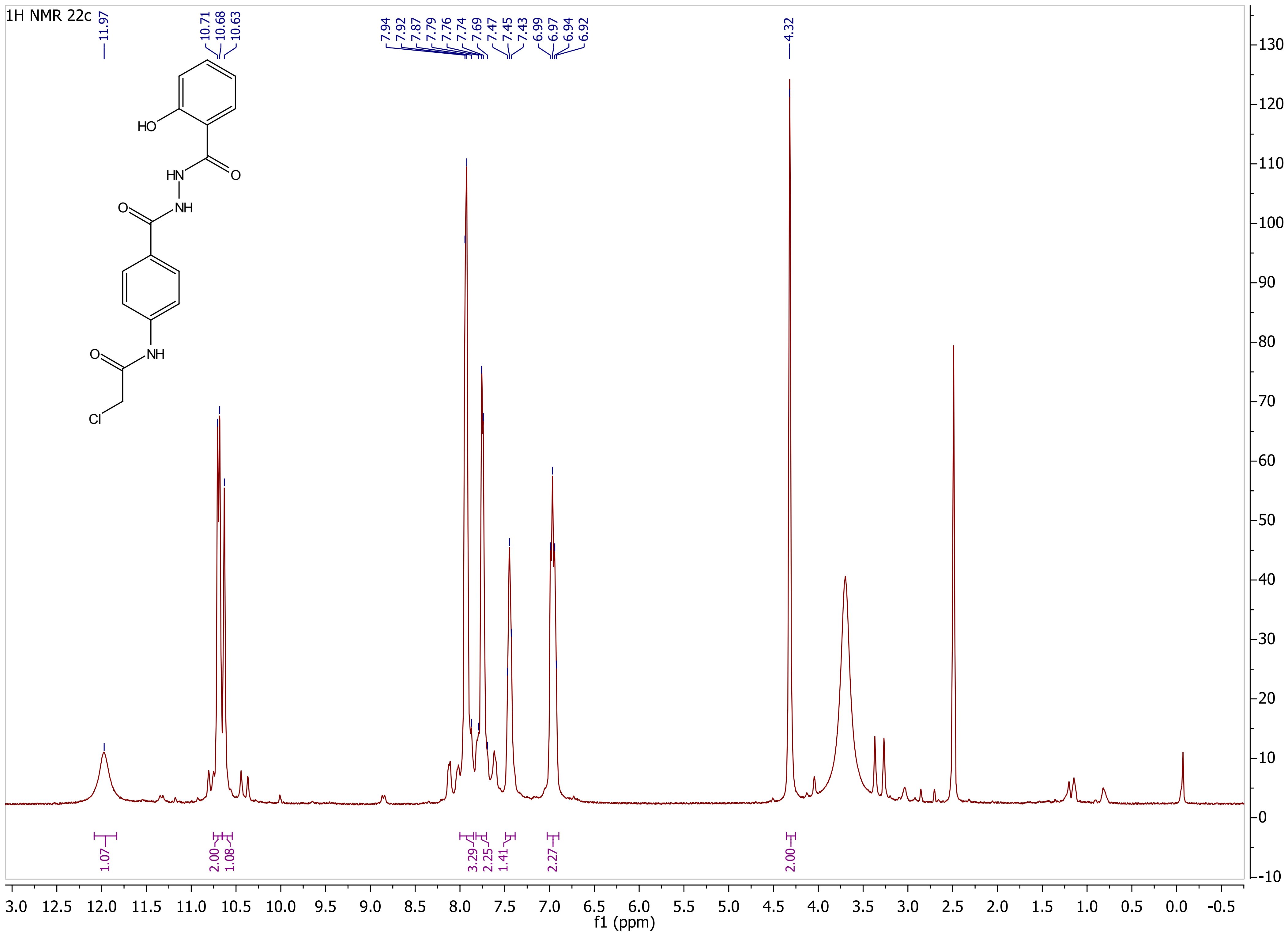
1H NMR 22c
11.97
10.71
10.68
10.63
7.94
7.92
7.87
7.79
7.76
7.74
7.69
7.47
7.45
7.43
6.99
6.97
6.94
6.92
4.32
HO
O
HN
NH
O
NH
O
Cl
1.07
2.00
1.08
3.29
2.25
1.41
2.27
2.00
f1 (ppm)

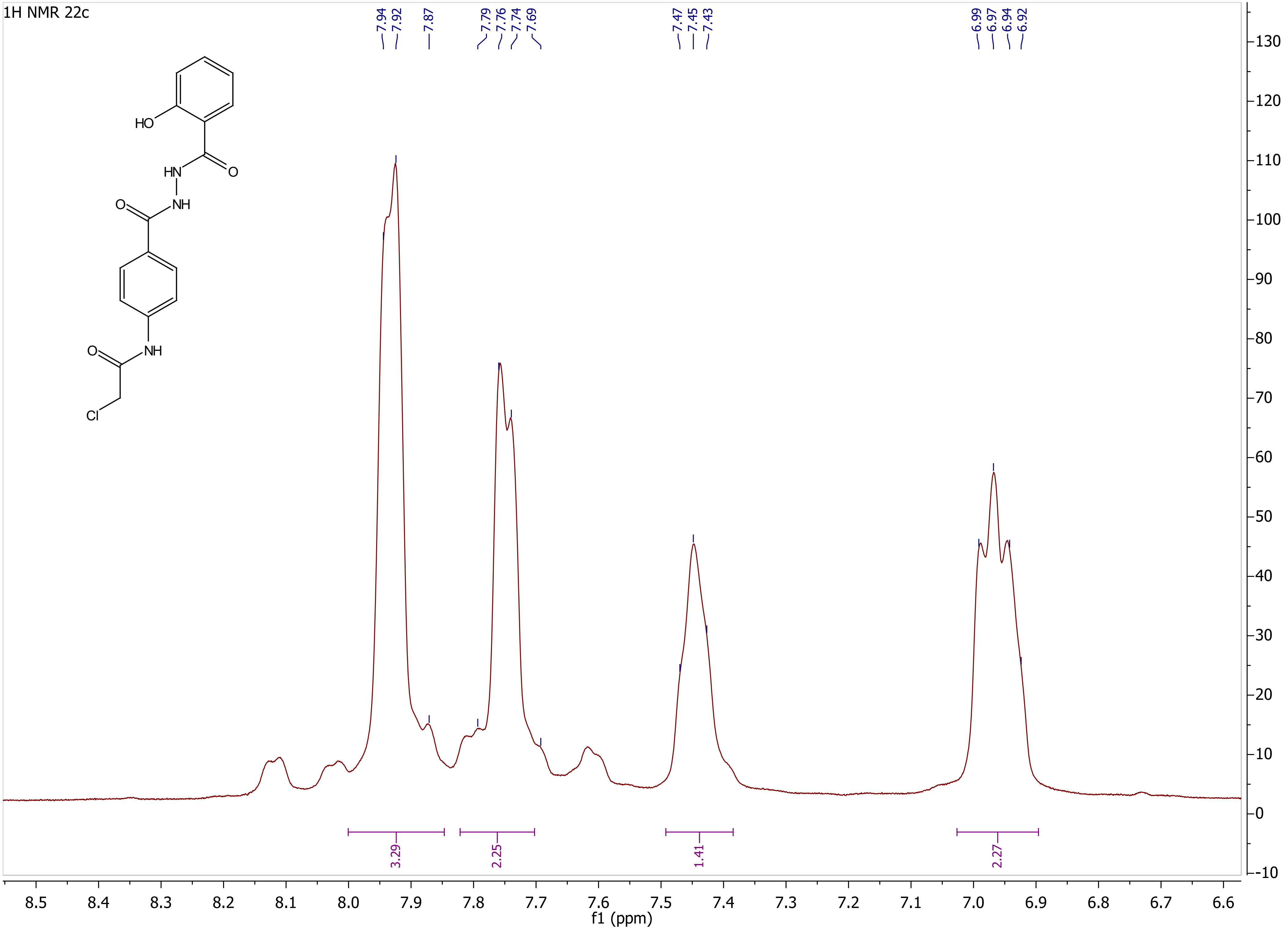

1H NMR 22c
7.94
7.92
7.87
7.79
7.76
7.74
7.69
7.47
7.45
7.43
6.99
6.97
6.94
6.92
HO
HN
O
NH
O
NH
O
Cl
3.29
2.25
1.41
2.27
f1 (ppm)

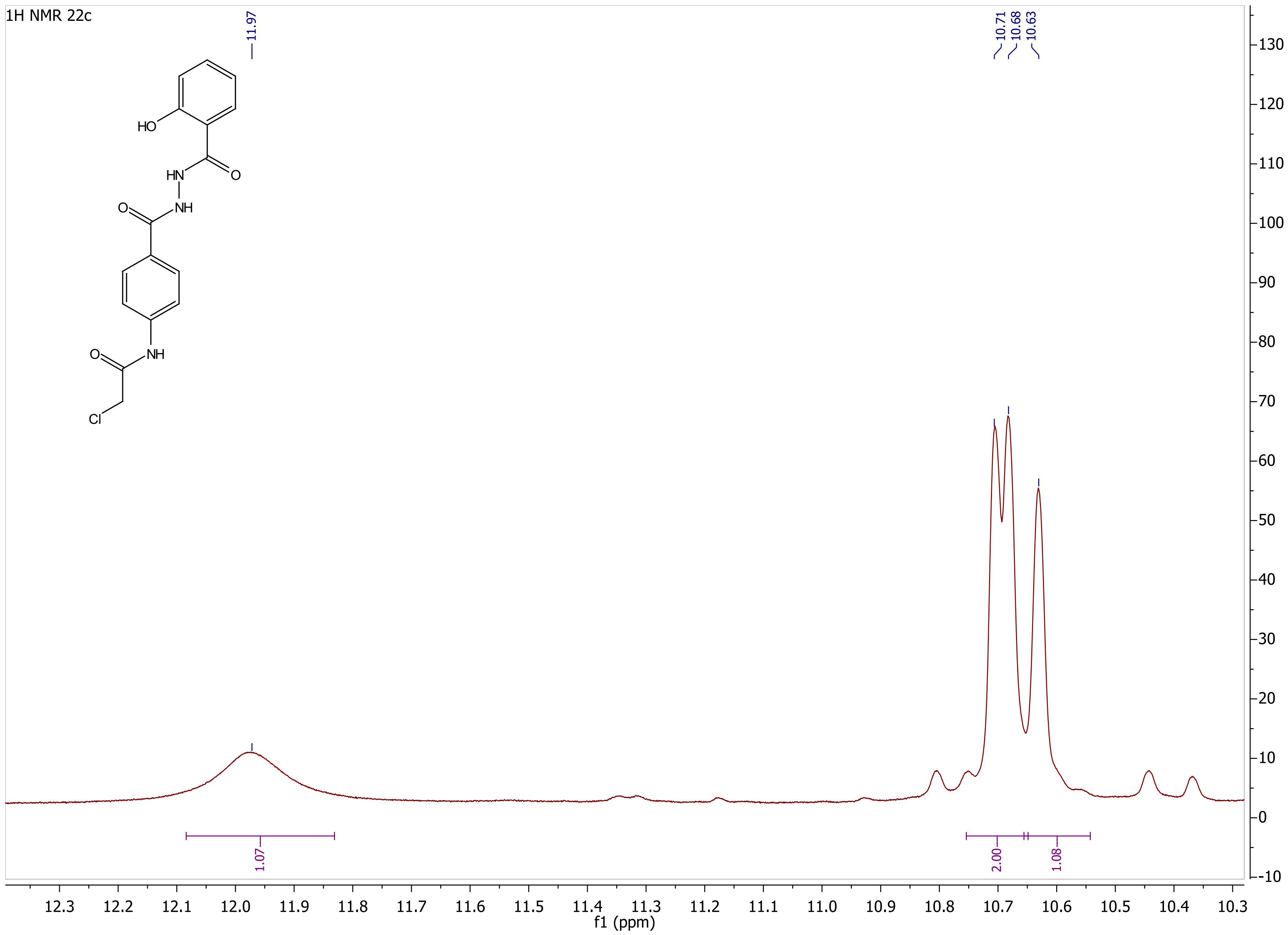

1H NMR 22c
11.97
10.71
10.68
10.63
HO
HN
O
NH
O
NH
O
Cl
1.07
2.00
1.08
f1 (ppm)

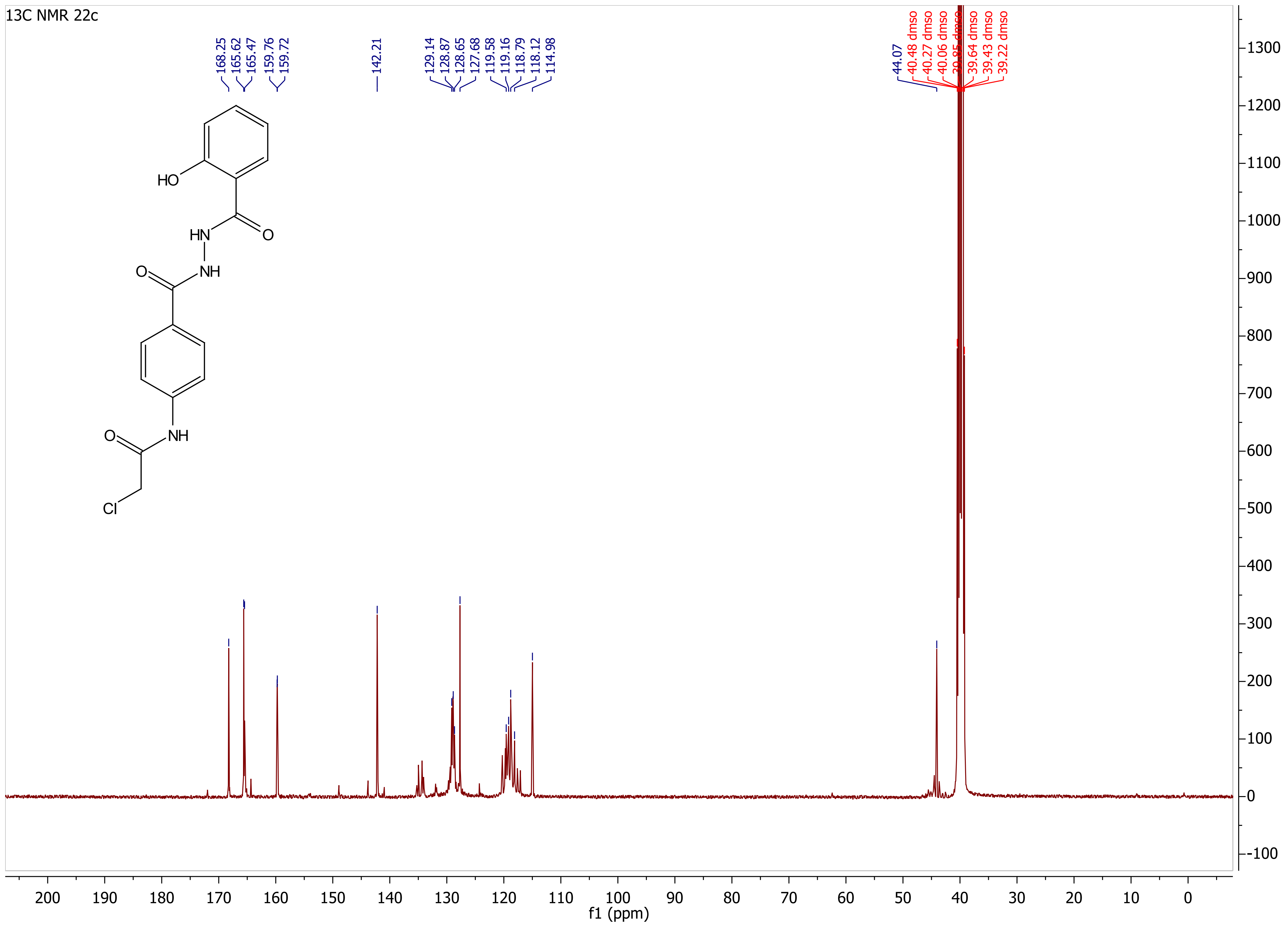

13C NMR 22c
168.25
165.62
165.47
159.76
159.72
142.21
129.14
128.87
128.65
127.68
119.58
119.16
118.79
118.12
114.98
44.07
40.48 dmso
40.27 dmso
40.06 dmso
39.64 dmso
39.43 dmso
39.22 dmso
HO
HN
O
NH
O
NH
O
Cl
f1 (ppm)

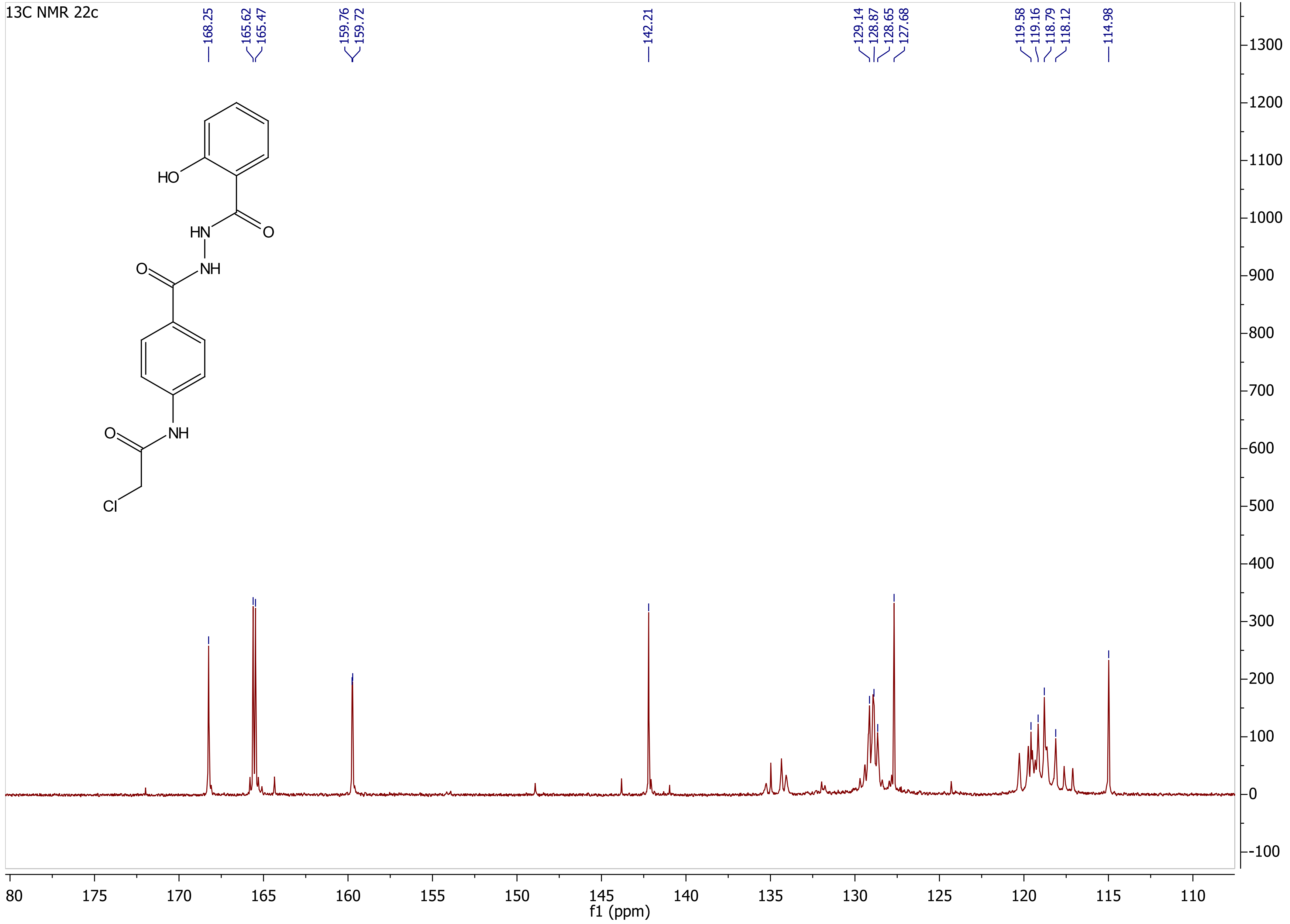
13C NMR 22c
168.25
165.62
165.47
159.76
159.72
142.21
129.14
128.87
128.65
127.68
119.58
119.16
118.79
118.12
114.98
HO
HN
O
O
NH
O
NH
Cl
1300
1200
1100
1000
900
800
700
600
500
400
300
200
100
0
-100
180
175
170
165
160
155
150
145
140
135
130
125
120
115
110
f1 (ppm)

IR of compound 23a

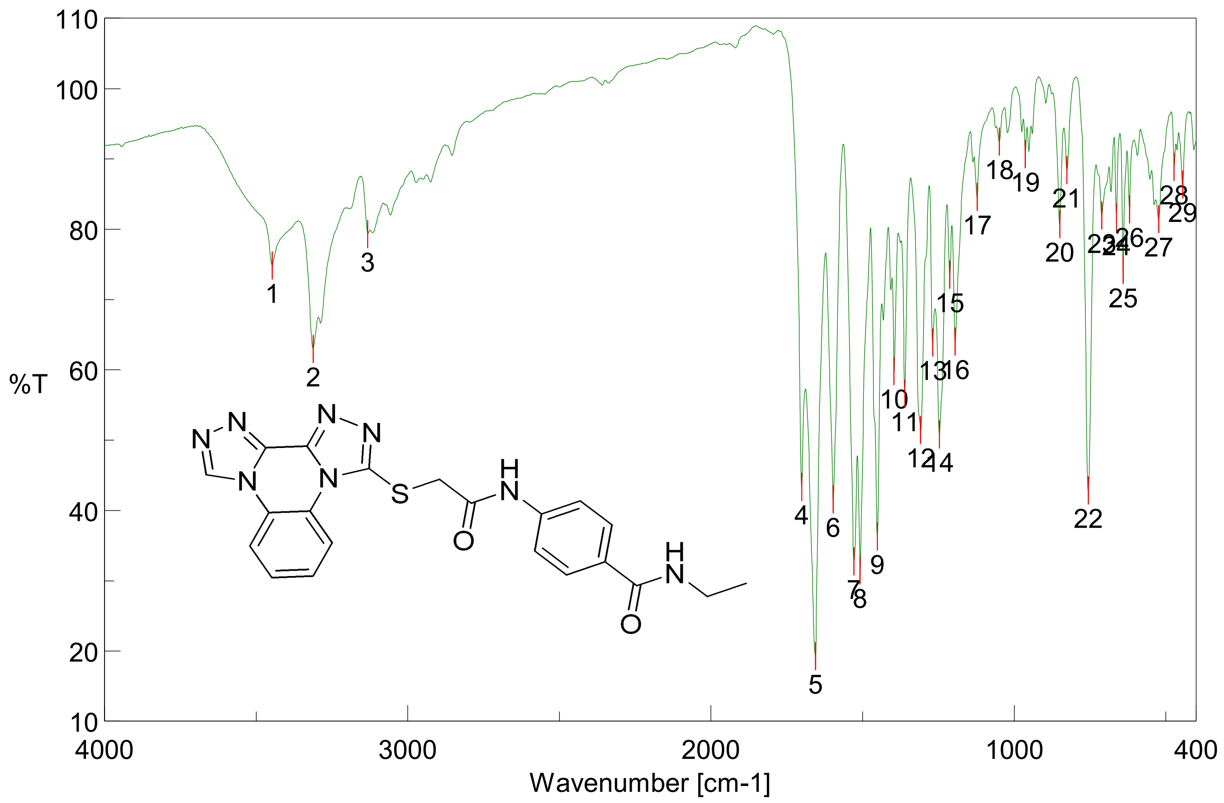

[Comments]

Sample name	F49
Comment	
User	
Division	
Company	KSU

[Detailed Information]		[Measurement Information]	
Creation date	10/29/2020 4:33 AM	Model Name	FT/IR-6600typeA
		Serial Number	A014661790
Data array type	Linear data array		
Horizontal axis	Wavenumber [cm-1]	Measurement Date	10/28/2020 1:17 AM
Vertical axis	%T		
Start	399.193 cm-1	Light Source	Standard
End	4000.6 cm-1	Detector	TGS
Data interval	0.964233 cm-1	Accumulation	Auto (16)
Data points	3736	Resolution	4 cm-1
		Zero Filling	On
		Apodization	Cosine
		Gain	Auto (2)
		Aperture	Auto (7.1 mm)
		Scanning Speed	Auto (2 mm/sec)
		Filter	Auto (10000 Hz)

[Result of Peak Picking]

No.	Position	Intensity	No.	Position	Intensity
1	3447.13	74.8372	2	3312.14	62.9582

[Result of Peak Picking]

No.	Position	Intensity	No.	Position	Intensity
3	3131.83	79.2891	4	1700.91	43.3097
5	1655.59	19.23	6	1596.77	41.5373
7	1528.31	32.7081	8	1508.06	31.4164
9	1451.17	36.2494	10	1396.21	59.7819
11	1360.53	56.5729	12	1308.46	51.4109
13	1268.93	63.8597	14	1246.75	50.7808
15	1213.01	73.4778	16	1194.69	63.974
17	1122.37	84.5537	18	1049.09	92.4428
19	963.269	90.6454	20	849.49	80.686
21	826.348	88.3693	22	755.959	42.8148
23	711.604	81.9333	24	663.393	81.599
25	641.215	74.1925	26	620.002	82.7813
27	523.579	81.399	28	472.474	88.8206
29	444.512	86.3387			

1H NMR 23a

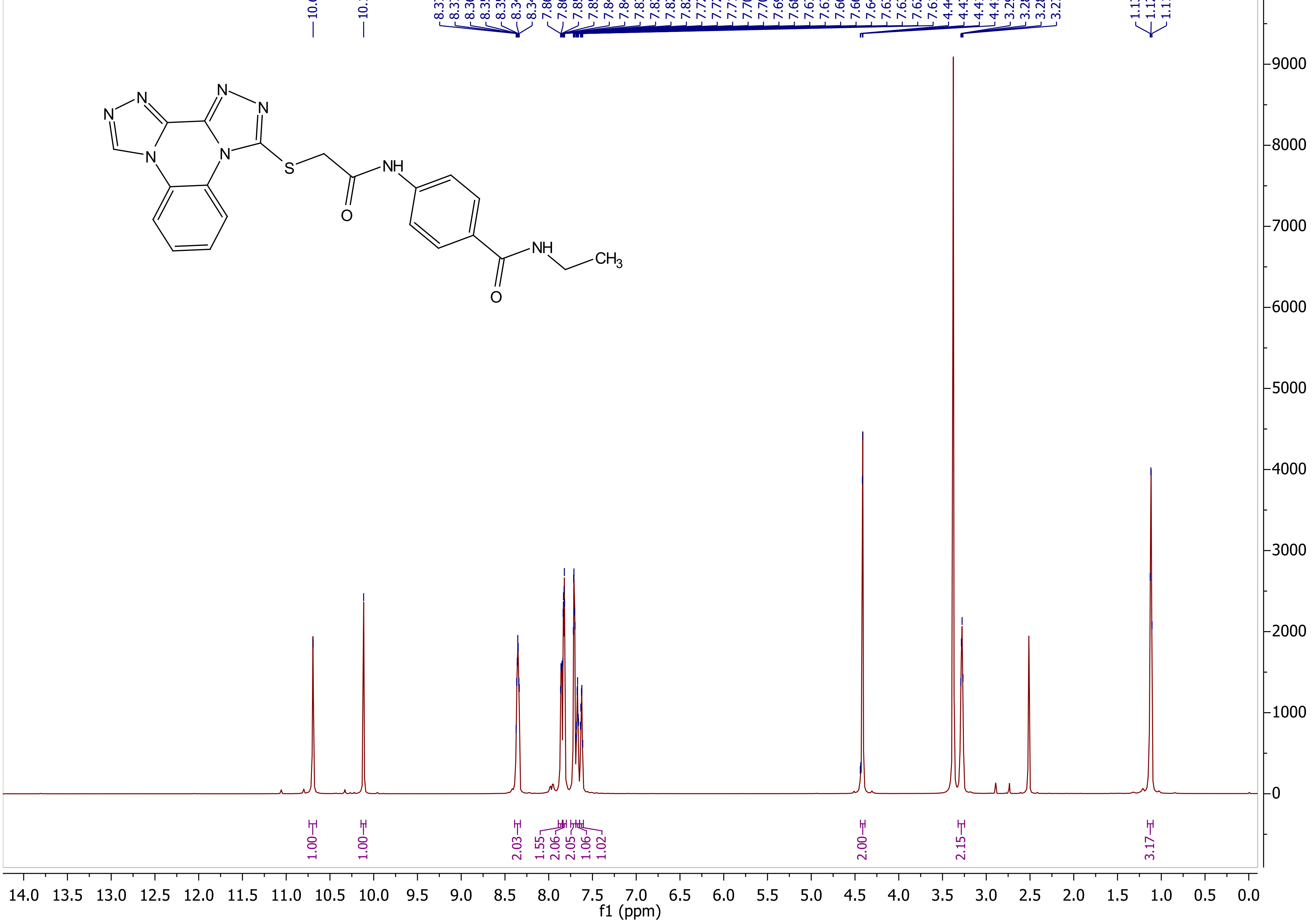

10.69
10.12
8.37 8.37 8.36 8.35 8.35 8.34 8.34
7.86 7.86 7.85 7.85 7.84 7.84 7.83 7.82 7.82 7.72 7.72 7.71 7.70 7.70 7.69 7.68 7.67 7.67 7.66 7.66 7.64 7.63 7.63 7.62 7.61
4.44 4.43 4.41 4.41
3.29 3.28 3.28 3.27
1.13 1.12 1.11
N
N
N
N
N
N
S
NH
O
NH
CH3
O
1.00
1.00
2.03
1.55
2.06
2.05
1.06
1.02
2.00
2.15
3.17
f1 (ppm)

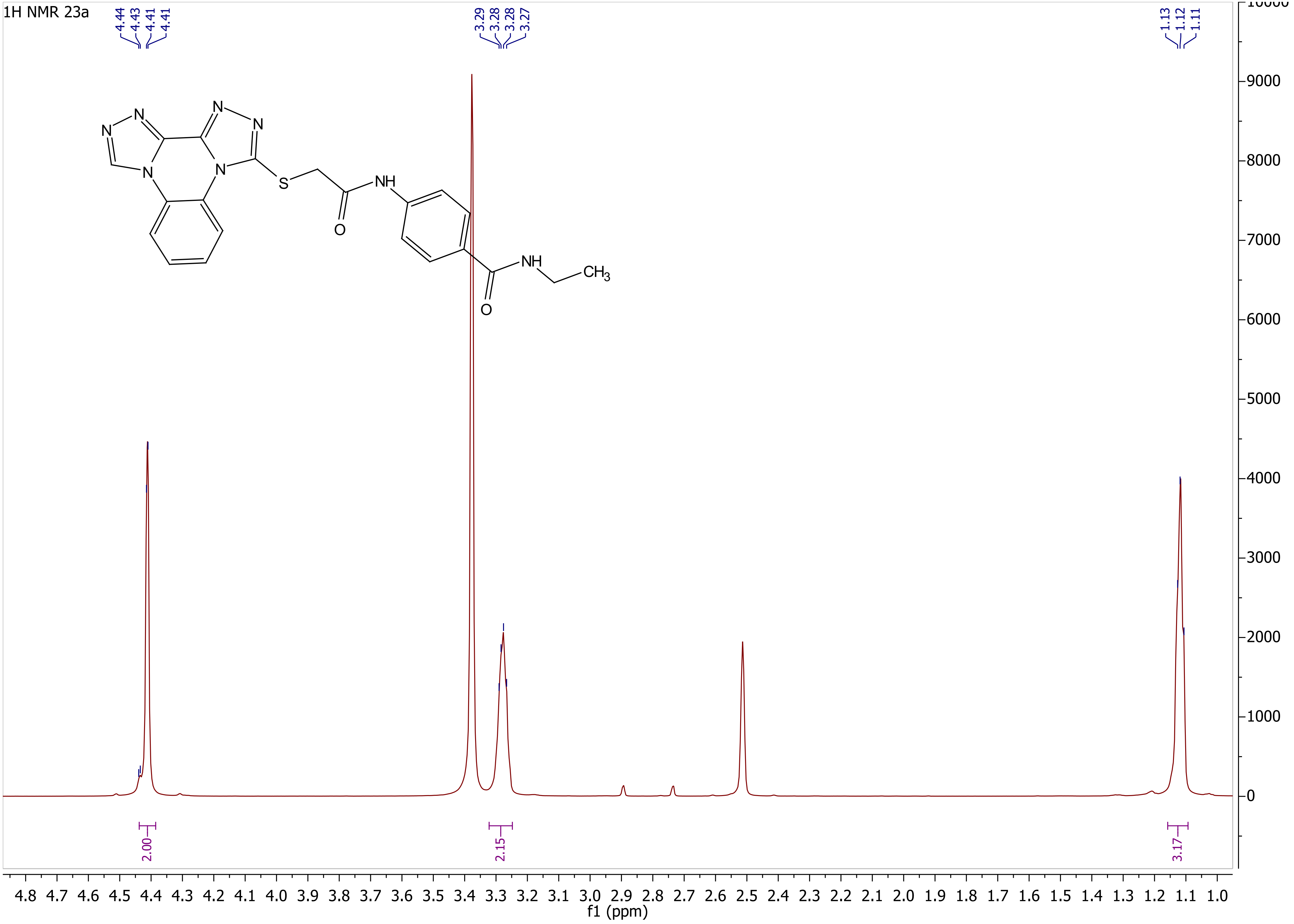

1H NMR 23a
4.44
4.43
4.41
4.41
3.29
3.28
3.28
3.27
1.13
1.12
1.11
N
N
N
N
N
N
N
S
NH
O
NH
O
CH3
2.00
2.15
3.17
f1 (ppm)

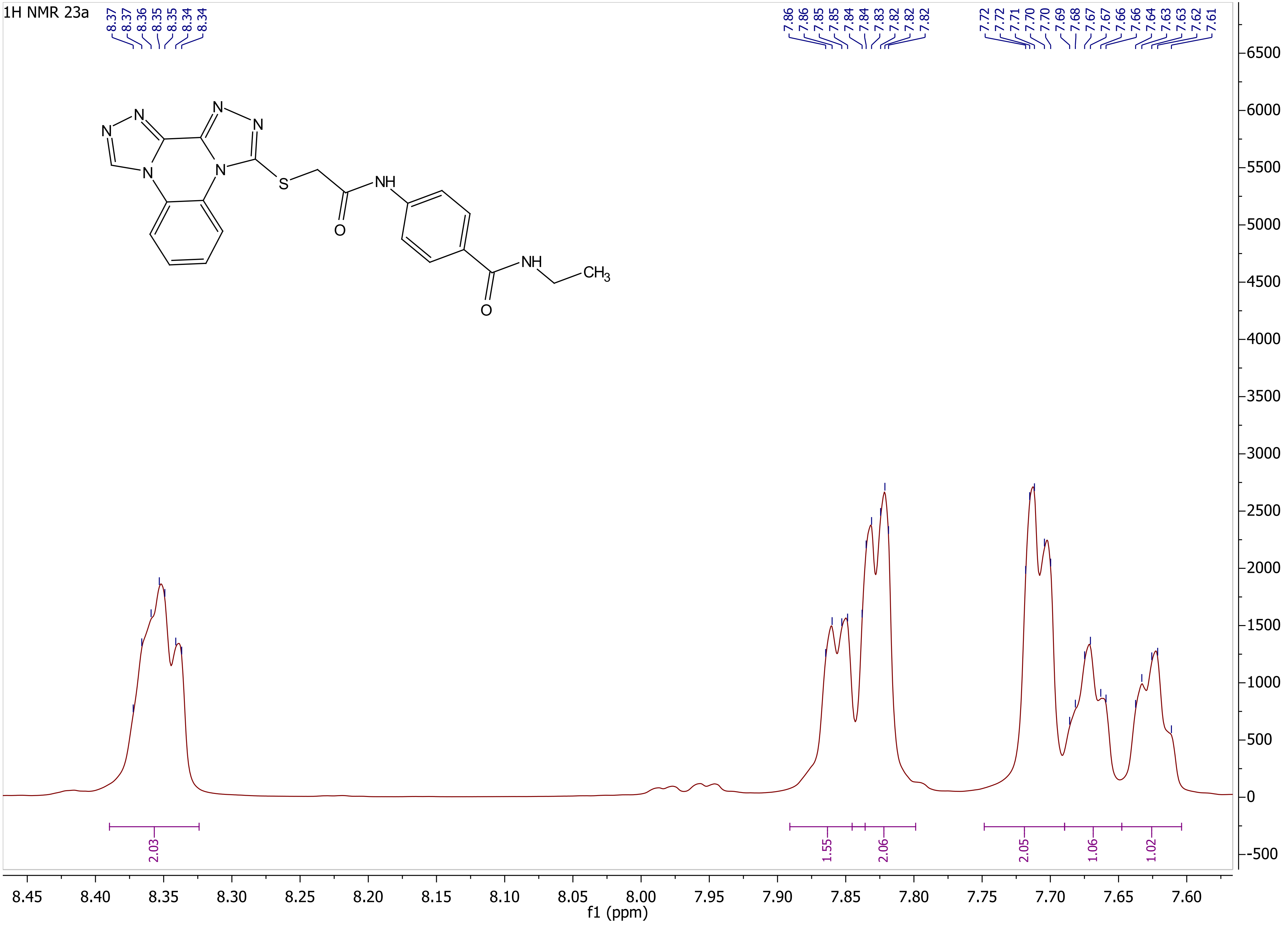

1H NMR 23a
8.37
8.37
8.36
8.35
8.35
8.34
8.34
7.86
7.86
7.85
7.85
7.84
7.84
7.83
7.82
7.82
7.82
7.72
7.72
7.71
7.70
7.70
7.69
7.68
7.67
7.67
7.66
7.66
7.64
7.63
7.63
7.62
7.61
N
N
N
N
N
N
N
N
S
NH
O
NH
O
CH3
2.03
1.55
2.06
2.05
1.06
1.02
f1 (ppm)

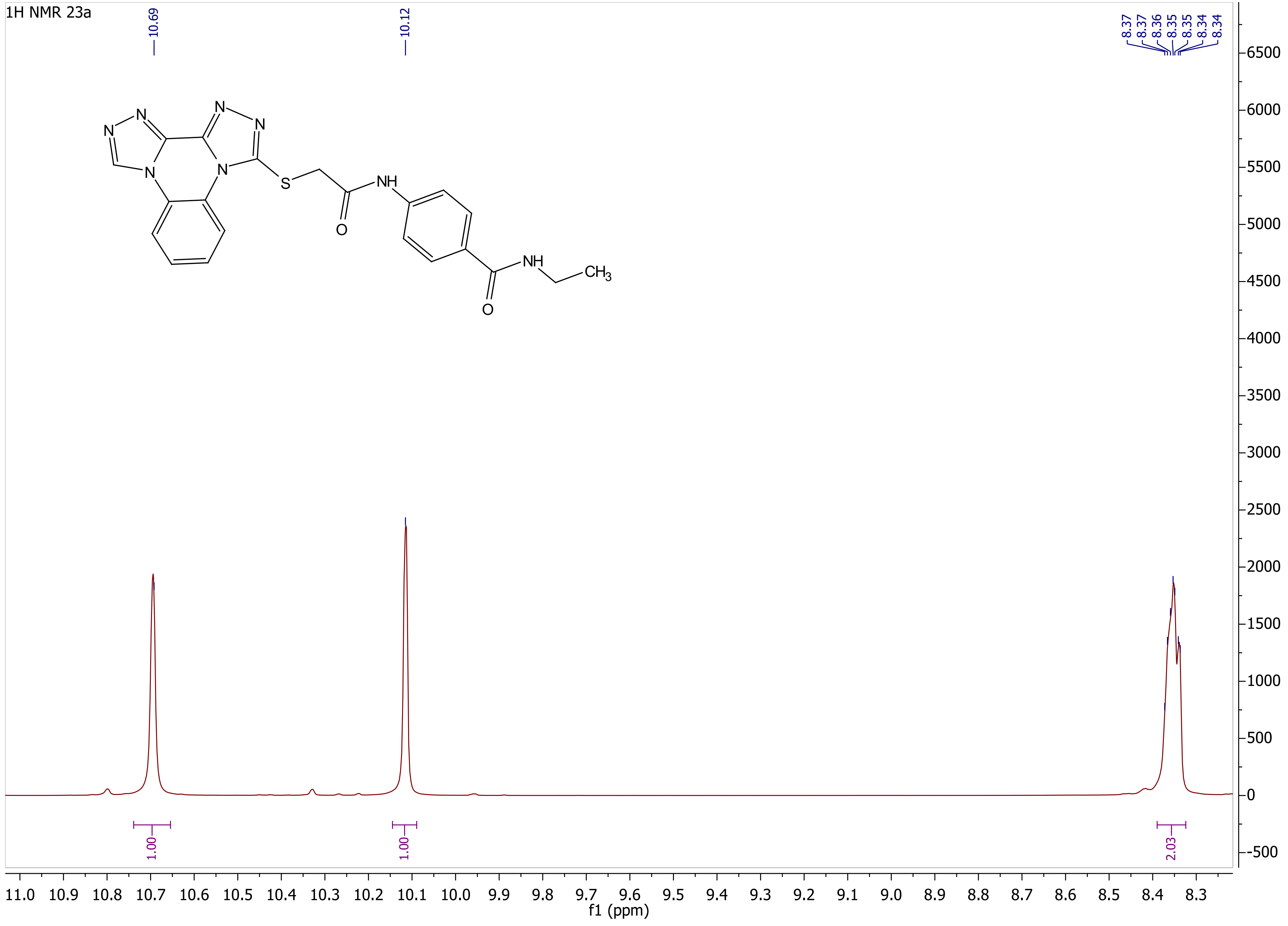

1H NMR 23a
10.69
10.12
8.37
8.37
8.36
8.35
8.35
8.34
8.34
N
N
N
N
N
N
S
NH
O
NH
CH3
O
1.00
1.00
2.03
11.0
10.9
10.8
10.7
10.6
10.5
10.4
10.3
10.2
10.1
10.0
9.9
9.8
9.7
9.6
9.5
9.4
9.3
9.2
9.1
9.0
8.9
8.8
8.7
8.6
8.5
8.4
8.3
f1 (ppm)
6500
6000
5500
5000
4500
4000
3500
3000
2500
2000
1500
1000
500
0
-500

13C NMR 23a

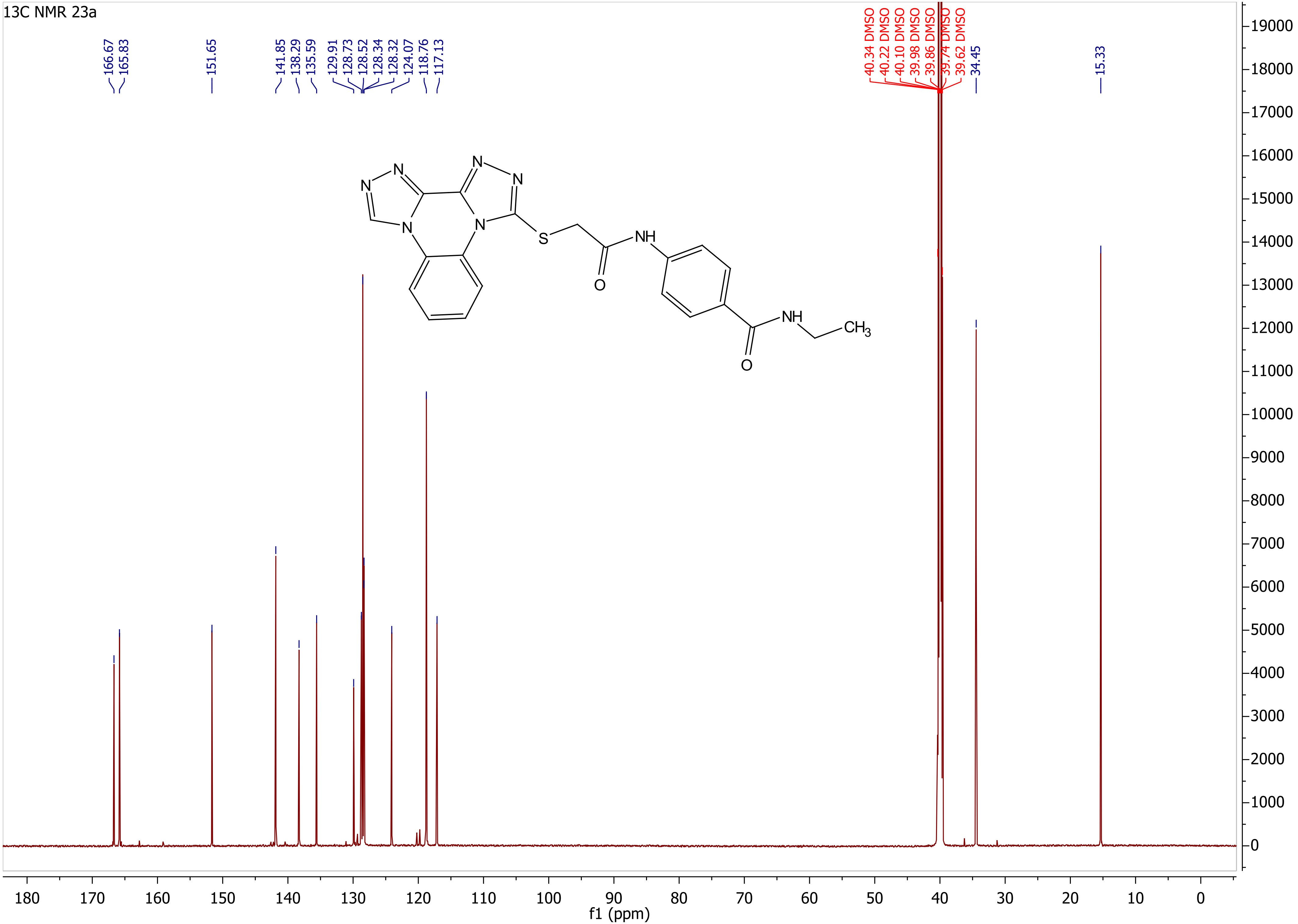

166.67
165.83
151.65
141.85
138.29
135.59
129.91
128.73
128.52
128.34
128.32
124.07
118.76
117.13
40.34 DMSO
40.22 DMSO
40.10 DMSO
39.98 DMSO
39.86 DMSO
39.74 DMSO
39.62 DMSO
34.45
15.33
N
N
N
N
N
N
S
O
NH
NH
CH3
O
f1 (ppm)

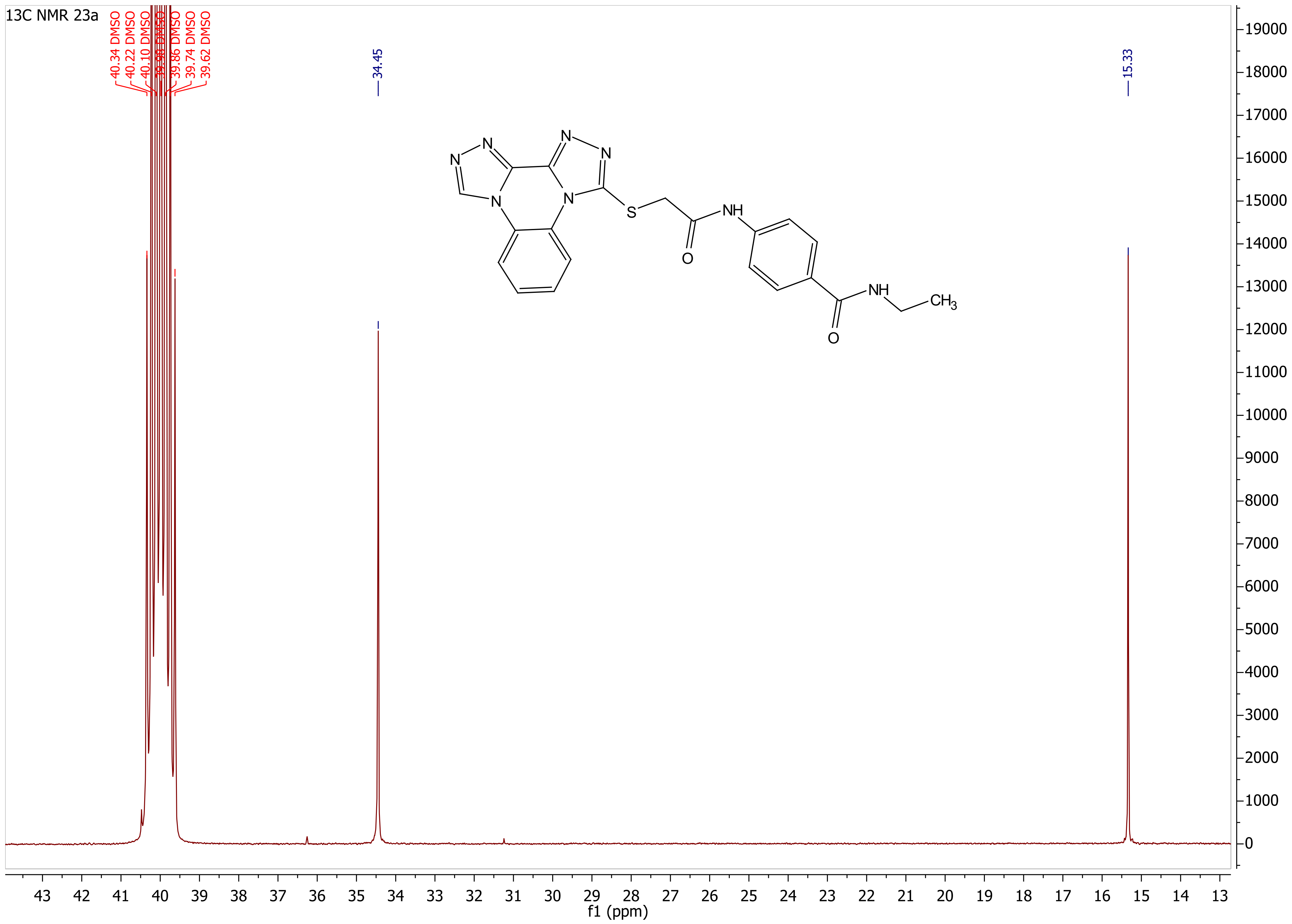
13C NMR 23a
40.34 DMSO
40.22 DMSO
40.10 DMSO
39.98 DMSO
39.86 DMSO
39.74 DMSO
39.62 DMSO
34.45
15.33
N
N
N
N
N
N
N
N
S
NH
O
NH
CH3
O
f1 (ppm)

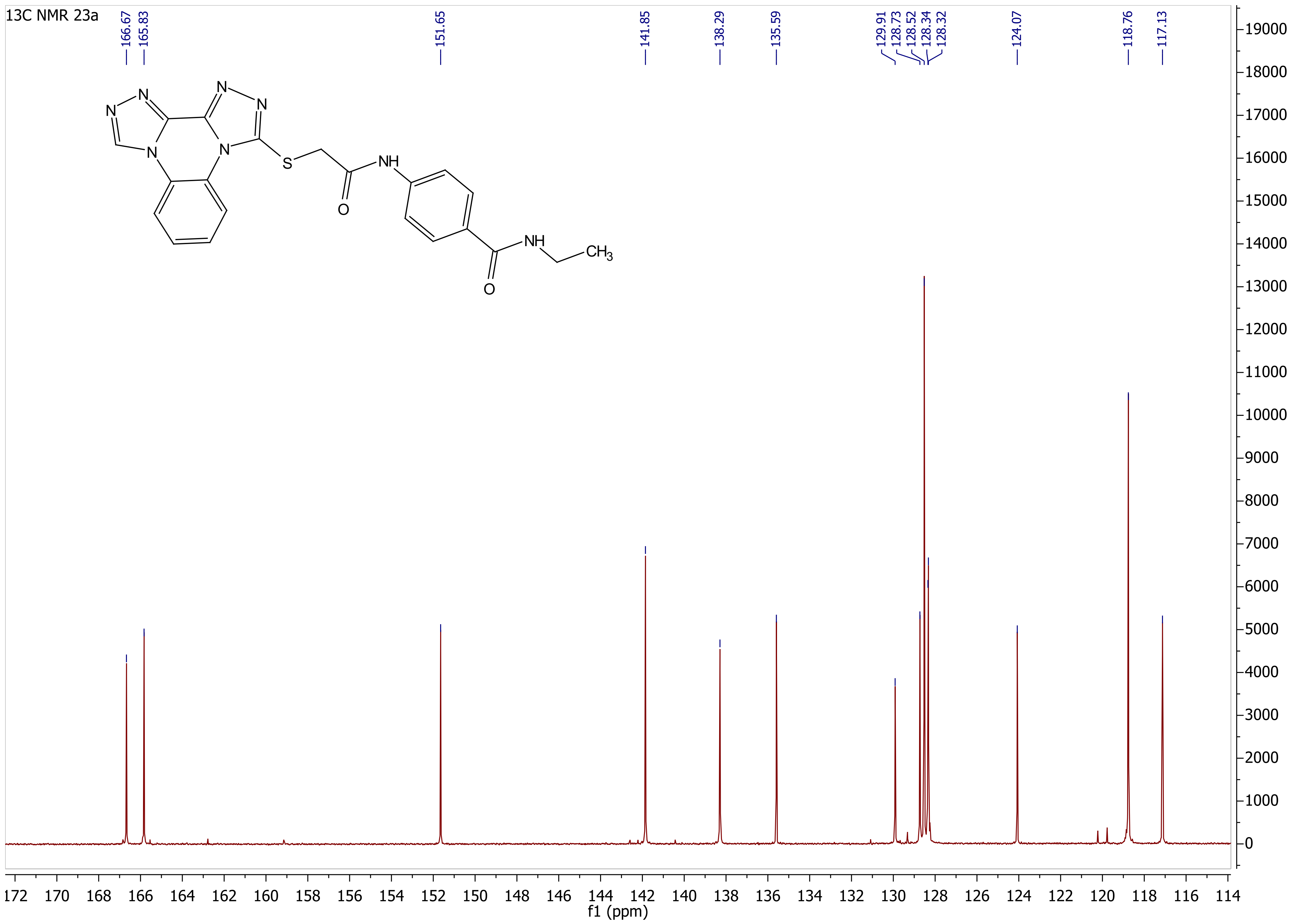

13C NMR 23a
166.67
165.83
151.65
141.85
138.29
135.59
129.91
128.73
128.52
128.34
128.32
124.07
118.76
117.13
N
N
N
N
N
N
S
NH
O
NH
CH3
O
f1 (ppm)
172 170 168 166 164 162 160 158 156 154 152 150 148 146 144 142 140 138 136 134 132 130 128 126 124 122 120 118 116 114
19000 18000 17000 16000 15000 14000 13000 12000 11000 10000 9000 8000 7000 6000 5000 4000 3000 2000 1000 0

IR of compound 23b

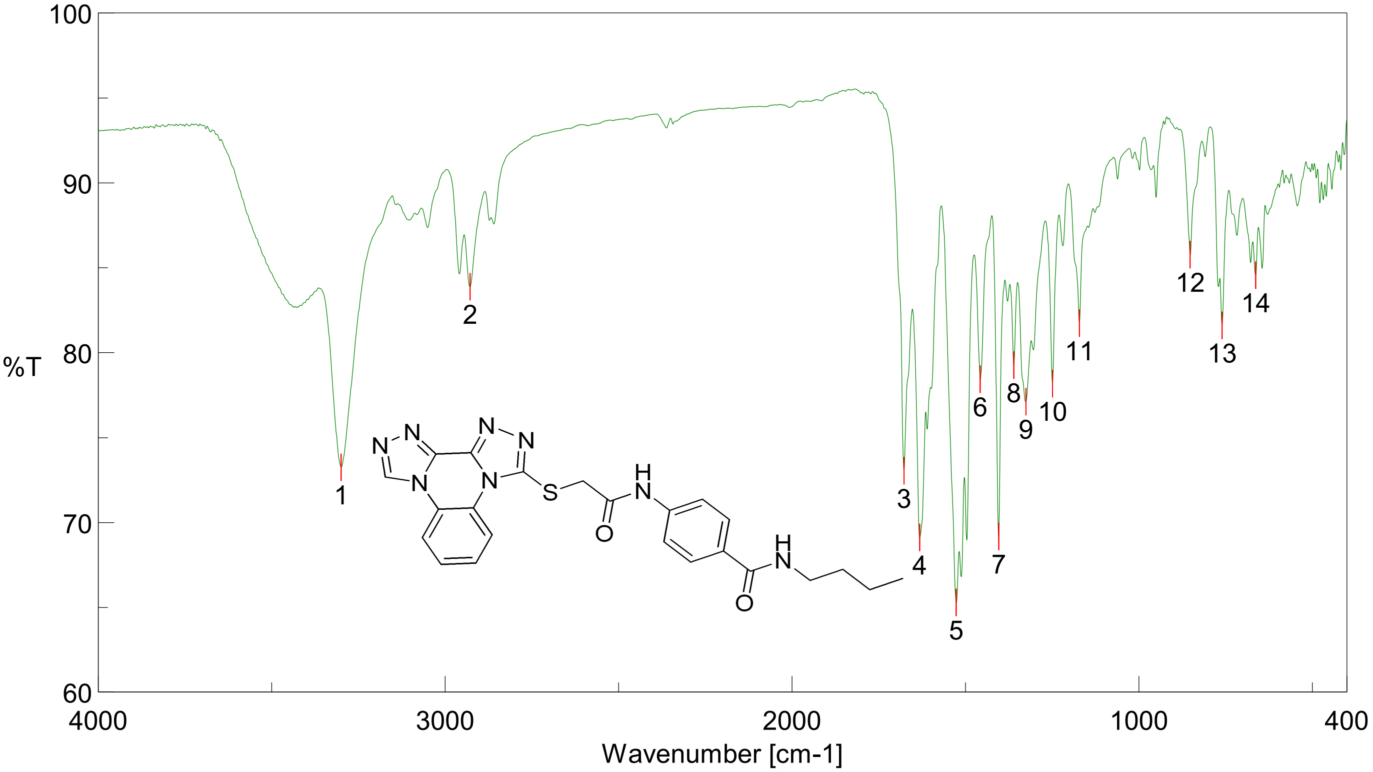

[Comments]

Sample name	F84
Comment	
User	
Division	
Company	KSU

[Detailed Information]

Creation date	10/29/2020 5:09 AM
Data array type	Linear data array
Horizontal axis	Wavenumber [cm-1]
Vertical axis	%T
Start	399.193 cm-1
End	4000.6 cm-1
Data interval	0.964233 cm-1
Data points	3736

[Measurement Information]

Model Name	FT/IR-6600typeA
Serial Number	A014661790
Measurement Date	10/28/2020 4:08 AM
Light Source	Standard
Detector	TGS
Accumulation	Auto (14)
Resolution	4 cm-1
Zero Filling	On
Apodization	Cosine
Gain	Auto (1)
Aperture	Auto (7.1 mm)
Scanning Speed	Auto (2 mm/sec)
Filter	Auto (10000 Hz)

[Result of Peak Picking]

No.	Position	Intensity	No.	Position	Intensity	No.	Position	Intensity
1	3299.61	73.2362	2	2928.38	83.8703	3	1676.8	73.035

[Result of Peak Picking]

No.	Position	Intensity	No.	Position	Intensity	No.	Position	Intensity
4	1631.48	69.101	5	1526.38	65.2801	6	1456.96	78.4328
7	1403.92	69.1576	8	1360.53	79.2458	9	1325.82	77.1072
10	1248.68	78.1692	11	1171.54	81.7284	12	852.382	85.7591
13	759.816	81.5975	14	663.393	84.5466			

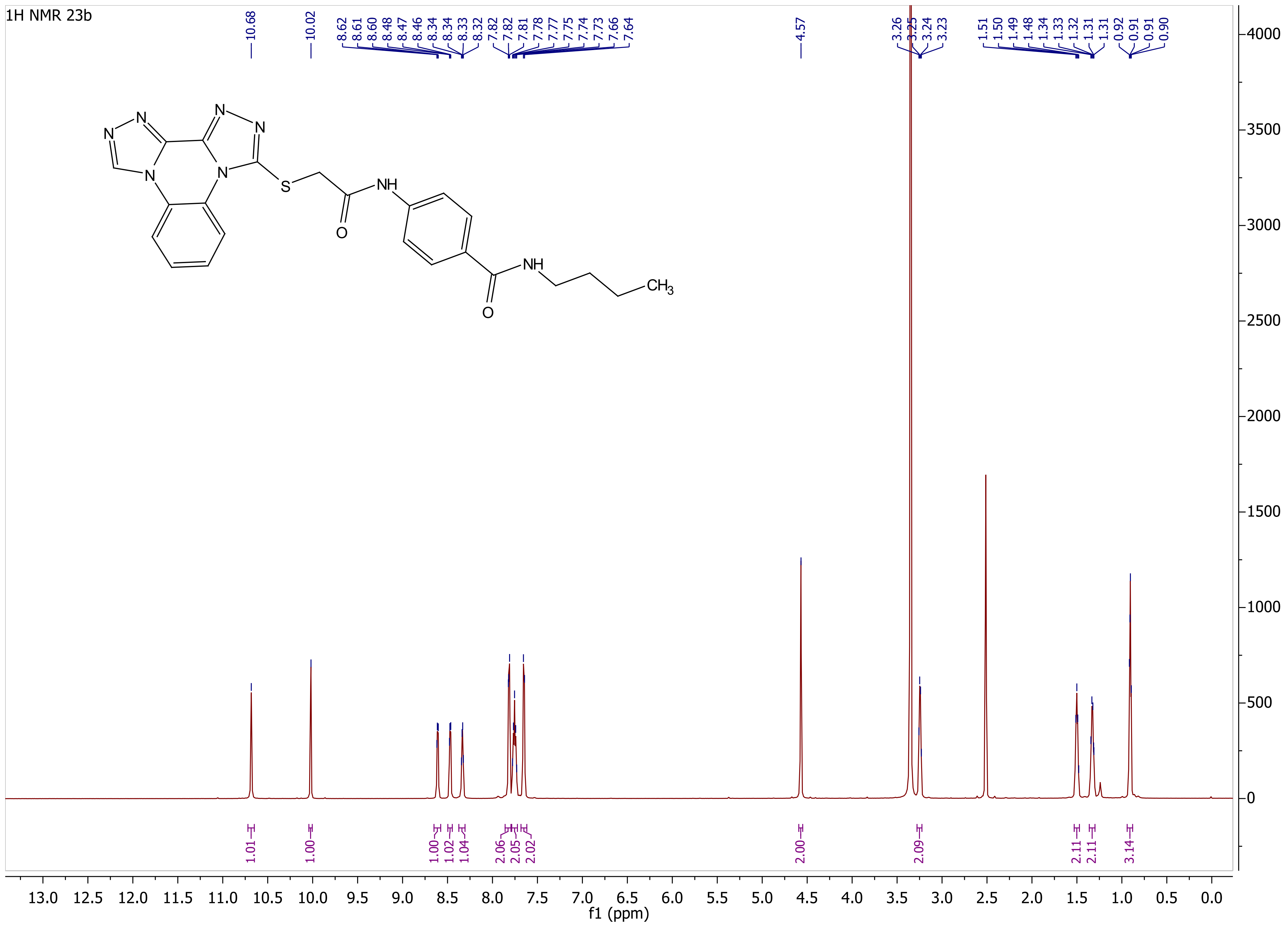

1H NMR 23b
10.68
10.02
8.62
8.61
8.60
8.48
8.47
8.46
8.34
8.34
8.33
8.32
7.82
7.82
7.81
7.78
7.77
7.75
7.74
7.73
7.66
7.64
4.57
3.26
3.25
3.24
3.23
1.51
1.50
1.49
1.48
1.34
1.33
1.32
1.31
1.31
0.92
0.91
0.91
0.90
1.01
1.00
1.00
1.02
1.04
2.06
2.05
2.02
2.00
2.09
2.11
2.11
3.14
f1 (ppm)

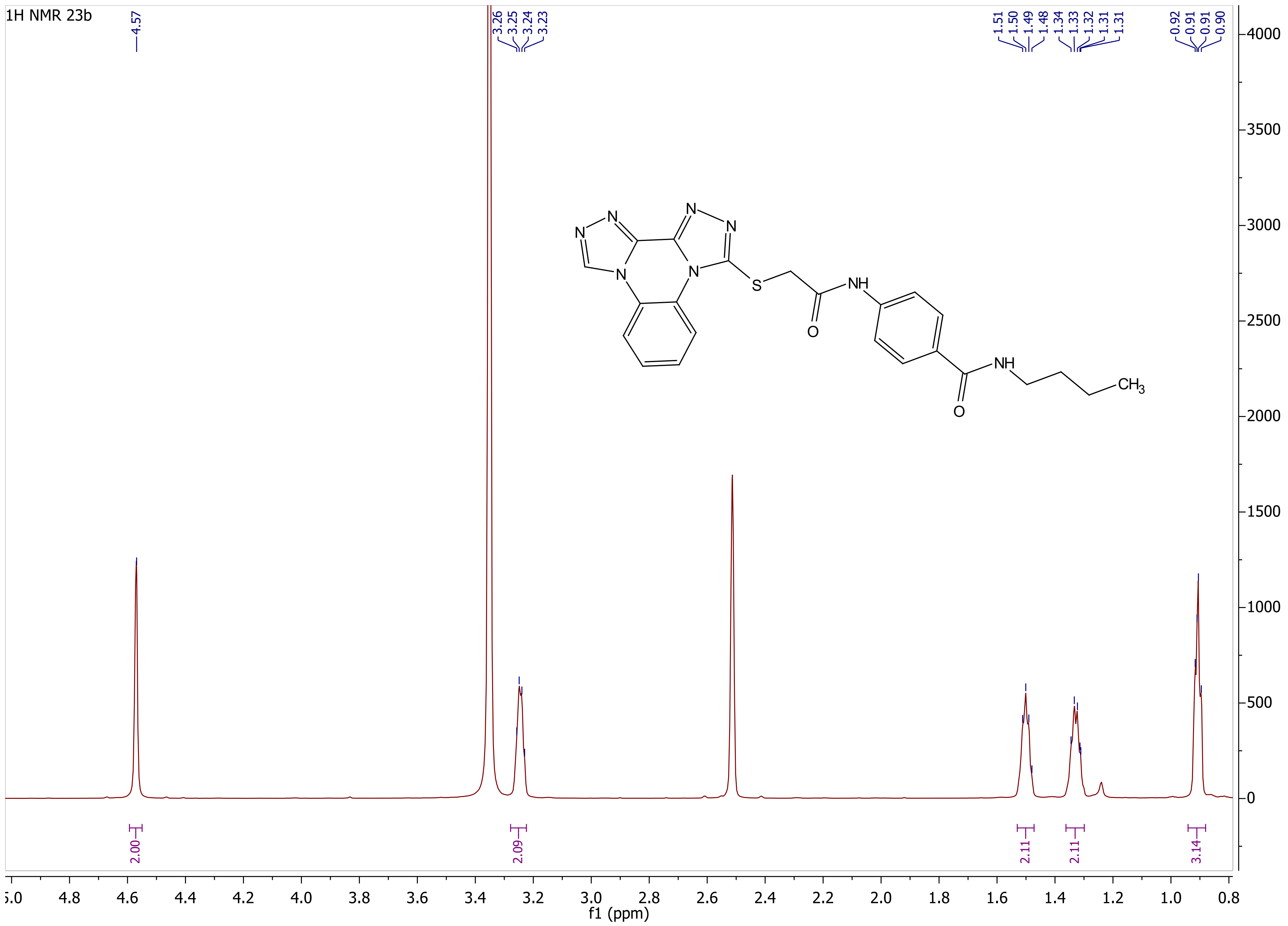

1H NMR 23b
4.57
3.26
3.25
3.24
3.23
1.51
1.50
1.49
1.48
1.34
1.33
1.32
1.31
1.31
0.92
0.91
0.91
0.90
2.00
2.09
2.11
2.11
3.14
f1 (ppm)

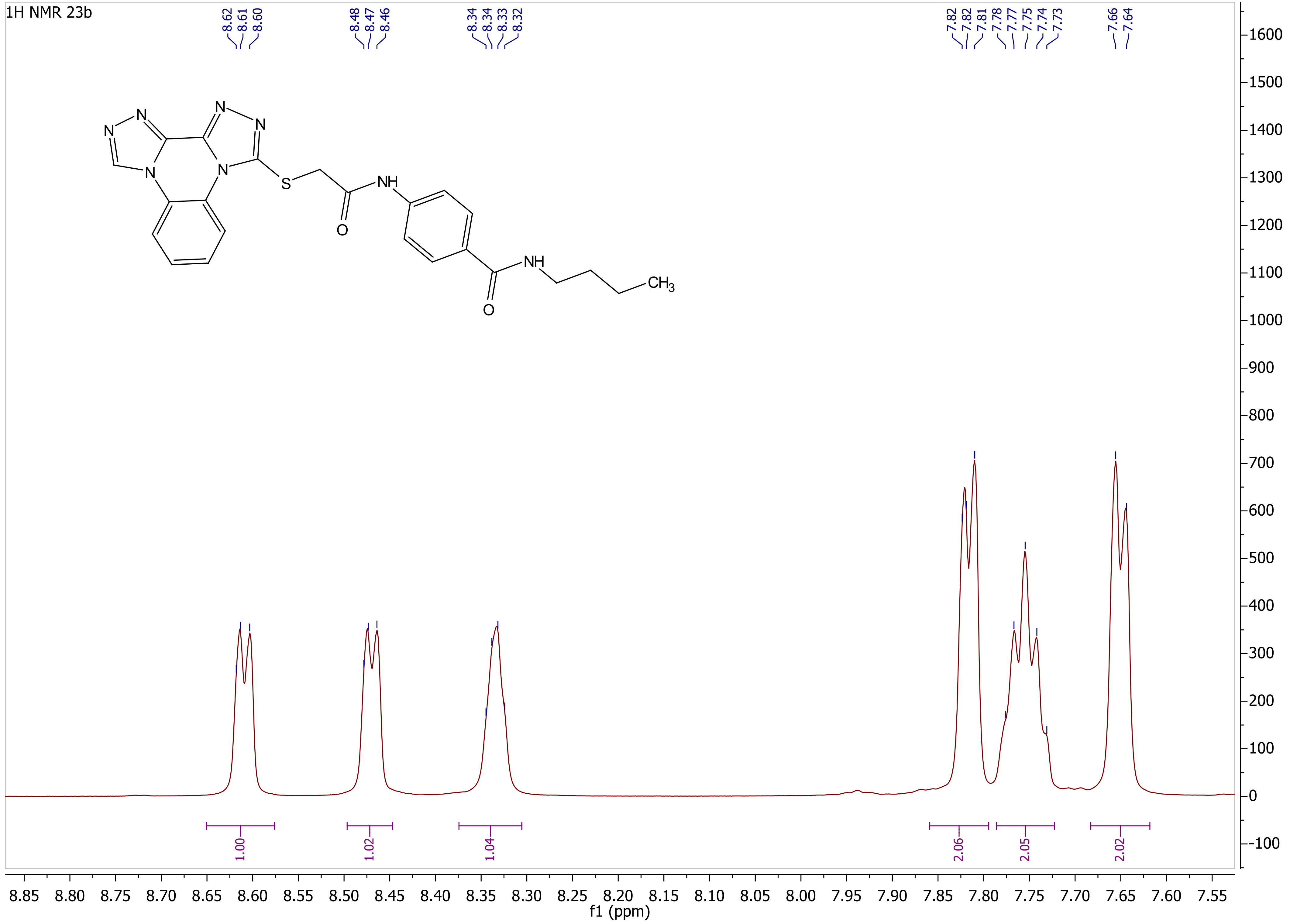

1H NMR 23b
8.62
8.61
8.60
8.48
8.47
8.46
8.34
8.34
8.33
8.32
7.82
7.82
7.81
7.78
7.77
7.75
7.74
7.73
7.66
7.64
N
N
N
N
N
N
S
NH
O
NH
O
CH3
1.00
1.02
1.04
2.06
2.05
2.02
f1 (ppm)

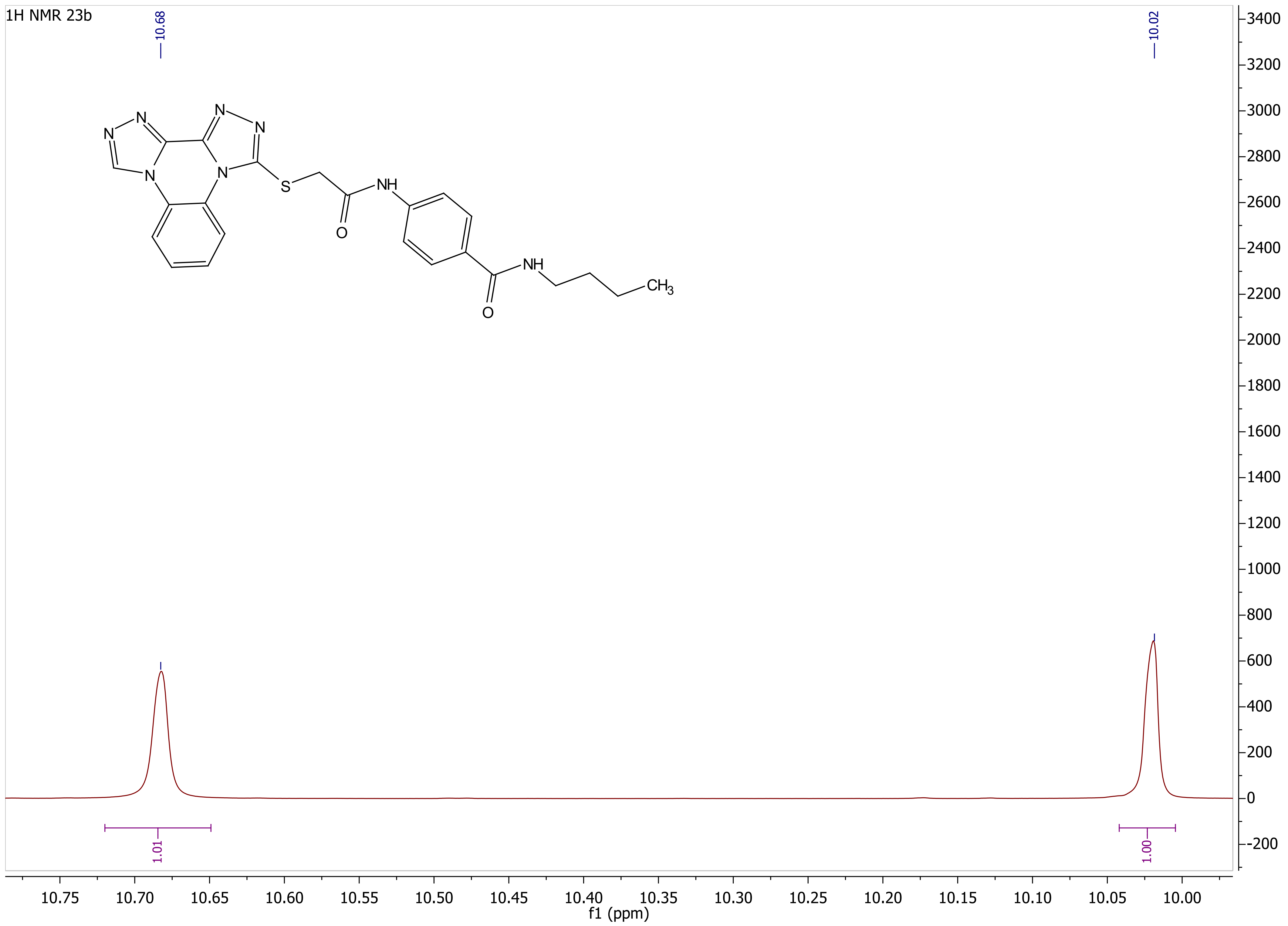

1H NMR 23b
10.68
10.02
N
N
N
N
N
N
S
NH
O
NH
O
CH3
1.01
1.00
f1 (ppm)

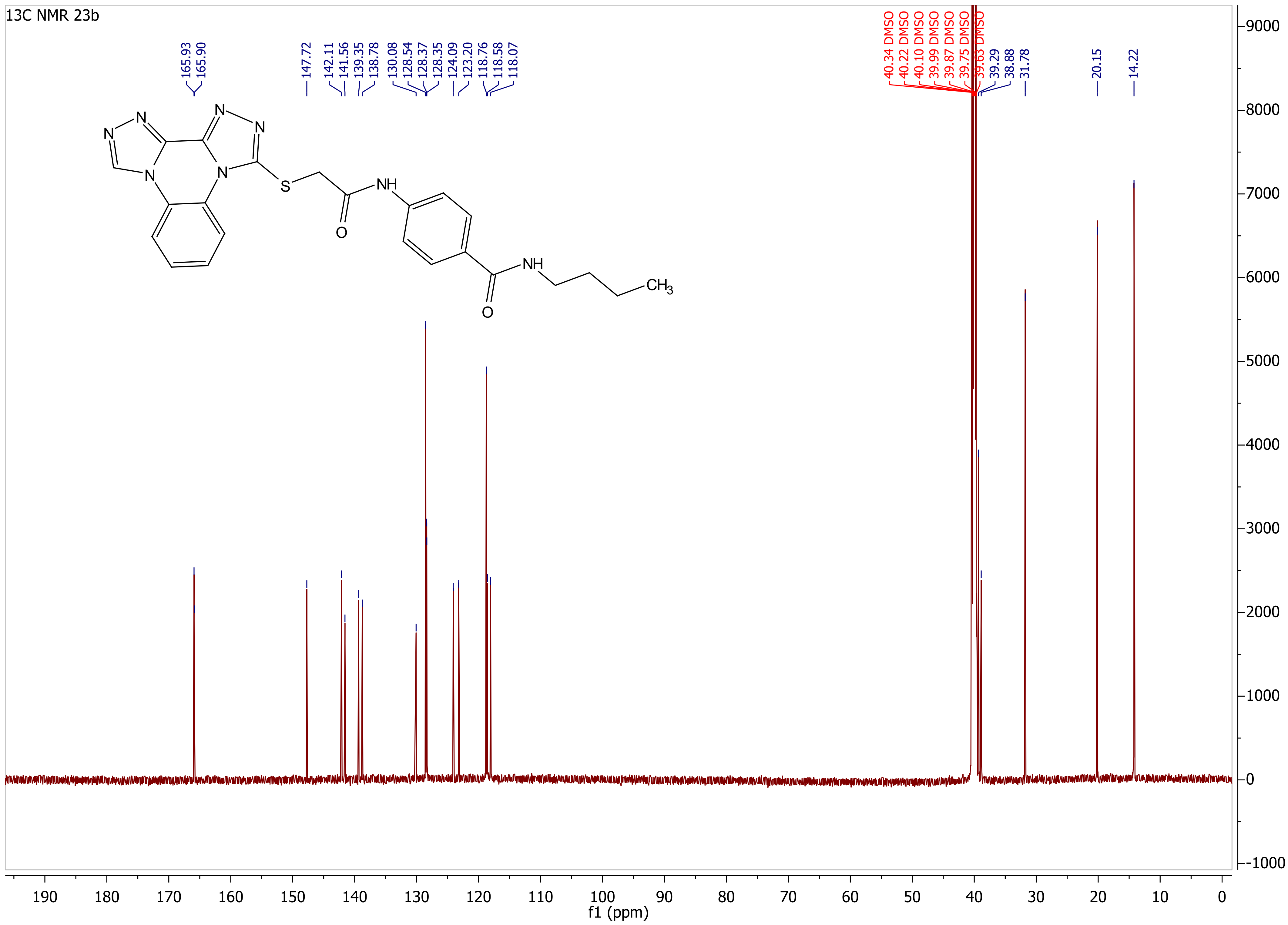

13C NMR 23b
165.93
165.90
147.72
142.11
141.56
139.35
138.78
130.08
128.54
128.37
128.35
124.09
123.20
118.76
118.58
118.07
40.34 DMSO
40.22 DMSO
40.10 DMSO
39.99 DMSO
39.87 DMSO
39.75 DMSO
39.63 DMSO
39.29
38.88
31.78
20.15
14.22
N
N
N
N
N
N
S
O
NH
NH
O
CH3
f1 (ppm)

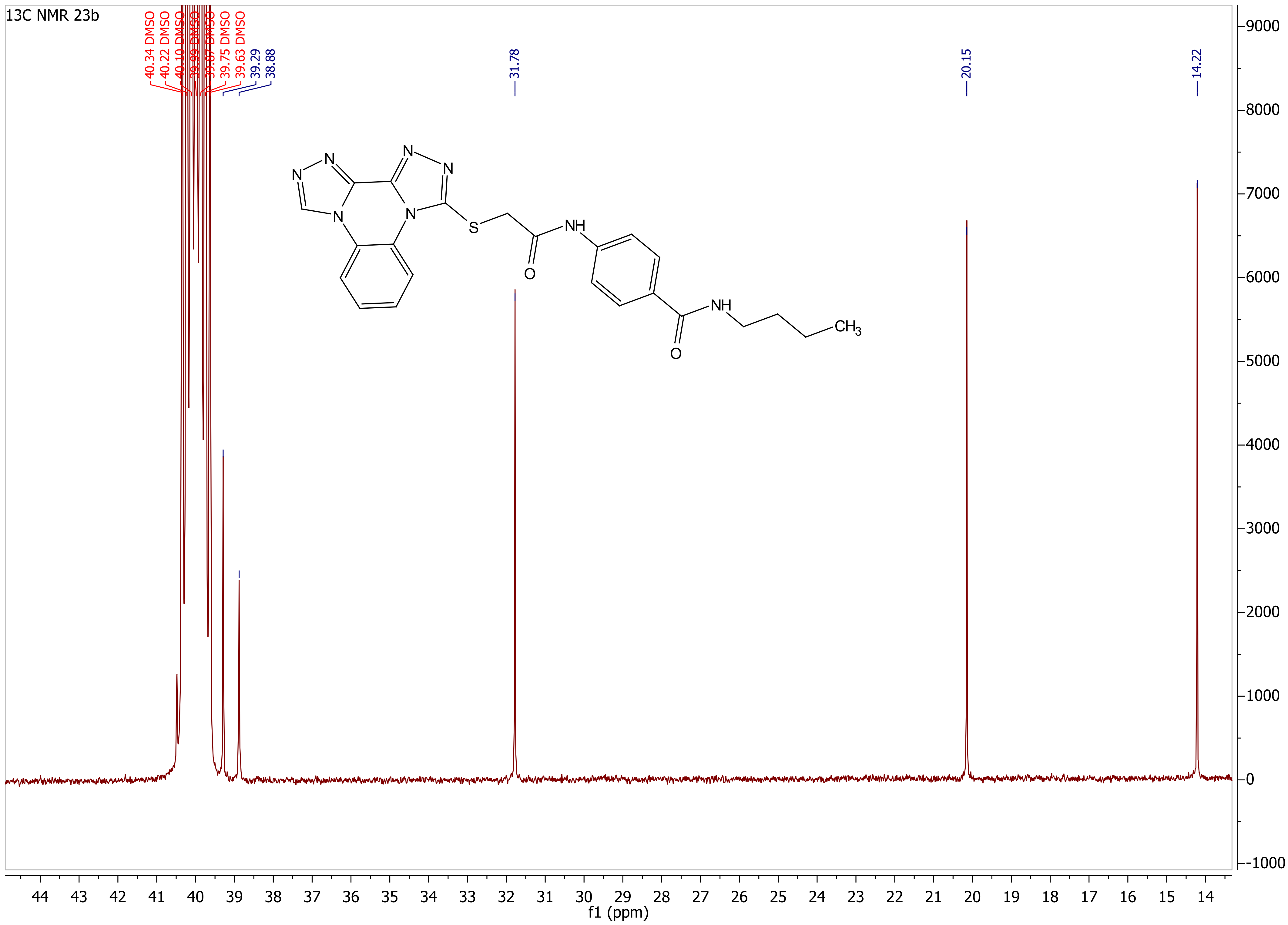

13C NMR 23b
40.34 DMSO
40.22 DMSO
40.10 DMSO
39.99 DMSO
39.87 DMSO
39.75 DMSO
39.63 DMSO
39.29
38.88
31.78
20.15
14.22
N
N
N
N
N
N
N
N
S
NH
O
NH
O
CH3
f1 (ppm)

13C NMR 23b

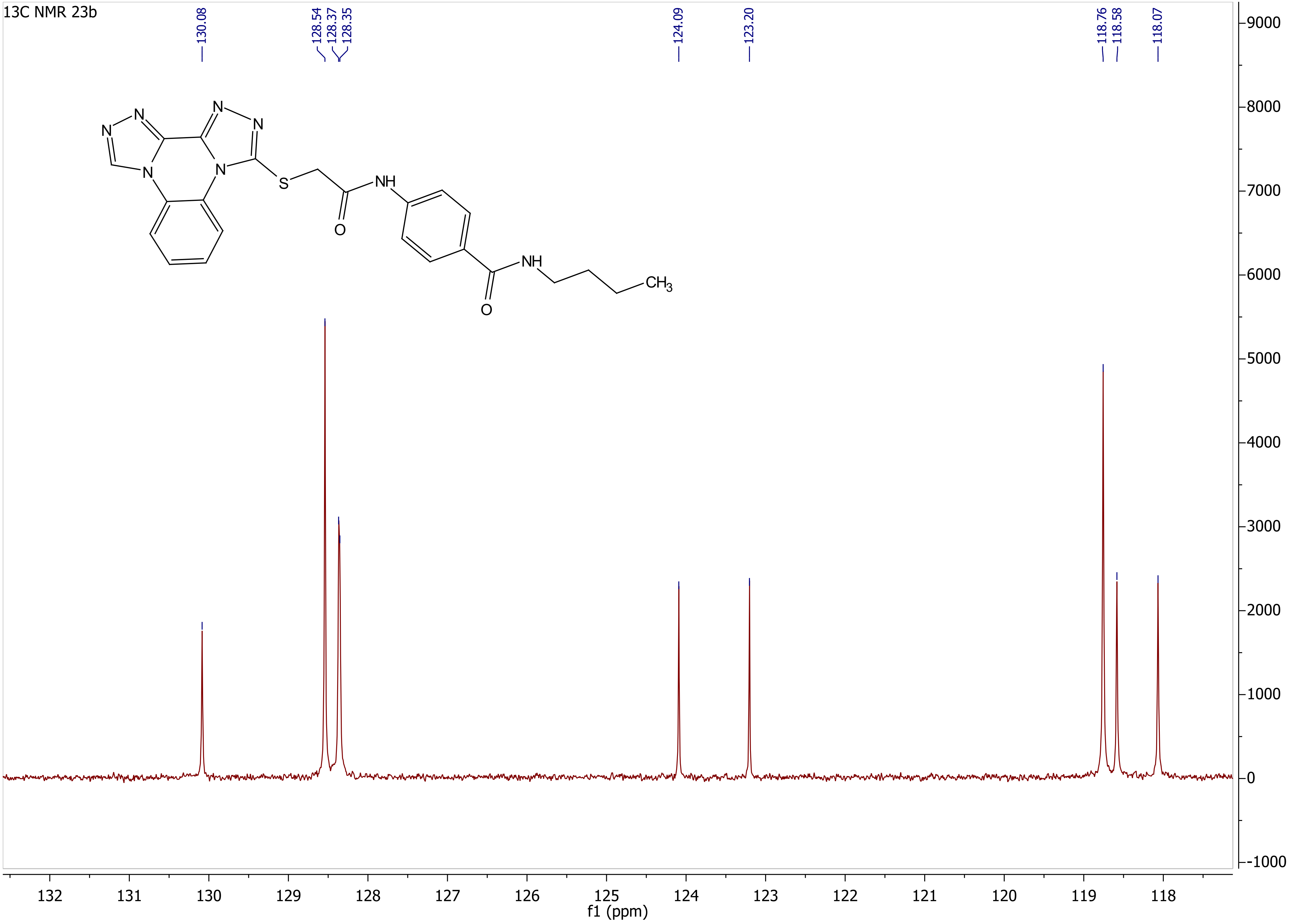

130.08
128.54
128.37
128.35
124.09
123.20
118.76
118.58
118.07
9000
8000
7000
6000
5000
4000
3000
2000
1000
0
-1000
132
131
130
129
128
127
126
125
124
123
122
121
120
119
118
f1 (ppm)
NH
NH
S
O
O
CH3
N

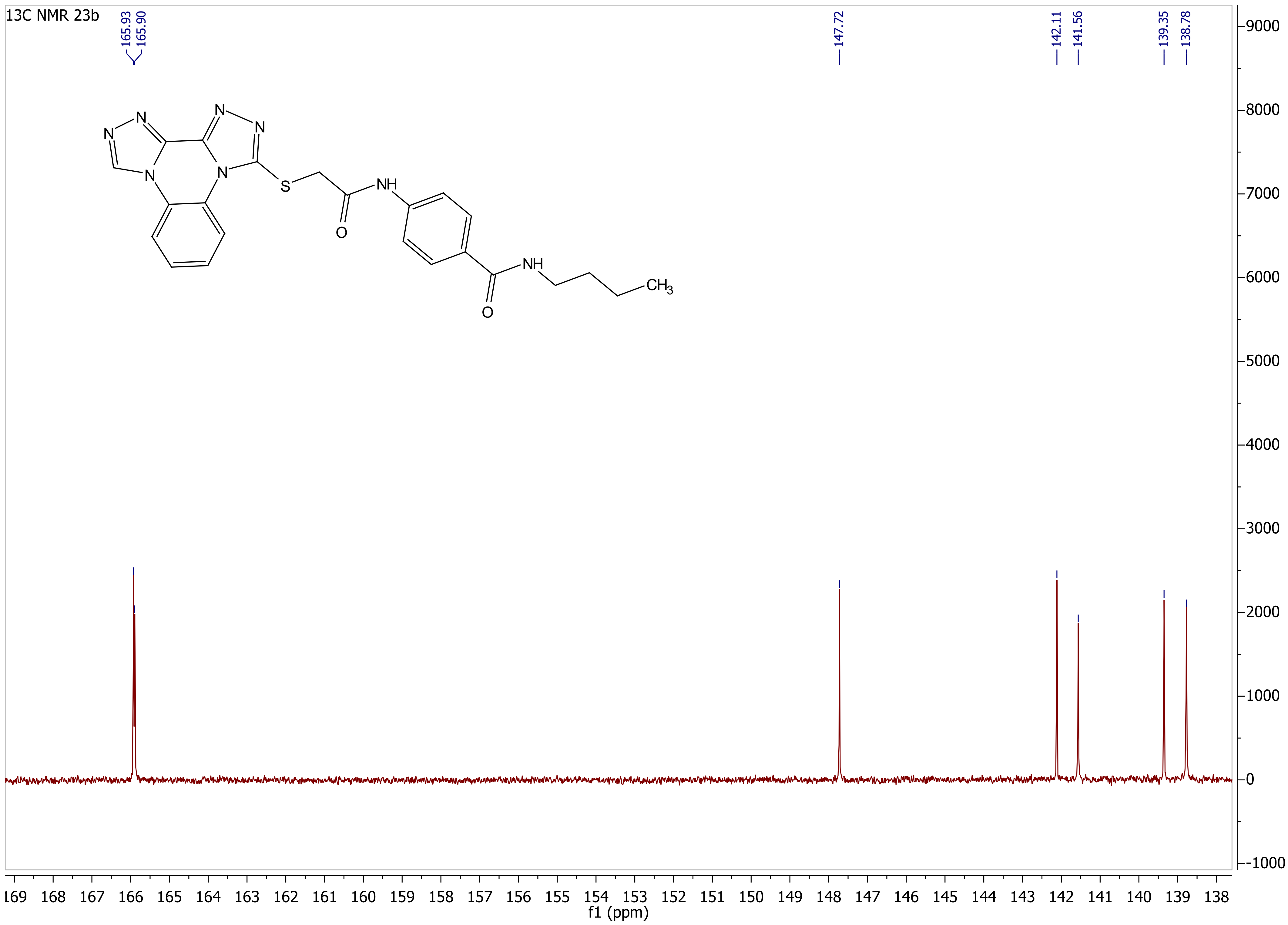
13C NMR 23b
165.93
165.90
147.72
142.11
141.56
139.35
138.78
N
NH
S
O
CH3
9000
8000
7000
6000
5000
4000
3000
2000
1000
0
-1000
169 168 167 166 165 164 163 162 161 160 159 158 157 156 155 154 153 152 151 150 149 148 147 146 145 144 143 142 141 140 139 138
f1 (ppm)

IR of compound 23c

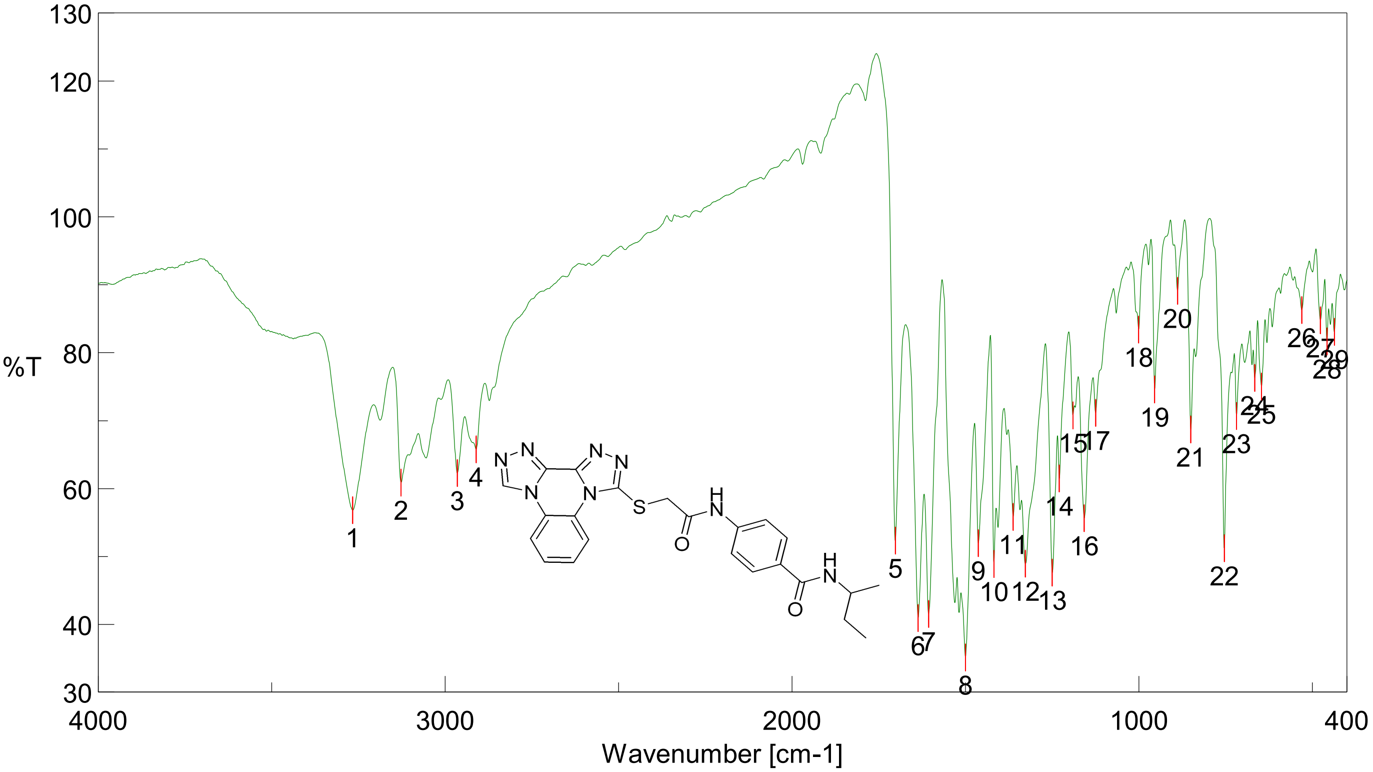

[Comments]
Sample name F99
Comment
User
Division
Company KSU

[Detailed Information]		[Measurement Information]	
Creation date	10/29/2020 5:15 AM	Model Name	FT/IR-6600typeA
		Serial Number	A014661790
Data array type	Linear data array		
Horizontal axis	Wavenumber [cm-1]	Measurement Date	10/28/2020 4:39 AM
Vertical axis	%T		
Start	399.193 cm-1	Light Source	Standard
End	4000.6 cm-1	Detector	TGS
Data interval	0.964233 cm-1	Accumulation	Auto (21)
Data points	3736	Resolution	4 cm-1
		Zero Filling	On
		Apodization	Cosine
		Gain	Auto (2)
		Aperture	Auto (7.1 mm)
		Scanning Speed	Auto (2 mm/sec)
		Filter	Auto (10000 Hz)

[Result of Peak Picking]

No.	Position	Intensity	No.	Position	Intensity	No.	Position	Intensity
1	3266.82	56.7707	2	3127.01	60.8552	3	2965.02	62.2262

[Result of Peak Picking]

No.	Position	Intensity	No.	Position	Intensity	No.	Position	Intensity
4	2910.06	65.7183	5	1701.87	52.2917	6	1636.3	40.8883
7	1605.45	41.511	8	1499.38	35.0862	9	1462.74	51.9247
10	1417.42	48.8758	11	1362.46	55.7806	12	1326.79	48.9049
13	1249.65	47.5751	14	1229.4	61.4307	15	1189.86	70.7456
16	1157.08	55.591	17	1124.3	71.1346	18	1000.87	83.3874
19	954.591	74.5562	20	888.059	89.0945	21	850.454	68.6752
22	753.066	51.1898	23	718.354	70.6287	24	665.321	76.2321
25	646.036	75.0016	26	530.328	86.2689	27	476.331	84.7175
28	457.047	81.6302	29	435.834	83.0401			

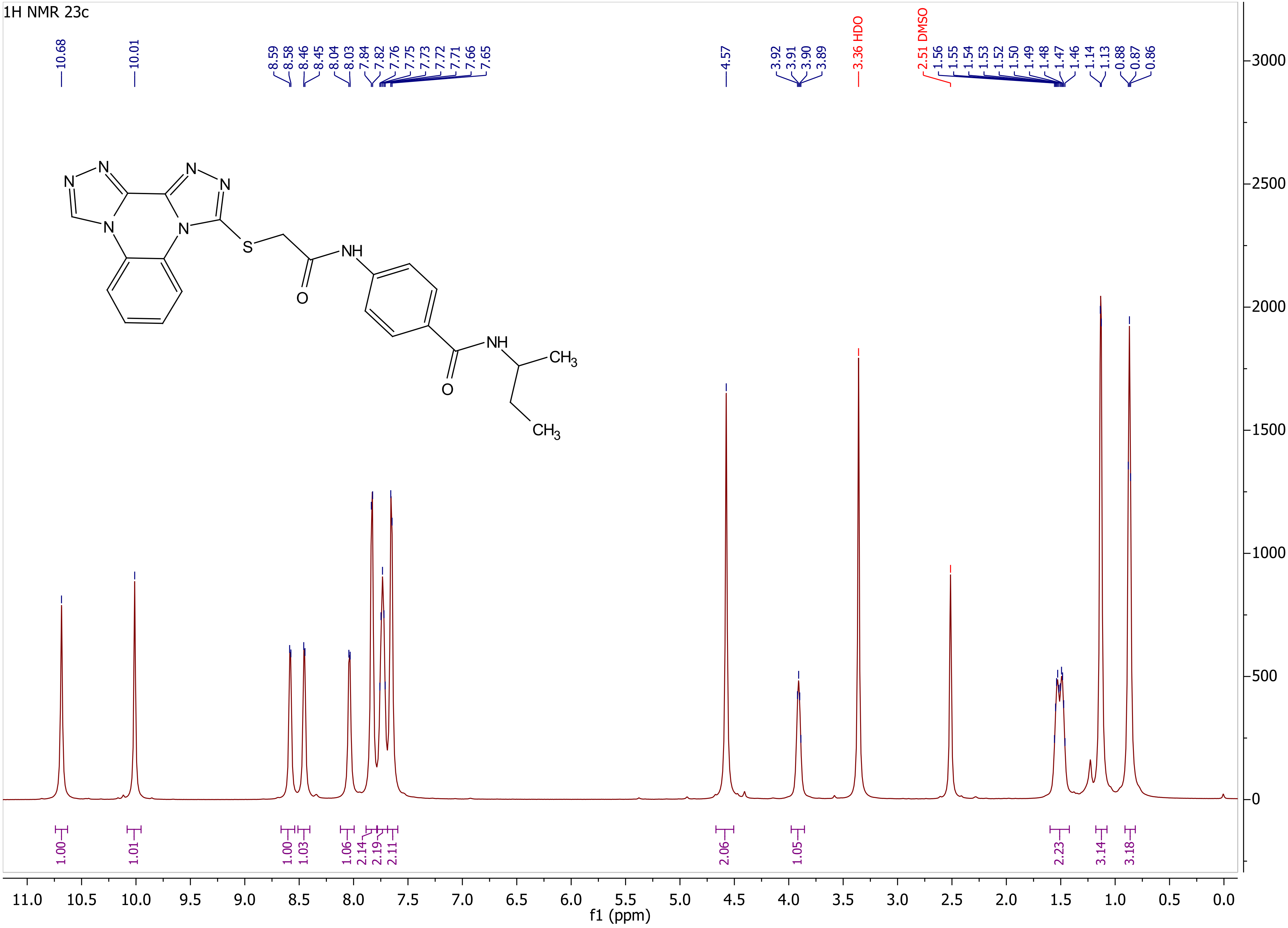
1H NMR 23c
10.68
10.01
8.59
8.58
8.46
8.45
8.04
8.03
7.84
7.82
7.76
7.75
7.73
7.72
7.71
7.66
7.65
4.57
3.92
3.91
3.90
3.89
3.36 HDO
2.51 DMSO
1.56
1.55
1.54
1.53
1.52
1.50
1.49
1.48
1.47
1.46
1.14
1.13
0.88
0.87
0.86
N
N
N
N
N
N
S
NH
O
NH
CH3
O
CH3
1.00
1.01
1.00
1.03
1.06
2.14
2.19
2.11
2.06
1.05
2.23
3.14
3.18
f1 (ppm)

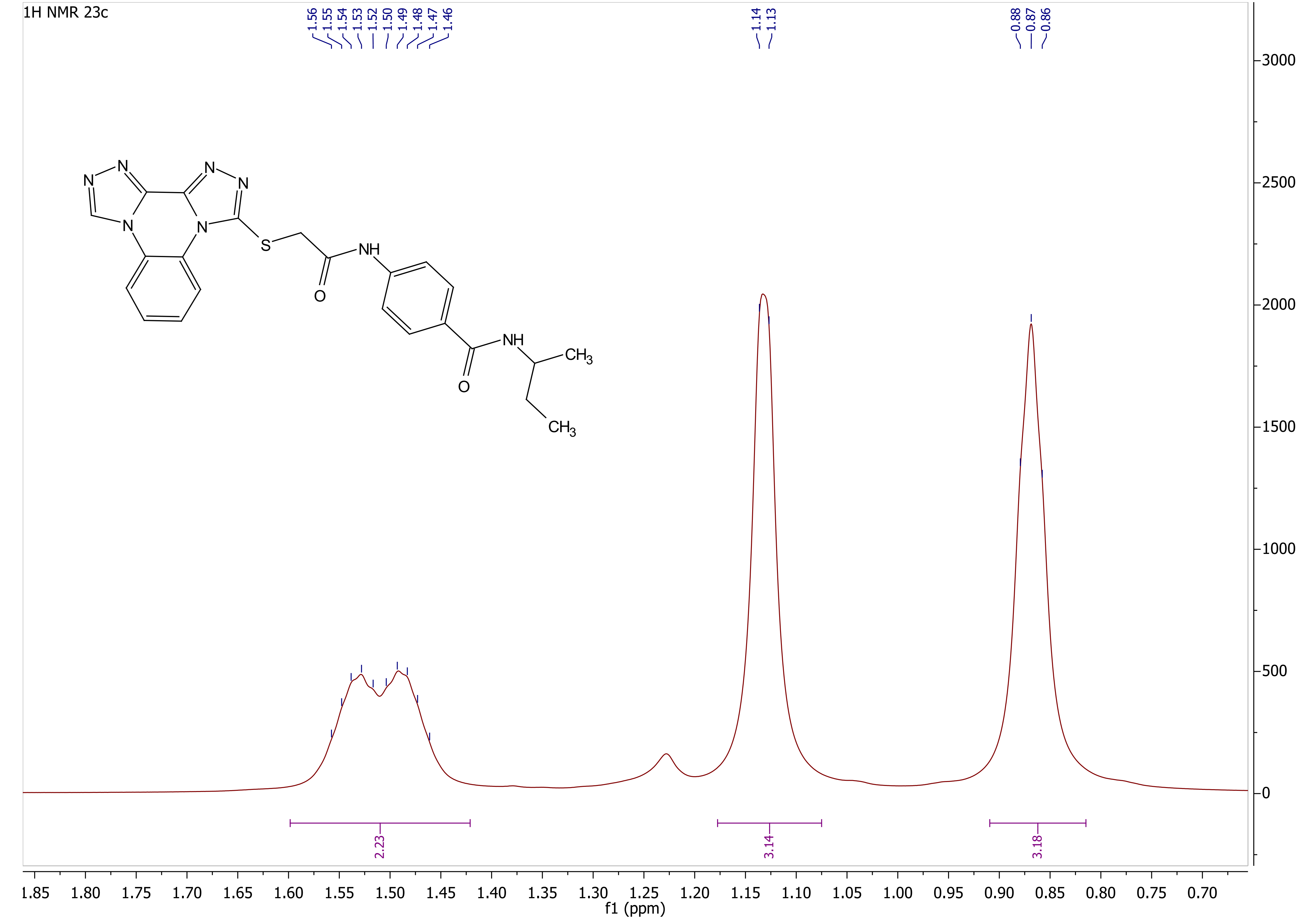
1H NMR 23c
1.56
1.55
1.54
1.53
1.52
1.50
1.49
1.48
1.47
1.46
1.14
1.13
0.88
0.87
0.86
N
N
N
N
N
N
S
NH
O
NH
O
CH3
CH3
2.23
3.14
3.18
f1 (ppm)

1H NMR 23c

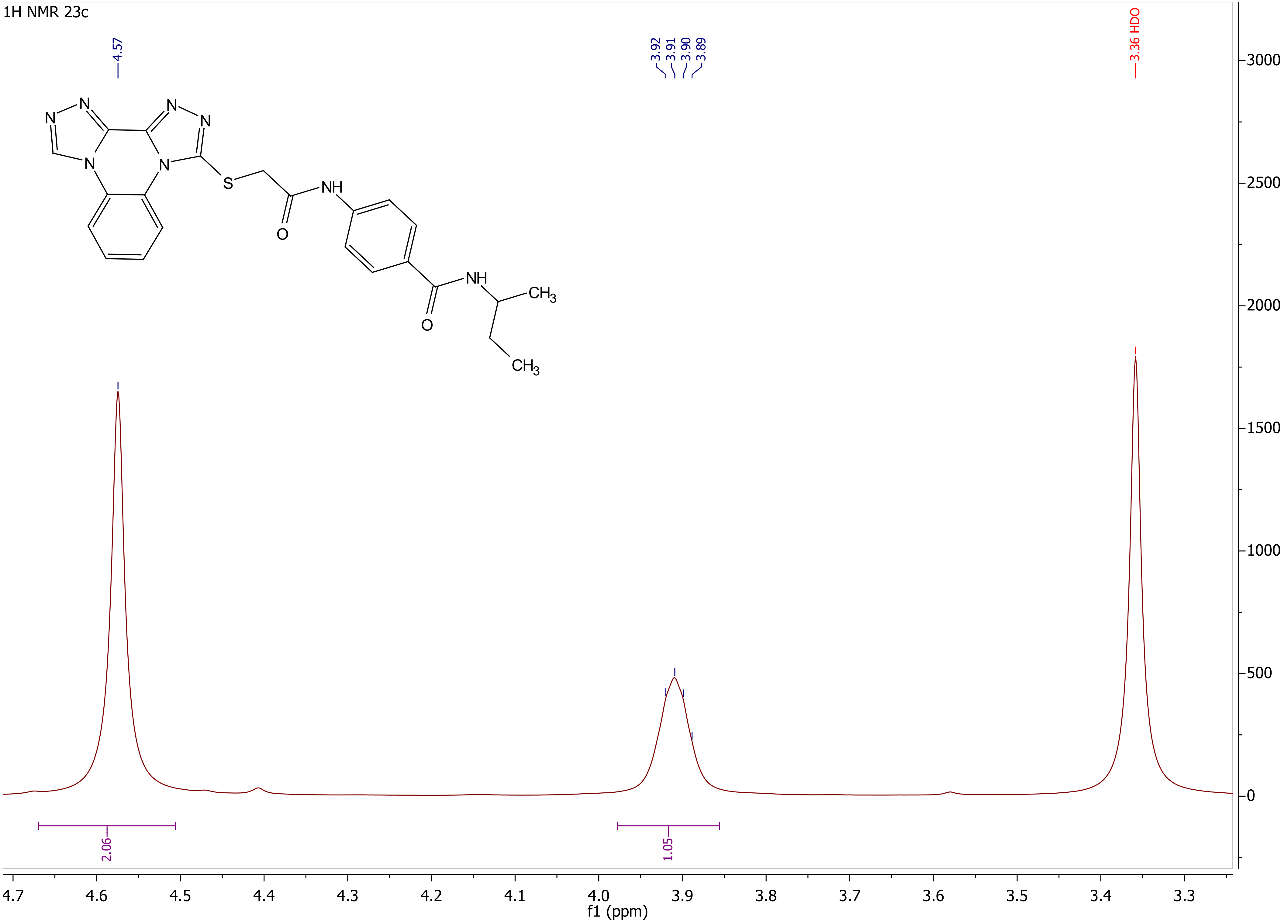
4.57
3.92
3.91
3.90
3.89
3.36 HDO
N
N
N
N
N
N
S
NH
O
NH
CH3
O
CH3
3000
2500
2000
1500
1000
500
0
2.06
1.05
4.7
4.6
4.5
4.4
4.3
4.2
4.1
4.0
3.9
3.8
3.7
3.6
3.5
3.4
3.3
f1 (ppm)

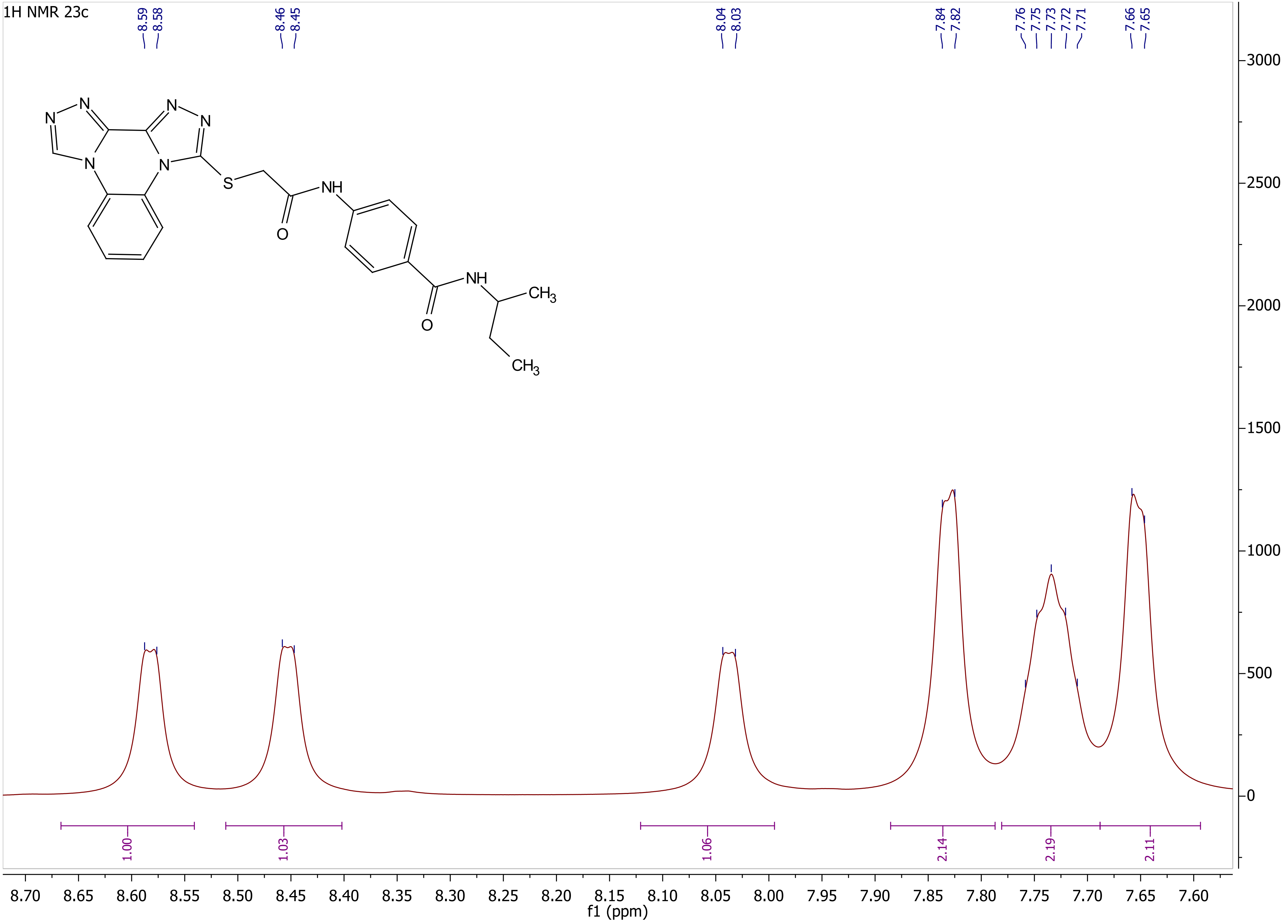
1H NMR 23c
8.59
8.58
8.46
8.45
8.04
8.03
7.84
7.82
7.76
7.75
7.73
7.72
7.71
7.66
7.65
N
N
N
N
N
N
S
NH
O
NH
O
CH3
CH3
3000
2500
2000
1500
1000
500
0
1.00
1.03
1.06
2.14
2.19
2.11
8.70
8.65
8.60
8.55
8.50
8.45
8.40
8.35
8.30
8.25
8.20
8.15
8.10
8.05
8.00
7.95
7.90
7.85
7.80
7.75
7.70
7.65
7.60
f1 (ppm)

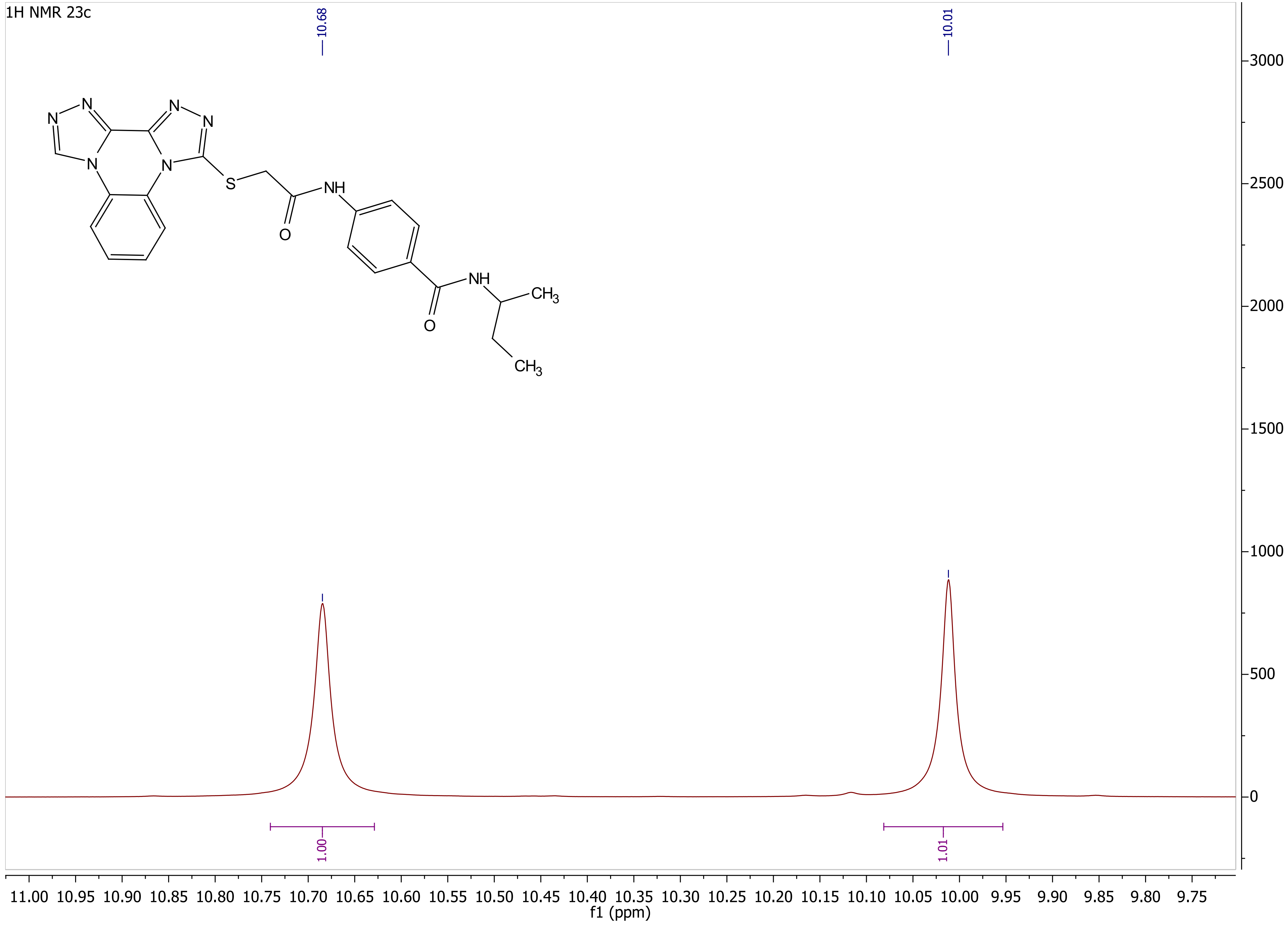
1H NMR 23c
10.68
10.01
1.00
1.01
f1 (ppm)
N
N
N
N
N
N
S
NH
O
NH
O
CH3
CH3

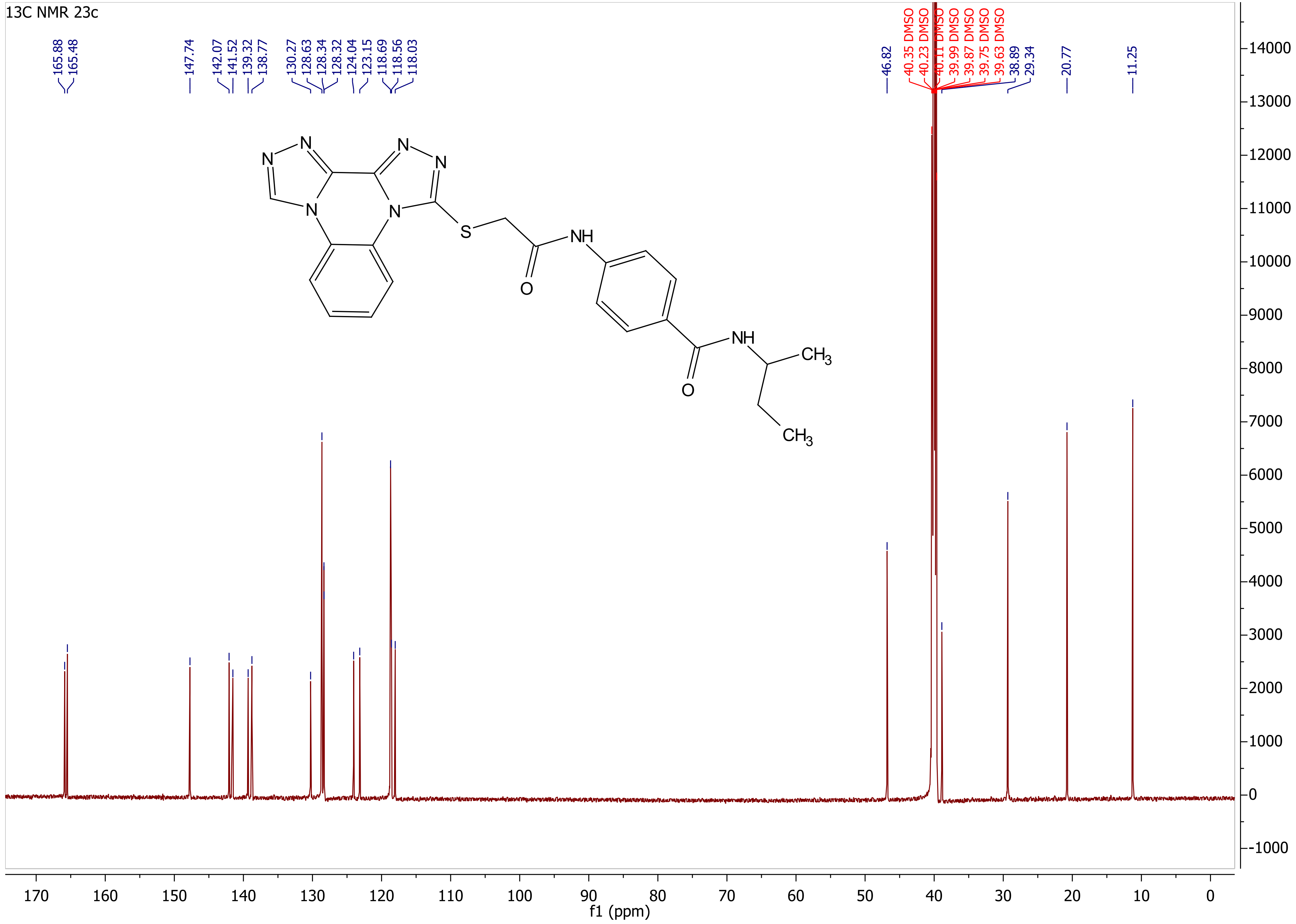

13C NMR 23c
165.88
165.48
147.74
142.07
141.52
139.32
138.77
130.27
128.63
128.34
128.32
124.04
123.15
118.69
118.56
118.03
46.82
40.35 DMSO
40.23 DMSO
40.11 DMSO
39.99 DMSO
39.87 DMSO
39.75 DMSO
39.63 DMSO
38.89
29.34
20.77
11.25
f1 (ppm)

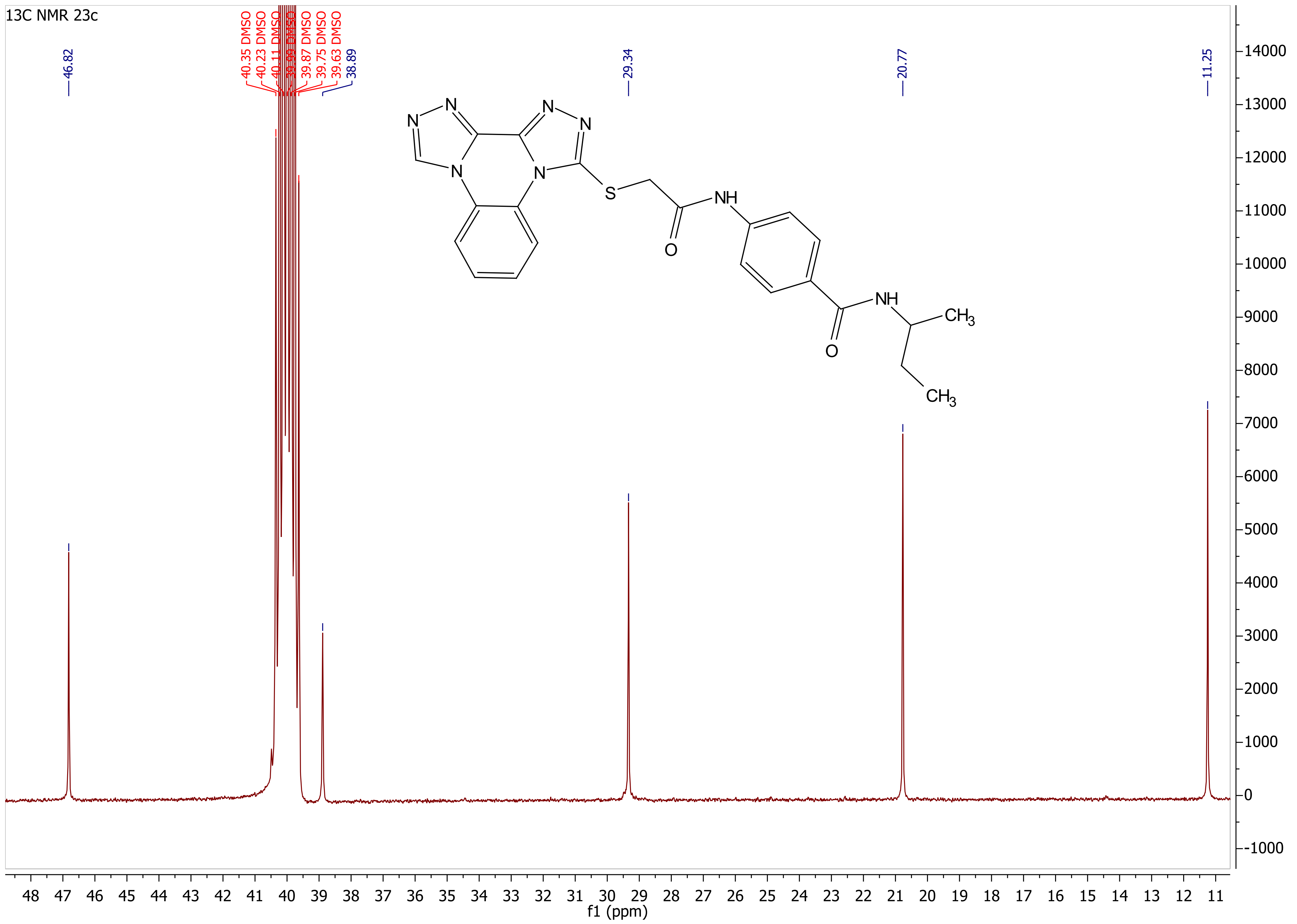

13C NMR 23c
46.82
40.35 DMSO
40.23 DMSO
40.11 DMSO
39.99 DMSO
39.87 DMSO
39.75 DMSO
39.63 DMSO
38.89
29.34
20.77
11.25
NH
O
NH
CH3
O
CH3
S
f1 (ppm)

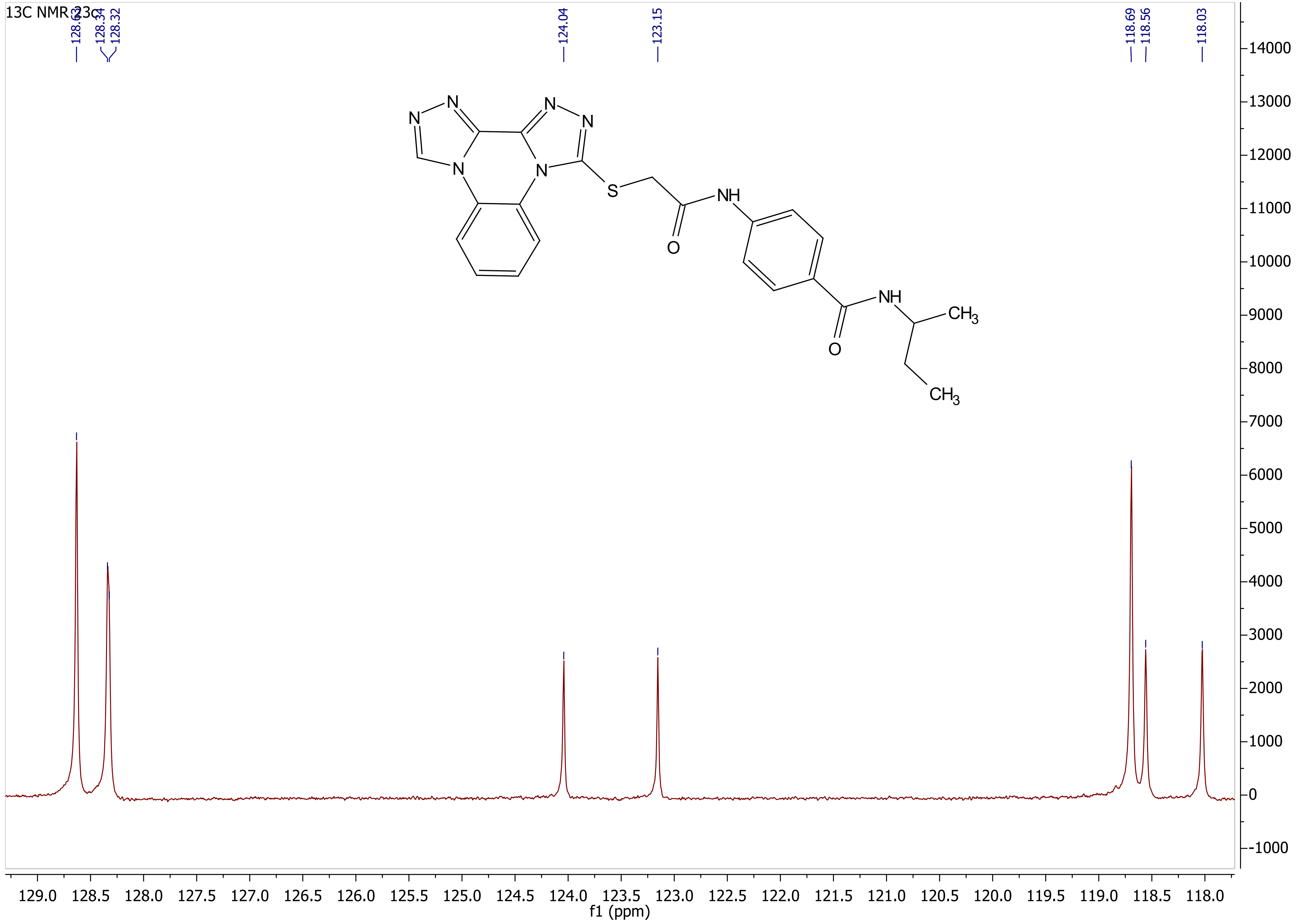
13C NMR 23c
128.63
128.34
128.32
124.04
123.15
118.69
118.56
118.03
N
N
N
N
N
N
S
NH
O
NH
O
CH_3
CH_3
f1 (ppm)
129.0 128.5 128.0 127.5 127.0 126.5 126.0 125.5 125.0 124.5 124.0 123.5 123.0 122.5 122.0 121.5 121.0 120.5 120.0 119.5 119.0 118.5 118.0
14000 13000 12000 11000 10000 9000 8000 7000 6000 5000 4000 3000 2000 1000 0 -1000

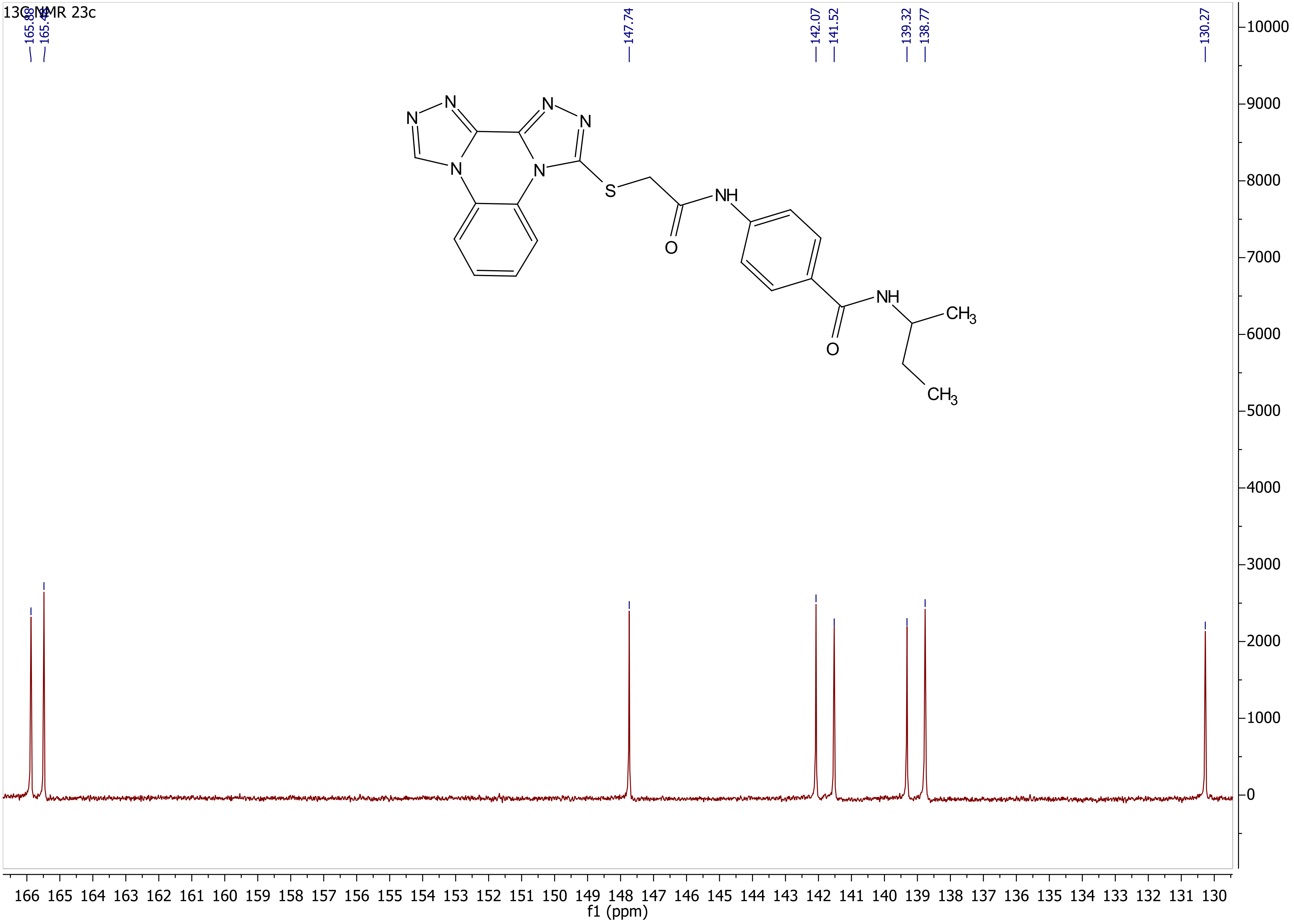
13C NMR 23c
165.88
165.48
147.74
142.07
141.52
139.32
138.77
130.27
N
N
N
N
N
N
S
NH
O
NH
O
CH3
CH3
f1 (ppm)
166 165 164 163 162 161 160 159 158 157 156 155 154 153 152 151 150 149 148 147 146 145 144 143 142 141 140 139 138 137 136 135 134 133 132 131 130
10000 9000 8000 7000 6000 5000 4000 3000 2000 1000 0

Mass spec. of 23c

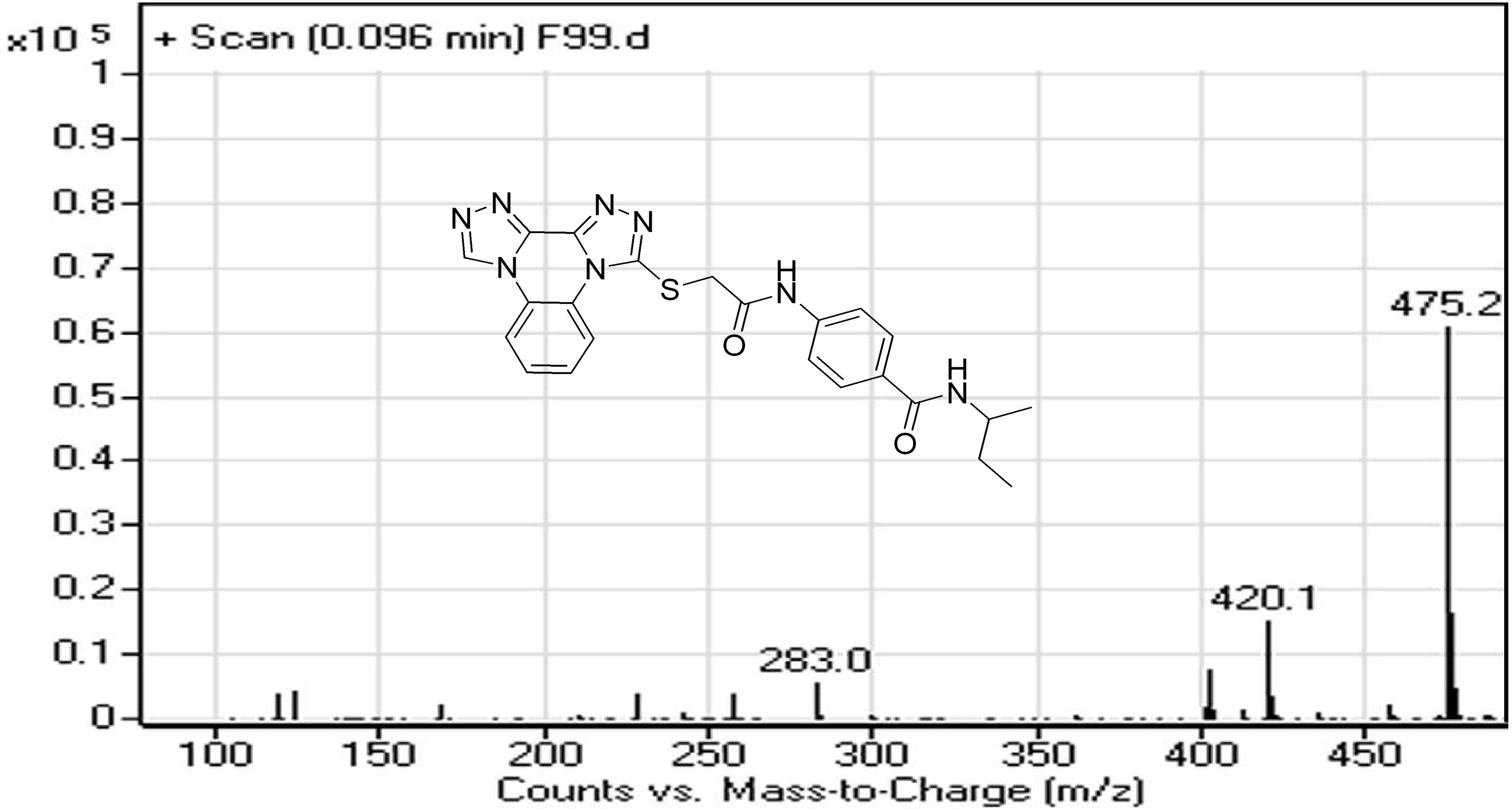

IR of compound 23d

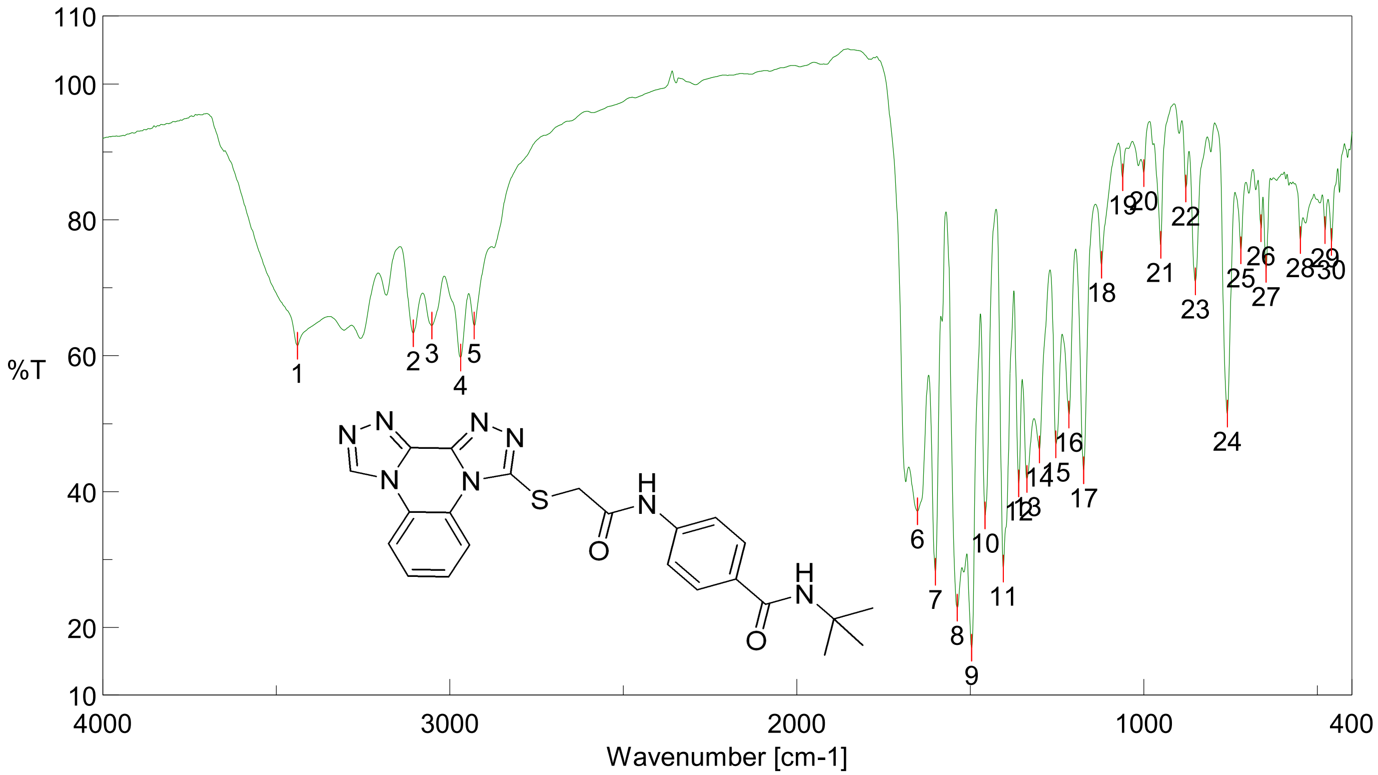

[Comments]
Sample name F102
Comment
User
Division
Company KSU

[Detailed Information]		[Measurement Information]	
Creation date	11/1/2020 12:18 AM	Model Name	FT/IR-6600typeA
		Serial Number	A014661790
Data array type	Linear data array		
Horizontal axis	Wavenumber [cm-1]	Measurement Date	10/28/2020 5:25 AM
Vertical axis	%T		
Start	399.193 cm-1	Light Source	Standard
End	4000.6 cm-1	Detector	TGS
Data interval	0.964233 cm-1	Accumulation	Auto (17)
Data points	3736	Resolution	4 cm-1
		Zero Filling	On
		Apodization	Cosine
		Gain	Auto (2)
		Aperture	Auto (7.1 mm)
		Scanning Speed	Auto (2 mm/sec)
		Filter	Auto (10000 Hz)

[Result of Peak Picking]

No.	Position	Intensity	No.	Position	Intensity	No.	Position	Intensity
1	3439.42	61.4235	2	3104.83	63.2838	3	3051.8	64.4089

[Result of Peak Picking]

No.	Position	Intensity	No.	Position	Intensity	No.	Position	Intensity
4	2968.87	59.6962	5	2929.34	64.4165	6	1651.73	37.0577
7	1600.63	28.1599	8	1537.95	22.8619	9	1496.49	16.9752
10	1456.96	36.4502	11	1404.89	28.6293	12	1360.53	41.163
13	1336.43	41.8359	14	1300.75	46.1852	15	1253.5	46.921
16	1215.9	51.2826	17	1173.47	43.1012	18	1121.4	73.3837
19	1060.66	86.2562	20	999.91	86.8706	21	950.734	76.3065
22	878.417	84.5872	23	851.418	70.9182	24	758.852	51.4539
25	720.282	75.4688	26	662.428	78.725	27	647.001	72.7644
28	548.649	77.0171	29	477.296	78.435	30	458.975	76.6945

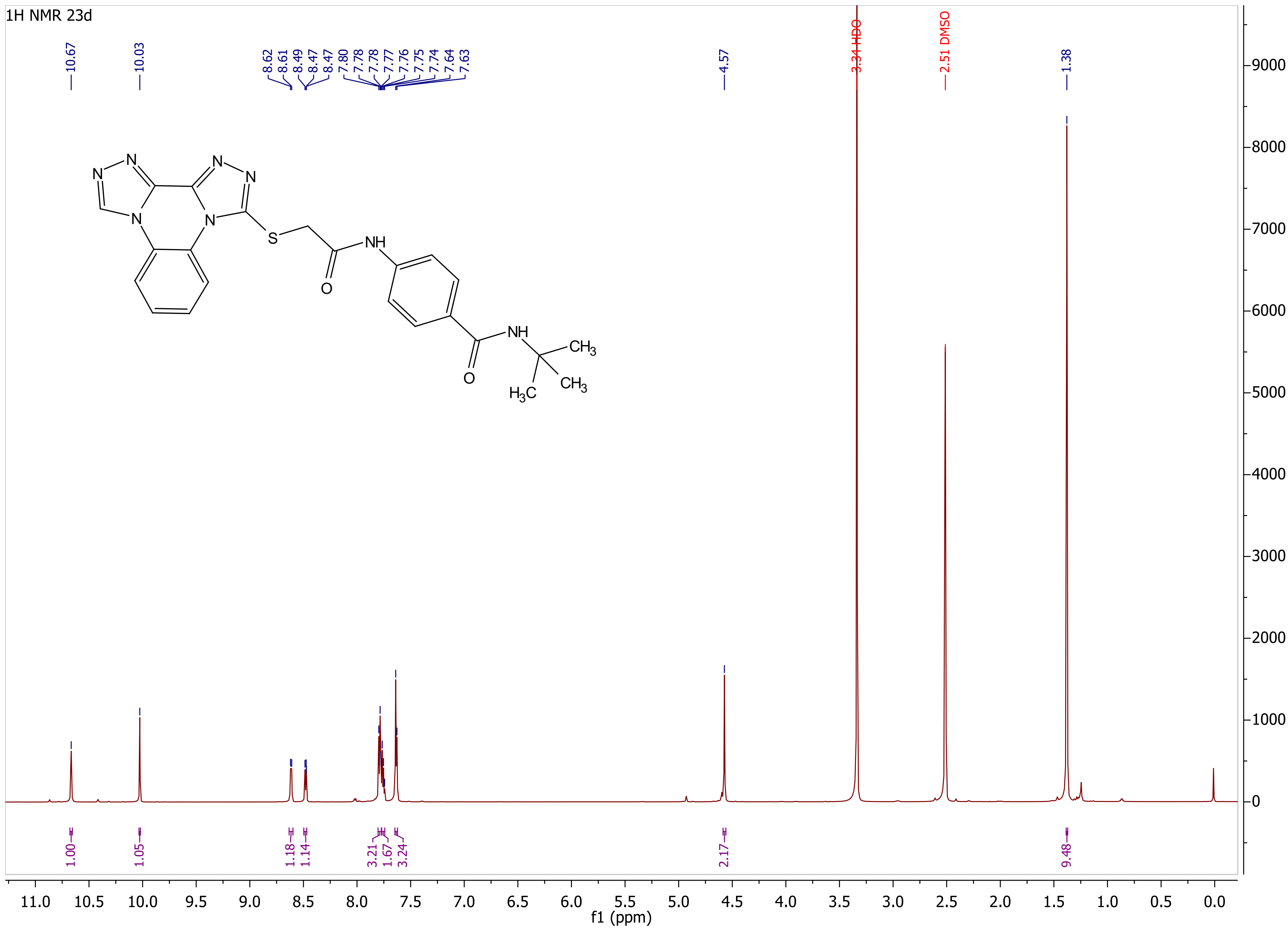
1H NMR 23d
10.67
10.03
8.62
8.61
8.49
8.47
8.47
7.80
7.78
7.78
7.77
7.76
7.75
7.74
7.64
7.63
4.57
3.34 HDO
2.51 DMSO
1.38
1.00
1.05
1.18
1.14
3.21
1.67
3.24
2.17
9.48
f1 (ppm)

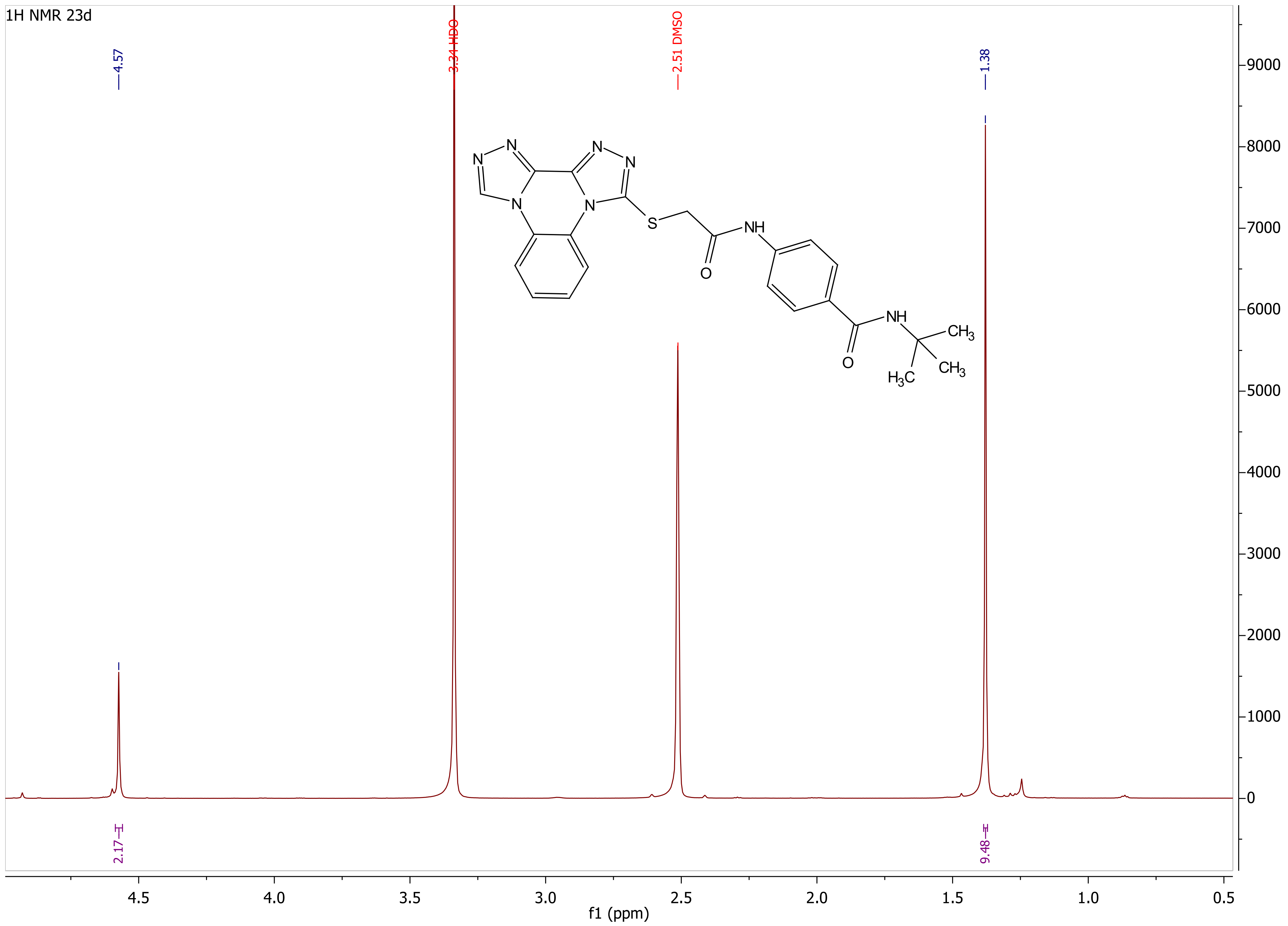
1H NMR 23d
4.57
3.34 HDO
2.51 DMSO
1.38
N
N
N
N
N
N
S
NH
O
NH
O
CH3
CH3
H3C
2.17
9.48
9000
8000
7000
6000
5000
4000
3000
2000
1000
0
4.5
4.0
3.5
3.0
2.5
2.0
1.5
1.0
0.5
f1 (ppm)

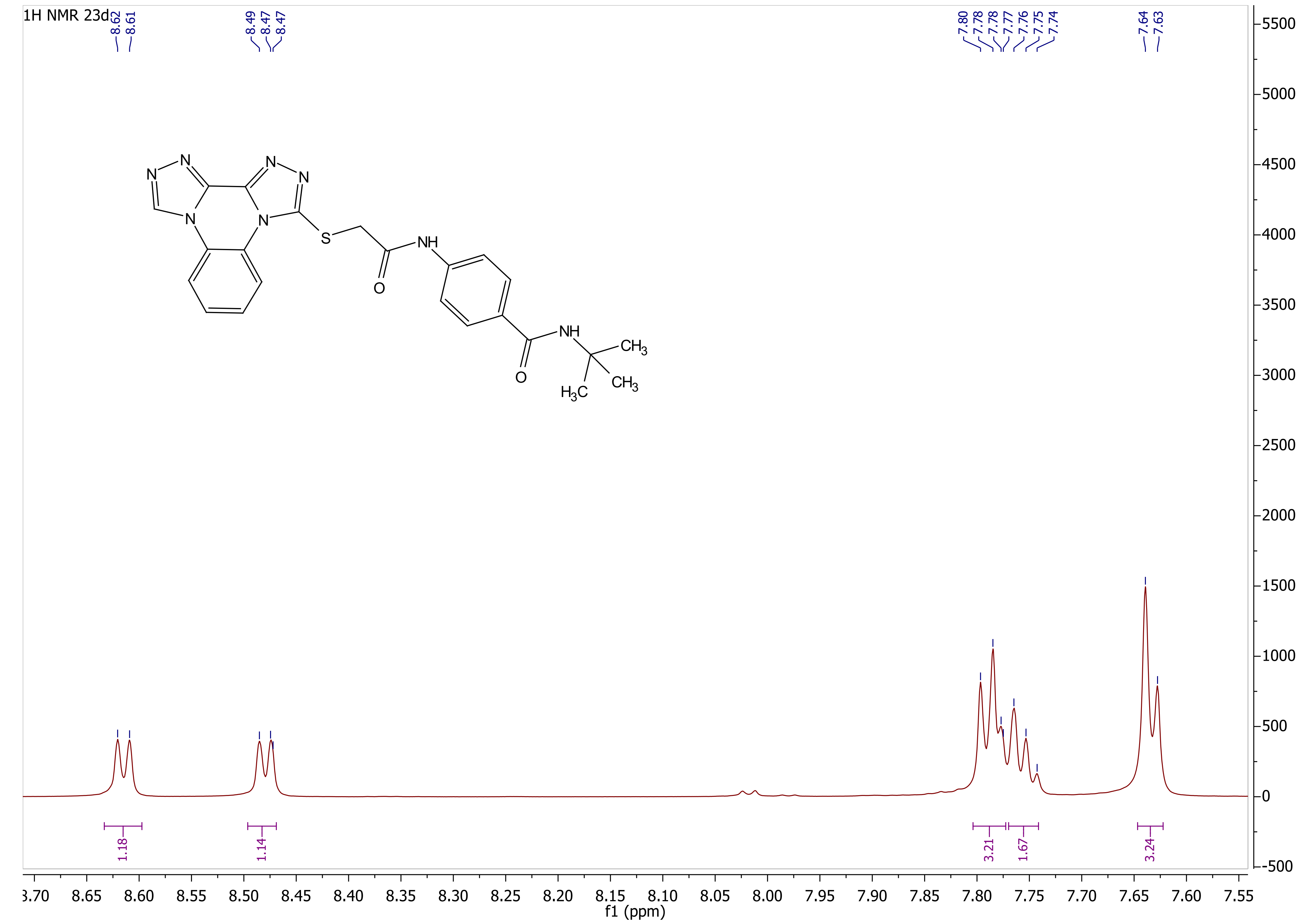

1H NMR 23d
8.62
8.61
8.49
8.47
8.47
7.80
7.78
7.78
7.77
7.76
7.75
7.74
7.64
7.63
1.18
1.14
3.21
1.67
3.24
f1 (ppm)

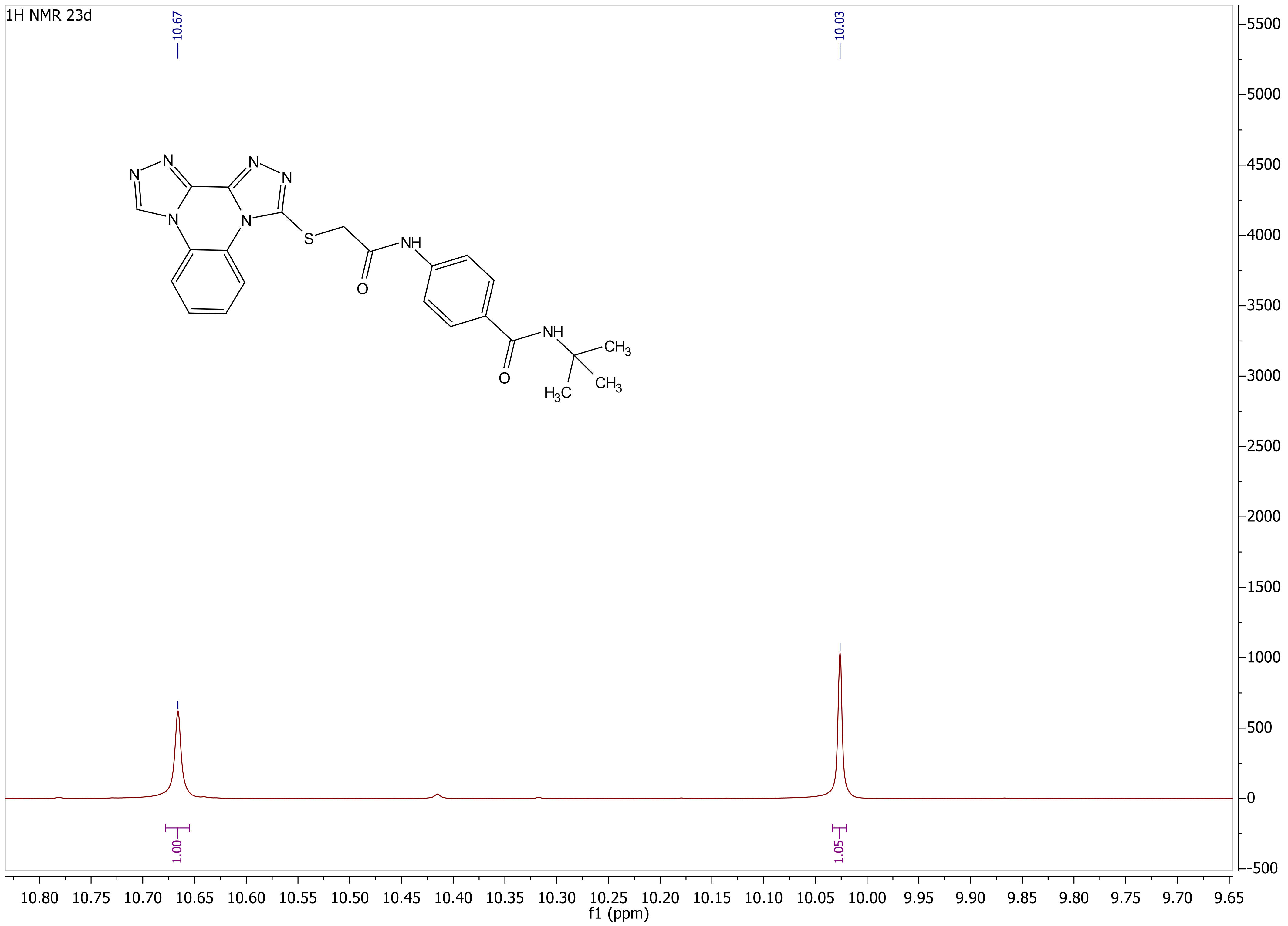
1H NMR 23d
10.67
10.03
N
N
N
N
N
N
N
S
O
NH
NH
O
CH3
CH3
H3C
1.00
1.05
10.80 10.75 10.70 10.65 10.60 10.55 10.50 10.45 10.40 10.35 10.30 10.25 10.20 10.15 10.10 10.05 10.00 9.95 9.90 9.85 9.80 9.75 9.70 9.65
f1 (ppm)
5500 5000 4500 4000 3500 3000 2500 2000 1500 1000 500 0 -500

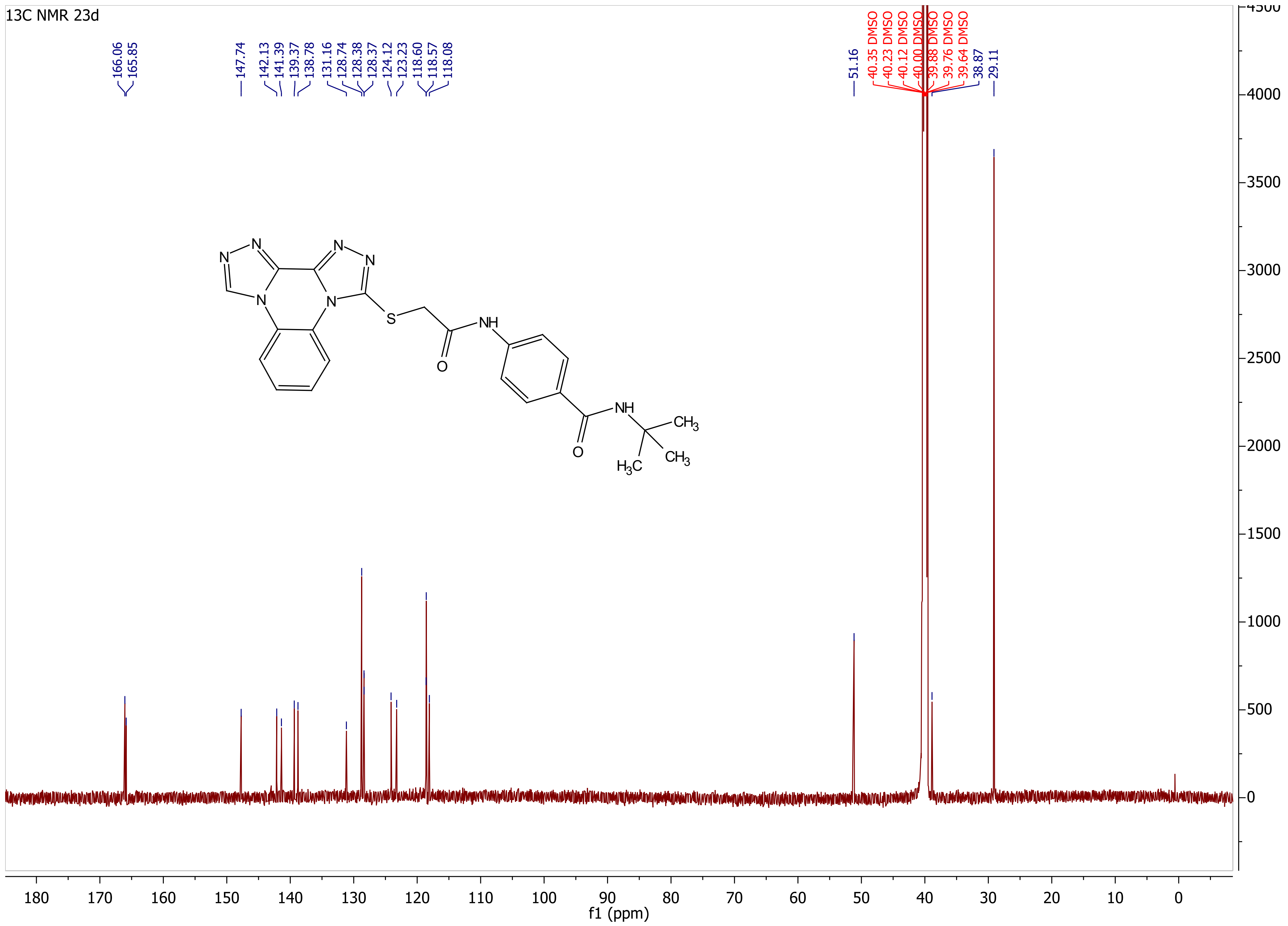

13C NMR 23d
166.06
165.85
147.74
142.13
141.39
139.37
138.78
131.16
128.74
128.38
128.37
124.12
123.23
118.60
118.57
118.08
51.16
40.35 DMSO
40.23 DMSO
40.12 DMSO
40.00 DMSO
39.88 DMSO
39.76 DMSO
39.64 DMSO
38.87
29.11
N
S
NH
O
CH3
H3C
f1 (ppm)

13C NMR 23d

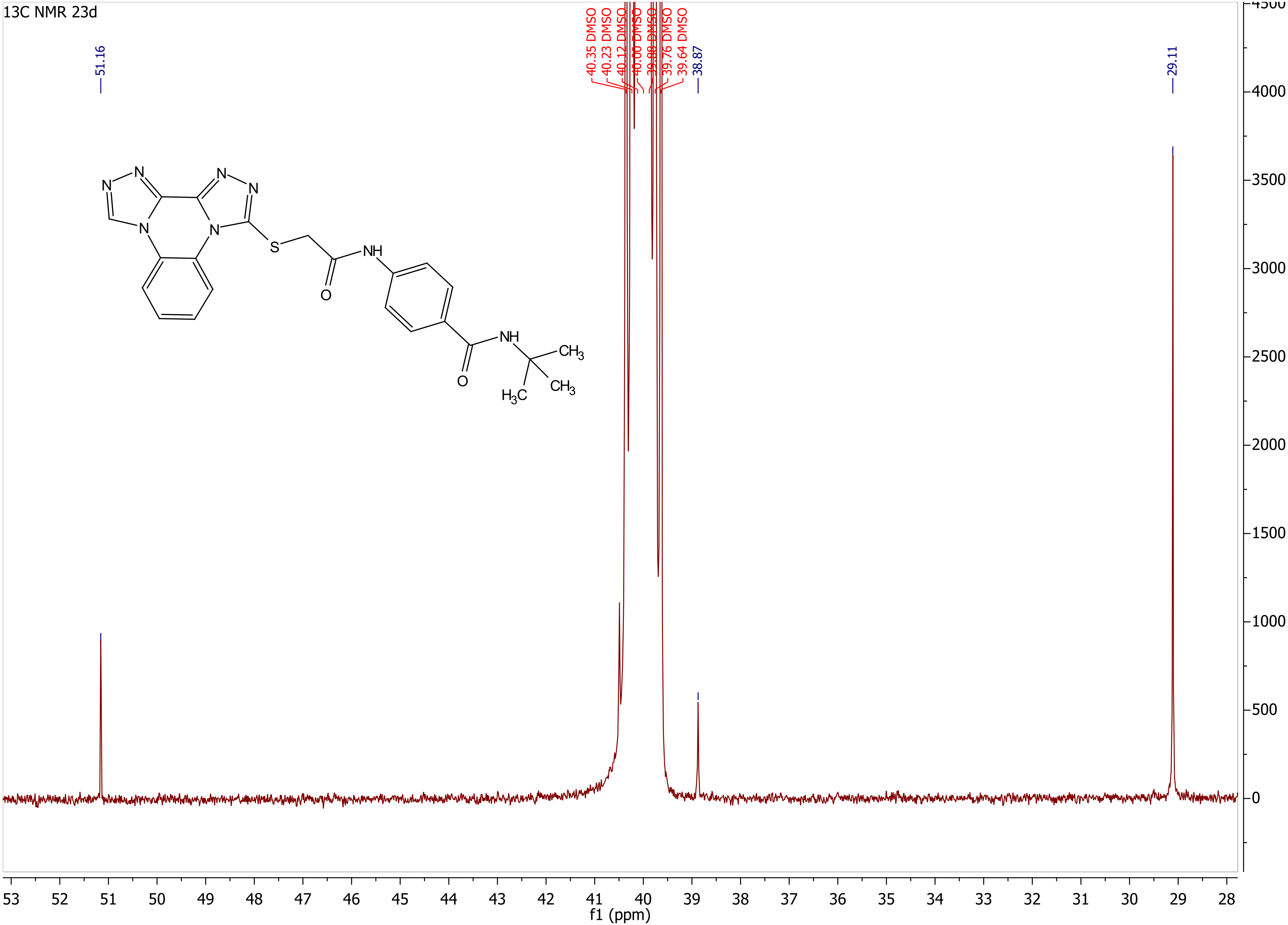

51.16
40.35 DMSO
40.23 DMSO
40.12 DMSO
40.00 DMSO
39.88 DMSO
39.76 DMSO
39.64 DMSO
38.87
29.11
f1 (ppm)

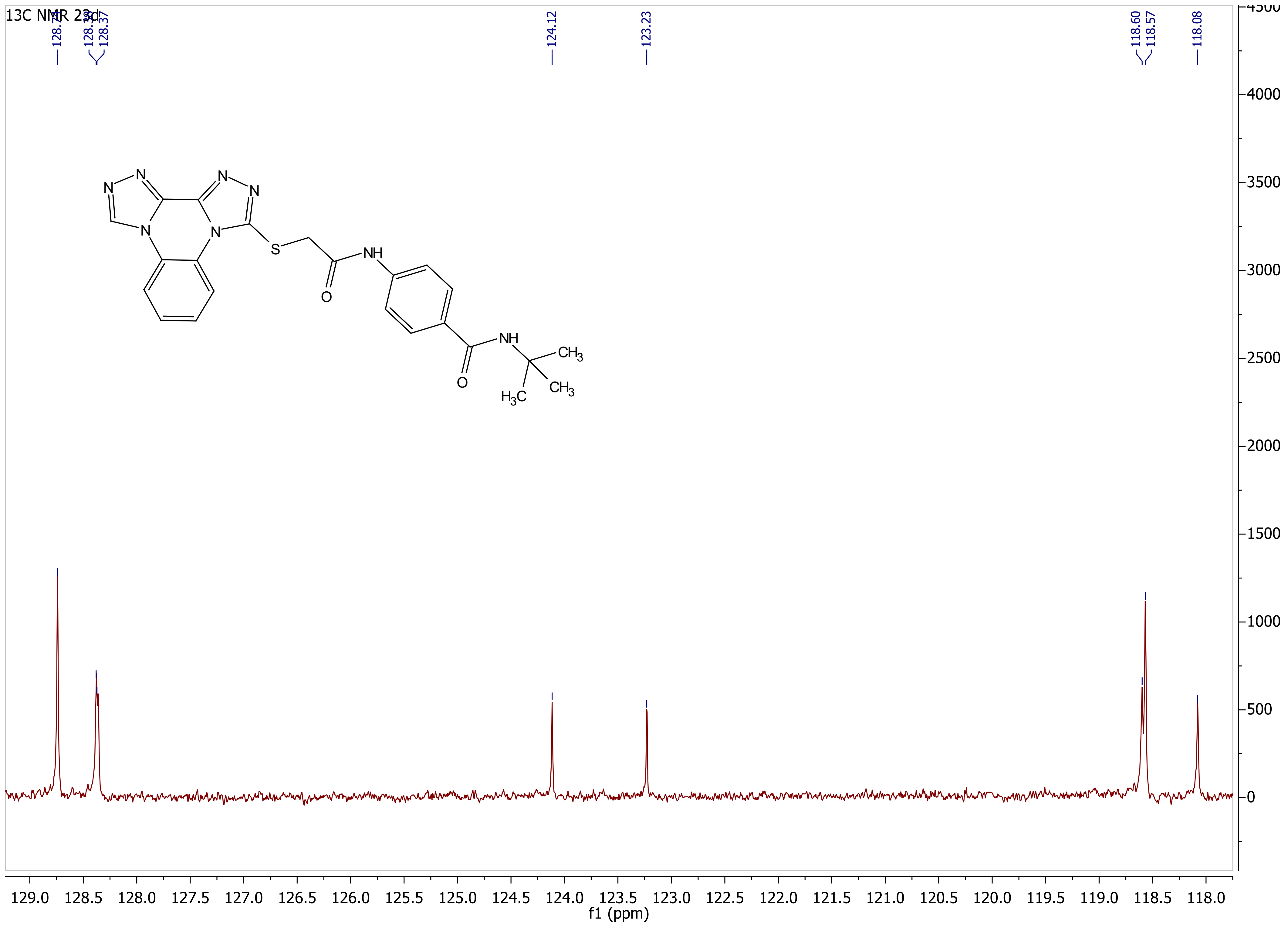

13C NMR 23d
128.73
128.39
128.37
124.12
123.23
118.60
118.57
118.08
N
N
N
N
N
N
S
O
NH
NH
O
CH3
CH3
H3C
f1 (ppm)

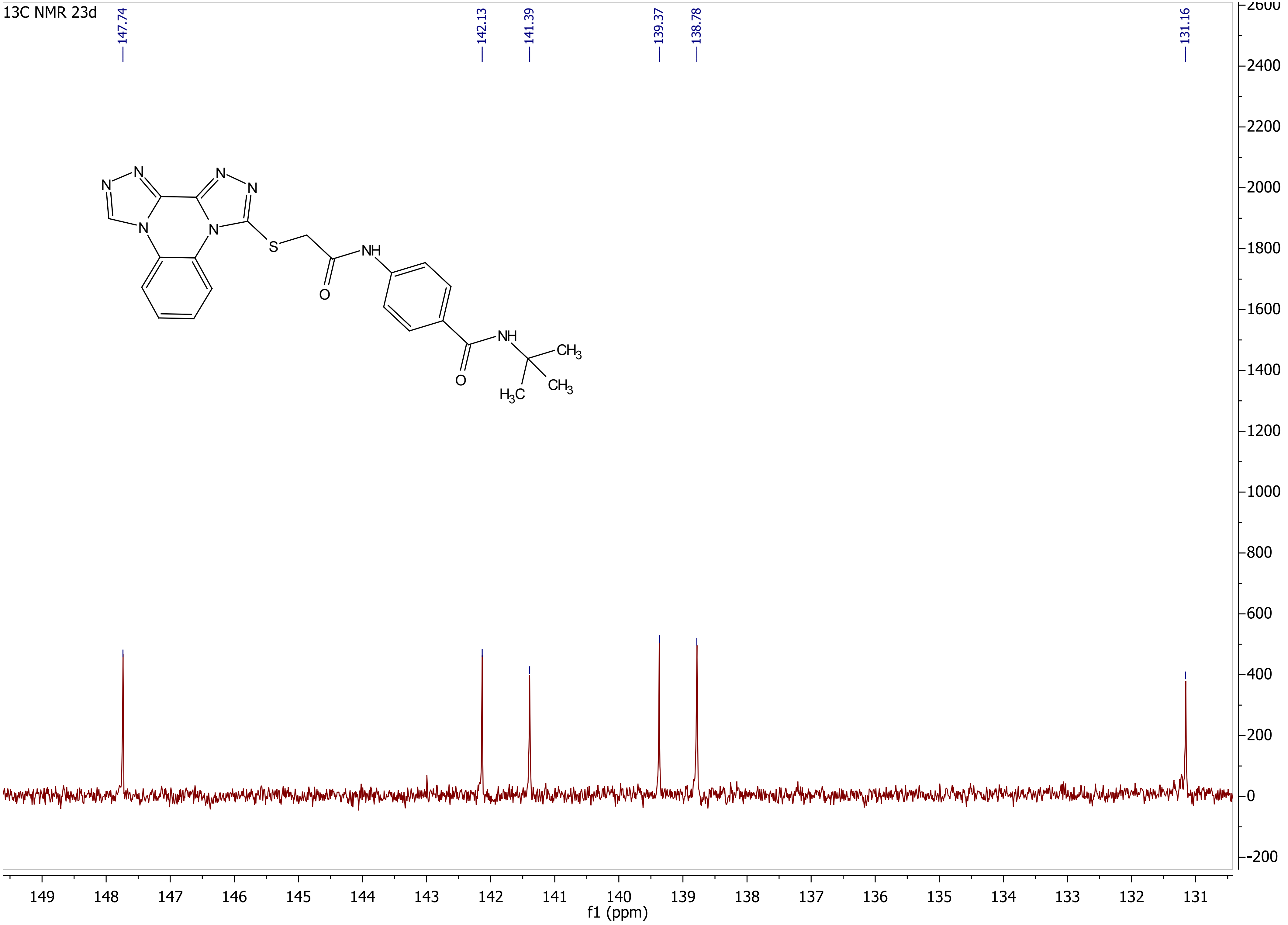
13C NMR 23d
147.74
142.13
141.39
139.37
138.78
131.16
N
N
N
N
N
N
S
NH
O
NH
O
CH3
CH3
H3C
f1 (ppm)

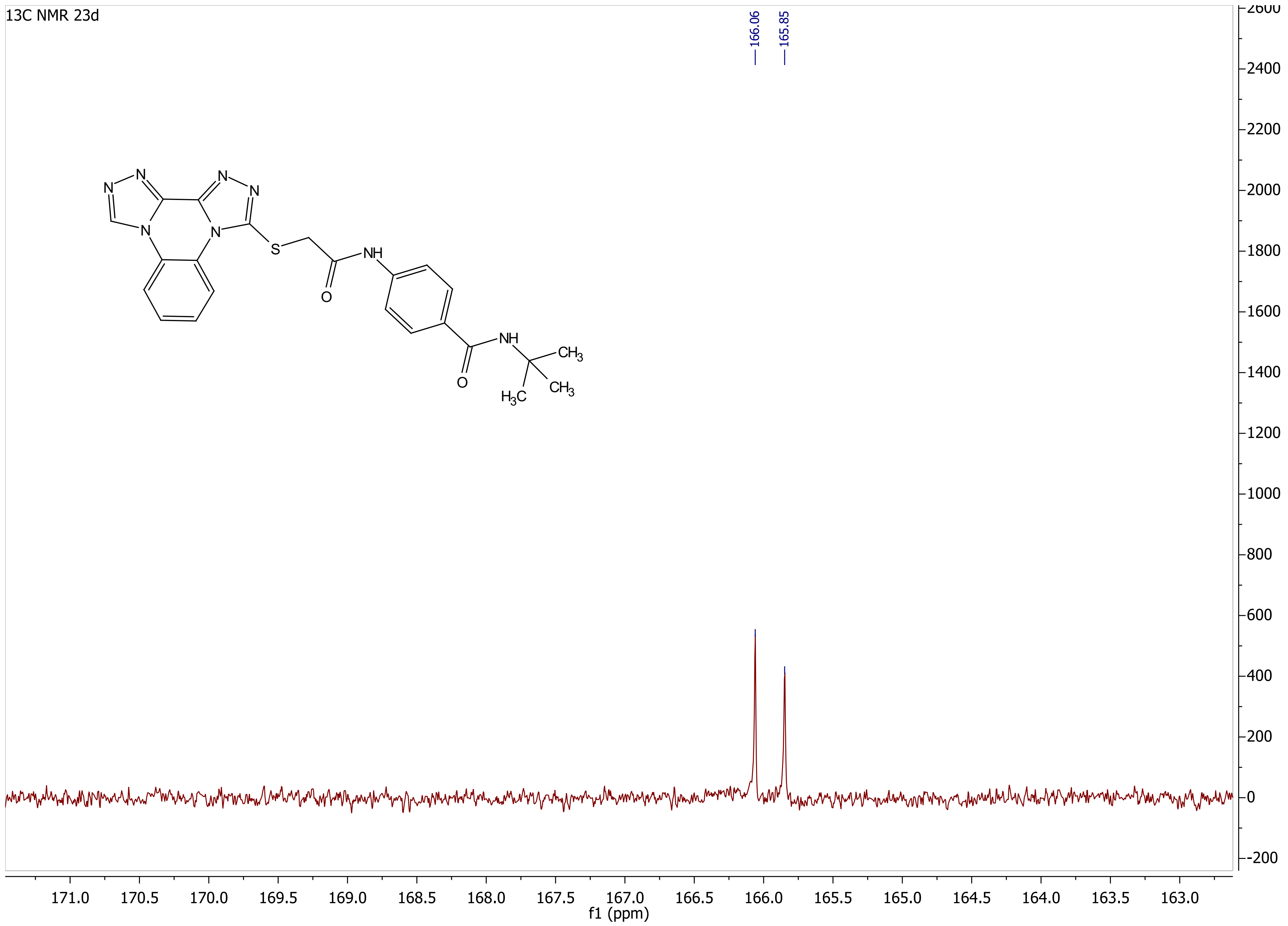
13C NMR 23d
166.06
165.85
N
N
N
N
N
N
S
O
NH
NH
O
CH3
CH3
H3C
2600
2400
2200
2000
1800
1600
1400
1200
1000
800
600
400
200
0
-200
171.0
170.5
170.0
169.5
169.0
168.5
168.0
167.5
167.0
166.5
166.0
165.5
165.0
164.5
164.0
163.5
163.0
f1 (ppm)

Mass spec. of 23d

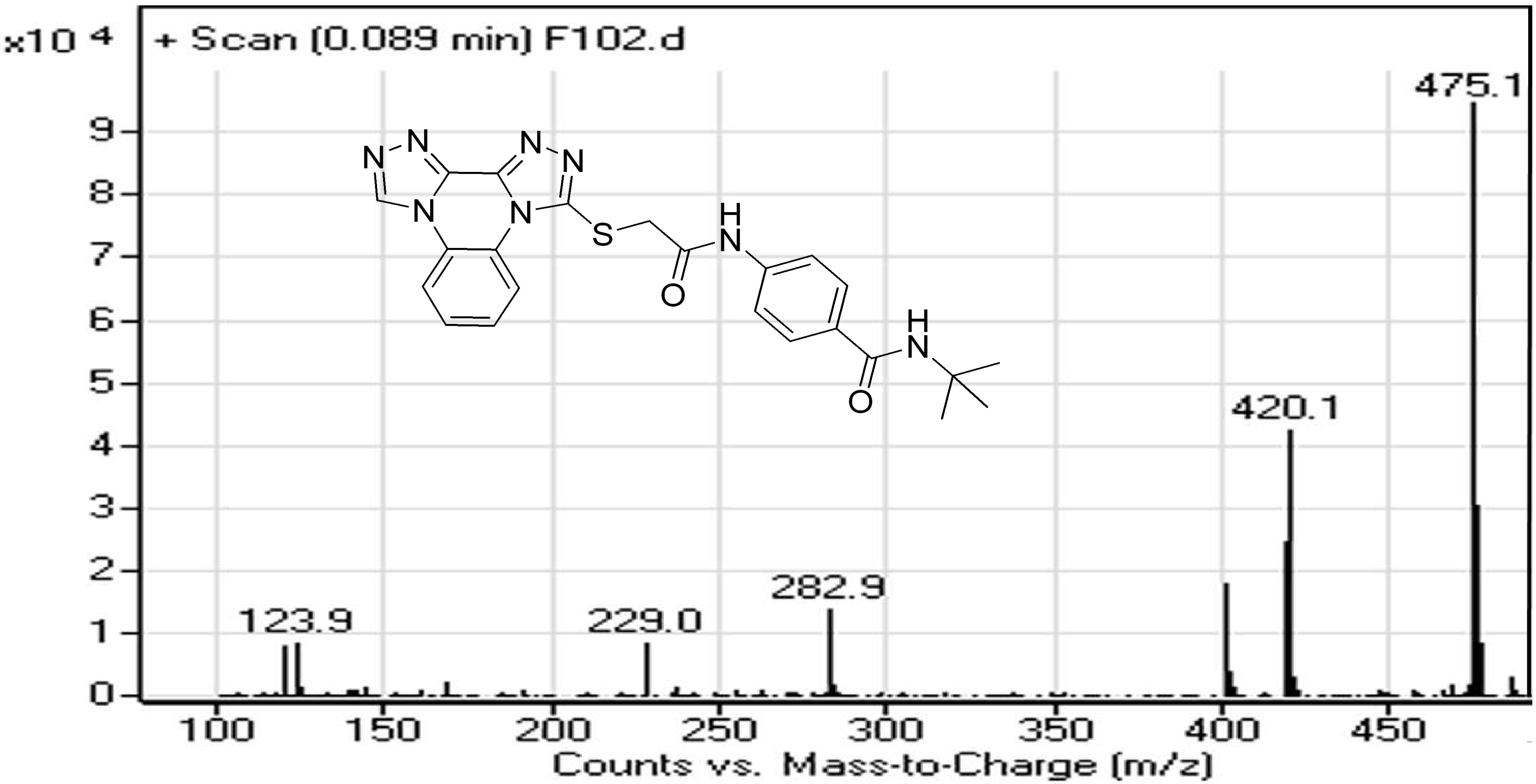

IR of compound 92

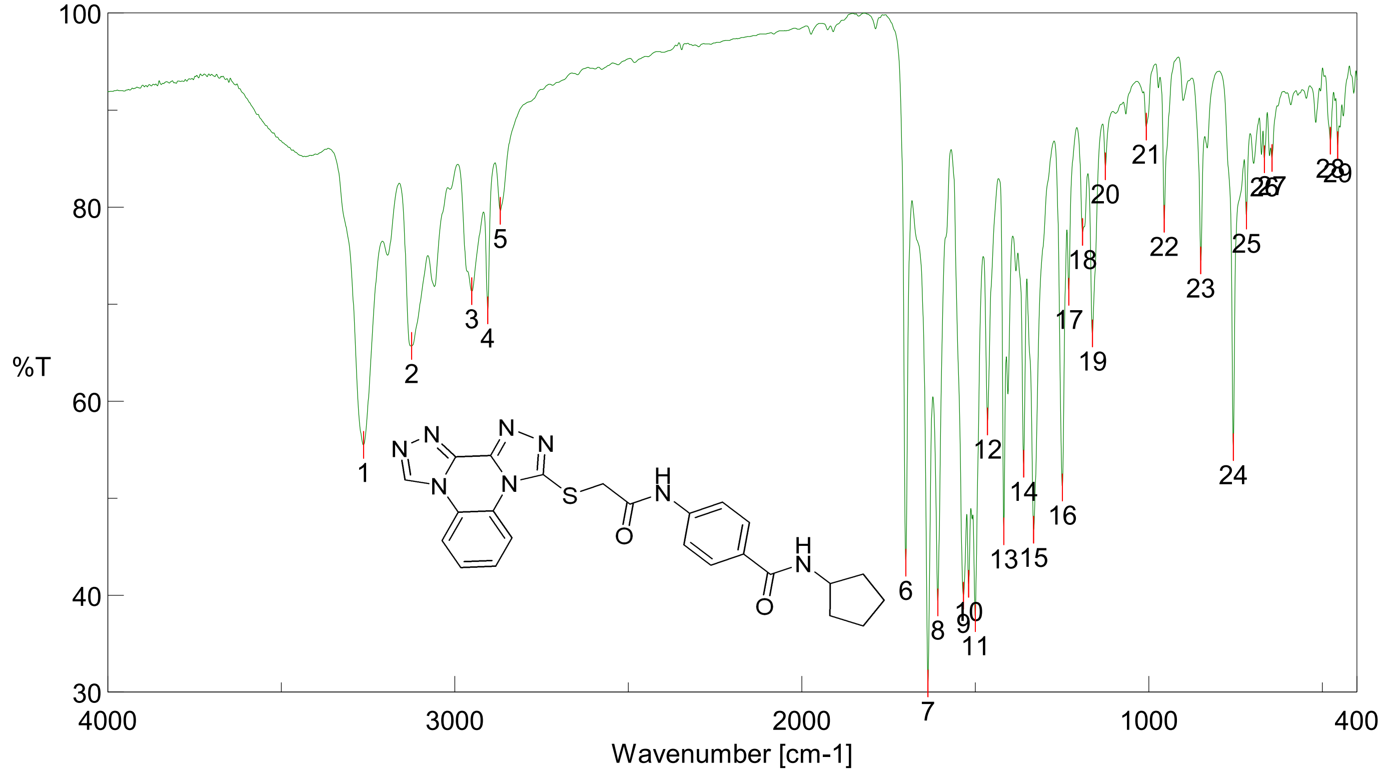

[Comments]

Sample name	F92
Comment	
User	
Division	
Company	KSU

[Detailed Information]

Creation date	10/29/2020 5:12 AM
Data array type	Linear data array
Horizontal axis	Wavenumber [cm-1]
Vertical axis	%T
Start	399.193 cm-1
End	4000.6 cm-1
Data interval	0.964233 cm-1
Data points	3736

[Measurement Information]

Model Name	FT/IR-6600typeA
Serial Number	A014661790
Measurement Date	10/28/2020 4:25 AM
Light Source	Standard
Detector	TGS
Accumulation	Auto (17)
Resolution	4 cm-1
Zero Filling	On
Apodization	Cosine
Gain	Auto (2)
Aperture	Auto (7.1 mm)
Scanning Speed	Auto (2 mm/sec)
Filter	Auto (10000 Hz)

[Result of Peak Picking]

No.	Position	Intensity	No.	Position	Intensity	No.	Position	Intensity
1	3262.97	55.4769	2	3124.12	65.6832	3	2950.55	71.3081

[Result of Peak Picking]

No.	Position	Intensity	No.	Position	Intensity	No.	Position	Intensity
4	2905.24	69.3508	5	2868.59	79.6154	6	1699.94	43.3354
7	1636.3	30.8948	8	1607.38	39.2453	9	1534.1	39.9475
10	1518.67	41.166	11	1499.38	37.6253	12	1464.67	57.8944
13	1417.42	46.5579	14	1360.53	53.5218	15	1331.61	46.7405
16	1248.68	51.0761	17	1230.36	71.2434	18	1190.83	77.4142
19	1161.9	66.9672	20	1125.26	84.2057	21	1006.66	88.2818
22	955.555	78.7798	23	850.454	74.503	24	755.959	55.2439
25	718.354	79.1077	26	666.285	84.9145	27	644.108	85.0496
28	476.331	86.8429	29	455.118	86.3826			

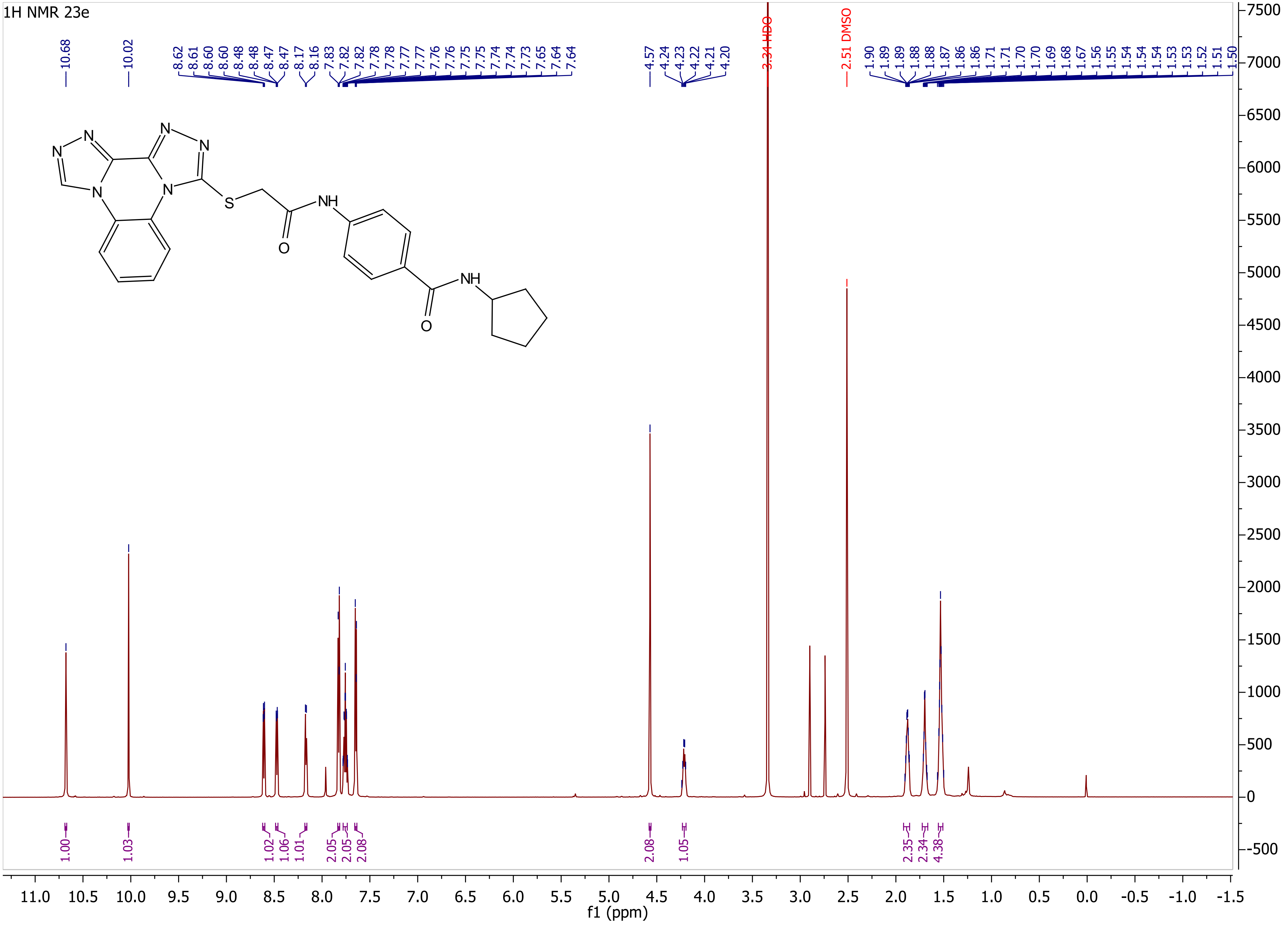

1H NMR 23e
10.68
10.02
8.62 8.61 8.60 8.60 8.48 8.48 8.47 8.47 8.17 8.16 7.83 7.82 7.82 7.78 7.78 7.77 7.77 7.76 7.76 7.75 7.75 7.74 7.74 7.73 7.65 7.64 7.64
4.57 4.24 4.23 4.22 4.21 4.20
3.34 HDO
2.51 DMSO
1.90 1.89 1.89 1.88 1.88 1.87 1.86 1.71 1.71 1.70 1.70 1.69 1.68 1.67 1.56 1.55 1.54 1.54 1.53 1.53 1.52 1.51 1.50
1.00 1.03 1.02 1.06 1.01 2.05 2.05 2.08 2.08 1.05 2.35 2.34 4.38
f1 (ppm)

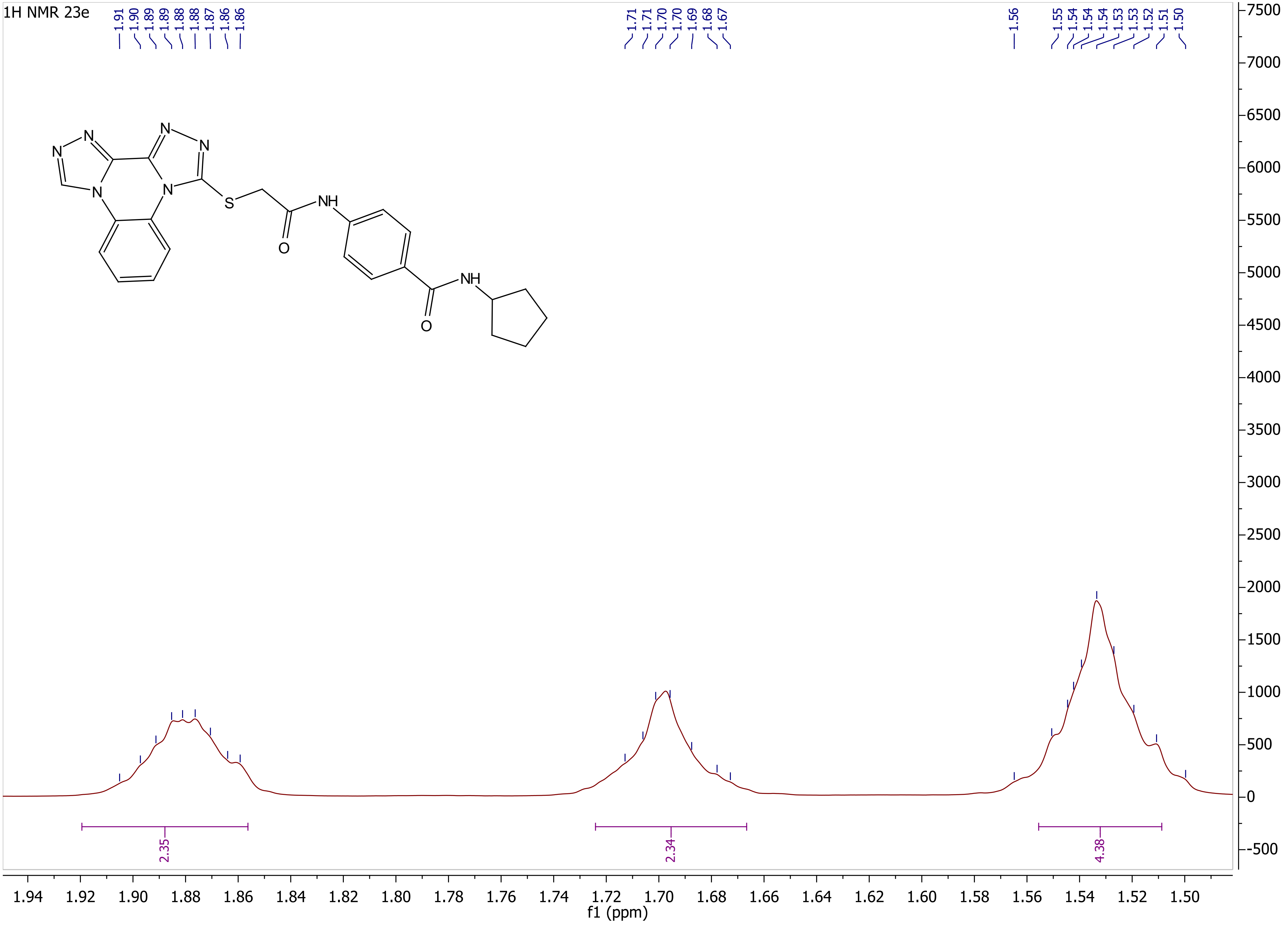
1H NMR 23e
1.91
1.90
1.89
1.89
1.88
1.88
1.87
1.86
1.86
1.71
1.71
1.70
1.70
1.69
1.68
1.67
1.56
1.55
1.54
1.54
1.54
1.53
1.53
1.52
1.51
1.50
N
N
N
N
N
N
S
NH
O
NH
O
2.35
2.34
4.38
1.94
1.92
1.90
1.88
1.86
1.84
1.82
1.80
1.78
1.76
1.74
1.72
1.70
1.68
1.66
1.64
1.62
1.60
1.58
1.56
1.54
1.52
1.50
f1 (ppm)
7500
7000
6500
6000
5500
5000
4500
4000
3500
3000
2500
2000
1500
1000
500
0
-500

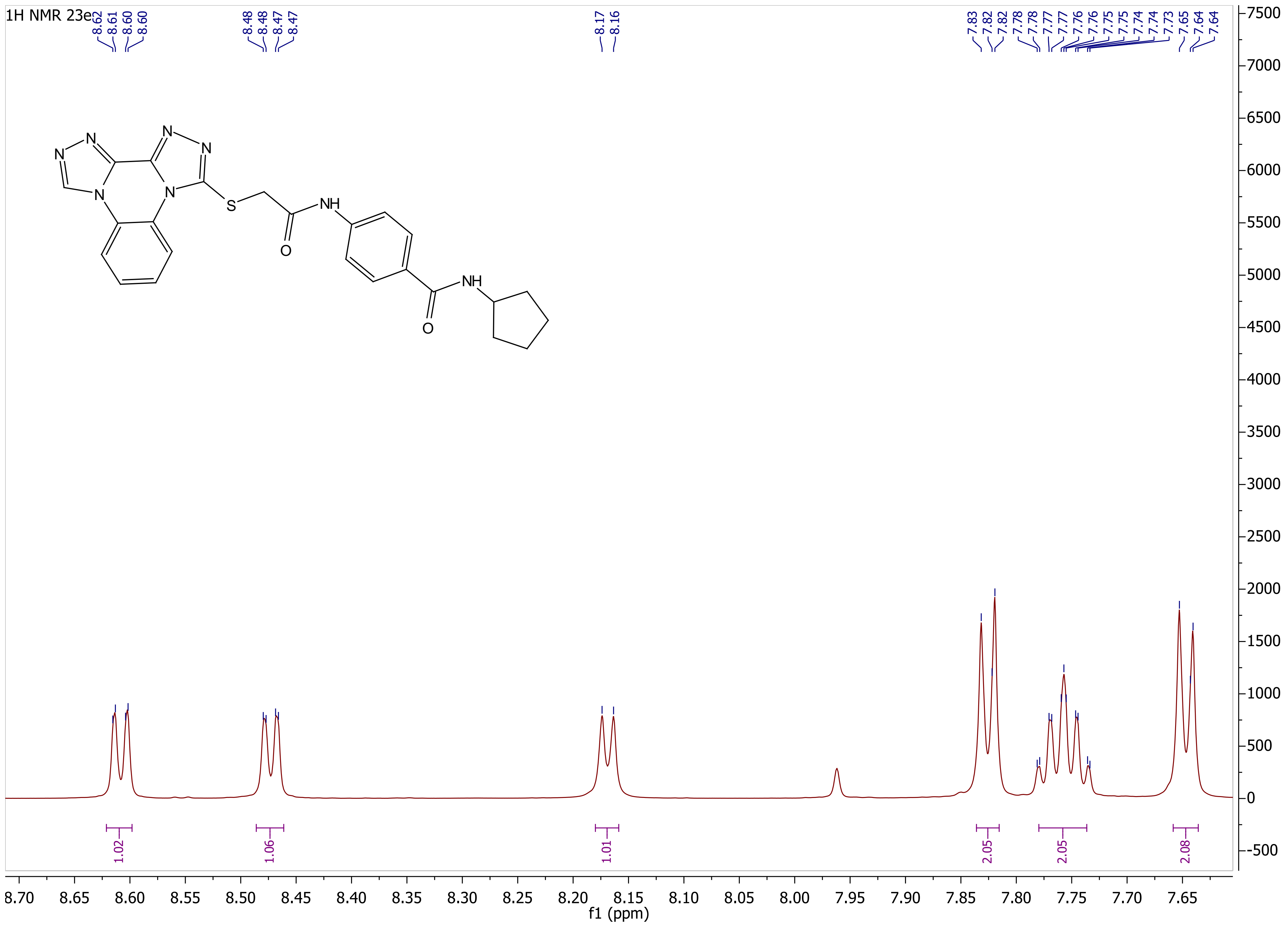
1H NMR 23e
8.62
8.61
8.60
8.60
8.48
8.48
8.47
8.47
8.17
8.16
7.83
7.82
7.82
7.78
7.78
7.77
7.77
7.76
7.75
7.75
7.74
7.74
7.73
7.65
7.64
7.64
N
N
N
N
N
N
S
NH
O
NH
O
1.02
1.06
1.01
2.05
2.05
2.08
f1 (ppm)

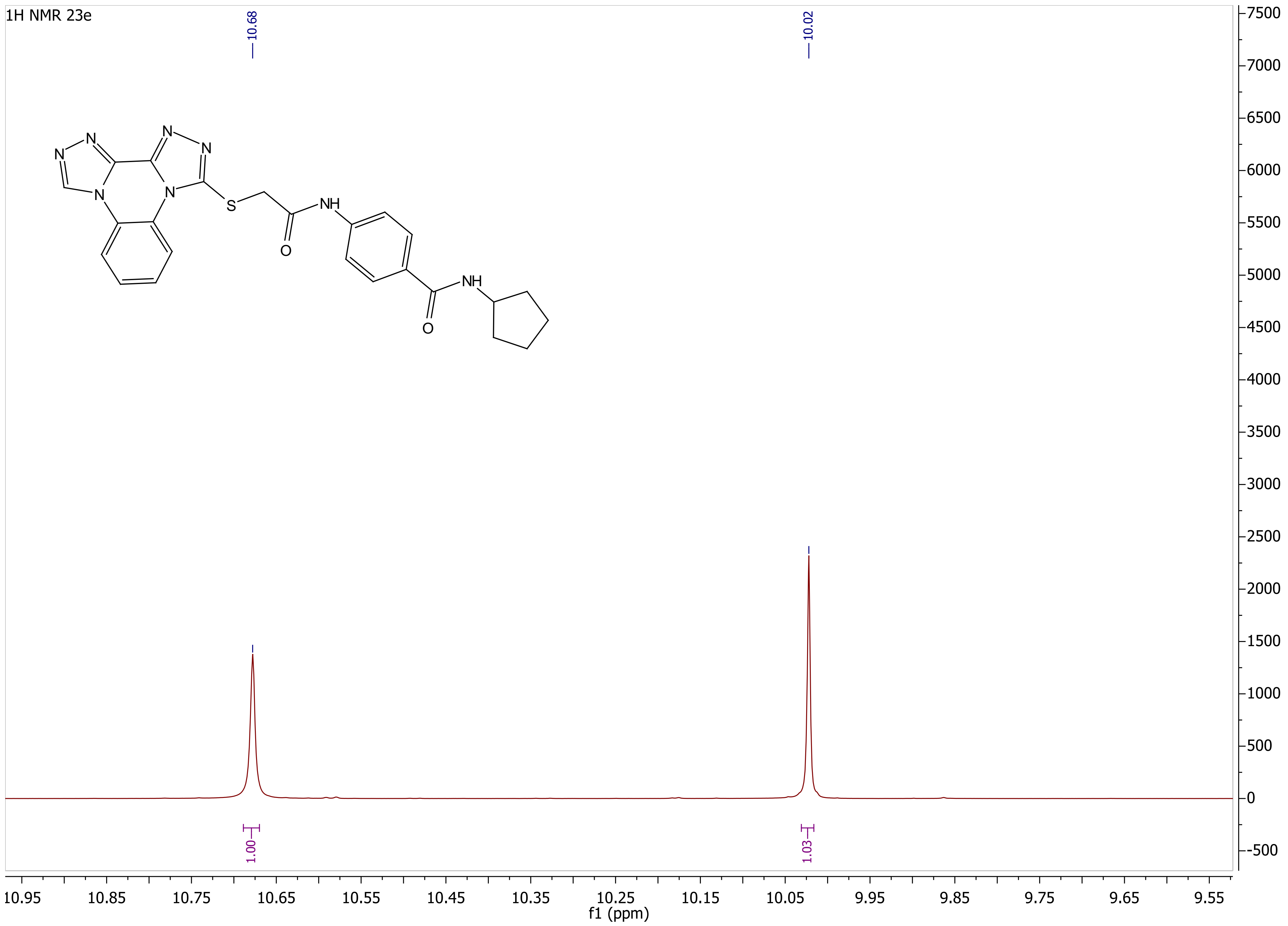
1H NMR 23e
10.68
10.02
N
N
N
N
N
N
N
S
O
NH
NH
O
1.00
1.03
7500
7000
6500
6000
5500
5000
4500
4000
3500
3000
2500
2000
1500
1000
500
0
-500
10.95
10.85
10.75
10.65
10.55
10.45
10.35
10.25
10.15
10.05
9.95
9.85
9.75
9.65
9.55
f1 (ppm)

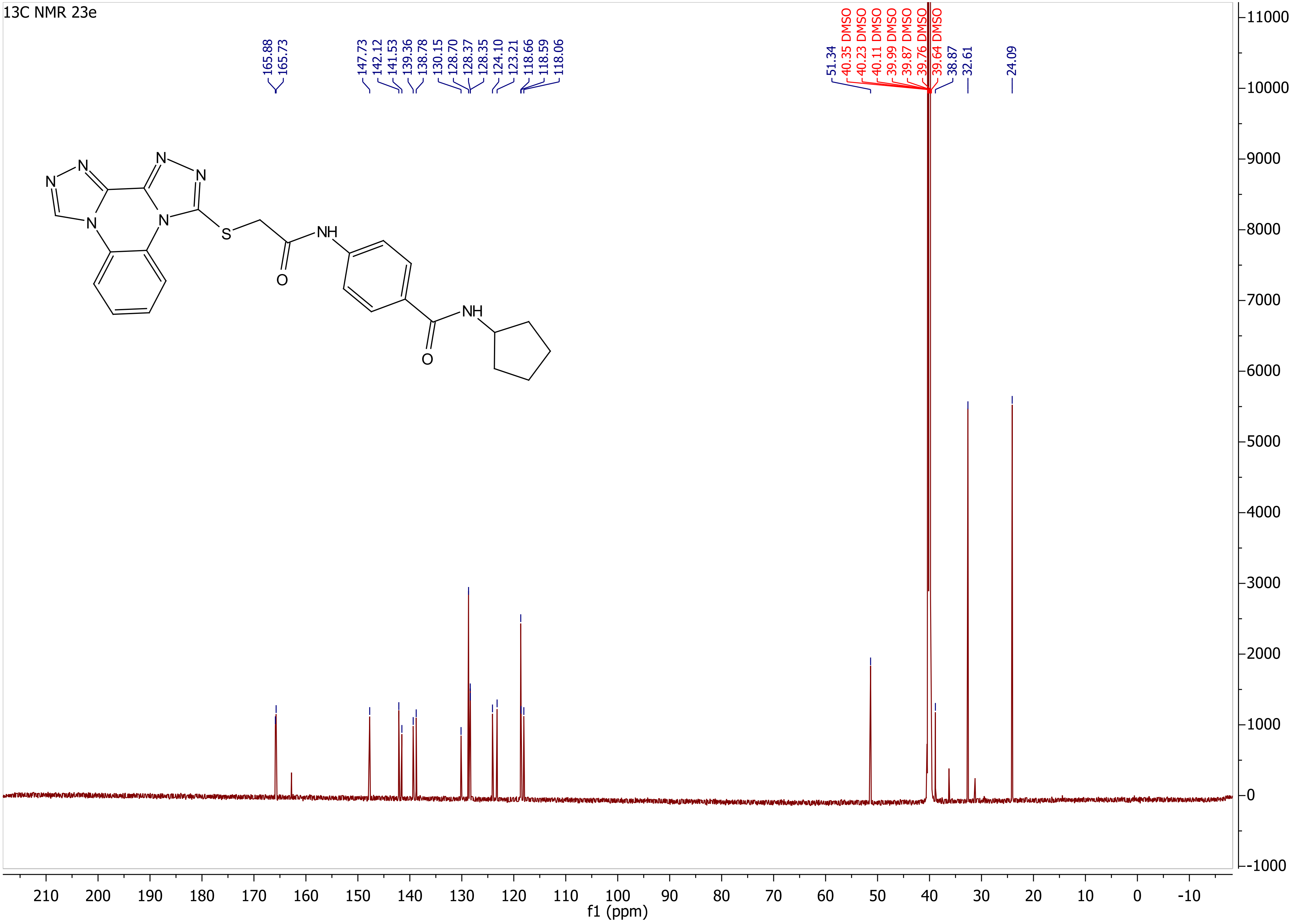

13C NMR 23e
165.88
165.73
147.73
142.12
141.53
139.36
138.78
130.15
128.70
128.37
128.35
124.10
123.21
118.66
118.59
118.06
51.34
40.35 DMSO
40.23 DMSO
40.11 DMSO
39.99 DMSO
39.87 DMSO
39.76 DMSO
39.64 DMSO
38.87
32.61
24.09
N
N
N
N
N
N
S
NH
O
NH
O
f1 (ppm)

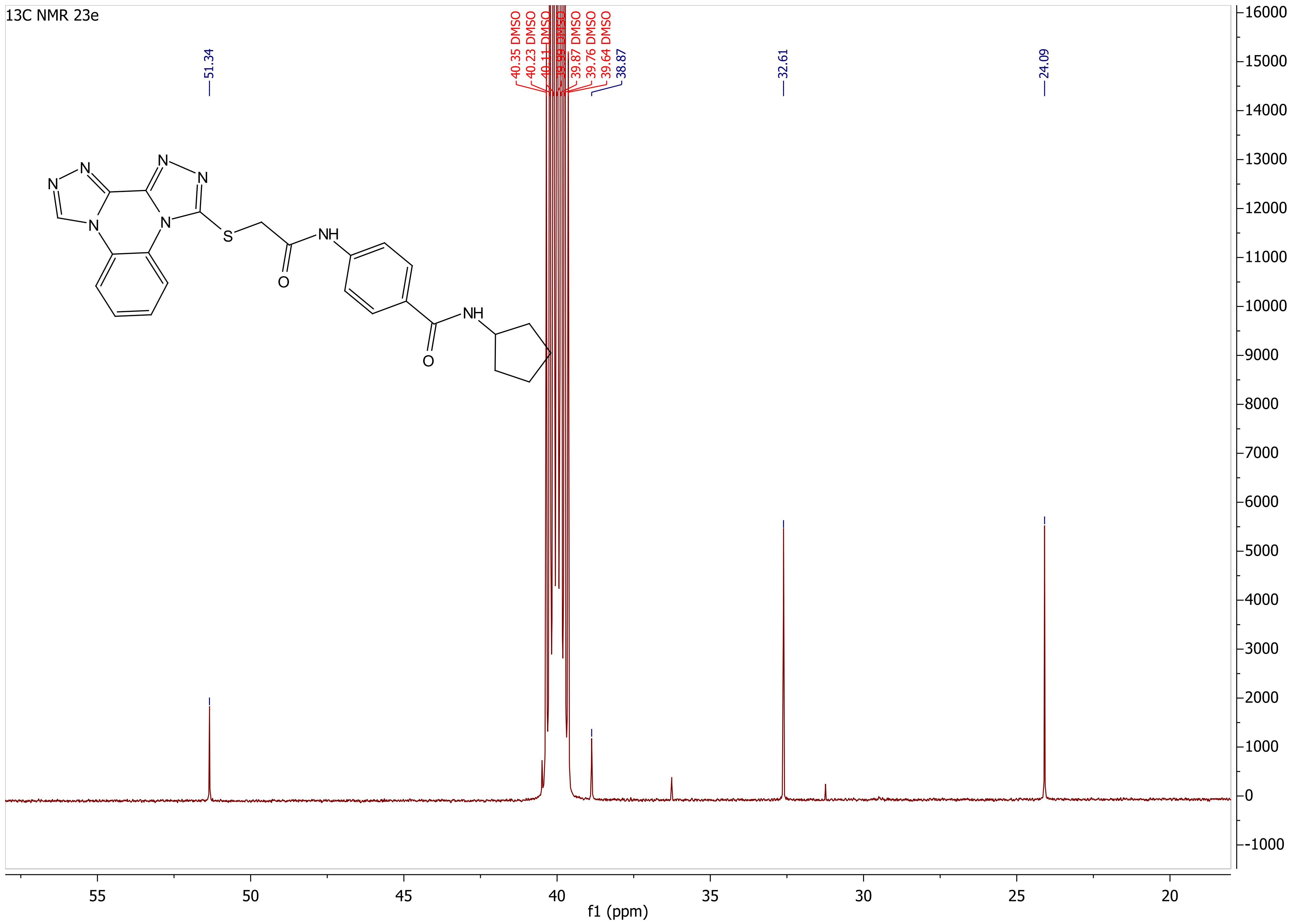
13C NMR 23e
51.34
40.35 DMSO
40.23 DMSO
40.11 DMSO
39.99 DMSO
39.87 DMSO
39.76 DMSO
39.64 DMSO
38.87
32.61
24.09
f1 (ppm)

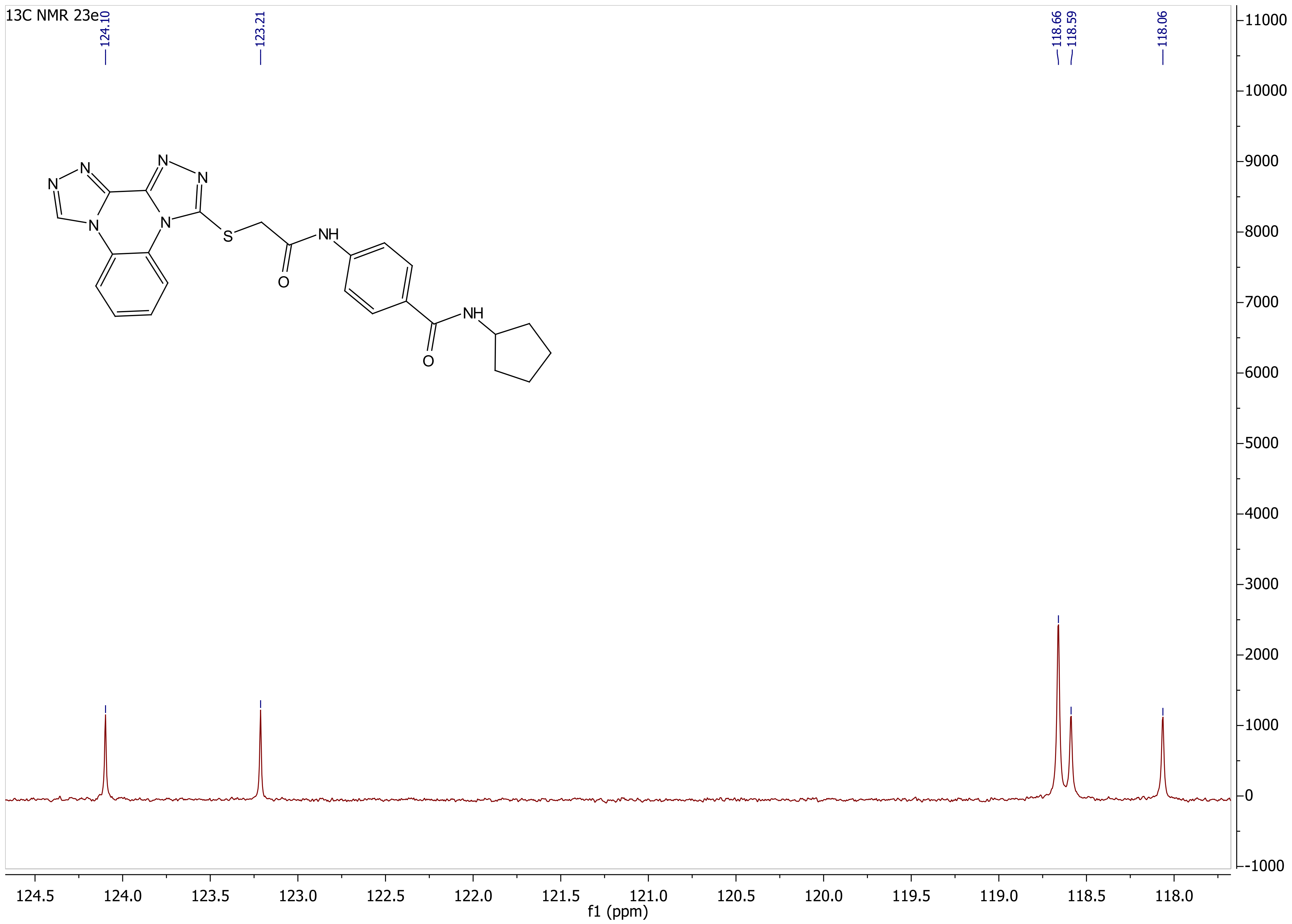

13C NMR 23e
124.10
123.21
118.66
118.59
118.06
11000
10000
9000
8000
7000
6000
5000
4000
3000
2000
1000
0
-1000
124.5
124.0
123.5
123.0
122.5
122.0
121.5
121.0
120.5
120.0
119.5
119.0
118.5
118.0
f1 (ppm)

13C NMR 23e

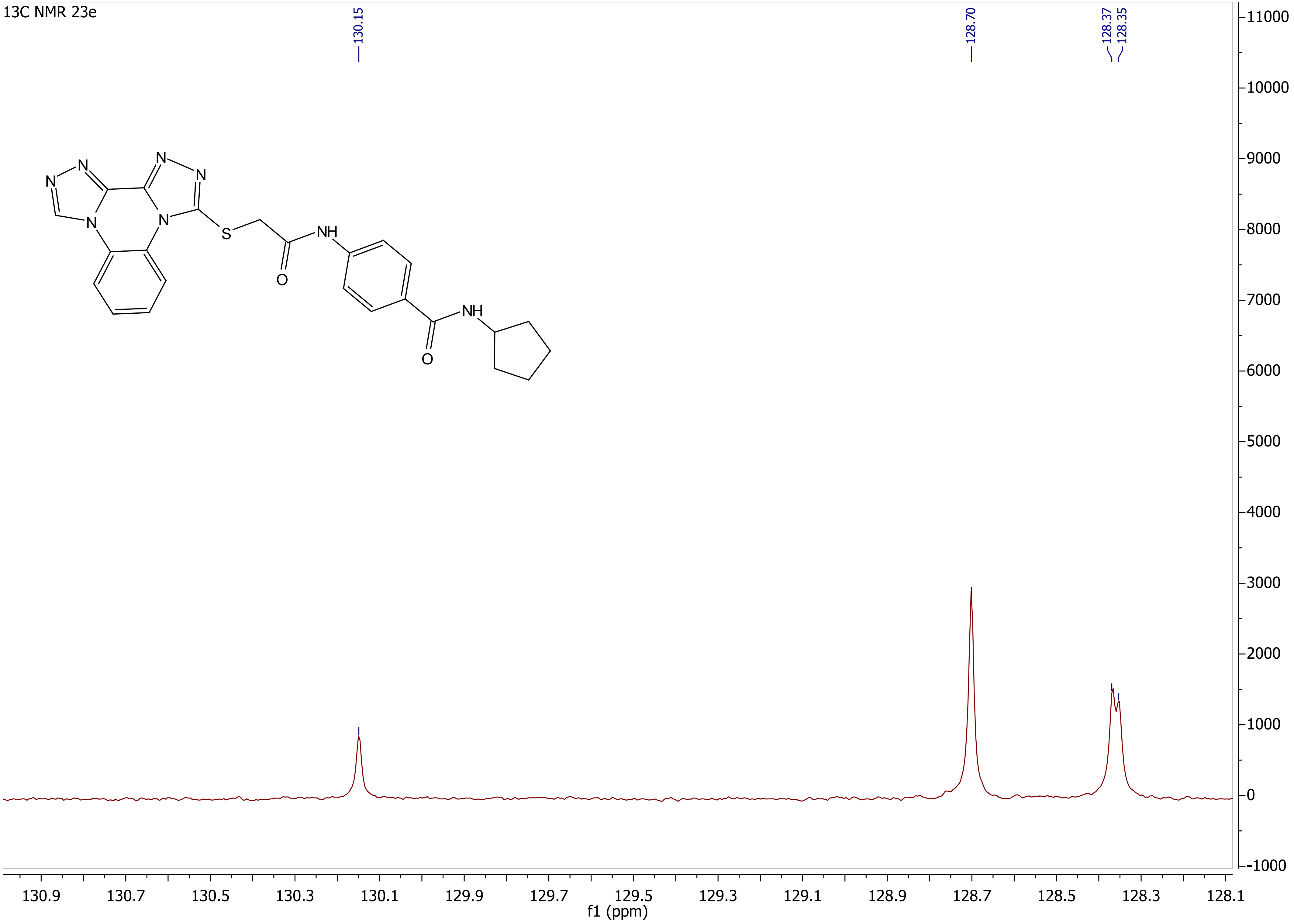
130.15
128.70
128.37
128.35
11000
10000
9000
8000
7000
6000
5000
4000
3000
2000
1000
0
-1000
130.9
130.7
130.5
130.3
130.1
129.9
129.7
129.5
129.3
129.1
128.9
128.7
128.5
128.3
128.1
f1 (ppm)

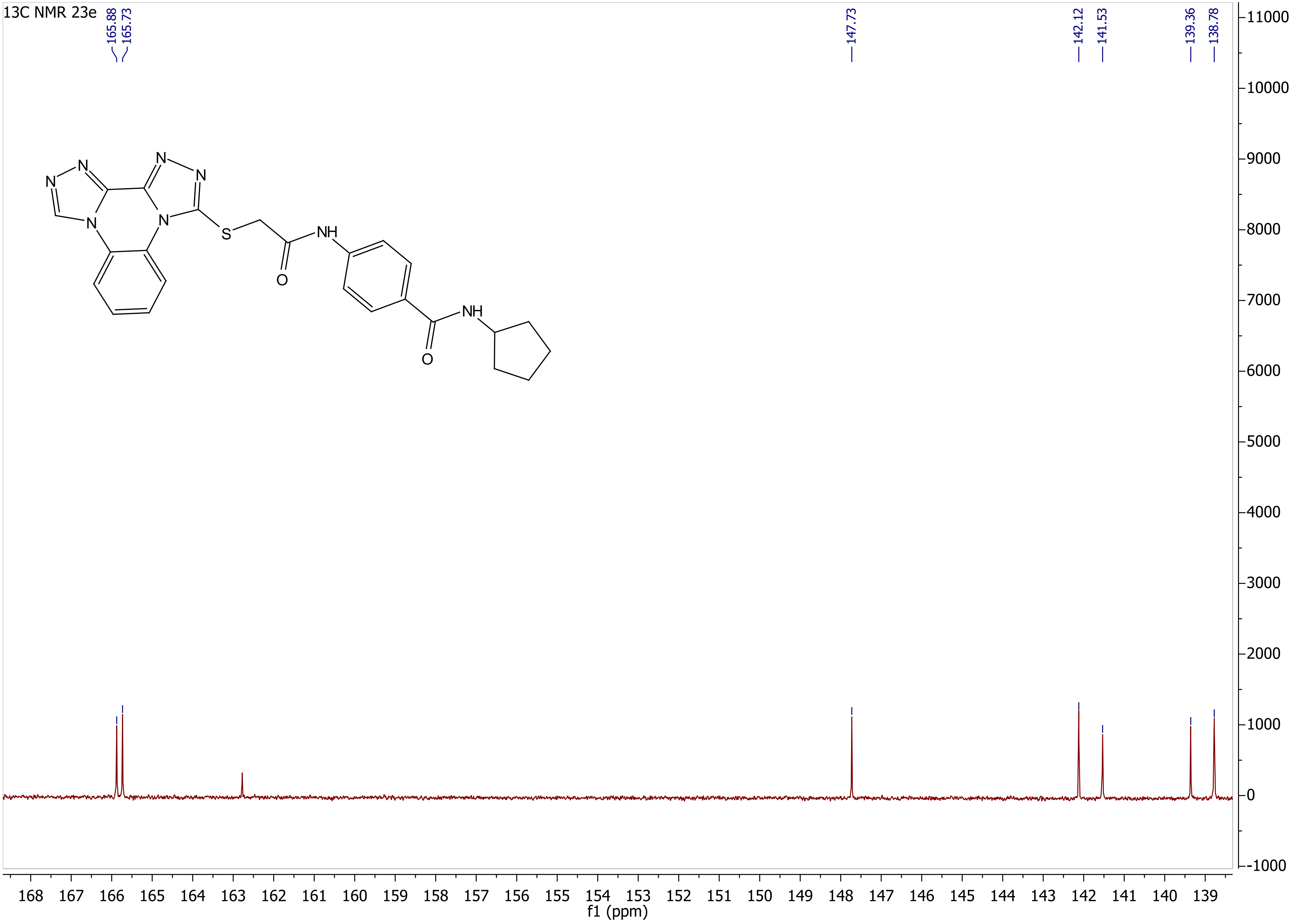

13C NMR 23e
165.88
165.73
147.73
142.12
141.53
139.36
138.78
N
N
N
N
N
N
S
NH
O
NH
O
11000
10000
9000
8000
7000
6000
5000
4000
3000
2000
1000
0
-1000
168 167 166 165 164 163 162 161 160 159 158 157 156 155 154 153 152 151 150 149 148 147 146 145 144 143 142 141 140 139
f1 (ppm)

Mass spec. of 23e

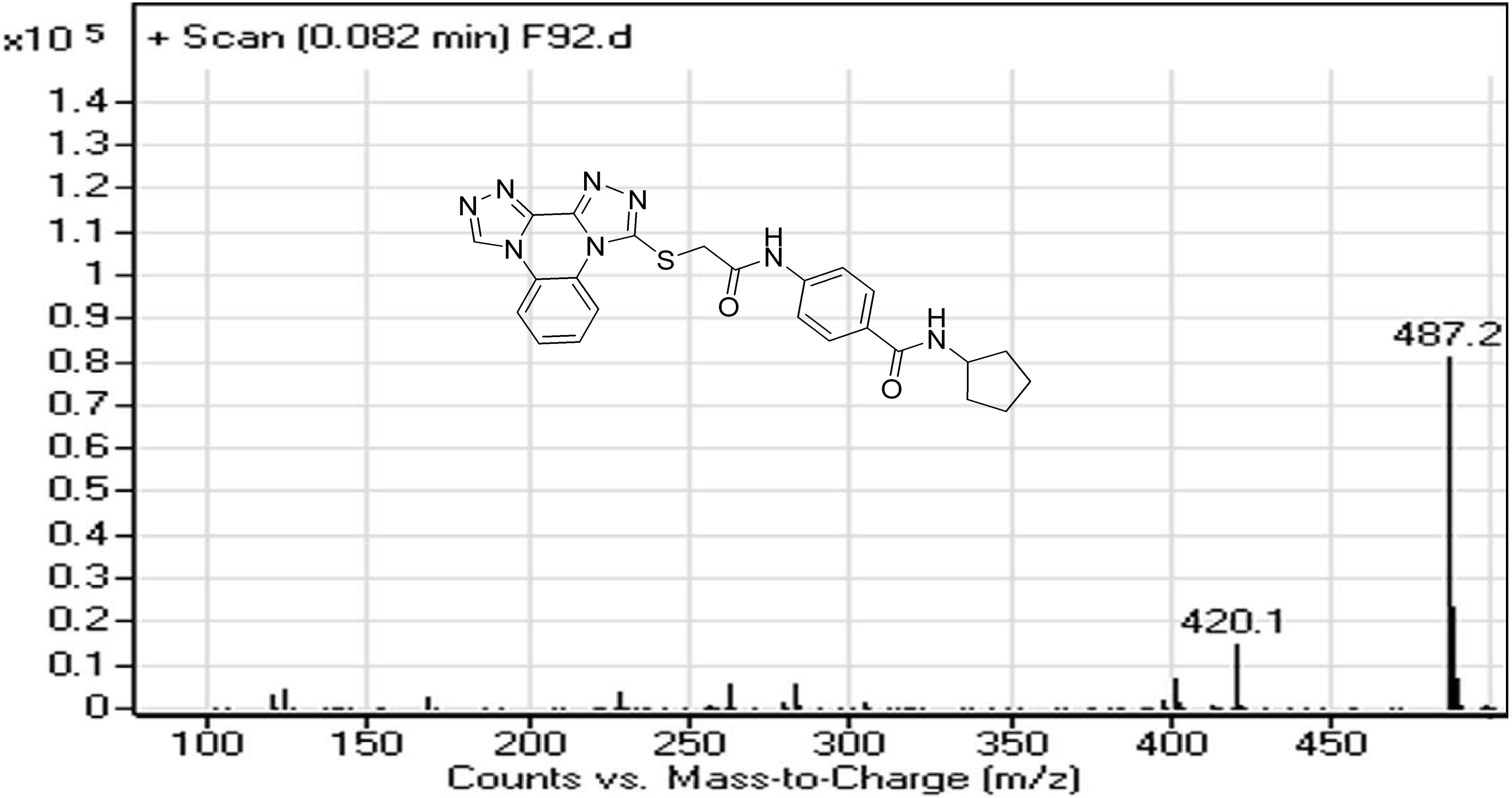

IR of compound 23f

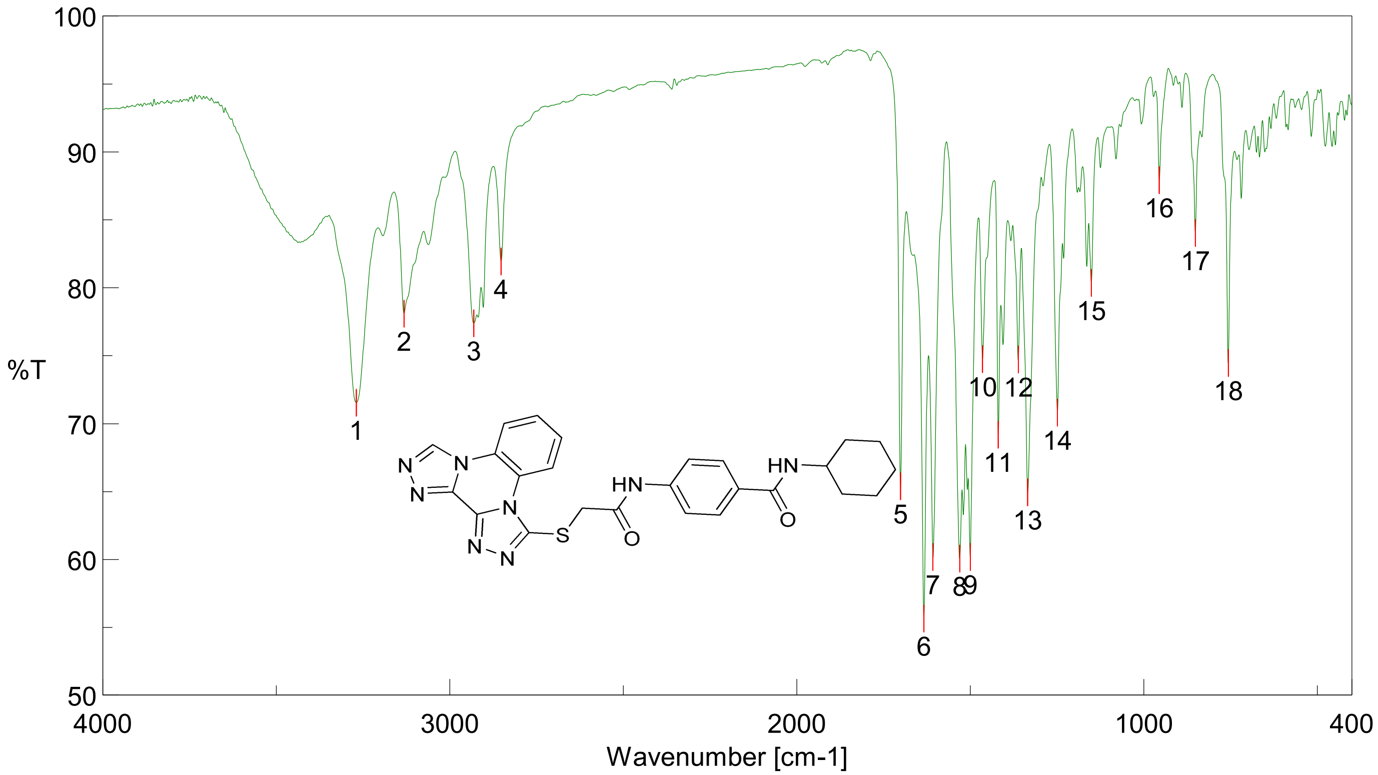

[Comments]

Sample name	F82
Comment	
User	
Division	
Company	KSU

[Detailed Information]

Creation date	10/29/2020 5:08 AM
Data array type	Linear data array
Horizontal axis	Wavenumber [cm-1]
Vertical axis	%T
Start	399.193 cm-1
End	4000.6 cm-1
Data interval	0.964233 cm-1
Data points	3736

[Measurement Information]

Model Name	FT/IR-6600typeA
Serial Number	A014661790
Measurement Date	10/28/2020 4:04 AM
Light Source	Standard
Detector	TGS
Accumulation	Auto (14)
Resolution	4 cm-1
Zero Filling	On
Apodization	Cosine
Gain	Auto (1)
Aperture	Auto (7.1 mm)
Scanning Speed	Auto (2 mm/sec)
Filter	Auto (10000 Hz)

[Result of Peak Picking]

No.	Position	Intensity	No.	Position	Intensity	No.	Position	Intensity
1	3269.72	71.5274	2	3131.83	78.0848	3	2930.31	77.362

[Result of Peak Picking]

No.	Position	Intensity	No.	Position	Intensity	No.	Position	Intensity
4	2852.2	81.9284	5	1700.91	65.3706	6	1633.41	55.6271
7	1607.38	60.1527	8	1530.24	60.051	9	1499.38	60.162
10	1464.67	74.7309	11	1419.35	69.1623	12	1361.5	74.6999
13	1334.5	64.9269	14	1248.68	70.7969	15	1151.29	80.3414
16	955.555	87.9139	17	851.418	84.0057	18	755.959	74.4502

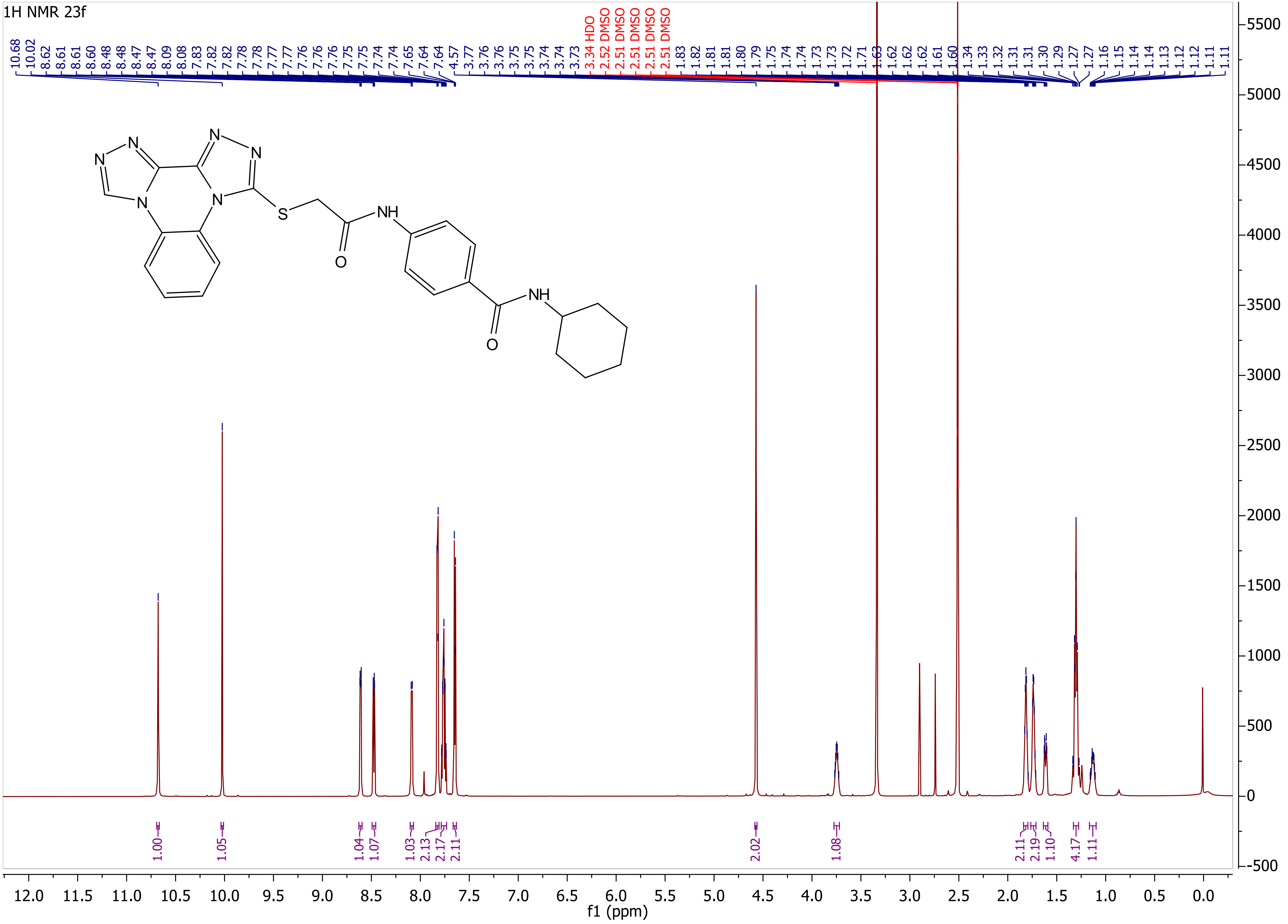
1H NMR 23f
10.68
10.02
8.62
8.61
8.61
8.60
8.48
8.48
8.47
8.47
8.09
8.08
7.83
7.82
7.82
7.78
7.77
7.77
7.76
7.76
7.75
7.75
7.74
7.74
7.65
7.64
7.64
4.57
3.77
3.76
3.76
3.75
3.75
3.74
3.74
3.73
3.34 HDO
2.52 DMSO
2.51 DMSO
2.51 DMSO
2.51 DMSO
2.51 DMSO
1.83
1.82
1.81
1.81
1.80
1.79
1.75
1.74
1.74
1.73
1.73
1.72
1.71
1.63
1.62
1.62
1.61
1.60
1.34
1.33
1.32
1.31
1.31
1.30
1.29
1.27
1.27
1.16
1.15
1.14
1.14
1.13
1.12
1.11
1.11
1.00
1.05
1.04
1.07
1.03
2.13
2.17
2.11
2.02
1.08
2.11
2.19
1.10
4.17
1.11
f1 (ppm)

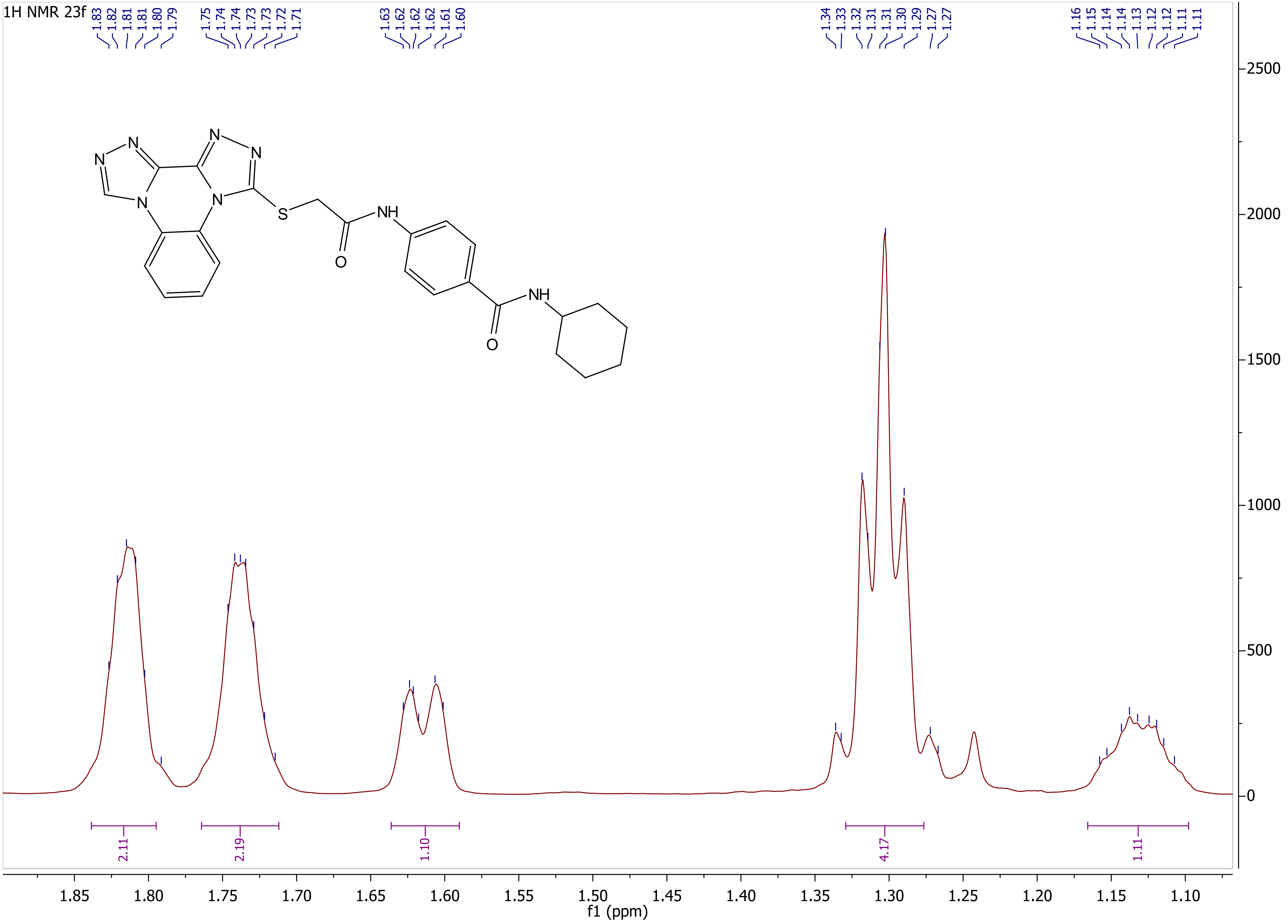

1H NMR 23f
f1 (ppm)

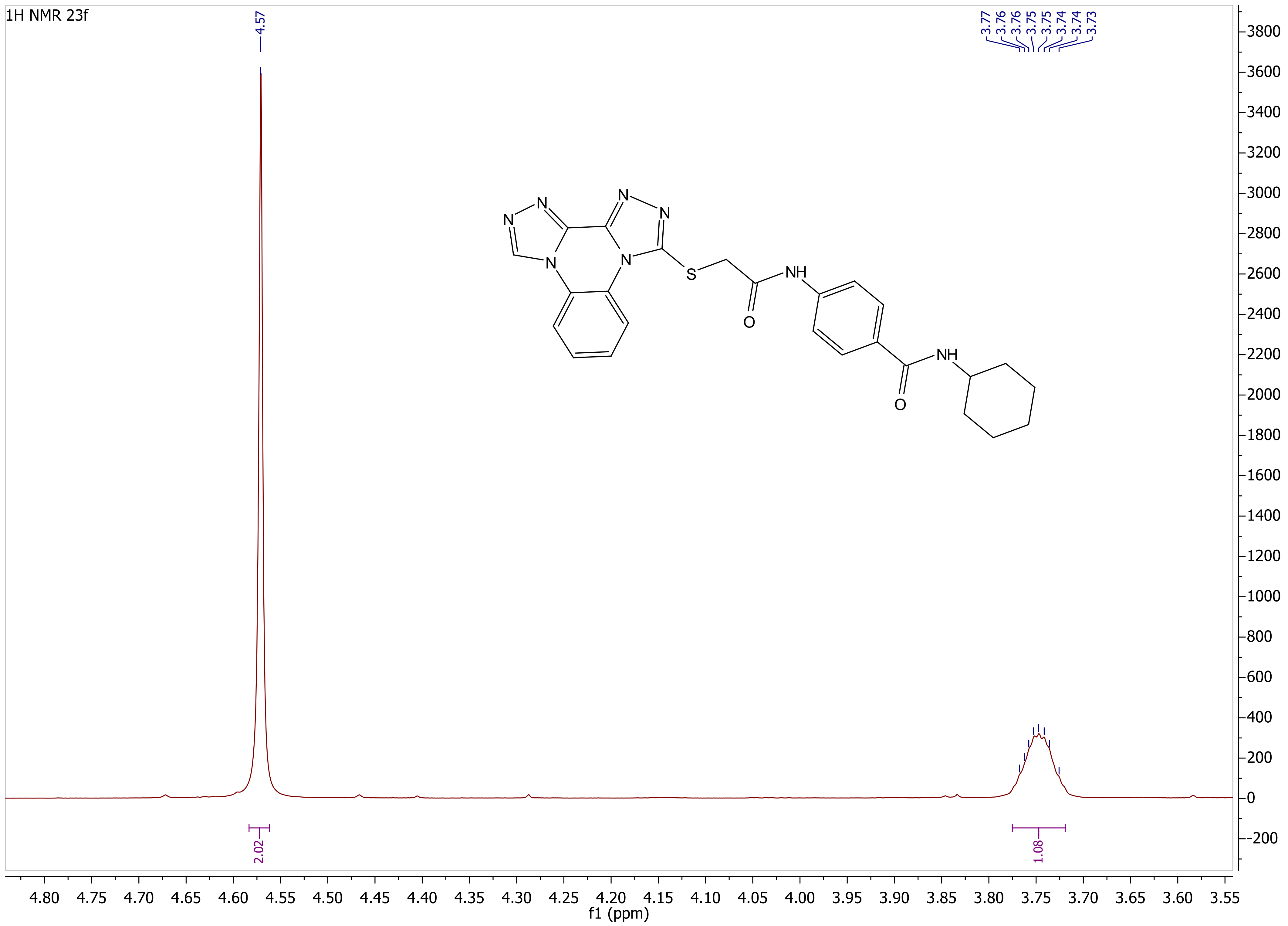

1H NMR 23f
4.57
3.77
3.76
3.76
3.75
3.75
3.74
3.74
3.73
2.02
1.08
f1 (ppm)

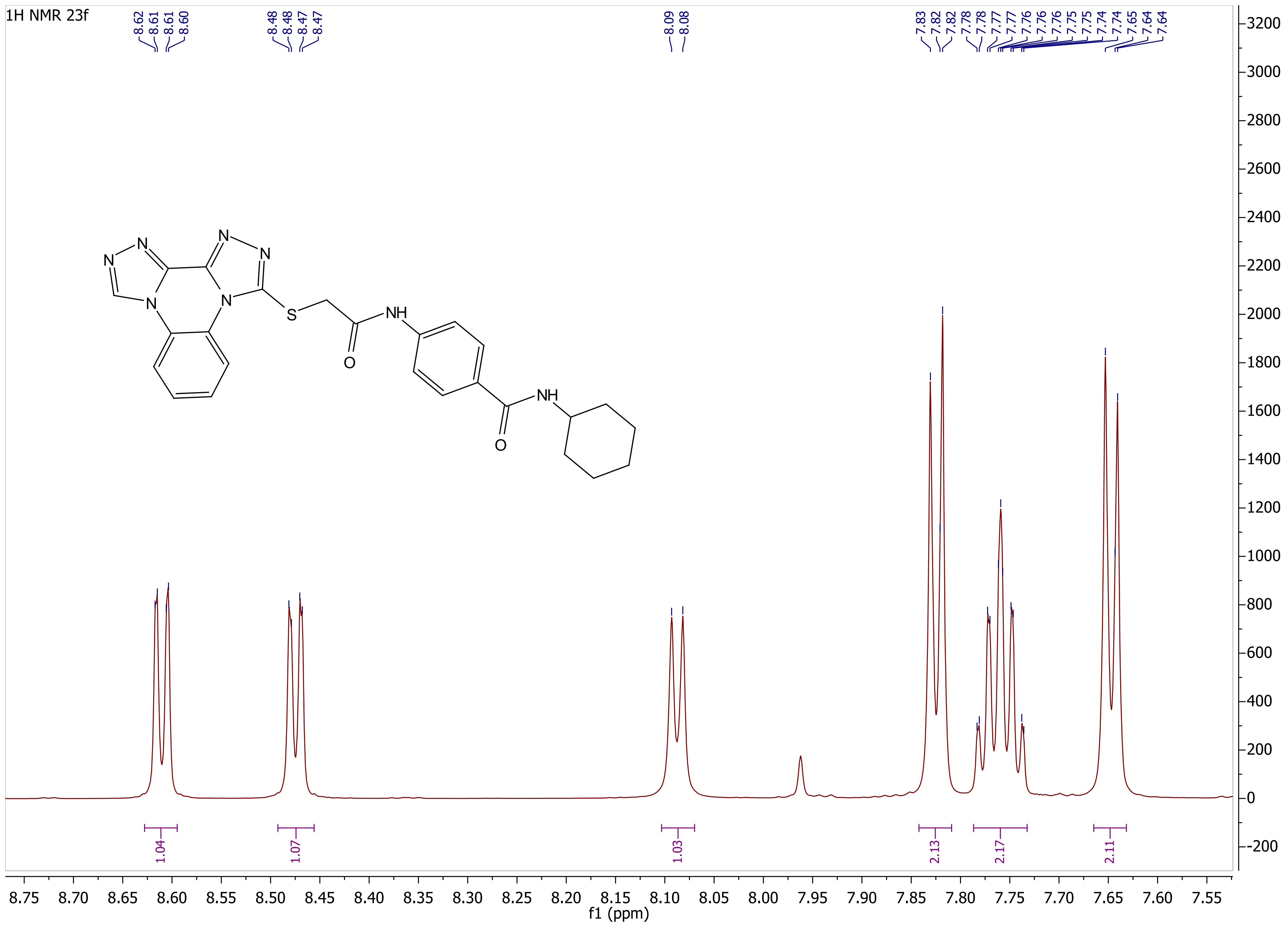
1H NMR 23f
8.62
8.61
8.61
8.60
8.48
8.48
8.47
8.47
8.09
8.08
7.83
7.82
7.82
7.78
7.78
7.77
7.77
7.76
7.76
7.76
7.75
7.75
7.74
7.74
7.65
7.64
7.64
1.04
1.07
1.03
2.13
2.17
2.11
f1 (ppm)

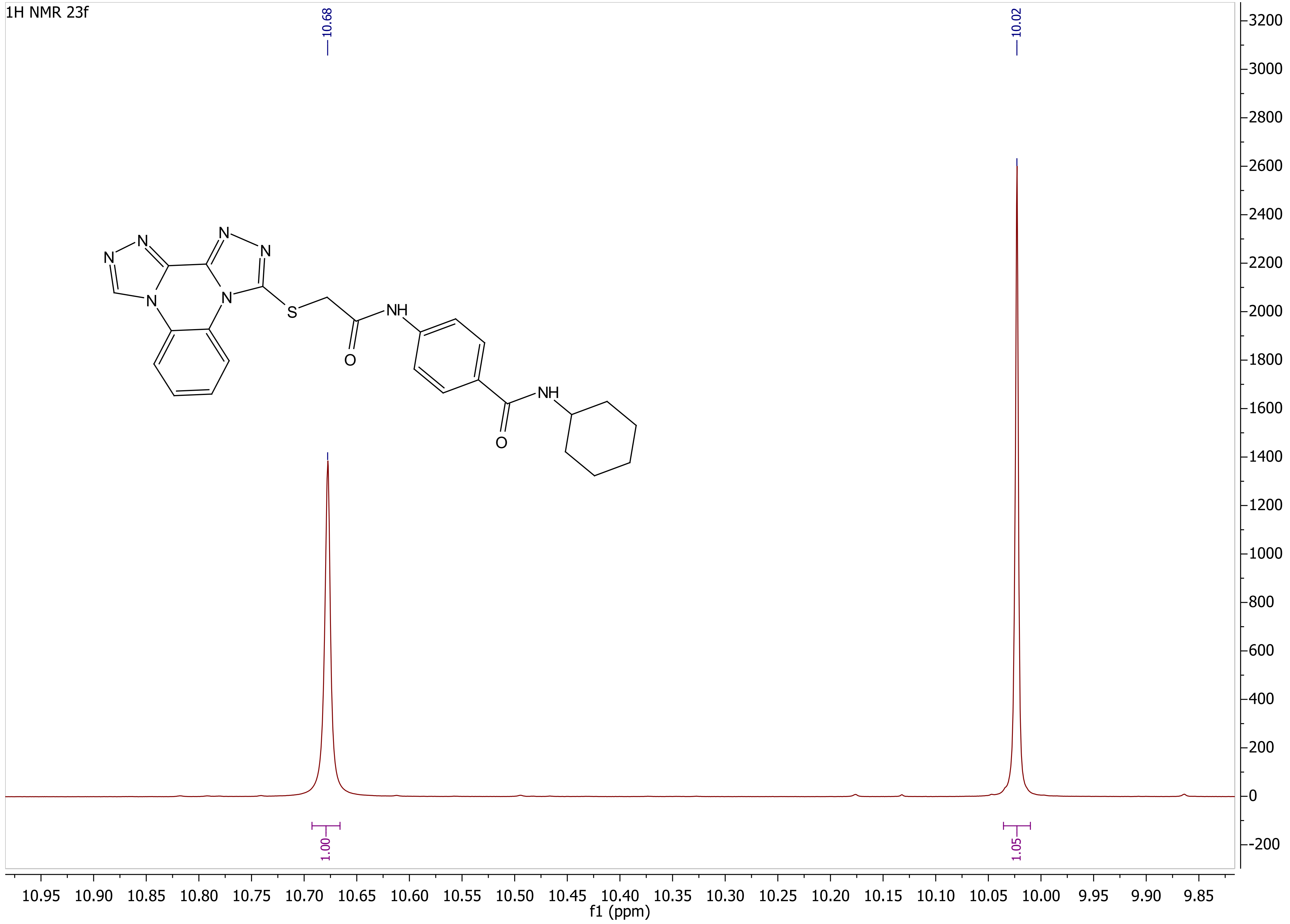

1H NMR 23f
10.68
10.02
1.00
1.05
f1 (ppm)
10.95 10.90 10.85 10.80 10.75 10.70 10.65 10.60 10.55 10.50 10.45 10.40 10.35 10.30 10.25 10.20 10.15 10.10 10.05 10.00 9.95 9.90 9.85
3200 3000 2800 2600 2400 2200 2000 1800 1600 1400 1200 1000 800 600 400 200 0 -200
N
N
N
N
N
N
S
NH
O
NH
O

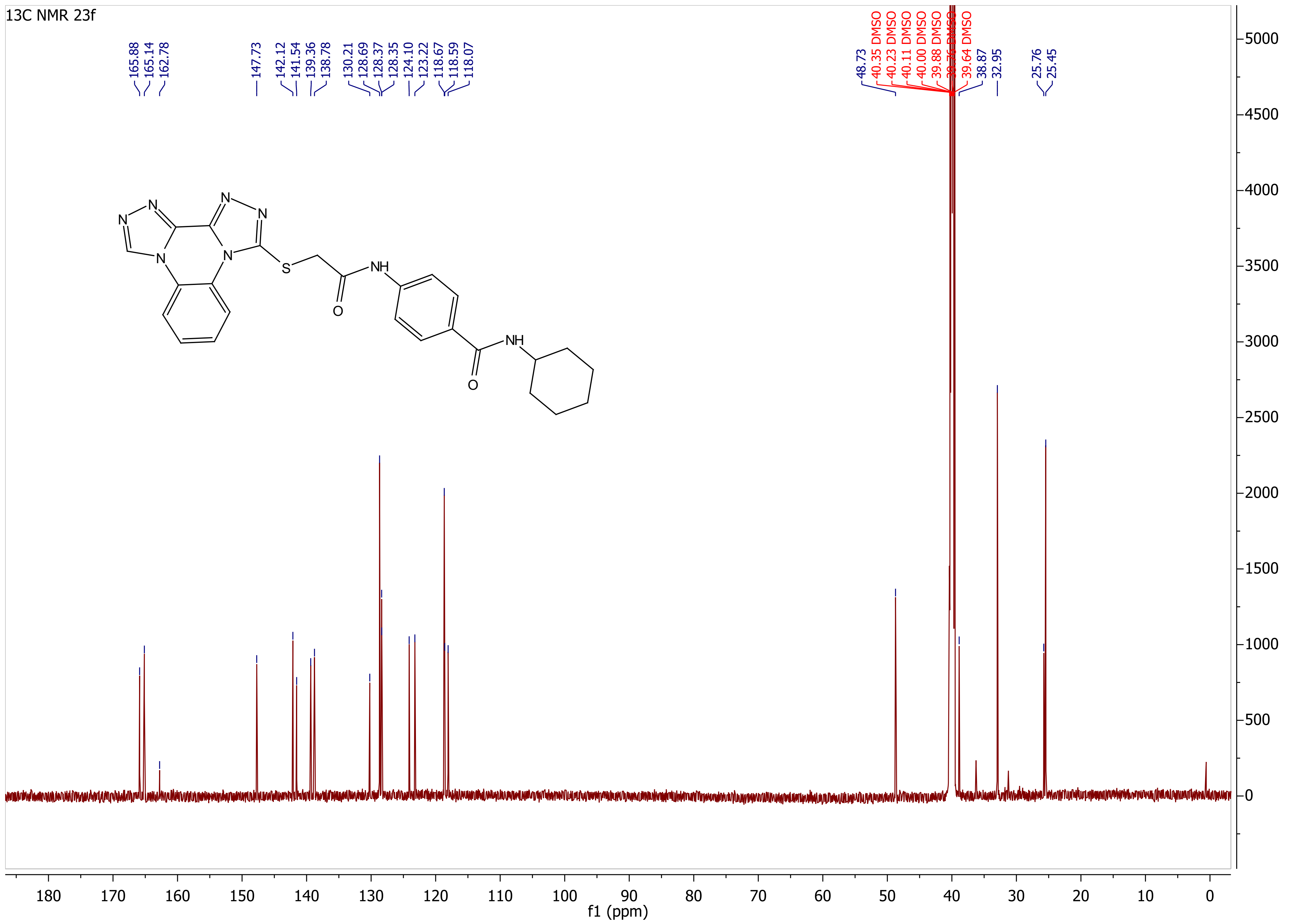
13C NMR 23f
165.88
165.14
162.78
147.73
142.12
141.54
139.36
138.78
130.21
128.69
128.37
128.35
124.10
123.22
118.67
118.59
118.07
48.73
40.35 DMSO
40.23 DMSO
40.11 DMSO
40.00 DMSO
39.88 DMSO
39.76 DMSO
39.64 DMSO
38.87
32.95
25.76
25.45
N
N
N
N
N
N
S
NH
O
NH
O
f1 (ppm)

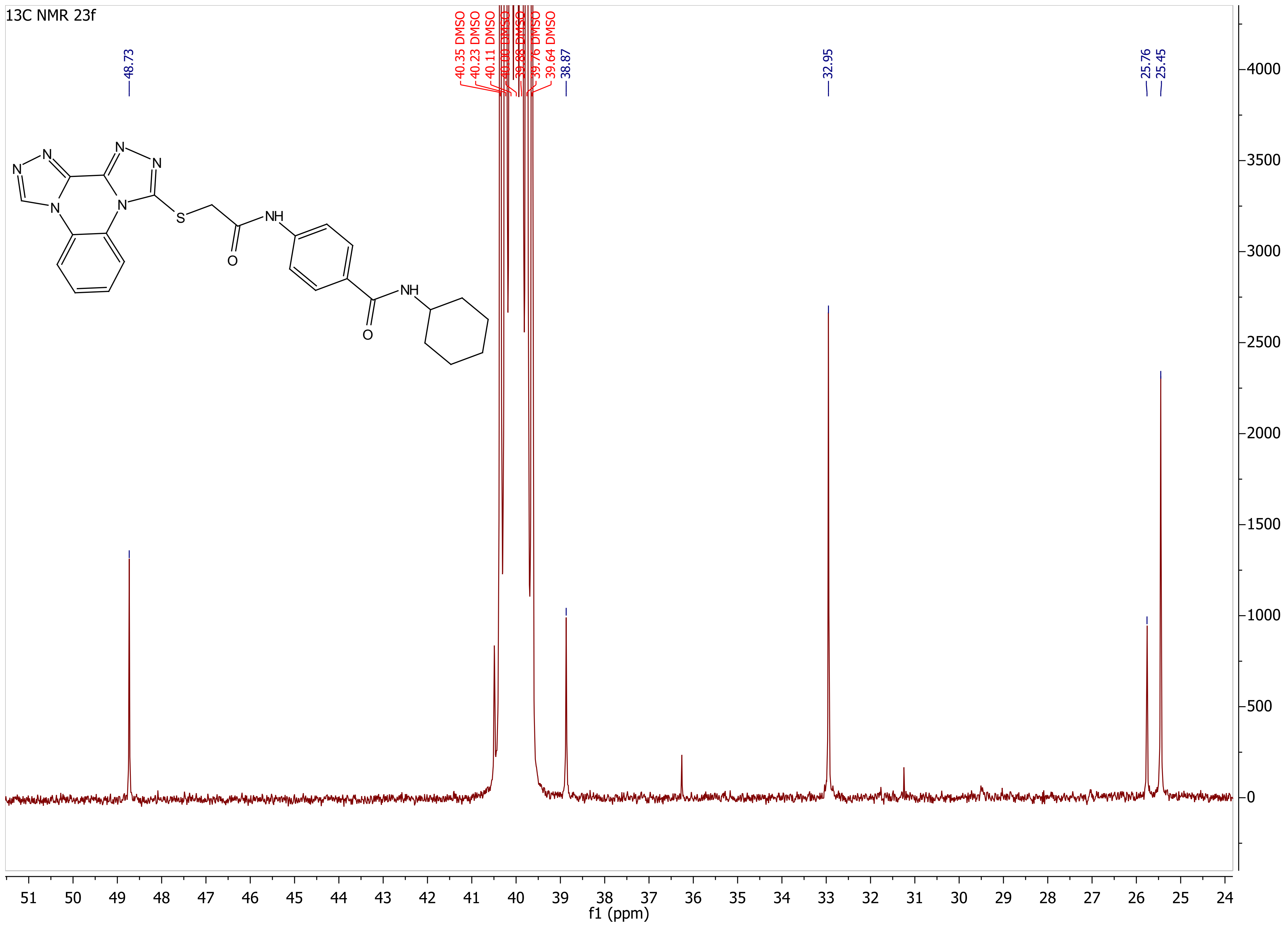

13C NMR 23f
48.73
40.35 DMSO
40.23 DMSO
40.11 DMSO
40.00 DMSO
39.88 DMSO
39.76 DMSO
39.64 DMSO
38.87
32.95
25.76
25.45
f1 (ppm)

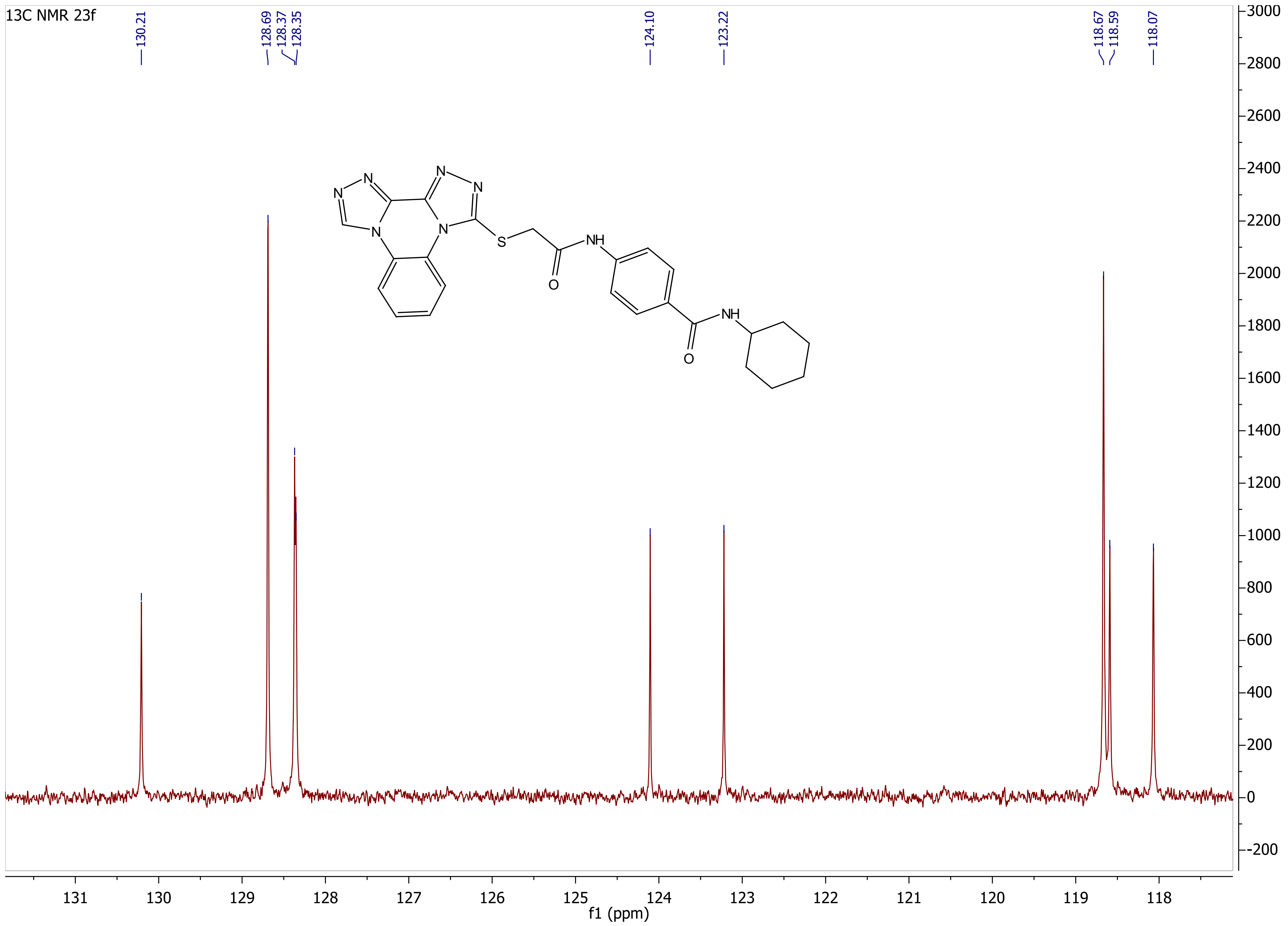
13C NMR 23f
130.21
128.69
128.37
128.35
124.10
123.22
118.67
118.59
118.07
N
N
N
N
N
N
S
NH
O
NH
O
f1 (ppm)
131
130
129
128
127
126
125
124
123
122
121
120
119
118
3000
2800
2600
2400
2200
2000
1800
1600
1400
1200
1000
800
600
400
200
0
-200

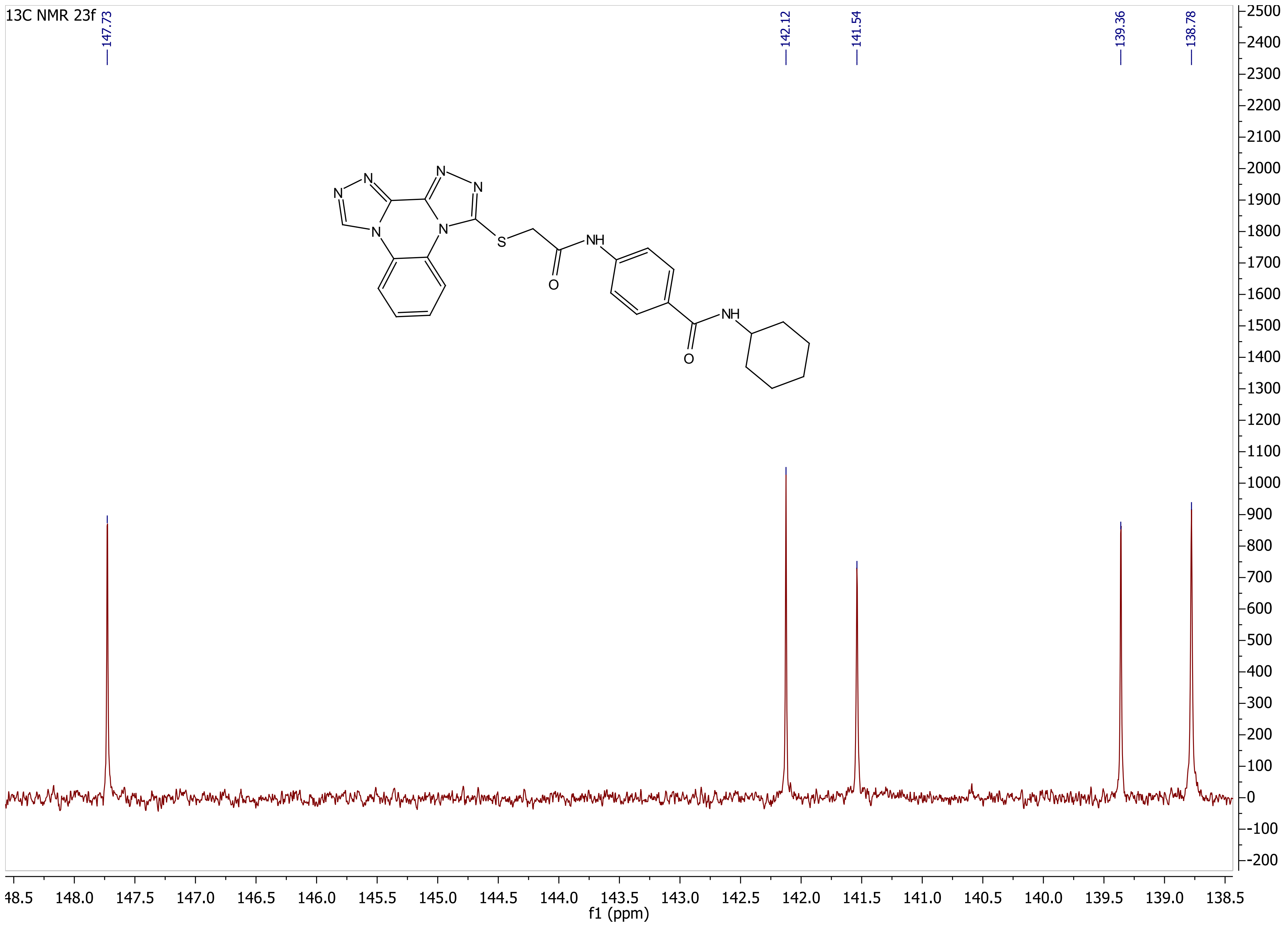
13C NMR 23f
147.73
142.12
141.54
139.36
138.78
N
N
N
N
N
N
S
NH
O
NH
O
f1 (ppm)

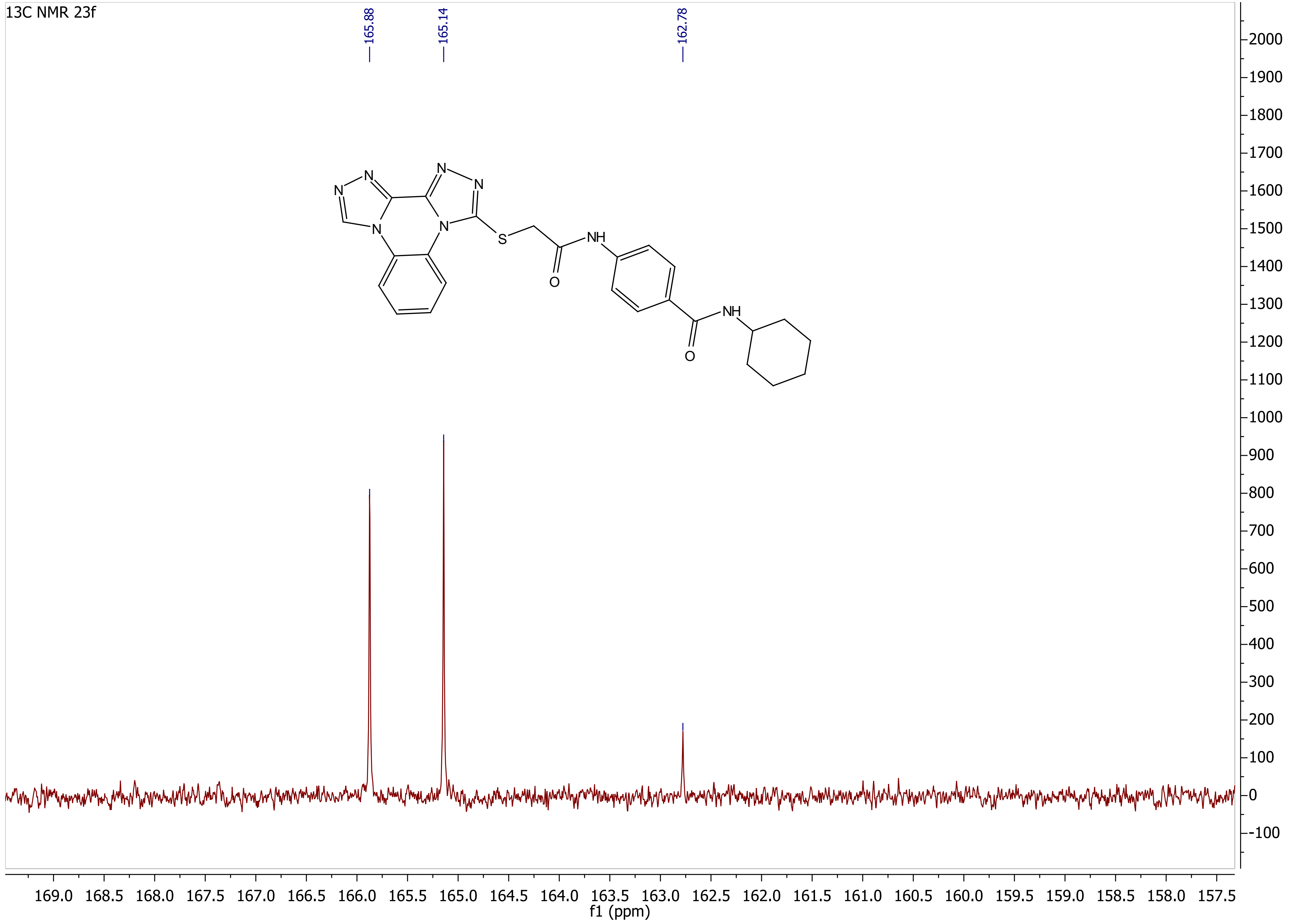
13C NMR 23f
165.88
165.14
162.78
N
N
N
N
N
N
S
NH
O
NH
O
2000
1900
1800
1700
1600
1500
1400
1300
1200
1100
1000
900
800
700
600
500
400
300
200
100
0
-100
169.0 168.5 168.0 167.5 167.0 166.5 166.0 165.5 165.0 164.5 164.0 163.5 163.0 162.5 162.0 161.5 161.0 160.5 160.0 159.5 159.0 158.5 158.0 157.5
f1 (ppm)

Mass spec. of 23f

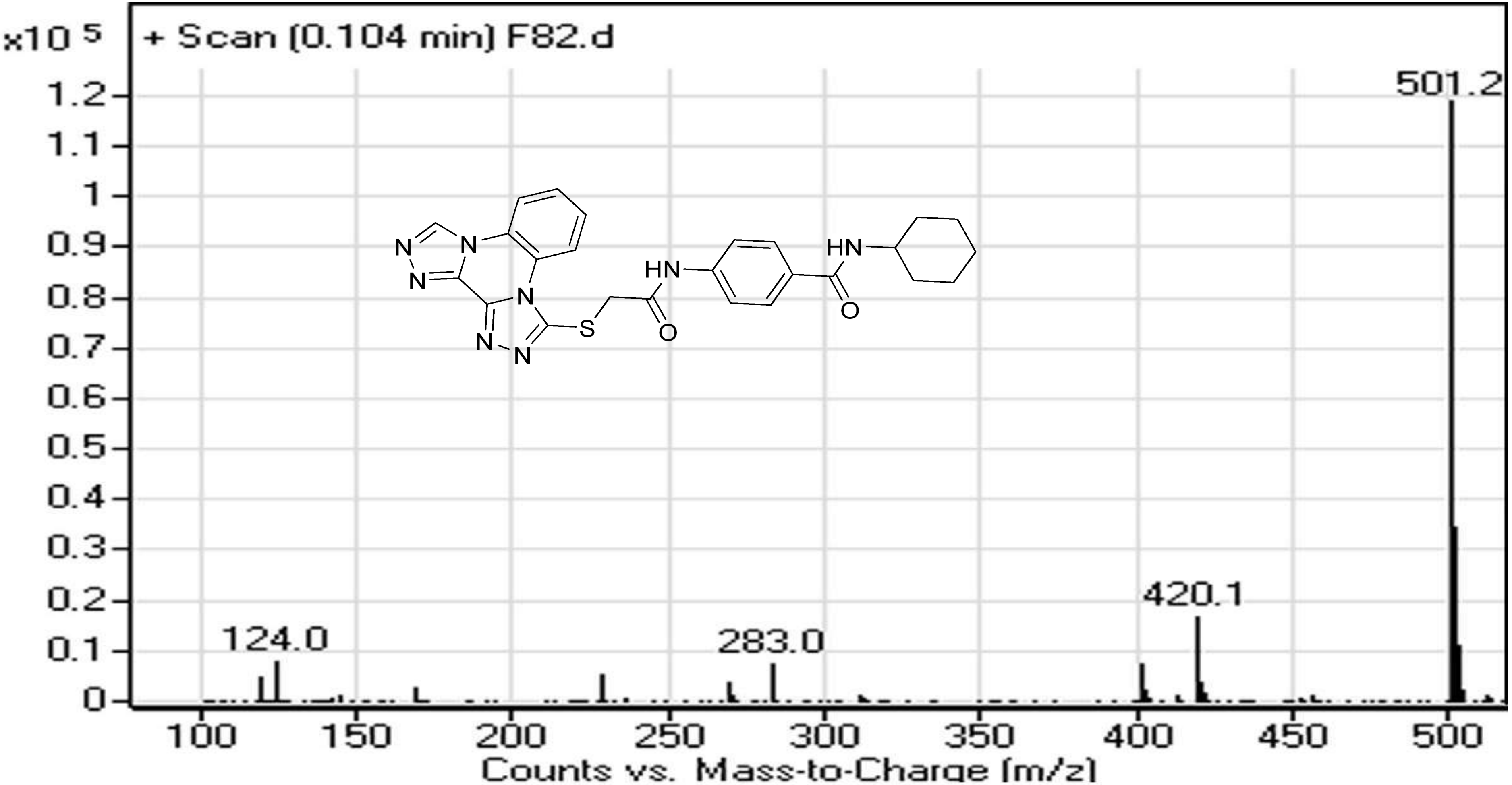

IR of compound 23g

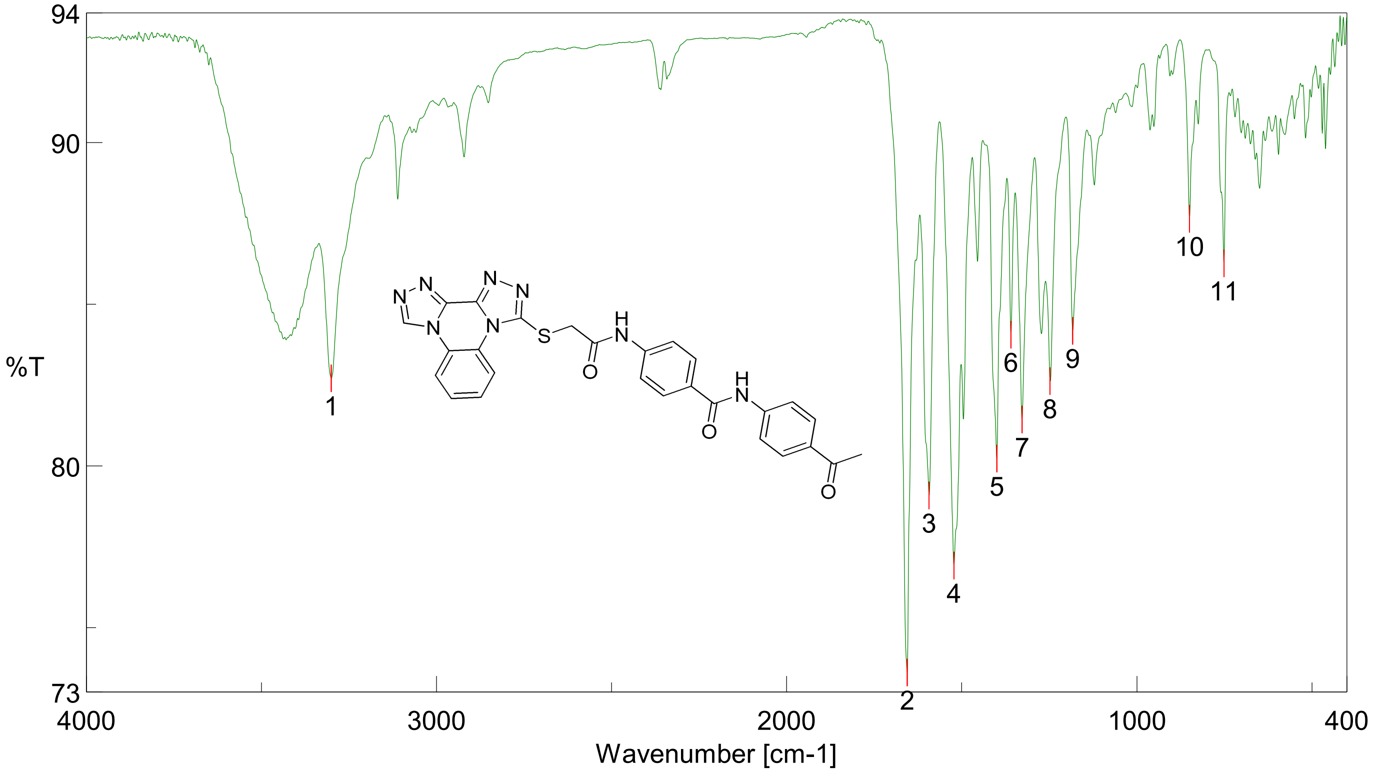

[Comments]

Sample name	F81
Comment	
User	
Division	
Company	KSU

[Detailed Information]

Creation date	10/29/2020 5:07 AM
Data array type	Linear data array
Horizontal axis	Wavenumber [cm-1]
Vertical axis	%T
Start	399.193 cm-1
End	4000.6 cm-1
Data interval	0.964233 cm-1
Data points	3736

[Measurement Information]

Model Name	FT/IR-6600typeA
Serial Number	A014661790
Measurement Date	10/28/2020 4:02 AM
Light Source	Standard
Detector	TGS
Accumulation	Auto (13)
Resolution	4 cm-1
Zero Filling	On
Apodization	Cosine
Gain	Auto (1)
Aperture	Auto (7.1 mm)
Scanning Speed	Auto (2 mm/sec)
Filter	Auto (10000 Hz)

[Result of Peak Picking]

No.	Position	Intensity	No.	Position	Intensity	No.	Position	Intensity
1	3300.57	82.7032	2	1655.59	73.5996	3	1593.88	79.0809

[Result of Peak Picking]

No.	Position	Intensity	No.	Position	Intensity	No.	Position	Intensity
4	1522.52	76.9078	5	1400.07	80.2169	6	1359.57	84.0464
7	1327.75	81.4258	8	1247.72	82.61	9	1183.11	84.1726
10	849.49	87.6317	11	751.138	86.2595			

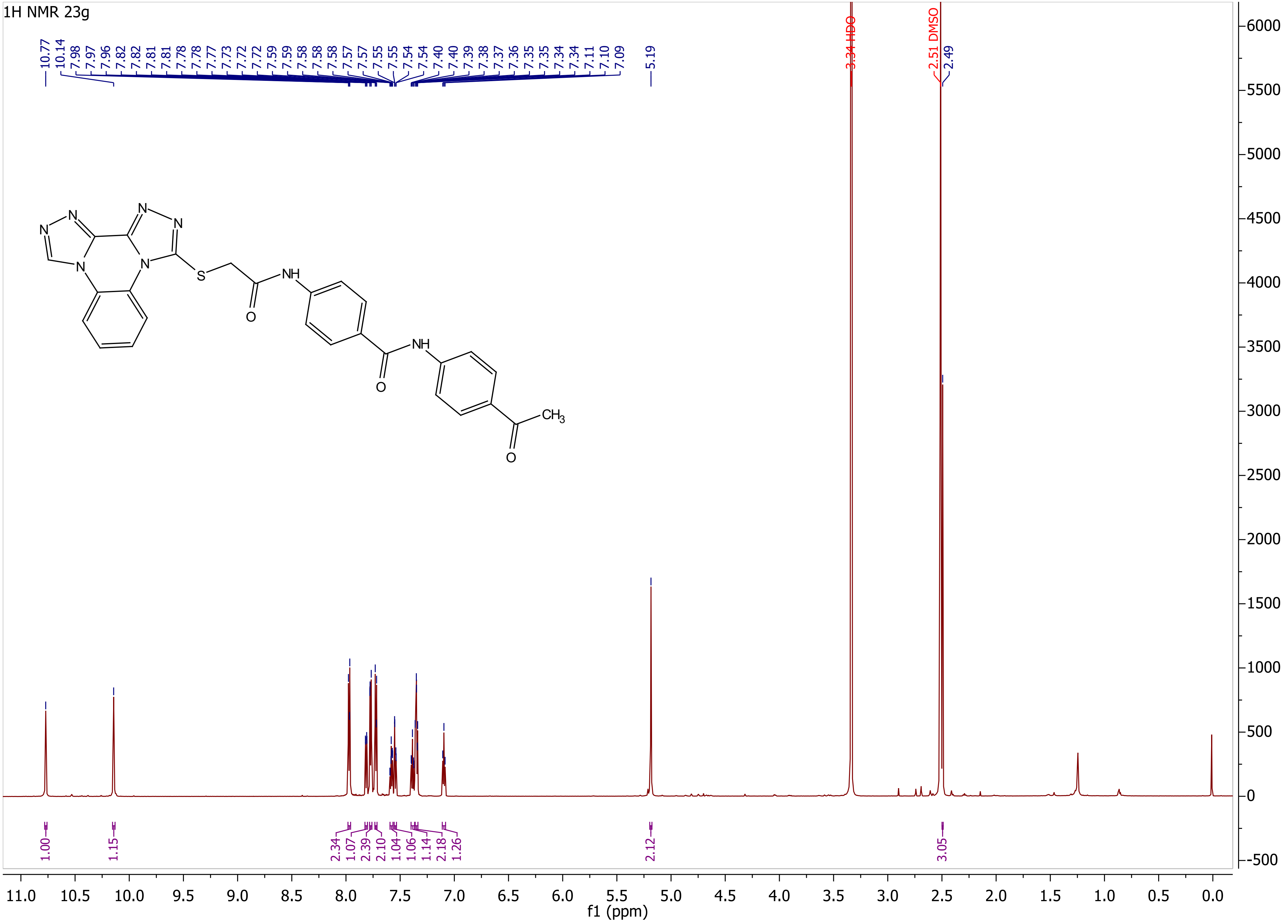
1H NMR 23g
10.77
10.14
7.98
7.97
7.96
7.82
7.82
7.81
7.81
7.78
7.78
7.77
7.73
7.72
7.72
7.59
7.59
7.58
7.58
7.58
7.57
7.57
7.55
7.55
7.54
7.54
7.40
7.40
7.39
7.38
7.37
7.36
7.35
7.35
7.34
7.34
7.11
7.10
7.09
5.19
3.34 HDO
2.51 DMSO
2.49
N
N
N
N
N
N
S
NH
O
NH
O
CH3
O
1.00
1.15
2.34
1.07
2.39
2.10
1.04
1.06
1.14
2.18
1.26
2.12
3.05
f1 (ppm)

1H NMR 23g

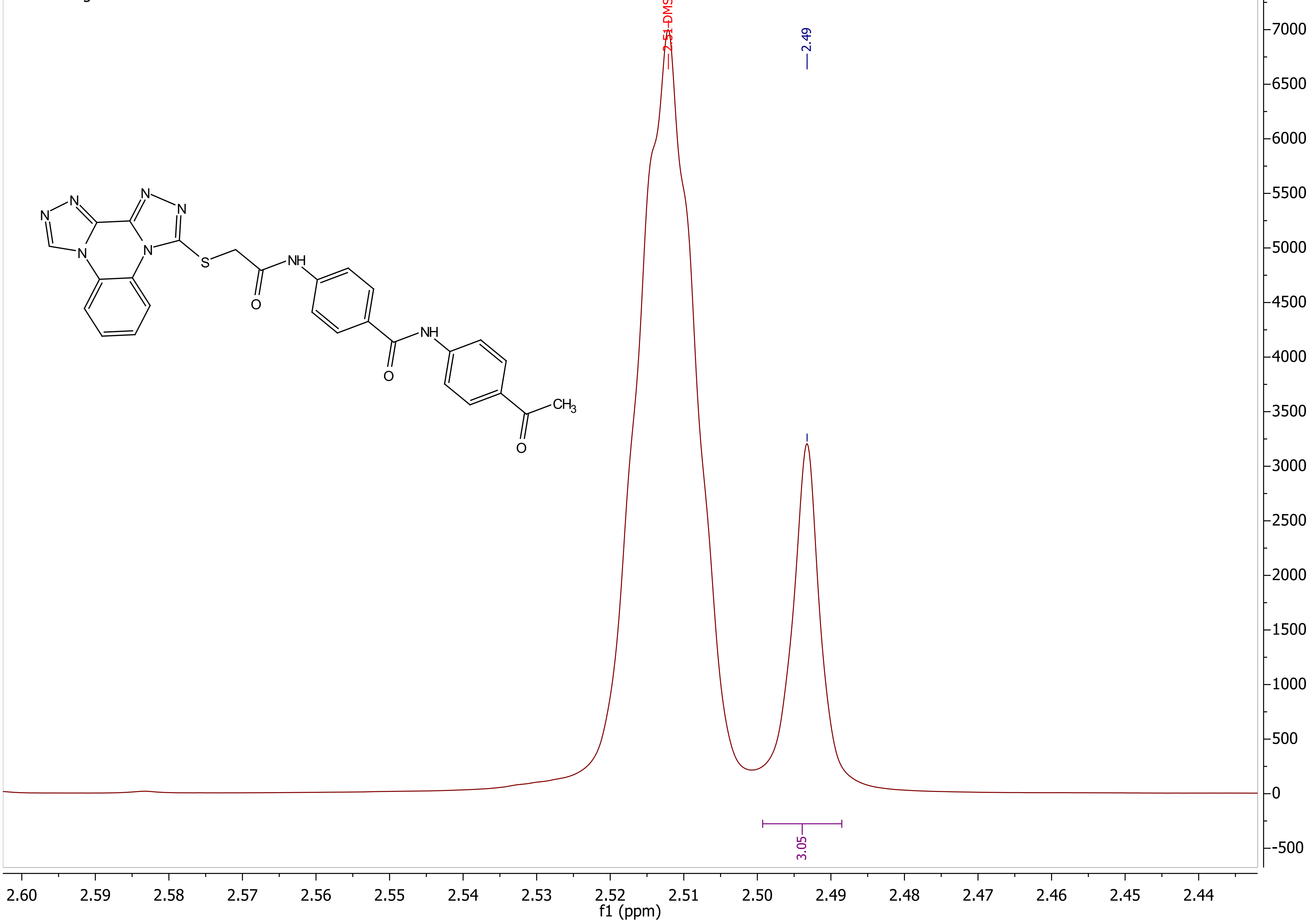

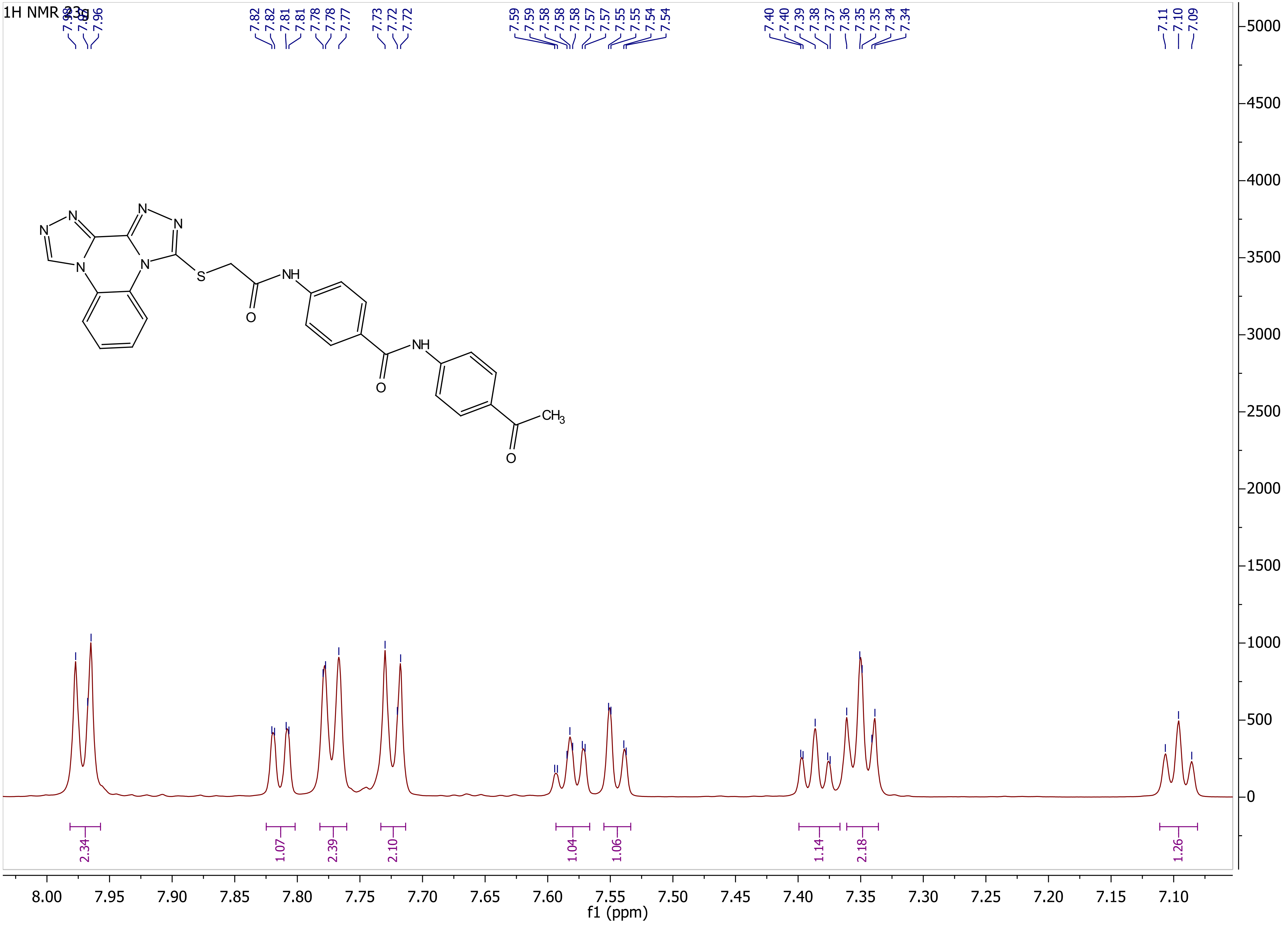
1H NMR 23g
7.96
7.82 7.82 7.81 7.81 7.78 7.78 7.77
7.73 7.72 7.72
7.59 7.59 7.58 7.58 7.58 7.57 7.57 7.55 7.55 7.54 7.54
7.40 7.40 7.39 7.38 7.37 7.36 7.35 7.35 7.34 7.34
7.11 7.10 7.09
N N N N N N N S NH O NH O CH3 O
2.34 1.07 2.39 2.10 1.04 1.06 1.14 2.18 1.26
8.00 7.95 7.90 7.85 7.80 7.75 7.70 7.65 7.60 7.55 7.50 7.45 7.40 7.35 7.30 7.25 7.20 7.15 7.10
f1 (ppm)
5000 4500 4000 3500 3000 2500 2000 1500 1000 500 0

1H NMR 23g

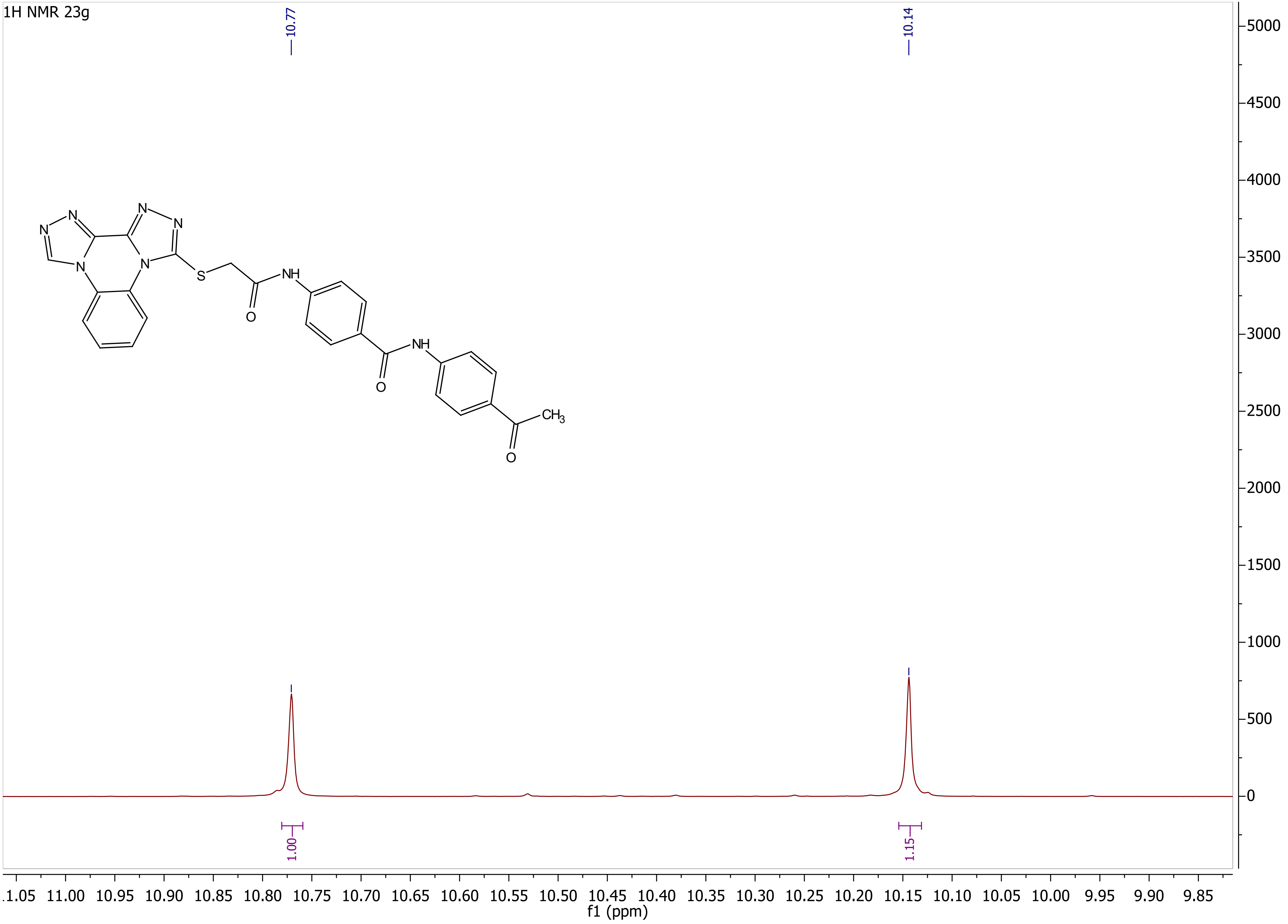

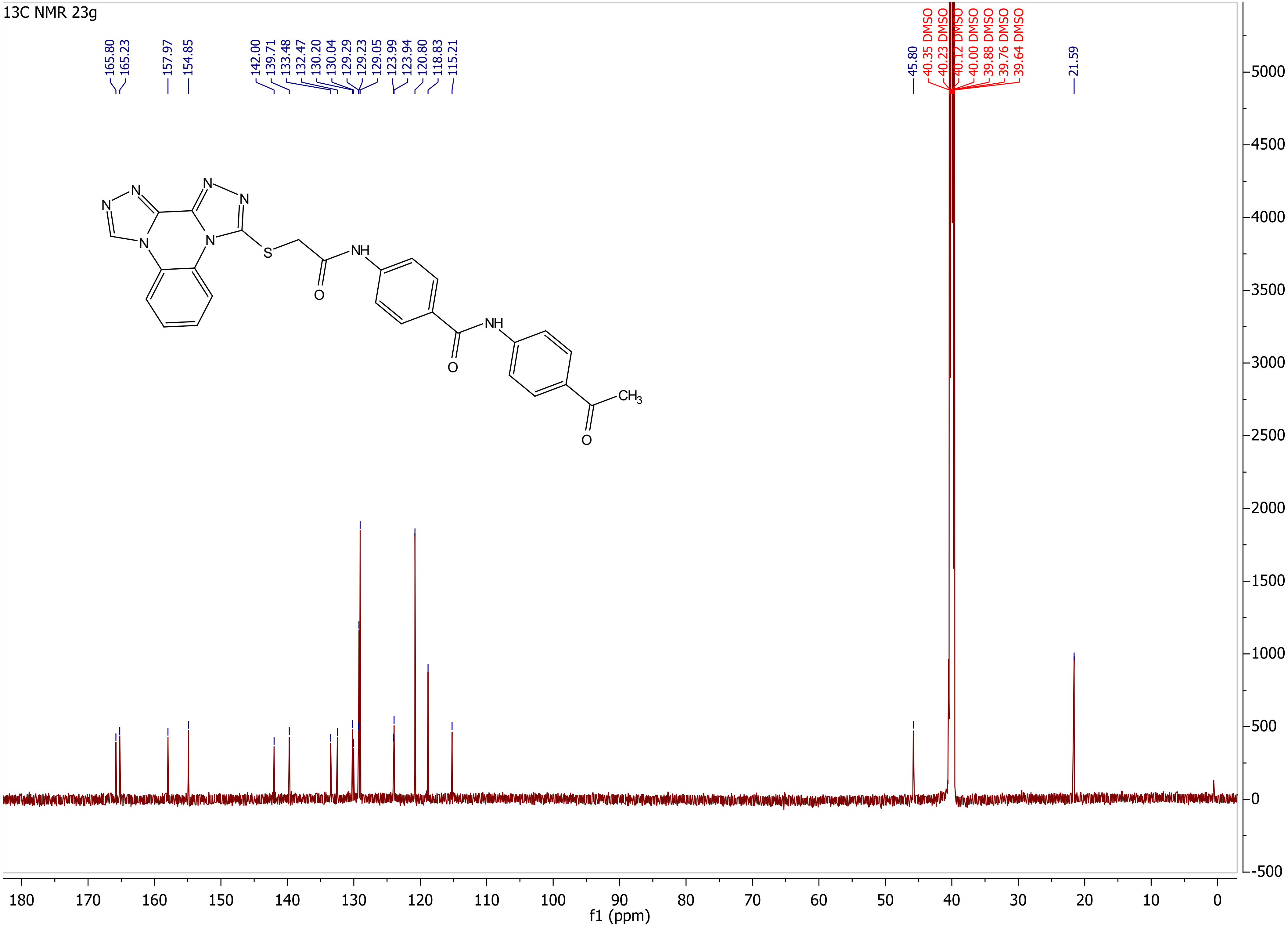
13C NMR 23g
165.80
165.23
157.97
154.85
142.00
139.71
133.48
132.47
130.20
130.04
129.29
129.23
129.05
123.99
123.94
120.80
118.83
115.21
45.80
40.35 DMSO
40.23 DMSO
40.12 DMSO
40.00 DMSO
39.88 DMSO
39.76 DMSO
39.64 DMSO
21.59
N
N
N
N
N
N
S
NH
O
NH
O
CH3
O
f1 (ppm)

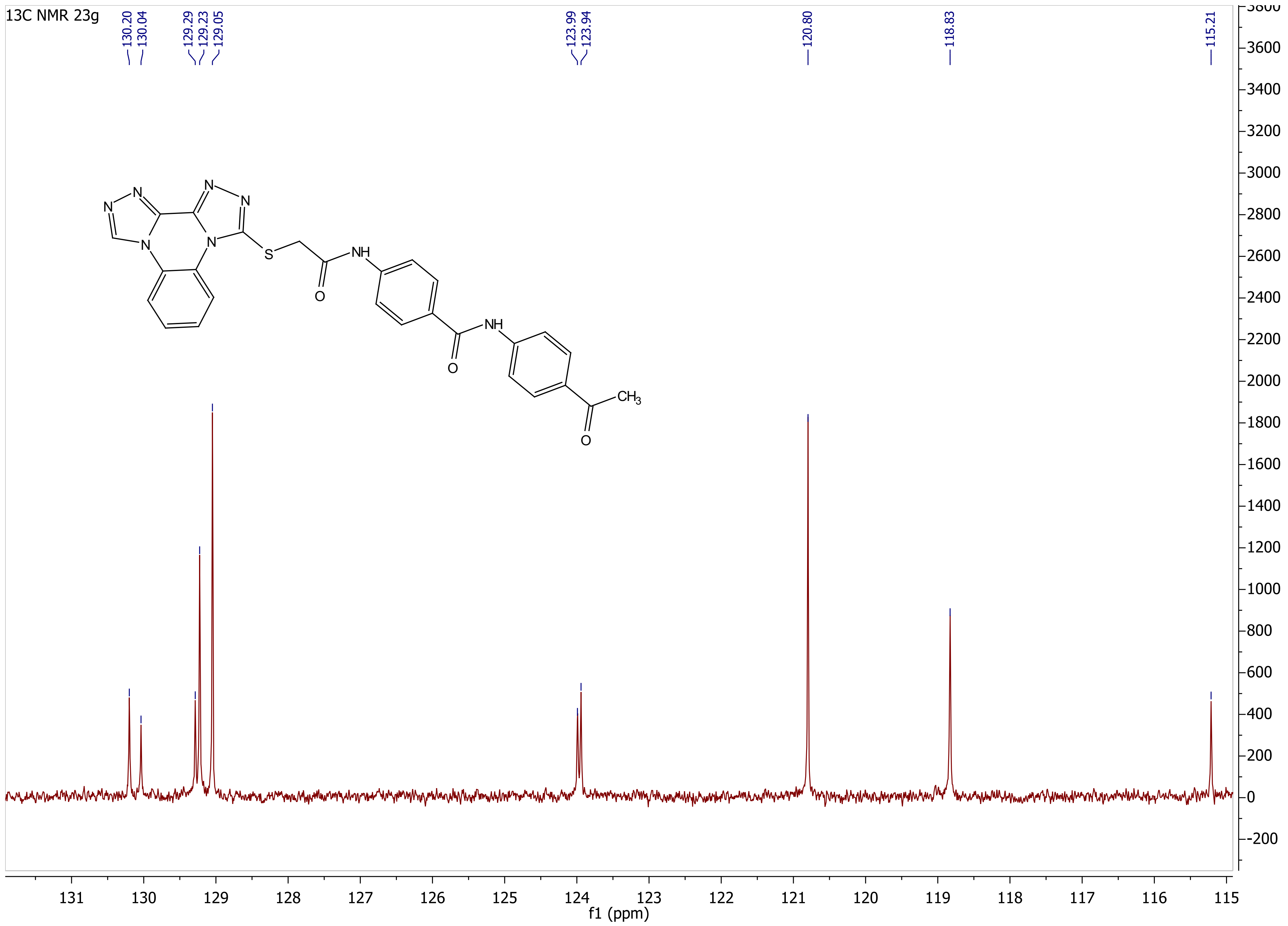
13C NMR 23g
130.20
130.04
129.29
129.23
129.05
123.99
123.94
120.80
118.83
115.21
N
N
N
N
N
N
S
NH
O
NH
O
CH3
O
f1 (ppm)

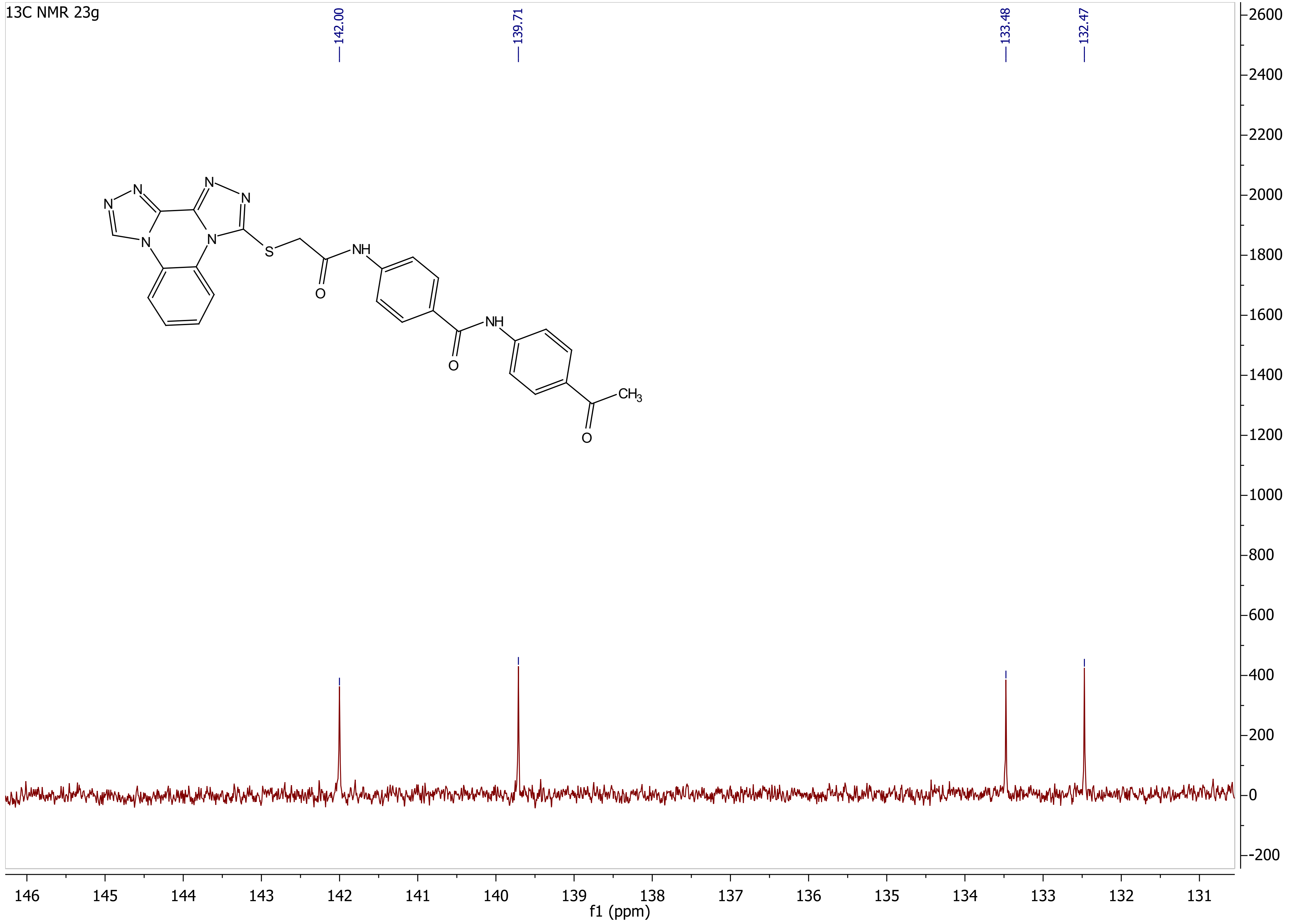

13C NMR 23g
142.00
139.71
133.48
132.47
N
N
N
N
N
N
S
NH
O
NH
O
CH3
O
f1 (ppm)

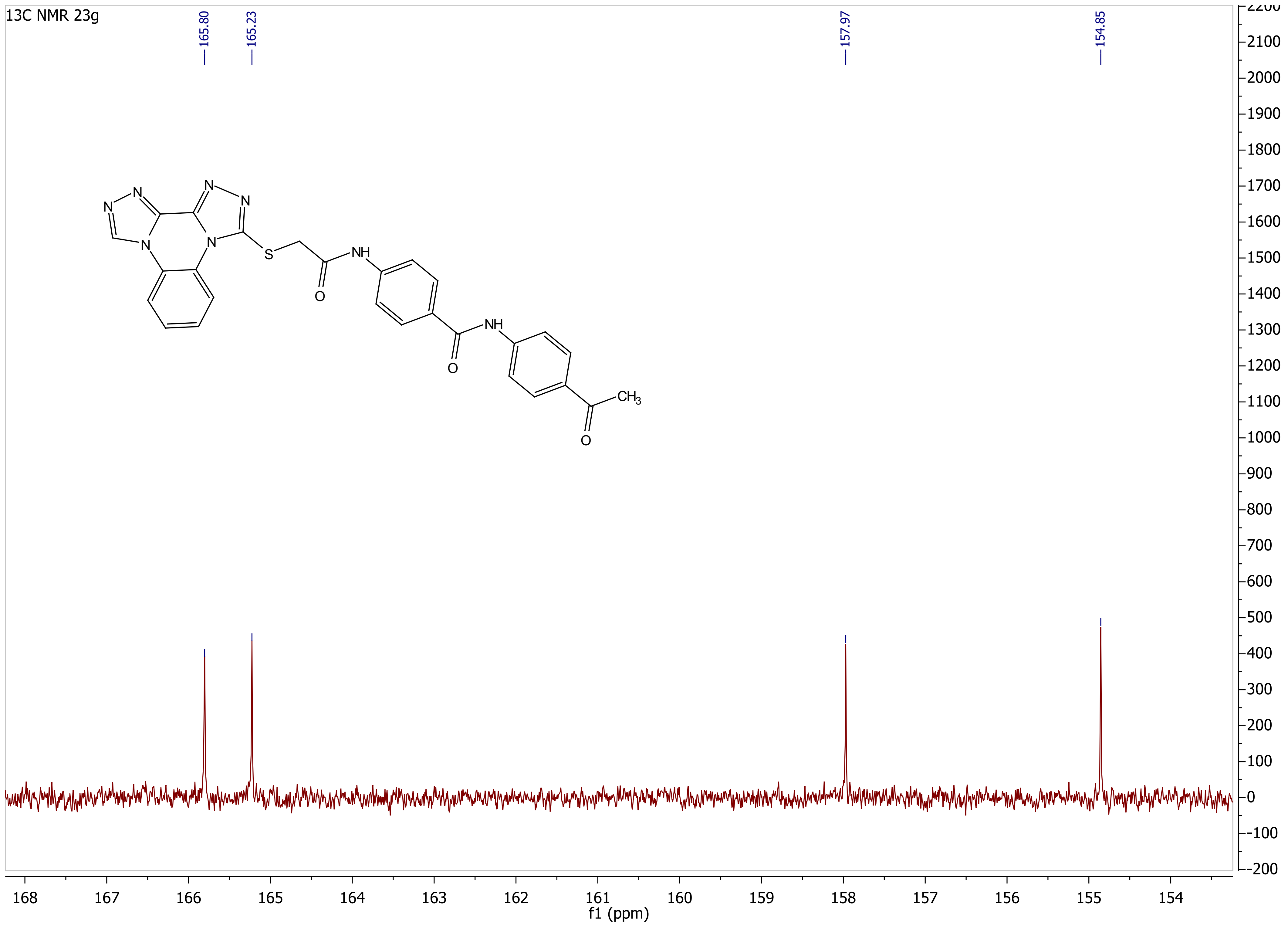

13C NMR 23g
165.80
165.23
157.97
154.85
N
N
N
N
N
N
S
NH
O
NH
O
CH3
O
2200
2100
2000
1900
1800
1700
1600
1500
1400
1300
1200
1100
1000
900
800
700
600
500
400
300
200
100
0
-100
-200
168
167
166
165
164
163
162
161
160
159
158
157
156
155
154
f1 (ppm)

Mass spec. of 23g

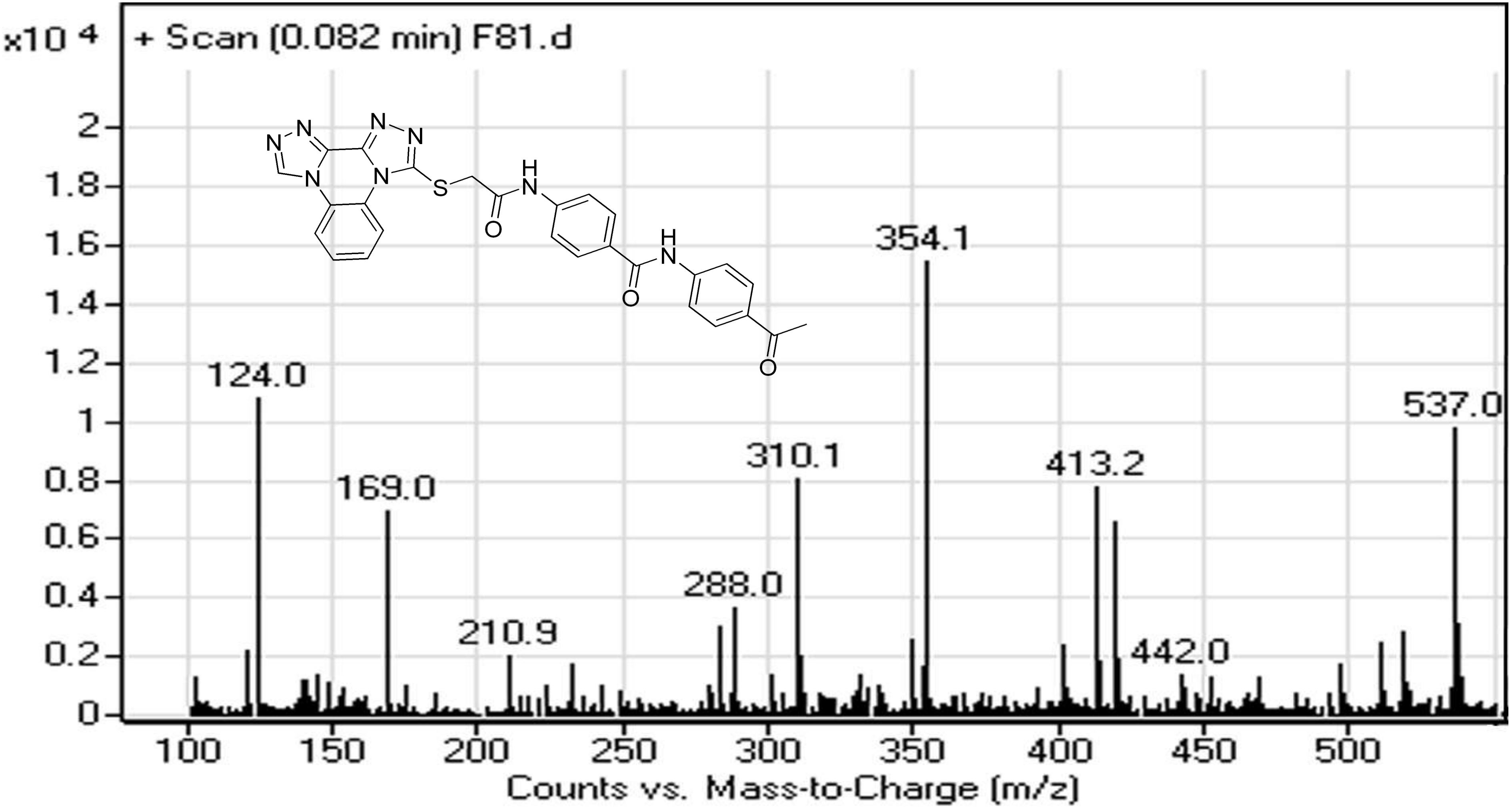

IR of compound 23h

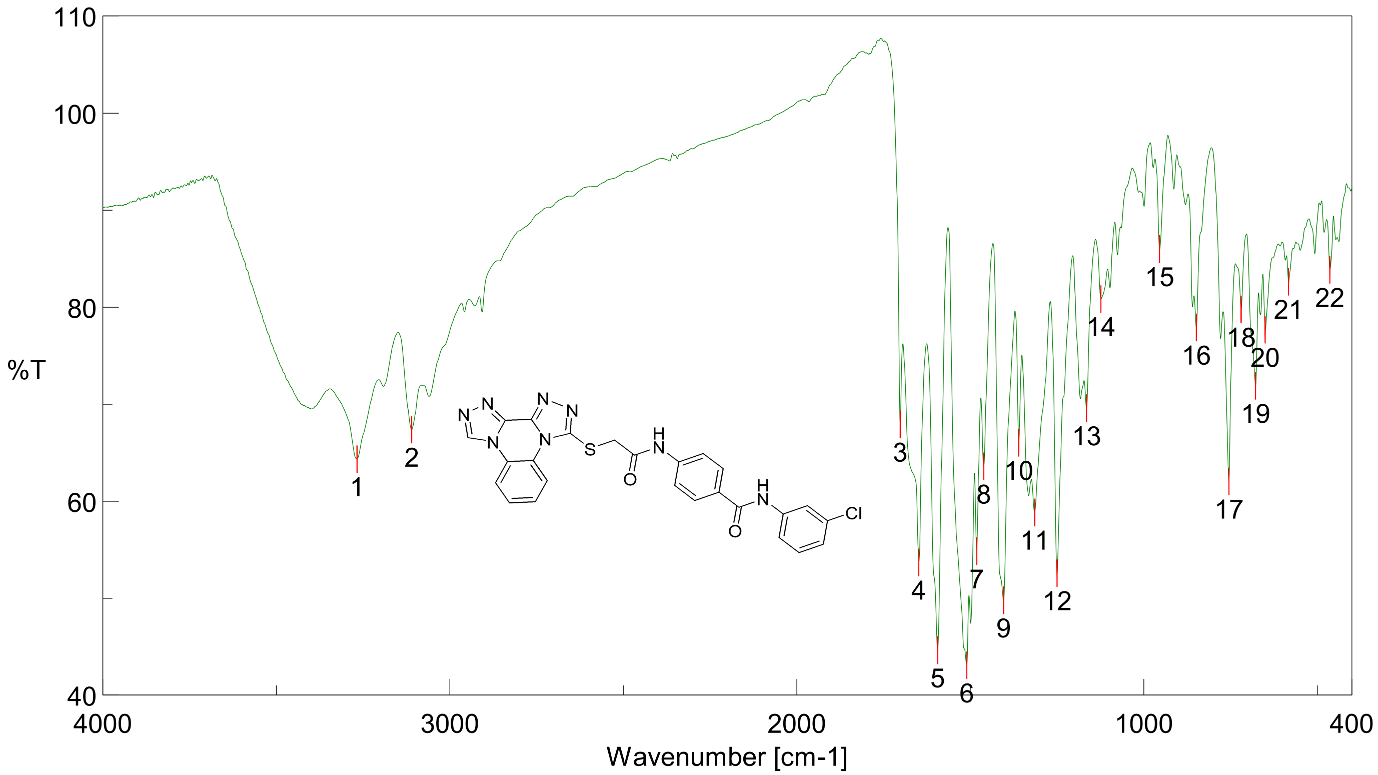

[Comments]

Sample name	F80
Comment	
User	
Division	
Company	KSU

[Detailed Information]

Creation date	10/29/2020 5:07 AM
Data array type	Linear data array
Horizontal axis	Wavenumber [cm-1]
Vertical axis	%T
Start	399.193 cm-1
End	4000.6 cm-1
Data interval	0.964233 cm-1
Data points	3736

[Measurement Information]

Model Name	FT/IR-6600typeA
Serial Number	A014661790
Measurement Date	10/28/2020 3:00 AM
Light Source	Standard
Detector	TGS
Accumulation	Auto (19)
Resolution	4 cm-1
Zero Filling	On
Apodization	Cosine
Gain	Auto (2)
Aperture	Auto (7.1 mm)
Scanning Speed	Auto (2 mm/sec)
Filter	Auto (10000 Hz)

[Result of Peak Picking]

No.	Position	Intensity	No.	Position	Intensity	No.	Position	Intensity
1	3267.79	64.3225	2	3109.65	67.349	3	1701.87	67.895

[Result of Peak Picking]

No.	Position	Intensity	No.	Position	Intensity	No.	Position	Intensity
4	1647.88	53.6526	5	1593.88	44.6097	6	1509.99	43.0796
7	1481.06	54.8225	8	1460.81	63.5677	9	1403.92	49.7525
10	1360.53	65.9995	11	1314.25	58.8253	12	1249.65	52.5638
13	1164.79	69.5728	14	1123.33	80.8099	15	954.591	85.9848
16	848.525	77.8986	17	754.031	62.0098	18	719.318	79.7438
19	677.856	71.8732	20	649.893	77.6448	21	582.397	82.6077
22	463.796	83.827						

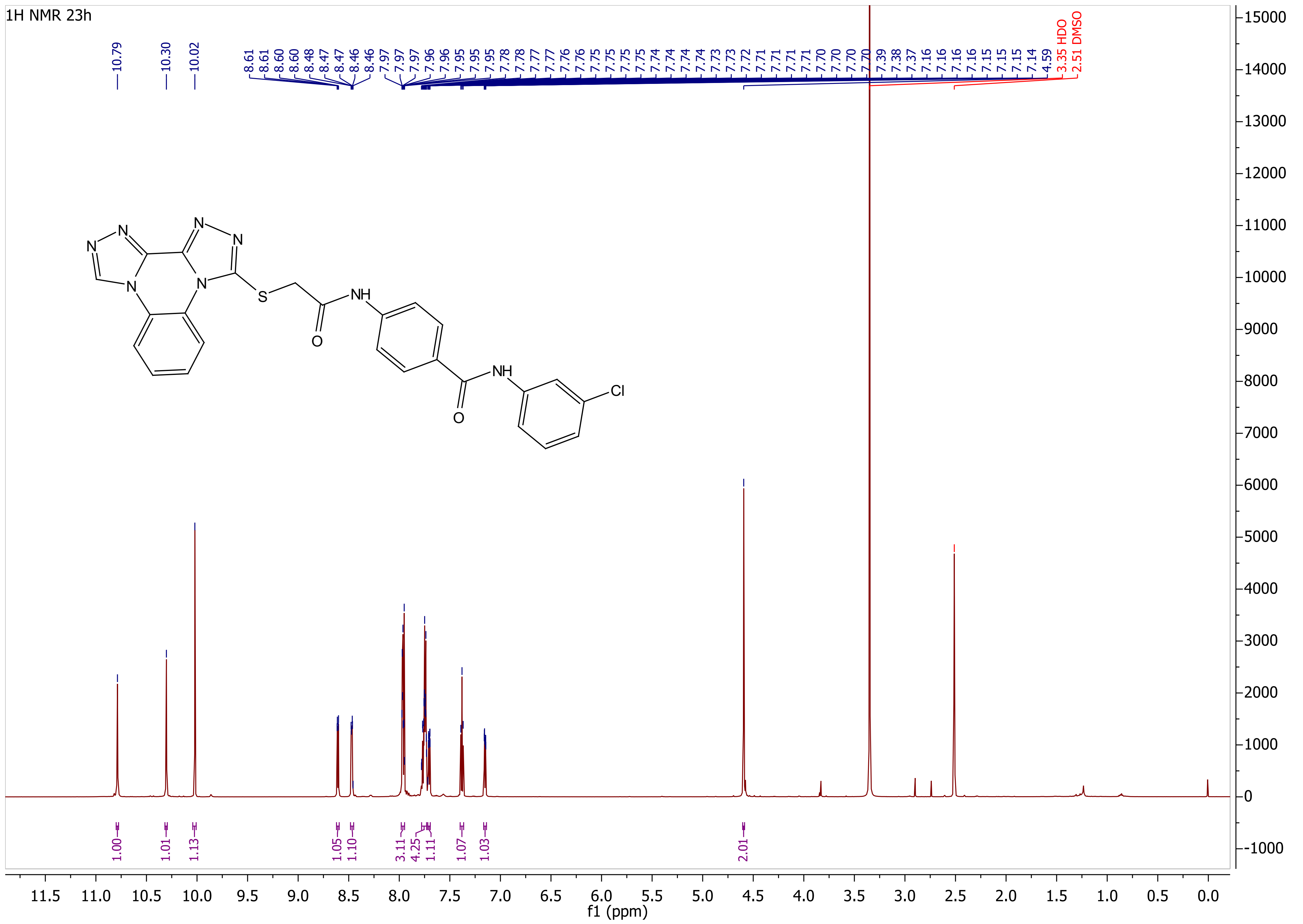

1H NMR 23h
10.79
10.30
10.02
8.61
8.61
8.60
8.60
8.48
8.47
8.47
8.46
8.46
7.97
7.97
7.97
7.96
7.96
7.95
7.95
7.95
7.78
7.78
7.77
7.77
7.76
7.76
7.75
7.75
7.75
7.75
7.74
7.74
7.74
7.74
7.73
7.73
7.72
7.71
7.71
7.71
7.71
7.70
7.70
7.70
7.70
7.39
7.38
7.37
7.16
7.16
7.16
7.15
7.15
7.15
7.14
4.59
3.35 HDO
2.51 DMSO
N
N
N
N
N
N
S
NH
O
NH
O
Cl
1.00
1.01
1.13
1.05
1.10
3.11
4.25
1.11
1.07
1.03
2.01
f1 (ppm)

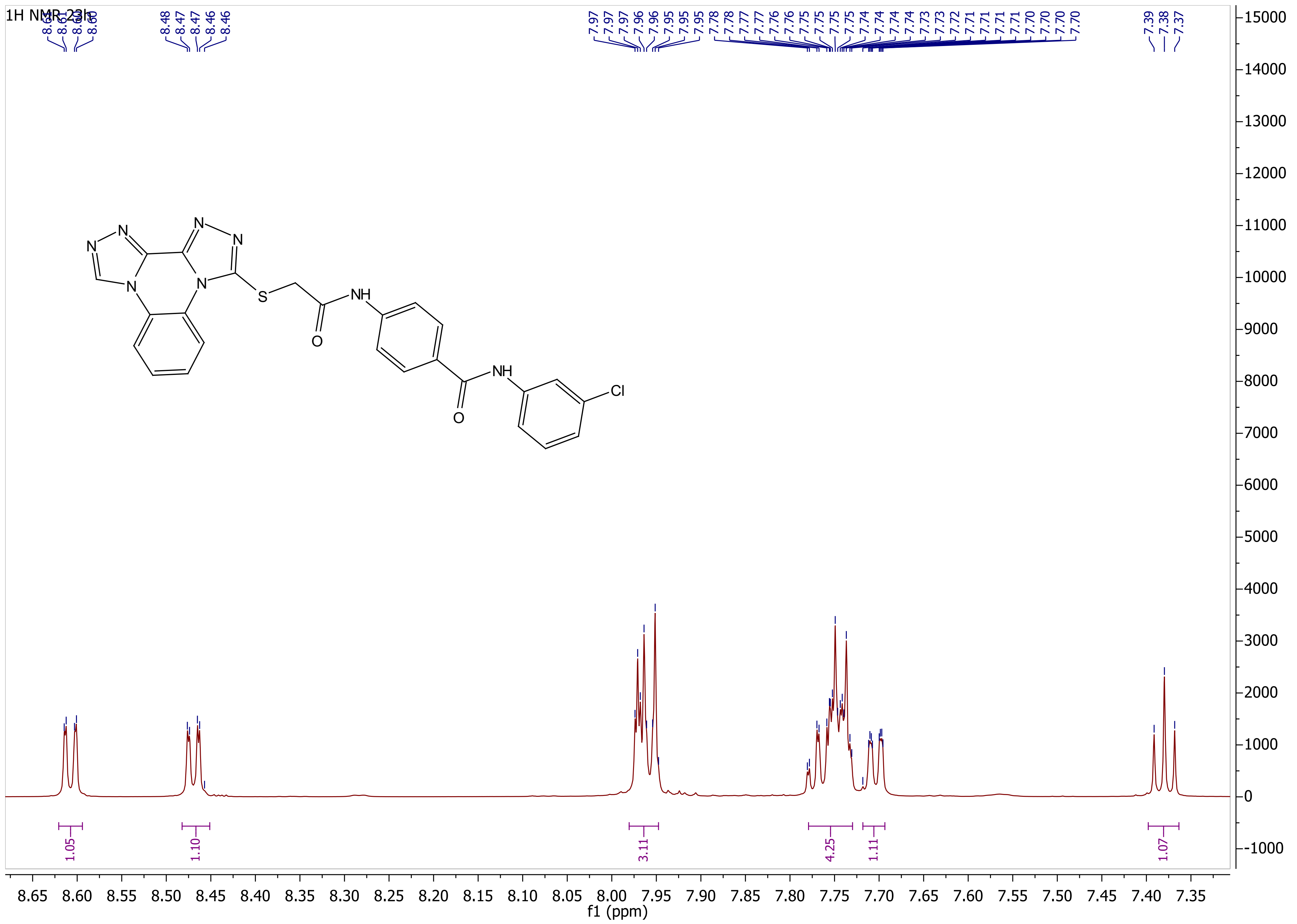

1H NMR 23h
8.48
8.47
8.47
8.46
8.46
7.97
7.97
7.97
7.96
7.96
7.95
7.95
7.95
7.78
7.78
7.77
7.77
7.76
7.76
7.75
7.75
7.75
7.75
7.74
7.74
7.74
7.74
7.73
7.73
7.72
7.71
7.71
7.71
7.71
7.70
7.70
7.70
7.70
7.39
7.38
7.37
N
N
N
N
N
N
S
NH
O
NH
O
Cl
1.05
1.10
3.11
4.25
1.11
1.07
f1 (ppm)

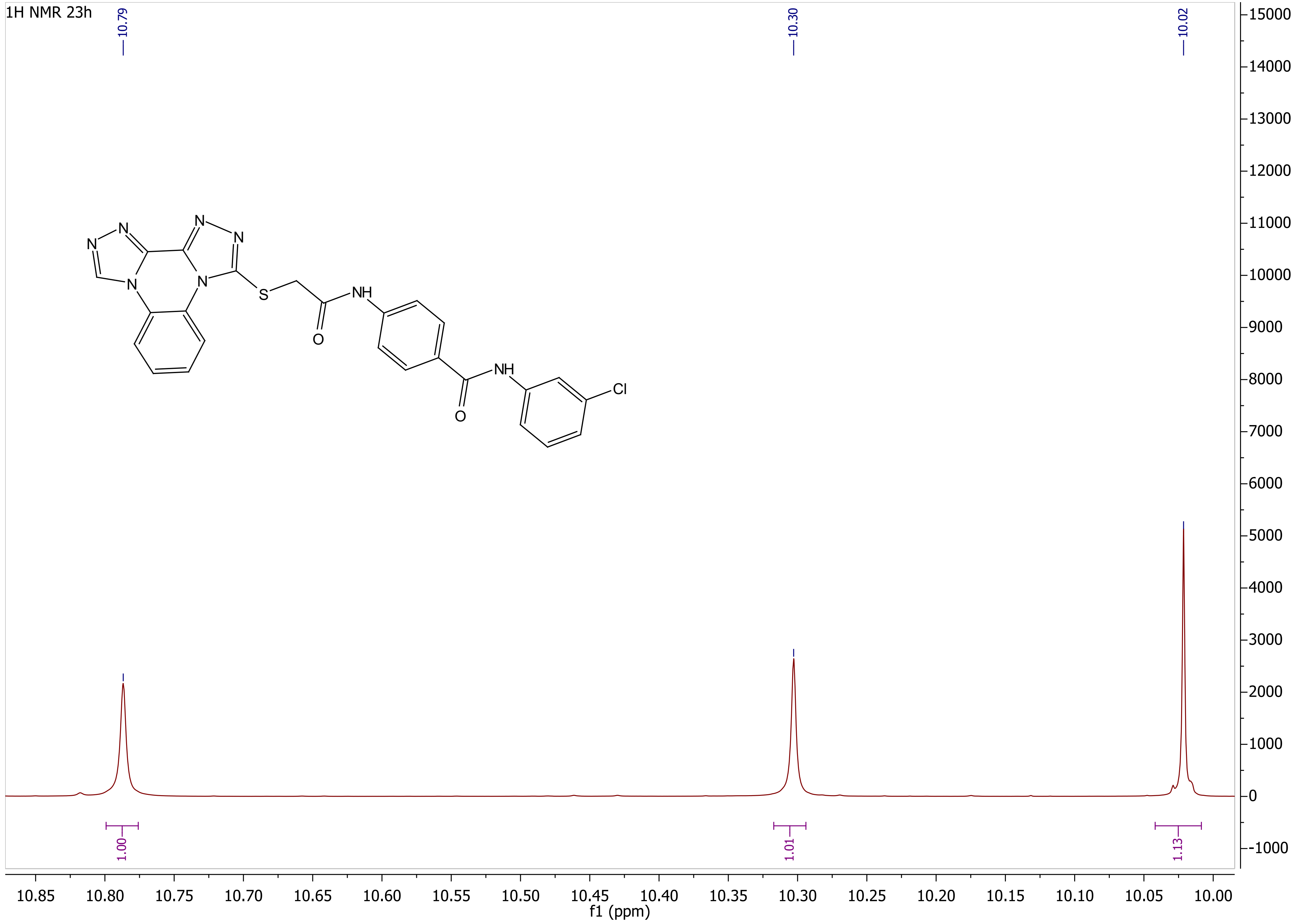

1H NMR 23h
10.79
10.30
10.02
N
N
N
N
N
N
S
NH
O
NH
O
Cl
1.00
1.01
1.13
f1 (ppm)

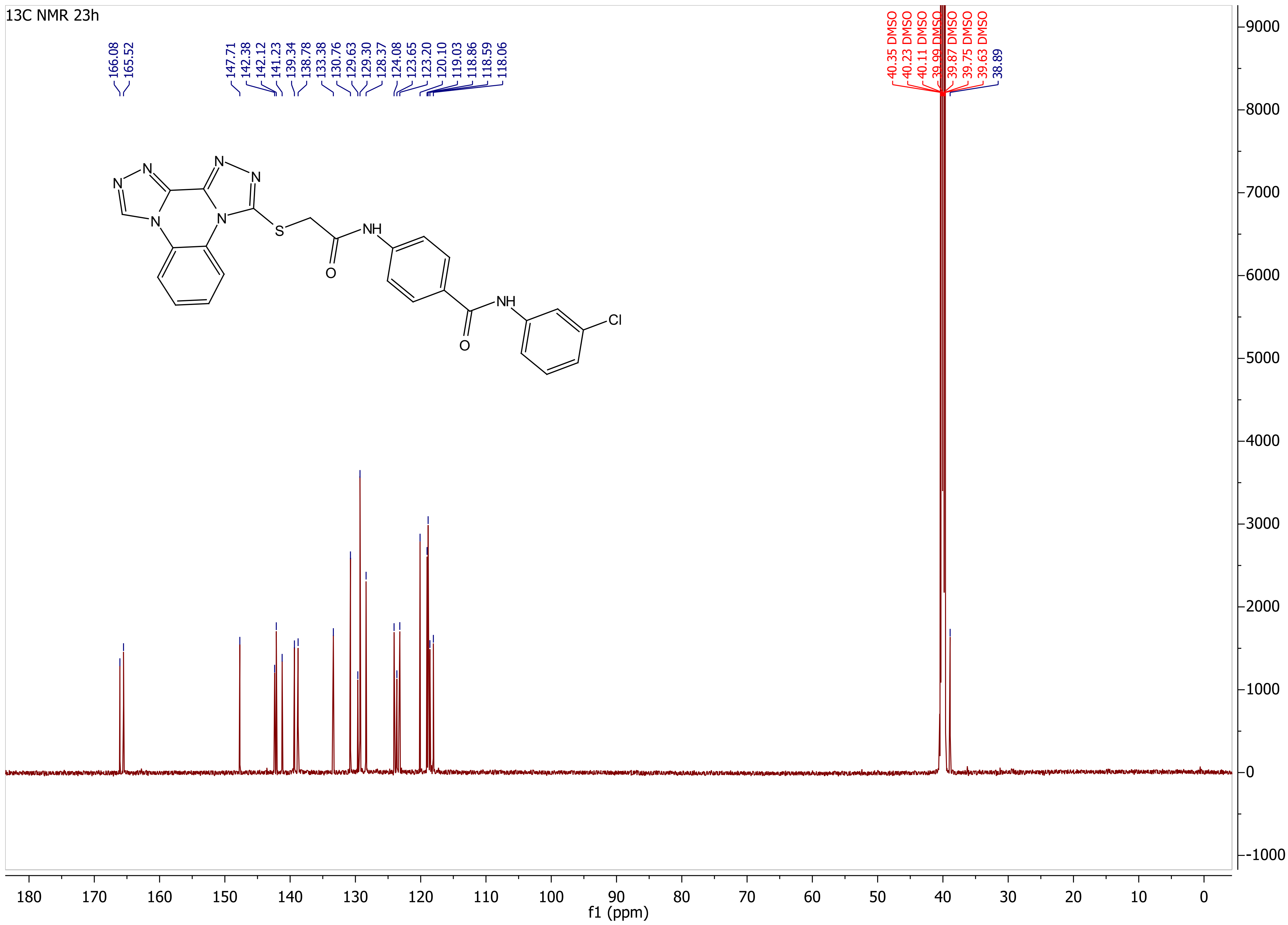

13C NMR 23h
166.08
165.52
147.71
142.38
142.12
141.23
139.34
138.78
133.38
130.76
129.63
129.30
128.37
124.08
123.65
123.20
120.10
119.03
118.86
118.59
118.06
40.35 DMSO
40.23 DMSO
40.11 DMSO
39.99 DMSO
39.87 DMSO
39.75 DMSO
39.63 DMSO
38.89
N
N
N
N
N
N
S
NH
O
NH
O
Cl
f1 (ppm)

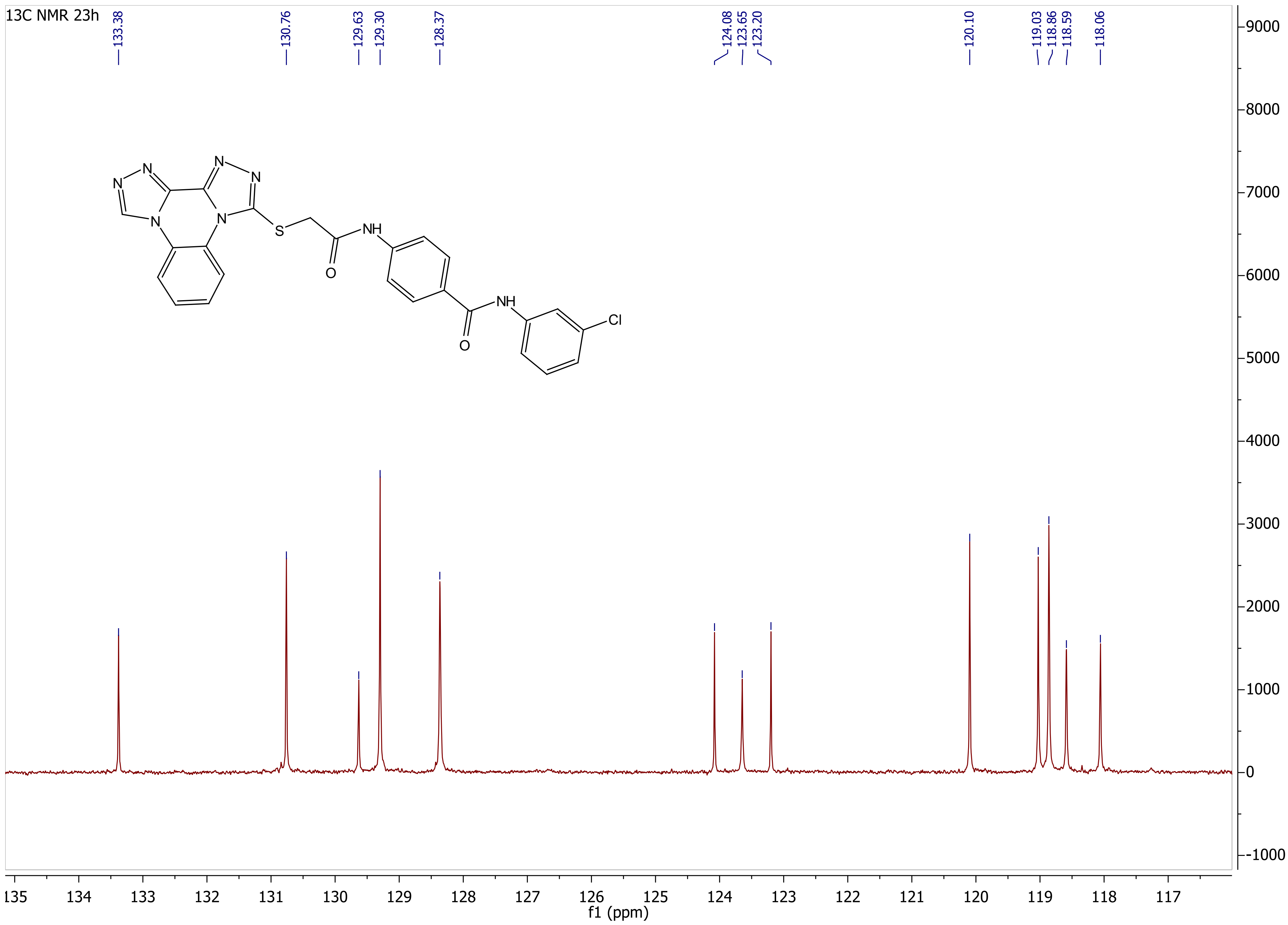
13C NMR 23h
133.38
130.76
129.63
129.30
128.37
124.08
123.65
123.20
120.10
119.03
118.86
118.59
118.06
N
N
N
N
N
N
S
O
NH
NH
O
Cl
9000
8000
7000
6000
5000
4000
3000
2000
1000
0
-1000
135
134
133
132
131
130
129
128
127
126
125
124
123
122
121
120
119
118
117
f1 (ppm)

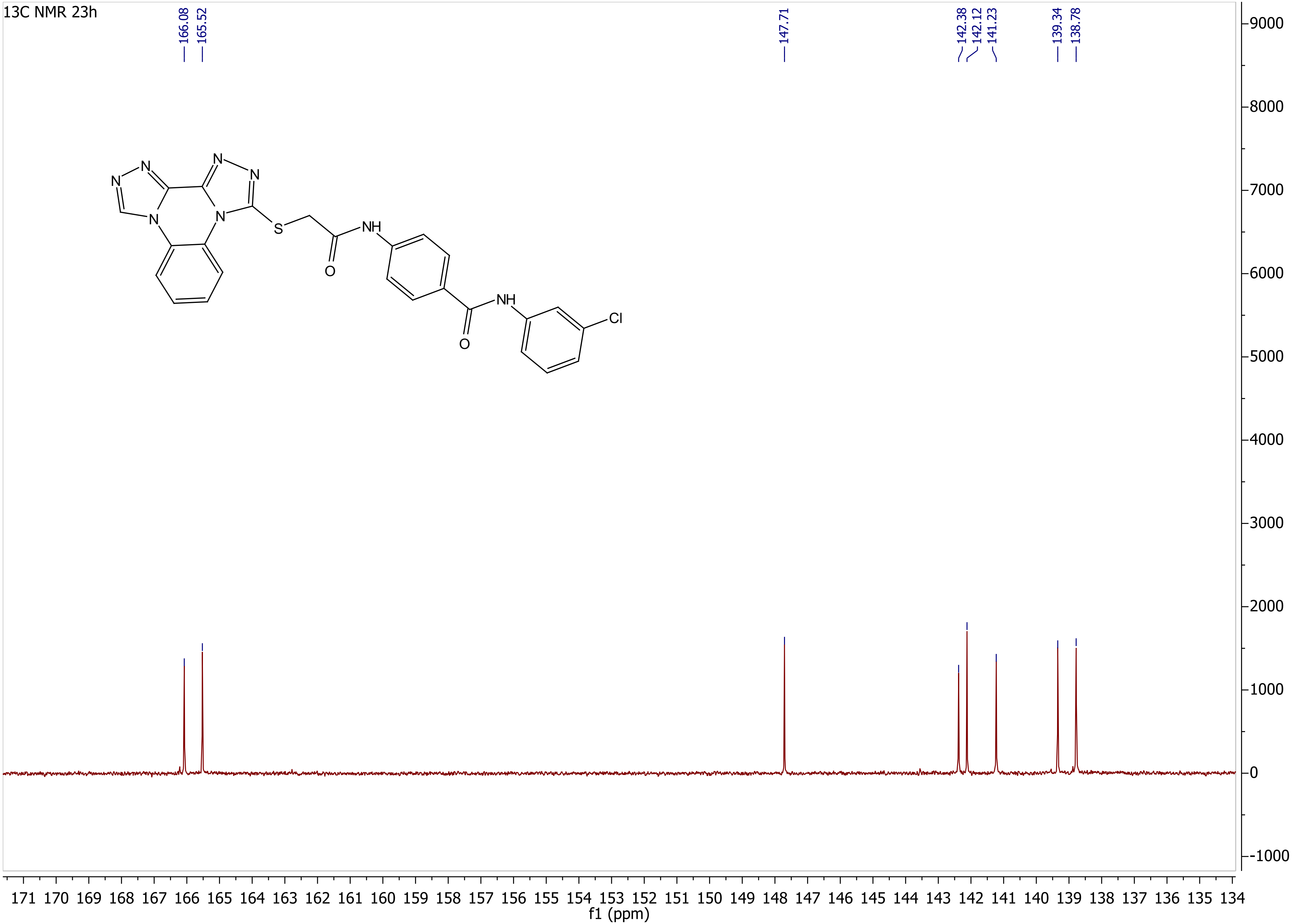
13C NMR 23h
166.08
165.52
147.71
142.38
142.12
141.23
139.34
138.78
N
N
N
N
N
N
S
NH
O
NH
O
Cl
9000
8000
7000
6000
5000
4000
3000
2000
1000
0
-1000
171 170 169 168 167 166 165 164 163 162 161 160 159 158 157 156 155 154 153 152 151 150 149 148 147 146 145 144 143 142 141 140 139 138 137 136 135 134
f1 (ppm)

IR for compound 23i

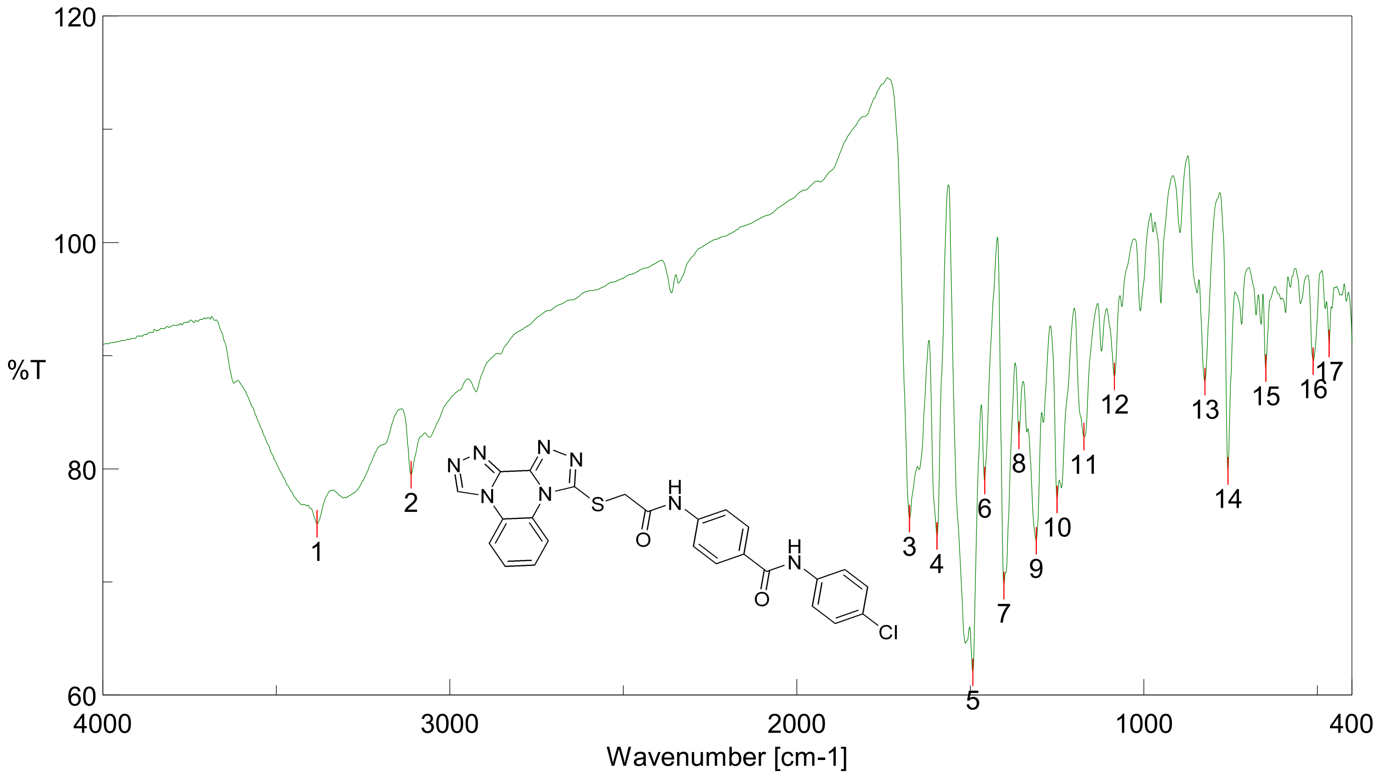

[Comments]

Sample name	F77
Comment	
User	
Division	
Company	KSU

[Detailed Information]

Creation date	10/29/2020 5:05 AM
Data array type	Linear data array
Horizontal axis	Wavenumber [cm-1]
Vertical axis	%T
Start	399.193 cm-1
End	4000.6 cm-1
Data interval	0.964233 cm-1
Data points	3736

[Measurement Information]

Model Name	FT/IR-6600typeA
Serial Number	A014661790
Measurement Date	10/28/2020 2:51 AM
Light Source	Standard
Detector	TGS
Accumulation	Auto (18)
Resolution	4 cm-1
Zero Filling	On
Apodization	Cosine
Gain	Auto (2)
Aperture	Auto (7.1 mm)
Scanning Speed	Auto (2 mm/sec)
Filter	Auto (10000 Hz)

[Result of Peak Picking]

No.	Position	Intensity	No.	Position	Intensity	No.	Position	Intensity
1	3382.53	75.1353	2	3111.58	79.4712	3	1674.87	75.6006

[Result of Peak Picking]

No.	Position	Intensity	No.	Position	Intensity	No.	Position	Intensity
4	1595.81	74.0606	5	1492.63	62.011	6	1457.92	78.9496
7	1402.96	69.6819	8	1359.57	82.9433	9	1309.43	73.649
10	1249.65	77.2888	11	1172.51	82.8252	12	1084.76	88.1455
13	823.455	87.7062	14	756.923	79.8058	15	647.965	88.8807
16	512.008	89.4952	17	465.725	91.0736			

1H NMR 23i

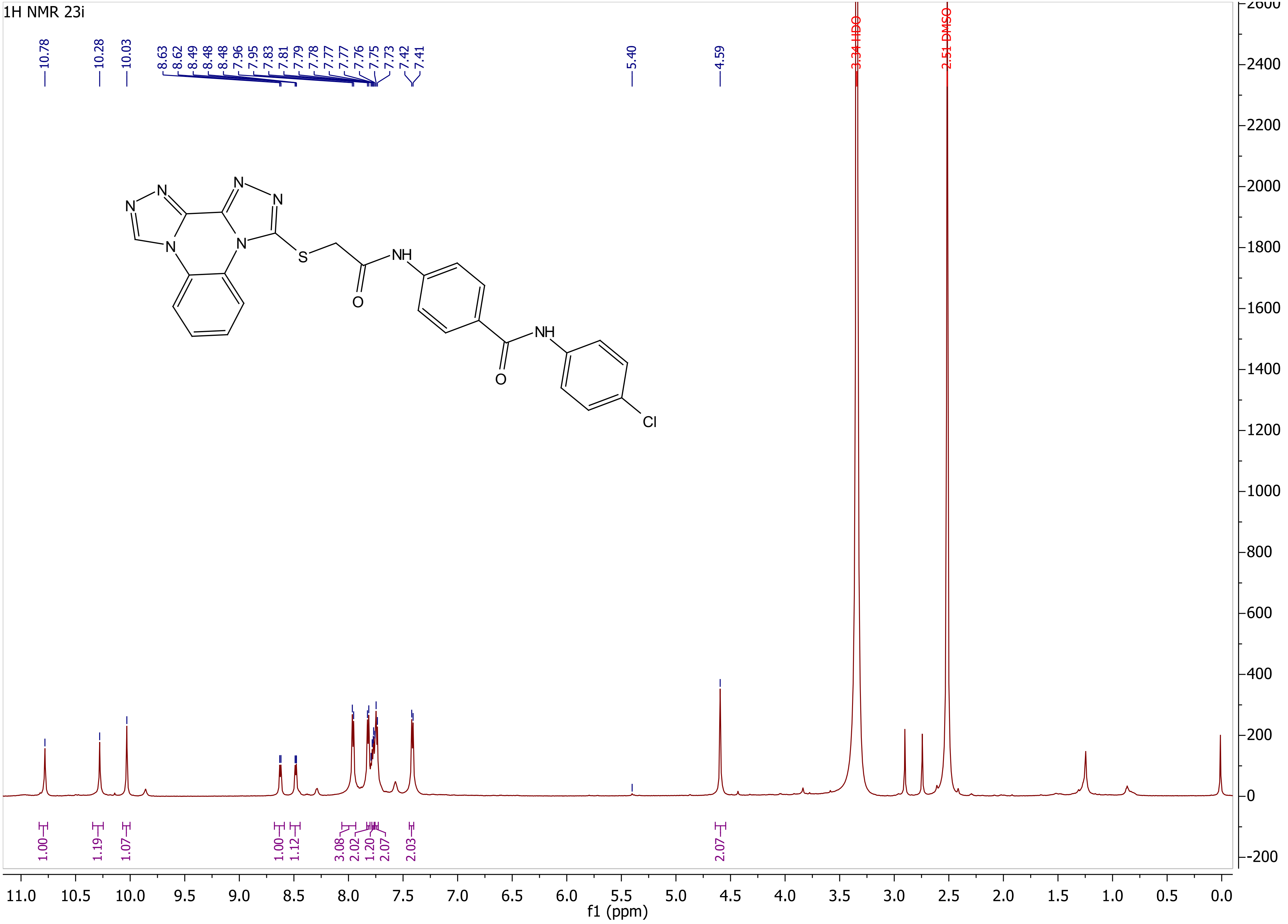

10.78
10.28
10.03
8.63
8.62
8.49
8.48
8.48
7.96
7.95
7.83
7.81
7.79
7.78
7.77
7.77
7.76
7.75
7.73
7.42
7.41
5.40
4.59
3.34 HDO
2.51 DMSO
N
N
N
N
N
N
S
NH
O
NH
O
Cl
1.00
1.19
1.07
1.00
1.12
3.08
2.02
1.20
2.07
2.03
2.07
f1 (ppm)

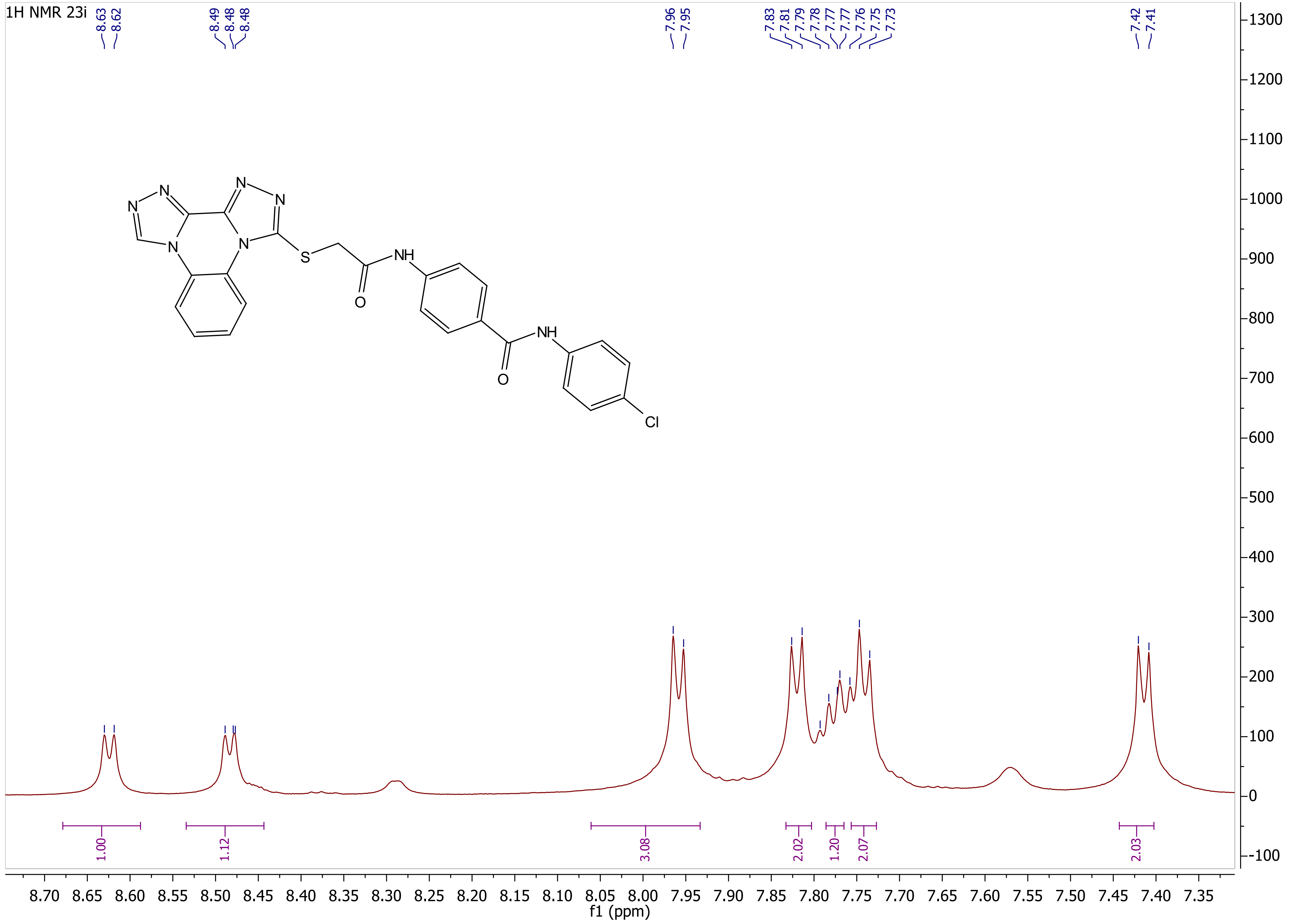
1H NMR 23i
8.63
8.62
8.49
8.48
8.48
7.96
7.95
7.83
7.81
7.79
7.78
7.77
7.77
7.76
7.75
7.73
7.42
7.41
1.00
1.12
3.08
2.02
1.20
2.07
2.03
f1 (ppm)
N
N
N
N
N
N
S
NH
O
NH
O
Cl

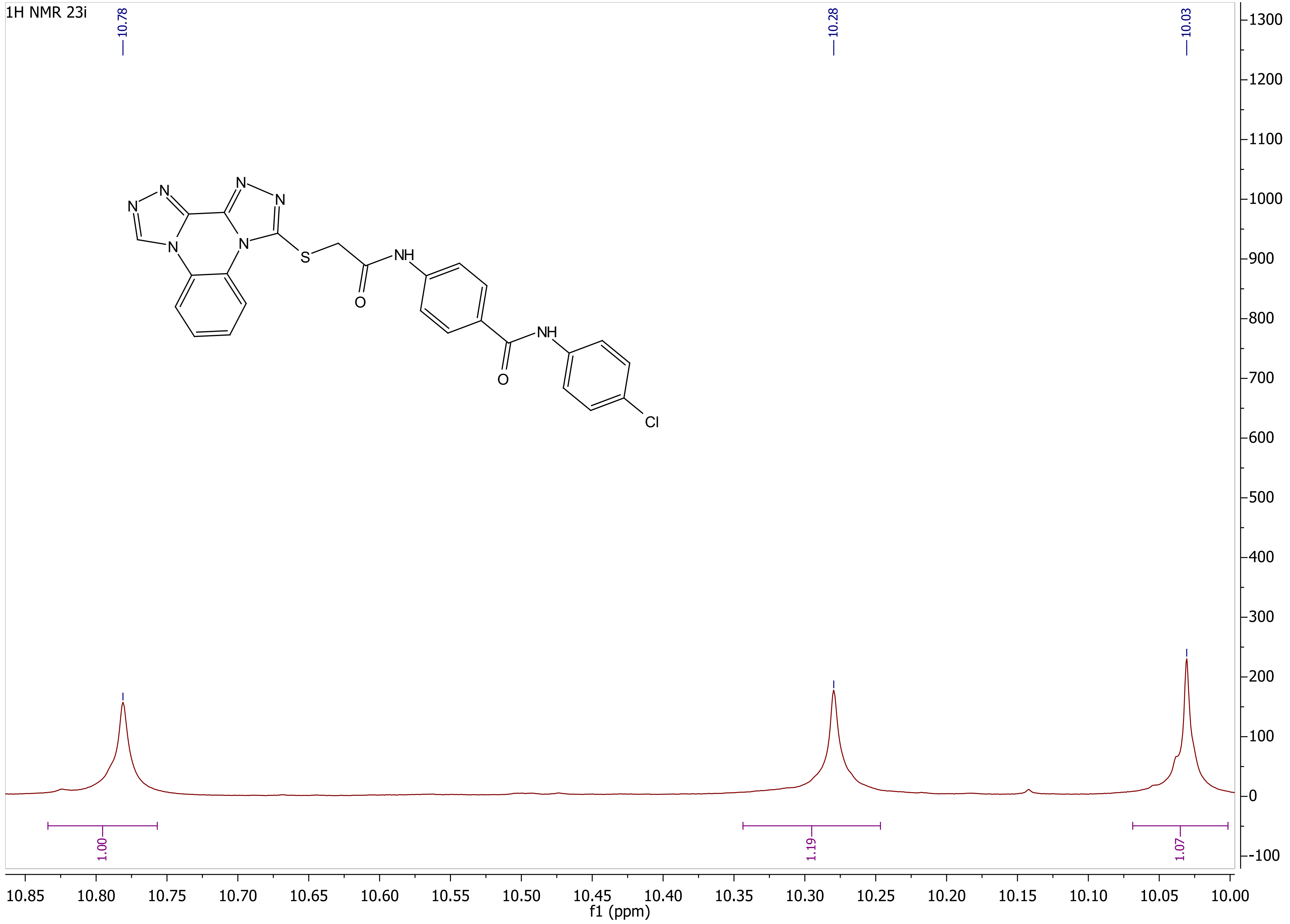
1H NMR 23i
10.78
10.28
10.03
1.00
1.19
1.07
N
N
N
N
N
N
S
O
NH
NH
O
Cl
f1 (ppm)
10.85
10.80
10.75
10.70
10.65
10.60
10.55
10.50
10.45
10.40
10.35
10.30
10.25
10.20
10.15
10.10
10.05
10.00
1300
1200
1100
1000
900
800
700
600
500
400
300
200
100
0
-100

13C NMR 23i

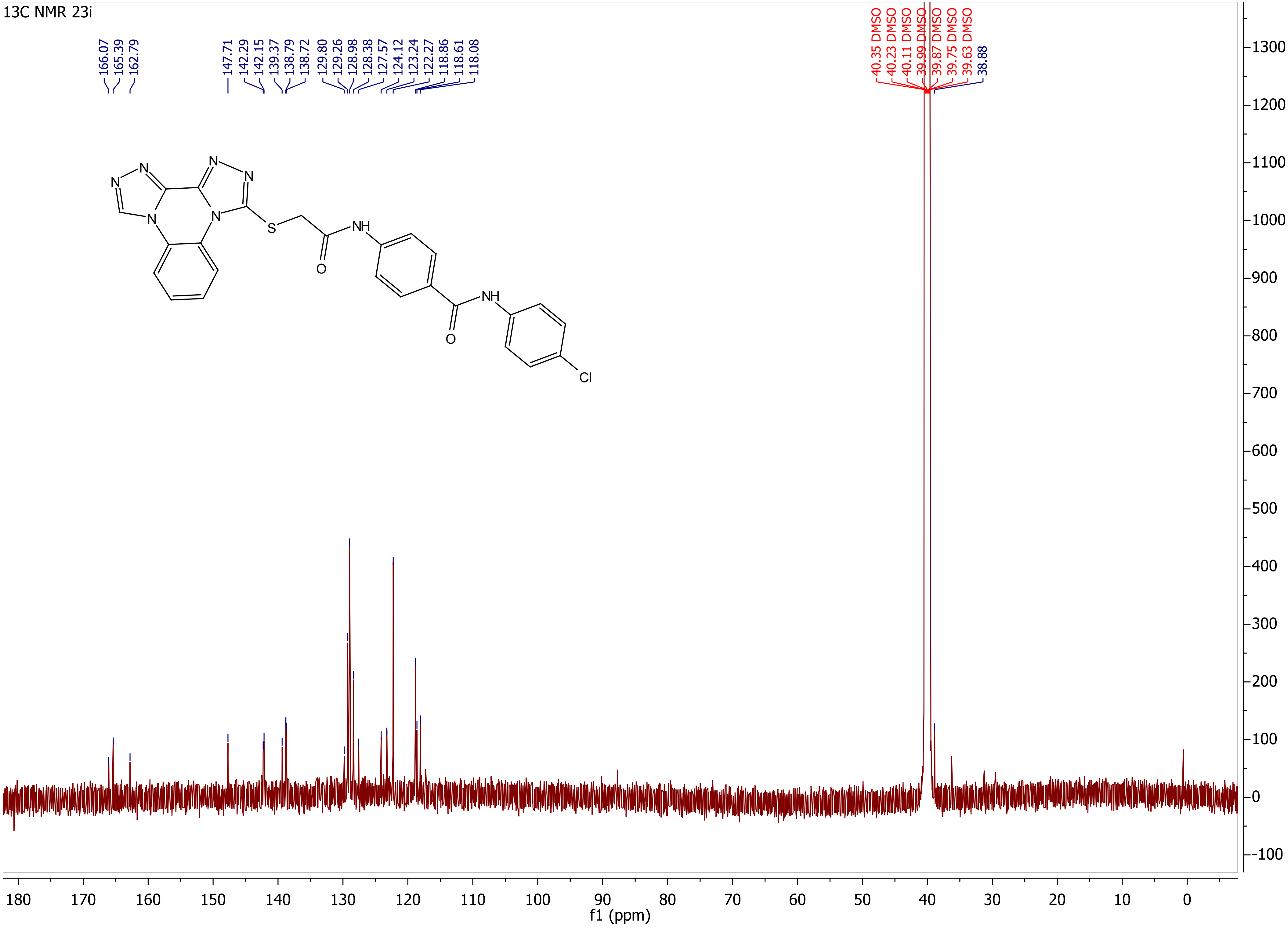
166.07
165.39
162.79
147.71
142.29
142.15
139.37
138.79
138.72
129.80
129.26
128.98
128.38
127.57
124.12
123.24
122.27
118.86
118.61
118.08
40.35 DMSO
40.23 DMSO
40.11 DMSO
39.99 DMSO
39.87 DMSO
39.75 DMSO
39.63 DMSO
38.88
N
N
N
N
N
N
S
NH
O
NH
O
Cl
f1 (ppm)

13C NMR 23i

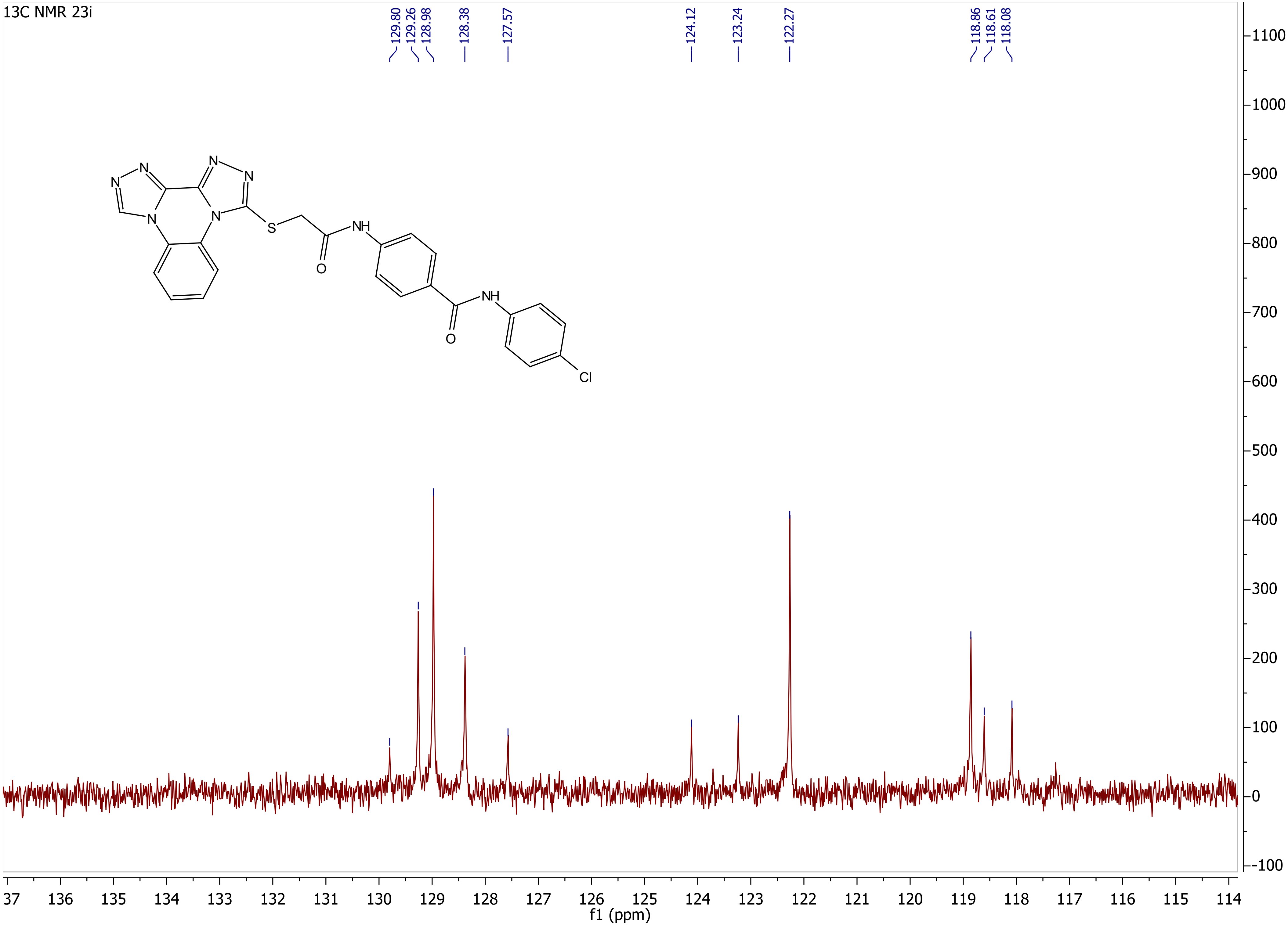

129.80
129.26
128.98
128.38
127.57
124.12
123.24
122.27
118.86
118.61
118.08
f1 (ppm)
NH
NH
O
O
S
N
Cl

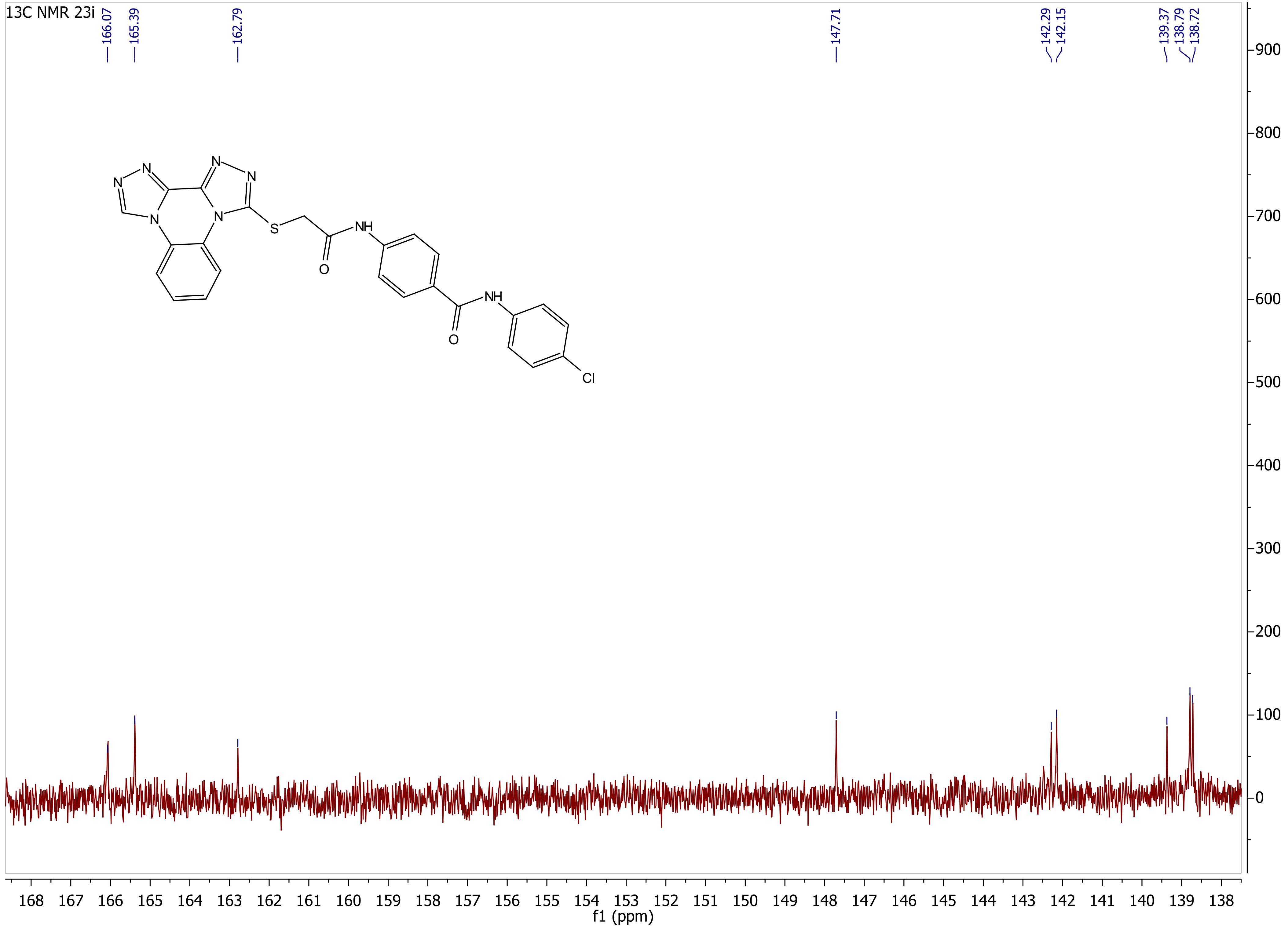
13C NMR 23i
166.07
165.39
162.79
147.71
142.29
142.15
139.37
138.79
138.72
f1 (ppm)
N
N
N
N
N
N
S
NH
O
NH
O
Cl

IR of compound 23j

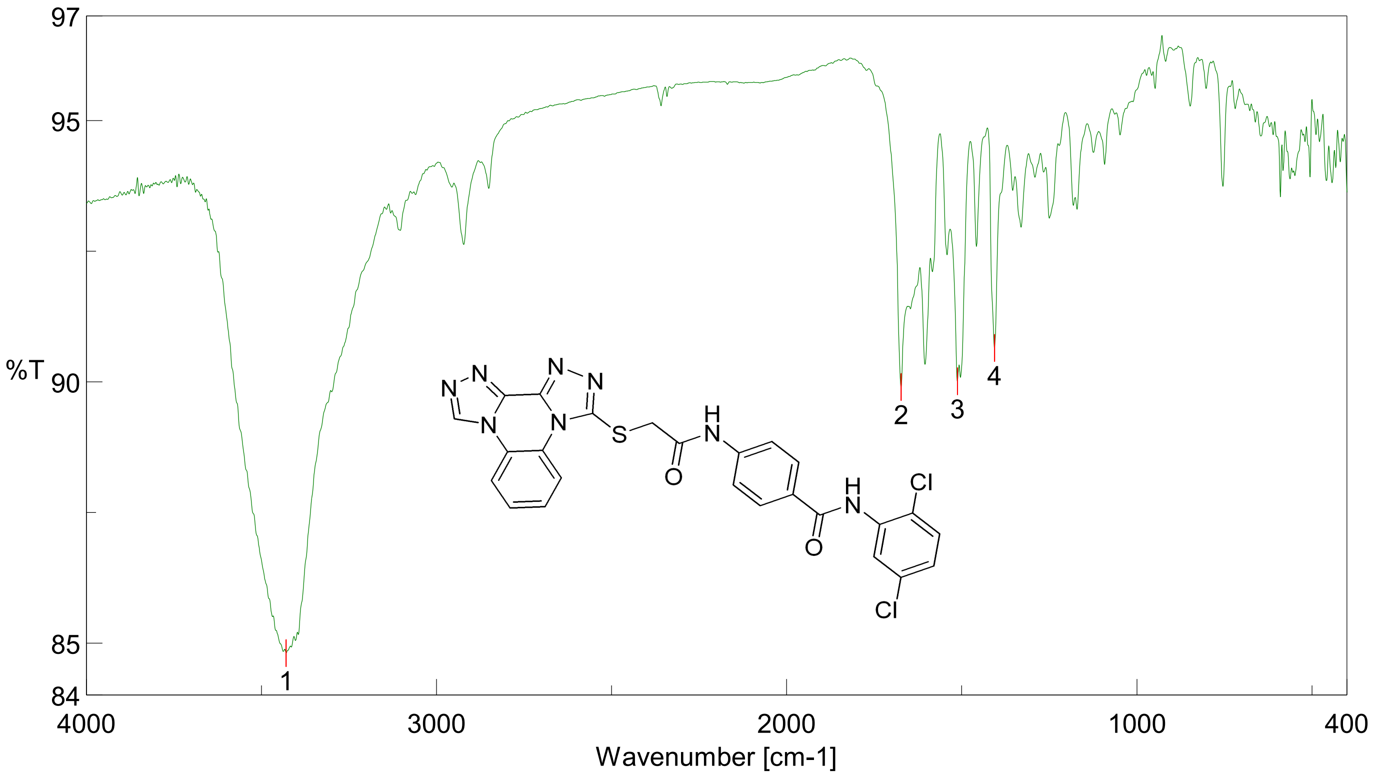

[Comments]

Sample name	F88
Comment	
User	
Division	
Company	KSU

[Detailed Information]		[Measurement Information]	
Creation date	10/29/2020 5:10 AM	Model Name	FT/IR-6600typeA
		Serial Number	A014661790
Data array type	Linear data array		
Horizontal axis	Wavenumber [cm-1]	Measurement Date	10/28/2020 4:16 AM
Vertical axis	%T		
Start	399.193 cm-1	Light Source	Standard
End	4000.6 cm-1	Detector	TGS
Data interval	0.964233 cm-1	Accumulation	Auto (13)
Data points	3736	Resolution	4 cm-1
		Zero Filling	On
		Apodization	Cosine
		Gain	Auto (1)
		Aperture	Auto (7.1 mm)
		Scanning Speed	Auto (2 mm/sec)
		Filter	Auto (10000 Hz)

[Result of Peak Picking]

No.	Position	Intensity	No.	Position	Intensity	No.	Position	Intensity
1	3429.78	84.8057	2	1672.95	89.8989	3	1511.92	90.009

[Result of Peak Picking]

No.	Position	Intensity	No.	Position	Intensity	No.	Position	Intensity
4	1405.85	90.6462						

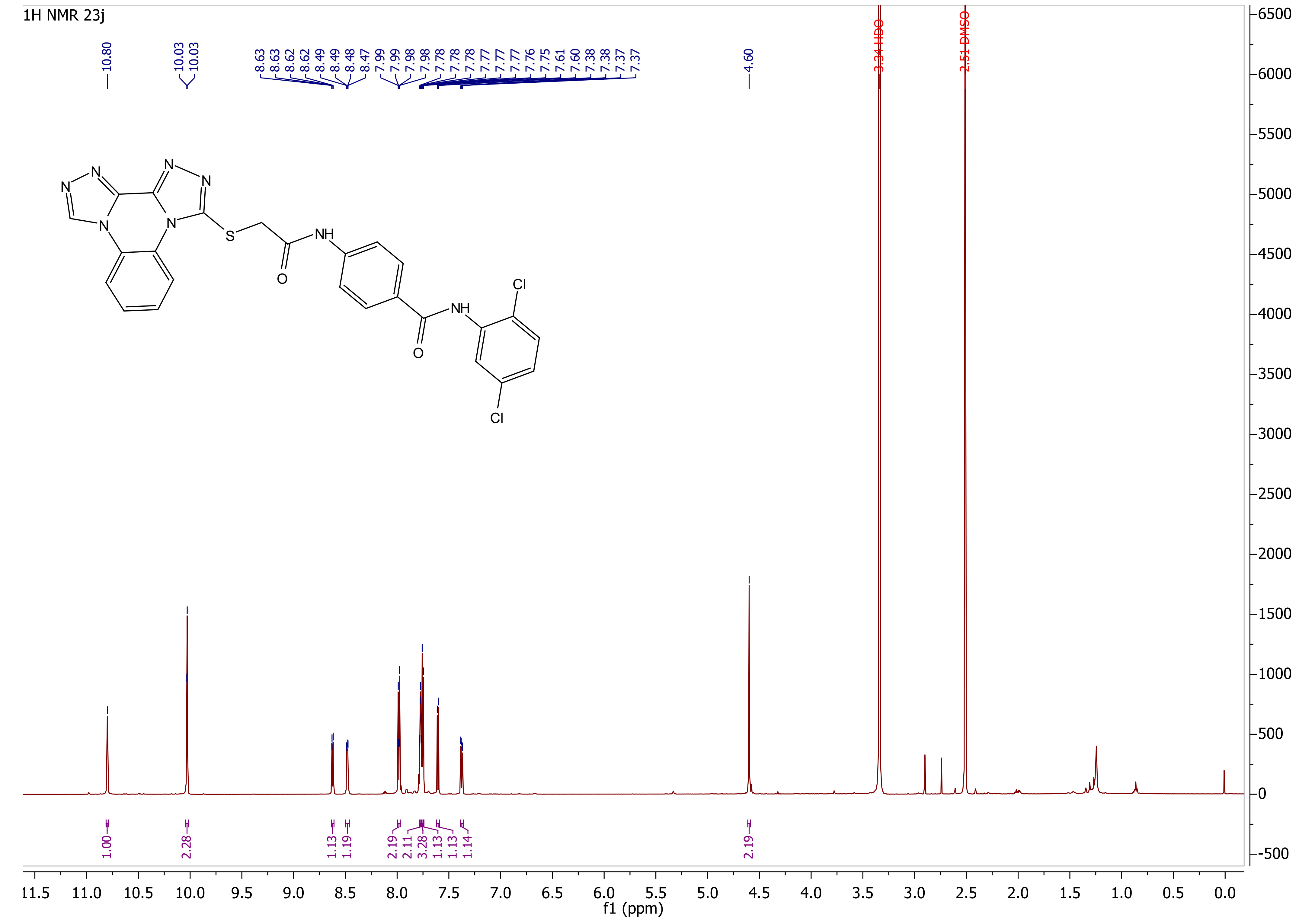
1H NMR 23j
10.80
10.03
10.03
8.63
8.63
8.62
8.62
8.49
8.49
8.48
8.47
7.99
7.99
7.98
7.98
7.78
7.78
7.78
7.77
7.77
7.77
7.76
7.75
7.61
7.60
7.38
7.38
7.37
7.37
4.60
3.34 HDO
2.51 DMSO
N
N
N
N
N
N
S
NH
O
NH
O
Cl
Cl
1.00
2.28
1.13
1.19
2.19
2.11
3.28
1.13
1.13
1.14
2.19
f1 (ppm)

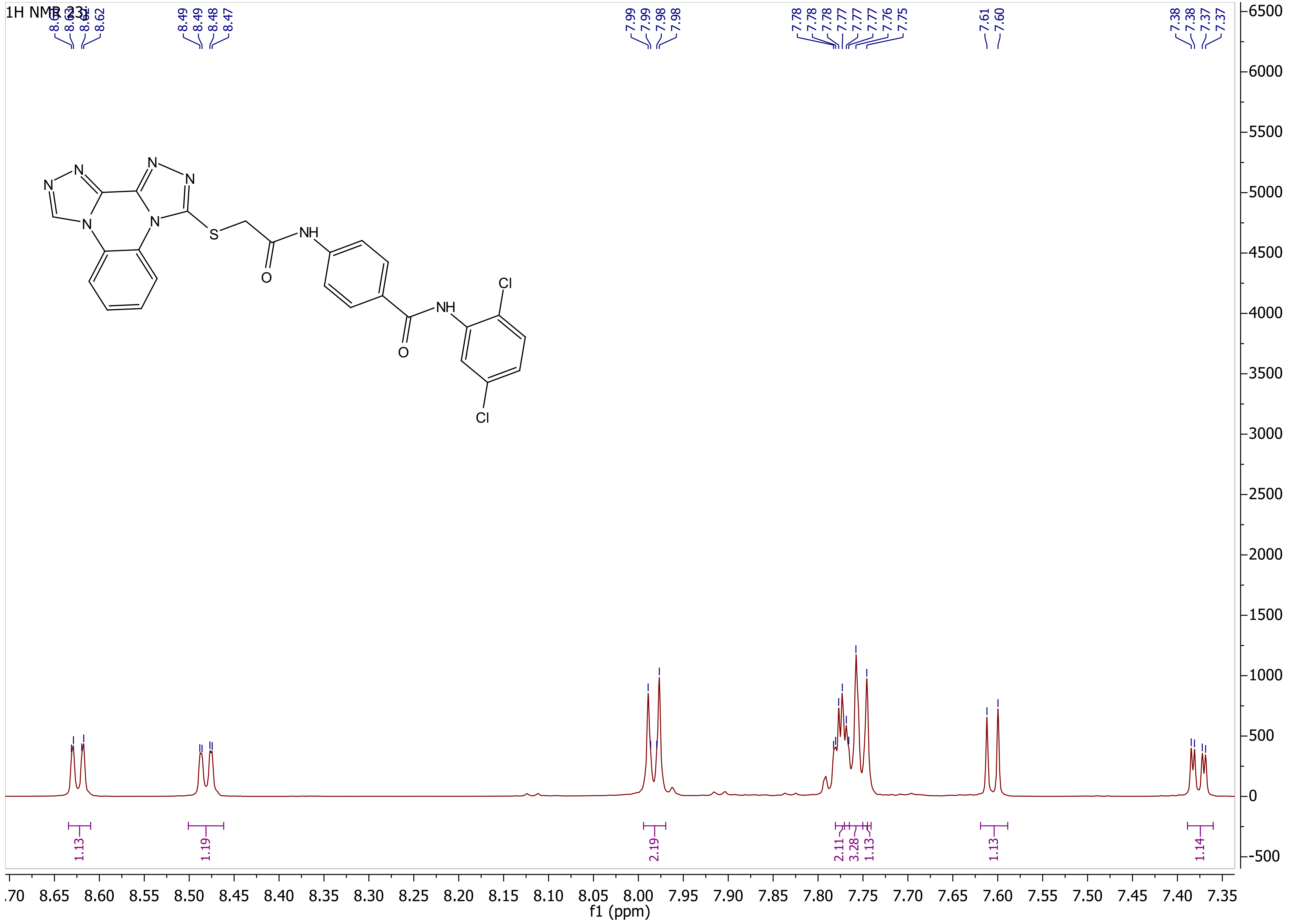
1H NMR 23j
8.62
8.49
8.49
8.48
8.47
7.99
7.99
7.98
7.98
7.78
7.78
7.78
7.77
7.77
7.77
7.76
7.75
7.61
7.60
7.38
7.38
7.37
7.37
N
N
N
N
N
N
S
NH
O
NH
O
Cl
Cl
1.13
1.19
2.19
2.11
3.28
1.13
1.13
1.14
6500
6000
5500
5000
4500
4000
3500
3000
2500
2000
1500
1000
500
0
-500
8.65 8.60 8.55 8.50 8.45 8.40 8.35 8.30 8.25 8.20 8.15 8.10 8.05 8.00 7.95 7.90 7.85 7.80 7.75 7.70 7.65 7.60 7.55 7.50 7.45 7.40 7.35
f1 (ppm)

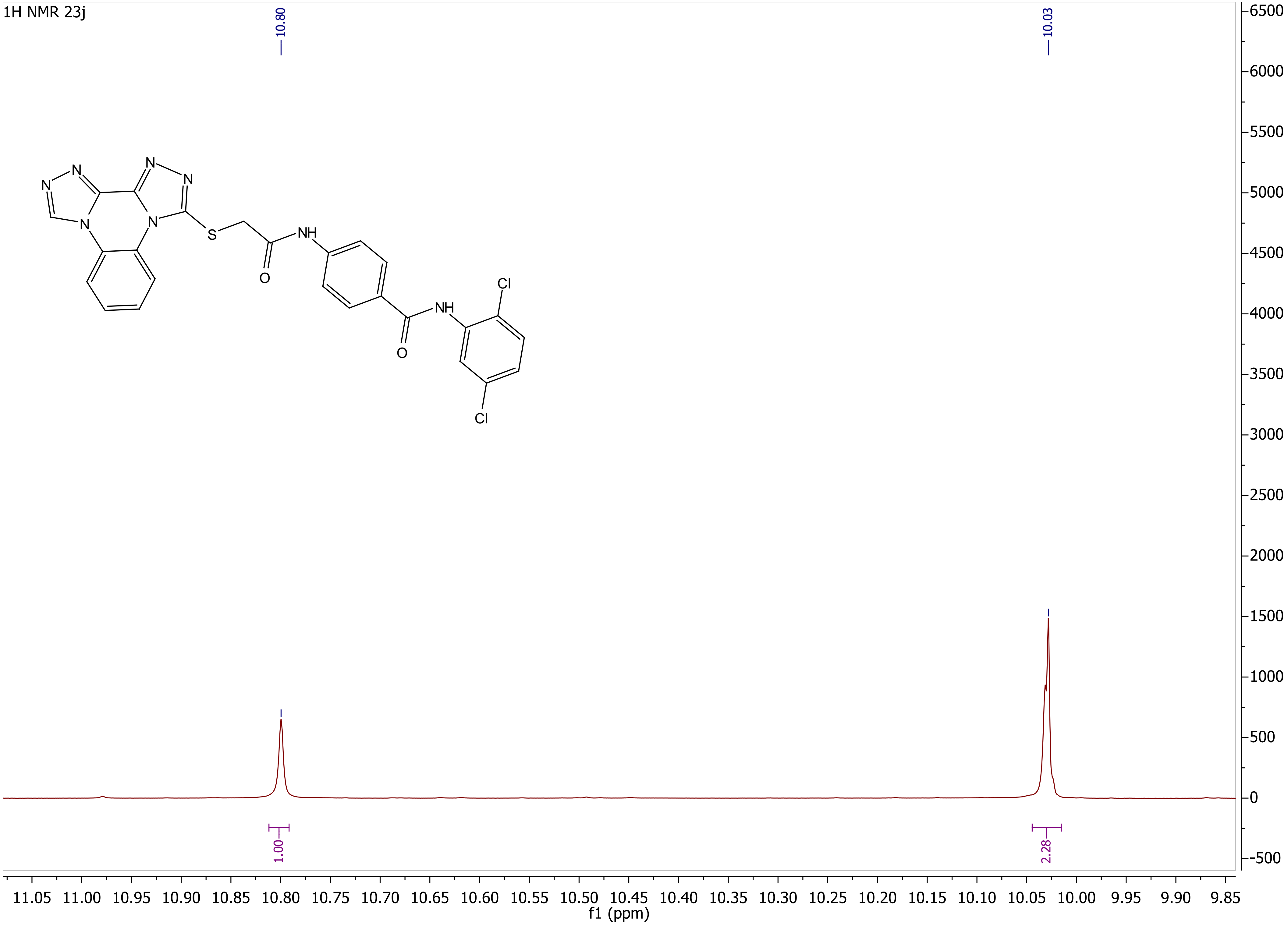

1H NMR 23j
10.80
10.03
1.00
2.28
f1 (ppm)
11.05 11.00 10.95 10.90 10.85 10.80 10.75 10.70 10.65 10.60 10.55 10.50 10.45 10.40 10.35 10.30 10.25 10.20 10.15 10.10 10.05 10.00 9.95 9.90 9.85
6500 6000 5500 5000 4500 4000 3500 3000 2500 2000 1500 1000 500 0 -500
N
N
N
N
N
N
S
NH
O
NH
O
Cl
Cl

13C NMR 23j

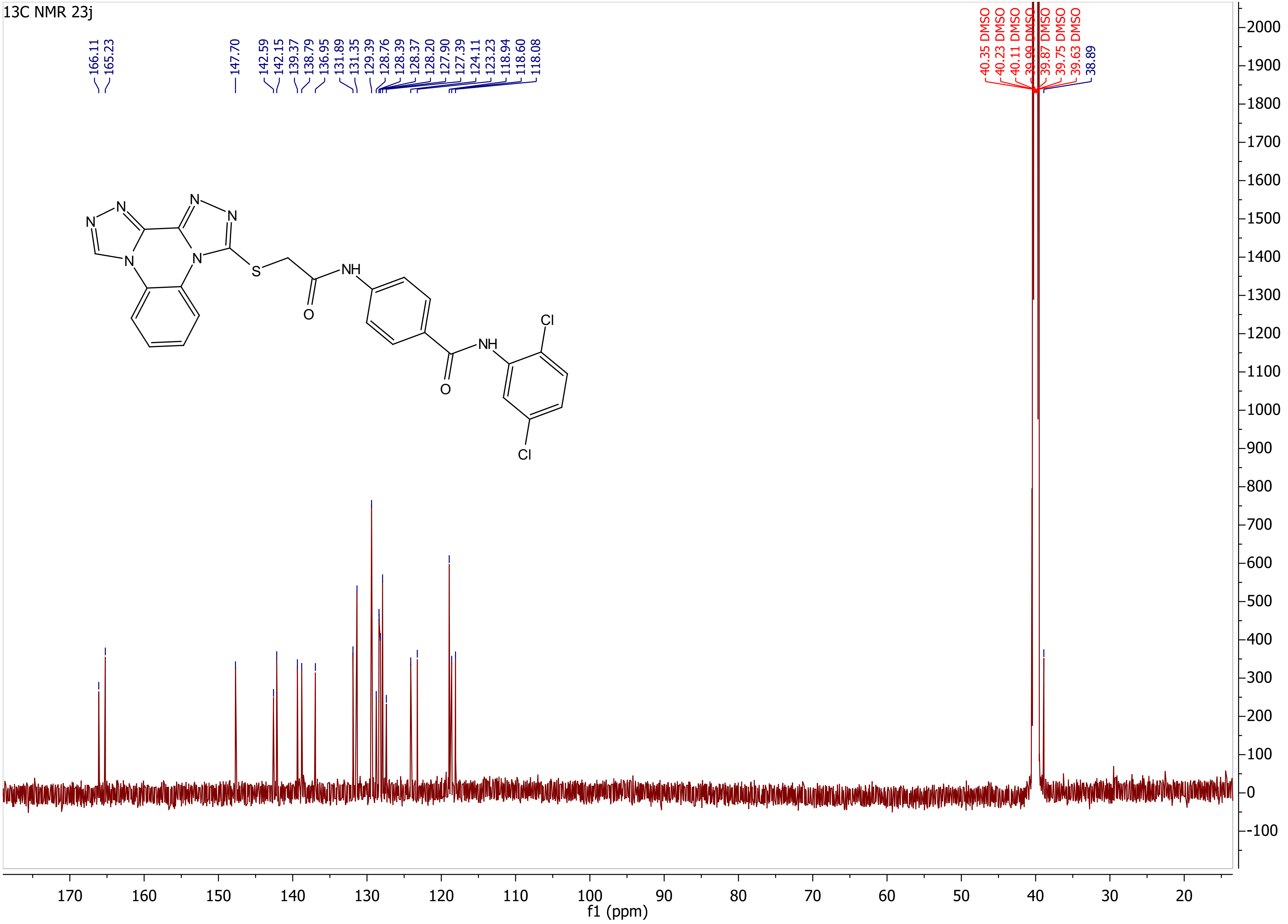

166.11
165.23
147.70
142.59
142.15
139.37
138.79
136.95
131.89
131.35
129.39
128.76
128.39
128.37
128.20
127.90
127.39
124.11
123.23
118.94
118.60
118.08
40.35 DMSO
40.23 DMSO
40.11 DMSO
39.99 DMSO
39.87 DMSO
39.75 DMSO
39.63 DMSO
38.89
f1 (ppm)

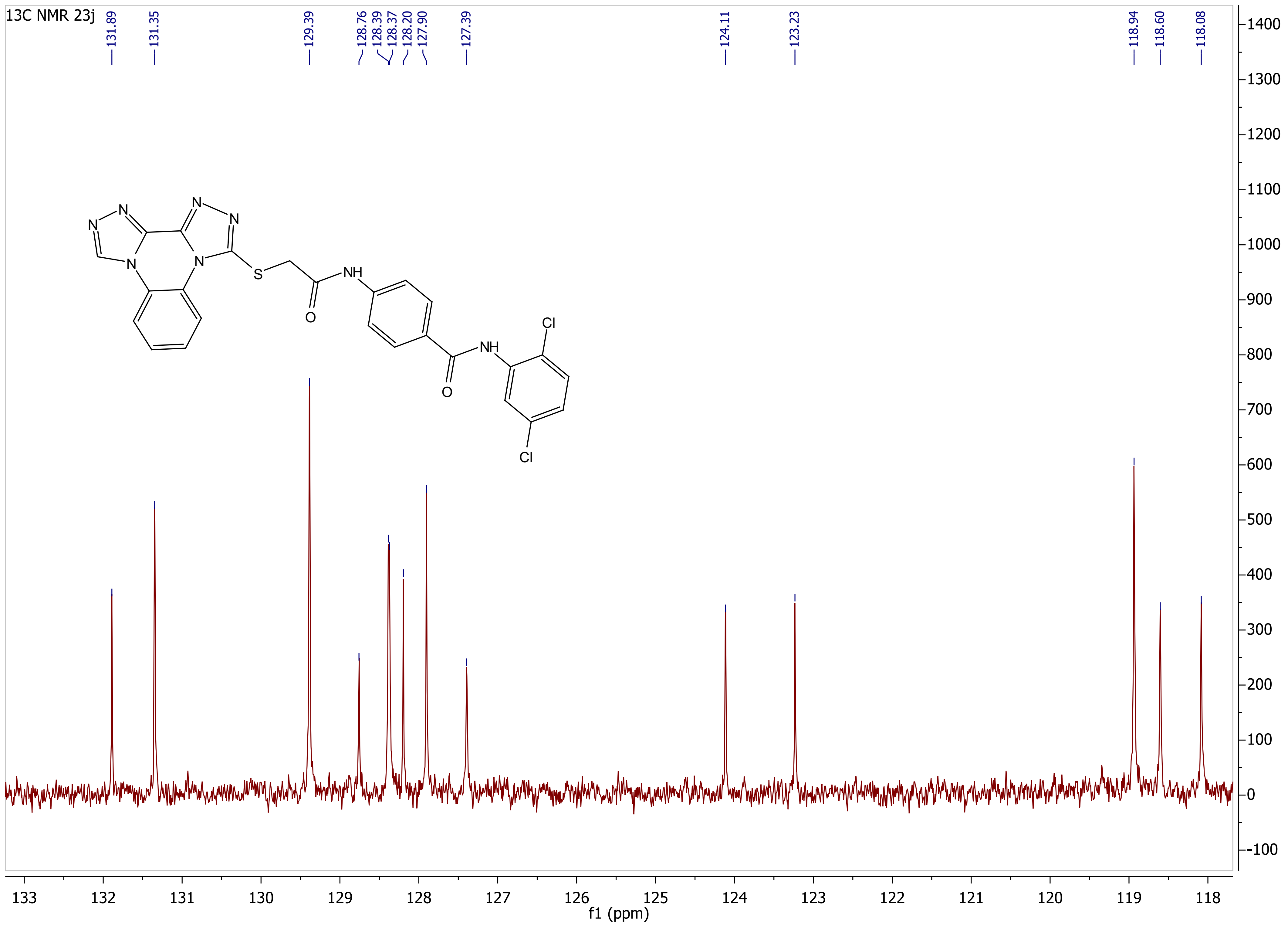
13C NMR 23j
131.89
131.35
129.39
128.76
128.39
128.37
128.20
127.90
127.39
124.11
123.23
118.94
118.60
118.08
N
N
N
N
N
N
S
NH
O
NH
O
Cl
Cl
1400
1300
1200
1100
1000
900
800
700
600
500
400
300
200
100
0
-100
133
132
131
130
129
128
127
126
125
124
123
122
121
120
119
118
f1 (ppm)

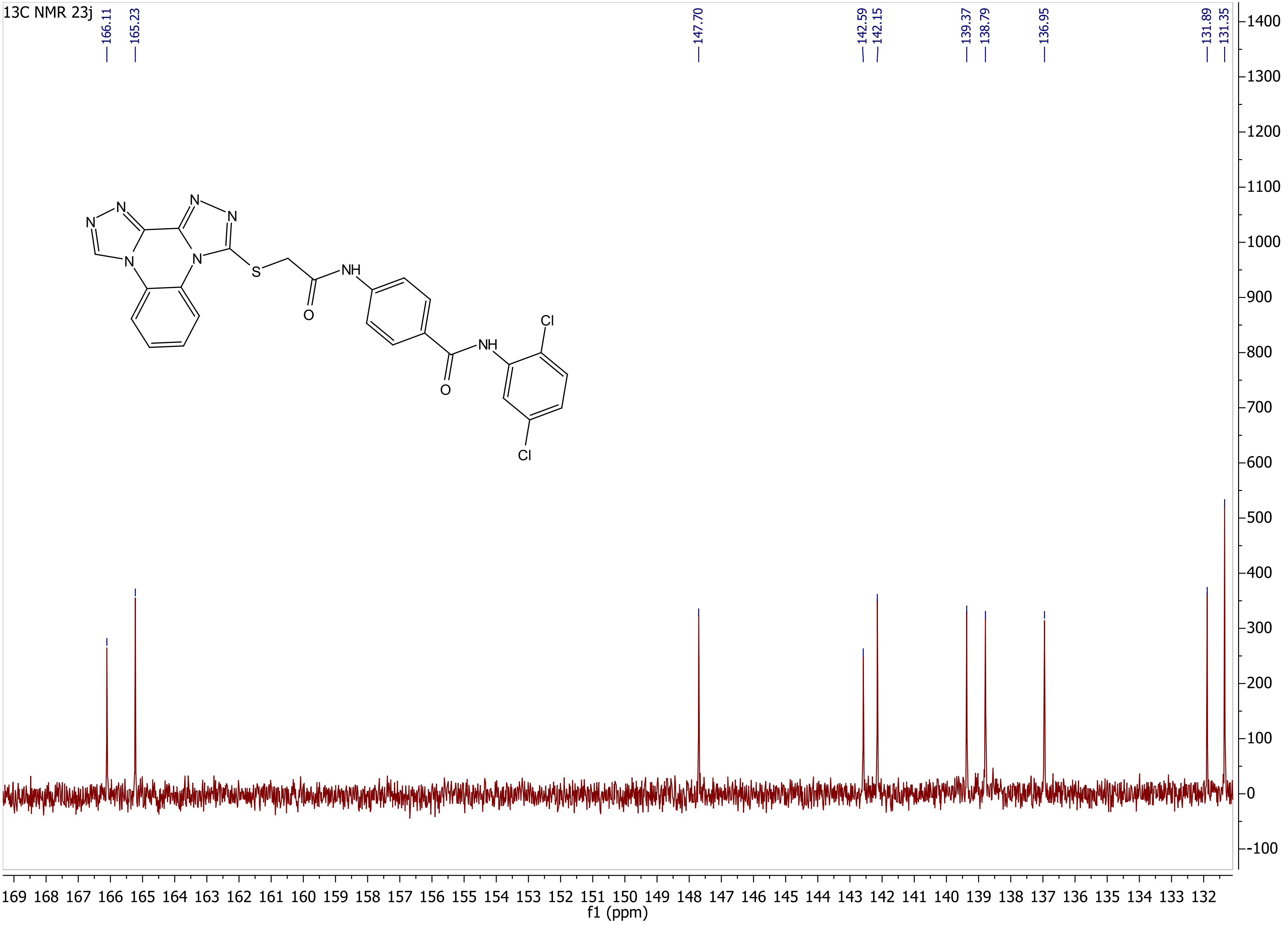
13C NMR 23j
166.11
165.23
147.70
142.59
142.15
139.37
138.79
136.95
131.89
131.35
N
N
N
N
N
N
S
NH
O
NH
O
Cl
Cl
f1 (ppm)

Mass spec. of 23j

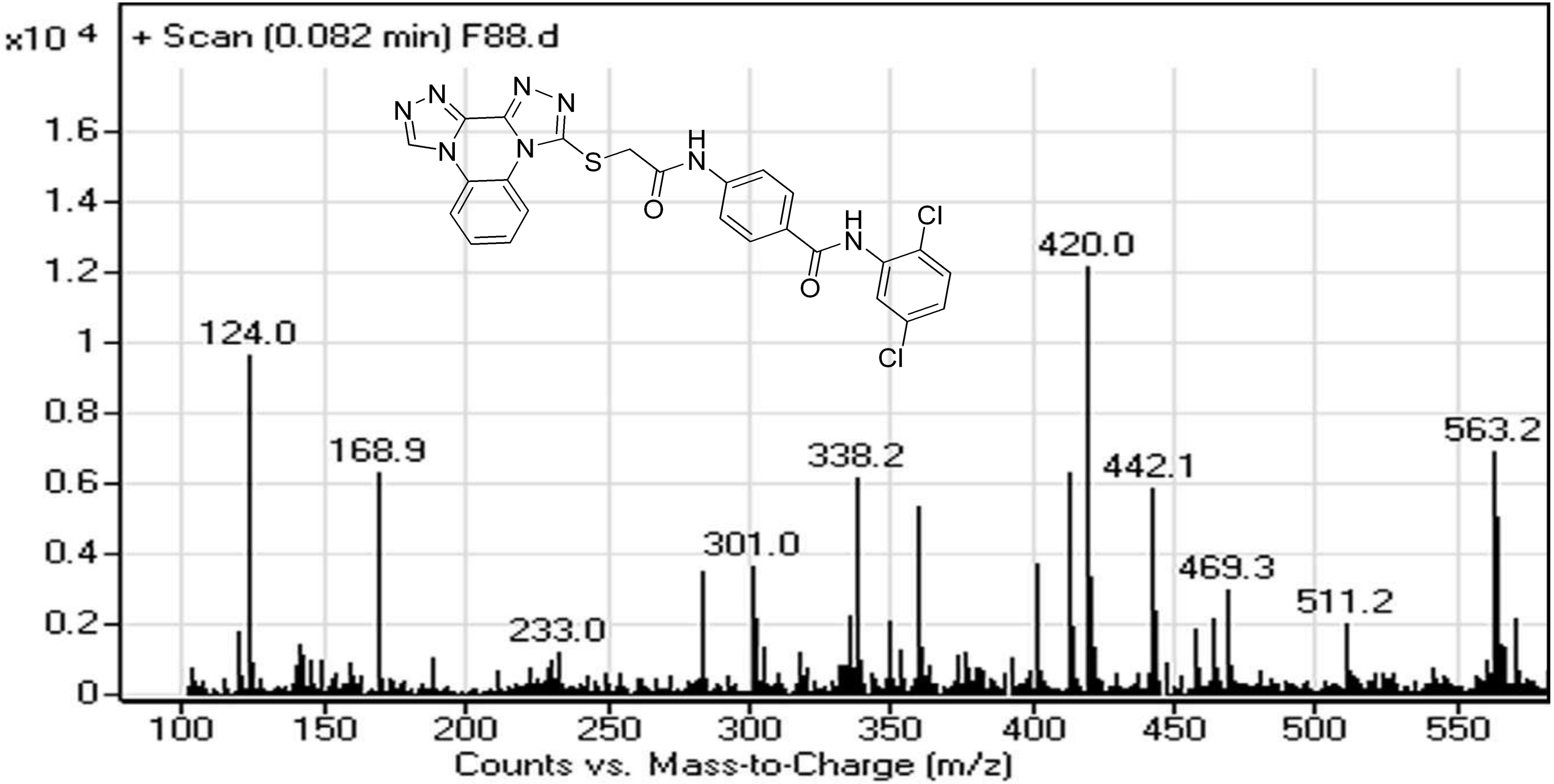

IR of compound 23k

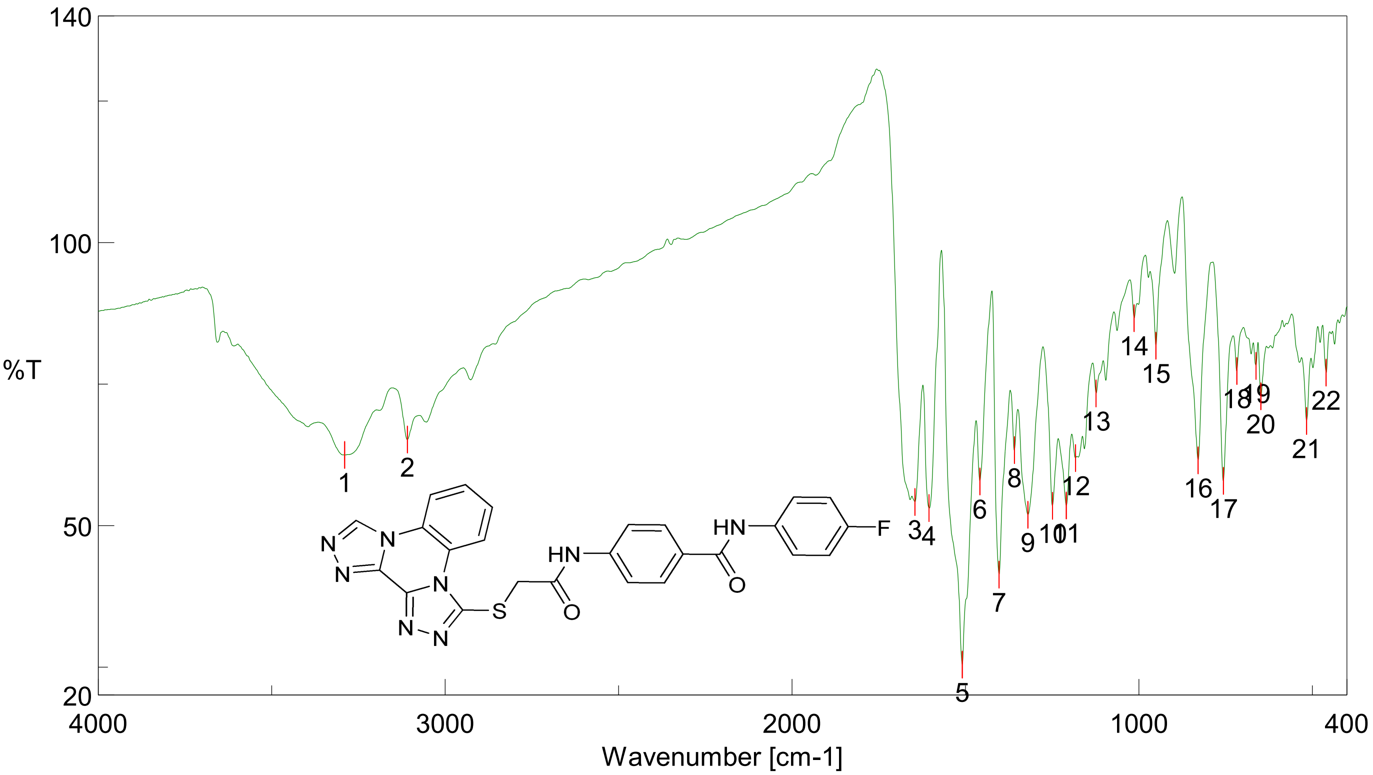

[Comments]

Sample name	F98
Comment	
User	
Division	
Company	KSU

[Detailed Information]

Creation date	10/29/2020 5:15 AM
Data array type	Linear data array
Horizontal axis	Wavenumber [cm-1]
Vertical axis	%T
Start	399.193 cm-1
End	4000.6 cm-1
Data interval	0.964233 cm-1
Data points	3736

[Measurement Information]

Model Name	FT/IR-6600typeA
Serial Number	A014661790
Measurement Date	10/28/2020 4:37 AM
Light Source	Standard
Detector	TGS
Accumulation	Auto (26)
Resolution	4 cm-1
Zero Filling	On
Apodization	Cosine
Gain	Auto (2)
Aperture	Auto (7.1 mm)
Scanning Speed	Auto (2 mm/sec)
Filter	Auto (10000 Hz)

[Result of Peak Picking]

No.	Position	Intensity	No.	Position	Intensity	No.	Position	Intensity
1	3289.96	62.4046	2	3108.69	65.1304	3	1645.95	54.1378

[Result of Peak Picking]

No.	Position	Intensity	No.	Position	Intensity	No.	Position	Intensity
4	1604.48	53.0329	5	1509.03	25.3958	6	1457.92	57.7597
7	1402.96	41.2468	8	1358.6	63.2576	9	1320.04	51.8609
10	1248.68	53.4518	11	1209.15	53.4887	12	1182.15	61.8934
13	1123.33	73.3131	14	1013.41	86.6054	15	950.734	81.7048
16	829.241	61.4653	17	755.959	57.8512	18	717.39	77.2669
19	662.428	78.1371	20	647.965	72.7619	21	515.865	68.4125
22	459.939	76.9502						

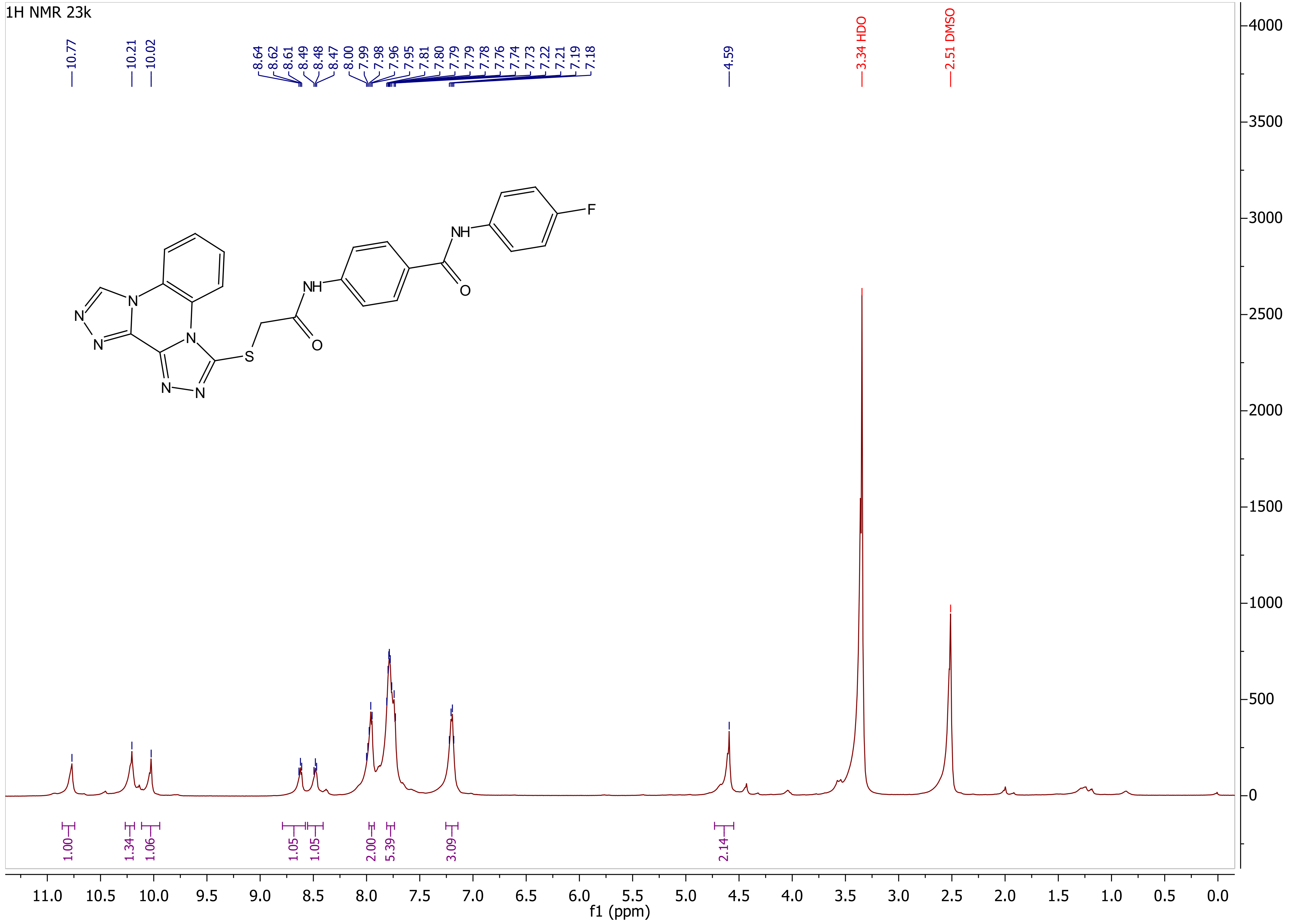
1H NMR 23k
10.77
10.21
10.02
8.64
8.62
8.61
8.49
8.48
8.47
8.00
7.99
7.98
7.96
7.95
7.81
7.80
7.79
7.79
7.78
7.76
7.74
7.73
7.22
7.21
7.19
7.18
4.59
3.34 HDO
2.51 DMSO
NH
F
NH
O
N
N
N
N
N
N
N
S
O
1.00
1.34
1.06
1.05
1.05
2.00
5.39
3.09
2.14
4000
3500
3000
2500
2000
1500
1000
500
0
11.0
10.5
10.0
9.5
9.0
8.5
8.0
7.5
7.0
6.5
6.0
5.5
5.0
4.5
4.0
3.5
3.0
2.5
2.0
1.5
1.0
0.5
0.0
f1 (ppm)

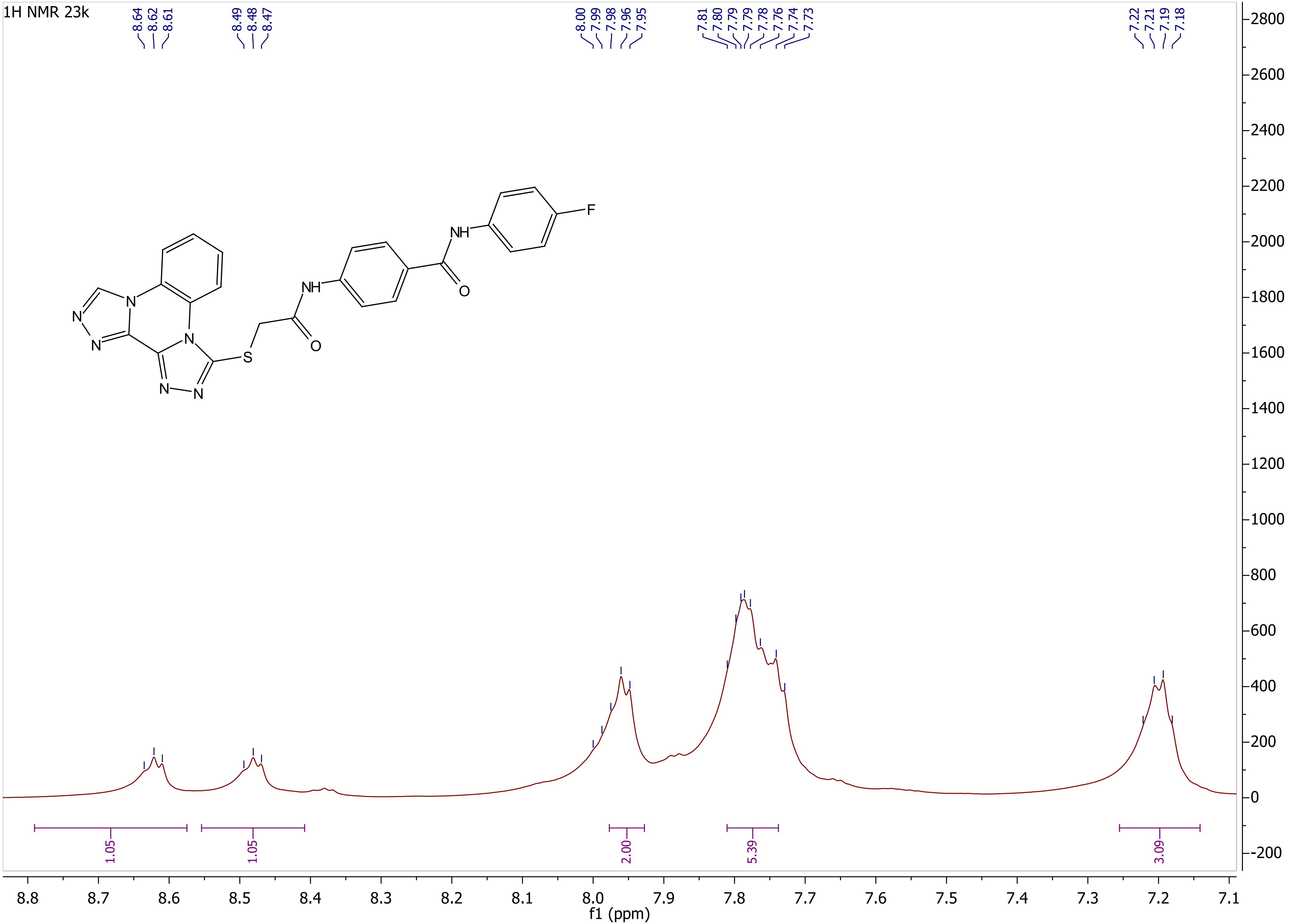
1H NMR 23k
8.64
8.62
8.61
8.49
8.48
8.47
8.00
7.99
7.98
7.96
7.95
7.81
7.80
7.79
7.79
7.78
7.76
7.74
7.73
7.22
7.21
7.19
7.18
NH
F
NH
O
N
N
N
N
S
O
N
N
1.05
1.05
2.00
5.39
3.09
f1 (ppm)

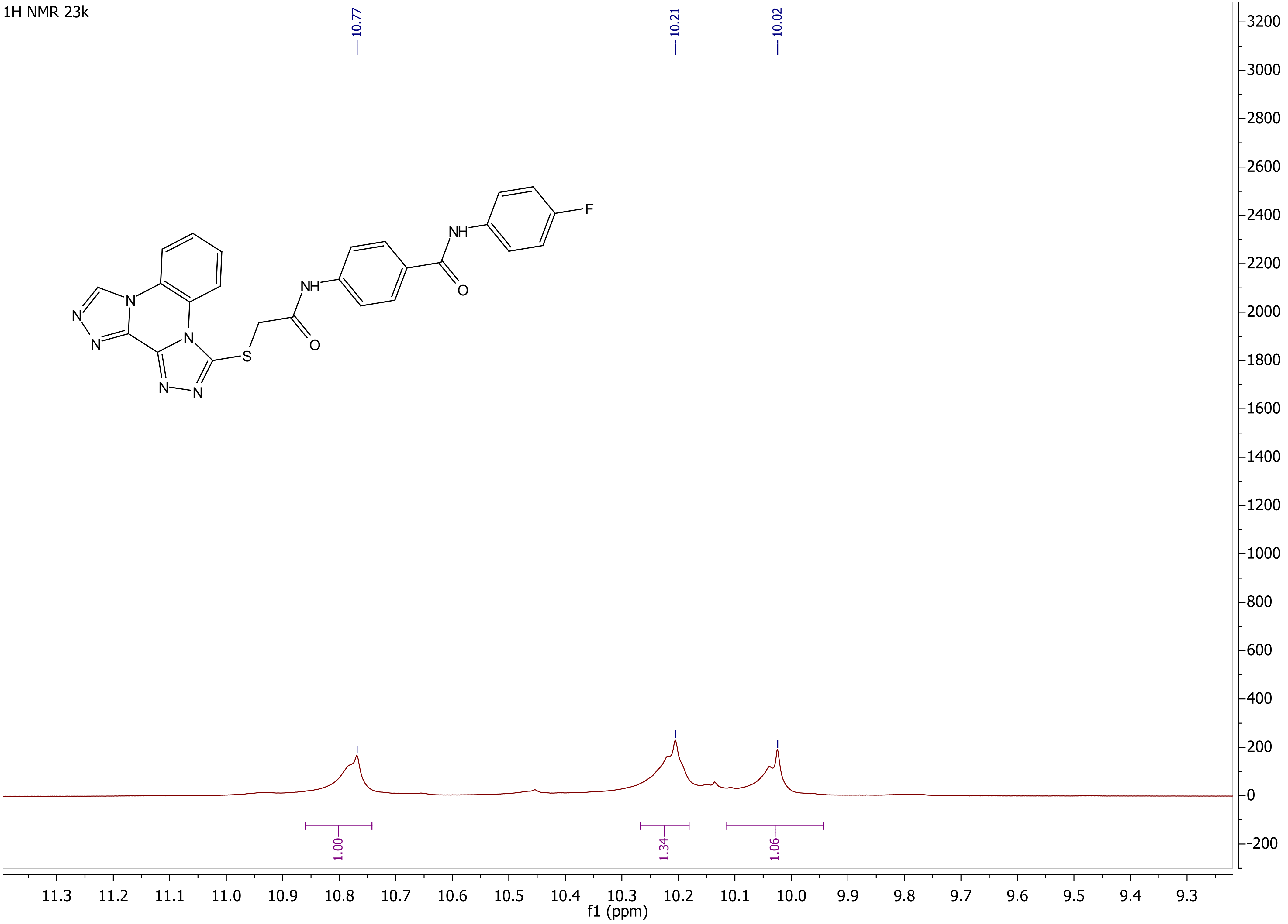
1H NMR 23k
10.77
10.21
10.02
1.00
1.34
1.06
f1 (ppm)

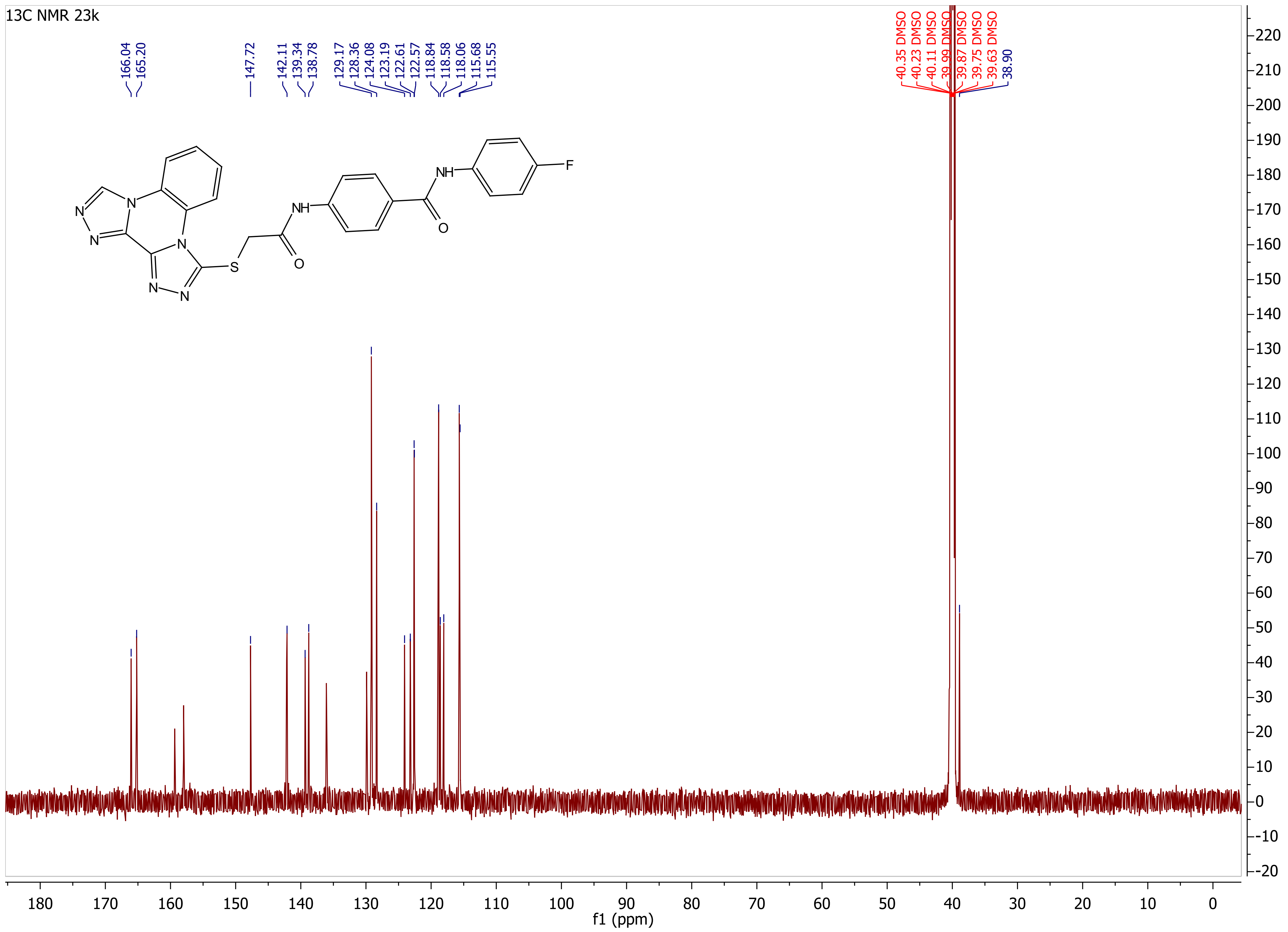
13C NMR 23k
166.04
165.20
147.72
142.11
139.34
138.78
129.17
128.36
124.08
123.19
122.61
122.57
118.84
118.58
118.06
115.68
115.55
40.35 DMSO
40.23 DMSO
40.11 DMSO
39.99 DMSO
39.87 DMSO
39.75 DMSO
39.63 DMSO
38.90
N
N
N
N
N
N
S
NH
O
NH
O
F
f1 (ppm)

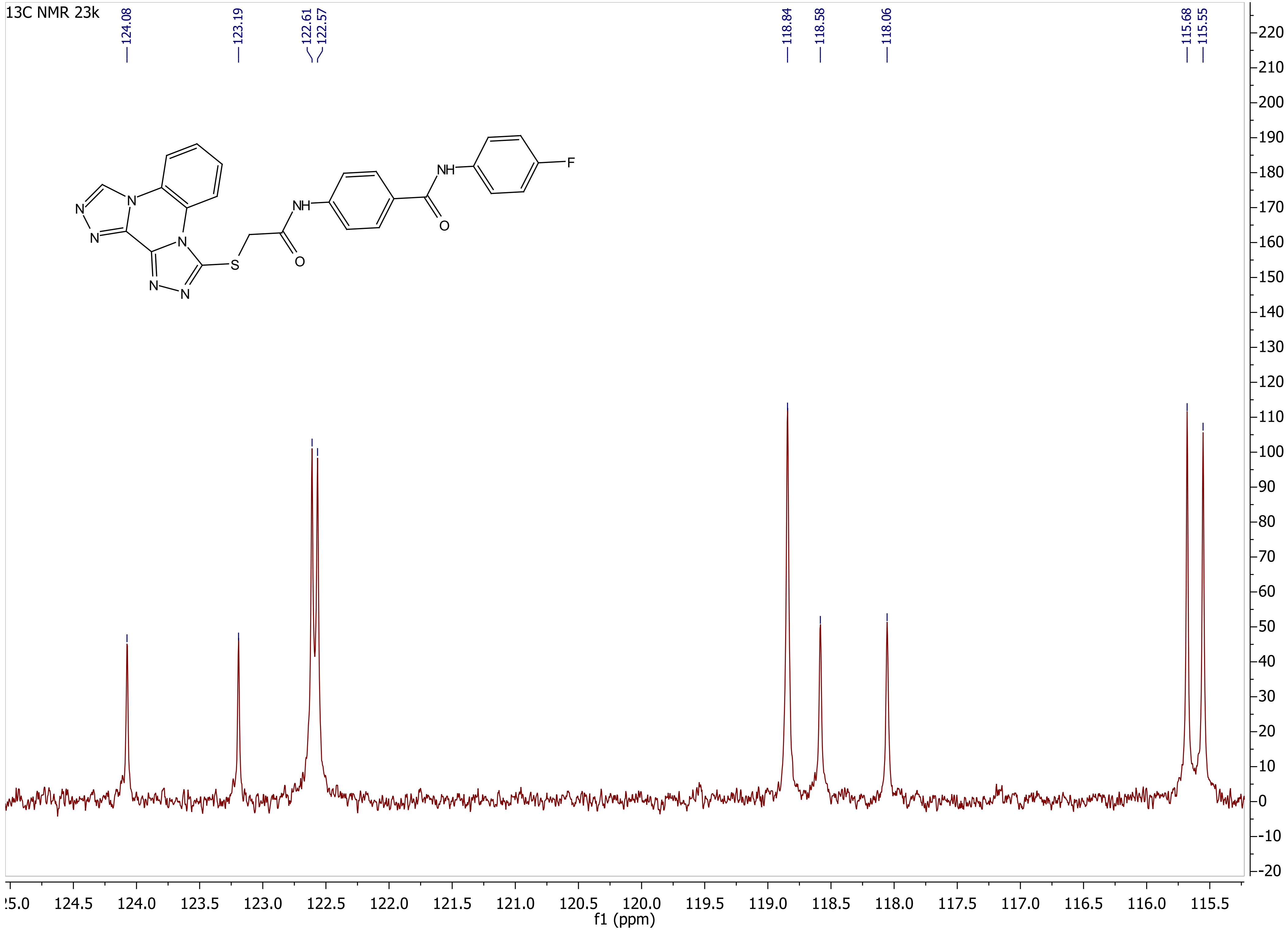
13C NMR 23k
124.08
123.19
122.61
122.57
118.84
118.58
118.06
115.68
115.55
N
N
N
N
N
N
S
NH
O
NH
O
F
f1 (ppm)
124.5
124.0
123.5
123.0
122.5
122.0
121.5
121.0
120.5
120.0
119.5
119.0
118.5
118.0
117.5
117.0
116.5
116.0
115.5
220
210
200
190
180
170
160
150
140
130
120
110
100
90
80
70
60
50
40
30
20
10
0
-10
-20

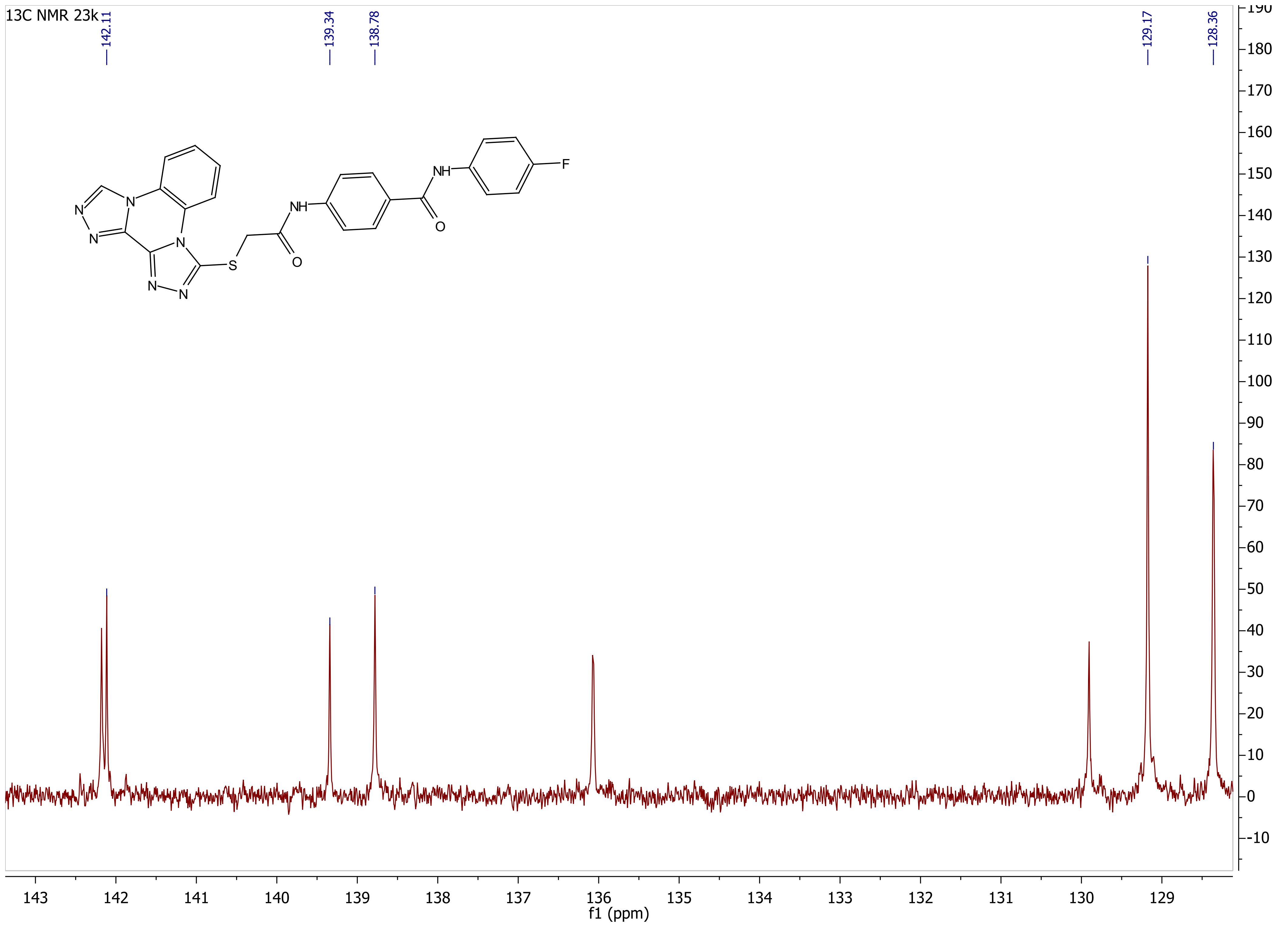

13C NMR 23k
142.11
139.34
138.78
129.17
128.36
f1 (ppm)

13C NMR 23k

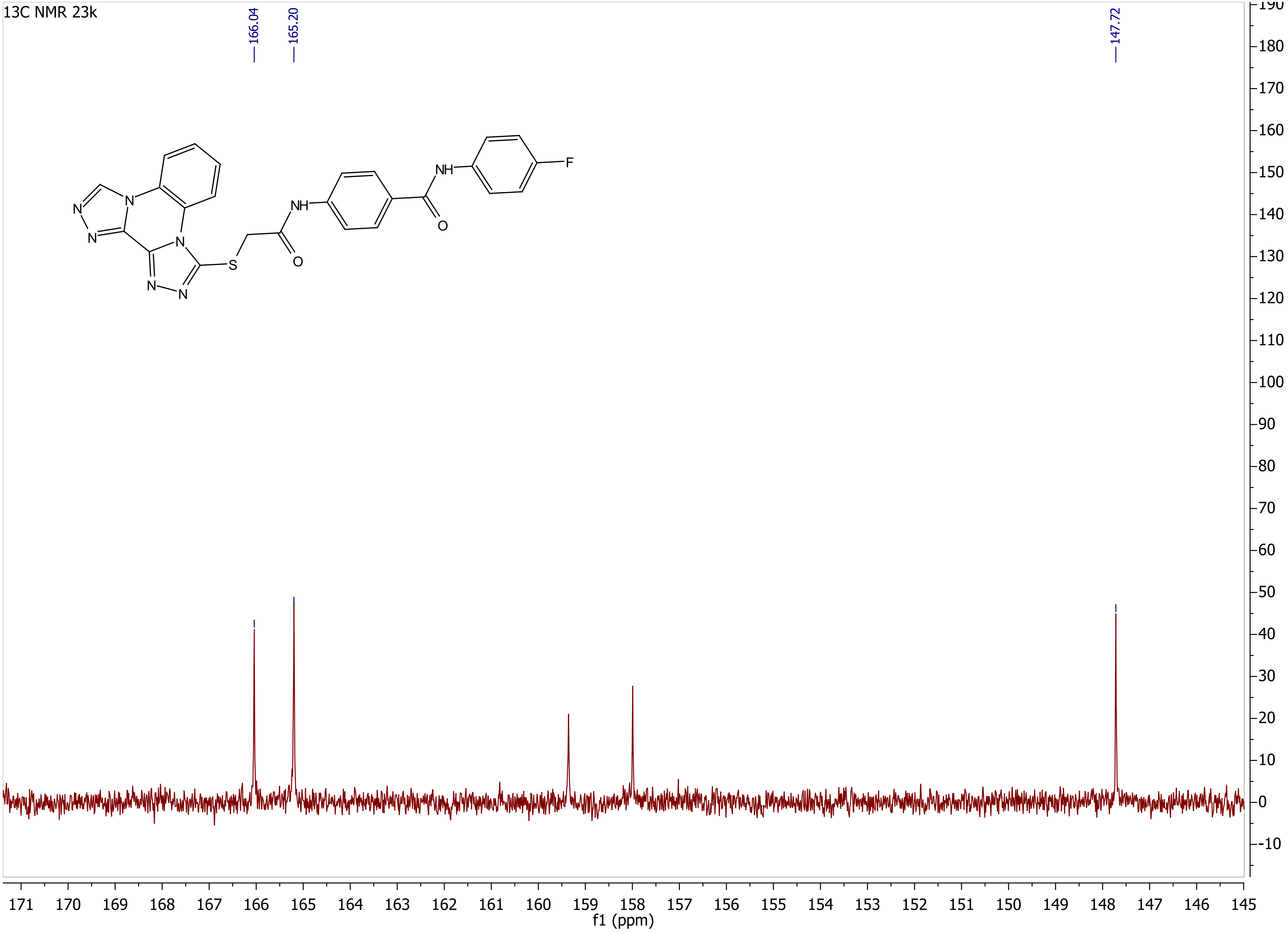
166.04
165.20
147.72
NH
NH
F
O
O
S
N
N
N
N
N
N
171
170
169
168
167
166
165
164
163
162
161
160
159
158
157
156
155
154
153
152
151
150
149
148
147
146
145
f1 (ppm)
190
180
170
160
150
140
130
120
110
100
90
80
70
60
50
40
30
20
10
0
-10

Mass spec. of 23k

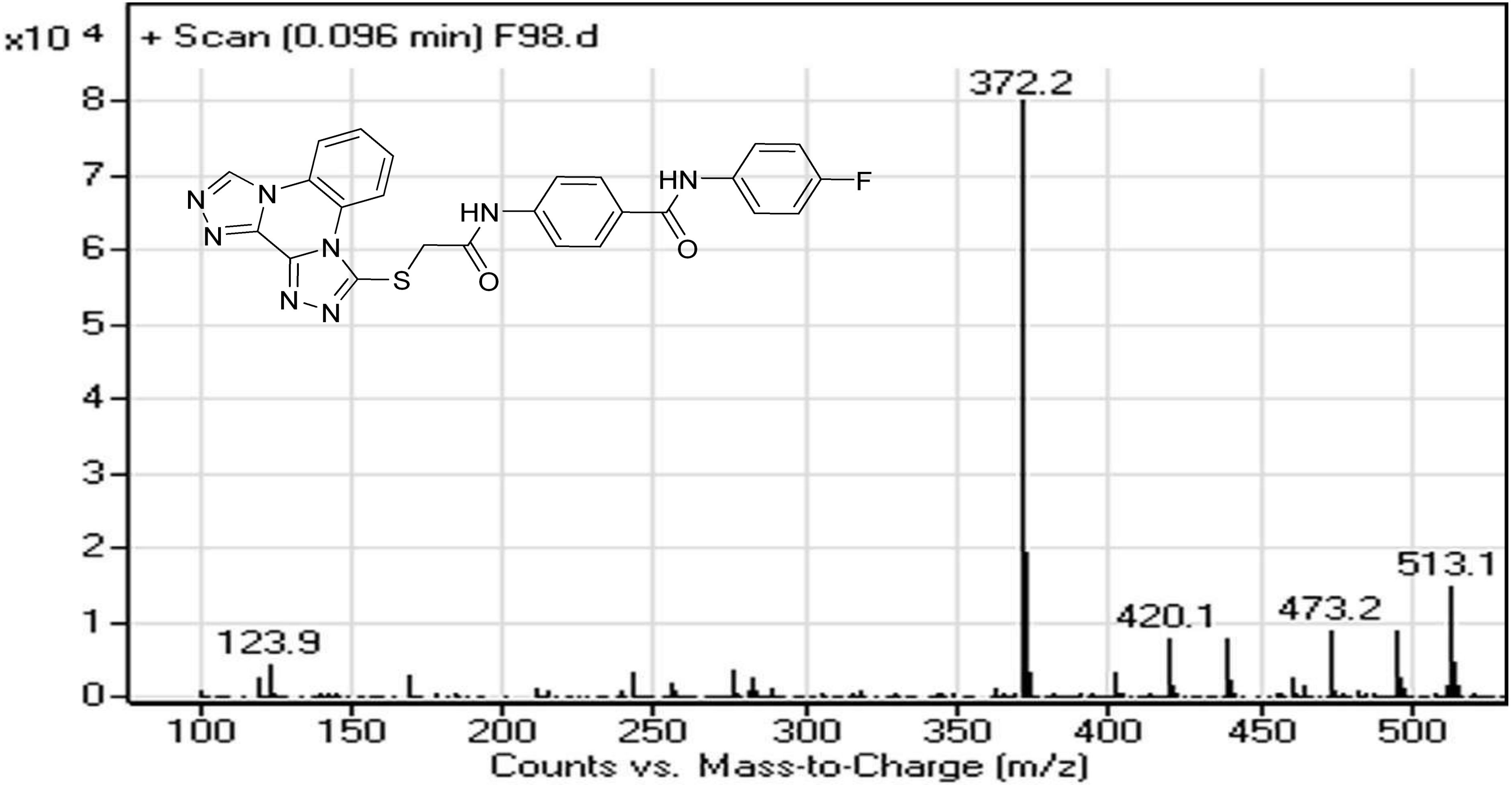

IR of compound 23l

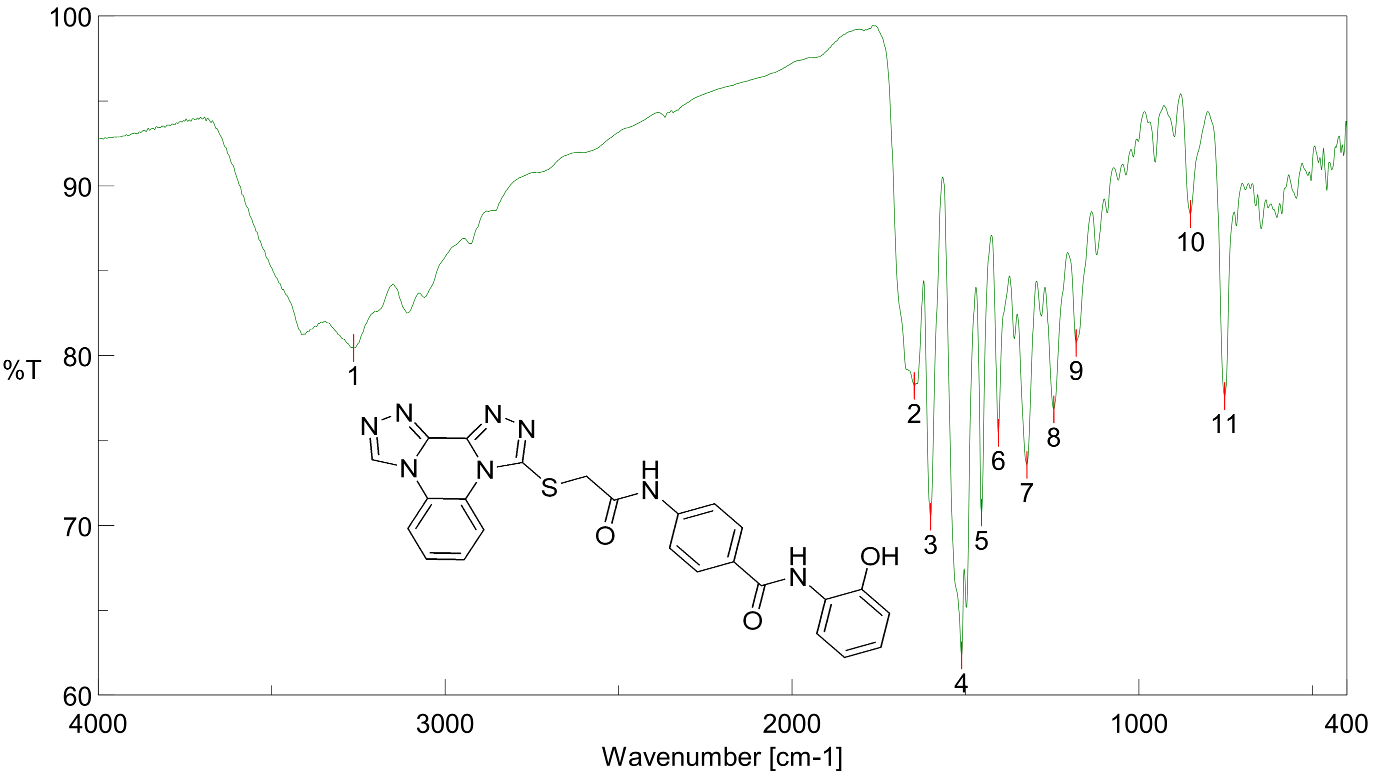

[Comments]

Sample name	F96
Comment	
User	
Division	
Company	KSU

[Detailed Information]

Creation date	10/29/2020 5:14 AM
Data array type	Linear data array
Horizontal axis	Wavenumber [cm-1]
Vertical axis	%T
Start	399.193 cm-1
End	4000.6 cm-1
Data interval	0.964233 cm-1
Data points	3736

[Measurement Information]

Model Name	FT/IR-6600typeA
Serial Number	A014661790
Measurement Date	10/28/2020 4:32 AM
Light Source	Standard
Detector	TGS
Accumulation	Auto (15)
Resolution	4 cm-1
Zero Filling	On
Apodization	Cosine
Gain	Auto (1)
Aperture	Auto (7.1 mm)
Scanning Speed	Auto (2 mm/sec)
Filter	Auto (10000 Hz)

[Result of Peak Picking]

No.	Position	Intensity	No.	Position	Intensity	No.	Position	Intensity
1	3263.93	80.4387	2	1646.91	78.2207	3	1600.63	70.5104

[Result of Peak Picking]

No.	Position	Intensity	No.	Position	Intensity	No.	Position	Intensity
4	1510.95	62.3469	5	1454.06	70.7569	6	1404.89	75.446
7	1322.93	73.5513	8	1244.83	76.8233	9	1180.22	80.7518
10	851.418	88.324	11	752.102	77.6111			

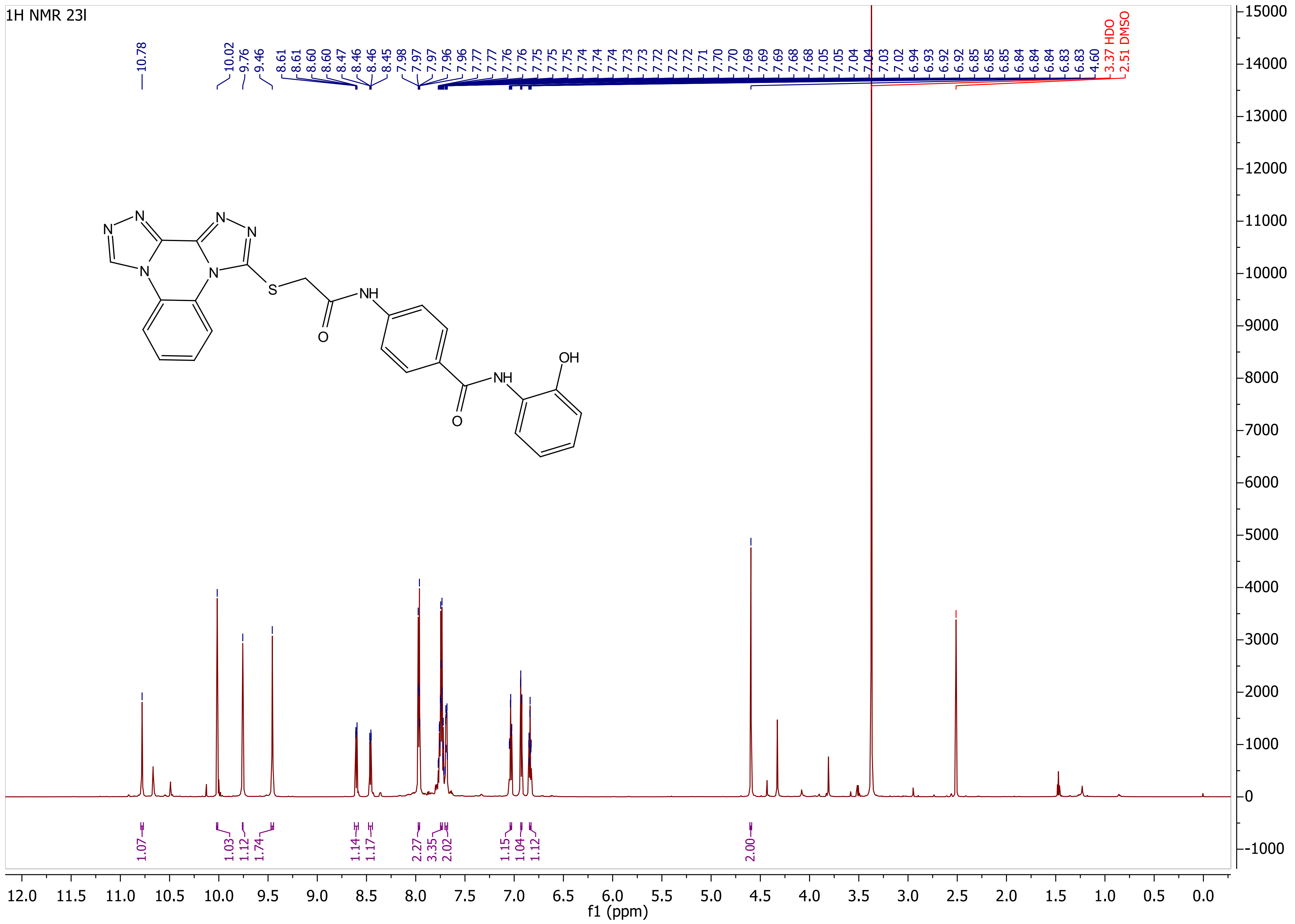

1H NMR 23l
10.78
10.02
9.76
9.46
8.61
8.61
8.60
8.60
8.47
8.46
8.46
8.45
7.98
7.97
7.97
7.96
7.96
7.77
7.77
7.76
7.76
7.75
7.75
7.75
7.74
7.74
7.74
7.73
7.73
7.72
7.72
7.72
7.71
7.70
7.70
7.69
7.69
7.69
7.68
7.68
7.05
7.05
7.04
7.04
7.03
7.02
6.94
6.93
6.92
6.92
6.85
6.85
6.84
6.84
6.84
6.83
6.83
4.60
3.37 HDO
2.51 DMSO
N
N
N
N
N
N
S
NH
O
NH
O
OH
1.07
1.03
1.12
1.74
1.14
1.17
2.27
3.35
2.02
1.15
1.04
1.12
2.00
f1 (ppm)

1H NMR 23l

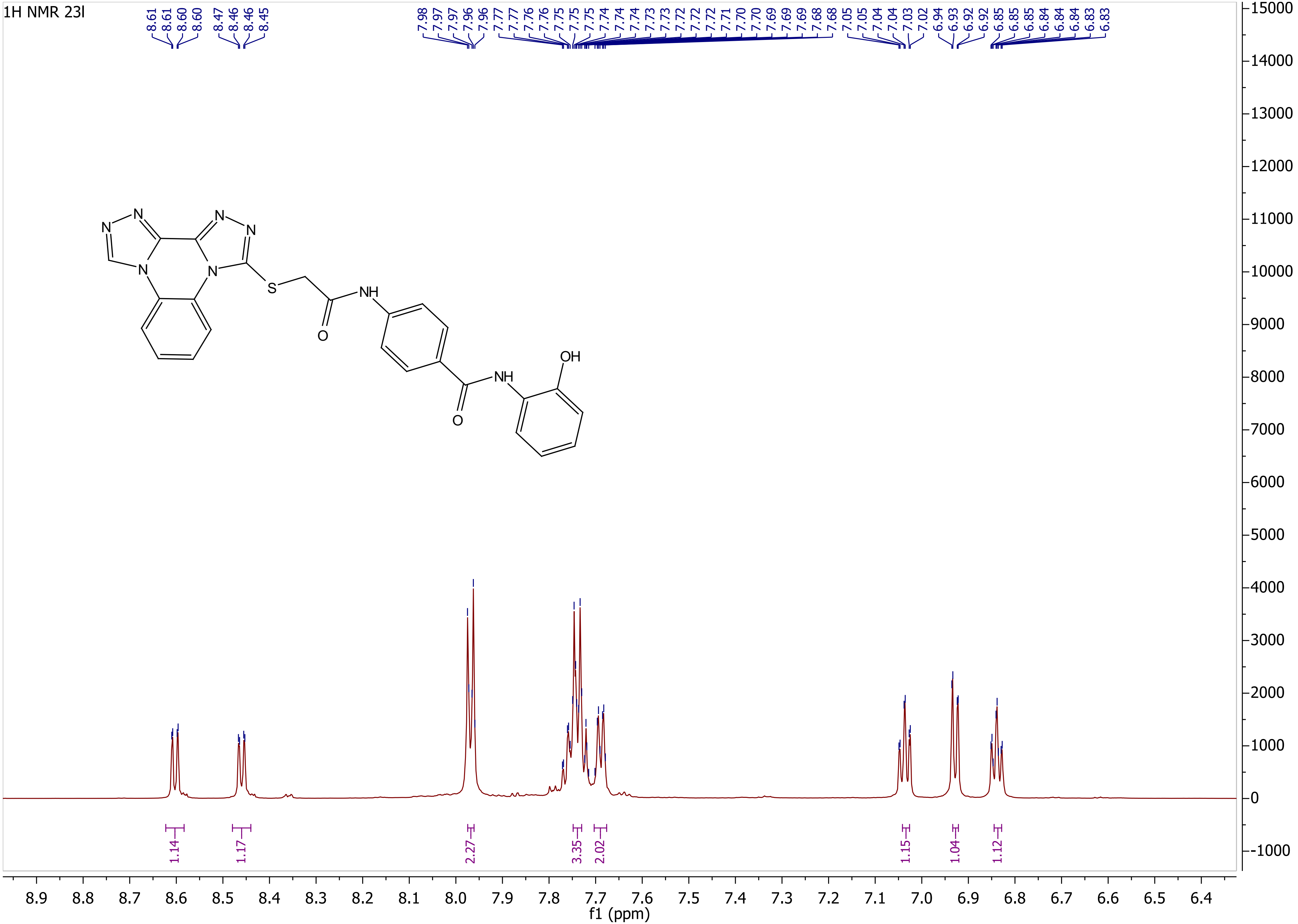

8.61
8.61
8.60
8.60
8.47
8.46
8.46
8.45
7.98
7.97
7.97
7.96
7.96
7.77
7.77
7.76
7.76
7.75
7.75
7.75
7.74
7.74
7.74
7.73
7.73
7.72
7.72
7.72
7.71
7.70
7.70
7.69
7.69
7.69
7.68
7.68
7.05
7.05
7.04
7.04
7.03
7.02
6.94
6.93
6.92
6.92
6.85
6.85
6.85
6.84
6.84
6.84
6.83
6.83
N
N
N
N
N
N
S
NH
O
NH
OH
O
1.14
1.17
2.27
3.35
2.02
1.15
1.04
1.12
f1 (ppm)

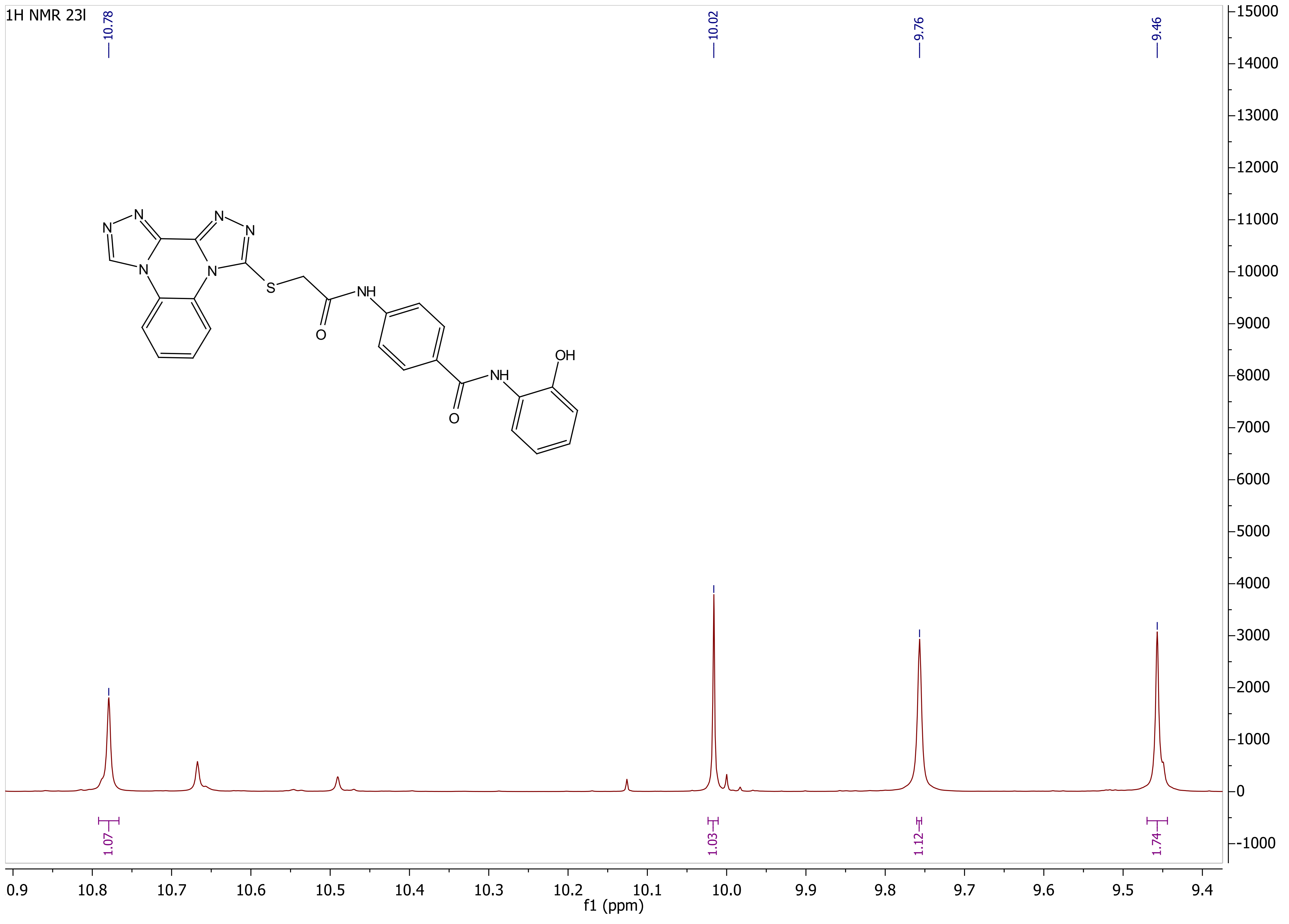
1H NMR 23l
10.78
10.02
9.76
9.46
1.07
1.03
1.12
1.74
f1 (ppm)
N
N
N
N
N
N
S
NH
O
NH
OH
O

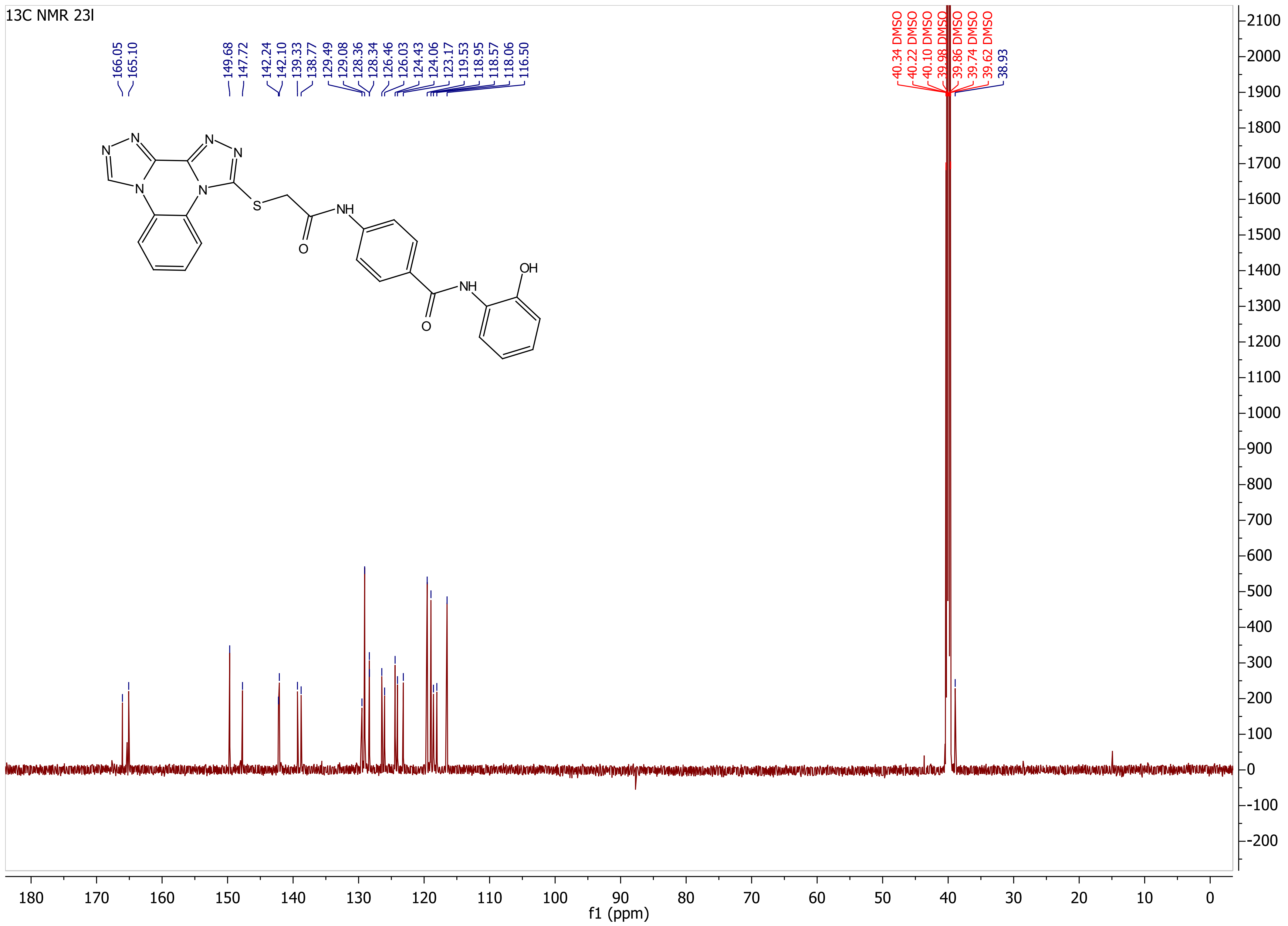
13C NMR 23l
166.05
165.10
149.68
147.72
142.24
142.10
139.33
138.77
129.49
129.08
128.36
128.34
126.46
126.03
124.43
124.06
123.17
119.53
118.95
118.57
118.06
116.50
40.34 DMSO
40.22 DMSO
40.10 DMSO
39.98 DMSO
39.86 DMSO
39.74 DMSO
39.62 DMSO
38.93
N
N
N
N
N
N
S
NH
O
NH
OH
O
f1 (ppm)

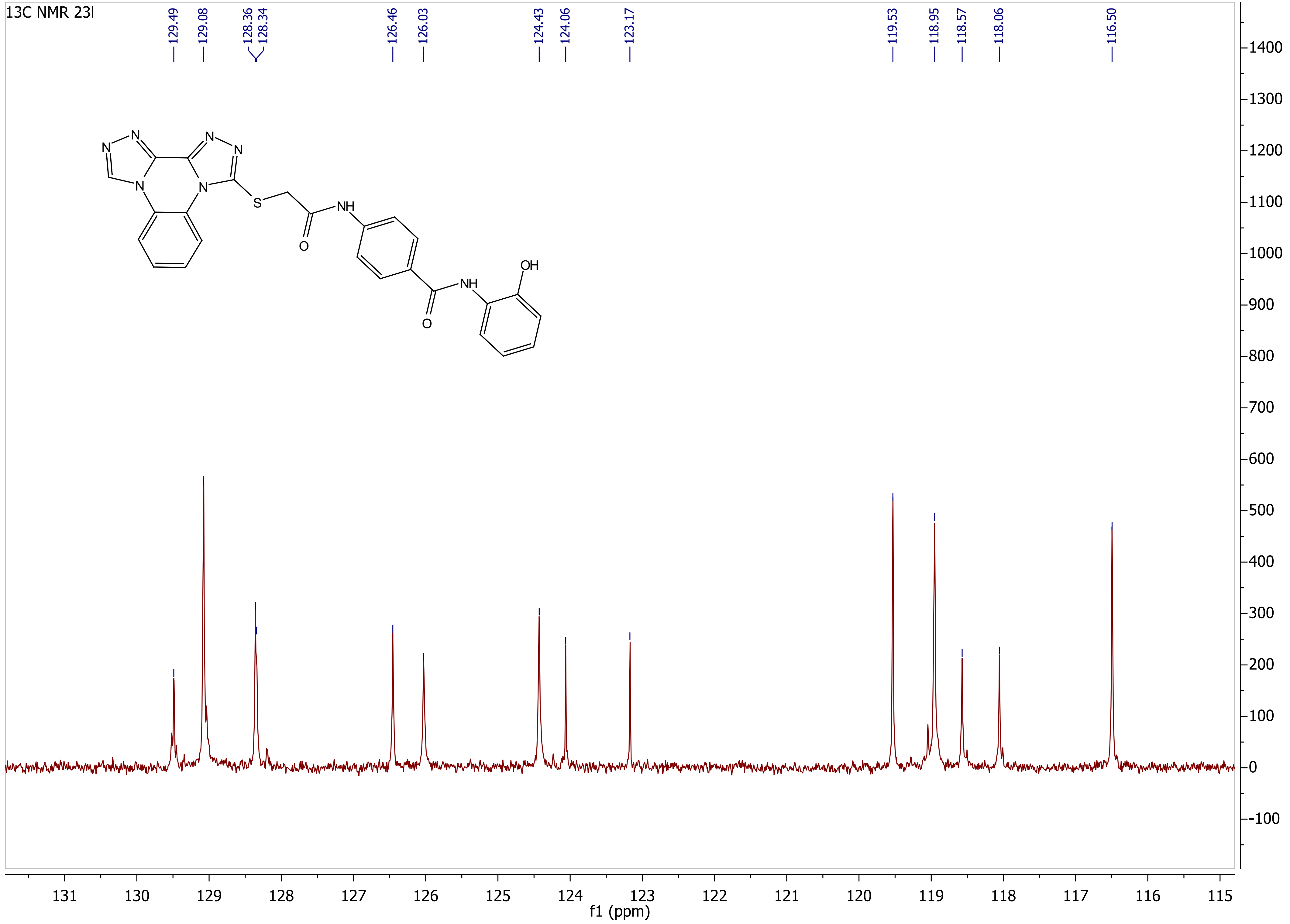
13C NMR 23l
129.49
129.08
128.36
128.34
126.46
126.03
124.43
124.06
123.17
119.53
118.95
118.57
118.06
116.50
N
N
N
N
N
N
S
NH
O
NH
OH
O
f1 (ppm)
131
130
129
128
127
126
125
124
123
122
121
120
119
118
117
116
115
1400
1300
1200
1100
1000
900
800
700
600
500
400
300
200
100
0
-100

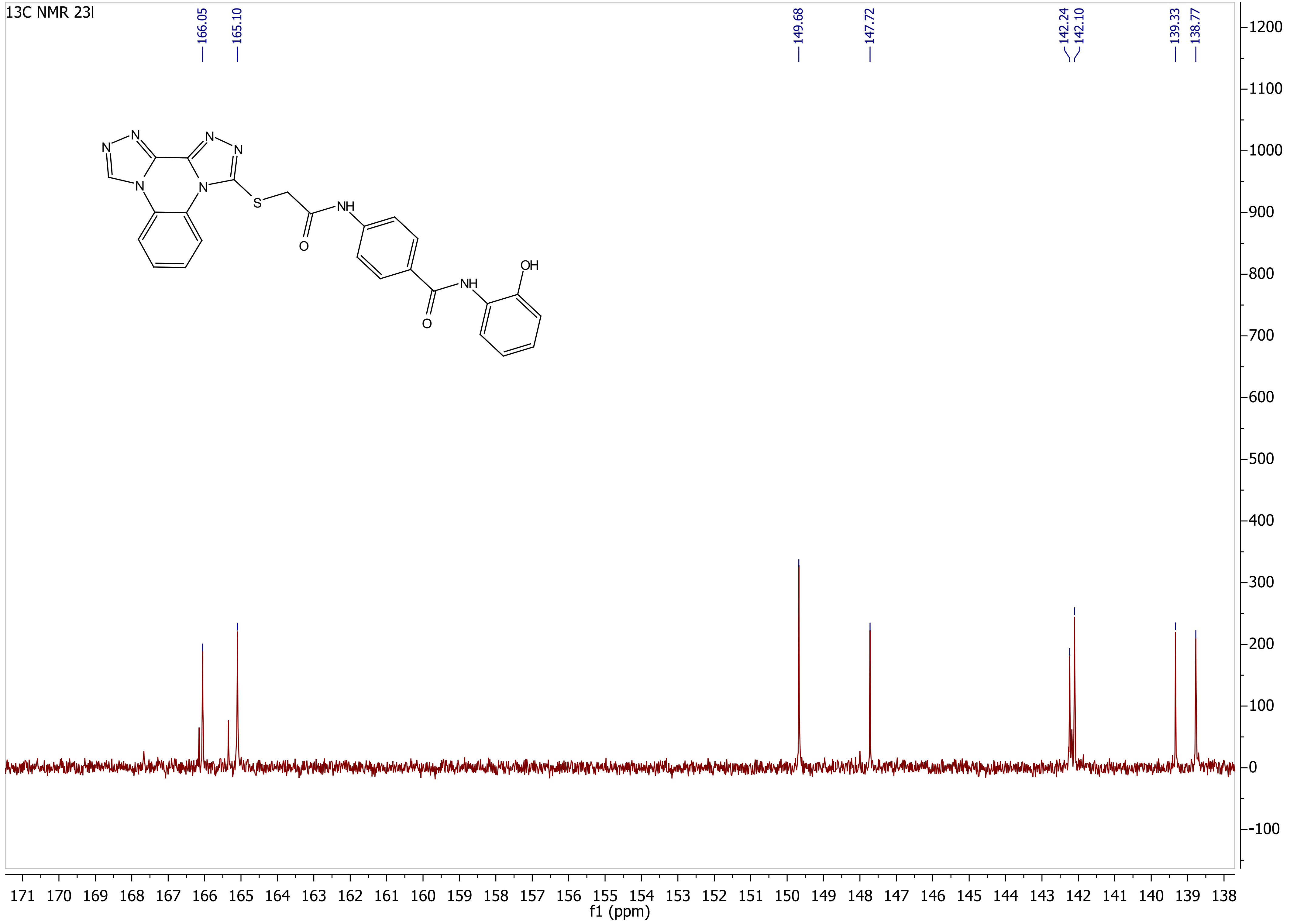
13C NMR 23l
166.05
165.10
149.68
147.72
142.24
142.10
139.33
138.77
N
N
N
N
N
N
N
N
S
O
NH
NH
O
OH
1200
1100
1000
900
800
700
600
500
400
300
200
100
0
-100
171 170 169 168 167 166 165 164 163 162 161 160 159 158 157 156 155 154 153 152 151 150 149 148 147 146 145 144 143 142 141 140 139 138
f1 (ppm)

Mass spec. of 23l

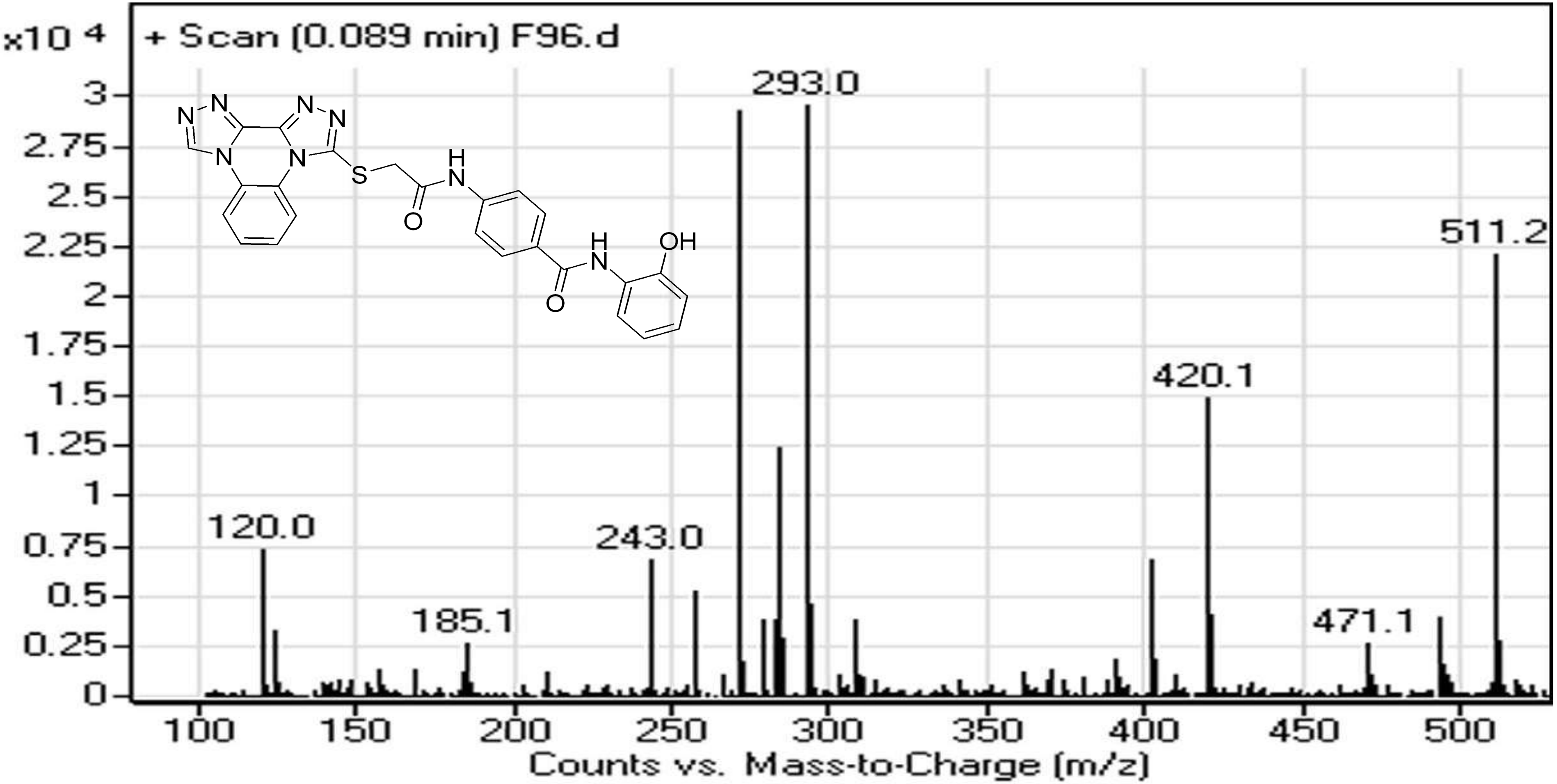

IR of compound 23m

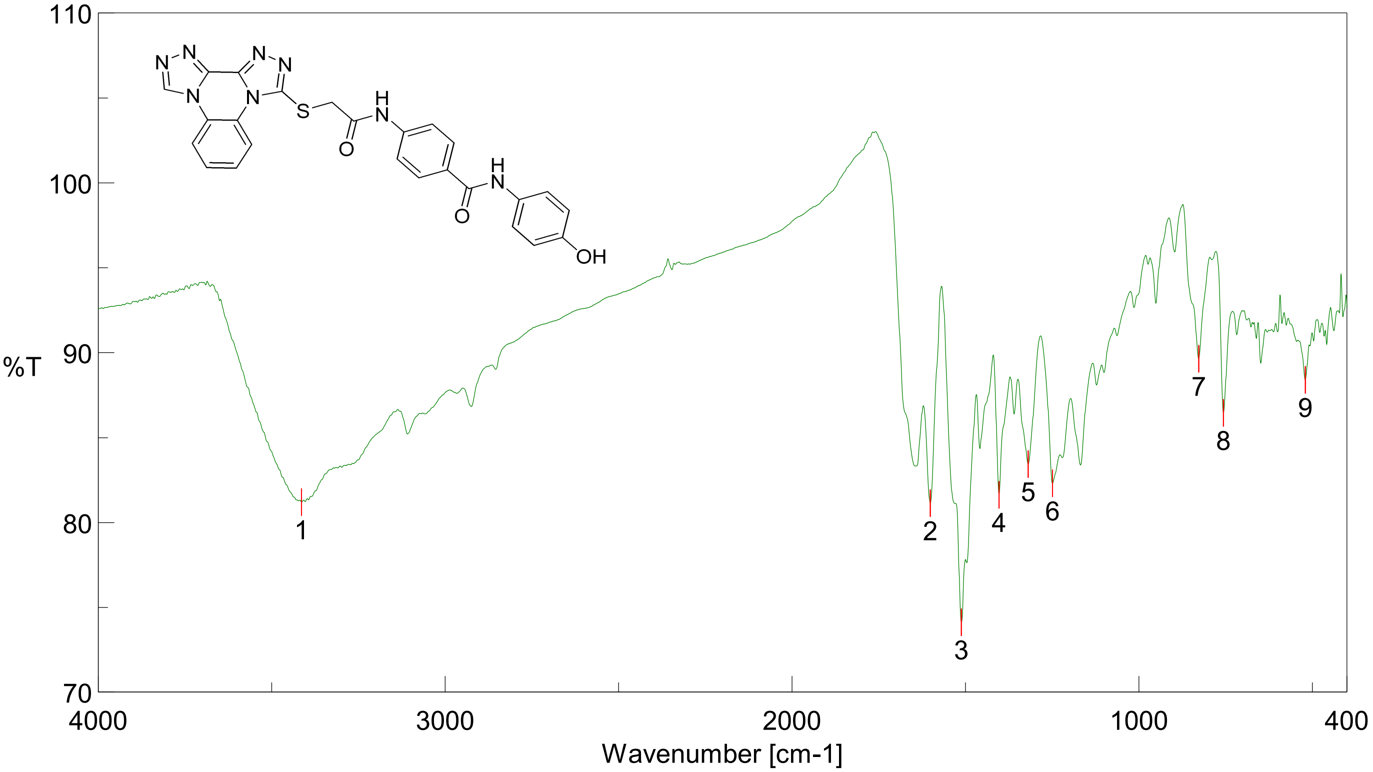

[Comments]

Sample name	F105
Comment	
User	
Division	
Company	KSU

[Detailed Information]

Creation date	11/1/2020 12:19 AM
Data array type	Linear data array
Horizontal axis	Wavenumber [cm-1]
Vertical axis	%T
Start	399.193 cm-1
End	4000.6 cm-1
Data interval	0.964233 cm-1
Data points	3736

[Measurement Information]

Model Name	FT/IR-6600typeA
Serial Number	A014661790
Measurement Date	10/28/2020 5:30 AM
Light Source	Standard
Detector	TGS
Accumulation	Auto (15)
Resolution	4 cm-1
Zero Filling	On
Apodization	Cosine
Gain	Auto (1)
Aperture	Auto (7.1 mm)
Scanning Speed	Auto (2 mm/sec)
Filter	Auto (10000 Hz)

[Result of Peak Picking]

No.	Position	Intensity	No.	Position	Intensity	No.	Position	Intensity
1	3414.35	81.1925	2	1601.59	81.1254	3	1511.92	74.115

[Result of Peak Picking]

No.	Position	Intensity	No.	Position	Intensity	No.	Position	Intensity
4	1402.96	81.6181	5	1319.07	83.4273	6	1248.68	82.2875
7	827.312	89.6385	8	755.959	86.4508	9	519.722	88.3957

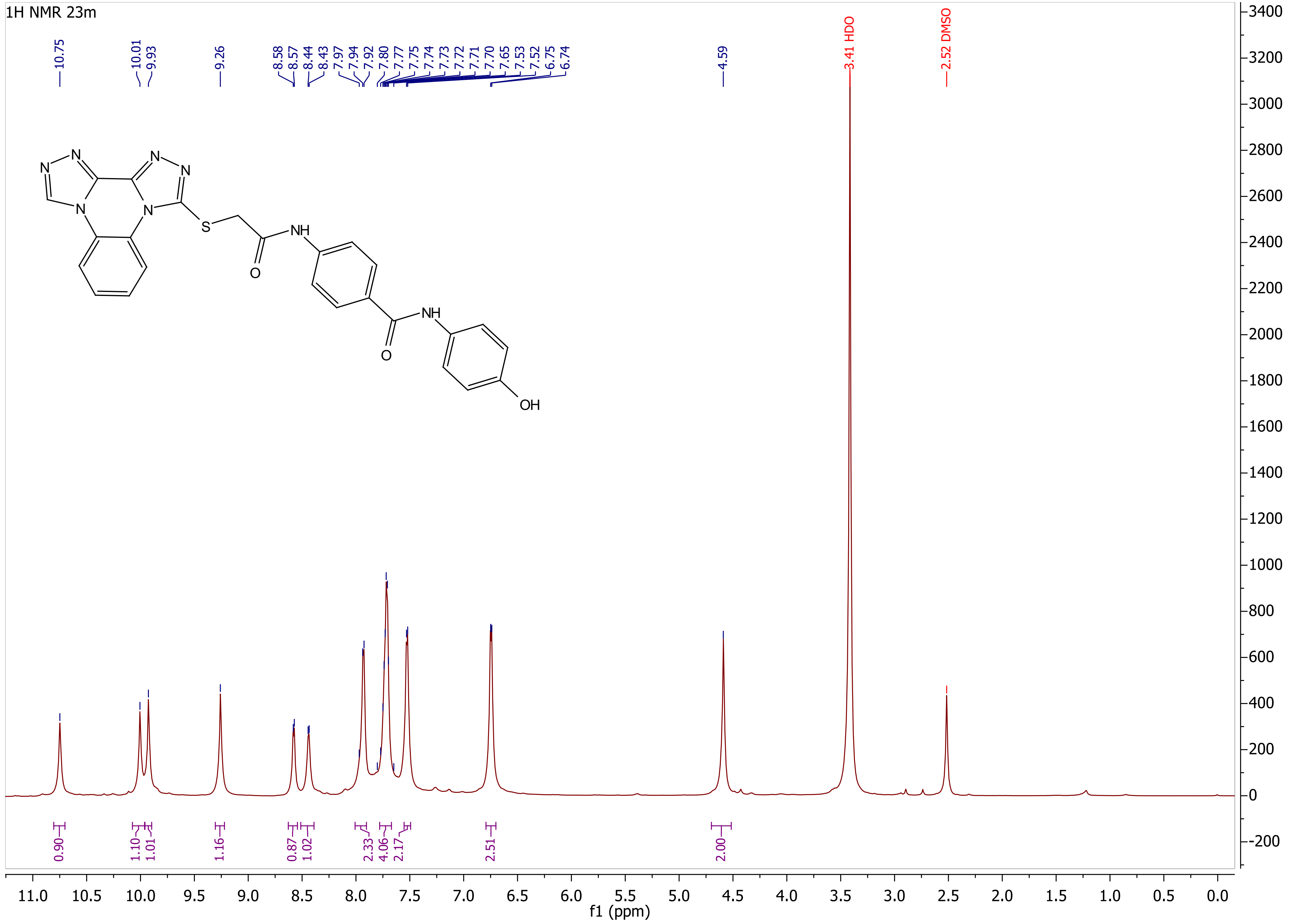
1H NMR 23m
10.75
10.01
9.93
9.26
8.58
8.57
8.44
8.43
7.97
7.94
7.92
7.80
7.77
7.75
7.74
7.73
7.72
7.71
7.70
7.65
7.53
7.52
6.75
6.74
4.59
3.41 HDO
2.52 DMSO
N
N
N
N
N
N
S
NH
O
NH
O
OH
0.90
1.10
1.01
1.16
0.87
1.02
2.33
4.06
2.17
2.51
2.00
f1 (ppm)
11.0
10.5
10.0
9.5
9.0
8.5
8.0
7.5
7.0
6.5
6.0
5.5
5.0
4.5
4.0
3.5
3.0
2.5
2.0
1.5
1.0
0.5
0.0
3400
3200
3000
2800
2600
2400
2200
2000
1800
1600
1400
1200
1000
800
600
400
200
0
-200

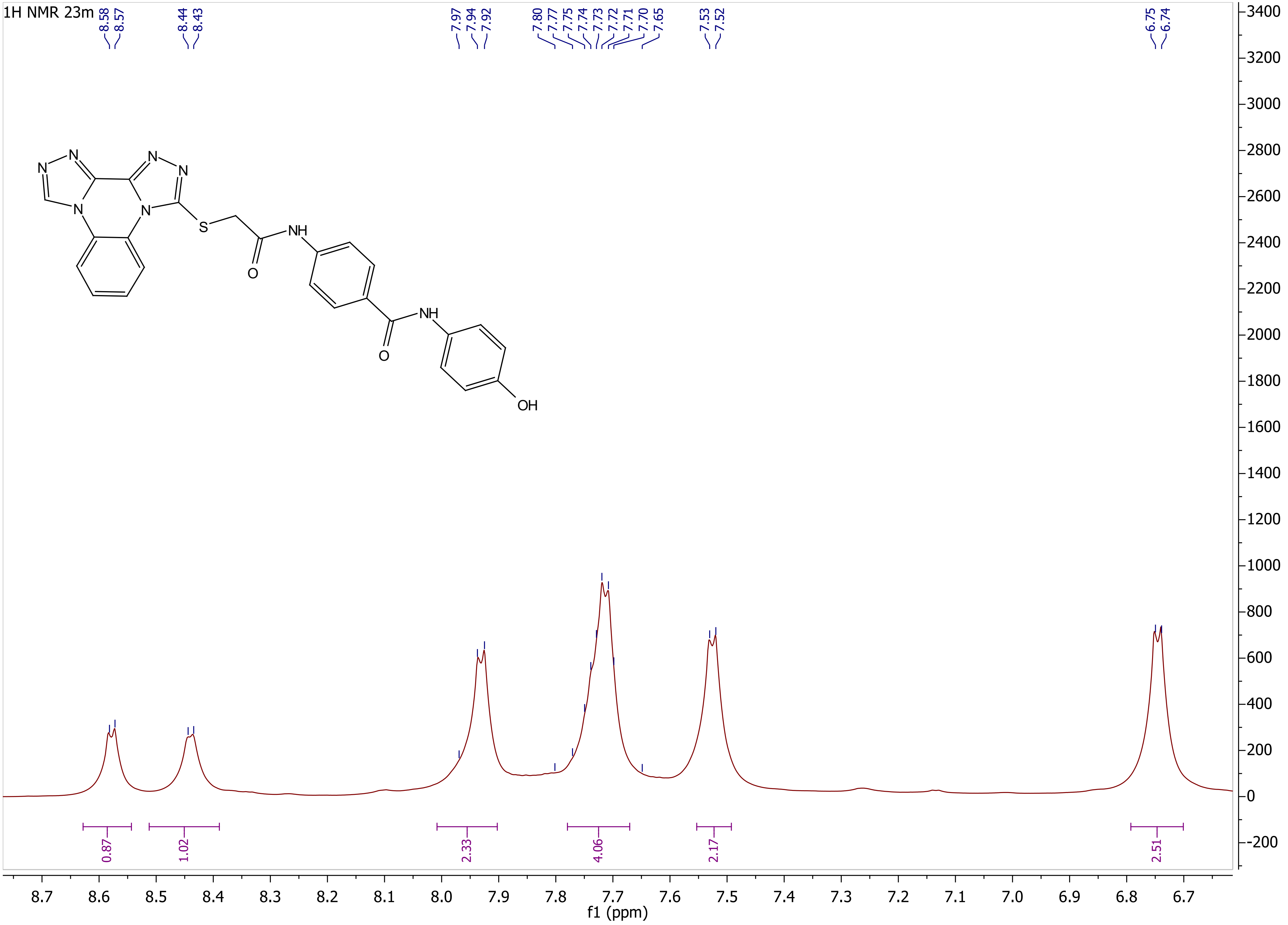

1H NMR 23m
8.58
8.57
8.44
8.43
7.97
7.94
7.92
7.80
7.77
7.75
7.74
7.73
7.72
7.71
7.70
7.65
7.53
7.52
6.75
6.74
N
N
N
N
N
N
S
NH
O
NH
O
OH
0.87
1.02
2.33
4.06
2.17
2.51
f1 (ppm)

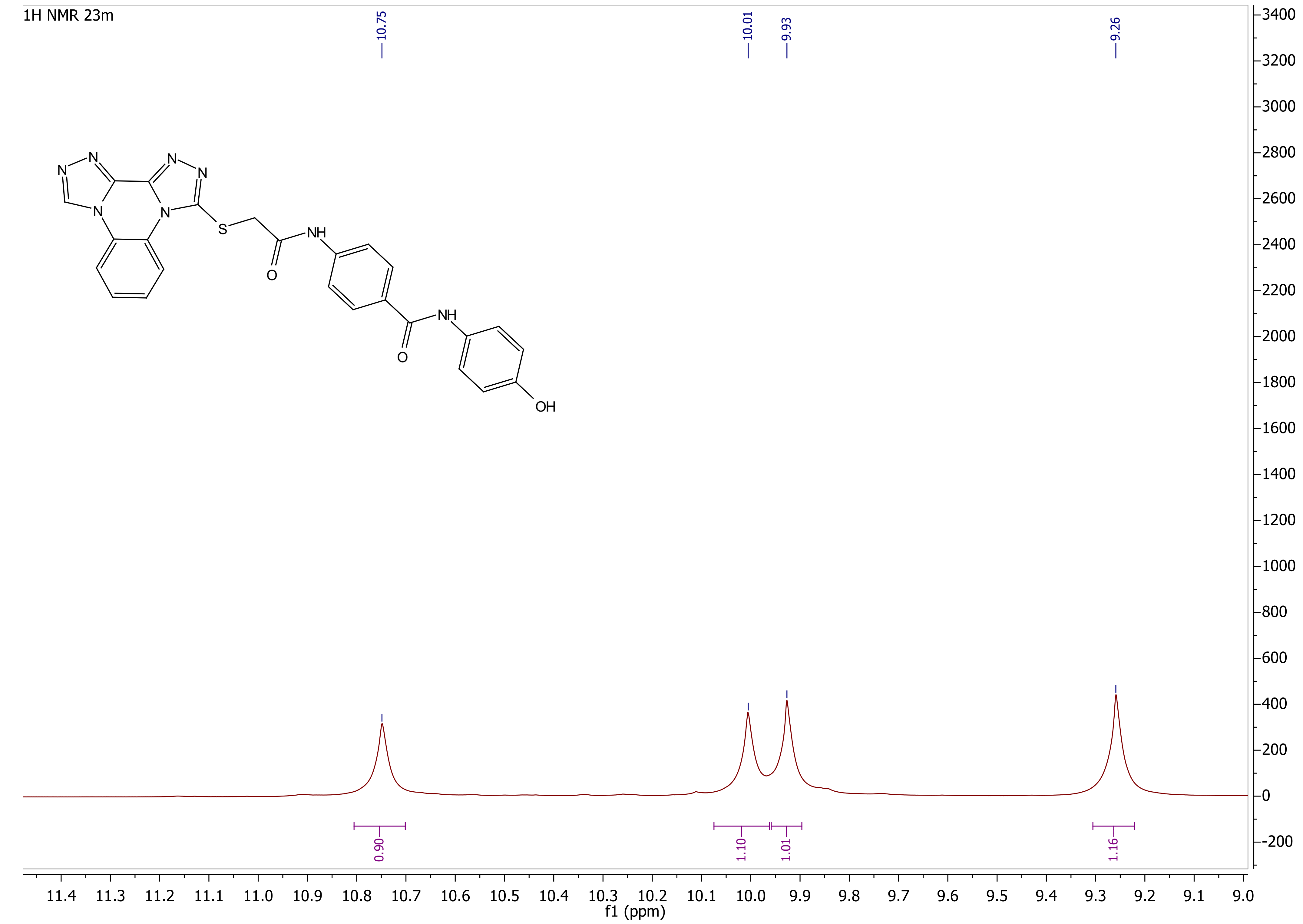
1H NMR 23m
10.75
10.01
9.93
9.26
0.90
1.10
1.01
1.16
f1 (ppm)
N
S
NH
O
OH

13C NMR 23m

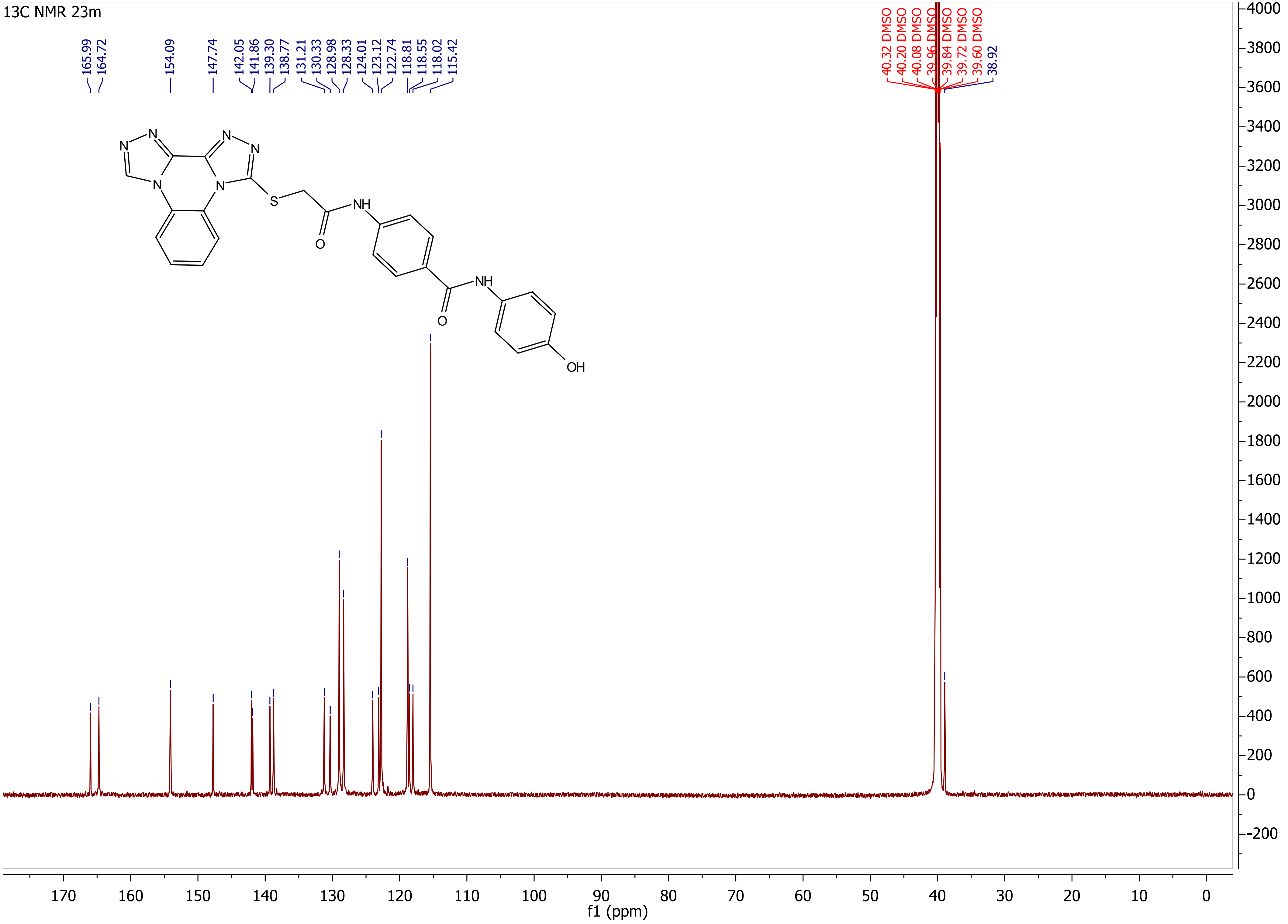

165.99
164.72
154.09
147.74
142.05
141.86
139.30
138.77
131.21
130.33
128.98
128.33
124.01
123.12
122.74
118.81
118.55
118.02
115.42
40.32 DMSO
40.20 DMSO
40.08 DMSO
39.96 DMSO
39.84 DMSO
39.72 DMSO
39.60 DMSO
38.92
N
N
N
N
N
N
S
O
NH
NH
O
OH
f1 (ppm)

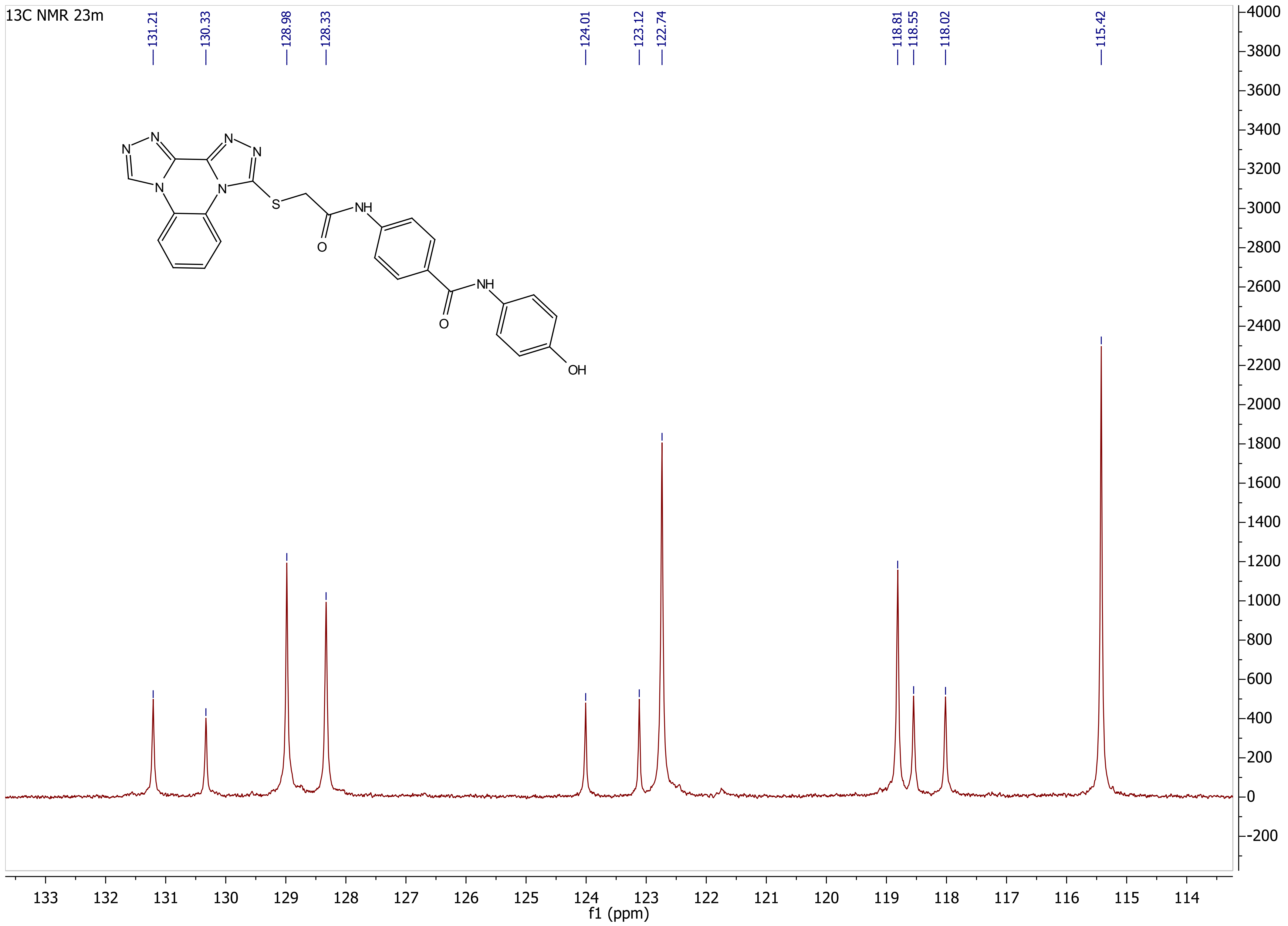

13C NMR 23m
131.21
130.33
128.98
128.33
124.01
123.12
122.74
118.81
118.55
118.02
115.42
f1 (ppm)

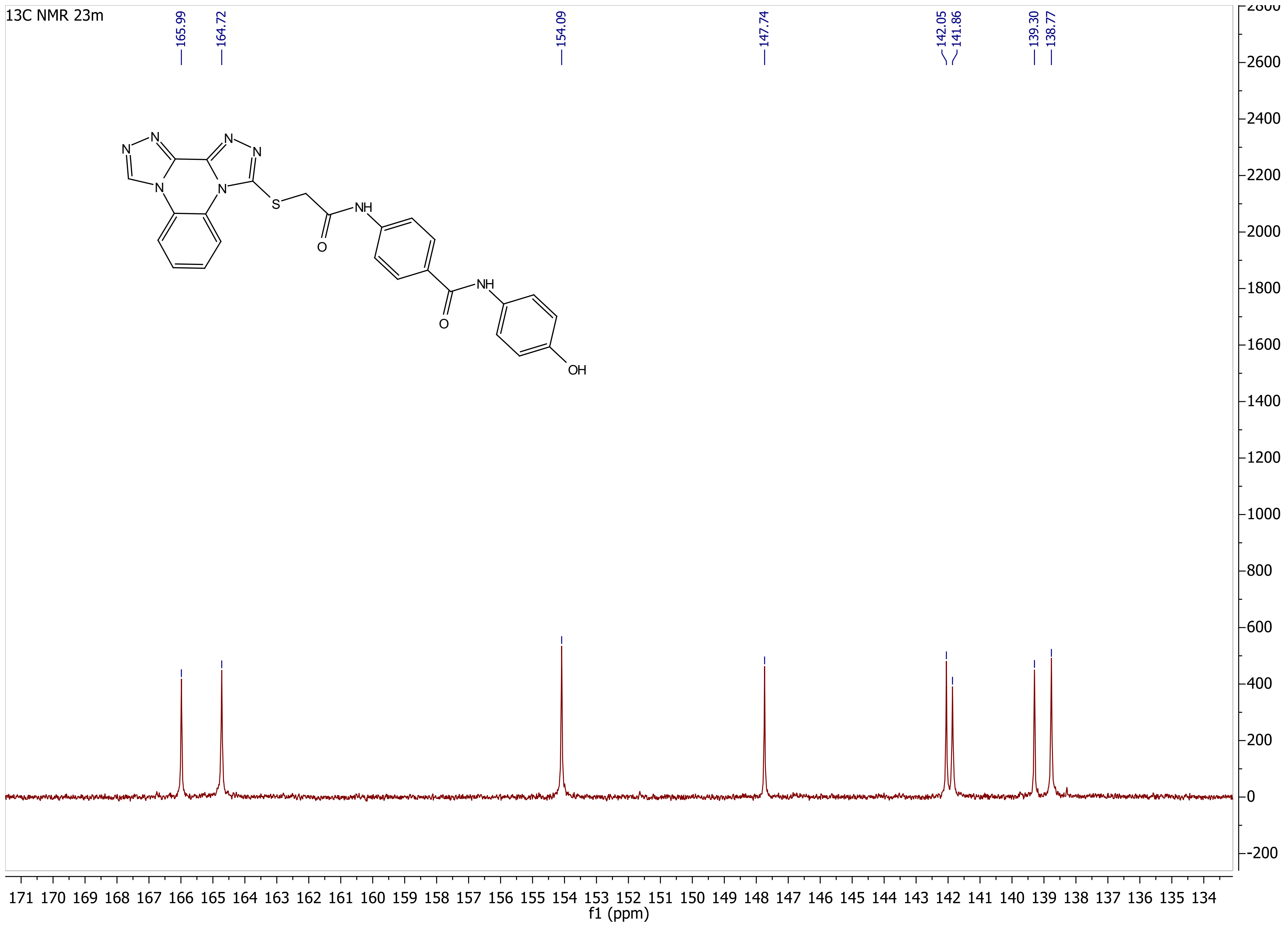
13C NMR 23m
165.99
164.72
154.09
147.74
142.05
141.86
139.30
138.77
N
N
N
N
N
N
S
NH
O
NH
O
OH
f1 (ppm)

Mass spec. of 23m

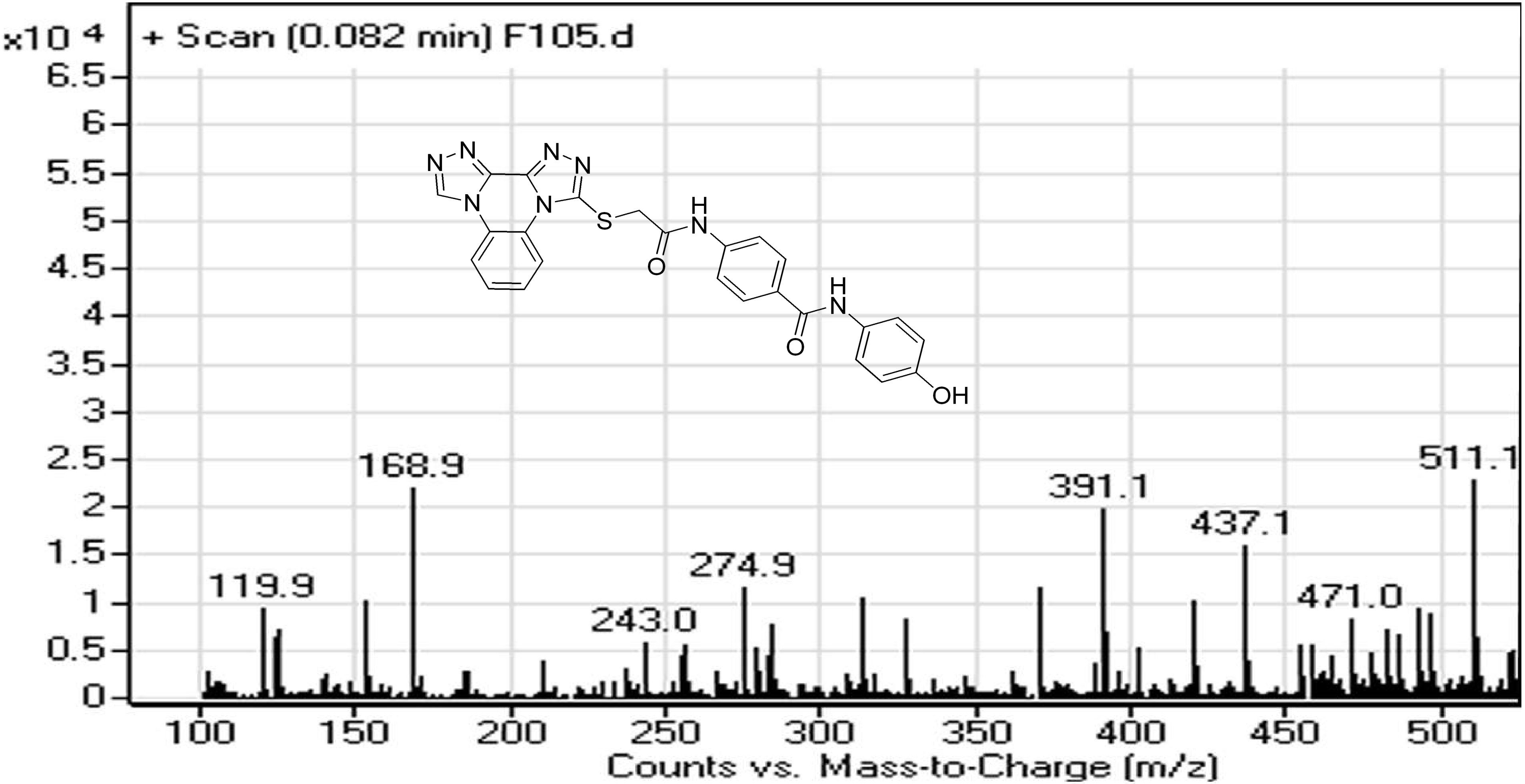

IR of compound 23n

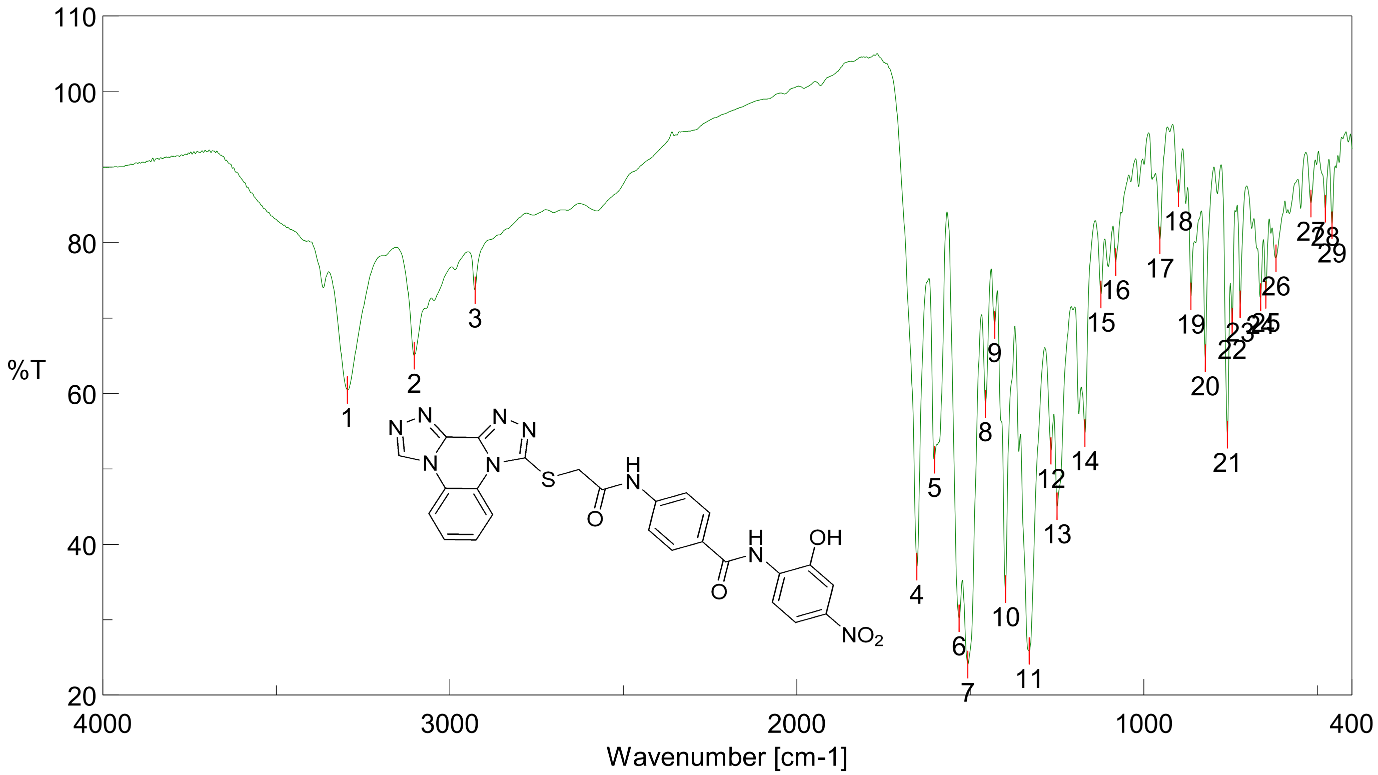

[Comments]

Sample name	F97
Comment	
User	
Division	
Company	KSU

[Detailed Information]

Creation date	10/29/2020 5:14 AM
Data array type	Linear data array
Horizontal axis	Wavenumber [cm-1]
Vertical axis	%T
Start	399.193 cm-1
End	4000.6 cm-1
Data interval	0.964233 cm-1
Data points	3736

[Measurement Information]

Model Name	FT/IR-6600typeA
Serial Number	A014661790
Measurement Date	10/28/2020 4:35 AM
Light Source	Standard
Detector	TGS
Accumulation	Auto (20)
Resolution	4 cm-1
Zero Filling	On
Apodization	Cosine
Gain	Auto (2)
Aperture	Auto (7.1 mm)
Scanning Speed	Auto (2 mm/sec)
Filter	Auto (10000 Hz)

[Result of Peak Picking]

No.	Position	Intensity	No.	Position	Intensity	No.	Position	Intensity
1	3294.79	60.4393	2	3101.94	64.9686	3	2927.41	73.6389

[Result of Peak Picking]

No.	Position	Intensity	No.	Position	Intensity	No.	Position	Intensity
4	1653.66	37.021	5	1603.52	51.1679	6	1532.17	30.1983
7	1507.1	24.0502	8	1455.99	58.587	9	1429.96	69.0429
10	1398.14	34.0374	11	1329.68	25.8703	12	1267	52.3844
13	1249.65	45.0487	14	1169.62	54.7201	15	1123.33	73.0879
16	1080.91	77.3667	17	953.627	80.2521	18	899.63	86.5059
19	863.953	72.8117	20	822.491	64.6224	21	758.852	54.4863
22	745.352	69.4865	23	722.211	71.7488	24	663.393	72.7445
25	647.965	73.0265	26	619.038	77.8935	27	517.793	85.1204
28	476.331	84.4487	29	457.047	82.2464			

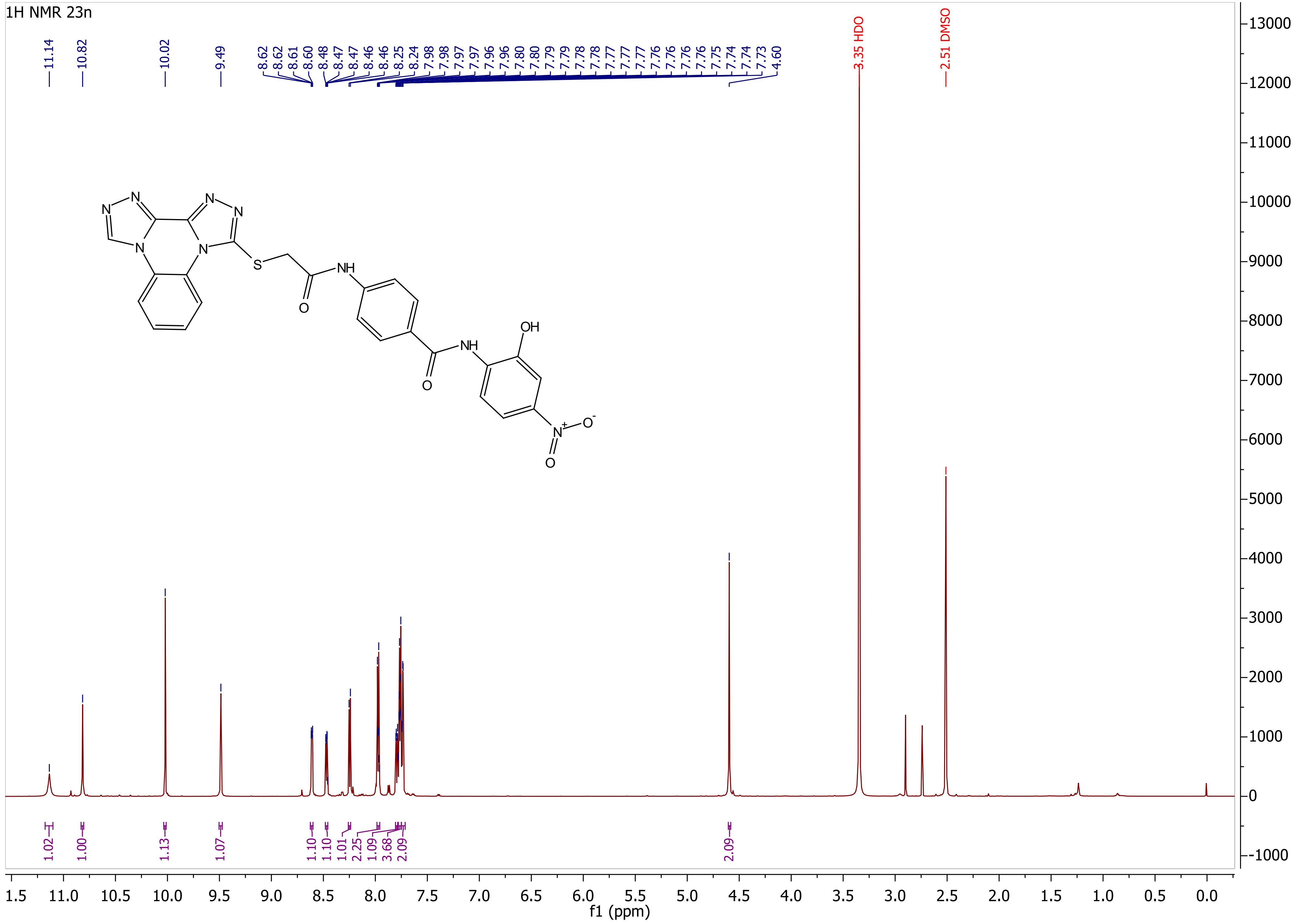
1H NMR 23n
11.14
10.82
10.02
9.49
8.62 8.62 8.61 8.60 8.48 8.47 8.47 8.46 8.46 8.25 8.24 7.98 7.98 7.97 7.97 7.96 7.96 7.80 7.80 7.79 7.79 7.78 7.78 7.77 7.77 7.77 7.76 7.76 7.76 7.76 7.75 7.74 7.74 7.73
4.60
3.35 HDO
2.51 DMSO
1.02 1.00 1.13 1.07 1.10 1.10 1.01 2.25 1.09 3.68 2.09 2.09
f1 (ppm)

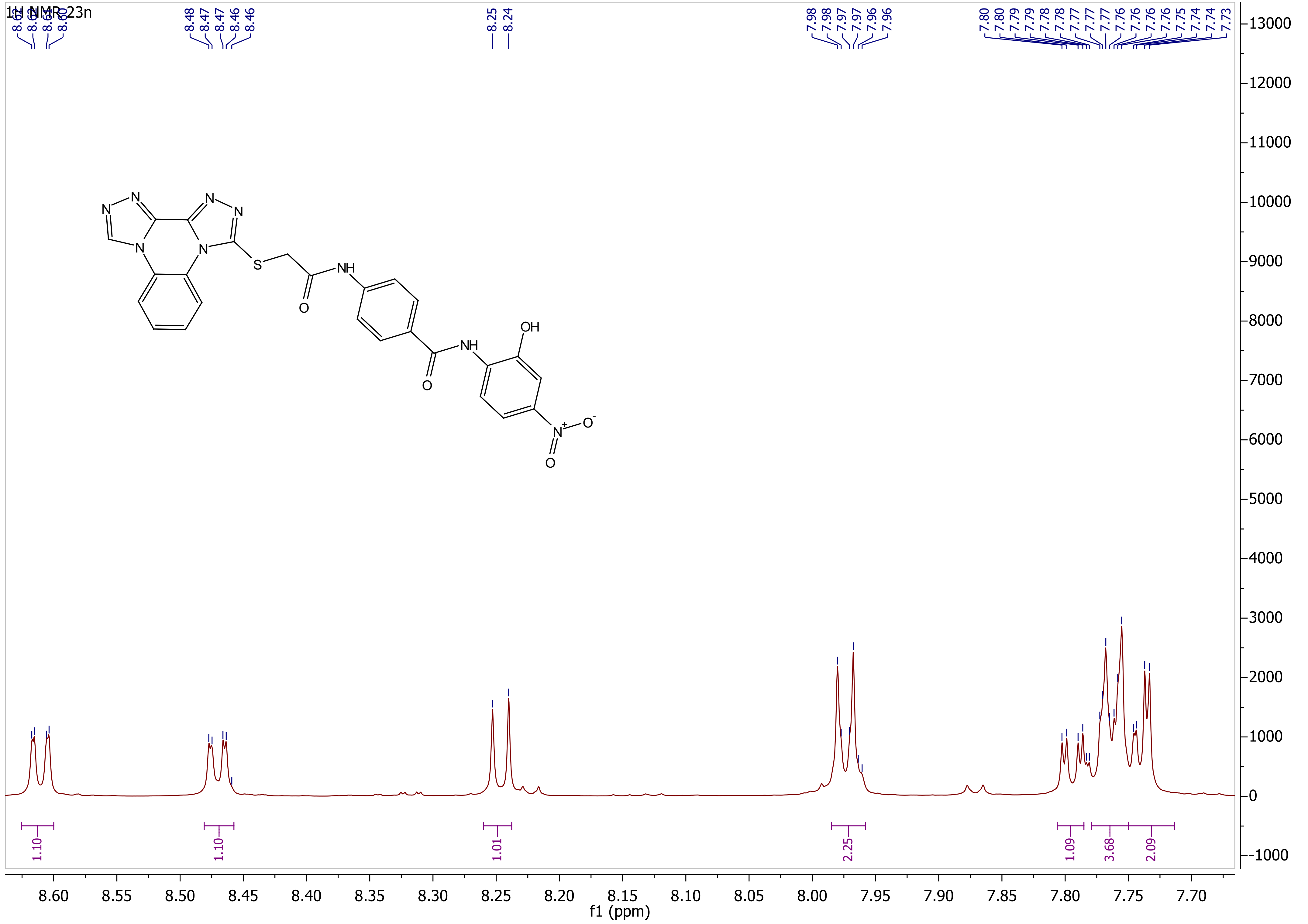
1H NMR 23n
8.48
8.47
8.47
8.46
8.46
8.25
8.24
7.98
7.98
7.97
7.97
7.96
7.96
7.80
7.80
7.79
7.79
7.78
7.78
7.77
7.77
7.77
7.76
7.76
7.76
7.76
7.75
7.74
7.74
7.73
1.10
1.10
1.01
2.25
1.09
3.68
2.09
f1 (ppm)

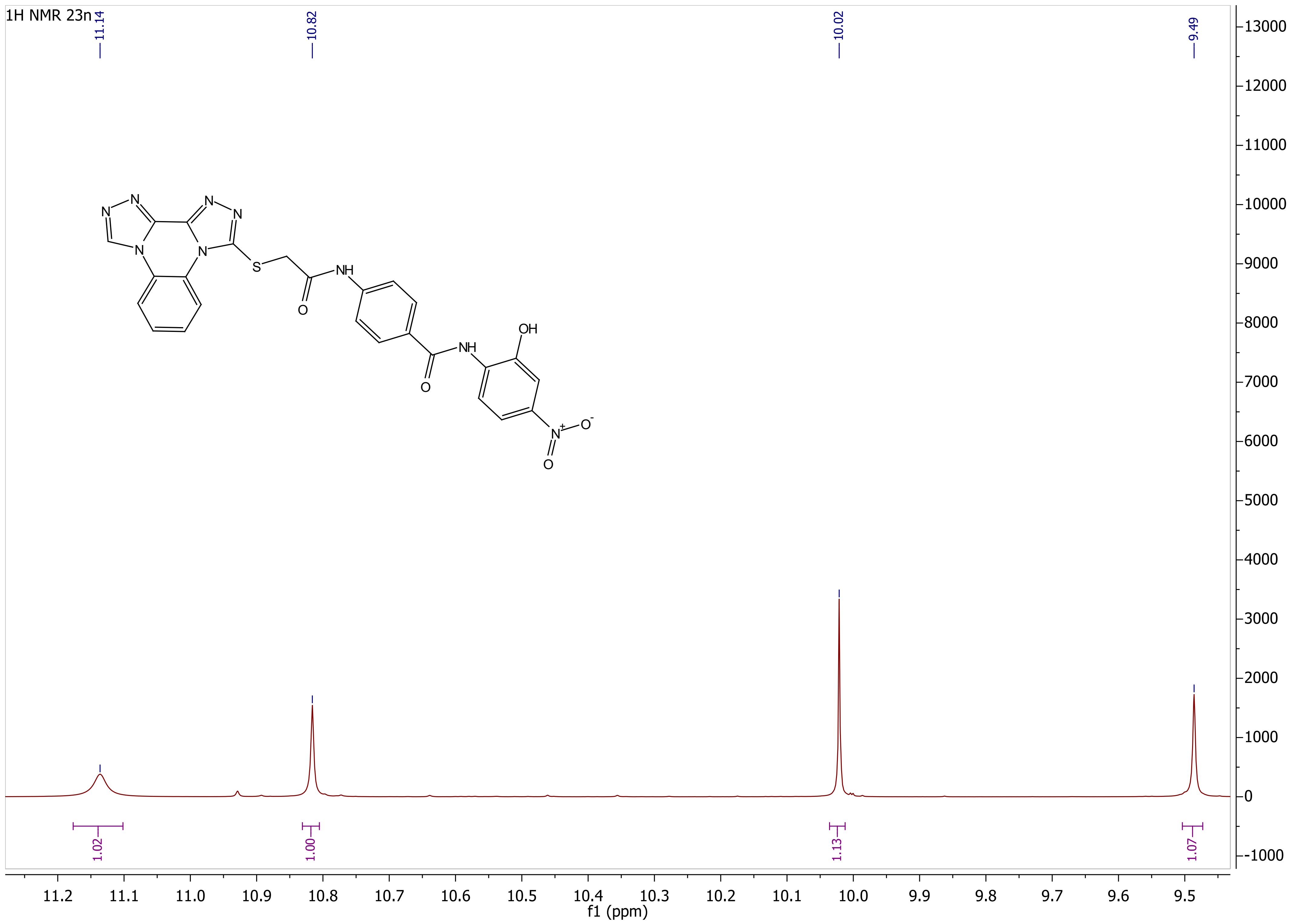
1H NMR 23n
11.14
10.82
10.02
9.49
1.02
1.00
1.13
1.07
f1 (ppm)

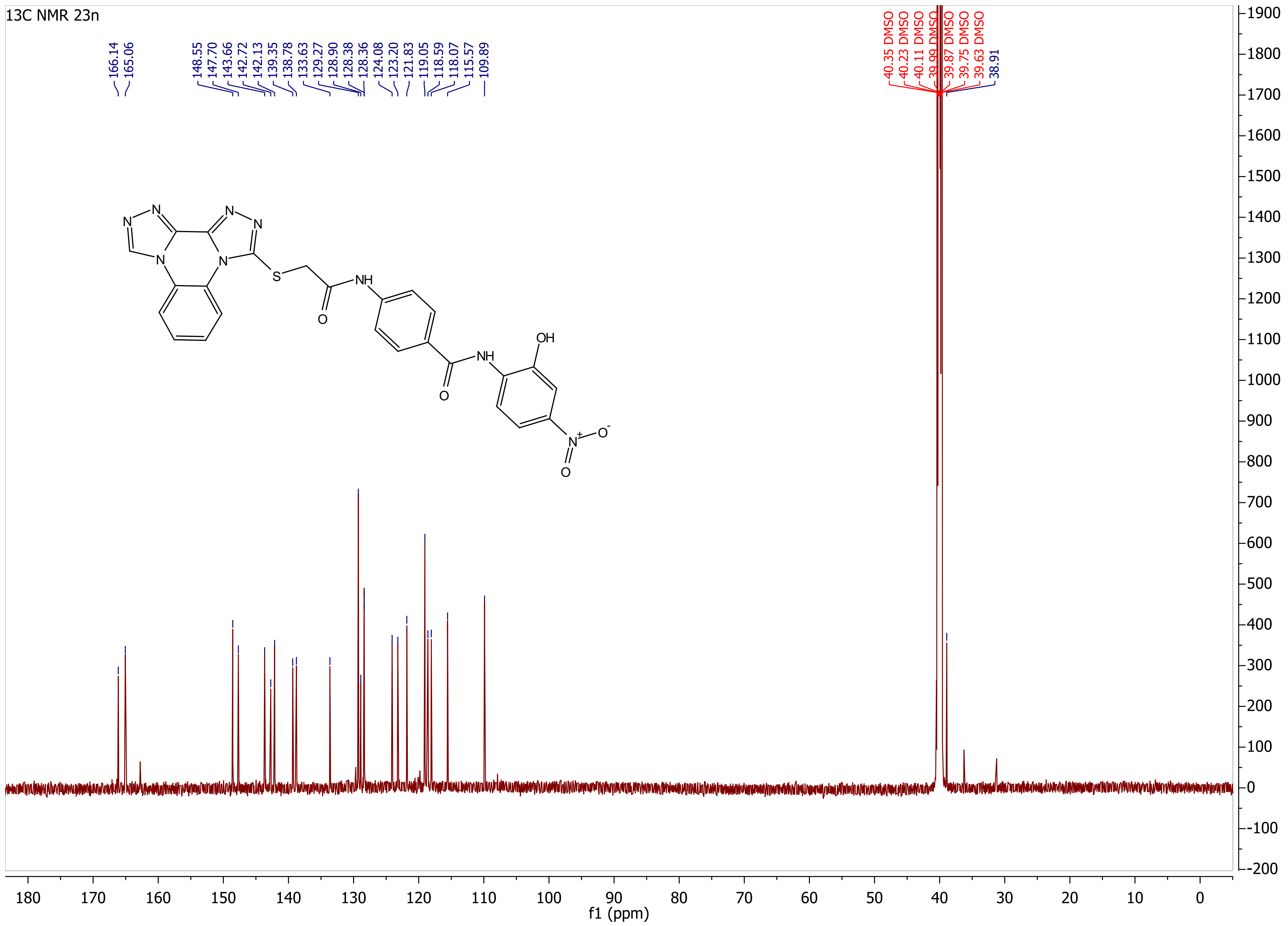
13C NMR 23n
166.14
165.06
148.55
147.70
143.66
142.72
142.13
139.35
138.78
133.63
129.27
128.90
128.38
128.36
124.08
123.20
121.83
119.05
118.59
118.07
115.57
109.89
40.35 DMSO
40.23 DMSO
40.11 DMSO
39.99 DMSO
39.87 DMSO
39.75 DMSO
39.63 DMSO
38.91
f1 (ppm)

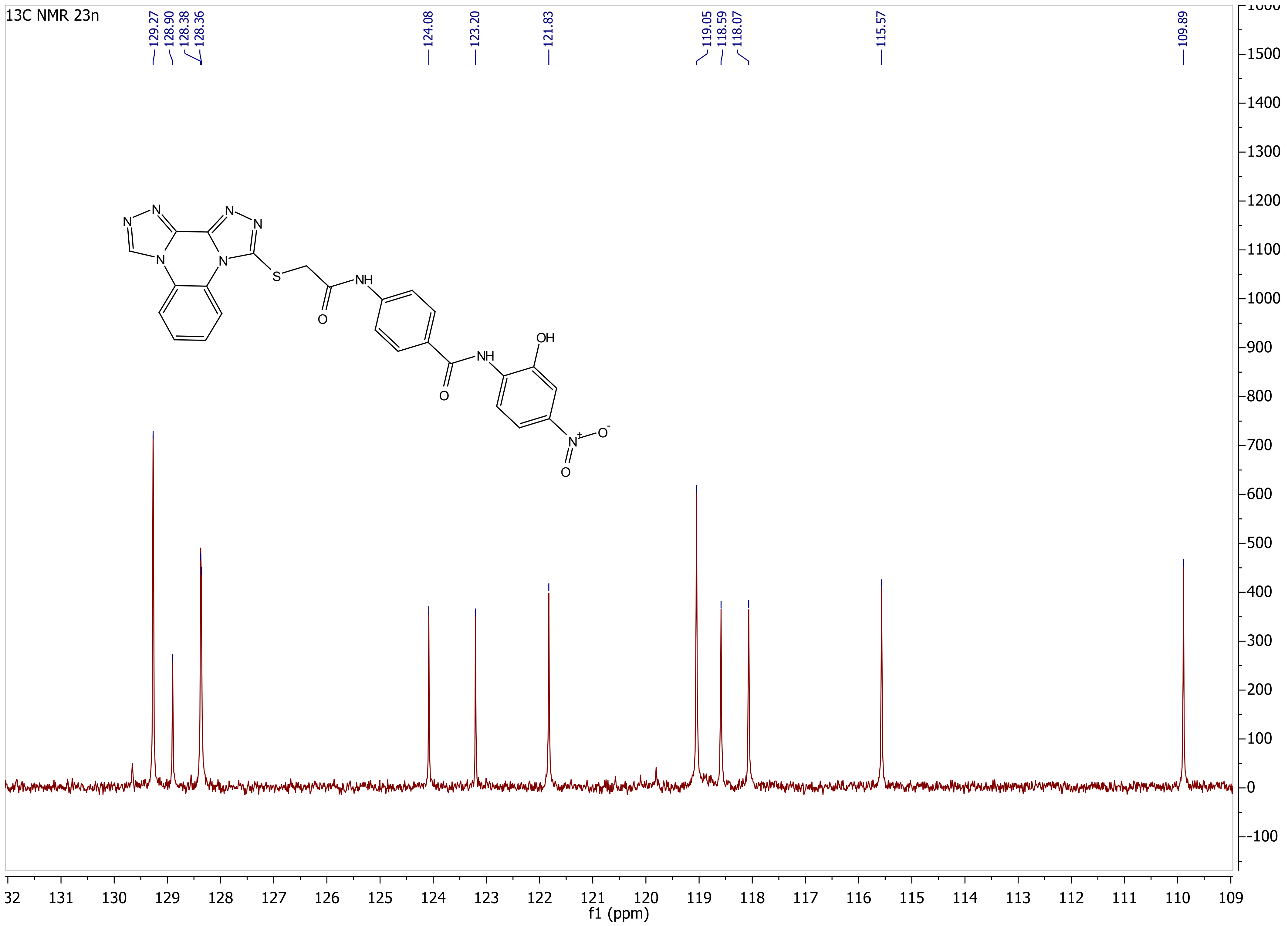
13C NMR 23n
129.27
128.90
128.38
128.36
124.08
123.20
121.83
119.05
118.59
118.07
115.57
109.89
f1 (ppm)

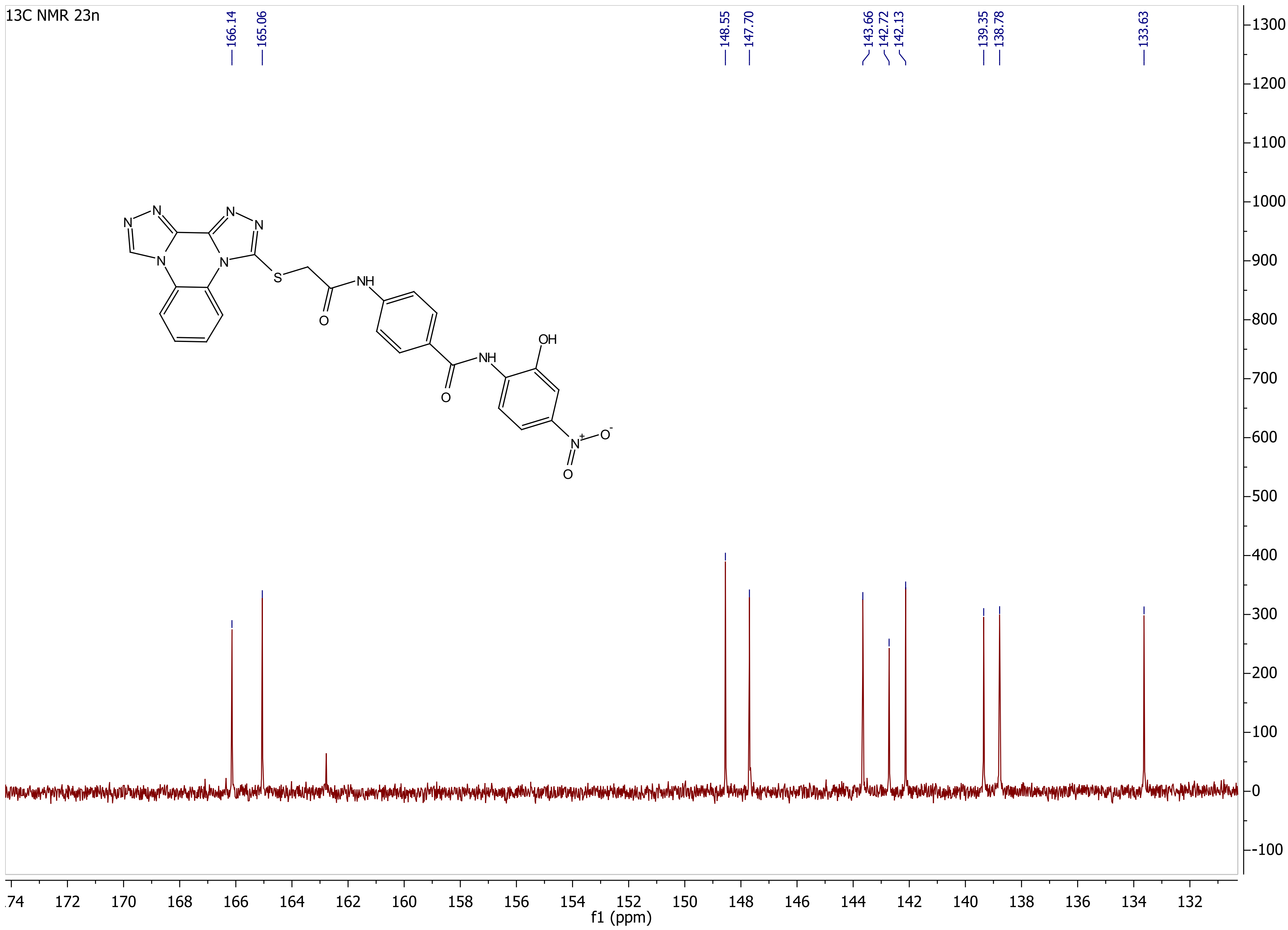
13C NMR 23n
166.14
165.06
148.55
147.70
143.66
142.72
142.13
139.35
138.78
133.63
f1 (ppm)

Mass spec. of 23n

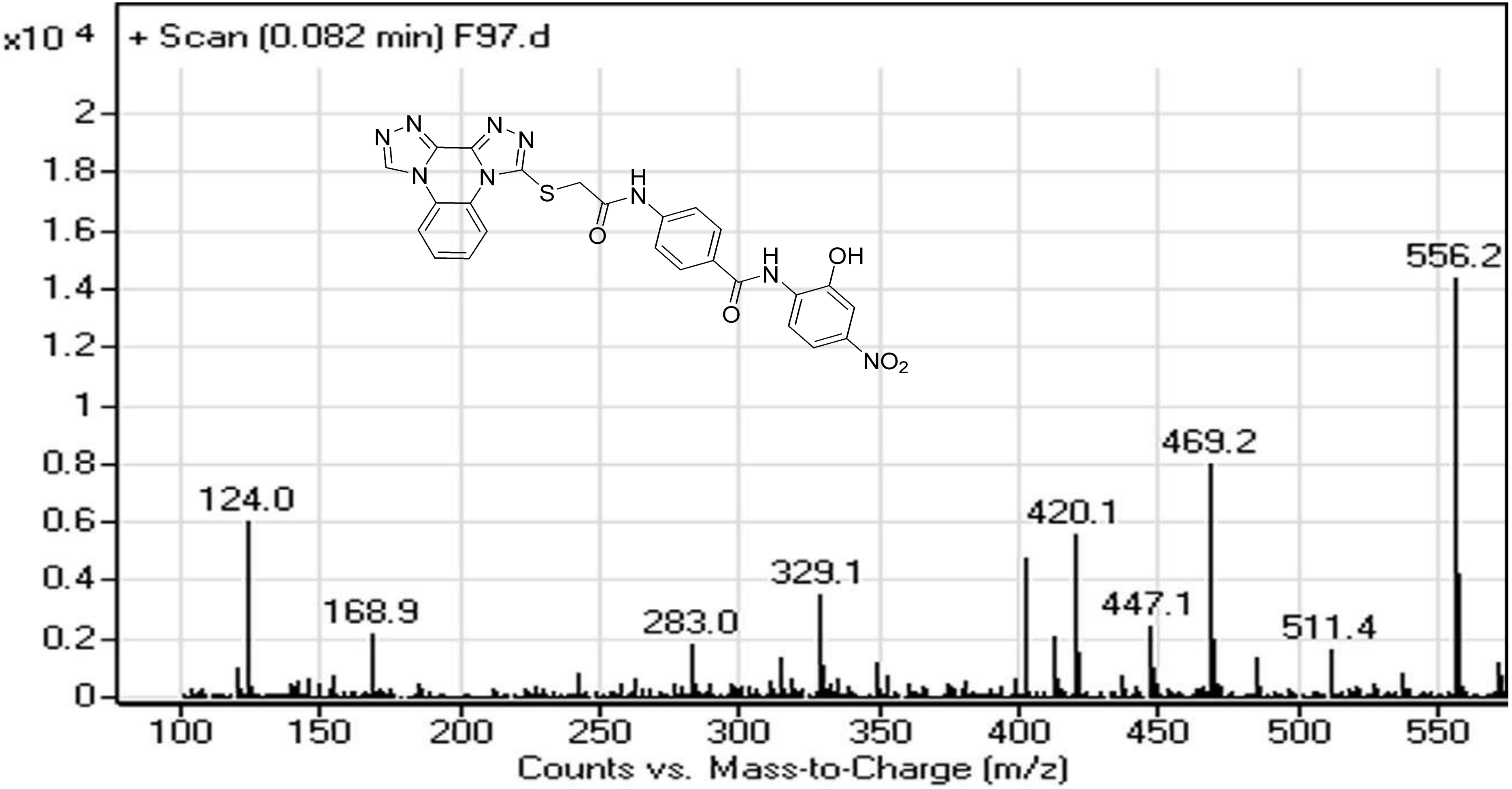

IR of compound 24a

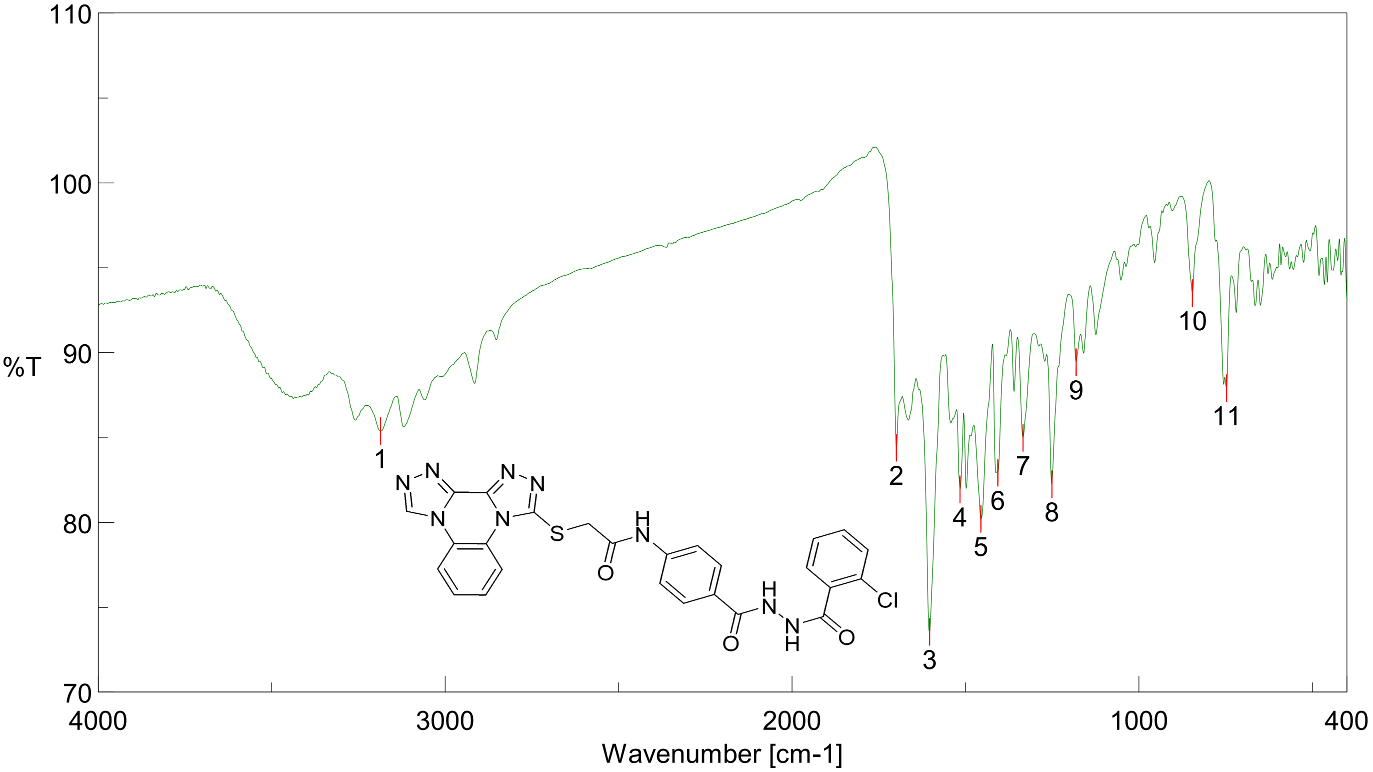

[Comments]

Sample name	F110
Comment	
User	
Division	
Company	KSU

[Detailed Information]

Creation date	11/1/2020 12:26 AM
Data array type	Linear data array
Horizontal axis	Wavenumber [cm-1]
Vertical axis	%T
Start	399.193 cm-1
End	4000.6 cm-1
Data interval	0.964233 cm-1
Data points	3736

[Measurement Information]

Model Name	FT/IR-6600typeA
Serial Number	A014661790
Measurement Date	10/28/2020 5:42 AM
Light Source	Standard
Detector	TGS
Accumulation	Auto (14)
Resolution	4 cm-1
Zero Filling	On
Apodization	Cosine
Gain	Auto (1)
Aperture	Auto (7.1 mm)
Scanning Speed	Auto (2 mm/sec)
Filter	Auto (10000 Hz)

[Result of Peak Picking]

No.	Position	Intensity	No.	Position	Intensity	No.	Position	Intensity
1	3185.83	85.3732	2	1698.98	84.3971	3	1603.52	73.5449

[Result of Peak Picking]

No.	Position	Intensity	No.	Position	Intensity	No.	Position	Intensity
4	1515.78	81.9355	5	1455.03	80.1959	6	1406.82	82.9165
7	1333.53	84.9751	8	1250.61	82.242	9	1180.22	89.4249
10	845.633	93.4947	11	747.281	87.8925			

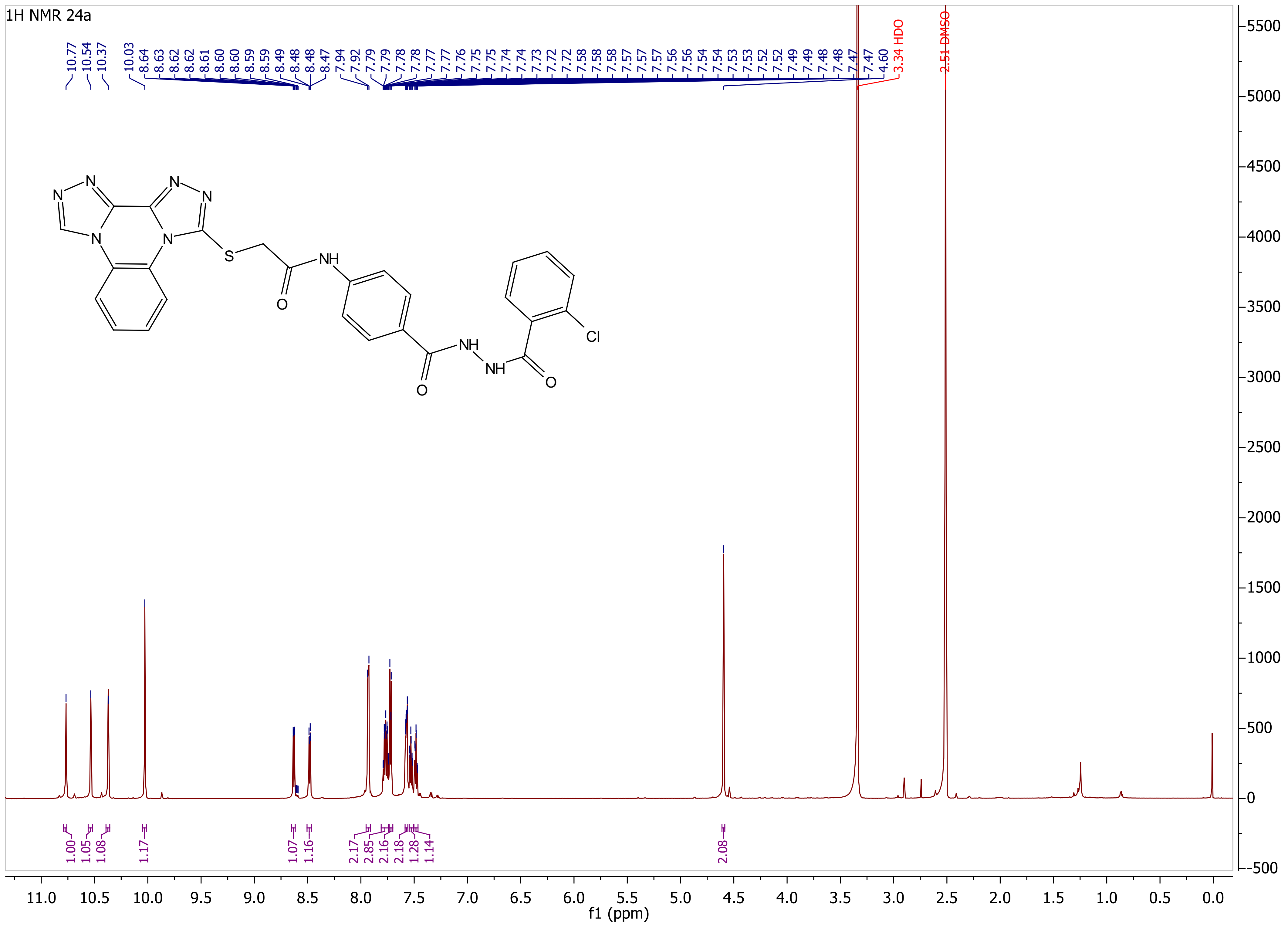
1H NMR 24a
10.77
10.54
10.37
10.03
8.64
8.63
8.62
8.62
8.61
8.60
8.60
8.59
8.59
8.49
8.48
8.48
8.47
7.94
7.92
7.79
7.79
7.78
7.78
7.77
7.77
7.76
7.75
7.75
7.74
7.74
7.73
7.72
7.72
7.58
7.58
7.58
7.57
7.57
7.57
7.56
7.56
7.54
7.54
7.53
7.53
7.52
7.52
7.49
7.49
7.48
7.48
7.47
7.47
4.60
3.34 HDO
2.51 DMSO
1.00
1.05
1.08
1.17
1.07
1.16
2.17
2.85
2.16
2.18
1.28
1.14
2.08
f1 (ppm)

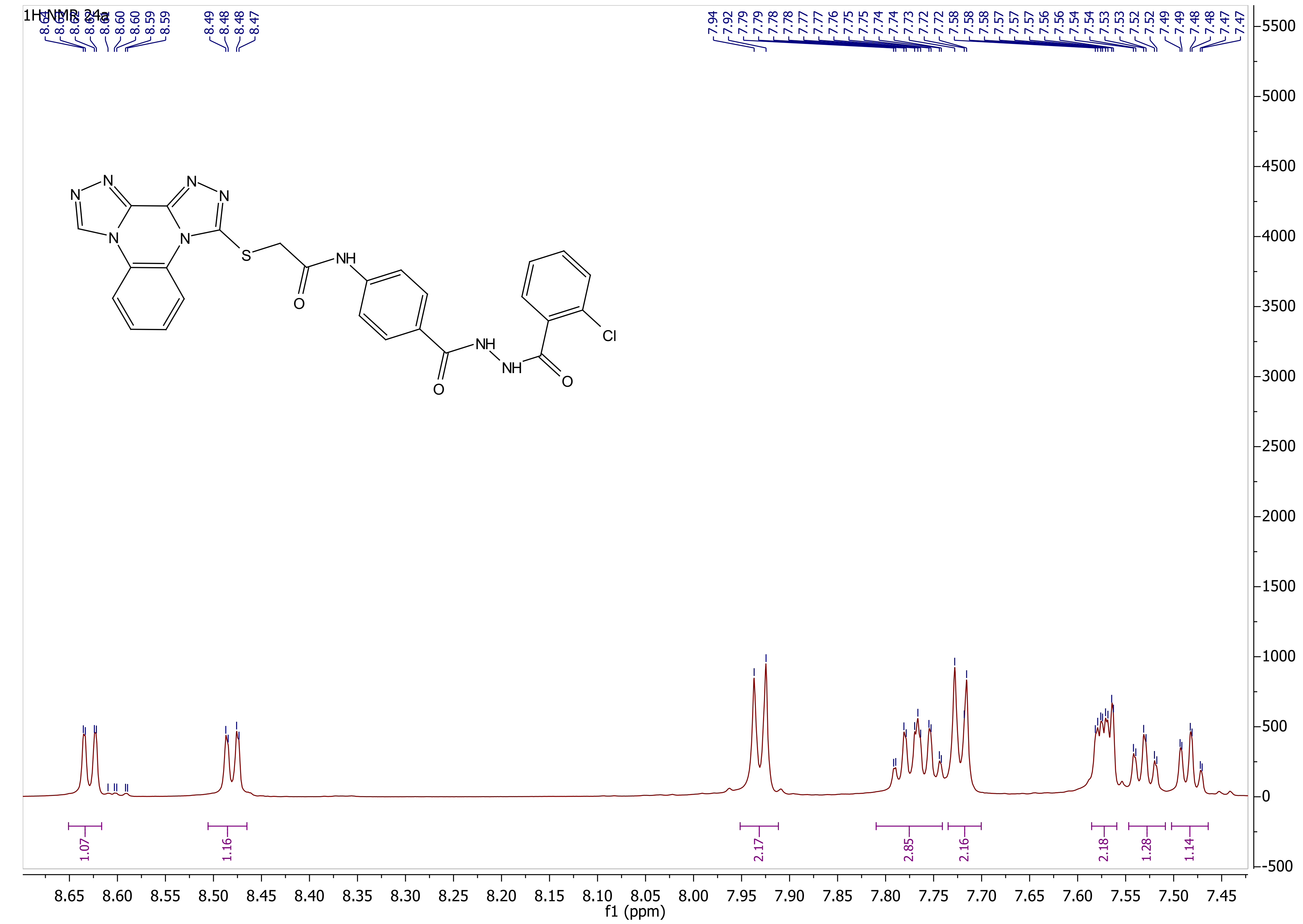

1H NMR 24a
8.60
8.60
8.59
8.59
8.49
8.48
8.48
8.47
7.94
7.92
7.79
7.79
7.78
7.78
7.77
7.77
7.76
7.75
7.75
7.74
7.74
7.73
7.72
7.72
7.58
7.58
7.58
7.57
7.57
7.57
7.56
7.56
7.54
7.54
7.53
7.53
7.52
7.52
7.49
7.49
7.48
7.48
7.47
7.47
N
N
N
N
N
N
S
O
NH
NH
NH
O
O
Cl
1.07
1.16
2.17
2.85
2.16
2.18
1.28
1.14
f1 (ppm)

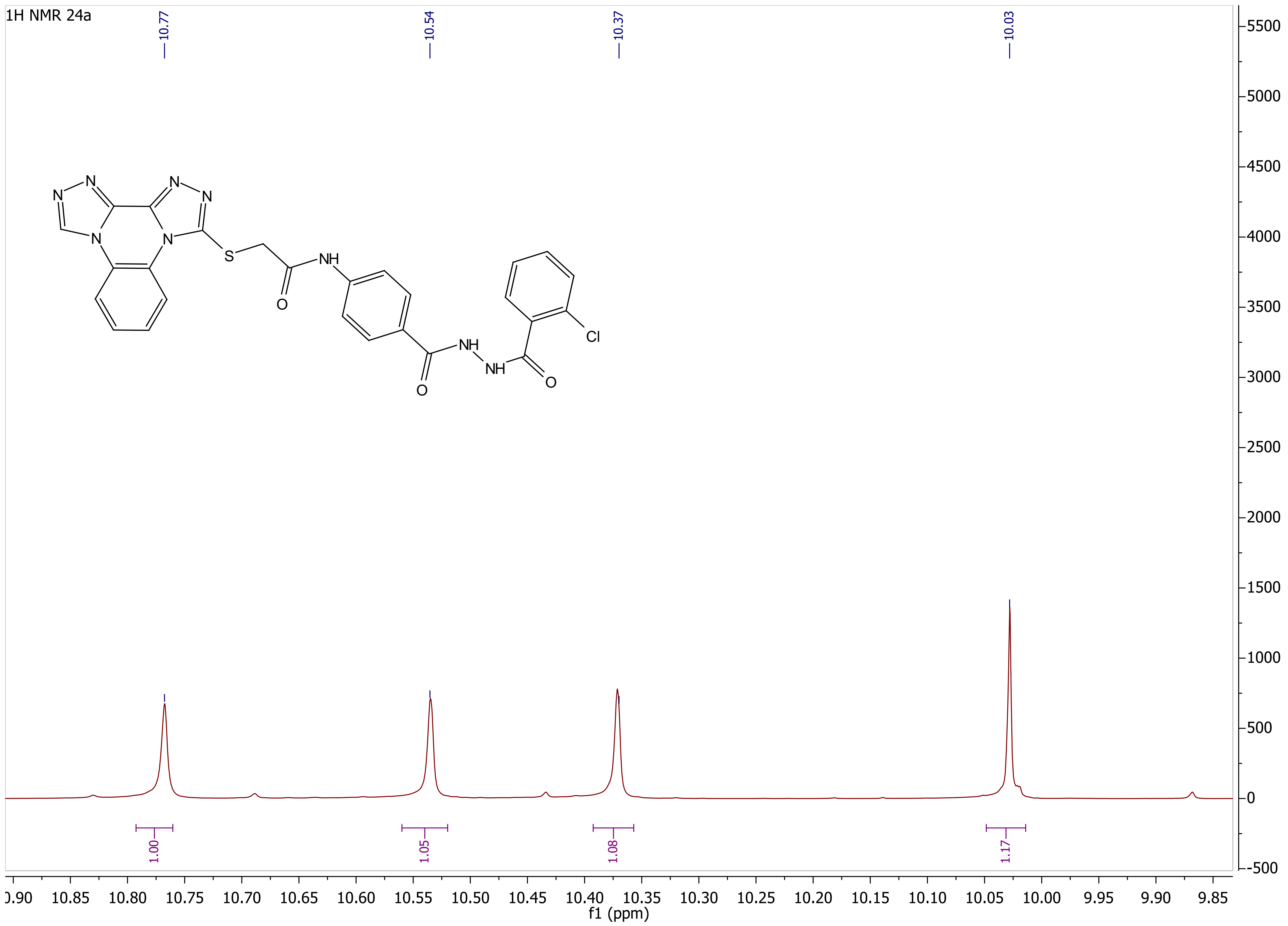
1H NMR 24a
10.77
10.54
10.37
10.03
1.00
1.05
1.08
1.17
f1 (ppm)

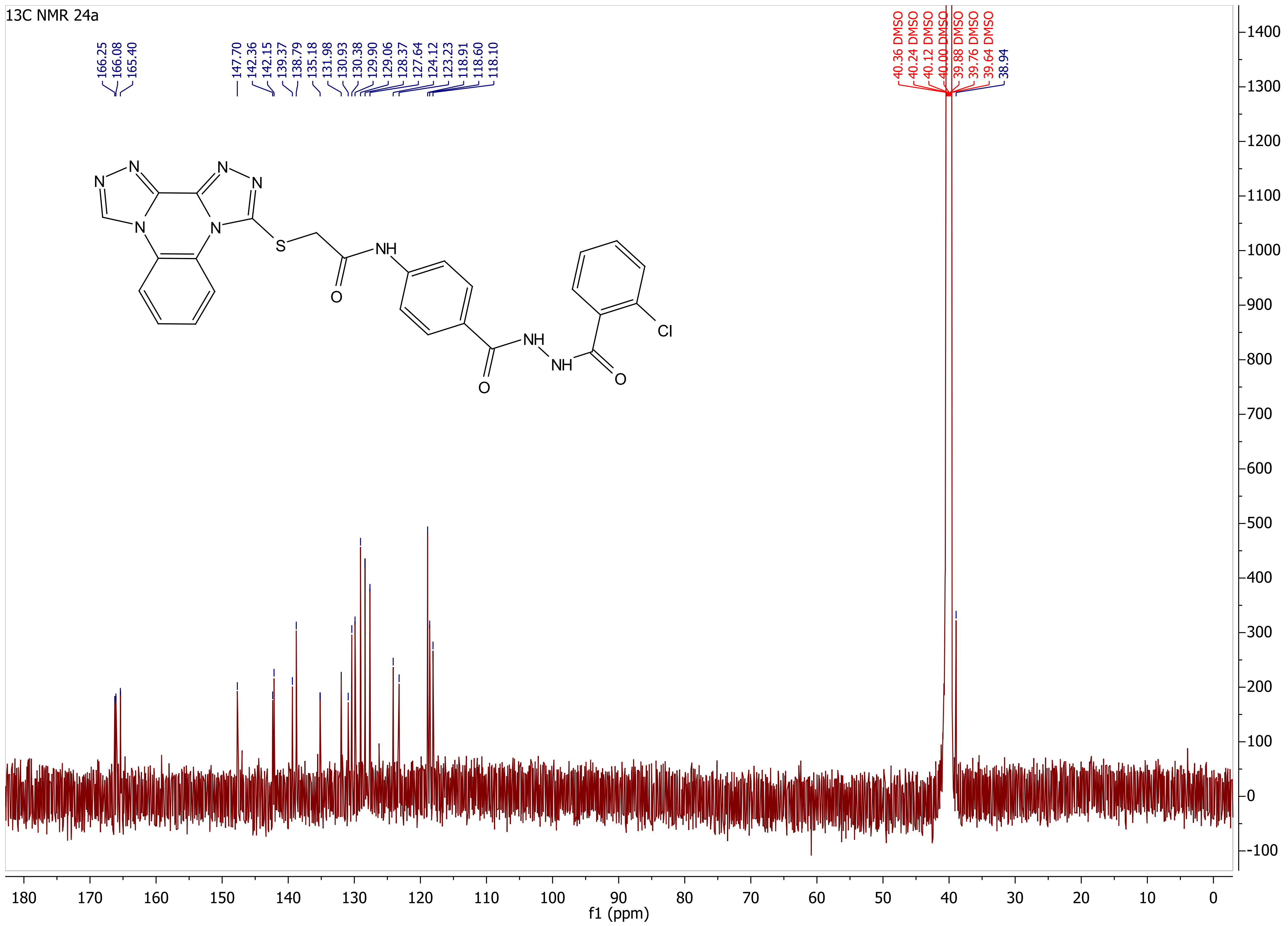
13C NMR 24a
166.25
166.08
165.40
147.70
142.36
142.15
139.37
138.79
135.18
131.98
130.93
130.38
129.90
129.06
128.37
127.64
124.12
123.23
118.91
118.60
118.10
40.36 DMSO
40.24 DMSO
40.12 DMSO
40.00 DMSO
39.88 DMSO
39.76 DMSO
39.64 DMSO
38.94
f1 (ppm)

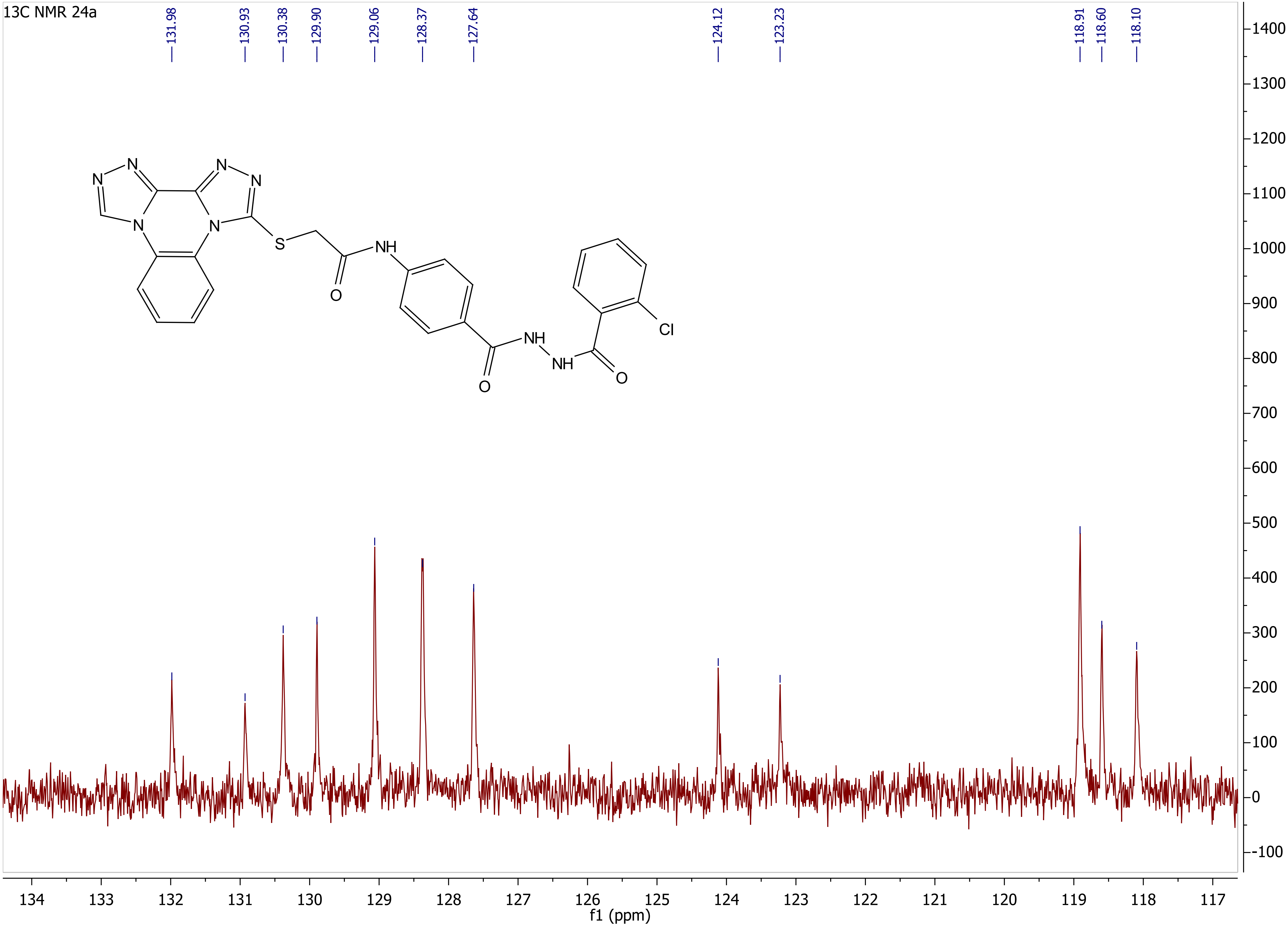
13C NMR 24a
131.98
130.93
130.38
129.90
129.06
128.37
127.64
124.12
123.23
118.91
118.60
118.10
N
N
N
N
N
N
N
N
S
NH
O
NH
NH
O
O
Cl
1400
1300
1200
1100
1000
900
800
700
600
500
400
300
200
100
0
-100
134
133
132
131
130
129
128
127
126
125
124
123
122
121
120
119
118
117
f1 (ppm)

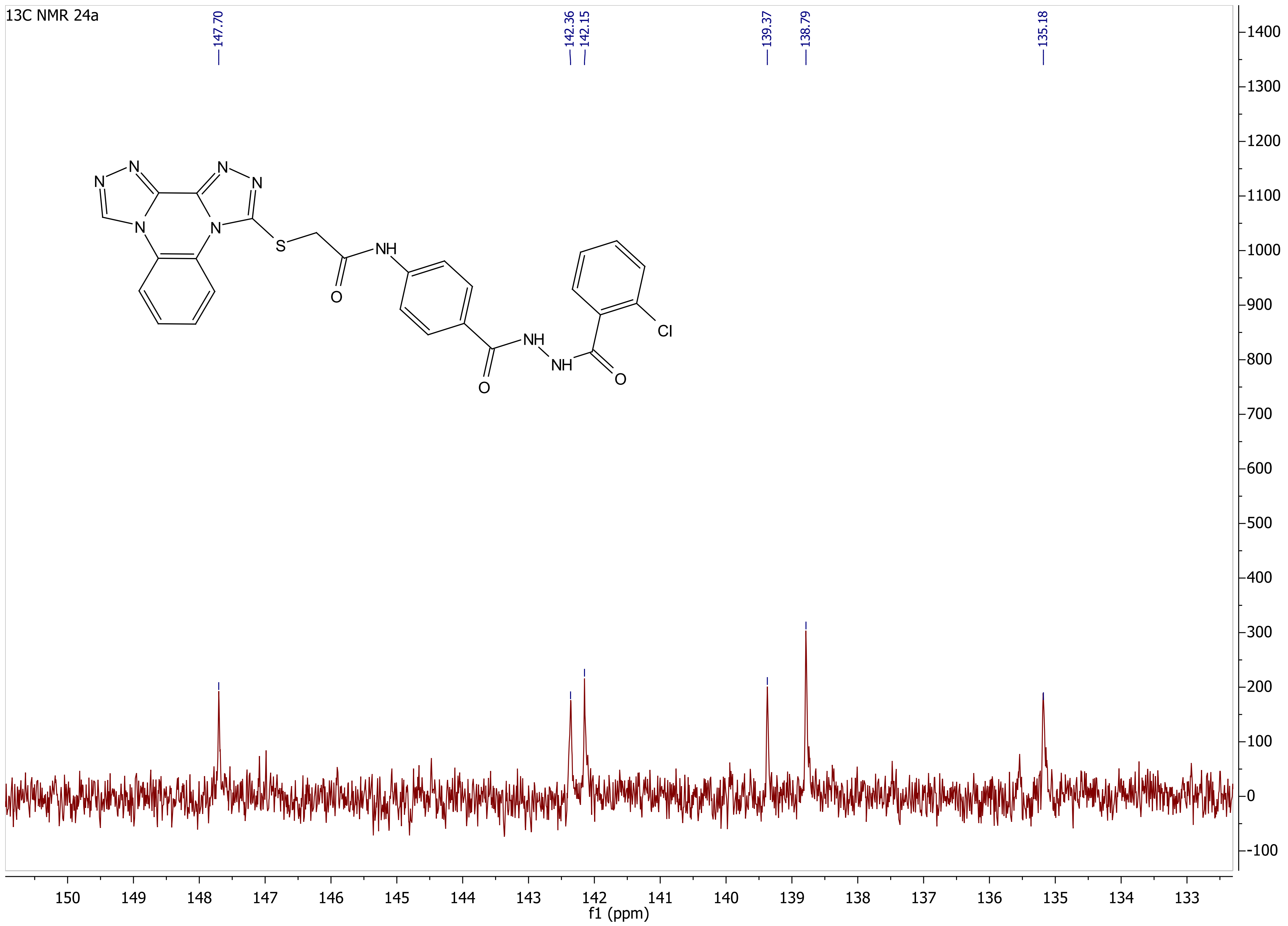

13C NMR 24a
147.70
142.36
142.15
139.37
138.79
135.18
N
N
N
N
N
N
N
N
S
NH
O
NH
NH
O
O
Cl
f1 (ppm)
150
149
148
147
146
145
144
143
142
141
140
139
138
137
136
135
134
133
1400
1300
1200
1100
1000
900
800
700
600
500
400
300
200
100
0
-100

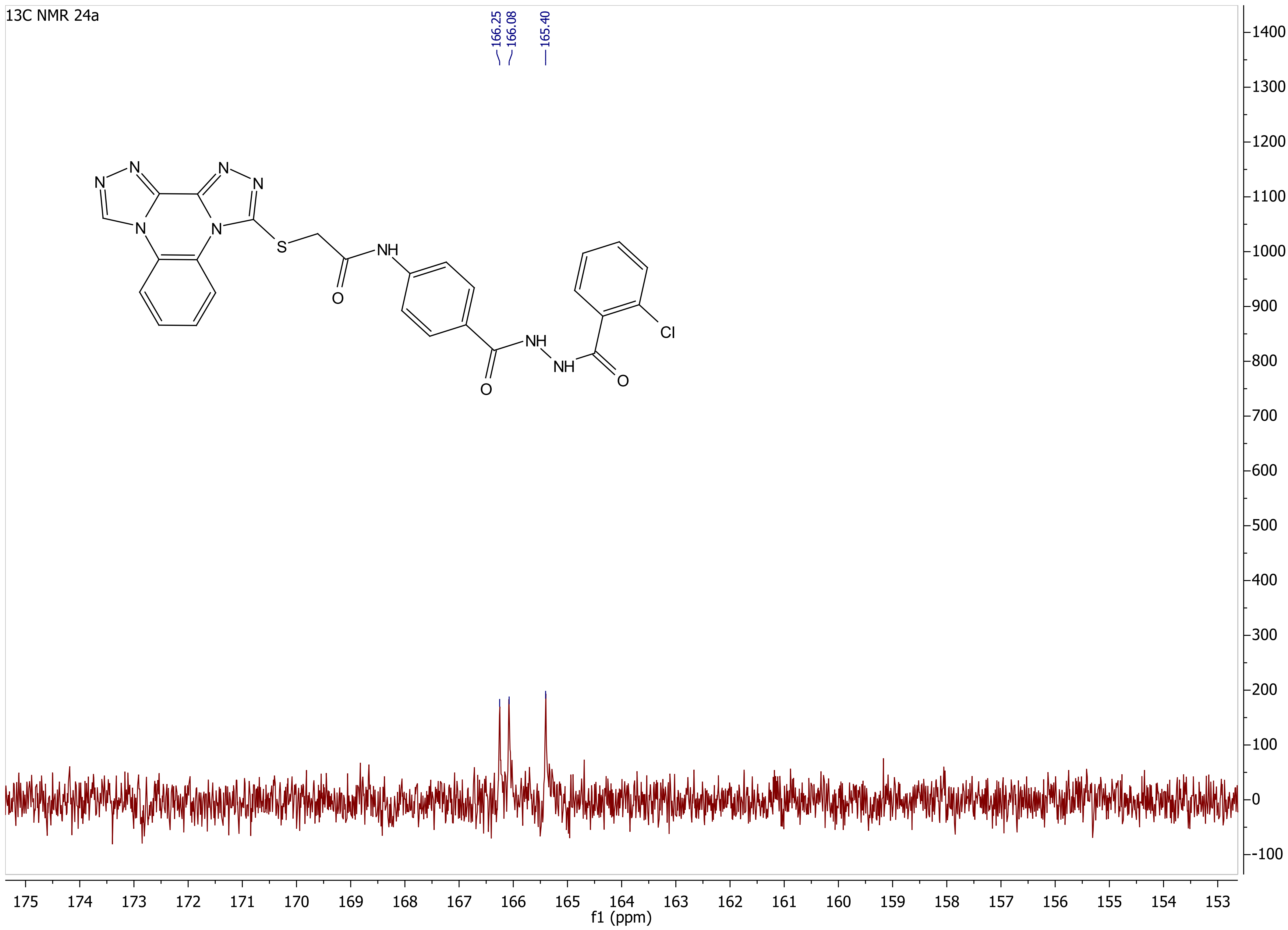
13C NMR 24a
166.25
166.08
165.40
N
N
N
N
N
N
S
NH
O
NH
NH
O
O
Cl
1400
1300
1200
1100
1000
900
800
700
600
500
400
300
200
100
0
-100
175
174
173
172
171
170
169
168
167
166
165
164
163
162
161
160
159
158
157
156
155
154
153
f1 (ppm)

Mass spec. of 24a

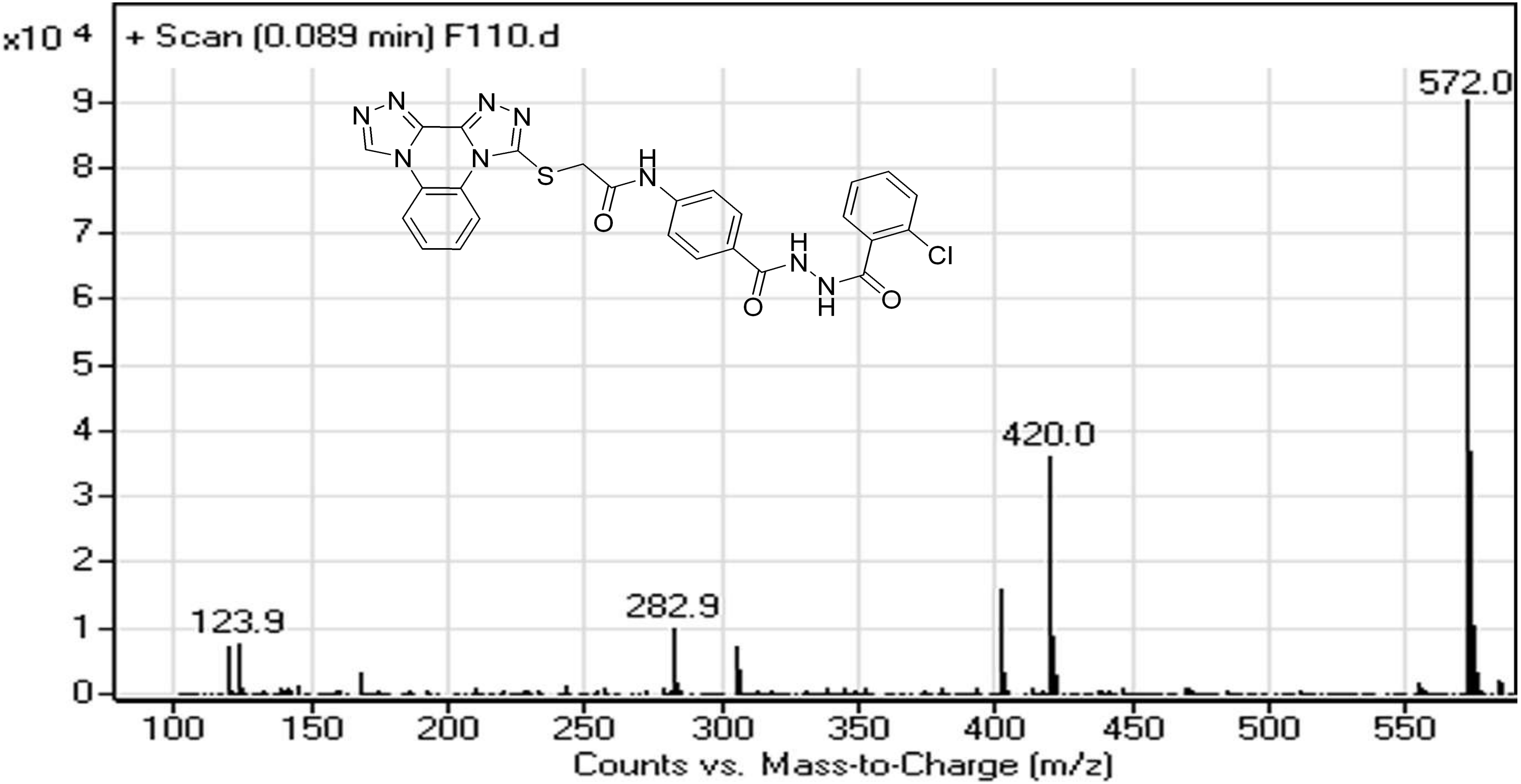

IR of compound 24b

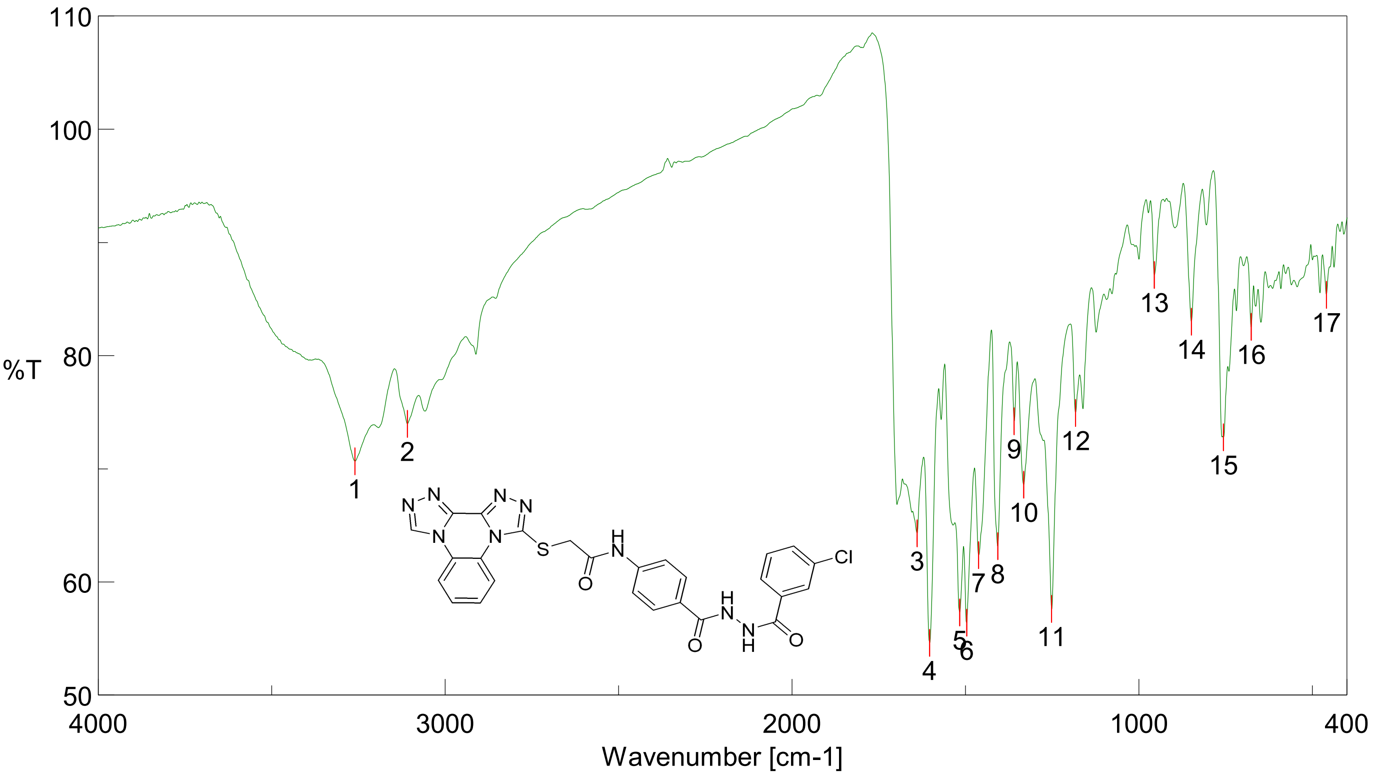

[Comments]

Sample name	F108
Comment	
User	
Division	
Company	KSU

[Detailed Information]

Creation date	11/1/2020 12:21 AM
Data array type	Linear data array
Horizontal axis	Wavenumber [cm-1]
Vertical axis	%T
Start	399.193 cm-1
End	4000.6 cm-1
Data interval	0.964233 cm-1
Data points	3736

[Measurement Information]

Model Name	FT/IR-6600typeA
Serial Number	A014661790
Measurement Date	10/28/2020 5:36 AM
Light Source	Standard
Detector	TGS
Accumulation	Auto (18)
Resolution	4 cm-1
Zero Filling	On
Apodization	Cosine
Gain	Auto (2)
Aperture	Auto (7.1 mm)
Scanning Speed	Auto (2 mm/sec)
Filter	Auto (10000 Hz)

[Result of Peak Picking]

No.	Position	Intensity	No.	Position	Intensity	No.	Position	Intensity
1	3260.07	70.6615	2	3108.69	73.964	3	1639.2	64.2974

[Result of Peak Picking]

No.	Position	Intensity	No.	Position	Intensity	No.	Position	Intensity
4	1603.52	54.6159	5	1516.74	57.292	6	1496.49	56.3844
7	1461.78	62.3667	8	1406.82	63.1568	9	1359.57	74.1972
10	1331.61	68.5721	11	1251.58	57.5998	12	1182.15	74.9142
13	955.555	87.1051	14	848.525	82.9756	15	755.959	72.7584
16	675.928	82.522	17	458.975	85.3512			

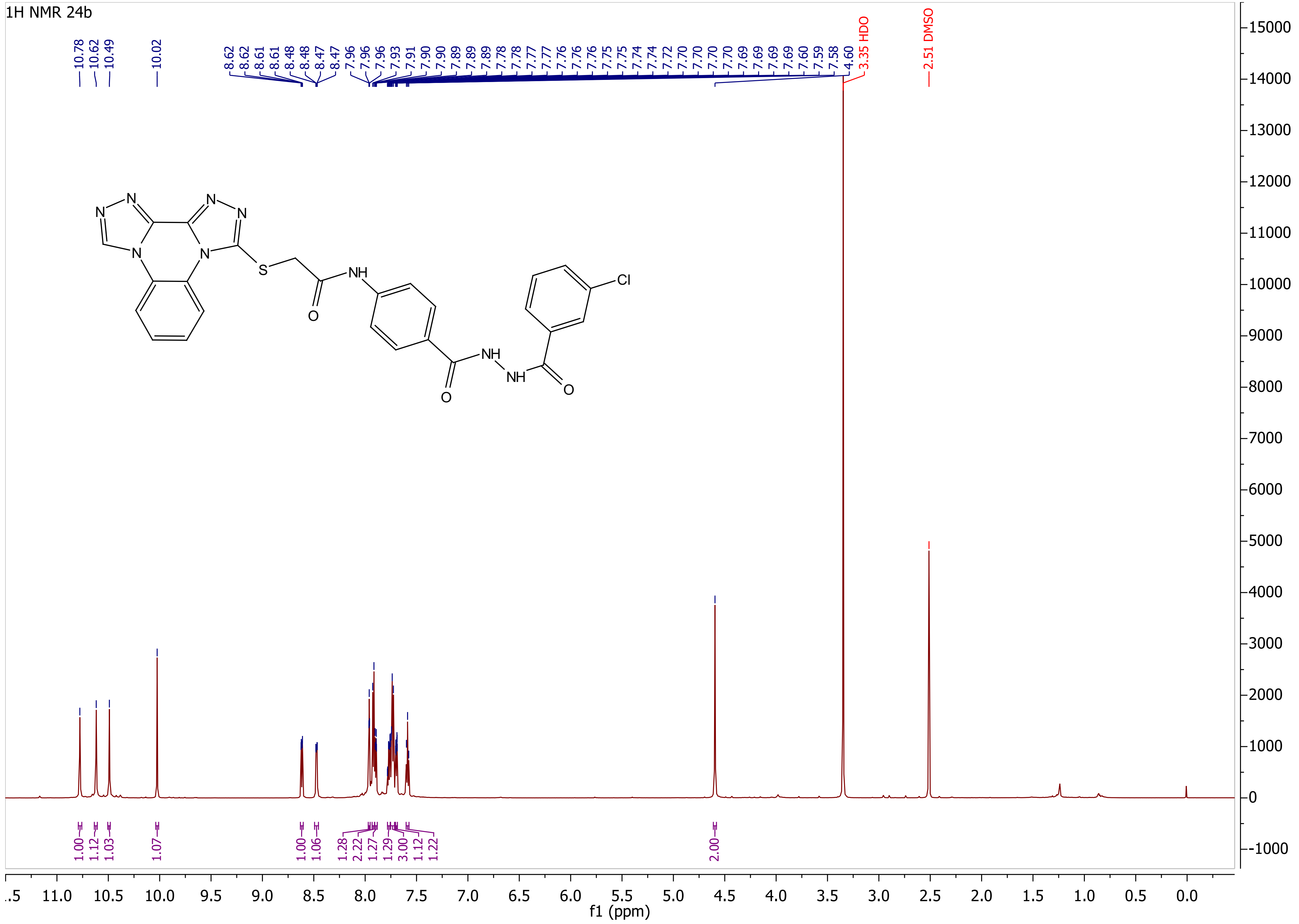
1H NMR 24b
10.78
10.62
10.49
10.02
8.62
8.62
8.61
8.61
8.48
8.48
8.47
8.47
7.96
7.96
7.96
7.93
7.91
7.90
7.90
7.89
7.89
7.89
7.78
7.78
7.77
7.77
7.76
7.76
7.76
7.75
7.75
7.74
7.74
7.72
7.70
7.70
7.70
7.69
7.69
7.69
7.69
7.60
7.59
7.58
4.60
3.35 HDO
2.51 DMSO
1.00
1.12
1.03
1.07
1.00
1.06
1.28
2.22
1.27
1.29
3.00
1.12
1.22
2.00
N
N
N
N
N
N
S
NH
O
NH
NH
O
O
Cl
f1 (ppm)

1H NMR 24b

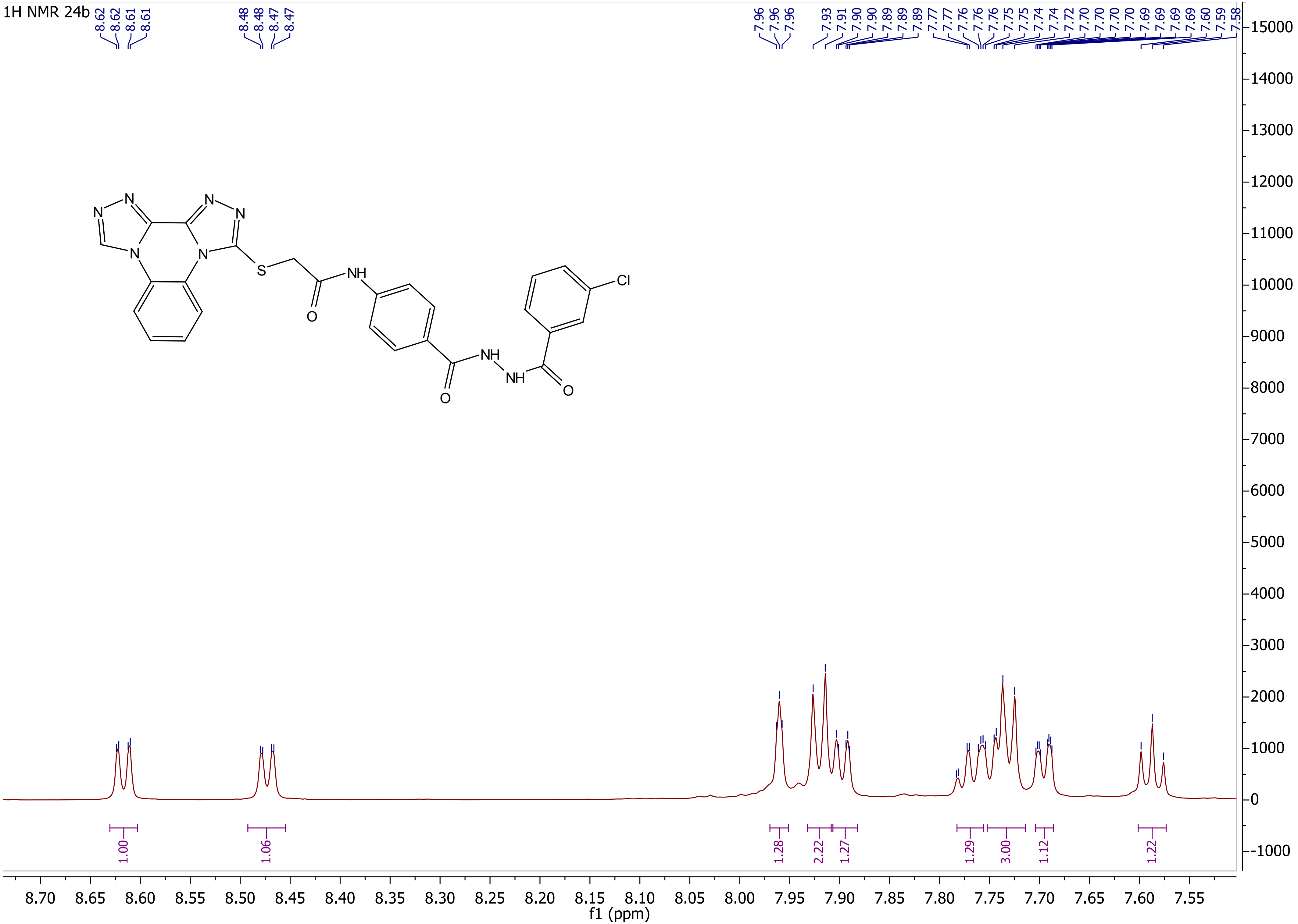

8.62
8.62
8.61
8.61
8.48
8.48
8.47
8.47
7.96
7.96
7.96
7.93
7.91
7.90
7.90
7.89
7.89
7.89
7.77
7.77
7.76
7.76
7.75
7.75
7.74
7.74
7.72
7.70
7.70
7.70
7.69
7.69
7.69
7.69
7.60
7.59
7.58
N
N
N
N
N
N
N
N
S
NH
O
NH
NH
O
O
Cl
1.00
1.06
1.28
2.22
1.27
1.29
3.00
1.12
1.22
f1 (ppm)

1H NMR 24b

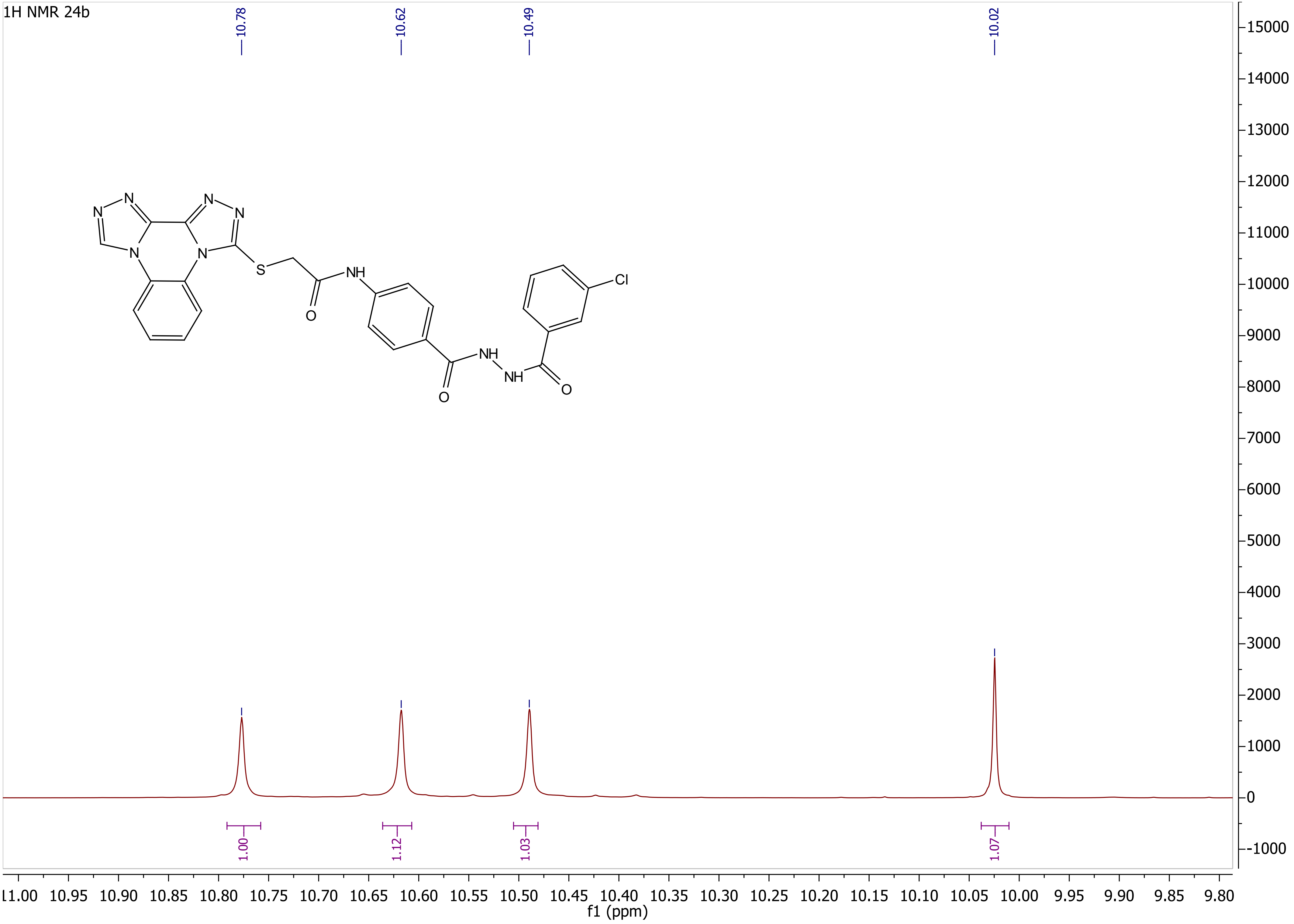

13C NMR 24b

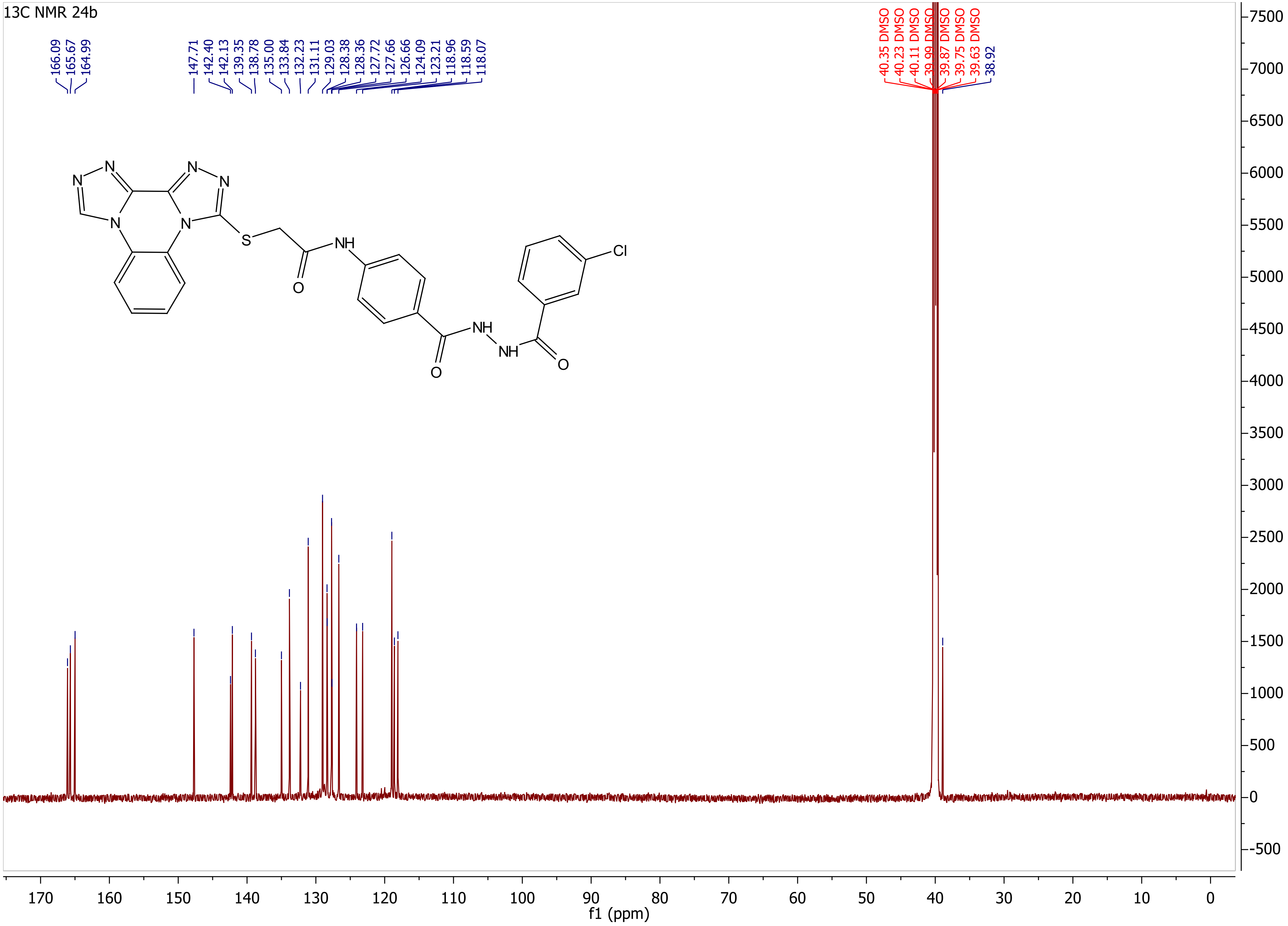

166.09
165.67
164.99
147.71
142.40
142.13
139.35
138.78
135.00
133.84
132.23
131.11
129.03
128.38
128.36
127.72
127.66
126.66
124.09
123.21
118.96
118.59
118.07
40.35 DMSO
40.23 DMSO
40.11 DMSO
39.99 DMSO
39.87 DMSO
39.75 DMSO
39.63 DMSO
38.92
f1 (ppm)

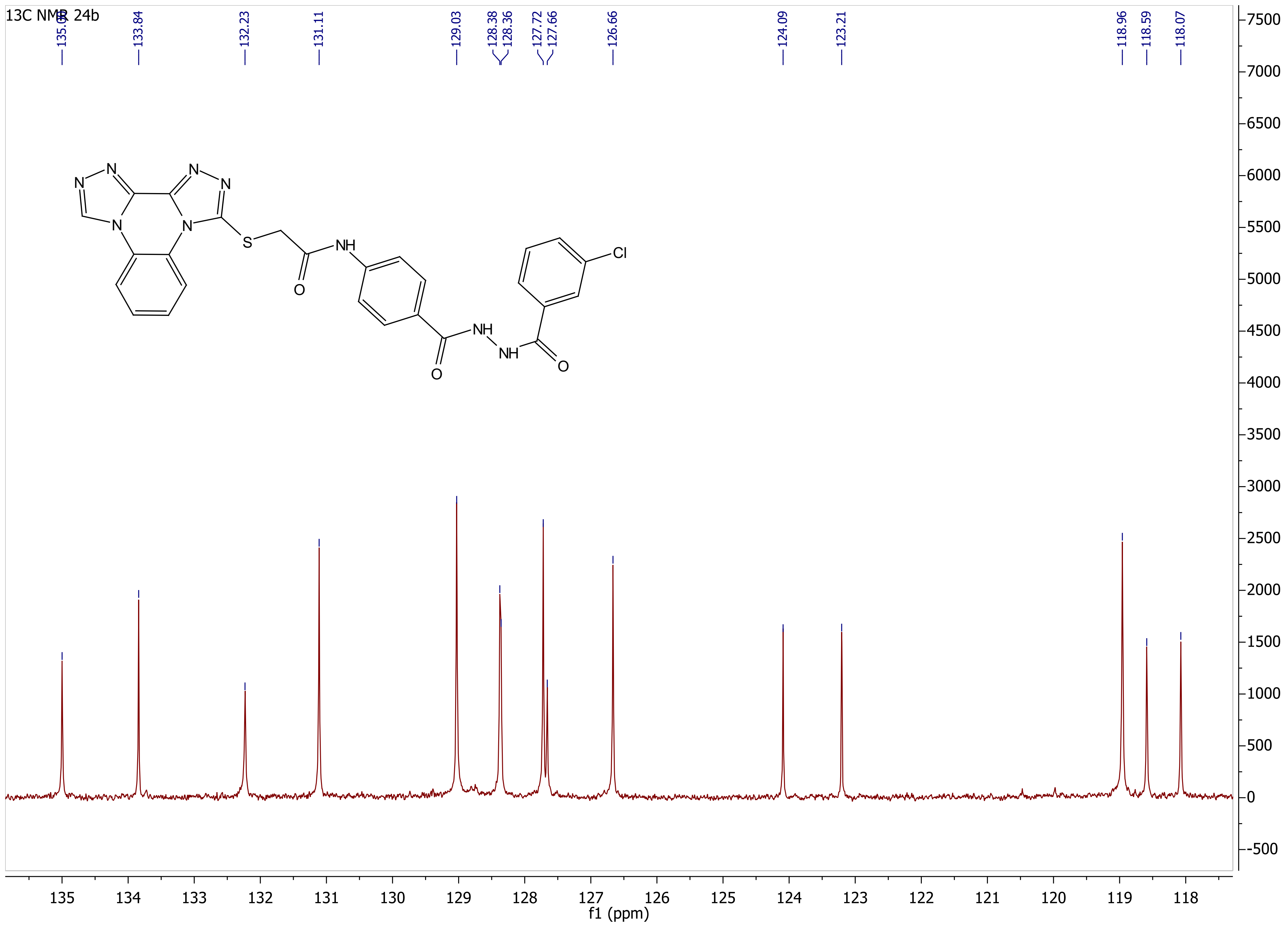
13C NMR 24b
133.84
132.23
131.11
129.03
128.38
128.36
127.72
127.66
126.66
124.09
123.21
118.96
118.59
118.07
N
N
N
N
N
N
S
NH
O
NH
NH
O
O
Cl
f1 (ppm)

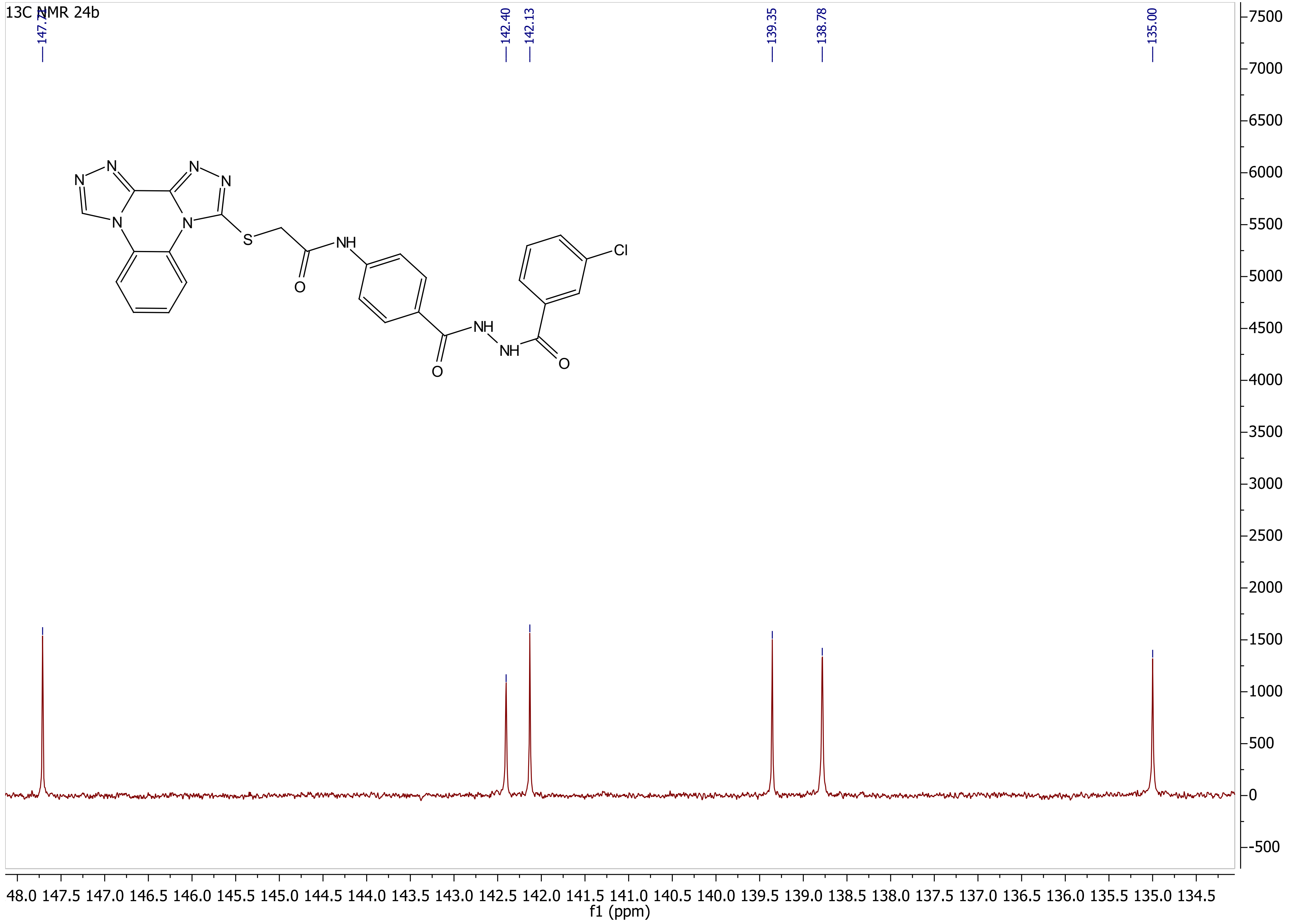
13C NMR 24b
147.7
142.40
142.13
139.35
138.78
135.00
N
N
N
N
N
N
S
NH
O
NH
NH
O
O
Cl
7500
7000
6500
6000
5500
5000
4500
4000
3500
3000
2500
2000
1500
1000
500
0
-500
48.0 147.5 147.0 146.5 146.0 145.5 145.0 144.5 144.0 143.5 143.0 142.5 142.0 141.5 141.0 140.5 140.0 139.5 139.0 138.5 138.0 137.5 137.0 136.5 136.0 135.5 135.0 134.5
f1 (ppm)

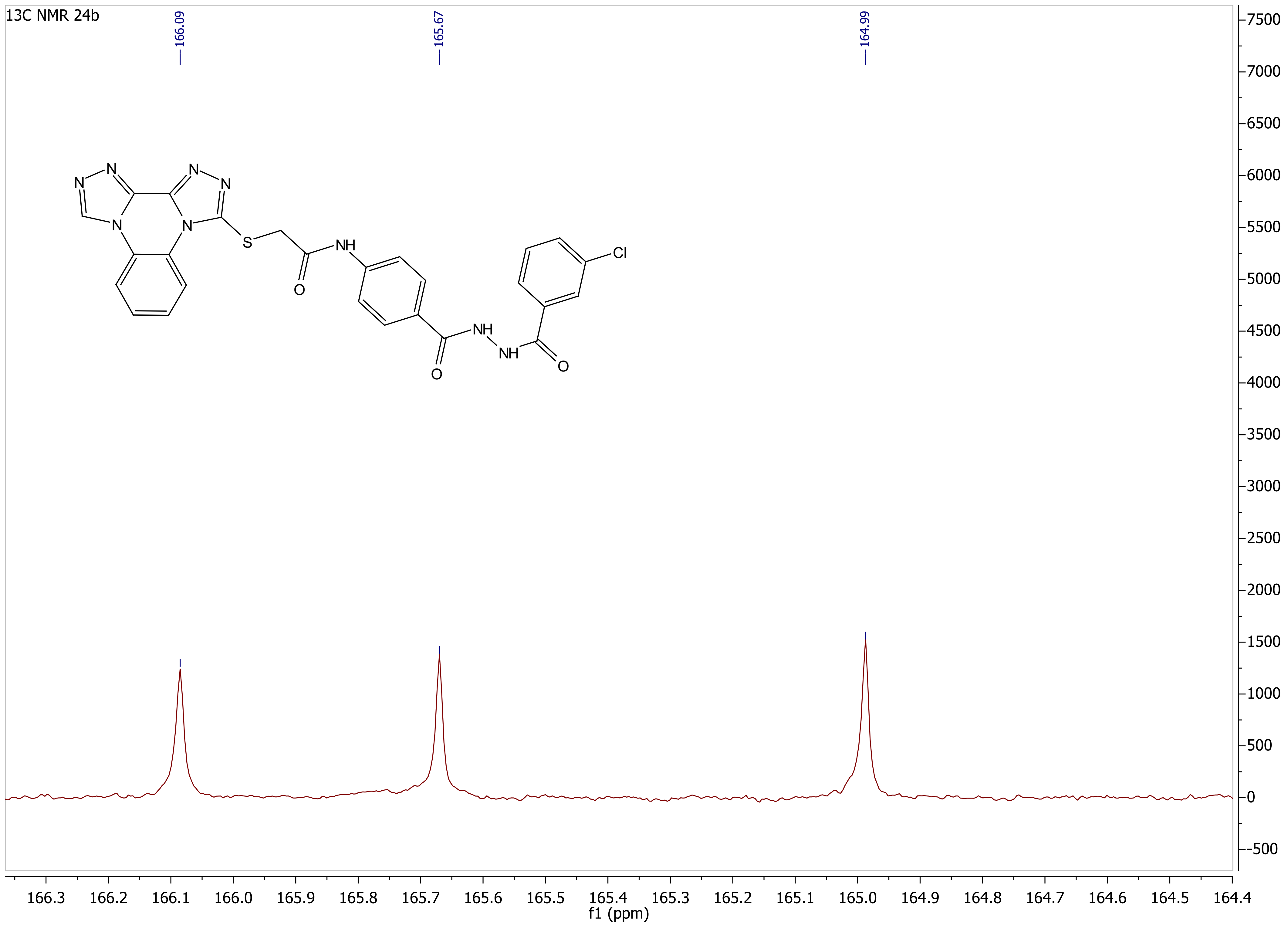

13C NMR 24b
166.09
165.67
164.99
N
N
N
N
N
N
N
N
S
O
NH
O
NH
NH
O
Cl
f1 (ppm)

Mass spec. of 24b

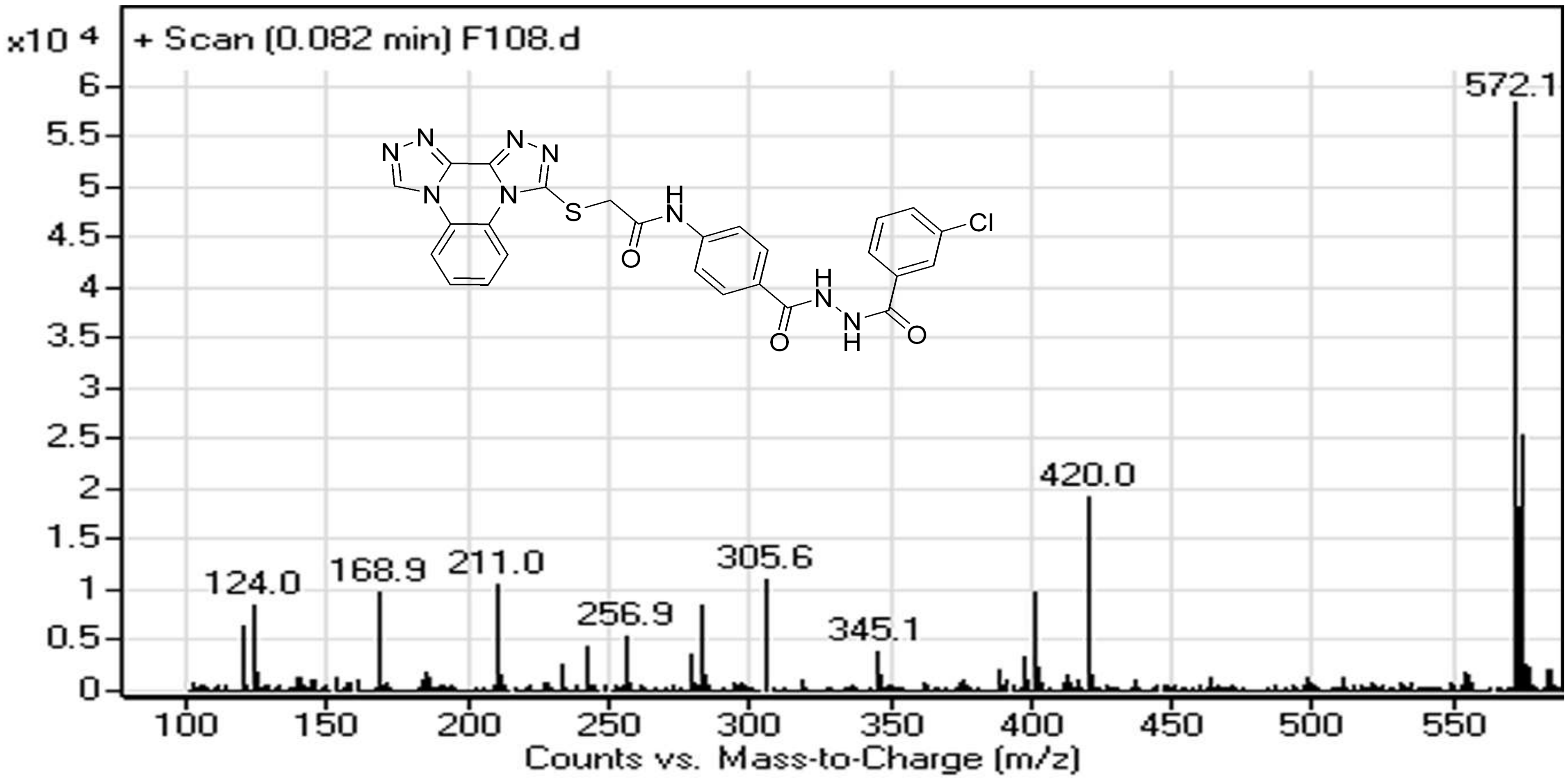

IR of compound 24c

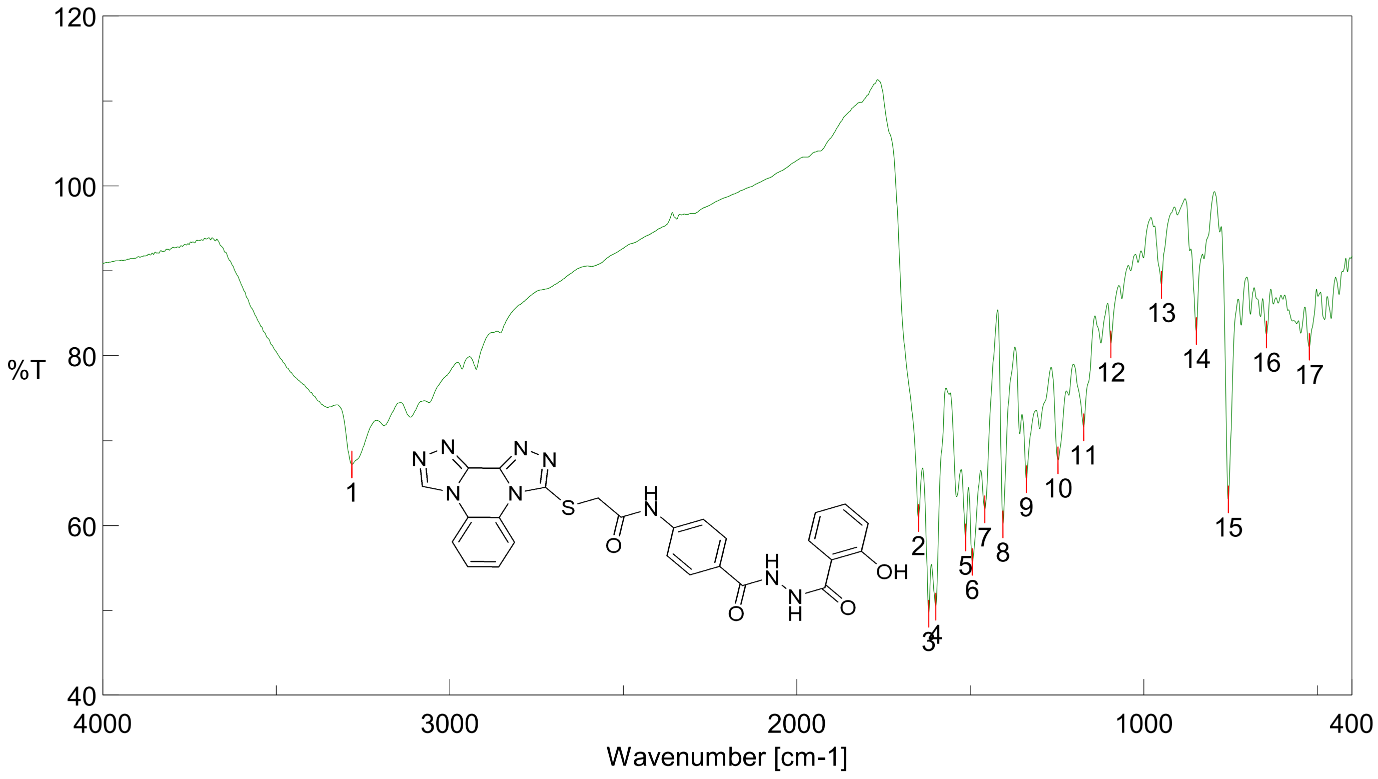

[Comments]
Sample name F111
Comment
User
Division
Company KSU

[Detailed Information]

Creation date	11/1/2020 12:26 AM
Data array type	Linear data array
Horizontal axis	Wavenumber [cm-1]
Vertical axis	%T
Start	399.193 cm-1
End	4000.6 cm-1
Data interval	0.964233 cm-1
Data points	3736

[Measurement Information]

Model Name	FT/IR-6600typeA
Serial Number	A014661790
Measurement Date	10/28/2020 5:44 AM
Light Source	Standard
Detector	TGS
Accumulation	Auto (19)
Resolution	4 cm-1
Zero Filling	On
Apodization	Cosine
Gain	Auto (2)
Aperture	Auto (7.1 mm)
Scanning Speed	Auto (2 mm/sec)
Filter	Auto (10000 Hz)

[Result of Peak Picking]

No.	Position	Intensity	No.	Position	Intensity	No.	Position	Intensity
1	3282.25	67.1703	2	1648.84	60.8623	3	1619.91	49.5665

[Result of Peak Picking]

No.	Position	Intensity	No.	Position	Intensity	No.	Position	Intensity
4	1599.66	50.3974	5	1513.85	58.5591	6	1494.56	55.6955
7	1457.92	61.8612	8	1405.85	60.088	9	1338.36	65.4155
10	1246.75	67.6479	11	1173.47	71.5227	12	1094.4	81.3154
13	948.806	88.3122	14	848.525	82.8906	15	755.959	63.0313
16	646.036	82.4815	17	522.615	81.0191			

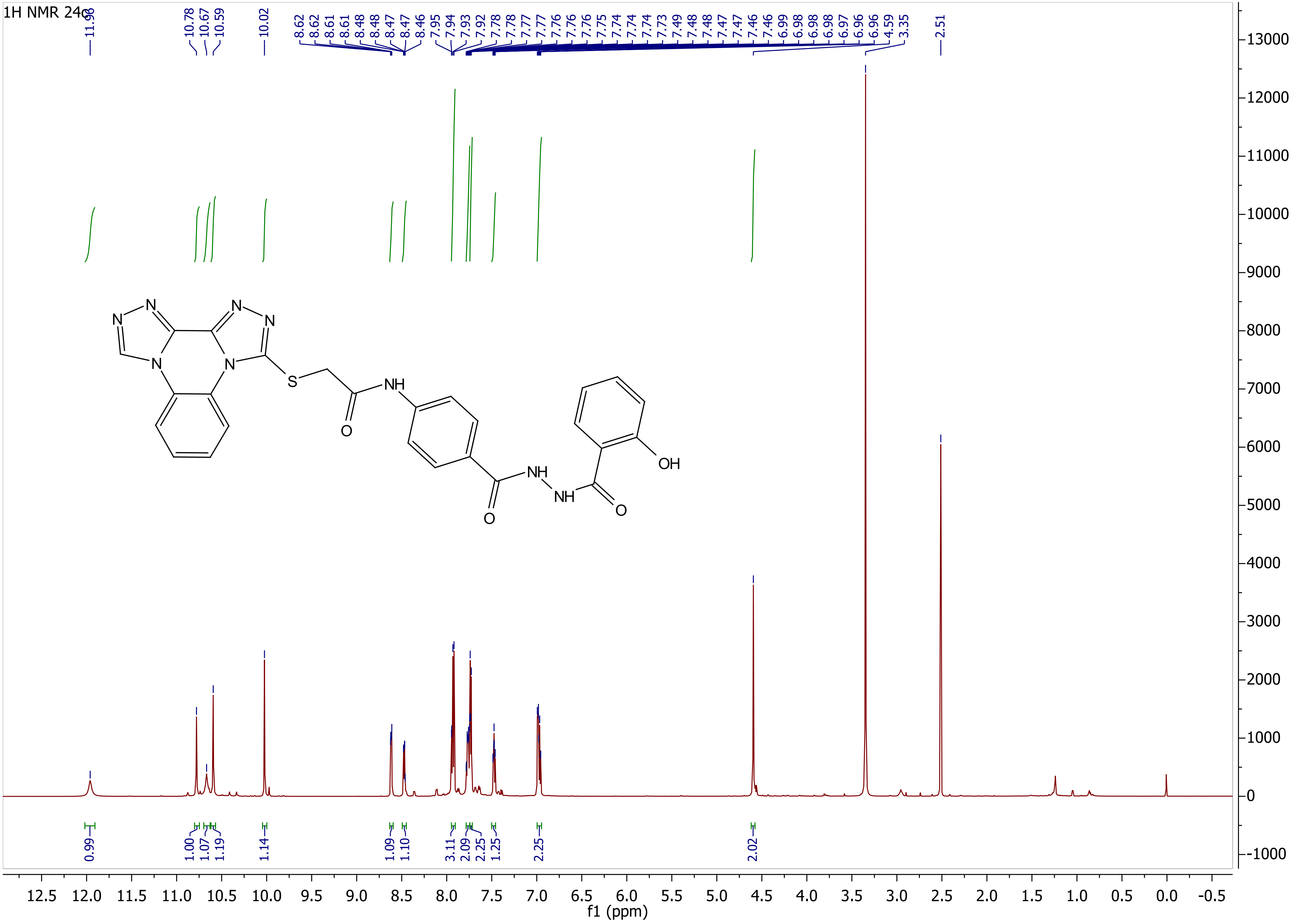
1H NMR 24c
11.96
10.78
10.67
10.59
10.02
8.62
8.62
8.61
8.61
8.48
8.48
8.47
8.47
8.46
7.95
7.94
7.93
7.92
7.78
7.78
7.77
7.77
7.76
7.76
7.76
7.75
7.74
7.74
7.74
7.73
7.49
7.48
7.48
7.47
7.47
7.46
7.46
6.99
6.98
6.98
6.98
6.97
6.96
6.96
4.59
3.35
2.51
0.99
1.00
1.07
1.19
1.14
1.09
1.10
3.11
2.09
2.25
1.25
2.25
2.02
N
N
N
N
N
N
S
NH
O
NH
NH
O
O
OH
f1 (ppm)

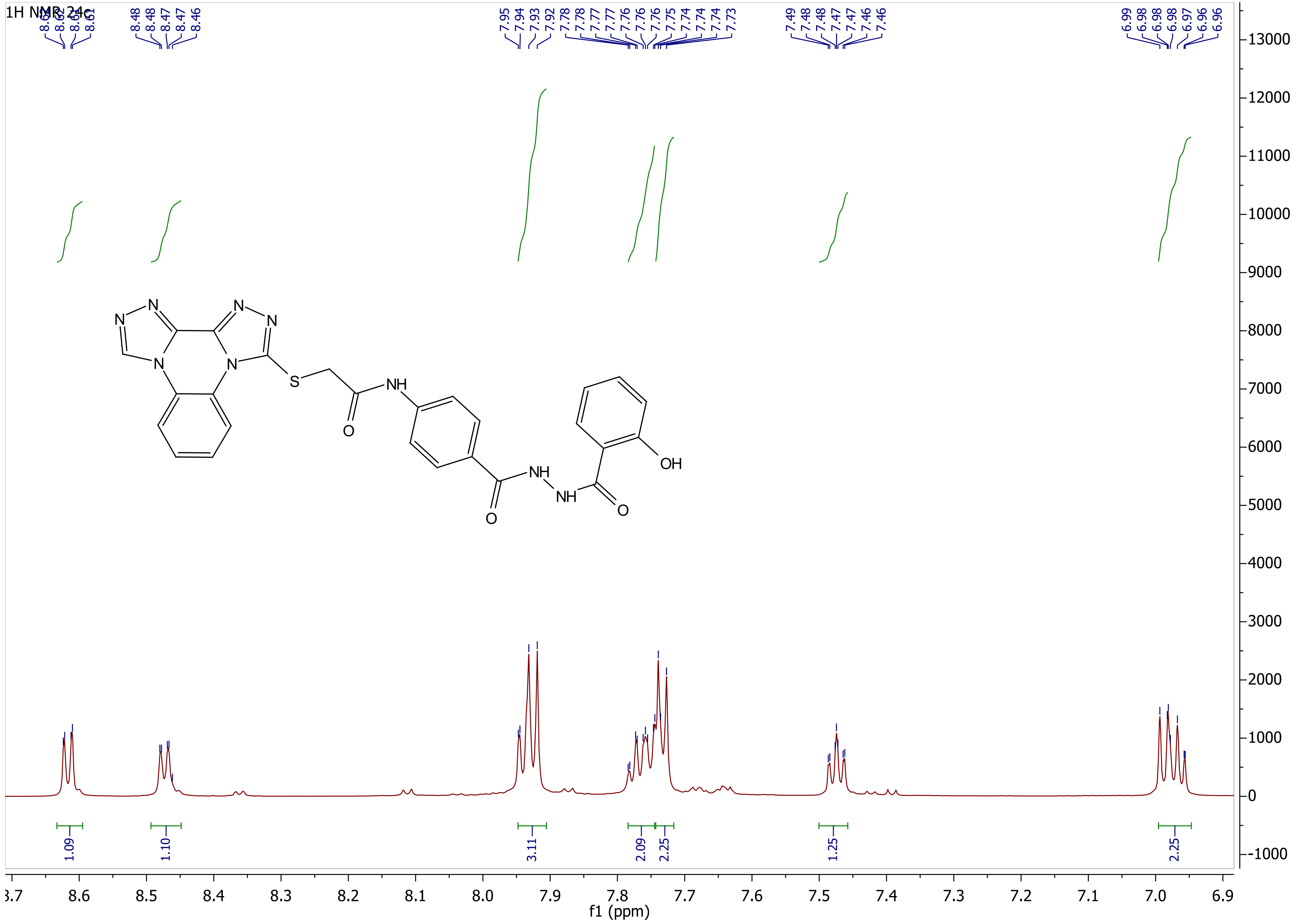

1H NMR 24c
OH
NH
NH
NH
S
f1 (ppm)

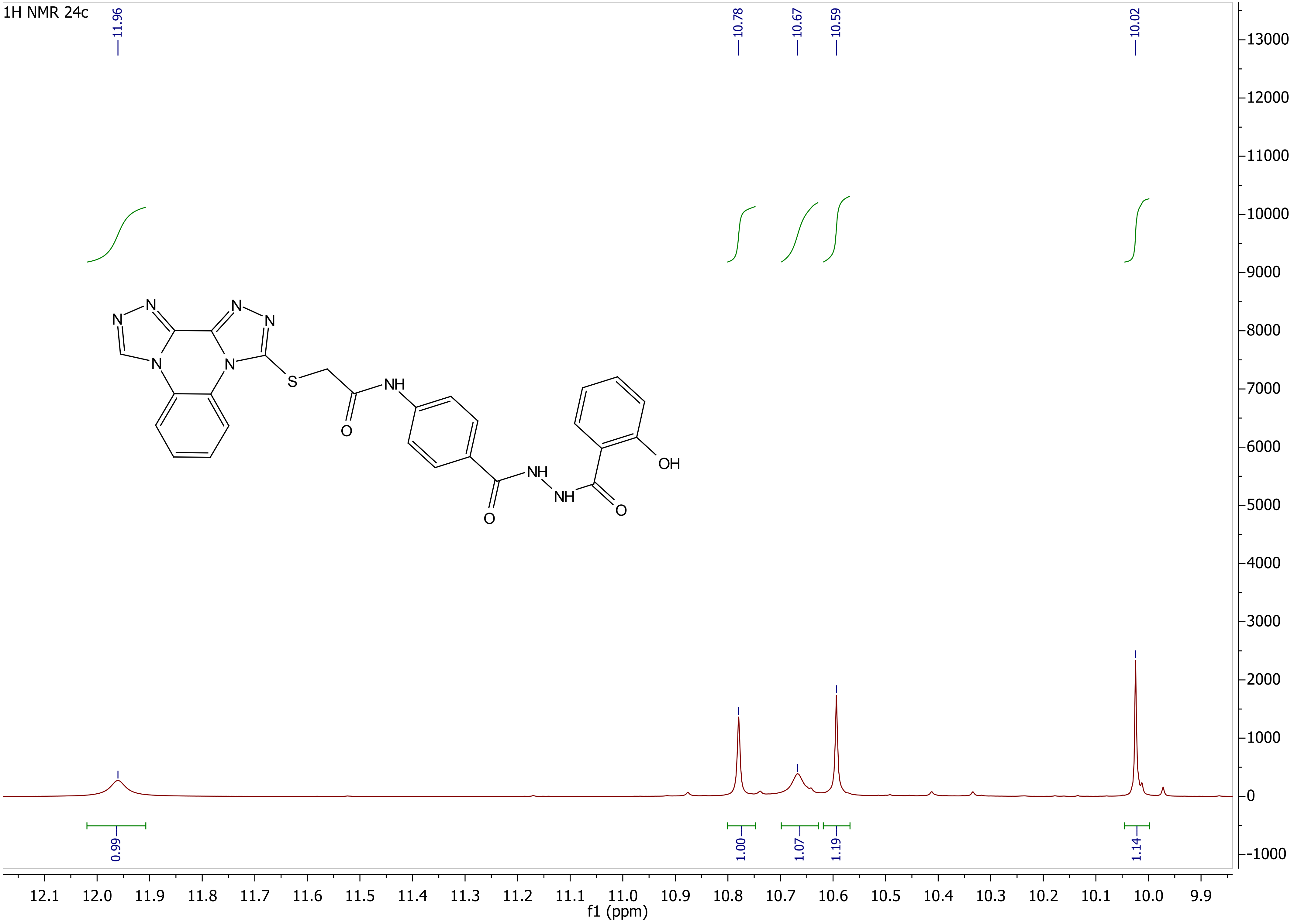
1H NMR 24c
11.96
10.78
10.67
10.59
10.02
0.99
1.00
1.07
1.19
1.14
N
N
N
N
N
N
S
NH
O
NH
NH
O
O
OH
13000
12000
11000
10000
9000
8000
7000
6000
5000
4000
3000
2000
1000
0
-1000
12.1
12.0
11.9
11.8
11.7
11.6
11.5
11.4
11.3
11.2
11.1
11.0
10.9
10.8
10.7
10.6
10.5
10.4
10.3
10.2
10.1
10.0
9.9
f1 (ppm)

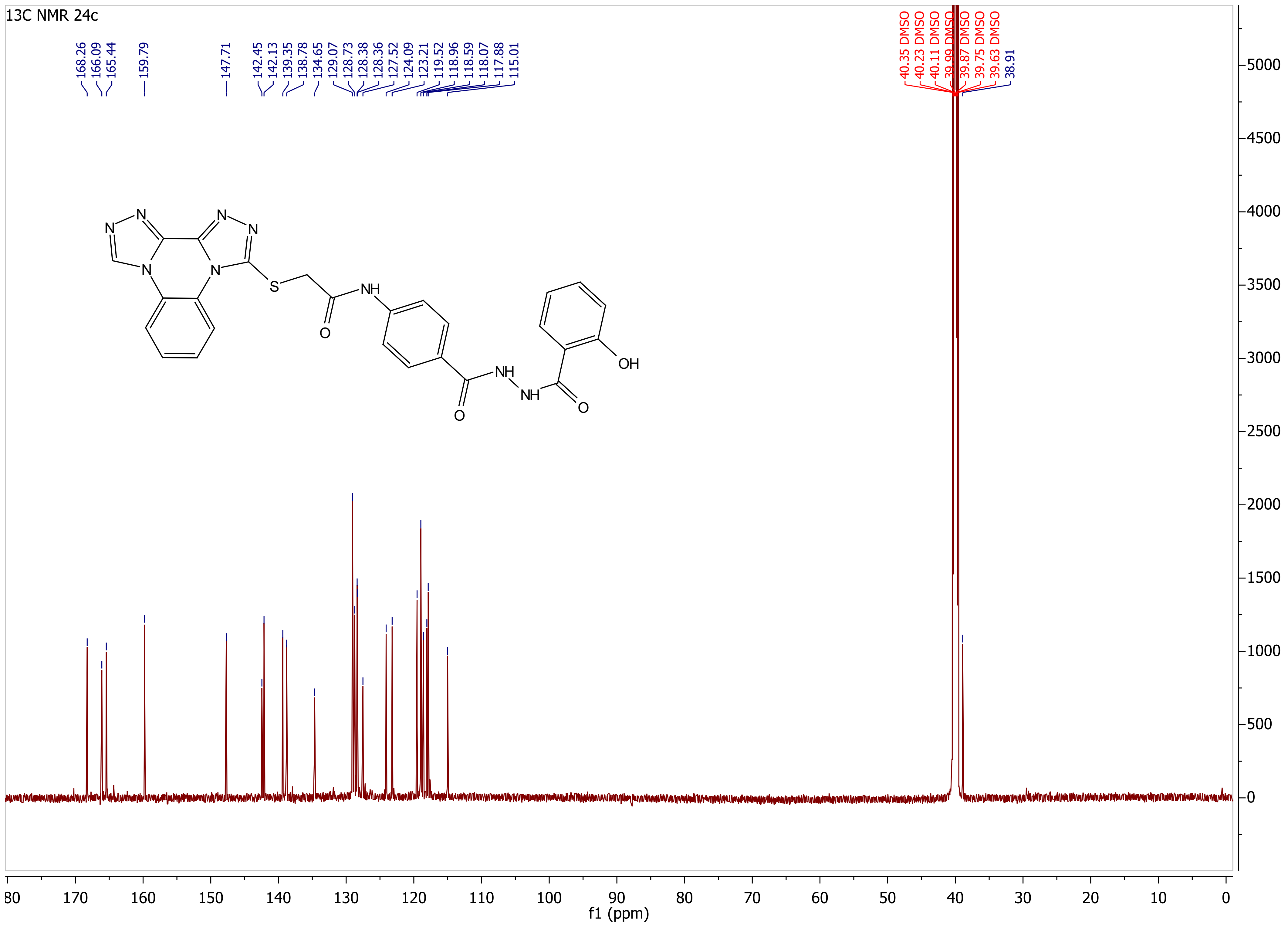

13C NMR 24c
168.26
166.09
165.44
159.79
147.71
142.45
142.13
139.35
138.78
134.65
129.07
128.73
128.38
128.36
127.52
124.09
123.21
119.52
118.96
118.59
118.07
117.88
115.01
40.35 DMSO
40.23 DMSO
40.11 DMSO
39.99 DMSO
39.87 DMSO
39.75 DMSO
39.63 DMSO
38.91
N
N
N
S
NH
O
NH
NH
O
O
OH
f1 (ppm)

13C NMR 24c

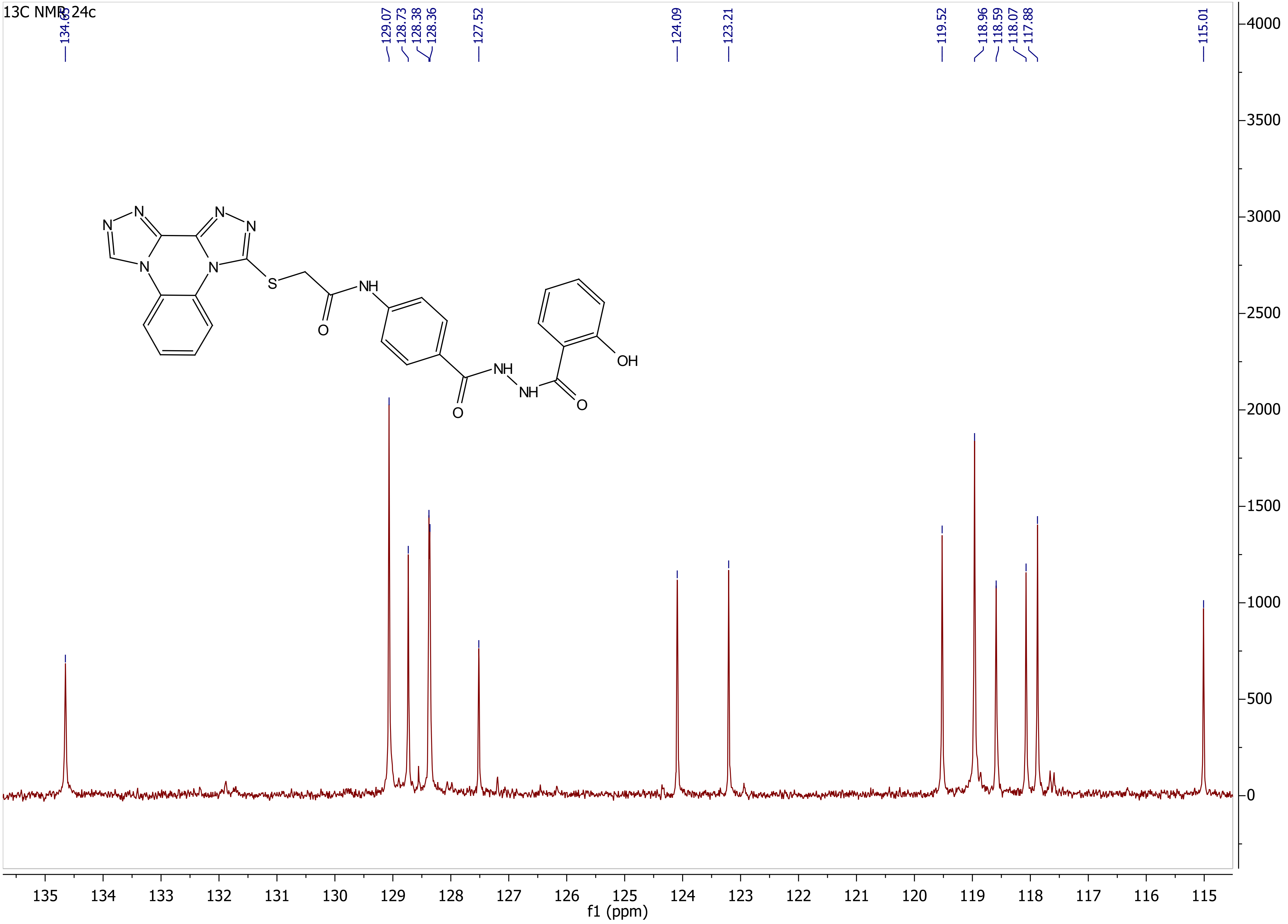

134.65
129.07
128.73
128.38
128.36
127.52
124.09
123.21
119.52
118.96
118.59
118.07
117.88
115.01
f1 (ppm)

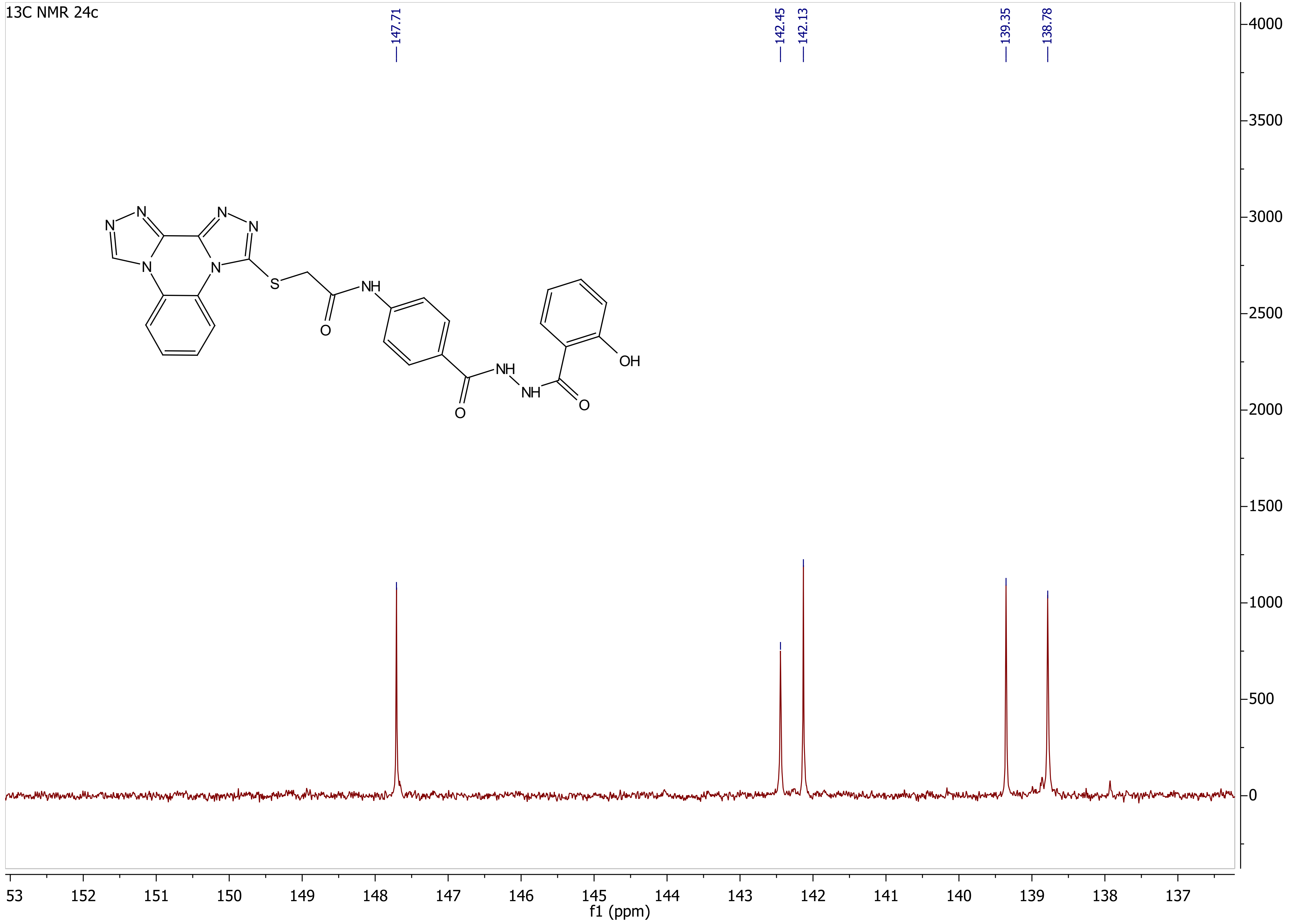
13C NMR 24c
147.71
142.45
142.13
139.35
138.78
N
N
N
N
N
N
S
NH
O
NH
NH
O
O
OH
f1 (ppm)
53
152
151
150
149
148
147
146
145
144
143
142
141
140
139
138
137
4000
3500
3000
2500
2000
1500
1000
500
0

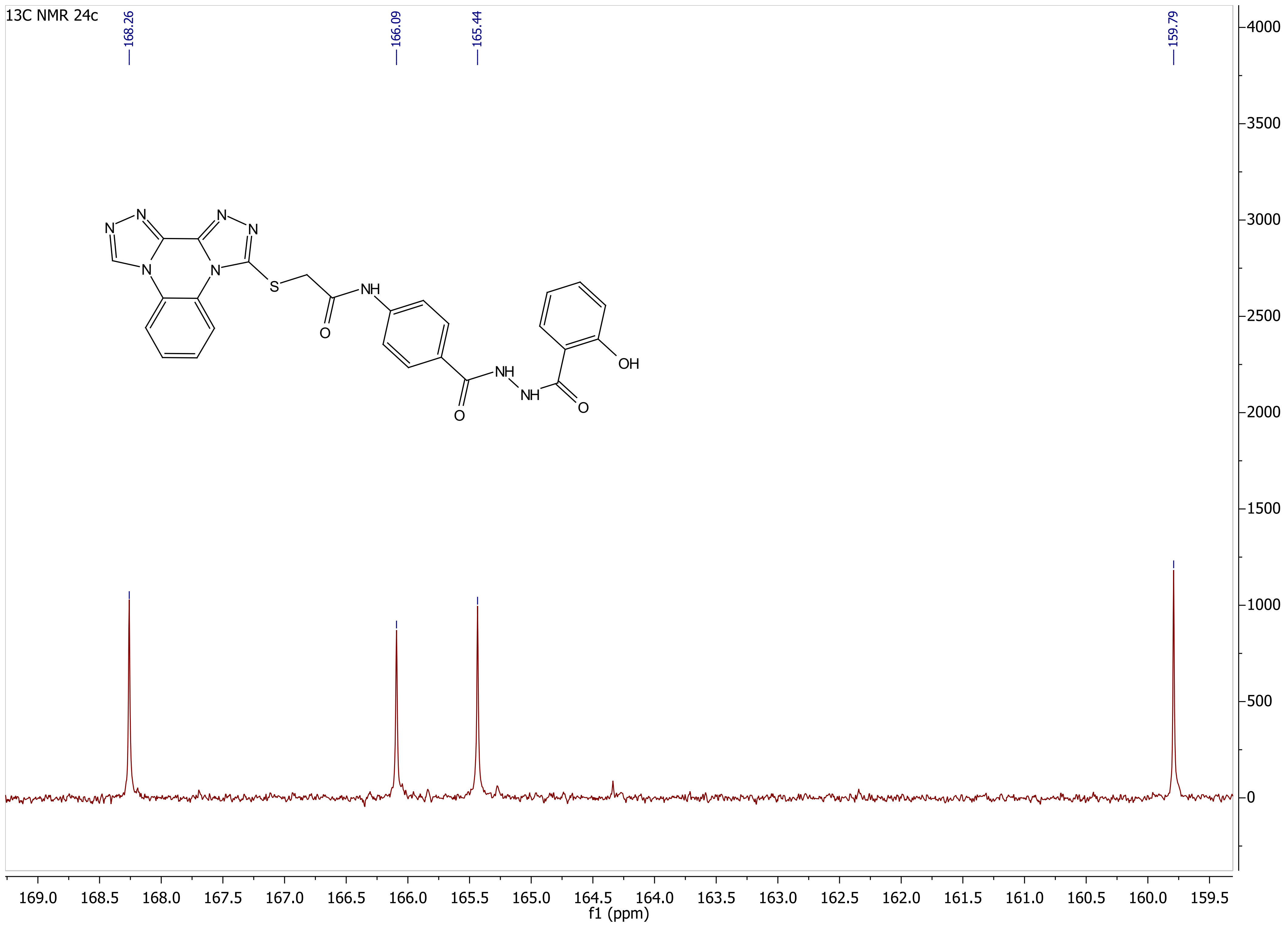
13C NMR 24c
168.26
166.09
165.44
159.79
N
N
N
N
N
N
N
N
S
O
NH
NH
NH
O
O
OH
4000
3500
3000
2500
2000
1500
1000
500
0
169.0 168.5 168.0 167.5 167.0 166.5 166.0 165.5 165.0 164.5 164.0 163.5 163.0 162.5 162.0 161.5 161.0 160.5 160.0 159.5
f1 (ppm)

Mass spec. of 24c

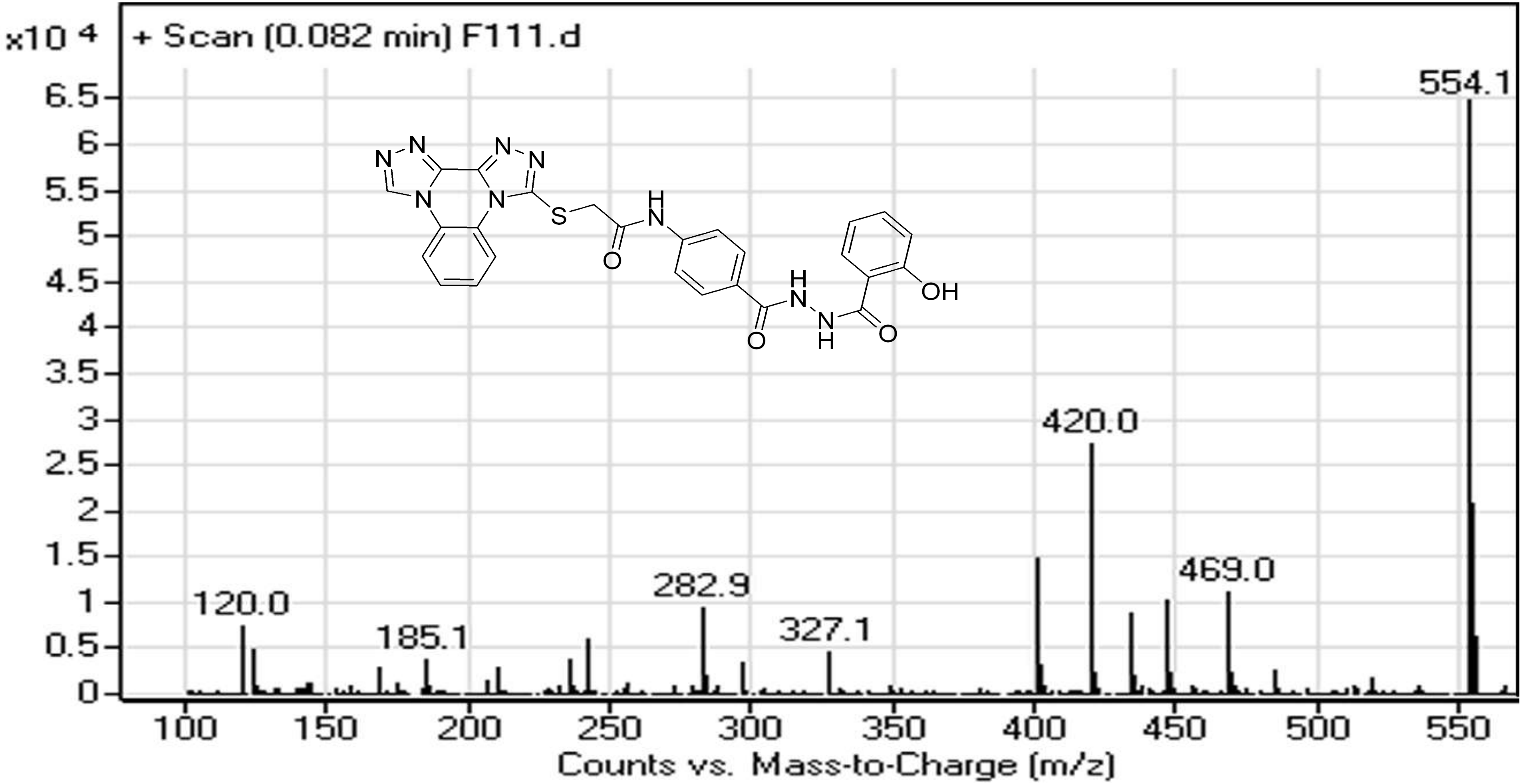

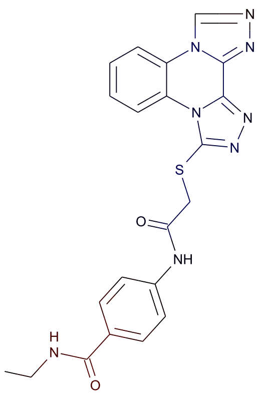

$C_{21}H_{18}N_8O_2S$

Molecular Weight: 446.48502

ALogP: 2.442

Rotatable Bonds: 6

Acceptors: 7

Donors: 2

Structural Similar Compounds

Name	Ochratoxin a	Lenampicillin .HCl (Free base form)	Prazosin .HCl (Free base form)
Structure	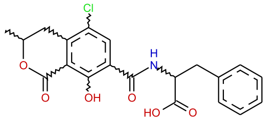	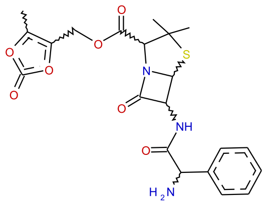	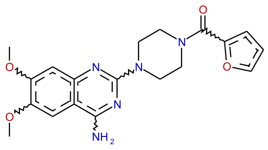
Actual Endpoint	Toxic	Non-Toxic	Toxic
Predicted Endpoint	Toxic	Non-Toxic	Toxic
Distance	0.636	0.666	0.671
Reference	Toxicol Appl Pharmacol 37(2):331-8; 1976	Chemotherapy 32:130-145; 1984	Oyo Yakuri 17:57-62; 1979

Model Prediction

Prediction: Non-Toxic

Probability: 0.461

Enrichment: 0.876

Bayesian Score: -2.444

Mahalanobis Distance: 9.770

Mahalanobis Distance p-value: 0.037

Prediction: Positive if the Bayesian score is above the estimated best cutoff value from minimizing the false positive and false negative rate.
Probability: The esimated probability that the sample is in the positive category. This assumes that the Bayesian score follows a normal distribution and is different from the prediction using a cutoff.
Enrichment: An estimate of enrichment, that is, the increased likelihood (versus random) of this sample being in the category.
Bayesian Score: The standard Laplacian-modified Bayesian score.
Mahalanobis Distance: The Mahalanobis distance (MD) is the distance to the center of the training data. The larger the MD, the less trustworthy the prediction.
Mahalanobis Distance p-value: The p-value gives the fraction of training data with an MD greater than or equal to the one for the given sample, assuming normally distributed data. The smaller the p-value, the less trustworthy the prediciton. For highly non-normal X properties (e.g., fingerprints), the MD p-value is wildly inaccurate.

Model Applicability

Unknown features are fingerprint features in the query molecule, but not found in the training set.

1. All properties and OPS components are within expected ranges.

Feature Contribution

Top features for positive contribution

Fingerprint	Bit/Smiles	Feature Structure	Score	Toxic in training set
SCFP_6	282594097	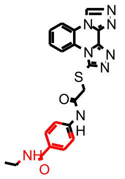 [*]NC(=O)[c]1:[cH]:[cH]:[*]:[cH]:[cH]:1	0.441	3 out of 3

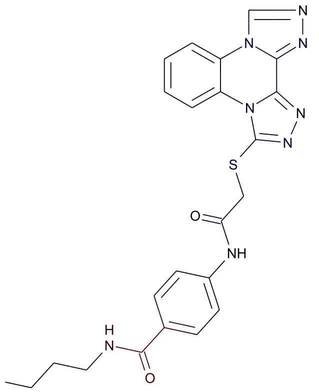

$C_{23}H_{22}N_8O_2S$
Molecular Weight: 474.53818
ALogP: 3.422
Rotatable Bonds: 8
Acceptors: 7
Donors: 2

Model Prediction

Prediction: Non-Toxic
Probability: 0.458
Enrichment: 0.871
Bayesian Score: -2.518
Mahalanobis Distance: 10.431
Mahalanobis Distance p-value: 0.00645

Prediction: Positive if the Bayesian score is above the estimated best cutoff value from minimizing the false positive and false negative rate.
Probability: The esimated probability that the sample is in the positive category. This assumes that the Bayesian score follows a normal distribution and is different from the prediction using a cutoff.
Enrichment: An estimate of enrichment, that is, the increased likelihood (versus random) of this sample being in the category.
Bayesian Score: The standard Laplacian-modified Bayesian score.
Mahalanobis Distance: The Mahalanobis distance (MD) is the distance to the center of the training data. The larger the MD, the less trustworthy the prediction.
Mahalanobis Distance p-value: The p-value gives the fraction of training data with an MD greater than or equal to the one for the given sample, assuming normally distributed data. The smaller the p-value, the less trustworthy the prediciton. For highly non-normal X properties (e.g., fingerprints), the MD p-value is wildly inaccurate.

Structural Similar Compounds

Name	Acemetacin	Hydrocortisone-17-butyrate-21-propionate	LY171883
Structure	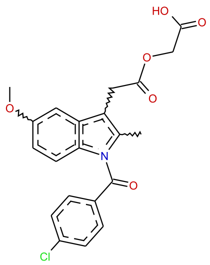	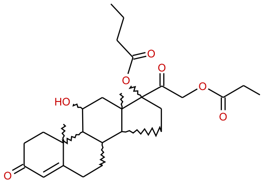	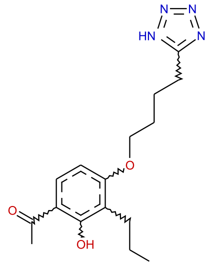
Actual Endpoint	Non-Toxic	Toxic	Non-Toxic
Predicted Endpoint	Non-Toxic	Toxic	Non-Toxic
Distance	0.667	0.680	0.684
Reference	Oyo Yakuri 22(6):777-786; 1981	Oyo Yakuri 21:441-466; 1981	Fundam Appl Toxicol 10(4):672-81; 1988

Model Applicability

Unknown features are fingerprint features in the query molecule, but not found in the training set.

1. All properties and OPS components are within expected ranges.

Feature Contribution

Top features for positive contribution

Fingerprint	Bit/Smiles	Feature Structure	Score	Toxic in training set
SCFP_6	282594097	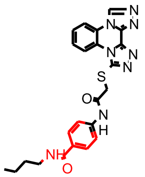 [*]NC(=O)[c]1:[cH]:[cH]:[*]:[cH]:[cH]:1	0.441	3 out of 3

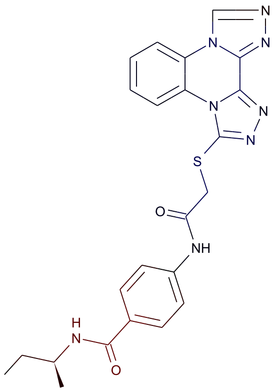

$C_{23}H_{22}N_8O_2S$
Molecular Weight: 474.53818
ALogP: 3.343
Rotatable Bonds: 7
Acceptors: 7
Donors: 2

Model Prediction

Prediction: Non-Toxic
Probability: 0.457
Enrichment: 0.869
Bayesian Score: -2.546
Mahalanobis Distance: 8.980
Mahalanobis Distance p-value: 0.183

Prediction: Positive if the Bayesian score is above the estimated best cutoff value from minimizing the false positive and false negative rate.
Probability: The esimated probability that the sample is in the positive category. This assumes that the Bayesian score follows a normal distribution and is different from the prediction using a cutoff.
Enrichment: An estimate of enrichment, that is, the increased likelihood (versus random) of this sample being in the category.
Bayesian Score: The standard Laplacian-modified Bayesian score.
Mahalanobis Distance: The Mahalanobis distance (MD) is the distance to the center of the training data. The larger the MD, the less trustworthy the prediction.
Mahalanobis Distance p-value: The p-value gives the fraction of training data with an MD greater than or equal to the one for the given sample, assuming normally distributed data. The smaller the p-value, the less trustworthy the prediciton. For highly non-normal X properties (e.g., fingerprints), the MD p-value is wildly inaccurate.

Structural Similar Compounds

Name	Ochratoxin a	Acemetacin	Amsacrine
Structure	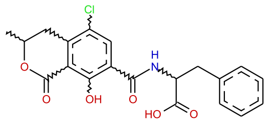	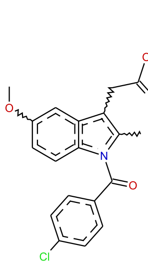	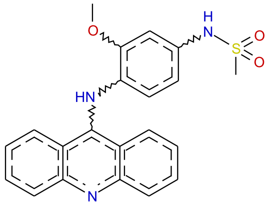
Actual Endpoint	Toxic	Non-Toxic	Toxic
Predicted Endpoint	Toxic	Non-Toxic	Toxic
Distance	0.655	0.659	0.681
Reference	Toxicol Appl Pharmacol 37(2):331-8; 1976	Oyo Yakuri 22(6):777-786; 1981	Fundam Appl Toxicol 7(2):214-20; 1986

Model Applicability

Unknown features are fingerprint features in the query molecule, but not found in the training set.

1. All properties and OPS components are within expected ranges.

Feature Contribution

Top features for positive contribution				
**Fingerprint**	**Bit/Smiles**	**Feature Structure**	**Score**	**Toxic in training set**
SCFP_6	282594097	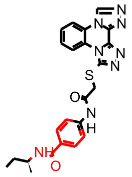 [*]NC(=O)[c]1:[cH]:[cH]:[*]:[cH]:[cH]:1	0.441	3 out of 3

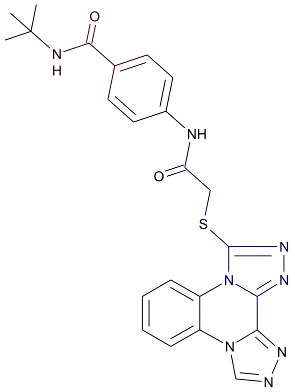

$C_{23}H_{22}N_8O_2S$
Molecular Weight: 474.53818
ALogP: 3.025
Rotatable Bonds: 6
Acceptors: 7
Donors: 2

Model Prediction

Prediction: Non-Toxic
Probability: 0.422
Enrichment: 0.802
Bayesian Score: -3.675
Mahalanobis Distance: 8.484
Mahalanobis Distance p-value: 0.374

Prediction: Positive if the Bayesian score is above the estimated best cutoff value from minimizing the false positive and false negative rate.
Probability: The esimated probability that the sample is in the positive category. This assumes that the Bayesian score follows a normal distribution and is different from the prediction using a cutoff.
Enrichment: An estimate of enrichment, that is, the increased likelihood (versus random) of this sample being in the category.
Bayesian Score: The standard Laplacian-modified Bayesian score.
Mahalanobis Distance: The Mahalanobis distance (MD) is the distance to the center of the training data. The larger the MD, the less trustworthy the prediction.
Mahalanobis Distance p-value: The p-value gives the fraction of training data with an MD greater than or equal to the one for the given sample, assuming normally distributed data. The smaller the p-value, the less trustworthy the prediciton. For highly non-normal X properties (e.g., fingerprints), the MD p-value is wildly inaccurate.

Structural Similar Compounds

Name	Ochratoxin a	Amsacrine	Acemetacin
Structure	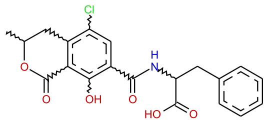	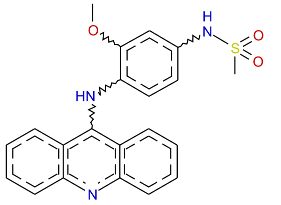	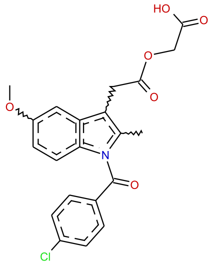
Actual Endpoint	Toxic	Toxic	Non-Toxic
Predicted Endpoint	Toxic	Toxic	Non-Toxic
Distance	0.628	0.661	0.671
Reference	Toxicol Appl Pharmacol 37(2):331-8; 1976	Fundam Appl Toxicol 7(2):214-20; 1986	Oyo Yakuri 22(6):777-786; 1981

Model Applicability

Unknown features are fingerprint features in the query molecule, but not found in the training set.

1. All properties and OPS components are within expected ranges.

Feature Contribution

Top features for positive contribution

Fingerprint	Bit/Smiles	Feature Structure	Score	Toxic in training set
SCFP_6	282594097	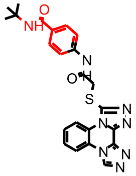 [*]NC(=O)[c]1:[cH]:[cH]:[*]:[cH]:[cH]:1	0.441	3 out of 3

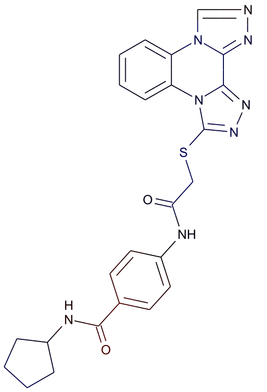

$C_{24}H_{22}N_8O_2S$
Molecular Weight: 486.54888
ALogP: 3.496
Rotatable Bonds: 6
Acceptors: 7
Donors: 2

Model Prediction

Prediction: Non-Toxic
Probability: 0.427
Enrichment: 0.813
Bayesian Score: -3.485
Mahalanobis Distance: 9.516
Mahalanobis Distance p-value: 0.0657

Prediction: Positive if the Bayesian score is above the estimated best cutoff value from minimizing the false positive and false negative rate.
Probability: The esimated probability that the sample is in the positive category. This assumes that the Bayesian score follows a normal distribution and is different from the prediction using a cutoff.
Enrichment: An estimate of enrichment, that is, the increased likelihood (versus random) of this sample being in the category.
Bayesian Score: The standard Laplacian-modified Bayesian score.
Mahalanobis Distance: The Mahalanobis distance (MD) is the distance to the center of the training data. The larger the MD, the less trustworthy the prediction.
Mahalanobis Distance p-value: The p-value gives the fraction of training data with an MD greater than or equal to the one for the given sample, assuming normally distributed data. The smaller the p-value, the less trustworthy the prediciton. For highly non-normal X properties (e.g., fingerprints), the MD p-value is wildly inaccurate.

Structural Similar Compounds

Name	Ochratoxin a	Amsacrine	Acemetacin
Structure	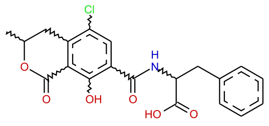	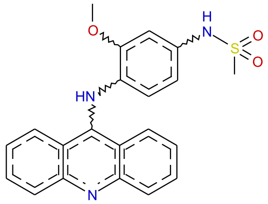	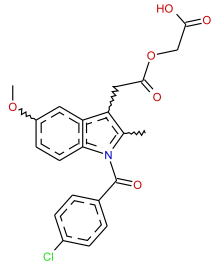
Actual Endpoint	Toxic	Toxic	Non-Toxic
Predicted Endpoint	Toxic	Toxic	Non-Toxic
Distance	0.644	0.669	0.675
Reference	Toxicol Appl Pharmacol 37(2):331-8; 1976	Fundam Appl Toxicol 7(2):214-20; 1986	Oyo Yakuri 22(6):777-786; 1981

Model Applicability

Unknown features are fingerprint features in the query molecule, but not found in the training set.

1. All properties and OPS components are within expected ranges.

Feature Contribution

Top features for positive contribution

Fingerprint	Bit/Smiles	Feature Structure	Score	Toxic in training set
SCFP_6	282594097	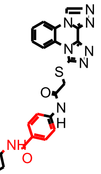 [*]NC(=O)[c]1:[cH]:[cH]:[*]:[cH]:[cH]:1	0.441	3 out of 3

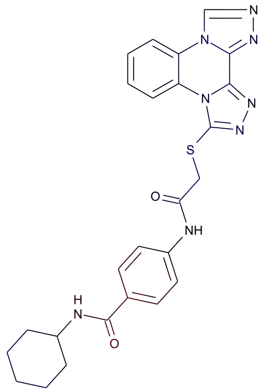

$C_{25}H_{24}N_8O_2S$
Molecular Weight: 500.57546
ALogP: 3.952
Rotatable Bonds: 6
Acceptors: 7
Donors: 2

Model Prediction

Prediction: Non-Toxic
Probability: 0.429
Enrichment: 0.816
Bayesian Score: -3.417
Mahalanobis Distance: 9.542
Mahalanobis Distance p-value: 0.0621

Prediction: Positive if the Bayesian score is above the estimated best cutoff value from minimizing the false positive and false negative rate.
Probability: The esimated probability that the sample is in the positive category. This assumes that the Bayesian score follows a normal distribution and is different from the prediction using a cutoff.
Enrichment: An estimate of enrichment, that is, the increased likelihood (versus random) of this sample being in the category.
Bayesian Score: The standard Laplacian-modified Bayesian score.
Mahalanobis Distance: The Mahalanobis distance (MD) is the distance to the center of the training data. The larger the MD, the less trustworthy the prediction.
Mahalanobis Distance p-value: The p-value gives the fraction of training data with an MD greater than or equal to the one for the given sample, assuming normally distributed data. The smaller the p-value, the less trustworthy the prediciton. For highly non-normal X properties (e.g., fingerprints), the MD p-value is wildly inaccurate.

Structural Similar Compounds

Name	Ochratoxin a	Amsacrine	Acemetacin
Structure	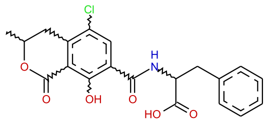	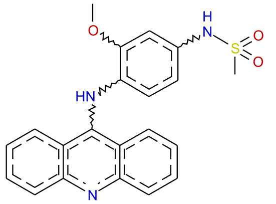	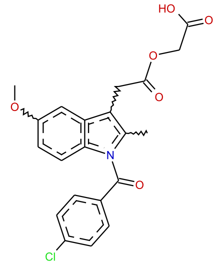
Actual Endpoint	Toxic	Toxic	Non-Toxic
Predicted Endpoint	Toxic	Toxic	Non-Toxic
Distance	0.662	0.672	0.672
Reference	Toxicol Appl Pharmacol 37(2):331-8; 1976	Fundam Appl Toxicol 7(2):214-20; 1986	Oyo Yakuri 22(6):777-786; 1981

Model Applicability

Unknown features are fingerprint features in the query molecule, but not found in the training set.

1. All properties and OPS components are within expected ranges.

Feature Contribution

Top features for positive contribution				
Fingerprint	Bit/Smiles	Feature Structure	Score	Toxic in training set
SCFP_6	282594097	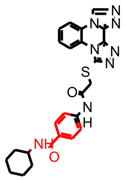 [*]NC(=O)[c]1:[cH]:[cH]:[*]:[cH]:[cH]:1	0.441	3 out of 3

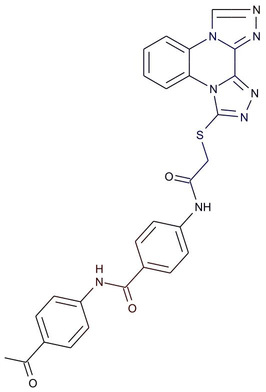

$C_{27}H_{20}N_8O_3S$

Molecular Weight: 536.56449

ALogP: 3.41

Rotatable Bonds: 7

Acceptors: 8

Donors: 2

Structural Similar Compounds

Name	Reserpate	Beclomethasone Dipropionate	Acemetacin
Structure	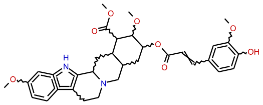	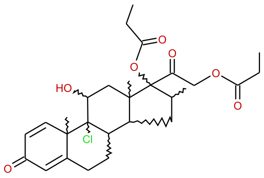	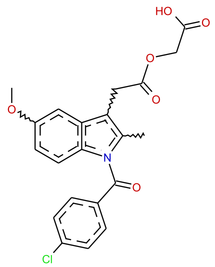
Actual Endpoint	Toxic	Toxic	Non-Toxic
Predicted Endpoint	Toxic	Toxic	Non-Toxic
Distance	0.706	0.715	0.730
Reference	Oyo Yakuri 18:105-124; 1979	Oyo Yakuri 18(6):1021-1038; 1979	Oyo Yakuri 22(6):777-786; 1981

Model Prediction

Prediction: Non-Toxic

Probability: 0.437

Enrichment: 0.831

Bayesian Score: -3.169

Mahalanobis Distance: 8.935

Mahalanobis Distance p-value: 0.197

Prediction: Positive if the Bayesian score is above the estimated best cutoff value from minimizing the false positive and false negative rate.
Probability: The esimated probability that the sample is in the positive category. This assumes that the Bayesian score follows a normal distribution and is different from the prediction using a cutoff.
Enrichment: An estimate of enrichment, that is, the increased likelihood (versus random) of this sample being in the category.
Bayesian Score: The standard Laplacian-modified Bayesian score.
Mahalanobis Distance: The Mahalanobis distance (MD) is the distance to the center of the training data. The larger the MD, the less trustworthy the prediction.
Mahalanobis Distance p-value: The p-value gives the fraction of training data with an MD greater than or equal to the one for the given sample, assuming normally distributed data. The smaller the p-value, the less trustworthy the prediciton. For highly non-normal X properties (e.g., fingerprints), the MD p-value is wildly inaccurate.

Model Applicability

Unknown features are fingerprint features in the query molecule, but not found in the training set.

1. All properties and OPS components are within expected ranges.

Feature Contribution

Top features for positive contribution

Fingerprint	Bit/Smiles	Feature Structure	Score	Toxic in training set
SCFP_6	282594097	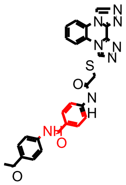 [*]NC(=O)[c]1:[cH]:[cH]:[*]:[cH]:[cH]:1	0.441	3 out of 3

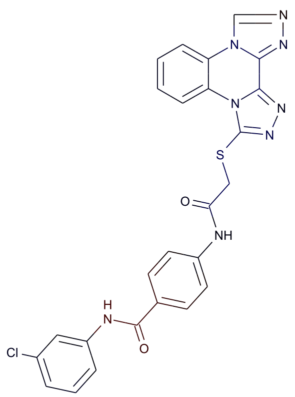

$C_{25}H_{17}ClN_8O_2S$
Molecular Weight: 528.97287
ALogP: 4.334
Rotatable Bonds: 6
Acceptors: 7
Donors: 2

Structural Similar Compounds

Name	Estramustine Phosphate Disodium (Free acid form)	Acemetacin	Ochratoxin a
Structure	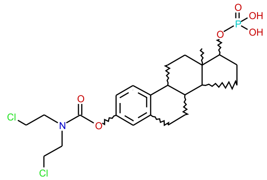	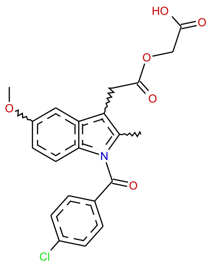	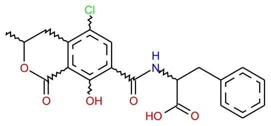
Actual Endpoint	Non-Toxic	Non-Toxic	Toxic
Predicted Endpoint	Non-Toxic	Non-Toxic	Toxic
Distance	0.667	0.673	0.693
Reference	Oyo Yakuri 20(6):1219-1236; 1980	Oyo Yakuri 22(6):777-786; 1981	Toxicol Appl Pharmacol 37(2):331-8; 1976

Model Prediction

Prediction: Non-Toxic
Probability: 0.423
Enrichment: 0.805
Bayesian Score: -3.611
Mahalanobis Distance: 8.644
Mahalanobis Distance p-value: 0.304

Prediction: Positive if the Bayesian score is above the estimated best cutoff value from minimizing the false positive and false negative rate.
Probability: The esimated probability that the sample is in the positive category. This assumes that the Bayesian score follows a normal distribution and is different from the prediction using a cutoff.
Enrichment: An estimate of enrichment, that is, the increased likelihood (versus random) of this sample being in the category.
Bayesian Score: The standard Laplacian-modified Bayesian score.
Mahalanobis Distance: The Mahalanobis distance (MD) is the distance to the center of the training data. The larger the MD, the less trustworthy the prediction.
Mahalanobis Distance p-value: The p-value gives the fraction of training data with an MD greater than or equal to the one for the given sample, assuming normally distributed data. The smaller the p-value, the less trustworthy the prediciton. For highly non-normal X properties (e.g., fingerprints), the MD p-value is wildly inaccurate.

Model Applicability

Unknown features are fingerprint features in the query molecule, but not found in the training set.

1. All properties and OPS components are within expected ranges.

Feature Contribution

Top features for positive contribution

Fingerprint	Bit/Smiles	Feature Structure	Score	Toxic in training set
SCFP_6	282594097	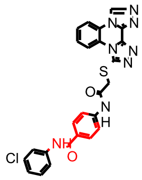 [*]NC(=O)[c]1:[cH]:[cH]:[*]:[cH]:[cH]:1	0.441	3 out of 3

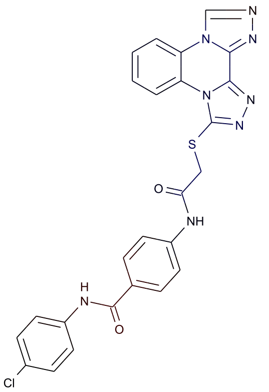

$C_{25}H_{17}ClN_8O_2S$
Molecular Weight: 528.97287
ALogP: 4.334
Rotatable Bonds: 6
Acceptors: 7
Donors: 2

Model Prediction

Prediction: Non-Toxic
Probability: 0.438
Enrichment: 0.832
Bayesian Score: -3.146
Mahalanobis Distance: 8.644
Mahalanobis Distance p-value: 0.304

Prediction: Positive if the Bayesian score is above the estimated best cutoff value from minimizing the false positive and false negative rate.
Probability: The esimated probability that the sample is in the positive category. This assumes that the Bayesian score follows a normal distribution and is different from the prediction using a cutoff.
Enrichment: An estimate of enrichment, that is, the increased likelihood (versus random) of this sample being in the category.
Bayesian Score: The standard Laplacian-modified Bayesian score.
Mahalanobis Distance: The Mahalanobis distance (MD) is the distance to the center of the training data. The larger the MD, the less trustworthy the prediction.
Mahalanobis Distance p-value: The p-value gives the fraction of training data with an MD greater than or equal to the one for the given sample, assuming normally distributed data. The smaller the p-value, the less trustworthy the prediciton. For highly non-normal X properties (e.g., fingerprints), the MD p-value is wildly inaccurate.

Structural Similar Compounds

Name	Estramustine Phosphate Disodium (Free acid form)	Acemetacin	Ochratoxin a
Structure	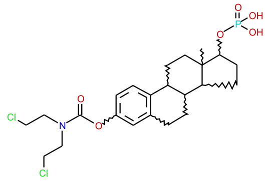	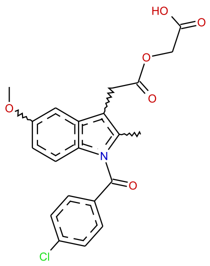	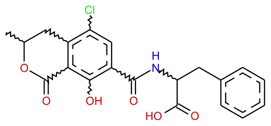
Actual Endpoint	Non-Toxic	Non-Toxic	Toxic
Predicted Endpoint	Non-Toxic	Non-Toxic	Toxic
Distance	0.667	0.667	0.693
Reference	Oyo Yakuri 20(6):1219-1236; 1980	Oyo Yakuri 22(6):777-786; 1981	Toxicol Appl Pharmacol 37(2):331-8; 1976

Model Applicability

Unknown features are fingerprint features in the query molecule, but not found in the training set.

1. All properties and OPS components are within expected ranges.

Feature Contribution

Top features for positive contribution

Fingerprint	Bit/Smiles	Feature Structure	Score	Toxic in training set
SCFP_6	282594097	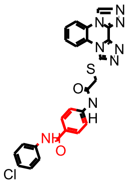 [*]NC(=O)[c]1:[cH]:[cH]:[*]:[cH]:[cH]:1	0.441	3 out of 3

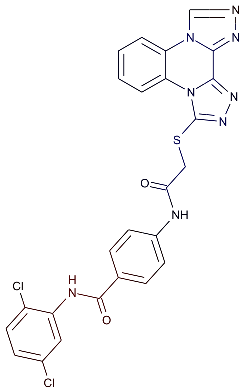

$C_{25}H_{16}Cl_2N_8O_2S$
Molecular Weight: 563.41793
ALogP: 4.999
Rotatable Bonds: 6
Acceptors: 7
Donors: 2

Model Prediction

Prediction: Non-Toxic
Probability: 0.490
Enrichment: 0.932
Bayesian Score: -1.566
Mahalanobis Distance: 8.725
Mahalanobis Distance p-value: 0.272

Prediction: Positive if the Bayesian score is above the estimated best cutoff value from minimizing the false positive and false negative rate.
Probability: The esimated probability that the sample is in the positive category. This assumes that the Bayesian score follows a normal distribution and is different from the prediction using a cutoff.
Enrichment: An estimate of enrichment, that is, the increased likelihood (versus random) of this sample being in the category.
Bayesian Score: The standard Laplacian-modified Bayesian score.
Mahalanobis Distance: The Mahalanobis distance (MD) is the distance to the center of the training data. The larger the MD, the less trustworthy the prediction.
Mahalanobis Distance p-value: The p-value gives the fraction of training data with an MD greater than or equal to the one for the given sample, assuming normally distributed data. The smaller the p-value, the less trustworthy the prediciton. For highly non-normal X properties (e.g., fingerprints), the MD p-value is wildly inaccurate.

Structural Similar Compounds

Name	Estramustine Phosphate Disodium (Free acid form)	Bromocriptine	Acemetacin
Structure	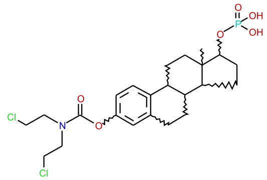	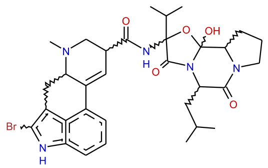	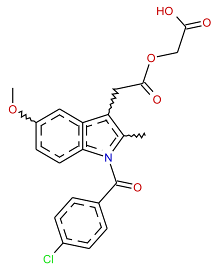
Actual Endpoint	Non-Toxic	Non-Toxic	Non-Toxic
Predicted Endpoint	Non-Toxic	Non-Toxic	Non-Toxic
Distance	0.647	0.694	0.714
Reference	Oyo Yakuri 20(6):1219-1236; 1980	Toxicol Lett 50:189-194; 1990	Oyo Yakuri 22(6):777-786; 1981

Model Applicability

Unknown features are fingerprint features in the query molecule, but not found in the training set.

1. All properties and OPS components are within expected ranges.

Feature Contribution

Top features for positive contribution				
**Fingerprint**	**Bit/Smiles**	**Feature Structure**	**Score**	**Toxic in training set**
SCFP_6	282594097	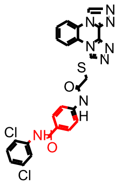 [*]NC(=O)[c]1:[cH]:[cH]:[*]:[cH]:[cH]:1	0.441	3 out of 3

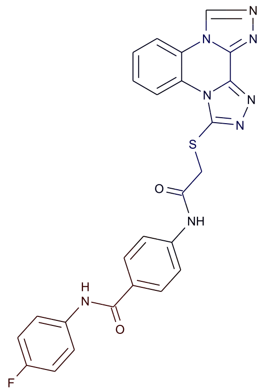

$C_{25}H_{17}FN_8O_2S$
Molecular Weight: 512.51828
ALogP: 3.875
Rotatable Bonds: 6
Acceptors: 7
Donors: 2

Model Prediction

Prediction: Non-Toxic
Probability: 0.483
Enrichment: 0.919
Bayesian Score: -1.768
Mahalanobis Distance: 8.485
Mahalanobis Distance p-value: 0.374

Prediction: Positive if the Bayesian score is above the estimated best cutoff value from minimizing the false positive and false negative rate.
Probability: The esimated probability that the sample is in the positive category. This assumes that the Bayesian score follows a normal distribution and is different from the prediction using a cutoff.
Enrichment: An estimate of enrichment, that is, the increased likelihood (versus random) of this sample being in the category.
Bayesian Score: The standard Laplacian-modified Bayesian score.
Mahalanobis Distance: The Mahalanobis distance (MD) is the distance to the center of the training data. The larger the MD, the less trustworthy the prediction.
Mahalanobis Distance p-value: The p-value gives the fraction of training data with an MD greater than or equal to the one for the given sample, assuming normally distributed data. The smaller the p-value, the less trustworthy the prediciton. For highly non-normal X properties (e.g., fingerprints), the MD p-value is wildly inaccurate.

Structural Similar Compounds

Name	Ochratoxin a	Acemetacin	Amsacrine
Structure	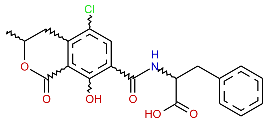	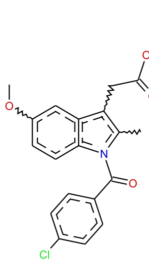	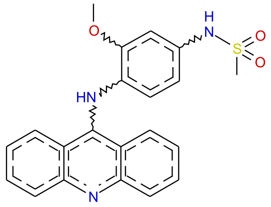
Actual Endpoint	Toxic	Non-Toxic	Toxic
Predicted Endpoint	Toxic	Non-Toxic	Toxic
Distance	0.678	0.681	0.682
Reference	Toxicol Appl Pharmacol 37(2):331-8; 1976	Oyo Yakuri 22(6):777-786; 1981	Fundam Appl Toxicol 7(2):214-20; 1986

Model Applicability

Unknown features are fingerprint features in the query molecule, but not found in the training set.

1. All properties and OPS components are within expected ranges.

Feature Contribution

Top features for positive contribution				
**Fingerprint**	**Bit/Smiles**	**Feature Structure**	**Score**	**Toxic in training set**
SCFP_6	282594097	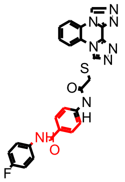 [*]NC(=O)[c]1:[cH]:[cH]:[*]:[cH]:[cH]:1	0.441	3 out of 3

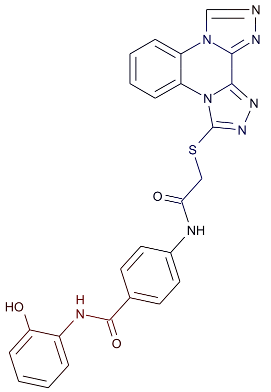

$C_{25}H_{18}N_8O_3S$

Molecular Weight: 510.52722

ALogP: 3.428

Rotatable Bonds: 6

Acceptors: 8

Donors: 3

Model Prediction

Prediction: Non-Toxic

Probability: 0.481

Enrichment: 0.915

Bayesian Score: -1.835

Mahalanobis Distance: 8.433

Mahalanobis Distance p-value: 0.398

Prediction: Positive if the Bayesian score is above the estimated best cutoff value from minimizing the false positive and false negative rate.
Probability: The esimated probability that the sample is in the positive category. This assumes that the Bayesian score follows a normal distribution and is different from the prediction using a cutoff.
Enrichment: An estimate of enrichment, that is, the increased likelihood (versus random) of this sample being in the category.
Bayesian Score: The standard Laplacian-modified Bayesian score.
Mahalanobis Distance: The Mahalanobis distance (MD) is the distance to the center of the training data. The larger the MD, the less trustworthy the prediction.
Mahalanobis Distance p-value: The p-value gives the fraction of training data with an MD greater than or equal to the one for the given sample, assuming normally distributed data. The smaller the p-value, the less trustworthy the prediciton. For highly non-normal X properties (e.g., fingerprints), the MD p-value is wildly inaccurate.

Structural Similar Compounds

Name	Ochratoxin a	Lenampicillin .HCl (Free base form)	Amsacrine
Structure	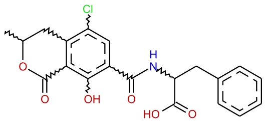	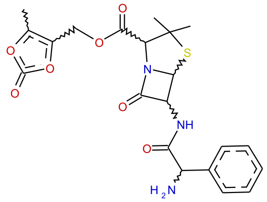	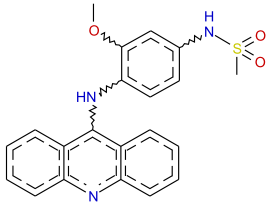
Actual Endpoint	Toxic	Non-Toxic	Toxic
Predicted Endpoint	Toxic	Non-Toxic	Toxic
Distance	0.661	0.754	0.768
Reference	Toxicol Appl Pharmacol 37(2):331-8; 1976	Chemotherapy 32:130-145; 1984	Fundam Appl Toxicol 7(2):214-20; 1986

Model Applicability

Unknown features are fingerprint features in the query molecule, but not found in the training set.

1. All properties and OPS components are within expected ranges.

Feature Contribution

Top features for positive contribution

Fingerprint	Bit/Smiles	Feature Structure	Score	Toxic in training set
SCFP_6	282594097	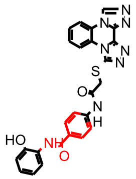 [*]NC(=O)[c]1:[cH]:[cH]:[*]:[cH]:[cH]:1	0.441	3 out of 3

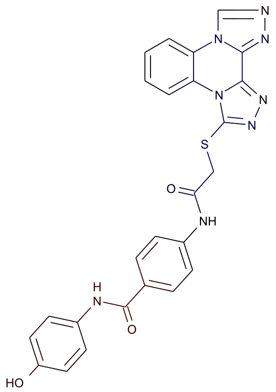

$C_{25}H_{18}N_8O_3S$
Molecular Weight: 510.52722
ALogP: 3.428
Rotatable Bonds: 6
Acceptors: 8
Donors: 3

Model Prediction

Prediction: Non-Toxic
Probability: 0.467
Enrichment: 0.888
Bayesian Score: -2.253
Mahalanobis Distance: 8.433
Mahalanobis Distance p-value: 0.398

Prediction: Positive if the Bayesian score is above the estimated best cutoff value from minimizing the false positive and false negative rate.
Probability: The esimated probability that the sample is in the positive category. This assumes that the Bayesian score follows a normal distribution and is different from the prediction using a cutoff.
Enrichment: An estimate of enrichment, that is, the increased likelihood (versus random) of this sample being in the category.
Bayesian Score: The standard Laplacian-modified Bayesian score.
Mahalanobis Distance: The Mahalanobis distance (MD) is the distance to the center of the training data. The larger the MD, the less trustworthy the prediction.
Mahalanobis Distance p-value: The p-value gives the fraction of training data with an MD greater than or equal to the one for the given sample, assuming normally distributed data. The smaller the p-value, the less trustworthy the prediciton. For highly non-normal X properties (e.g., fingerprints), the MD p-value is wildly inaccurate.

Structural Similar Compounds

Name	Ochratoxin a	Lenampicillin .HCl (Free base form)	Amsacrine
Structure	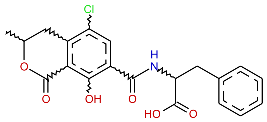	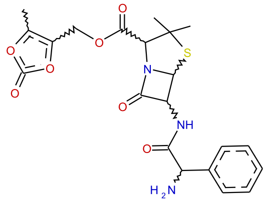	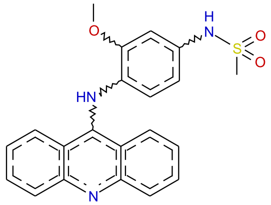
Actual Endpoint	Toxic	Non-Toxic	Toxic
Predicted Endpoint	Toxic	Non-Toxic	Toxic
Distance	0.660	0.754	0.767
Reference	Toxicol Appl Pharmacol 37(2):331-8; 1976	Chemotherapy 32:130-145; 1984	Fundam Appl Toxicol 7(2):214-20; 1986

Model Applicability

Unknown features are fingerprint features in the query molecule, but not found in the training set.

1. All properties and OPS components are within expected ranges.

Feature Contribution

Top features for positive contribution				
**Fingerprint**	**Bit/Smiles**	**Feature Structure**	**Score**	**Toxic in training set**
SCFP_6	282594097	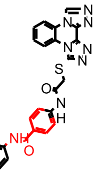 [*]NC(=O)[c]1:[cH]:[cH]:[*]:[cH]:[cH]:1	0.441	3 out of 3

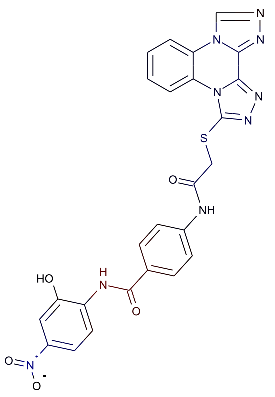

$C_{25}H_{17}N_9O_5S$
Molecular Weight: 555.52477
ALogP: 3.322
Rotatable Bonds: 7
Acceptors: 10
Donors: 3

Model Prediction

Prediction: Non-Toxic
Probability: 0.388
Enrichment: 0.738
Bayesian Score: -4.830
Mahalanobis Distance: 11.020
Mahalanobis Distance p-value: 0.00103

Prediction: Positive if the Bayesian score is above the estimated best cutoff value from minimizing the false positive and false negative rate.
Probability: The esimated probability that the sample is in the positive category. This assumes that the Bayesian score follows a normal distribution and is different from the prediction using a cutoff.
Enrichment: An estimate of enrichment, that is, the increased likelihood (versus random) of this sample being in the category.
Bayesian Score: The standard Laplacian-modified Bayesian score.
Mahalanobis Distance: The Mahalanobis distance (MD) is the distance to the center of the training data. The larger the MD, the less trustworthy the prediction.
Mahalanobis Distance p-value: The p-value gives the fraction of training data with an MD greater than or equal to the one for the given sample, assuming normally distributed data. The smaller the p-value, the less trustworthy the prediciton. For highly non-normal X properties (e.g., fingerprints), the MD p-value is wildly inaccurate.

Structural Similar Compounds

Name	Cyclic AMP Bucladesine	Tartrazine	Lenampicillin .HCl (Free base form)
Structure	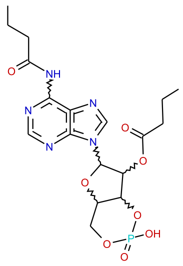	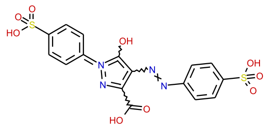	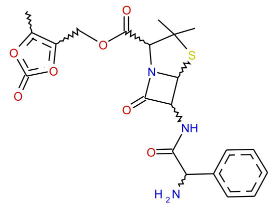
Actual Endpoint	Non-Toxic	Non-Toxic	Non-Toxic
Predicted Endpoint	Non-Toxic	Non-Toxic	Non-Toxic
Distance	0.750	0.761	0.768
Reference	Oyo Yakuri 27(3):585-597; 1984	Oyo Yakuri 399-404; 1982	Chemotherapy 32:130-145; 1984

Model Applicability

Unknown features are fingerprint features in the query molecule, but not found in the training set.

1. All properties and OPS components are within expected ranges.

Feature Contribution

Top features for positive contribution

Fingerprint	Bit/Smiles	Feature Structure	Score	Toxic in training set
SCFP_6	282594097	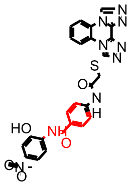 [*]NC(=O)[c]1:[cH]:[cH]:[*]:[cH]:[cH]:1	0.441	3 out of 3

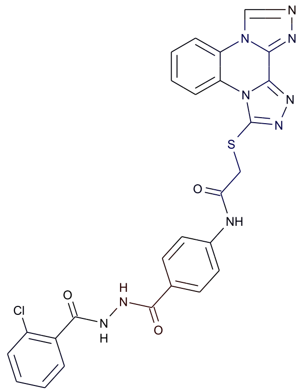

$C_{26}H_{18}ClN_9O_3S$
Molecular Weight: 571.99761
ALogP: 3.591
Rotatable Bonds: 7
Acceptors: 8
Donors: 3

Model Prediction

Prediction: Non-Toxic
Probability: 0.425
Enrichment: 0.808
Bayesian Score: -3.570
Mahalanobis Distance: 9.297
Mahalanobis Distance p-value: 0.103

Prediction: Positive if the Bayesian score is above the estimated best cutoff value from minimizing the false positive and false negative rate.
Probability: The esimated probability that the sample is in the positive category. This assumes that the Bayesian score follows a normal distribution and is different from the prediction using a cutoff.
Enrichment: An estimate of enrichment, that is, the increased likelihood (versus random) of this sample being in the category.
Bayesian Score: The standard Laplacian-modified Bayesian score.
Mahalanobis Distance: The Mahalanobis distance (MD) is the distance to the center of the training data. The larger the MD, the less trustworthy the prediction.
Mahalanobis Distance p-value: The p-value gives the fraction of training data with an MD greater than or equal to the one for the given sample, assuming normally distributed data. The smaller the p-value, the less trustworthy the prediciton. For highly non-normal X properties (e.g., fingerprints), the MD p-value is wildly inaccurate.

Structural Similar Compounds

Name	Ochratoxin a	Reserpate	Bromocriptine
Structure	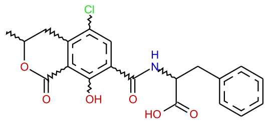	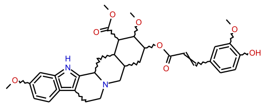	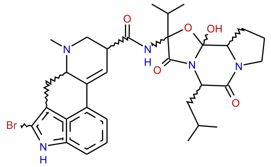
Actual Endpoint	Toxic	Toxic	Non-Toxic
Predicted Endpoint	Toxic	Toxic	Non-Toxic
Distance	0.736	0.740	0.742
Reference	Toxicol Appl Pharmacol 37(2):331-8; 1976	Oyo Yakuri 18:105-124; 1979	Toxicol Lett 50:189-194; 1990

Model Applicability

Unknown features are fingerprint features in the query molecule, but not found in the training set.

1. All properties and OPS components are within expected ranges.

Feature Contribution

Top features for positive contribution				
**Fingerprint**	**Bit/Smiles**	**Feature Structure**	**Score**	**Toxic in training set**
SCFP_6	282594097	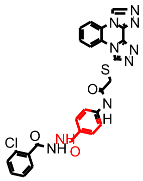 [*]NC(=O)[c]1:[cH]:[cH]:[*]:[cH]:[cH]:1	0.441	3 out of 3

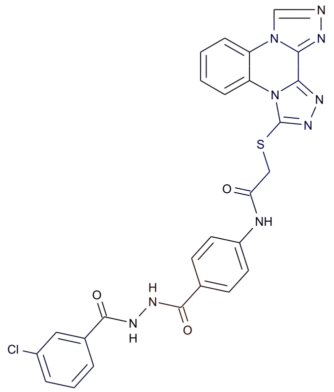

$C_{26}H_{18}ClN_9O_3S$
Molecular Weight: 571.99761
ALogP: 3.591
Rotatable Bonds: 7
Acceptors: 8
Donors: 3

Model Prediction

Prediction: Non-Toxic
Probability: 0.415
Enrichment: 0.789
Bayesian Score: -3.887
Mahalanobis Distance: 8.974
Mahalanobis Distance p-value: 0.185

Prediction: Positive if the Bayesian score is above the estimated best cutoff value from minimizing the false positive and false negative rate.
Probability: The esimated probability that the sample is in the positive category. This assumes that the Bayesian score follows a normal distribution and is different from the prediction using a cutoff.
Enrichment: An estimate of enrichment, that is, the increased likelihood (versus random) of this sample being in the category.
Bayesian Score: The standard Laplacian-modified Bayesian score.
Mahalanobis Distance: The Mahalanobis distance (MD) is the distance to the center of the training data. The larger the MD, the less trustworthy the prediction.
Mahalanobis Distance p-value: The p-value gives the fraction of training data with an MD greater than or equal to the one for the given sample, assuming normally distributed data. The smaller the p-value, the less trustworthy the prediciton. For highly non-normal X properties (e.g., fingerprints), the MD p-value is wildly inaccurate.

Structural Similar Compounds

Name	Ochratoxin a	Reserpate	Bromocriptine
Structure	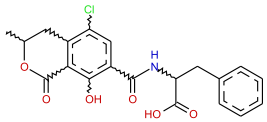	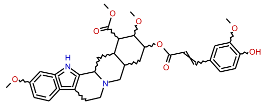	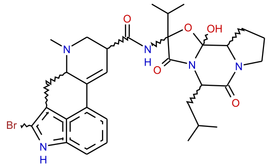
Actual Endpoint	Toxic	Toxic	Non-Toxic
Predicted Endpoint	Toxic	Toxic	Non-Toxic
Distance	0.731	0.740	0.742
Reference	Toxicol Appl Pharmacol 37(2):331-8; 1976	Oyo Yakuri 18:105-124; 1979	Toxicol Lett 50:189-194; 1990

Model Applicability

Unknown features are fingerprint features in the query molecule, but not found in the training set.

1. All properties and OPS components are within expected ranges.

Feature Contribution

Top features for positive contribution

Fingerprint	Bit/Smiles	Feature Structure	Score	Toxic in training set
SCFP_6	282594097	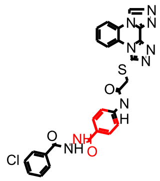 [*]NC(=O)[c]1:[cH]:[cH]:[*]:[cH]:[cH]:1	0.441	3 out of 3

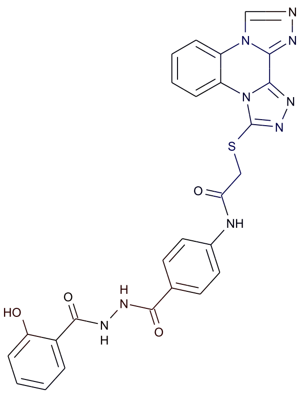

$C_{26}H_{19}N_9O_4S$
Molecular Weight: 553.55195
ALogP: 2.685
Rotatable Bonds: 7
Acceptors: 9
Donors: 4

Model Prediction

Prediction: Non-Toxic
Probability: 0.457
Enrichment: 0.868
Bayesian Score: -2.562
Mahalanobis Distance: 8.677
Mahalanobis Distance p-value: 0.291

Prediction: Positive if the Bayesian score is above the estimated best cutoff value from minimizing the false positive and false negative rate.
Probability: The esimated probability that the sample is in the positive category. This assumes that the Bayesian score follows a normal distribution and is different from the prediction using a cutoff.
Enrichment: An estimate of enrichment, that is, the increased likelihood (versus random) of this sample being in the category.
Bayesian Score: The standard Laplacian-modified Bayesian score.
Mahalanobis Distance: The Mahalanobis distance (MD) is the distance to the center of the training data. The larger the MD, the less trustworthy the prediction.
Mahalanobis Distance p-value: The p-value gives the fraction of training data with an MD greater than or equal to the one for the given sample, assuming normally distributed data. The smaller the p-value, the less trustworthy the prediciton. For highly non-normal X properties (e.g., fingerprints), the MD p-value is wildly inaccurate.

Structural Similar Compounds

Name	Lenampicillin .HCl (Free base form)	Ochratoxin a	Tartrazine
Structure	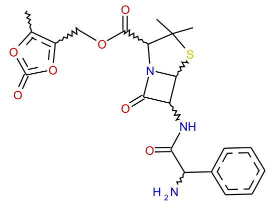	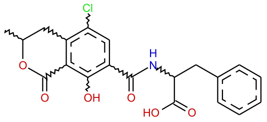	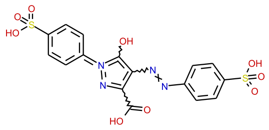
Actual Endpoint	Non-Toxic	Toxic	Non-Toxic
Predicted Endpoint	Non-Toxic	Toxic	Non-Toxic
Distance	0.793	0.796	0.796
Reference	Chemotherapy 32:130-145; 1984	Toxicol Appl Pharmacol 37(2):331-8; 1976	Oyo Yakuri 399-404; 1982

Model Applicability

Unknown features are fingerprint features in the query molecule, but not found in the training set.

1. All properties and OPS components are within expected ranges.

Feature Contribution

Top features for positive contribution

Fingerprint	Bit/Smiles	Feature Structure	Score	Toxic in training set
SCFP_6	282594097	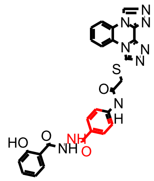 [*]NC(=O)[c]1:[cH]:[cH]:[*]:[cH]:[cH]:1	0.441	3 out of 3

Sorafenib

TOPKAT_Developmental_Toxicity_Potential

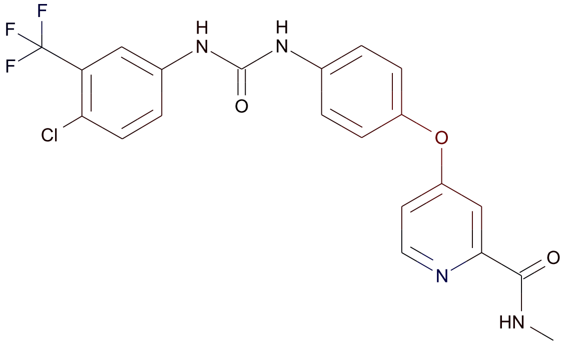

$C_{21}H_{16}ClF_3N_4O_3$
Molecular Weight: 464.82494
ALogP: 4.175
Rotatable Bonds: 6
Acceptors: 4
Donors: 3

Structural Similar Compounds

Name	Chenodiol	Amsacrine	Ochratoxin a
Structure	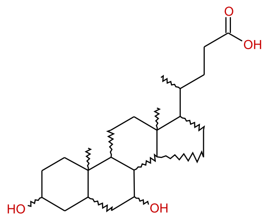	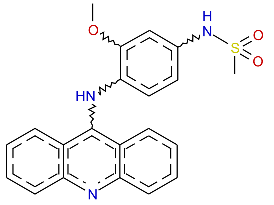	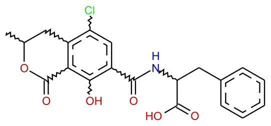
Actual Endpoint	Toxic	Toxic	Toxic
Predicted Endpoint	Toxic	Toxic	Toxic
Distance	0.631	0.637	0.644
Reference	Arch Int Pharm 246:149-158; 1980	Fundam Appl Toxicol 7(2):214-20; 1986	Toxicol Appl Pharmacol 37(2):331-8; 1976

Model Applicability

Unknown features are fingerprint features in the query molecule, but not found in the training set.

1. All properties and OPS components are within expected ranges.

Model Prediction

Prediction: Toxic

Probability: 0.592

Enrichment: 1.125

Bayesian Score: 1.149

Mahalanobis Distance: 12.645

Mahalanobis Distance p-value: 2.07e-006

Prediction: Positive if the Bayesian score is above the estimated best cutoff value from minimizing the false positive and false negative rate.
Probability: The esimated probability that the sample is in the positive category. This assumes that the Bayesian score follows a normal distribution and is different from the prediction using a cutoff.
Enrichment: An estimate of enrichment, that is, the increased likelihood (versus random) of this sample being in the category.
Bayesian Score: The standard Laplacian-modified Bayesian score.
Mahalanobis Distance: The Mahalanobis distance (MD) is the distance to the center of the training data. The larger the MD, the less trustworthy the prediction.
Mahalanobis Distance p-value: The p-value gives the fraction of training data with an MD greater than or equal to the one for the given sample, assuming normally distributed data. The smaller the p-value, the less trustworthy the prediciton. For highly non-normal X properties (e.g., fingerprints), the MD p-value is wildly inaccurate.

Feature Contribution

Top features for positive contribution				
**Fingerprint**	**Bit/Smiles**	**Feature Structure**	**Score**	**Toxic in training set**
SCFP_6	1559190850	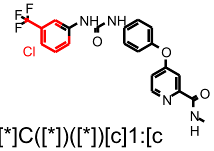 [*]C([*])([*])[c]1:[cH]:[*]:[cH]:[cH]:[c]:1Cl	0.441	3 out of 3

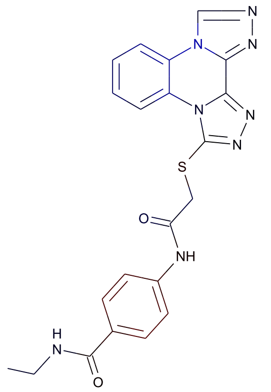

$C_{21}H_{18}N_8O_2S$
Molecular Weight: 446.48502
ALogP: 2.442
Rotatable Bonds: 6
Acceptors: 7
Donors: 2

Model Prediction

Prediction: Non-Carcinogen
Probability: 0.206
Enrichment: 0.643
Bayesian Score: -5.320
Mahalanobis Distance: 11.035
Mahalanobis Distance p-value: 0.0975

Prediction: Positive if the Bayesian score is above the estimated best cutoff value from minimizing the false positive and false negative rate.
Probability: The esimated probability that the sample is in the positive category. This assumes that the Bayesian score follows a normal distribution and is different from the prediction using a cutoff.
Enrichment: An estimate of enrichment, that is, the increased likelihood (versus random) of this sample being in the category.
Bayesian Score: The standard Laplacian-modified Bayesian score.
Mahalanobis Distance: The Mahalanobis distance (MD) is the distance to the center of the training data. The larger the MD, the less trustworthy the prediction.
Mahalanobis Distance p-value: The p-value gives the fraction of training data with an MD greater than or equal to the one for the given sample, assuming normally distributed data. The smaller the p-value, the less trustworthy the prediciton. For highly non-normal X properties (e.g., fingerprints), the MD p-value is wildly inaccurate.

Structural Similar Compounds

Name	Nedocromil	Glipizide	Bicalutamide
Structure	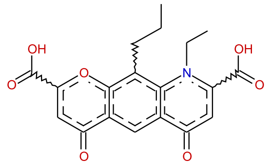	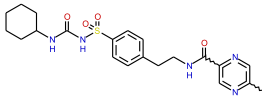	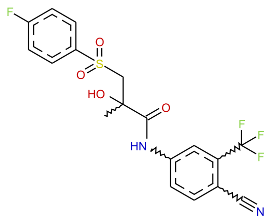
Actual Endpoint	Non-Carcinogen	Non-Carcinogen	Non-Carcinogen
Predicted Endpoint	Non-Carcinogen	Non-Carcinogen	Non-Carcinogen
Distance	0.636	0.642	0.644
Reference	US FDA (Centre for Drug Eval.& Res./Off. Testing & Res.) Sept. 1997	US FDA (Centre for Drug Eval.& Res./Off. Testing & Res.) Sept. 1997	US FDA (Centre for Drug Eval.& Res./Off. Testing & Res.) Sept. 1997

Model Applicability

Unknown features are fingerprint features in the query molecule, but not found in the training set.

1. All properties and OPS components are within expected ranges.
2. Unknown ECFP_2 feature: -955816473: [*]SCC(=[*])[*]
3. Unknown ECFP_2 feature: 77911192: [*][c]1:[*]:[*]:[c](:[*]):n:1:[c](:[*]):[*]
4. Unknown ECFP_2 feature: 1986731747: [*]S[c]1:n:[*]:[*]:n:1:[*]
5. Unknown ECFP_2 feature: 1427820655: [*]CS[c](:[*]):[*]

Feature Contribution

Top features for positive contribution

Fingerprint	Bit/Smiles	Feature Structure	Score	Carcinogen in training set
ECFP_6	738938915	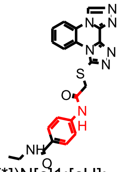 [*]C(=[*])N[c]1:[cH]:[cH]:[*]:[cH]:[cH]:1	0.617	2 out of 2

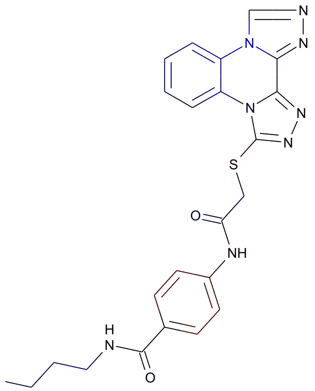

$C_{23}H_{22}N_8O_2S$

Molecular Weight: 474.53818

ALogP: 3.422

Rotatable Bonds: 8

Acceptors: 7

Donors: 2

Model Prediction

Prediction: Non-Carcinogen

Probability: 0.205

Enrichment: 0.639

Bayesian Score: -6.222

Mahalanobis Distance: 12.843

Mahalanobis Distance p-value: 0.000901

Prediction: Positive if the Bayesian score is above the estimated best cutoff value from minimizing the false positive and false negative rate.
Probability: The esimated probability that the sample is in the positive category. This assumes that the Bayesian score follows a normal distribution and is different from the prediction using a cutoff.
Enrichment: An estimate of enrichment, that is, the increased likelihood (versus random) of this sample being in the category.
Bayesian Score: The standard Laplacian-modified Bayesian score.
Mahalanobis Distance: The Mahalanobis distance (MD) is the distance to the center of the training data. The larger the MD, the less trustworthy the prediction.
Mahalanobis Distance p-value: The p-value gives the fraction of training data with an MD greater than or equal to the one for the given sample, assuming normally distributed data. The smaller the p-value, the less trustworthy the prediciton. For highly non-normal X properties (e.g., fingerprints), the MD p-value is wildly inaccurate.

Structural Similar Compounds

Name	Cisapride	Glipizide	Bicalutamide
Structure	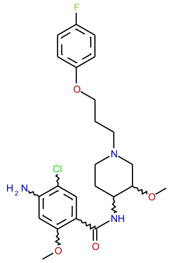	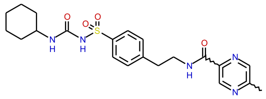	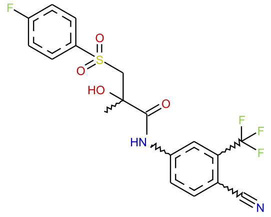
Actual Endpoint	Non-Carcinogen	Non-Carcinogen	Non-Carcinogen
Predicted Endpoint	Non-Carcinogen	Non-Carcinogen	Non-Carcinogen
Distance	0.674	0.682	0.686
Reference	US FDA (Centre for Drug Eval.& Res./Off. Testing & Res.) Sept. 1997	US FDA (Centre for Drug Eval.& Res./Off. Testing & Res.) Sept. 1997	US FDA (Centre for Drug Eval.& Res./Off. Testing & Res.) Sept. 1997

Model Applicability

Unknown features are fingerprint features in the query molecule, but not found in the training set.

1. All properties and OPS components are within expected ranges.
2. Unknown ECFP_2 feature: -955816473: [*]SCC(=[*])[*]
3. Unknown ECFP_2 feature: 77911192: [*][c]1:[*]:[*]:[c](:[*]):n:1:[c](:[*]):[*]
4. Unknown ECFP_2 feature: 1986731747: [*]S[c]1:n:[*]:[*]:n:1:[*]
5. Unknown ECFP_2 feature: 1427820655: [*]CS[c](:[*]):[*]

Feature Contribution

Top features for positive contribution

Fingerprint	Bit/Smiles	Feature Structure	Score	Carcinogen in training set
ECFP_6	738938915	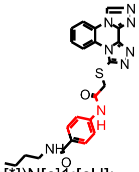 [*]C(=[*])N[c]1:[cH]:[cH]:[*]:[cH]:[cH]:1	0.617	2 out of 2

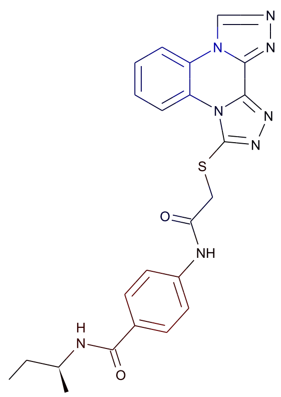

$C_{23}H_{22}N_8O_2S$
Molecular Weight: 474.53818
ALogP: 3.343
Rotatable Bonds: 7
Acceptors: 7
Donors: 2

Structural Similar Compounds

Name	Bicalutamide	Moricizine	Glipizide
Structure	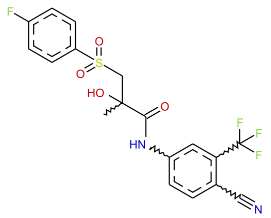	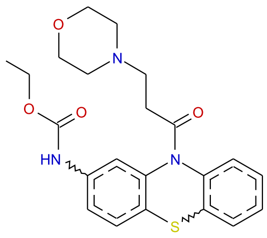	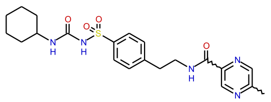
Actual Endpoint	Non-Carcinogen	Carcinogen	Non-Carcinogen
Predicted Endpoint	Non-Carcinogen	Carcinogen	Non-Carcinogen
Distance	0.658	0.673	0.679
Reference	US FDA (Centre for Drug Eval.& Res./Off. Testing & Res.) Sept. 1997	US FDA (Centre for Drug Eval.& Res./Off. Testing & Res.) Sept. 1997	US FDA (Centre for Drug Eval.& Res./Off. Testing & Res.) Sept. 1997

Model Prediction

Prediction: Non-Carcinogen
Probability: 0.214
Enrichment: 0.669
Bayesian Score: -3.645
Mahalanobis Distance: 11.445
Mahalanobis Distance p-value: 0.0418

Prediction: Positive if the Bayesian score is above the estimated best cutoff value from minimizing the false positive and false negative rate.
Probability: The esimated probability that the sample is in the positive category. This assumes that the Bayesian score follows a normal distribution and is different from the prediction using a cutoff.
Enrichment: An estimate of enrichment, that is, the increased likelihood (versus random) of this sample being in the category.
Bayesian Score: The standard Laplacian-modified Bayesian score.
Mahalanobis Distance: The Mahalanobis distance (MD) is the distance to the center of the training data. The larger the MD, the less trustworthy the prediction.
Mahalanobis Distance p-value: The p-value gives the fraction of training data with an MD greater than or equal to the one for the given sample, assuming normally distributed data. The smaller the p-value, the less trustworthy the prediciton. For highly non-normal X properties (e.g., fingerprints), the MD p-value is wildly inaccurate.

Model Applicability

Unknown features are fingerprint features in the query molecule, but not found in the training set.

1. All properties and OPS components are within expected ranges.
2. Unknown ECFP_2 feature: -955816473: [*]SCC(=[*])[*]
3. Unknown ECFP_2 feature: 77911192: [*][c]1:[*]:[*]:[c](:[*]):n:1:[c](:[*]):[*]
4. Unknown ECFP_2 feature: 1986731747: [*]S[c]1:n:[*]:[*]:n:1:[*]
5. Unknown ECFP_2 feature: 1427820655: [*]CS[c](:[*]):[*]

Feature Contribution

Top features for positive contribution

Fingerprint	Bit/Smiles	Feature Structure	Score	Carcinogen in training set
ECFP_6	738938915	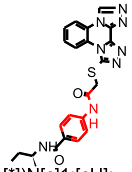 [*]C(=[*])N[c]1:[cH]:[cH]:[*]:[cH]:[cH]:1	0.617	2 out of 2

ter. butyl.cdx

TOPKAT_Mouse_Female_FDA_None_vs_Carcinogen

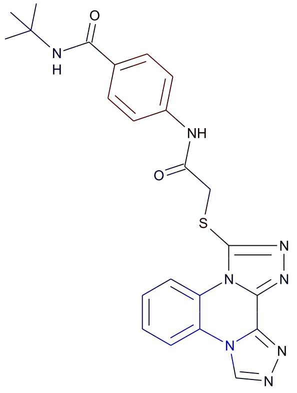

$C_{23}H_{22}N_8O_2S$

Molecular Weight: 474.53818

ALogP: 3.025

Rotatable Bonds: 6

Acceptors: 7

Donors: 2

Model Prediction

Prediction: Non-Carcinogen

Probability: 0.207

Enrichment: 0.645

Bayesian Score: -5.088

Mahalanobis Distance: 11.584

Mahalanobis Distance p-value: 0.0304

Prediction: Positive if the Bayesian score is above the estimated best cutoff value from minimizing the false positive and false negative rate.

Probability: The esimated probability that the sample is in the positive category. This assumes that the Bayesian score follows a normal distribution and is different from the prediction using a cutoff.

Enrichment: An estimate of enrichment, that is, the increased likelihood (versus random) of this sample being in the category.

Bayesian Score: The standard Laplacian-modified Bayesian score.

Mahalanobis Distance: The Mahalanobis distance (MD) is the distance to the center of the training data. The larger the MD, the less trustworthy the prediction.

Mahalanobis Distance p-value: The p-value gives the fraction of training data with an MD greater than or equal to the one for the given sample, assuming normally distributed data. The smaller the p-value, the less trustworthy the prediciton. For highly non-normal X properties (e.g., fingerprints), the MD p-value is wildly inaccurate.

Structural Similar Compounds

Name	Bicalutamide	Moricizine	Glipizide
Structure	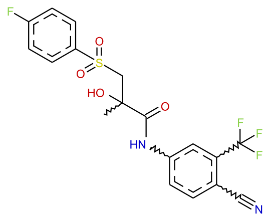	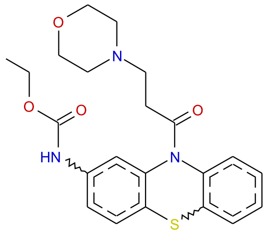	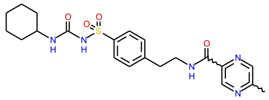
Actual Endpoint	Non-Carcinogen	Carcinogen	Non-Carcinogen
Predicted Endpoint	Non-Carcinogen	Carcinogen	Non-Carcinogen
Distance	0.634	0.661	0.671
Reference	US FDA (Centre for Drug Eval.& Res./Off. Testing & Res.) Sept. 1997	US FDA (Centre for Drug Eval.& Res./Off. Testing & Res.) Sept. 1997	US FDA (Centre for Drug Eval.& Res./Off. Testing & Res.) Sept. 1997

Model Applicability

Unknown features are fingerprint features in the query molecule, but not found in the training set.

1. All properties and OPS components are within expected ranges.
2. Unknown ECFP_2 feature: -955816473: [*]SCC(=[*])[*]
3. Unknown ECFP_2 feature: 1427820655: [*]CS[c](:[*]):[*]
4. Unknown ECFP_2 feature: 77911192: [*][c]1:[*]:[*]:[c](:[*]):n:1:[c](:[*]):[*]
5. Unknown ECFP_2 feature: 1986731747: [*]S[c]1:n:[*]:[*]:n:1:[*]

Feature Contribution

Top features for positive contribution

Fingerprint	Bit/Smiles	Feature Structure	Score	Carcinogen in training set
ECFP_6	738938915	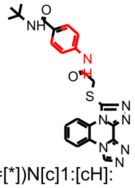 [*]C(=[*])N[c]1:[cH]:[cH]:[*]:[cH]:[cH]:1	0.617	2 out of 2

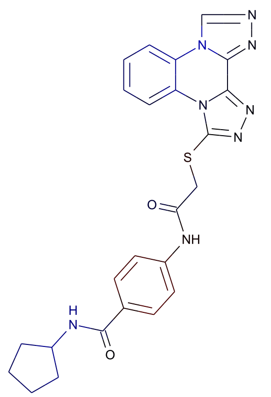

$C_{24}H_{22}N_8O_2S$
Molecular Weight: 486.54888
ALogP: 3.496
Rotatable Bonds: 6
Acceptors: 7
Donors: 2

Model Prediction

Prediction: Non-Carcinogen
Probability: 0.206
Enrichment: 0.641
Bayesian Score: -7.124
Mahalanobis Distance: 11.225
Mahalanobis Distance p-value: 0.067

Prediction: Positive if the Bayesian score is above the estimated best cutoff value from minimizing the false positive and false negative rate.
Probability: The esimated probability that the sample is in the positive category. This assumes that the Bayesian score follows a normal distribution and is different from the prediction using a cutoff.
Enrichment: An estimate of enrichment, that is, the increased likelihood (versus random) of this sample being in the category.
Bayesian Score: The standard Laplacian-modified Bayesian score.
Mahalanobis Distance: The Mahalanobis distance (MD) is the distance to the center of the training data. The larger the MD, the less trustworthy the prediction.
Mahalanobis Distance p-value: The p-value gives the fraction of training data with an MD greater than or equal to the one for the given sample, assuming normally distributed data. The smaller the p-value, the less trustworthy the prediciton. For highly non-normal X properties (e.g., fingerprints), the MD p-value is wildly inaccurate.

Structural Similar Compounds

Name	Bicalutamide	Glipizide	Moricizine
Structure	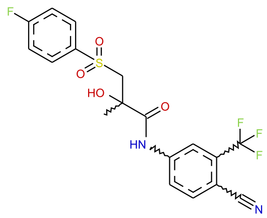	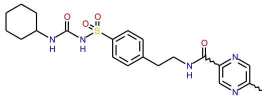	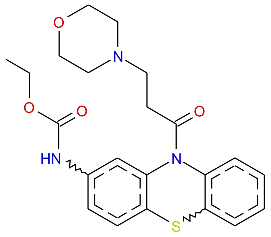
Actual Endpoint	Non-Carcinogen	Non-Carcinogen	Carcinogen
Predicted Endpoint	Non-Carcinogen	Non-Carcinogen	Carcinogen
Distance	0.666	0.668	0.683
Reference	US FDA (Centre for Drug Eval.& Res./Off. Testing & Res.) Sept. 1997	US FDA (Centre for Drug Eval.& Res./Off. Testing & Res.) Sept. 1997	US FDA (Centre for Drug Eval.& Res./Off. Testing & Res.) Sept. 1997

Model Applicability

Unknown features are fingerprint features in the query molecule, but not found in the training set.

1. All properties and OPS components are within expected ranges.
2. Unknown ECFP_2 feature: -955816473: [*]SCC(=[*])[*]
3. Unknown ECFP_2 feature: 77911192: [*][c]1:[*]:[*]:[c](:[*]):n:1:[c](:[*]):[*]
4. Unknown ECFP_2 feature: 1986731747: [*]S[c]1:n:[*]:[*]:n:1:[*]
5. Unknown ECFP_2 feature: 1427820655: [*]CS[c](:[*]):[*]

Feature Contribution

Top features for positive contribution				
**Fingerprint**	**Bit/Smiles**	**Feature Structure**	**Score**	**Carcinogen in training set**
ECFP_6	738938915	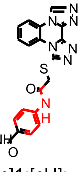 [*]C(=[*])N[c]1:[cH]:[cH]:[*]:[cH]:[cH]:1	0.617	2 out of 2

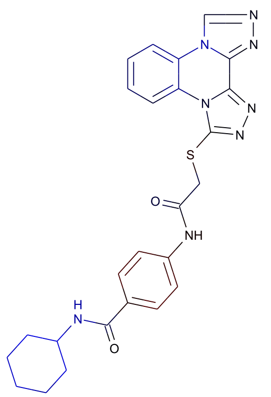

$C_{25}H_{24}N_8O_2S$

Molecular Weight: 500.57546

ALogP: 3.952

Rotatable Bonds: 6

Acceptors: 7

Donors: 2

Structural Similar Compounds

Name	Fluticasone	Glyburide	Glimepride
Structure	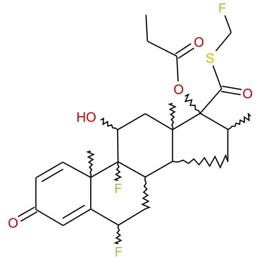	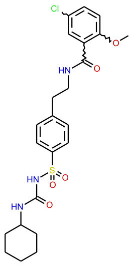	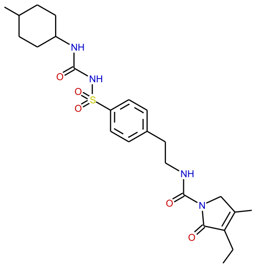
Actual Endpoint	Non-Carcinogen	Non-Carcinogen	Carcinogen
Predicted Endpoint	Non-Carcinogen	Non-Carcinogen	Carcinogen
Distance	0.673	0.684	0.688
Reference	US FDA (Centre for Drug Eval.& Res./Off. Testing & Res.) Sept. 1997	US FDA (Centre for Drug Eval.& Res./Off. Testing & Res.) Sept. 1997	US FDA (Centre for Drug Eval.& Res./Off. Testing & Res.) Sept. 1997

Model Prediction

Prediction: Non-Carcinogen

Probability: 0.217

Enrichment: 0.677

Bayesian Score: -9.302

Mahalanobis Distance: 11.251

Mahalanobis Distance p-value: 0.0634

Prediction: Positive if the Bayesian score is above the estimated best cutoff value from minimizing the false positive and false negative rate.
Probability: The esimated probability that the sample is in the positive category. This assumes that the Bayesian score follows a normal distribution and is different from the prediction using a cutoff.
Enrichment: An estimate of enrichment, that is, the increased likelihood (versus random) of this sample being in the category.
Bayesian Score: The standard Laplacian-modified Bayesian score.
Mahalanobis Distance: The Mahalanobis distance (MD) is the distance to the center of the training data. The larger the MD, the less trustworthy the prediction.
Mahalanobis Distance p-value: The p-value gives the fraction of training data with an MD greater than or equal to the one for the given sample, assuming normally distributed data. The smaller the p-value, the less trustworthy the prediciton. For highly non-normal X properties (e.g., fingerprints), the MD p-value is wildly inaccurate.

Model Applicability

Unknown features are fingerprint features in the query molecule, but not found in the training set.

1. All properties and OPS components are within expected ranges.
2. Unknown ECFP_2 feature: -955816473: [*]SCC(=[*])[*]
3. Unknown ECFP_2 feature: 77911192: [*][c]1:[*]:[*]:[c](:[*]):n:1:[c](:[*]):[*]
4. Unknown ECFP_2 feature: 1986731747: [*]S[c]1:n:[*]:[*]:n:1:[*]
5. Unknown ECFP_2 feature: 1427820655: [*]CS[c](:[*]):[*]

Feature Contribution

Top features for positive contribution

Fingerprint	Bit/Smiles	Feature Structure	Score	Carcinogen in training set
ECFP_6	738938915	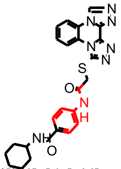 [*]C(=[*])N[c]1:[cH]:[cH]:[*]:[cH]:[cH]:1	0.617	2 out of 2

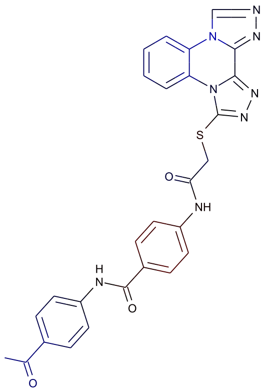

$C_{27}H_{20}N_8O_3S$

Molecular Weight: 536.56449

ALogP: 3.41

Rotatable Bonds: 7

Acceptors: 8

Donors: 2

Model Prediction

Prediction: Non-Carcinogen

Probability: 0.205

Enrichment: 0.639

Bayesian Score: -6.249

Mahalanobis Distance: 12.101

Mahalanobis Distance p-value: 0.0082

Prediction: Positive if the Bayesian score is above the estimated best cutoff value from minimizing the false positive and false negative rate.
Probability: The esimated probability that the sample is in the positive category. This assumes that the Bayesian score follows a normal distribution and is different from the prediction using a cutoff.
Enrichment: An estimate of enrichment, that is, the increased likelihood (versus random) of this sample being in the category.
Bayesian Score: The standard Laplacian-modified Bayesian score.
Mahalanobis Distance: The Mahalanobis distance (MD) is the distance to the center of the training data. The larger the MD, the less trustworthy the prediction.
Mahalanobis Distance p-value: The p-value gives the fraction of training data with an MD greater than or equal to the one for the given sample, assuming normally distributed data. The smaller the p-value, the less trustworthy the prediciton. For highly non-normal X properties (e.g., fingerprints), the MD p-value is wildly inaccurate.

Structural Similar Compounds

Name	Fluticasone	Glipizide	Bacampicillin
Structure	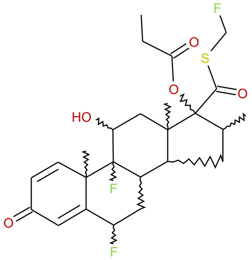	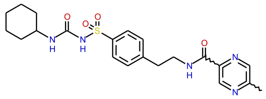	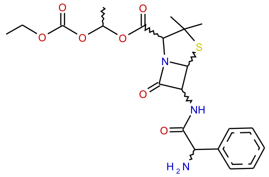
Actual Endpoint	Non-Carcinogen	Non-Carcinogen	Non-Carcinogen
Predicted Endpoint	Non-Carcinogen	Non-Carcinogen	Non-Carcinogen
Distance	0.752	0.765	0.765
Reference	US FDA (Centre for Drug Eval.& Res./Off. Testing & Res.) Sept. 1997	US FDA (Centre for Drug Eval.& Res./Off. Testing & Res.) Sept. 1997	US FDA (Centre for Drug Eval.& Res./Off. Testing & Res.) Sept. 1997

Model Applicability

Unknown features are fingerprint features in the query molecule, but not found in the training set.

1. All properties and OPS components are within expected ranges.
2. Unknown ECFP_2 feature: -955816473: [*]SCC(=[*])[*]
3. Unknown ECFP_2 feature: 77911192: [*][c]1:[*]:[*]:[c](:[*]):n:1:[c](:[*]):[*]
4. Unknown ECFP_2 feature: 1986731747: [*]S[c]1:n:[*]:[*]:n:1:[*]
5. Unknown ECFP_2 feature: 1427820655: [*]CS[c](:[*]):[*]

Feature Contribution

Top features for positive contribution				
**Fingerprint**	**Bit/Smiles**	**Feature Structure**	**Score**	**Carcinogen in training set**
ECFP_6	738938915	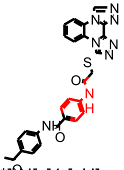 [*]C(=[*])N[c]1:[cH]:[cH]:[*]:[cH]:[cH]:1	0.617	2 out of 2

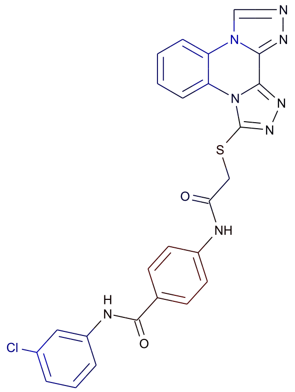

$C_{25}H_{17}ClN_8O_2S$
Molecular Weight: 528.97287
ALogP: 4.334
Rotatable Bonds: 6
Acceptors: 7
Donors: 2

Model Prediction

Prediction: Non-Carcinogen
Probability: 0.206
Enrichment: 0.642
Bayesian Score: -7.256
Mahalanobis Distance: 18.879
Mahalanobis Distance p-value: 1.72e-015

Prediction: Positive if the Bayesian score is above the estimated best cutoff value from minimizing the false positive and false negative rate.
Probability: The esimated probability that the sample is in the positive category. This assumes that the Bayesian score follows a normal distribution and is different from the prediction using a cutoff.
Enrichment: An estimate of enrichment, that is, the increased likelihood (versus random) of this sample being in the category.
Bayesian Score: The standard Laplacian-modified Bayesian score.
Mahalanobis Distance: The Mahalanobis distance (MD) is the distance to the center of the training data. The larger the MD, the less trustworthy the prediction.
Mahalanobis Distance p-value: The p-value gives the fraction of training data with an MD greater than or equal to the one for the given sample, assuming normally distributed data. The smaller the p-value, the less trustworthy the prediciton. For highly non-normal X properties (e.g., fingerprints), the MD p-value is wildly inaccurate.

Structural Similar Compounds

Name	Fluticasone	Glyburide	Glimepride
Structure	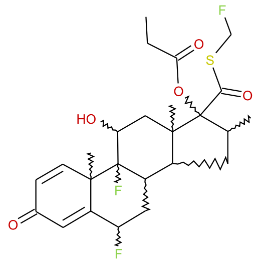	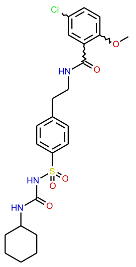	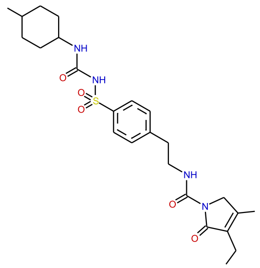
Actual Endpoint	Non-Carcinogen	Non-Carcinogen	Carcinogen
Predicted Endpoint	Non-Carcinogen	Non-Carcinogen	Carcinogen
Distance	0.682	0.719	0.721
Reference	US FDA (Centre for Drug Eval.& Res./Off. Testing & Res.) Sept. 1997	US FDA (Centre for Drug Eval.& Res./Off. Testing & Res.) Sept. 1997	US FDA (Centre for Drug Eval.& Res./Off. Testing & Res.) Sept. 1997

Model Applicability

Unknown features are fingerprint features in the query molecule, but not found in the training set.

1. All properties and OPS components are within expected ranges.
2. Unknown ECFP_2 feature: -955816473: [*]SCC(=[*])[*]
3. Unknown ECFP_2 feature: 77911192: [*][c]1:[*]:[*]:[c](:[*]):n:1:[c](:[*]):[*]
4. Unknown ECFP_2 feature: 1986731747: [*]S[c]1:n:[*]:[*]:n:1:[*]
5. Unknown ECFP_2 feature: 1427820655: [*]CS[c](:[*]):[*]

Feature Contribution

Top features for positive contribution

Fingerprint	Bit/Smiles	Feature Structure	Score	Carcinogen in training set
ECFP_6	738938915	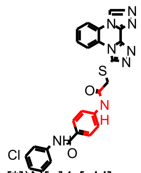 [*]C(=[*])N[c]1:[cH]:[cH]:[*]:[cH]:[cH]:1	0.617	2 out of 2

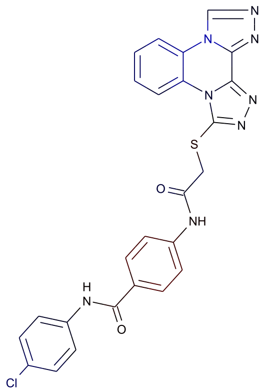

$C_{25}H_{17}ClN_8O_2S$
Molecular Weight: 528.97287
ALogP: 4.334
Rotatable Bonds: 6
Acceptors: 7
Donors: 2

Model Prediction

Prediction: Non-Carcinogen
Probability: 0.205
Enrichment: 0.640
Bayesian Score: -5.696
Mahalanobis Distance: 12.986
Mahalanobis Distance p-value: 0.000565

Prediction: Positive if the Bayesian score is above the estimated best cutoff value from minimizing the false positive and false negative rate.
Probability: The esimated probability that the sample is in the positive category. This assumes that the Bayesian score follows a normal distribution and is different from the prediction using a cutoff.
Enrichment: An estimate of enrichment, that is, the increased likelihood (versus random) of this sample being in the category.
Bayesian Score: The standard Laplacian-modified Bayesian score.
Mahalanobis Distance: The Mahalanobis distance (MD) is the distance to the center of the training data. The larger the MD, the less trustworthy the prediction.
Mahalanobis Distance p-value: The p-value gives the fraction of training data with an MD greater than or equal to the one for the given sample, assuming normally distributed data. The smaller the p-value, the less trustworthy the prediciton. For highly non-normal X properties (e.g., fingerprints), the MD p-value is wildly inaccurate.

Structural Similar Compounds

Name	Fluticasone	Glimepride	Glyburide
Structure	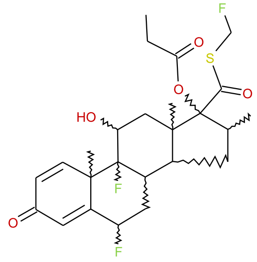	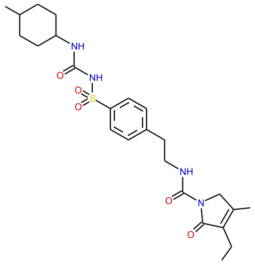	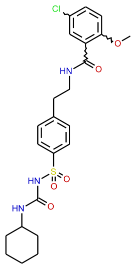
Actual Endpoint	Non-Carcinogen	Carcinogen	Non-Carcinogen
Predicted Endpoint	Non-Carcinogen	Carcinogen	Non-Carcinogen
Distance	0.682	0.720	0.721
Reference	US FDA (Centre for Drug Eval.& Res./Off. Testing & Res.) Sept. 1997	US FDA (Centre for Drug Eval.& Res./Off. Testing & Res.) Sept. 1997	US FDA (Centre for Drug Eval.& Res./Off. Testing & Res.) Sept. 1997

Model Applicability

Unknown features are fingerprint features in the query molecule, but not found in the training set.

1. All properties and OPS components are within expected ranges.
2. Unknown ECFP_2 feature: -955816473: [*]SCC(=[*])[*]
3. Unknown ECFP_2 feature: 77911192: [*][c]1:[*]:[*]:[c](:[*]):n:1:[c](:[*]):[*]
4. Unknown ECFP_2 feature: 1986731747: [*]S[c]1:n:[*]:[*]:n:1:[*]
5. Unknown ECFP_2 feature: 1427820655: [*]CS[c](:[*]):[*]

Feature Contribution

Top features for positive contribution

Fingerprint	Bit/Smiles	Feature Structure	Score	Carcinogen in training set
ECFP_6	738938915	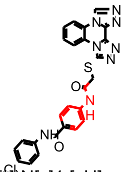 [*]C(=[*])N[c]1:[cH]:[cH]:[*]:[cH]:[cH]:1	0.617	2 out of 2

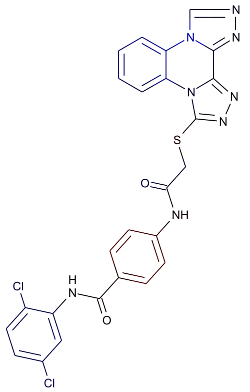

$C_{25}H_{16}Cl_2N_8O_2S$
Molecular Weight: 563.41793
ALogP: 4.999
Rotatable Bonds: 6
Acceptors: 7
Donors: 2

Model Prediction

Prediction: Non-Carcinogen
Probability: 0.205
Enrichment: 0.639
Bayesian Score: -6.713
Mahalanobis Distance: 11.978
Mahalanobis Distance p-value: 0.0114

Prediction: Positive if the Bayesian score is above the estimated best cutoff value from minimizing the false positive and false negative rate.
Probability: The esimated probability that the sample is in the positive category. This assumes that the Bayesian score follows a normal distribution and is different from the prediction using a cutoff.
Enrichment: An estimate of enrichment, that is, the increased likelihood (versus random) of this sample being in the category.
Bayesian Score: The standard Laplacian-modified Bayesian score.
Mahalanobis Distance: The Mahalanobis distance (MD) is the distance to the center of the training data. The larger the MD, the less trustworthy the prediction.
Mahalanobis Distance p-value: The p-value gives the fraction of training data with an MD greater than or equal to the one for the given sample, assuming normally distributed data. The smaller the p-value, the less trustworthy the prediciton. For highly non-normal X properties (e.g., fingerprints), the MD p-value is wildly inaccurate.

Structural Similar Compounds

Name	Fluticasone	Glyburide	Bromocriptine
Structure	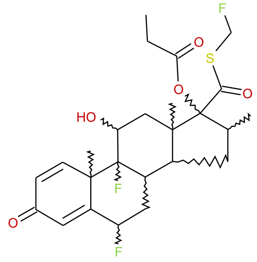	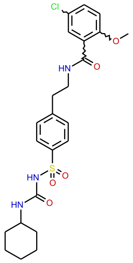	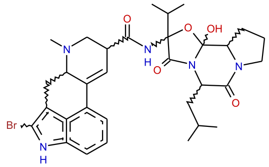
Actual Endpoint	Non-Carcinogen	Non-Carcinogen	Non-Carcinogen
Predicted Endpoint	Non-Carcinogen	Non-Carcinogen	Non-Carcinogen
Distance	0.712	0.744	0.744
Reference	US FDA (Centre for Drug Eval.& Res./Off. Testing & Res.) Sept. 1997	US FDA (Centre for Drug Eval.& Res./Off. Testing & Res.) Sept. 1997	US FDA (Centre for Drug Eval.& Res./Off. Testing & Res.) Sept. 1997

Model Applicability

Unknown features are fingerprint features in the query molecule, but not found in the training set.

1. All properties and OPS components are within expected ranges.
2. Unknown ECFP_2 feature: -955816473: [*]SCC(=[*])[*]
3. Unknown ECFP_2 feature: 77911192: [*][c]1:[*]:[*]:[c](:[*]):n:1:[c](:[*]):[*]
4. Unknown ECFP_2 feature: 1986731747: [*]S[c]1:n:[*]:[*]:n:1:[*]
5. Unknown ECFP_2 feature: 1427820655: [*]CS[c](:[*]):[*]

Feature Contribution

Top features for positive contribution				
**Fingerprint**	**Bit/Smiles**	**Feature Structure**	**Score**	**Carcinogen in training set**
ECFP_6	738938915	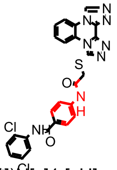 [*]C(=[*])N[c]1:[cH]:[cH]:[*]:[cH]:[cH]:1	0.617	2 out of 2

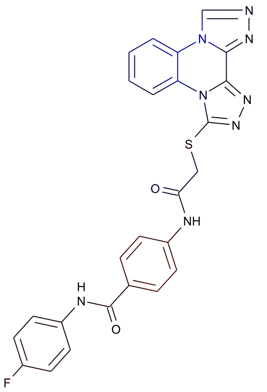

$C_{25}H_{17}FN_8O_2S$
Molecular Weight: 512.51828
ALogP: 3.875
Rotatable Bonds: 6
Acceptors: 7
Donors: 2

Model Prediction

Prediction: Non-Carcinogen
Probability: 0.214
Enrichment: 0.669
Bayesian Score: -3.648
Mahalanobis Distance: 12.024
Mahalanobis Distance p-value: 0.0101

Prediction: Positive if the Bayesian score is above the estimated best cutoff value from minimizing the false positive and false negative rate.
Probability: The esimated probability that the sample is in the positive category. This assumes that the Bayesian score follows a normal distribution and is different from the prediction using a cutoff.
Enrichment: An estimate of enrichment, that is, the increased likelihood (versus random) of this sample being in the category.
Bayesian Score: The standard Laplacian-modified Bayesian score.
Mahalanobis Distance: The Mahalanobis distance (MD) is the distance to the center of the training data. The larger the MD, the less trustworthy the prediction.
Mahalanobis Distance p-value: The p-value gives the fraction of training data with an MD greater than or equal to the one for the given sample, assuming normally distributed data. The smaller the p-value, the less trustworthy the prediciton. For highly non-normal X properties (e.g., fingerprints), the MD p-value is wildly inaccurate.

Structural Similar Compounds

Name	Bicalutamide	Fluticasone	Glimepride
Structure	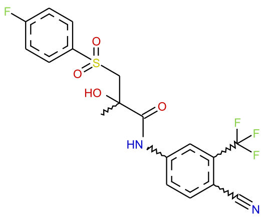	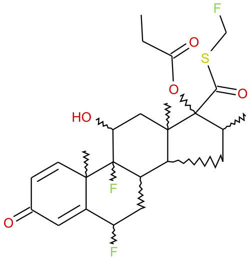	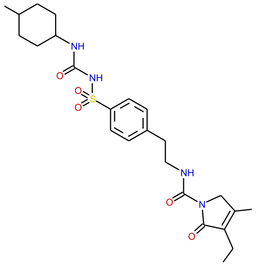
Actual Endpoint	Non-Carcinogen	Non-Carcinogen	Carcinogen
Predicted Endpoint	Non-Carcinogen	Non-Carcinogen	Carcinogen
Distance	0.674	0.678	0.710
Reference	US FDA (Centre for Drug Eval.& Res./Off. Testing & Res.) Sept. 1997	US FDA (Centre for Drug Eval.& Res./Off. Testing & Res.) Sept. 1997	US FDA (Centre for Drug Eval.& Res./Off. Testing & Res.) Sept. 1997

Model Applicability

Unknown features are fingerprint features in the query molecule, but not found in the training set.

1. All properties and OPS components are within expected ranges.
2. Unknown ECFP_2 feature: -955816473: [*]SCC(=[*])[*]
3. Unknown ECFP_2 feature: 77911192: [*][c]1:[*]:[*]:[c](:[*]):n:1:[c](:[*]):[*]
4. Unknown ECFP_2 feature: 1986731747: [*]S[c]1:n:[*]:[*]:n:1:[*]
5. Unknown ECFP_2 feature: 1427820655: [*]CS[c](:[*]):[*]

Feature Contribution

Top features for positive contribution				
**Fingerprint**	**Bit/Smiles**	**Feature Structure**	**Score**	**Carcinogen in training set**
ECFP_6	738938915	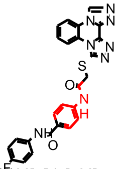 [*]C(=[*])N[c]1:[cH]:[cH]:[*]:[cH]:[cH]:1	0.617	2 out of 2

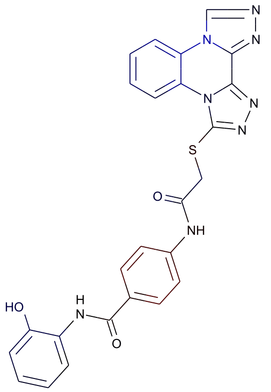

$C_{25}H_{18}N_8O_3S$
Molecular Weight: 510.52722
ALogP: 3.428
Rotatable Bonds: 6
Acceptors: 8
Donors: 3

Model Prediction

Prediction: Non-Carcinogen
Probability: 0.205
Enrichment: 0.640
Bayesian Score: -5.821
Mahalanobis Distance: 12.708
Mahalanobis Distance p-value: 0.00138

Prediction: Positive if the Bayesian score is above the estimated best cutoff value from minimizing the false positive and false negative rate.
Probability: The esimated probability that the sample is in the positive category. This assumes that the Bayesian score follows a normal distribution and is different from the prediction using a cutoff.
Enrichment: An estimate of enrichment, that is, the increased likelihood (versus random) of this sample being in the category.
Bayesian Score: The standard Laplacian-modified Bayesian score.
Mahalanobis Distance: The Mahalanobis distance (MD) is the distance to the center of the training data. The larger the MD, the less trustworthy the prediction.
Mahalanobis Distance p-value: The p-value gives the fraction of training data with an MD greater than or equal to the one for the given sample, assuming normally distributed data. The smaller the p-value, the less trustworthy the prediciton. For highly non-normal X properties (e.g., fingerprints), the MD p-value is wildly inaccurate.

Structural Similar Compounds

Name	Sulfasalazine	Glipizide	Glimepride
Structure	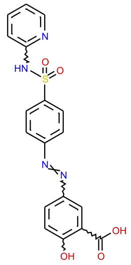	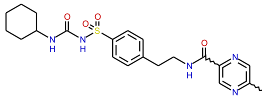	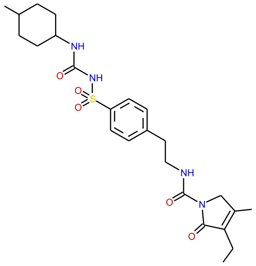
Actual Endpoint	Carcinogen	Non-Carcinogen	Carcinogen
Predicted Endpoint	Carcinogen	Non-Carcinogen	Carcinogen
Distance	0.659	0.725	0.758
Reference	US FDA (Centre for Drug Eval.& Res./Off. Testing & Res.) Sept. 1997	US FDA (Centre for Drug Eval.& Res./Off. Testing & Res.) Sept. 1997	US FDA (Centre for Drug Eval.& Res./Off. Testing & Res.) Sept. 1997

Model Applicability

Unknown features are fingerprint features in the query molecule, but not found in the training set.

1. All properties and OPS components are within expected ranges.
2. Unknown ECFP_2 feature: -955816473: [*]SCC(=[*])[*]
3. Unknown ECFP_2 feature: 77911192: [*][c]1:[*]:[*]:[c](:[*]):n:1:[c](:[*]):[*]
4. Unknown ECFP_2 feature: 1986731747: [*]S[c]1:n:[*]:[*]:n:1:[*]
5. Unknown ECFP_2 feature: 1427820655: [*]CS[c](:[*]):[*]

Feature Contribution

Top features for positive contribution

Fingerprint	Bit/Smiles	Feature Structure	Score	Carcinogen in training set
ECFP_6	738938915	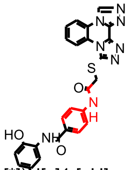 [*]C(=[*])N[c]1:[cH]:[cH]:[*]:[cH]:[cH]:1	0.617	2 out of 2

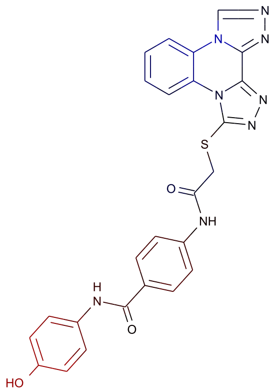

$C_{25}H_{18}N_8O_3S$
Molecular Weight: 510.52722
ALogP: 3.428
Rotatable Bonds: 6
Acceptors: 8
Donors: 3

Model Prediction

Prediction: Non-Carcinogen
Probability: 0.238
Enrichment: 0.742
Bayesian Score: -1.460
Mahalanobis Distance: 11.913
Mahalanobis Distance p-value: 0.0135

Prediction: Positive if the Bayesian score is above the estimated best cutoff value from minimizing the false positive and false negative rate.
Probability: The esimated probability that the sample is in the positive category. This assumes that the Bayesian score follows a normal distribution and is different from the prediction using a cutoff.
Enrichment: An estimate of enrichment, that is, the increased likelihood (versus random) of this sample being in the category.
Bayesian Score: The standard Laplacian-modified Bayesian score.
Mahalanobis Distance: The Mahalanobis distance (MD) is the distance to the center of the training data. The larger the MD, the less trustworthy the prediction.
Mahalanobis Distance p-value: The p-value gives the fraction of training data with an MD greater than or equal to the one for the given sample, assuming normally distributed data. The smaller the p-value, the less trustworthy the prediciton. For highly non-normal X properties (e.g., fingerprints), the MD p-value is wildly inaccurate.

Structural Similar Compounds

Name	Sulfasalazine	Glipizide	Glimepride
Structure	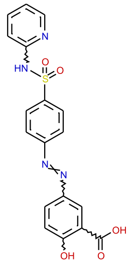	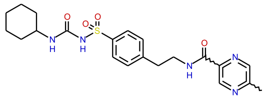	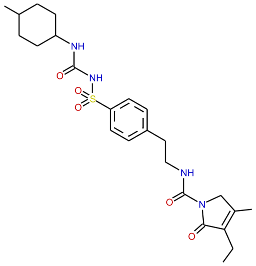
Actual Endpoint	Carcinogen	Non-Carcinogen	Carcinogen
Predicted Endpoint	Carcinogen	Non-Carcinogen	Carcinogen
Distance	0.662	0.724	0.758
Reference	US FDA (Centre for Drug Eval.& Res./Off. Testing & Res.) Sept. 1997	US FDA (Centre for Drug Eval.& Res./Off. Testing & Res.) Sept. 1997	US FDA (Centre for Drug Eval.& Res./Off. Testing & Res.) Sept. 1997

Model Applicability

Unknown features are fingerprint features in the query molecule, but not found in the training set.

1. All properties and OPS components are within expected ranges.
2. Unknown ECFP_2 feature: -955816473: [*]SCC(=[*])[*]
3. Unknown ECFP_2 feature: 77911192: [*][c]1:[*]:[*]:[c](:[*]):n:1:[c](:[*]):[*]
4. Unknown ECFP_2 feature: 1986731747: [*]S[c]1:n:[*]:[*]:n:1:[*]
5. Unknown ECFP_2 feature: 1427820655: [*]CS[c](:[*]):[*]

Feature Contribution

Top features for positive contribution

Fingerprint	Bit/Smiles	Feature Structure	Score	Carcinogen in training set
ECFP_6	1419645508	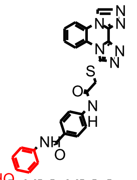 [*][c]1:[cH]:[cH]:[c](O):[cH]:[cH]:1	0.675	4 out of 5

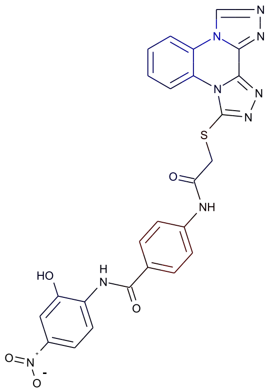

$C_{25}H_{17}N_9O_5S$

Molecular Weight: 555.52477

ALogP: 3.322

Rotatable Bonds: 7

Acceptors: 10

Donors: 3

Model Prediction

Prediction: Non-Carcinogen

Probability: 0.207

Enrichment: 0.645

Bayesian Score: -5.062

Mahalanobis Distance: 13.449

Mahalanobis Distance p-value: 0.000114

Prediction: Positive if the Bayesian score is above the estimated best cutoff value from minimizing the false positive and false negative rate.
Probability: The esimated probability that the sample is in the positive category. This assumes that the Bayesian score follows a normal distribution and is different from the prediction using a cutoff.
Enrichment: An estimate of enrichment, that is, the increased likelihood (versus random) of this sample being in the category.
Bayesian Score: The standard Laplacian-modified Bayesian score.
Mahalanobis Distance: The Mahalanobis distance (MD) is the distance to the center of the training data. The larger the MD, the less trustworthy the prediction.
Mahalanobis Distance p-value: The p-value gives the fraction of training data with an MD greater than or equal to the one for the given sample, assuming normally distributed data. The smaller the p-value, the less trustworthy the prediciton. For highly non-normal X properties (e.g., fingerprints), the MD p-value is wildly inaccurate.

Structural Similar Compounds

Name	Sulfasalazine	Bacampicillin	Nedocromil
Structure	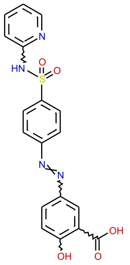	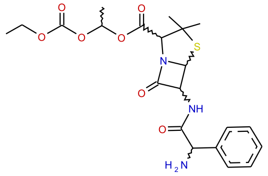	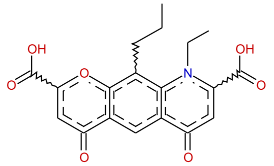
Actual Endpoint	Carcinogen	Non-Carcinogen	Non-Carcinogen
Predicted Endpoint	Carcinogen	Non-Carcinogen	Non-Carcinogen
Distance	0.777	0.822	0.894
Reference	US FDA (Centre for Drug Eval.& Res./Off. Testing & Res.) Sept. 1997	US FDA (Centre for Drug Eval.& Res./Off. Testing & Res.) Sept. 1997	US FDA (Centre for Drug Eval.& Res./Off. Testing & Res.) Sept. 1997

Model Applicability

Unknown features are fingerprint features in the query molecule, but not found in the training set.

1. All properties and OPS components are within expected ranges.
2. Unknown ECFP_2 feature: 1043790491: [*][N+](=[*])[*]
3. Unknown ECFP_2 feature: 781519895: [*][O-]
4. Unknown ECFP_2 feature: -955816473: [*]SCC(=[*])[*]
5. Unknown ECFP_2 feature: 77911192: [*][c]1:[*]:[*]:[c](:[*]):n:1:[c](:[*]):[*]
6. Unknown ECFP_2 feature: 1986731747: [*]S[c]1:n:[*]:[*]:n:1:[*]
7. Unknown ECFP_2 feature: 1427820655: [*]CS[c](:[*]):[*]
8. Unknown ECFP_2 feature: -179073144: [*][N+](=[*])[c](:c:[*]):c:[*]
9. Unknown ECFP_2 feature: -215026467: [*]:[c](:[*])[N+](=O)[O-]
10. Unknown ECFP_2 feature: 2104376220: [*][N+](=O)[*]
11. Unknown ECFP_2 feature: -659271057: [*][N+](=[*])[O-]

Feature Contribution

Top features for positive contribution				
**Fingerprint**	**Bit/Smiles**	**Feature Structure**	**Score**	**Carcinogen in training set**

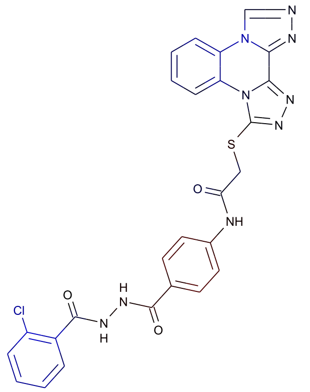

$C_{26}H_{18}ClN_9O_3S$
Molecular Weight: 571.99761
ALogP: 3.591
Rotatable Bonds: 7
Acceptors: 8
Donors: 3

Model Prediction

Prediction: Non-Carcinogen
Probability: 0.210
Enrichment: 0.655
Bayesian Score: -8.292
Mahalanobis Distance: 11.550
Mahalanobis Distance p-value: 0.0329

Prediction: Positive if the Bayesian score is above the estimated best cutoff value from minimizing the false positive and false negative rate.
Probability: The esimated probability that the sample is in the positive category. This assumes that the Bayesian score follows a normal distribution and is different from the prediction using a cutoff.
Enrichment: An estimate of enrichment, that is, the increased likelihood (versus random) of this sample being in the category.
Bayesian Score: The standard Laplacian-modified Bayesian score.
Mahalanobis Distance: The Mahalanobis distance (MD) is the distance to the center of the training data. The larger the MD, the less trustworthy the prediction.
Mahalanobis Distance p-value: The p-value gives the fraction of training data with an MD greater than or equal to the one for the given sample, assuming normally distributed data. The smaller the p-value, the less trustworthy the prediciton. For highly non-normal X properties (e.g., fingerprints), the MD p-value is wildly inaccurate.

Structural Similar Compounds

Name	Glyburide	Glimepride	Sulfasalazine
Structure	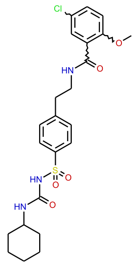	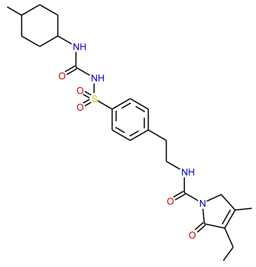	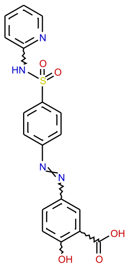
Actual Endpoint	Non-Carcinogen	Carcinogen	Carcinogen
Predicted Endpoint	Non-Carcinogen	Carcinogen	Carcinogen
Distance	0.775	0.777	0.780
Reference	US FDA (Centre for Drug Eval.& Res./Off. Testing & Res.) Sept. 1997	US FDA (Centre for Drug Eval.& Res./Off. Testing & Res.) Sept. 1997	US FDA (Centre for Drug Eval.& Res./Off. Testing & Res.) Sept. 1997

Model Applicability

Unknown features are fingerprint features in the query molecule, but not found in the training set.

1. All properties and OPS components are within expected ranges.
2. Unknown ECFP_2 feature: -955816473: [*]SCC(=[*])[*]
3. Unknown ECFP_2 feature: 77911192: [*][c]1:[*]:[*]:[c](:[*]):n:1:[c](:[*]):[*]
4. Unknown ECFP_2 feature: 1986731747: [*]S[c]1:n:[*]:[*]:n:1:[*]
5. Unknown ECFP_2 feature: 1427820655: [*]CS[c](:[*]):[*]

Feature Contribution

Top features for positive contribution				
**Fingerprint**	**Bit/Smiles**	**Feature Structure**	**Score**	**Carcinogen in training set**
ECFP_6	738938915	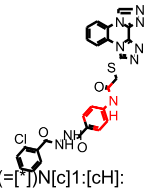 [*]C(=[*])N[c]1:[cH]:[cH]:[*]:[cH]:[cH]:1	0.617	2 out of 2

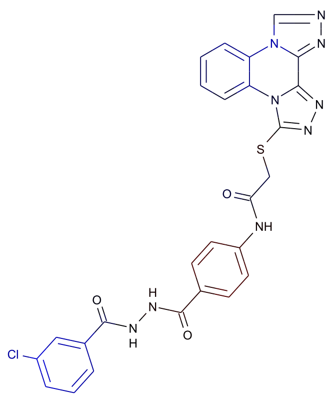

$C_{26}H_{18}ClN_9O_3S$
Molecular Weight: 571.99761
ALogP: 3.591
Rotatable Bonds: 7
Acceptors: 8
Donors: 3

Model Prediction

Prediction: Non-Carcinogen
Probability: 0.214
Enrichment: 0.669
Bayesian Score: -8.980
Mahalanobis Distance: 19.007
Mahalanobis Distance p-value: 8.82e-016

Prediction: Positive if the Bayesian score is above the estimated best cutoff value from minimizing the false positive and false negative rate.
Probability: The esimated probability that the sample is in the positive category. This assumes that the Bayesian score follows a normal distribution and is different from the prediction using a cutoff.
Enrichment: An estimate of enrichment, that is, the increased likelihood (versus random) of this sample being in the category.
Bayesian Score: The standard Laplacian-modified Bayesian score.
Mahalanobis Distance: The Mahalanobis distance (MD) is the distance to the center of the training data. The larger the MD, the less trustworthy the prediction.
Mahalanobis Distance p-value: The p-value gives the fraction of training data with an MD greater than or equal to the one for the given sample, assuming normally distributed data. The smaller the p-value, the less trustworthy the prediciton. For highly non-normal X properties (e.g., fingerprints), the MD p-value is wildly inaccurate.

Structural Similar Compounds

Name	Glyburide	Glimepride	Sulfasalazine
Structure	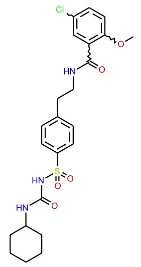	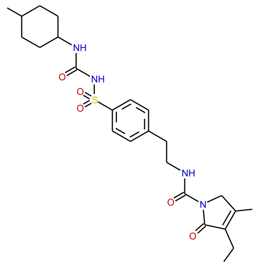	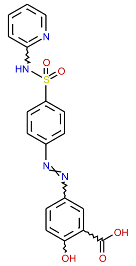
Actual Endpoint	Non-Carcinogen	Carcinogen	Carcinogen
Predicted Endpoint	Non-Carcinogen	Carcinogen	Carcinogen
Distance	0.768	0.777	0.780
Reference	US FDA (Centre for Drug Eval.& Res./Off. Testing & Res.) Sept. 1997	US FDA (Centre for Drug Eval.& Res./Off. Testing & Res.) Sept. 1997	US FDA (Centre for Drug Eval.& Res./Off. Testing & Res.) Sept. 1997

Model Applicability

Unknown features are fingerprint features in the query molecule, but not found in the training set.

1. All properties and OPS components are within expected ranges.
2. Unknown ECFP_2 feature: -955816473: [*]SCC(=[*])[*]
3. Unknown ECFP_2 feature: 77911192: [*][c]1:[*]:[*]:[c](:[*]):n:1:[c](:[*]):[*]
4. Unknown ECFP_2 feature: 1986731747: [*]S[c]1:n:[*]:[*]:n:1:[*]
5. Unknown ECFP_2 feature: 1427820655: [*]CS[c](:[*]):[*]

Feature Contribution

Top features for positive contribution

Fingerprint	Bit/Smiles	Feature Structure	Score	Carcinogen in training set
ECFP_6	738938915	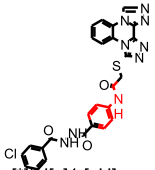 [*]C(=[*])N[c]1:[cH]:[cH]:[*]:[cH]:[cH]:1	0.617	2 out of 2

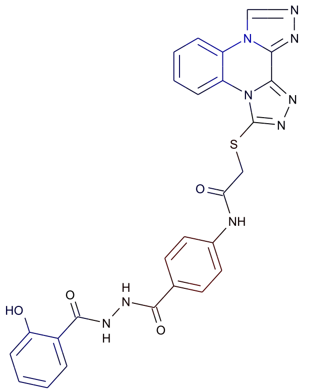

$C_{26}H_{19}N_9O_4S$
Molecular Weight: 553.55195
ALogP: 2.685
Rotatable Bonds: 7
Acceptors: 9
Donors: 4

Model Prediction

Prediction: Non-Carcinogen
Probability: 0.206
Enrichment: 0.642
Bayesian Score: -7.183
Mahalanobis Distance: 13.015
Mahalanobis Distance p-value: 0.000513

Prediction: Positive if the Bayesian score is above the estimated best cutoff value from minimizing the false positive and false negative rate.
Probability: The esimated probability that the sample is in the positive category. This assumes that the Bayesian score follows a normal distribution and is different from the prediction using a cutoff.
Enrichment: An estimate of enrichment, that is, the increased likelihood (versus random) of this sample being in the category.
Bayesian Score: The standard Laplacian-modified Bayesian score.
Mahalanobis Distance: The Mahalanobis distance (MD) is the distance to the center of the training data. The larger the MD, the less trustworthy the prediction.
Mahalanobis Distance p-value: The p-value gives the fraction of training data with an MD greater than or equal to the one for the given sample, assuming normally distributed data. The smaller the p-value, the less trustworthy the prediciton. For highly non-normal X properties (e.g., fingerprints), the MD p-value is wildly inaccurate.

Structural Similar Compounds

Name	Sulfasalazine	Daunorubicin	Glipizide
Structure	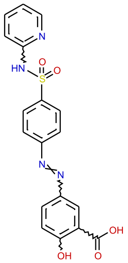	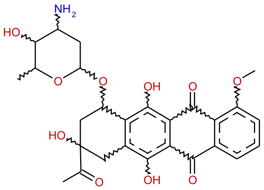	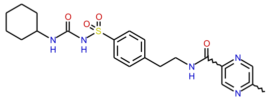
Actual Endpoint	Carcinogen	Non-Carcinogen	Non-Carcinogen
Predicted Endpoint	Carcinogen	Non-Carcinogen	Non-Carcinogen
Distance	0.775	0.820	0.829
Reference	US FDA (Centre for Drug Eval.& Res./Off. Testing & Res.) Sept. 1997	US FDA (Centre for Drug Eval.& Res./Off. Testing & Res.) Sept. 1997	US FDA (Centre for Drug Eval.& Res./Off. Testing & Res.) Sept. 1997

Model Applicability

Unknown features are fingerprint features in the query molecule, but not found in the training set.

1. All properties and OPS components are within expected ranges.
2. Unknown ECFP_2 feature: -955816473: [*]SCC(=[*])[*]
3. Unknown ECFP_2 feature: 77911192: [*][c]1:[*]:[*]:[c](:[*]):n:1:[c](:[*]):[*]
4. Unknown ECFP_2 feature: 1986731747: [*]S[c]1:n:[*]:[*]:n:1:[*]
5. Unknown ECFP_2 feature: 1427820655: [*]CS[c](:[*]):[*]

Feature Contribution

Top features for positive contribution				
Fingerprint	Bit/Smiles	Feature Structure	Score	Carcinogen in training set
ECFP_6	738938915	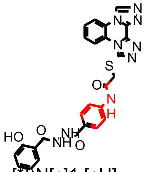 [*]C(=[*])N[c]1:[cH]:[cH]:[*]:[cH]:[cH]:1	0.617	2 out of 2

Sorafenib

TOPKAT_Mouse_Female_FDA_None_vs_Carcinogen

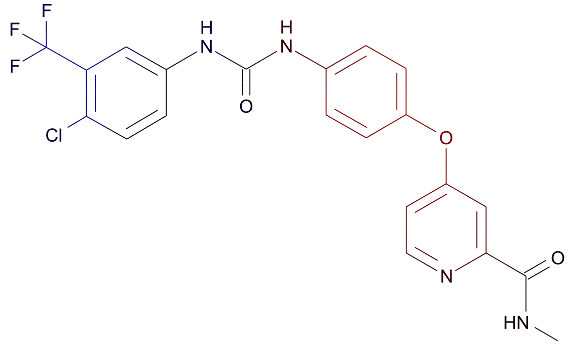

$C_{21}H_{16}ClF_3N_4O_3$
Molecular Weight: 464.82494
ALogP: 4.175
Rotatable Bonds: 6
Acceptors: 4
Donors: 3

Model Prediction

Prediction: Carcinogen
Probability: 0.257
Enrichment: 0.801
Bayesian Score: -0.321
Mahalanobis Distance: 14.877
Mahalanobis Distance p-value: 4.21e-007

Prediction: Positive if the Bayesian score is above the estimated best cutoff value from minimizing the false positive and false negative rate.
Probability: The esimated probability that the sample is in the positive category. This assumes that the Bayesian score follows a normal distribution and is different from the prediction using a cutoff.
Enrichment: An estimate of enrichment, that is, the increased likelihood (versus random) of this sample being in the category.
Bayesian Score: The standard Laplacian-modified Bayesian score.
Mahalanobis Distance: The Mahalanobis distance (MD) is the distance to the center of the training data. The larger the MD, the less trustworthy the prediction.
Mahalanobis Distance p-value: The p-value gives the fraction of training data with an MD greater than or equal to the one for the given sample, assuming normally distributed data. The smaller the p-value, the less trustworthy the prediciton. For highly non-normal X properties (e.g., fingerprints), the MD p-value is wildly inaccurate.

Structural Similar Compounds

Name	Glimepride	Glyburide	Fluvastatin
Structure	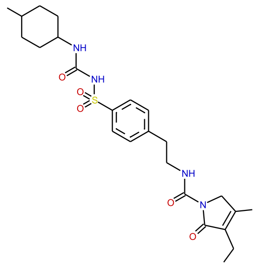	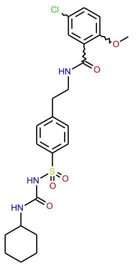	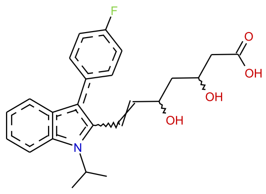
Actual Endpoint	Carcinogen	Non-Carcinogen	Non-Carcinogen
Predicted Endpoint	Carcinogen	Non-Carcinogen	Non-Carcinogen
Distance	0.605	0.615	0.625
Reference	US FDA (Centre for Drug Eval.& Res./Off. Testing & Res.) Sept. 1997	US FDA (Centre for Drug Eval.& Res./Off. Testing & Res.) Sept. 1997	US FDA (Centre for Drug Eval.& Res./Off. Testing & Res.) Sept. 1997

Model Applicability

Unknown features are fingerprint features in the query molecule, but not found in the training set.

1. OPS PC20 out of range. Value: -3.3309. Training min, max, SD, explained variance: -3.1862, 4.4571, 1.28, 0.0167.

Feature Contribution

Top features for positive contribution

Fingerprint	Bit/Smiles	Feature Structure	Score	Carcinogen in training set
ECFP_6	738938915	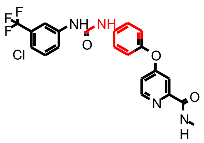 [*]C(=[*])N[c]1:[cH]:[cH]:[*]:[cH]:[cH]:1	0.617	2 out of 2

Sorafenib

TOPKAT_Mouse_Female_FDA_Single_vs_Multiple

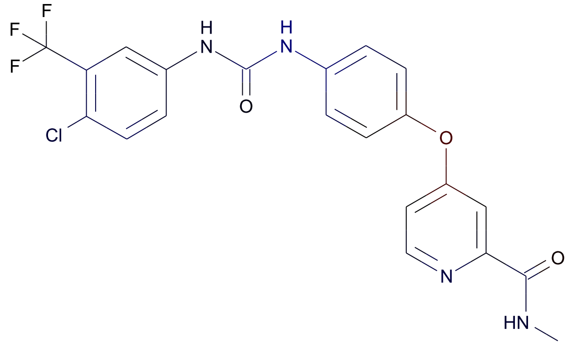

$C_{21}H_{16}ClF_3N_4O_3$

Molecular Weight: 464.82494

ALogP: 4.175

Rotatable Bonds: 6

Acceptors: 4

Donors: 3

Model Prediction

Prediction: Single-Carcinogen

Probability: 0.283

Enrichment: 0.691

Bayesian Score: -3.895

Mahalanobis Distance: 11.116

Mahalanobis Distance p-value: 0.00221

Prediction: Positive if the Bayesian score is above the estimated best cutoff value from minimizing the false positive and false negative rate.
Probability: The esimated probability that the sample is in the positive category. This assumes that the Bayesian score follows a normal distribution and is different from the prediction using a cutoff.
Enrichment: An estimate of enrichment, that is, the increased likelihood (versus random) of this sample being in the category.
Bayesian Score: The standard Laplacian-modified Bayesian score.
Mahalanobis Distance: The Mahalanobis distance (MD) is the distance to the center of the training data. The larger the MD, the less trustworthy the prediction.
Mahalanobis Distance p-value: The p-value gives the fraction of training data with an MD greater than or equal to the one for the given sample, assuming normally distributed data. The smaller the p-value, the less trustworthy the prediciton. For highly non-normal X properties (e.g., fingerprints), the MD p-value is wildly inaccurate.

Structural Similar Compounds

Name	Glimepride	Labetalol	Lansoprazole
Structure	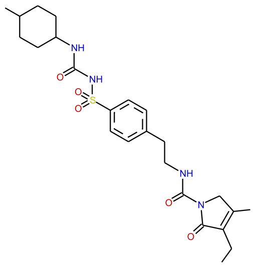	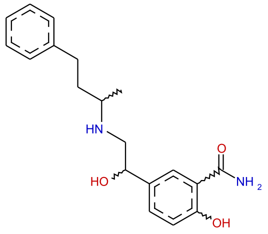	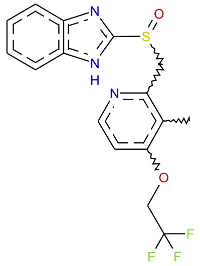
Actual Endpoint	Single-Carcinogen	Single-Carcinogen	Single-Carcinogen
Predicted Endpoint	Single-Carcinogen	Single-Carcinogen	Single-Carcinogen
Distance	0.599	0.808	0.820
Reference	US FDA (Centre for Drug Eval.& Res./Off. Testing & Res.) Sept. 1997	US FDA (Centre for Drug Eval.& Res./Off. Testing & Res.) Sept. 1997	US FDA (Centre for Drug Eval.& Res./Off. Testing & Res.) Sept. 1997

Model Applicability

Unknown features are fingerprint features in the query molecule, but not found in the training set.

1. All properties and OPS components are within expected ranges.
2. Unknown ECFP_2 feature: 1336678434: [*][c](:[*]):[c](C([*])([*])[*]):c:[*]
3. Unknown ECFP_2 feature: -1952889961: [*]:[c](:[*])C(F)(F)F

Feature Contribution

Top features for positive contribution

Fingerprint	Bit/Smiles	Feature Structure	Score	Multiple-Carcinogen in training set
ECFP_4	1407472008	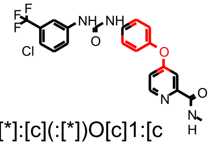 [*]:[c](:[*])O[c]1:[cH]:[cH]:[*]:[cH]:[cH]:1	0.351	1 out of 1

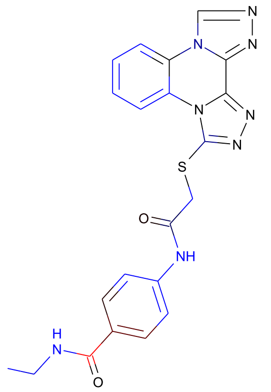

$C_{21}H_{18}N_8O_2S$
Molecular Weight: 446.48502
ALogP: 2.442
Rotatable Bonds: 6
Acceptors: 7
Donors: 2

Model Prediction

Prediction: 35.802
Unit: mg/kg_body_weight/day
Mahalanobis Distance: 16.005
Mahalanobis Distance p-value: 2.68e-014

Mahalanobis Distance: The Mahalanobis distance (MD) is a generalization of the Euclidean distance that accounts for correlations among the X properties. It is calculated as the distance to the center of the training data. The larger the MD, the less trustworthy the prediction.
Mahalanobis Distance p-value: The p-value gives the fraction of training data with an MD greater than or equal to the one for the given sample, assuming normally distributed data. The smaller the p-value, the less trustworthy the prediciton. For highly non-normal X properties (e.g., fingerprints), the MD p-value is wildly inaccurate.

Structural Similar Compounds

Name	4-Bis(2-hydroxyethyl)amino-2-(5-nitro-2-thienyl)quinazoline	C.I. direct brown 95	623
Structure	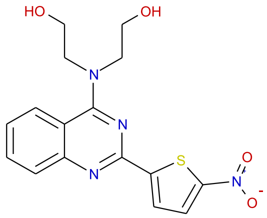	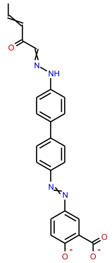	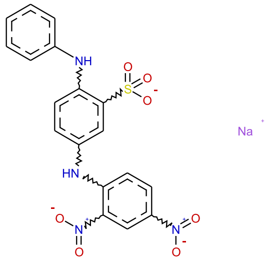
Actual Endpoint (-log C)	5.05984	5.31387	2.39985
Predicted Endpoint (-log C)	4.23808	4.30266	3.4177
Distance	0.818	0.840	0.857
Reference	CPDB	CPDB	CPDB

Model Applicability

Unknown features are fingerprint features in the query molecule, but not found in the training set.

1. All properties and OPS components are within expected ranges.

Feature Contribution

Top features for positive contribution			
Fingerprint	Bit/Smiles	Feature Structure	Score
FCFP_6	1	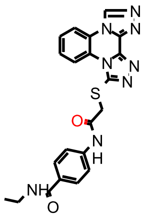 [*]=O	0.234

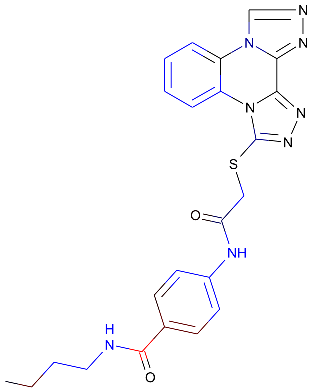

$C_{23}H_{22}N_8O_2S$
Molecular Weight: 474.53818
ALogP: 3.422
Rotatable Bonds: 8
Acceptors: 7
Donors: 2

Model Prediction

Prediction: 33.907
Unit: mg/kg_body_weight/day
Mahalanobis Distance: 17.940
Mahalanobis Distance p-value: 4.88e-021

Mahalanobis Distance: The Mahalanobis distance (MD) is a generalization of the Euclidean distance that accounts for correlations among the X properties. It is calculated as the distance to the center of the training data. The larger the MD, the less trustworthy the prediction.
Mahalanobis Distance p-value: The p-value gives the fraction of training data with an MD greater than or equal to the one for the given sample, assuming normally distributed data. The smaller the p-value, the less trustworthy the prediciton. For highly non-normal X properties (e.g., fingerprints), the MD p-value is wildly inaccurate.

Structural Similar Compounds

Name	C.I. direct brown 95	4-Bis(2-hydroxyethyl)amino-2-(5-nitro-2-thienyl)quinazoline	623
Structure	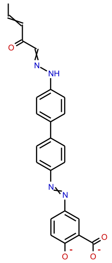	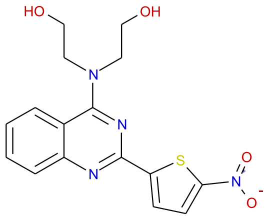	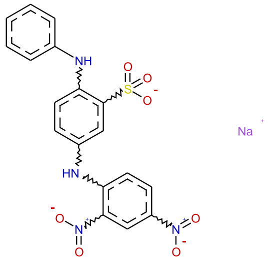
Actual Endpoint (-log C)	5.31387	5.05984	2.39985
Predicted Endpoint (-log C)	4.30266	4.23808	3.4177
Distance	0.827	0.842	0.876
Reference	CPDB	CPDB	CPDB

Model Applicability

Unknown features are fingerprint features in the query molecule, but not found in the training set.

1. All properties and OPS components are within expected ranges.

Feature Contribution

Top features for positive contribution			
Fingerprint	Bit/Smiles	Feature Structure	Score
FCFP_6	1	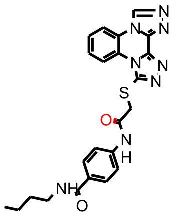 [*]=O	0.234

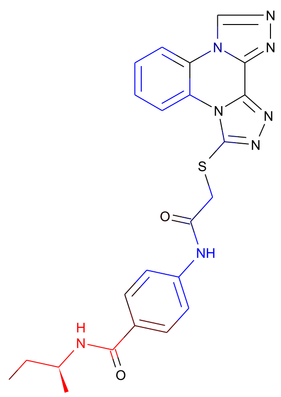

$C_{23}H_{22}N_8O_2S$
Molecular Weight: 474.53818
ALogP: 3.343
Rotatable Bonds: 7
Acceptors: 7
Donors: 2

Model Prediction

Prediction: 0.543
Unit: mg/kg_body_weight/day
Mahalanobis Distance: 16.971
Mahalanobis Distance p-value: 1.5e-017

Mahalanobis Distance: The Mahalanobis distance (MD) is a generalization of the Euclidean distance that accounts for correlations among the X properties. It is calculated as the distance to the center of the training data. The larger the MD, the less trustworthy the prediction.
Mahalanobis Distance p-value: The p-value gives the fraction of training data with an MD greater than or equal to the one for the given sample, assuming normally distributed data. The smaller the p-value, the less trustworthy the prediciton. For highly non-normal X properties (e.g., fingerprints), the MD p-value is wildly inaccurate.

Structural Similar Compounds

Name	C.I. direct brown 95	4-Bis(2-hydroxyethyl)amino-2-(5-nitro-2-thienyl)quinazoline	623
Structure	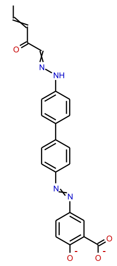	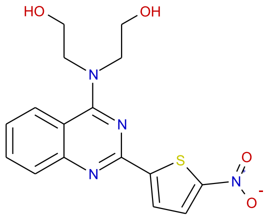	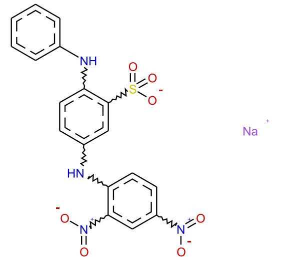
Actual Endpoint (-log C)	5.31387	5.05984	2.39985
Predicted Endpoint (-log C)	4.30266	4.23808	3.4177
Distance	0.829	0.843	0.872
Reference	CPDB	CPDB	CPDB

Model Applicability

Unknown features are fingerprint features in the query molecule, but not found in the training set.

1. All properties and OPS components are within expected ranges.

Feature Contribution

Top features for positive contribution			
**Fingerprint**	**Bit/Smiles**	**Feature Structure**	**Score**
FCFP_6	-1043250487	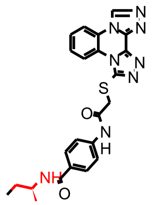 [*]C[C@H](C)N[*]	1.153

ter. butyl.cdx

TOPKAT_Carcinogenic_Potency_TD50_Rat

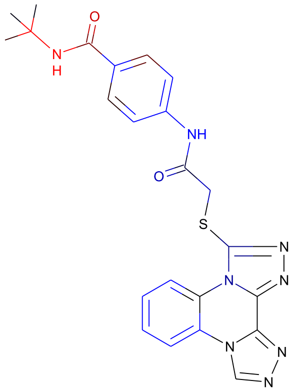

$C_{23}H_{22}N_8O_2S$

Molecular Weight: 474.53818

ALogP: 3.025

Rotatable Bonds: 6

Acceptors: 7

Donors: 2

Structural Similar Compounds

Name	C.I. direct brown 95	4-Bis(2-hydroxyethyl)amino-2-(5-nitro-2-thienyl)quinazoline	623
Structure	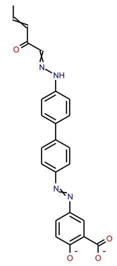	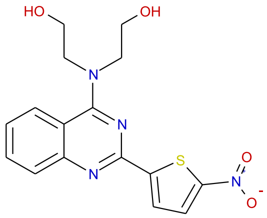	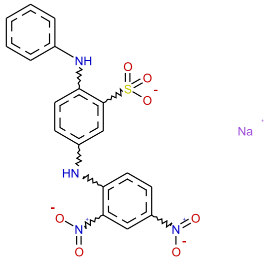
Actual Endpoint (-log C)	5.31387	5.05984	2.39985
Predicted Endpoint (-log C)	4.30266	4.23808	3.4177
Distance	0.837	0.842	0.868
Reference	CPDB	CPDB	CPDB

Model Prediction

Prediction: 10.185

Unit: mg/kg_body_weight/day

Mahalanobis Distance: 17.935

Mahalanobis Distance p-value: 5.07e-021

Mahalanobis Distance: The Mahalanobis distance (MD) is a generalization of the Euclidean distance that accounts for correlations among the X properties. It is calculated as the distance to the center of the training data. The larger the MD, the less trustworthy the prediction.
Mahalanobis Distance p-value: The p-value gives the fraction of training data with an MD greater than or equal to the one for the given sample, assuming normally distributed data. The smaller the p-value, the less trustworthy the prediciton. For highly non-normal X properties (e.g., fingerprints), the MD p-value is wildly inaccurate.

Model Applicability

Unknown features are fingerprint features in the query molecule, but not found in the training set.

1. All properties and OPS components are within expected ranges.

Feature Contribution

Top features for positive contribution

Fingerprint	Bit/Smiles	Feature Structure	Score
FCFP_6	1	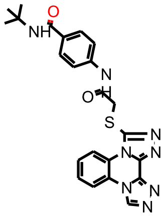 [*]=O	0.234

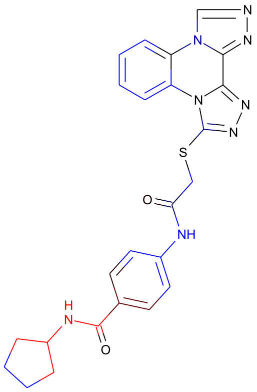

$C_{24}H_{22}N_{8}O_{2}S$
Molecular Weight: 486.54888
ALogP: 3.496
Rotatable Bonds: 6
Acceptors: 7
Donors: 2

Model Prediction

Prediction: 0.857
Unit: mg/kg_body_weight/day
Mahalanobis Distance: 18.069
Mahalanobis Distance p-value: 1.61e-021

Mahalanobis Distance: The Mahalanobis distance (MD) is a generalization of the Euclidean distance that accounts for correlations among the X properties. It is calculated as the distance to the center of the training data. The larger the MD, the less trustworthy the prediction.
Mahalanobis Distance p-value: The p-value gives the fraction of training data with an MD greater than or equal to the one for the given sample, assuming normally distributed data. The smaller the p-value, the less trustworthy the prediciton. For highly non-normal X properties (e.g., fingerprints), the MD p-value is wildly inaccurate.

Structural Similar Compounds

Name	C.I. direct brown 95	223	4-Bis(2-hydroxyethyl)amino-2-(5-nitro-2-thienyl)quinazoline
Structure	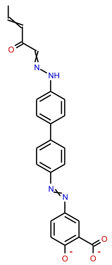	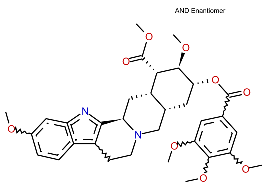	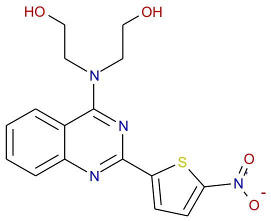
Actual Endpoint (-log C)	5.31387	6.29867	5.05984
Predicted Endpoint (-log C)	4.30266	7.5657	4.23808
Distance	0.904	0.906	0.914
Reference	CPDB	CPDB	CPDB

Model Applicability

Unknown features are fingerprint features in the query molecule, but not found in the training set.

1. All properties and OPS components are within expected ranges.

Feature Contribution

Top features for positive contribution			
Fingerprint	Bit/Smiles	Feature Structure	Score
FCFP_6	-1043250487	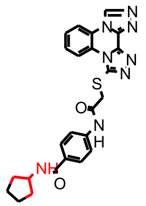 [*]C[C@H](C)N[*]	1.153

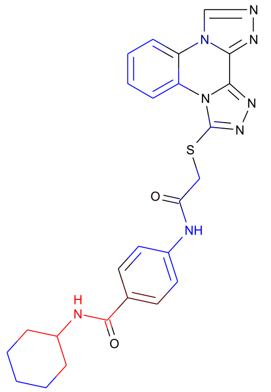

$C_{25}H_{24}N_8O_2S$
Molecular Weight: 500.57546
ALogP: 3.952
Rotatable Bonds: 6
Acceptors: 7
Donors: 2

Model Prediction

Prediction: 0.794
Unit: mg/kg_body_weight/day
Mahalanobis Distance: 18.048
Mahalanobis Distance p-value: 1.93e-021

Mahalanobis Distance: The Mahalanobis distance (MD) is a generalization of the Euclidean distance that accounts for correlations among the X properties. It is calculated as the distance to the center of the training data. The larger the MD, the less trustworthy the prediction.
Mahalanobis Distance p-value: The p-value gives the fraction of training data with an MD greater than or equal to the one for the given sample, assuming normally distributed data. The smaller the p-value, the less trustworthy the prediciton. For highly non-normal X properties (e.g., fingerprints), the MD p-value is wildly inaccurate.

Structural Similar Compounds

Name	223	C.I. direct brown 95	426
Structure	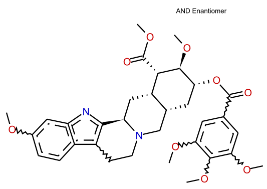	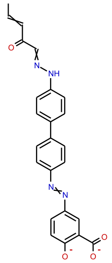	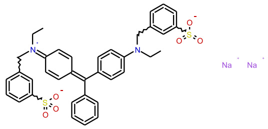
Actual Endpoint (-log C)	6.29867	5.31387	2.04218
Predicted Endpoint (-log C)	7.5657	4.30266	3.24288
Distance	0.895	0.909	0.918
Reference	CPDB	CPDB	CPDB

Model Applicability

Unknown features are fingerprint features in the query molecule, but not found in the training set.

1. All properties and OPS components are within expected ranges.

Feature Contribution

Top features for positive contribution

Fingerprint	Bit/Smiles	Feature Structure	Score
FCFP_6	-1043250487	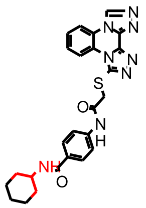 [*]C[C@H](C)N[*]	1.153

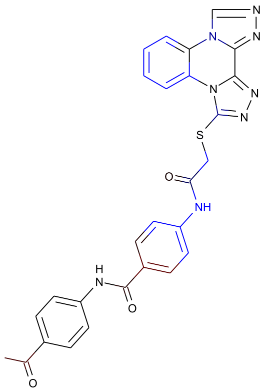

$C_{27}H_{20}N_8O_3S$
Molecular Weight: 536.56449
ALogP: 3.41
Rotatable Bonds: 7
Acceptors: 8
Donors: 2

Model Prediction

Prediction: 7.641
Unit: mg/kg_body_weight/day
Mahalanobis Distance: 16.676
Mahalanobis Distance p-value: 1.56e-016

Mahalanobis Distance: The Mahalanobis distance (MD) is a generalization of the Euclidean distance that accounts for correlations among the X properties. It is calculated as the distance to the center of the training data. The larger the MD, the less trustworthy the prediction.
Mahalanobis Distance p-value: The p-value gives the fraction of training data with an MD greater than or equal to the one for the given sample, assuming normally distributed data. The smaller the p-value, the less trustworthy the prediciton. For highly non-normal X properties (e.g., fingerprints), the MD p-value is wildly inaccurate.

Structural Similar Compounds

Name	411	426	223
Structure	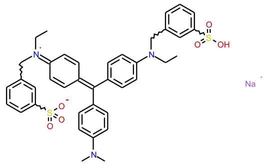	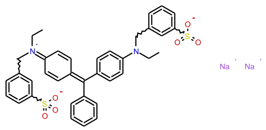	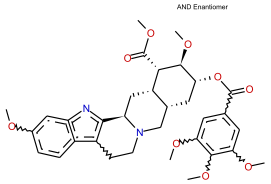
Actual Endpoint (-log C)	3.06566	2.04218	6.29867
Predicted Endpoint (-log C)	4.8672	3.24288	7.5657
Distance	0.929	0.930	0.964
Reference	CPDB	CPDB	CPDB

Model Applicability

Unknown features are fingerprint features in the query molecule, but not found in the training set.

1. All properties and OPS components are within expected ranges.

Feature Contribution

Top features for positive contribution			
Fingerprint	Bit/Smiles	Feature Structure	Score
FCFP_6	1	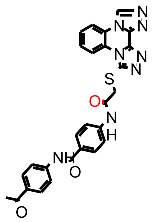 [*]=O	0.234

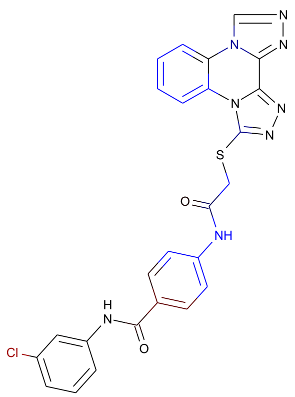

$C_{25}H_{17}ClN_8O_2S$
Molecular Weight: 528.97287
ALogP: 4.334
Rotatable Bonds: 6
Acceptors: 7
Donors: 2

Model Prediction

Prediction: 4.519
Unit: mg/kg_body_weight/day
Mahalanobis Distance: 17.345
Mahalanobis Distance p-value: 7.12e-019

Mahalanobis Distance: The Mahalanobis distance (MD) is a generalization of the Euclidean distance that accounts for correlations among the X properties. It is calculated as the distance to the center of the training data. The larger the MD, the less trustworthy the prediction.
Mahalanobis Distance p-value: The p-value gives the fraction of training data with an MD greater than or equal to the one for the given sample, assuming normally distributed data. The smaller the p-value, the less trustworthy the prediciton. For highly non-normal X properties (e.g., fingerprints), the MD p-value is wildly inaccurate.

Structural Similar Compounds

Name	411	426	223
Structure	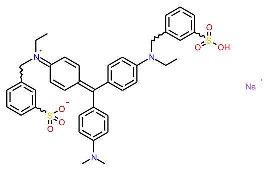	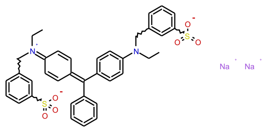	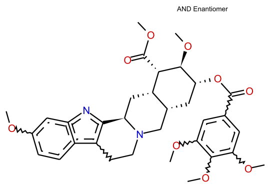
Actual Endpoint (-log C)	3.06566	2.04218	6.29867
Predicted Endpoint (-log C)	4.8672	3.24288	7.5657
Distance	0.955	0.960	0.977
Reference	CPDB	CPDB	CPDB

Model Applicability

Unknown features are fingerprint features in the query molecule, but not found in the training set.

1. All properties and OPS components are within expected ranges.

Feature Contribution

Top features for positive contribution

Fingerprint	Bit/Smiles	Feature Structure	Score
FCFP_6	1	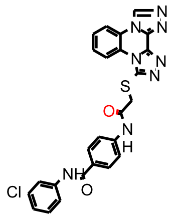 [*]=O	0.234

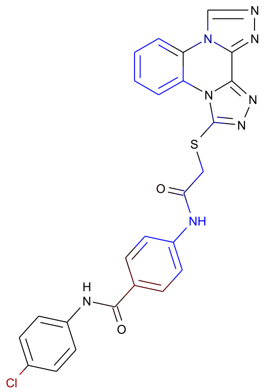

$C_{25}H_{17}ClN_8O_2S$
Molecular Weight: 528.97287
ALogP: 4.334
Rotatable Bonds: 6
Acceptors: 7
Donors: 2

Model Prediction

Prediction: 4.519
Unit: mg/kg_body_weight/day
Mahalanobis Distance: 17.345
Mahalanobis Distance p-value: 7.12e-019

Mahalanobis Distance: The Mahalanobis distance (MD) is a generalization of the Euclidean distance that accounts for correlations among the X properties. It is calculated as the distance to the center of the training data. The larger the MD, the less trustworthy the prediction.
Mahalanobis Distance p-value: The p-value gives the fraction of training data with an MD greater than or equal to the one for the given sample, assuming normally distributed data. The smaller the p-value, the less trustworthy the prediciton. For highly non-normal X properties (e.g., fingerprints), the MD p-value is wildly inaccurate.

Structural Similar Compounds

Name	411	426	223
Structure	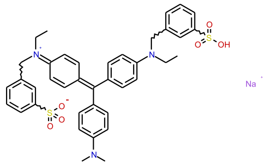	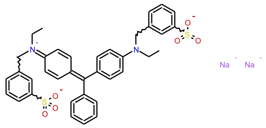	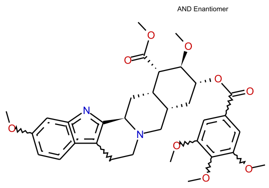
Actual Endpoint (-log C)	3.06566	2.04218	6.29867
Predicted Endpoint (-log C)	4.8672	3.24288	7.5657
Distance	0.955	0.959	0.976
Reference	CPDB	CPDB	CPDB

Model Applicability

Unknown features are fingerprint features in the query molecule, but not found in the training set.

1. All properties and OPS components are within expected ranges.

Feature Contribution

Top features for positive contribution			
**Fingerprint**	**Bit/Smiles**	**Feature Structure**	**Score**
FCFP_6	1	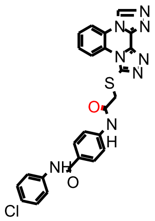 [*]=O	0.234

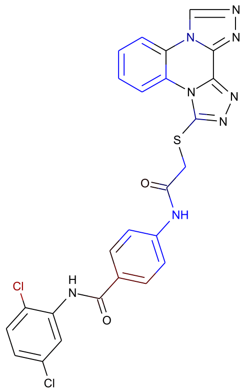

$C_{25}H_{16}Cl_2N_8O_2S$
Molecular Weight: 563.41793
ALogP: 4.999
Rotatable Bonds: 6
Acceptors: 7
Donors: 2

Model Prediction

Prediction: 4.056
Unit: mg/kg_body_weight/day
Mahalanobis Distance: 17.223
Mahalanobis Distance p-value: 1.94e-018

Mahalanobis Distance: The Mahalanobis distance (MD) is a generalization of the Euclidean distance that accounts for correlations among the X properties. It is calculated as the distance to the center of the training data. The larger the MD, the less trustworthy the prediction.
Mahalanobis Distance p-value: The p-value gives the fraction of training data with an MD greater than or equal to the one for the given sample, assuming normally distributed data. The smaller the p-value, the less trustworthy the prediciton. For highly non-normal X properties (e.g., fingerprints), the MD p-value is wildly inaccurate.

Structural Similar Compounds

Name	411	426	223
Structure	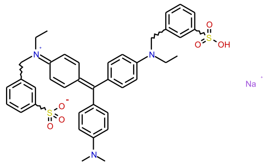	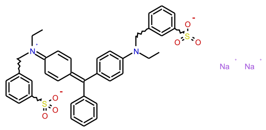	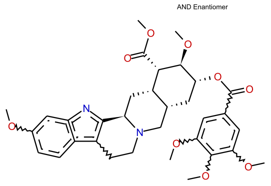
Actual Endpoint (-log C)	3.06566	2.04218	6.29867
Predicted Endpoint (-log C)	4.8672	3.24288	7.5657
Distance	0.934	0.958	0.972
Reference	CPDB	CPDB	CPDB

Model Applicability

Unknown features are fingerprint features in the query molecule, but not found in the training set.

1. All properties and OPS components are within expected ranges.

Feature Contribution

Top features for positive contribution			
Fingerprint	Bit/Smiles	Feature Structure	Score
FCFP_6	1	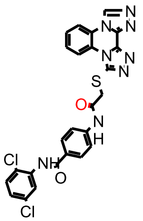 [*]=O	0.234

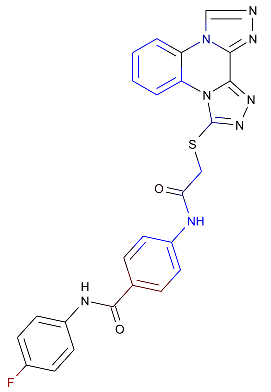

$C_{25}H_{17}FN_8O_2S$
Molecular Weight: 512.51828
ALogP: 3.875
Rotatable Bonds: 6
Acceptors: 7
Donors: 2

Model Prediction

Prediction: 4.882
Unit: mg/kg_body_weight/day
Mahalanobis Distance: 17.433
Mahalanobis Distance p-value: 3.44e-019

Mahalanobis Distance: The Mahalanobis distance (MD) is a generalization of the Euclidean distance that accounts for correlations among the X properties. It is calculated as the distance to the center of the training data. The larger the MD, the less trustworthy the prediction.
Mahalanobis Distance p-value: The p-value gives the fraction of training data with an MD greater than or equal to the one for the given sample, assuming normally distributed data. The smaller the p-value, the less trustworthy the prediciton. For highly non-normal X properties (e.g., fingerprints), the MD p-value is wildly inaccurate.

Structural Similar Compounds

Name	426	411	223
Structure	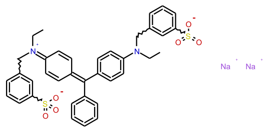	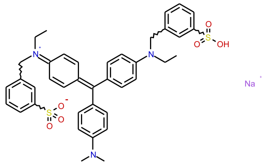	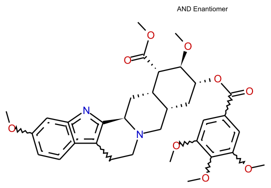
Actual Endpoint (-log C)	2.04218	3.06566	6.29867
Predicted Endpoint (-log C)	3.24288	4.8672	7.5657
Distance	0.959	0.966	0.980
Reference	CPDB	CPDB	CPDB

Model Applicability

Unknown features are fingerprint features in the query molecule, but not found in the training set.

1. All properties and OPS components are within expected ranges.

Feature Contribution

Top features for positive contribution

Fingerprint	Bit/Smiles	Feature Structure	Score
FCFP_6	1	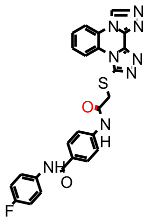 [*]=O	0.234

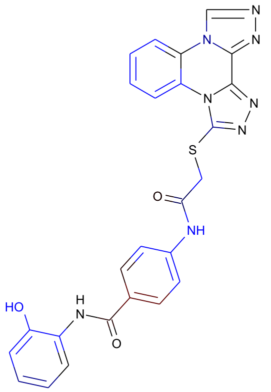

$C_{25}H_{18}N_8O_3S$
Molecular Weight: 510.52722
ALogP: 3.428
Rotatable Bonds: 6
Acceptors: 8
Donors: 3

Model Prediction

Prediction: 37.552
Unit: mg/kg_body_weight/day
Mahalanobis Distance: 17.095
Mahalanobis Distance p-value: 5.51e-018

Mahalanobis Distance: The Mahalanobis distance (MD) is a generalization of the Euclidean distance that accounts for correlations among the X properties. It is calculated as the distance to the center of the training data. The larger the MD, the less trustworthy the prediction.
Mahalanobis Distance p-value: The p-value gives the fraction of training data with an MD greater than or equal to the one for the given sample, assuming normally distributed data. The smaller the p-value, the less trustworthy the prediciton. For highly non-normal X properties (e.g., fingerprints), the MD p-value is wildly inaccurate.

Structural Similar Compounds

Name	623	188	411
Structure	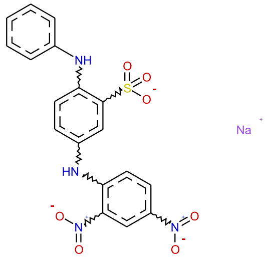	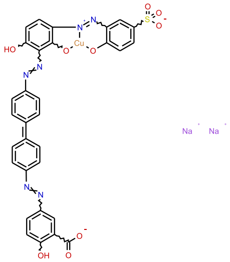	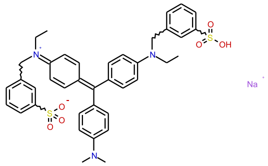
Actual Endpoint (-log C)	2.39985	5.5378	3.06566
Predicted Endpoint (-log C)	3.4177	5.71925	4.8672
Distance	0.999	1.012	1.015
Reference	CPDB	CPDB	CPDB

Model Applicability

Unknown features are fingerprint features in the query molecule, but not found in the training set.

1. All properties and OPS components are within expected ranges.

Feature Contribution

Top features for positive contribution			
**Fingerprint**	**Bit/Smiles**	**Feature Structure**	**Score**
FCFP_6	1	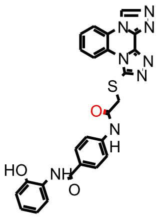 [*]=O	0.234

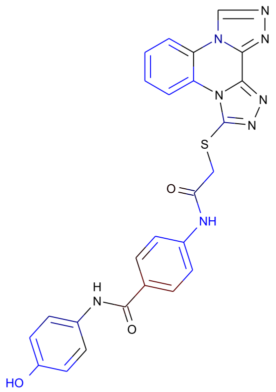

$C_{25}H_{18}N_8O_3S$
Molecular Weight: 510.52722
ALogP: 3.428
Rotatable Bonds: 6
Acceptors: 8
Donors: 3

Model Prediction

Prediction: 37.552
Unit: mg/kg_body_weight/day
Mahalanobis Distance: 17.095
Mahalanobis Distance p-value: 5.51e-018

Mahalanobis Distance: The Mahalanobis distance (MD) is a generalization of the Euclidean distance that accounts for correlations among the X properties. It is calculated as the distance to the center of the training data. The larger the MD, the less trustworthy the prediction.
Mahalanobis Distance p-value: The p-value gives the fraction of training data with an MD greater than or equal to the one for the given sample, assuming normally distributed data. The smaller the p-value, the less trustworthy the prediciton. For highly non-normal X properties (e.g., fingerprints), the MD p-value is wildly inaccurate.

Structural Similar Compounds

Name	623	188	411
Structure	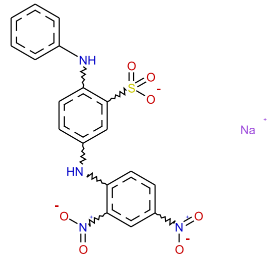	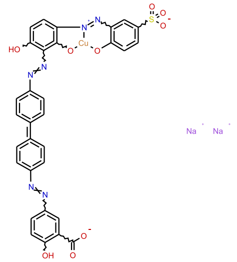	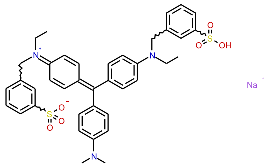
Actual Endpoint (-log C)	2.39985	5.5378	3.06566
Predicted Endpoint (-log C)	3.4177	5.71925	4.8672
Distance	1.003	1.011	1.015
Reference	CPDB	CPDB	CPDB

Model Applicability

Unknown features are fingerprint features in the query molecule, but not found in the training set.

1. All properties and OPS components are within expected ranges.

Feature Contribution

Top features for positive contribution

Fingerprint	Bit/Smiles	Feature Structure	Score
FCFP_6	1	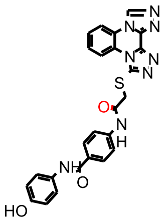 [*]=O	0.234

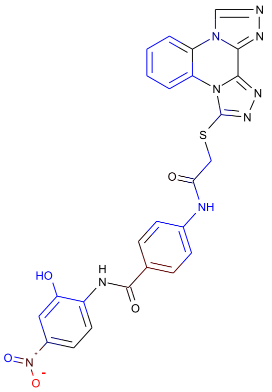

$C_{25}H_{17}N_9O_5S$
Molecular Weight: 555.52477
ALogP: 3.322
Rotatable Bonds: 7
Acceptors: 10
Donors: 3

Structural Similar Compounds

Name	188	654	623
Structure	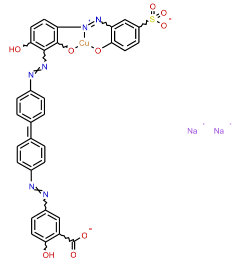	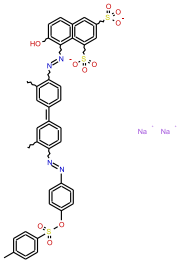	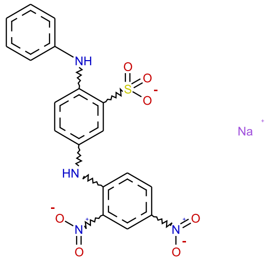
Actual Endpoint (-log C)	5.5378	5.30483	2.39985
Predicted Endpoint (-log C)	5.71925	5.10882	3.4177
Distance	0.825	0.968	0.983
Reference	CPDB	CPDB	CPDB

Model Prediction

Prediction: 13.503
Unit: mg/kg_body_weight/day
Mahalanobis Distance: 20.232
Mahalanobis Distance p-value: 5.18e-030

Mahalanobis Distance: The Mahalanobis distance (MD) is a generalization of the Euclidean distance that accounts for correlations among the X properties. It is calculated as the distance to the center of the training data. The larger the MD, the less trustworthy the prediction.
Mahalanobis Distance p-value: The p-value gives the fraction of training data with an MD greater than or equal to the one for the given sample, assuming normally distributed data. The smaller the p-value, the less trustworthy the prediciton. For highly non-normal X properties (e.g., fingerprints), the MD p-value is wildly inaccurate.

Model Applicability

Unknown features are fingerprint features in the query molecule, but not found in the training set.

1. All properties and OPS components are within expected ranges.

Feature Contribution

Top features for positive contribution			
**Fingerprint**	**Bit/Smiles**	**Feature Structure**	**Score**
FCFP_6	5	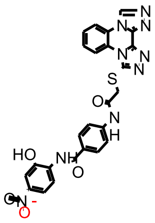 [*][O-]	0.431

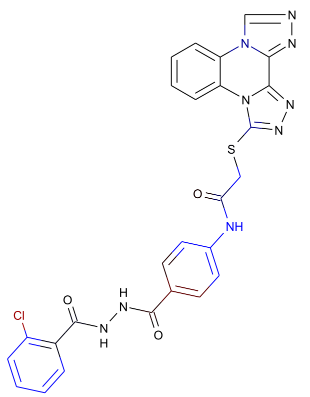

$C_{26}H_{18}ClN_9O_3S$
Molecular Weight: 571.99761
ALogP: 3.591
Rotatable Bonds: 7
Acceptors: 8
Donors: 3

Model Prediction

Prediction: 5.280
Unit: mg/kg_body_weight/day
Mahalanobis Distance: 17.394
Mahalanobis Distance p-value: 4.77e-019

Mahalanobis Distance: The Mahalanobis distance (MD) is a generalization of the Euclidean distance that accounts for correlations among the X properties. It is calculated as the distance to the center of the training data. The larger the MD, the less trustworthy the prediction.
Mahalanobis Distance p-value: The p-value gives the fraction of training data with an MD greater than or equal to the one for the given sample, assuming normally distributed data. The smaller the p-value, the less trustworthy the prediciton. For highly non-normal X properties (e.g., fingerprints), the MD p-value is wildly inaccurate.

Structural Similar Compounds

Name	188	411	426
Structure	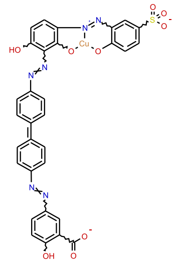	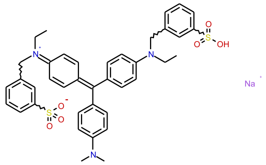	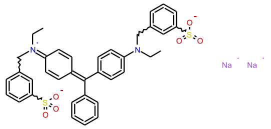
Actual Endpoint (-log C)	5.5378	3.06566	2.04218
Predicted Endpoint (-log C)	5.71925	4.8672	3.24288
Distance	0.966	0.972	1.007
Reference	CPDB	CPDB	CPDB

Model Applicability

Unknown features are fingerprint features in the query molecule, but not found in the training set.

1. OPS PC18 out of range. Value: 5.0069. Training min, max, SD, explained variance: -4.1023, 4.8669, 1.305, 0.0164.

Feature Contribution

Top features for positive contribution

Fingerprint	Bit/Smiles	Feature Structure	Score
FCFP_6	1	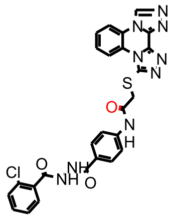 [*]=O	0.234

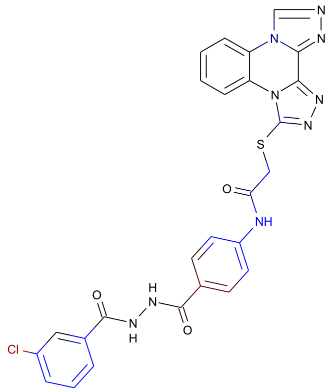

$C_{26}H_{18}ClN_9O_3S$
Molecular Weight: 571.99761
ALogP: 3.591
Rotatable Bonds: 7
Acceptors: 8
Donors: 3

Model Prediction

Prediction: 5.080
Unit: mg/kg_body_weight/day
Mahalanobis Distance: 17.344
Mahalanobis Distance p-value: 7.17e-019

Mahalanobis Distance: The Mahalanobis distance (MD) is a generalization of the Euclidean distance that accounts for correlations among the X properties. It is calculated as the distance to the center of the training data. The larger the MD, the less trustworthy the prediction.
Mahalanobis Distance p-value: The p-value gives the fraction of training data with an MD greater than or equal to the one for the given sample, assuming normally distributed data. The smaller the p-value, the less trustworthy the prediciton. For highly non-normal X properties (e.g., fingerprints), the MD p-value is wildly inaccurate.

Structural Similar Compounds

Name	188	411	426
Structure	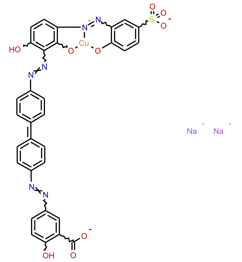	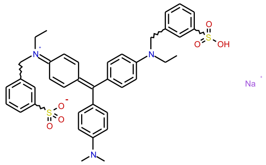	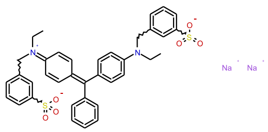
Actual Endpoint (-log C)	5.5378	3.06566	2.04218
Predicted Endpoint (-log C)	5.71925	4.8672	3.24288
Distance	0.966	0.972	1.011
Reference	CPDB	CPDB	CPDB

Model Applicability

Unknown features are fingerprint features in the query molecule, but not found in the training set.

1. All properties and OPS components are within expected ranges.

Feature Contribution

Top features for positive contribution

Fingerprint	Bit/Smiles	Feature Structure	Score
FCFP_6	1	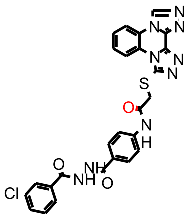 [*]=O	0.234

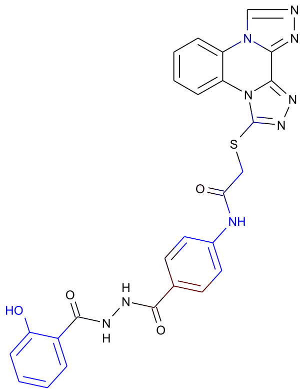

$C_{26}H_{19}N_9O_4S$

Molecular Weight: 553.55195

ALogP: 2.685

Rotatable Bonds: 7

Acceptors: 9

Donors: 4

Model Prediction

Prediction: 44.001

Unit: mg/kg_body_weight/day

Mahalanobis Distance: 17.320

Mahalanobis Distance p-value: 8.73e-019

Mahalanobis Distance: The Mahalanobis distance (MD) is a generalization of the Euclidean distance that accounts for correlations among the X properties. It is calculated as the distance to the center of the training data. The larger the MD, the less trustworthy the prediction.
Mahalanobis Distance p-value: The p-value gives the fraction of training data with an MD greater than or equal to the one for the given sample, assuming normally distributed data. The smaller the p-value, the less trustworthy the prediciton. For highly non-normal X properties (e.g., fingerprints), the MD p-value is wildly inaccurate.

Structural Similar Compounds

Name	188	623	411
Structure	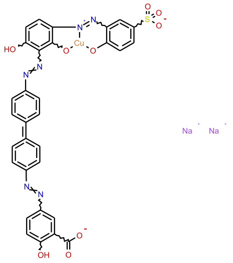	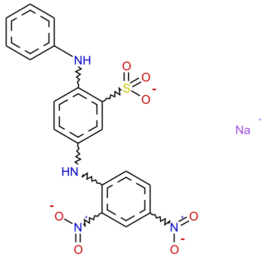	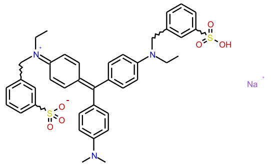
Actual Endpoint (-log C)	5.5378	2.39985	3.06566
Predicted Endpoint (-log C)	5.71925	3.4177	4.8672
Distance	0.942	1.045	1.083
Reference	CPDB	CPDB	CPDB

Model Applicability

Unknown features are fingerprint features in the query molecule, but not found in the training set.

1. All properties and OPS components are within expected ranges.

Feature Contribution

Top features for positive contribution			
**Fingerprint**	**Bit/Smiles**	**Feature Structure**	**Score**
FCFP_6	1	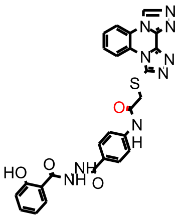 [*]=O	0.234

Sorafenib

TOPKAT_Carcinogenic_Potency_TD50_Rat

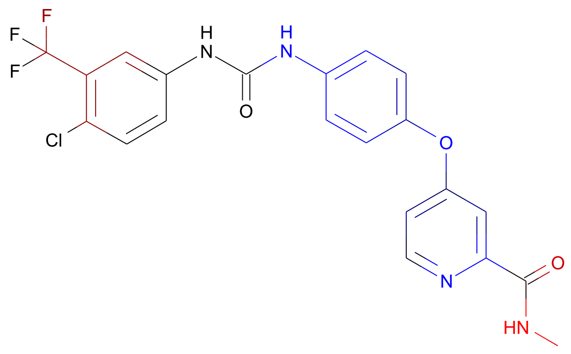

$C_{21}H_{16}ClF_3N_4O_3$

Molecular Weight: 464.82494

ALogP: 4.175

Rotatable Bonds: 6

Acceptors: 4

Donors: 3

Model Prediction

Prediction: 14.244

Unit: mg/kg_body_weight/day

Mahalanobis Distance: 20.410

Mahalanobis Distance p-value: 9.56e-031

Mahalanobis Distance: The Mahalanobis distance (MD) is a generalization of the Euclidean distance that accounts for correlations among the X properties. It is calculated as the distance to the center of the training data. The larger the MD, the less trustworthy the prediction.
Mahalanobis Distance p-value: The p-value gives the fraction of training data with an MD greater than or equal to the one for the given sample, assuming normally distributed data. The smaller the p-value, the less trustworthy the prediciton. For highly non-normal X properties (e.g., fingerprints), the MD p-value is wildly inaccurate.

Structural Similar Compounds

Name	Fluvastatin	913	Ochratoxin A
Structure	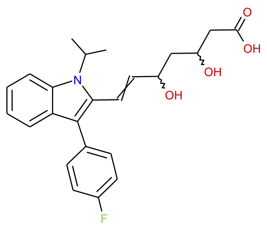	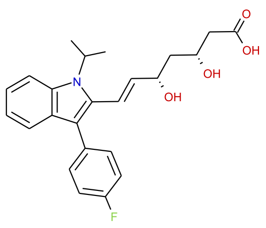	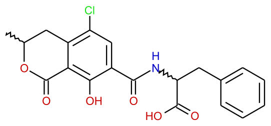
Actual Endpoint (-log C)	3.51742	3.51742	6.47264
Predicted Endpoint (-log C)	5.41573	5.41573	5.06501
Distance	0.597	0.597	0.666
Reference	CPDB	CPDB	CPDB

Model Applicability

Unknown features are fingerprint features in the query molecule, but not found in the training set.

1. All properties and OPS components are within expected ranges.
2. Unknown FCFP_2 feature: -1029533685: [*]:[c](:[*])C(F)(F)F

Feature Contribution

Top features for positive contribution

Fingerprint	Bit/Smiles	Feature Structure	Score
FCFP_6	1	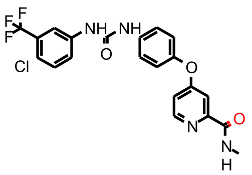 [*]=O	0.234

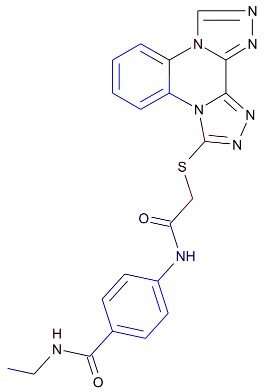

$C_{21}H_{18}N_8O_2S$
Molecular Weight: 446.48502
ALogP: 2.442
Rotatable Bonds: 6
Acceptors: 7
Donors: 2

Model Prediction

Prediction: 0.153
Unit: g/kg_body_weight
Mahalanobis Distance: 40.244
Mahalanobis Distance p-value: 1.44e-042

Mahalanobis Distance: The Mahalanobis distance (MD) is a generalization of the Euclidean distance that accounts for correlations among the X properties. It is calculated as the distance to the center of the training data. The larger the MD, the less trustworthy the prediction.
Mahalanobis Distance p-value: The p-value gives the fraction of training data with an MD greater than or equal to the one for the given sample, assuming normally distributed data. The smaller the p-value, the less trustworthy the prediciton. For highly non-normal X properties (e.g., fingerprints), the MD p-value is wildly inaccurate.

Structural Similar Compounds

Name	C.I.PIGMENT RED 23	C.I. ACID RED 14	C.I. ACID ORANGE 10
Structure	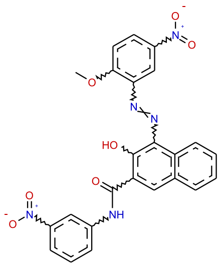	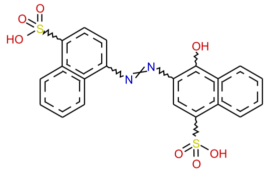	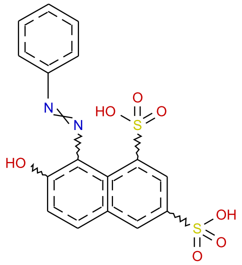
Actual Endpoint (-log C)	2.28997	2.8654	3.435
Predicted Endpoint (-log C)	3.52921	3.29295	3.28295
Distance	0.791	0.795	0.891
Reference	NTP 411 146	NTP REPORT # 220	NTP REPORT # 211

Model Applicability

Unknown features are fingerprint features in the query molecule, but not found in the training set.

1. All properties and OPS components are within expected ranges.
2. Unknown ECFP_6 feature: 672362763: [*]:n(:[*]):[*]
3. Unknown ECFP_6 feature: -830332112: [*]S[*]
4. Unknown ECFP_6 feature: -955816473: [*]SCC(=[*])[*]
5. Unknown ECFP_6 feature: 1731843802: [*]CC(=O)N[*]
6. Unknown ECFP_6 feature: -177077903: [*]N[c](:[cH]:[*]):[cH]:[*]
7. Unknown ECFP_6 feature: -175146122: [*]C(=[*])[c](:[cH]:[*]):[cH]:[*]
8. Unknown ECFP_6 feature: 1430169877: [*]NC(=O)[c](:[*]):[*]
9. Unknown ECFP_6 feature: 497523368: [*]CNC(=[*])[*]
10. Unknown ECFP_6 feature: -950223878: [*]NCC
11. Unknown ECFP_6 feature: 1997021792: [*]:[cH]:[cH]:[cH]:[*]
12. Unknown ECFP_6 feature: 1334415134: [*]:[cH]:[c](:n(:[*]):[*]):[c](:[*]):[*]
13. Unknown ECFP_6 feature: -1221742244: [*]:[c](:[*]):n1:[cH]:[*]:[*]:[c]:1:[*]
14. Unknown ECFP_6 feature: 1049075205: [*]:n1:[*]:[*]:n:[c]:1:[c](:[*]):[*]
15. Unknown ECFP_6 feature: 77911192: [*][c]1:[*]:[*]:[c](:[*]):n:1:[c](:[*]):[*]
16. Unknown ECFP_6 feature: 911256832: [*]:[c]1:[*]:[*]:n:n:1
17. Unknown ECFP_6 feature: 1986731747: [*]S[c]1:n:[*]:[*]:n:1:[*]
18. Unknown ECFP_6 feature: -708878603: [*]:n1:[*]:[*]:n:[cH]:1
19. Unknown ECFP_6 feature: -677914300: [*]1:[*]:n:n:[cH]:1
20. Unknown ECFP_6 feature: 1427820655: [*]CS[c](:[*]):[*]

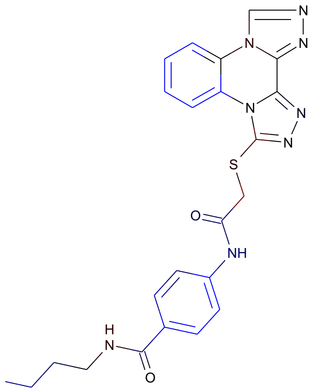

$C_{23}H_{22}N_8O_2S$
Molecular Weight: 474.53818
ALogP: 3.422
Rotatable Bonds: 8
Acceptors: 7
Donors: 2

Model Prediction

Prediction: 0.329
Unit: g/kg_body_weight
Mahalanobis Distance: 40.274
Mahalanobis Distance p-value: 1.29e-042

Mahalanobis Distance: The Mahalanobis distance (MD) is a generalization of the Euclidean distance that accounts for correlations among the X properties. It is calculated as the distance to the center of the training data. The larger the MD, the less trustworthy the prediction.
Mahalanobis Distance p-value: The p-value gives the fraction of training data with an MD greater than or equal to the one for the given sample, assuming normally distributed data. The smaller the p-value, the less trustworthy the prediciton. For highly non-normal X properties (e.g., fingerprints), the MD p-value is wildly inaccurate.

Structural Similar Compounds

Name	C.I.PIGMENT RED 23	C.I. ACID RED 14	DIARYLANILIDE YELLOW
Structure	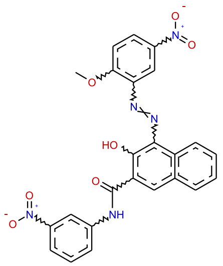	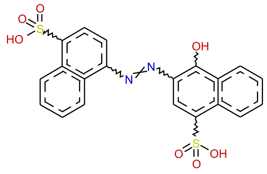	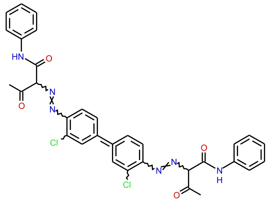
Actual Endpoint (-log C)	2.28997	2.8654	2.70208
Predicted Endpoint (-log C)	3.52921	3.29295	3.76154
Distance	0.741	0.818	0.890
Reference	NTP 411 146	NTP REPORT # 220	NTP 30 C-4

Model Applicability

Unknown features are fingerprint features in the query molecule, but not found in the training set.

1. All properties and OPS components are within expected ranges.
2. Unknown ECFP_6 feature: 672362763: [*]:n(:[*]):[*]
3. Unknown ECFP_6 feature: -830332112: [*]S[*]
4. Unknown ECFP_6 feature: -955816473: [*]SCC(=[*])[*]
5. Unknown ECFP_6 feature: 1731843802: [*]CC(=O)N[*]
6. Unknown ECFP_6 feature: -177077903: [*]N[c](:[cH]:[*]):[cH]:[*]
7. Unknown ECFP_6 feature: -175146122: [*]C(=[*])[c](:[cH]:[*]):[cH]:[*]
8. Unknown ECFP_6 feature: 1430169877: [*]NC(=O)[c](:[*]):[*]
9. Unknown ECFP_6 feature: 497523368: [*]CNC(=[*])[*]
10. Unknown ECFP_6 feature: -1791034651: [*]CCN[*]
11. Unknown ECFP_6 feature: 1997021792: [*]:[cH]:[cH]:[cH]:[*]
12. Unknown ECFP_6 feature: 1334415134: [*]:[cH]:[c](:n(:[*]):[*]):[c](:[*]):[*]
13. Unknown ECFP_6 feature: -1221742244: [*]:[c](:[*]):n1:[cH]:[*]:[*]:[c]:1:[*]
14. Unknown ECFP_6 feature: 1049075205: [*]:n1:[*]:[*]:n:[c]:1:[c](:[*]):[*]
15. Unknown ECFP_6 feature: 77911192: [*][c]1:[*]:[*]:[c](:[*]):n:1:[c](:[*]):[*]
16. Unknown ECFP_6 feature: 911256832: [*]:[c]1:[*]:[*]:n:n:1
17. Unknown ECFP_6 feature: 1986731747: [*]S[c]1:n:[*]:[*]:n:1:[*]
18. Unknown ECFP_6 feature: -708878603: [*]:n1:[*]:[*]:n:[cH]:1
19. Unknown ECFP_6 feature: -677914300: [*]1:[*]:n:n:[cH]:1
20. Unknown ECFP_6 feature: 1427820655: [*]CS[c](:[*]):[*]
21. Unknown ECFP_6 feature: -1793471910: [*]CCC

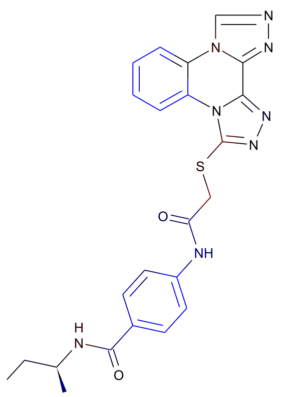

$C_{23}H_{22}N_8O_2S$
Molecular Weight: 474.53818
ALogP: 3.343
Rotatable Bonds: 7
Acceptors: 7
Donors: 2

Model Prediction

Prediction: 0.206
Unit: g/kg_body_weight
Mahalanobis Distance: 42.675
Mahalanobis Distance p-value: 1.63e-046

Mahalanobis Distance: The Mahalanobis distance (MD) is a generalization of the Euclidean distance that accounts for correlations among the X properties. It is calculated as the distance to the center of the training data. The larger the MD, the less trustworthy the prediction.
Mahalanobis Distance p-value: The p-value gives the fraction of training data with an MD greater than or equal to the one for the given sample, assuming normally distributed data. The smaller the p-value, the less trustworthy the prediciton. For highly non-normal X properties (e.g., fingerprints), the MD p-value is wildly inaccurate.

Structural Similar Compounds

Name	C.I.PIGMENT RED 23	C.I. ACID RED 14	C.I. ACID ORANGE 3
Structure	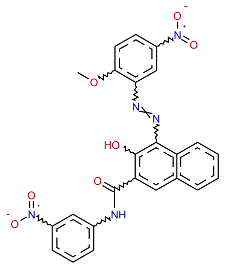	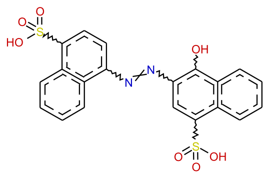	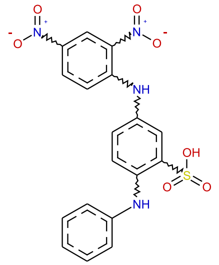
Actual Endpoint (-log C)	2.28997	2.8654	3.20573
Predicted Endpoint (-log C)	3.52921	3.29295	3.55956
Distance	0.736	0.792	0.885
Reference	NTP 411 146	NTP REPORT # 220	NTP REPORT # 335

Model Applicability

Unknown features are fingerprint features in the query molecule, but not found in the training set.

1. All properties and OPS components are within expected ranges.
2. Unknown ECFP_6 feature: 672362763: [*]:n(:[*]):[*]
3. Unknown ECFP_6 feature: -830332112: [*]S[*]
4. Unknown ECFP_6 feature: -955816473: [*]SCC(=[*])[*]
5. Unknown ECFP_6 feature: 1731843802: [*]CC(=O)N[*]
6. Unknown ECFP_6 feature: -177077903: [*]N[c](:[cH]:[*]):[cH]:[*]
7. Unknown ECFP_6 feature: -175146122: [*]C(=[*])[c](:[cH]:[*]):[cH]:[*]
8. Unknown ECFP_6 feature: 1430169877: [*]NC(=O)[c](:[*]):[*]
9. Unknown ECFP_6 feature: 975766354: [*]C([*])NC(=[*])[*]
10. Unknown ECFP_6 feature: 1037602861: [*]C[C@H](C)N[*]
11. Unknown ECFP_6 feature: 1997021792: [*]:[cH]:[cH]:[cH]:[*]
12. Unknown ECFP_6 feature: 1334415134: [*]:[cH]:[c](:n(:[*]):[*]):[c](:[*]):[*]
13. Unknown ECFP_6 feature: -1221742244: [*]:[c](:[*]):n1:[cH]:[*]:[*]:[c]:1:[*]
14. Unknown ECFP_6 feature: 1049075205: [*]:n1:[*]:[*]:n:[c]:1:[c](:[*]):[*]
15. Unknown ECFP_6 feature: 77911192: [*][c]1:[*]:[*]:[c](:[*]):n:1:[c](:[*]):[*]
16. Unknown ECFP_6 feature: 911256832: [*]:[c]1:[*]:[*]:n:n:1
17. Unknown ECFP_6 feature: 1986731747: [*]S[c]1:n:[*]:[*]:n:1:[*]
18. Unknown ECFP_6 feature: -708878603: [*]:n1:[*]:[*]:n:[cH]:1
19. Unknown ECFP_6 feature: -677914300: [*]1:[*]:n:n:[cH]:1
20. Unknown ECFP_6 feature: 1427820655: [*]CS[c](:[*]):[*]
21. Unknown ECFP_6 feature: -949992060: [*]C([*])CC

ter. butyl.cdx

TOPKAT_Chronic_LOAEL

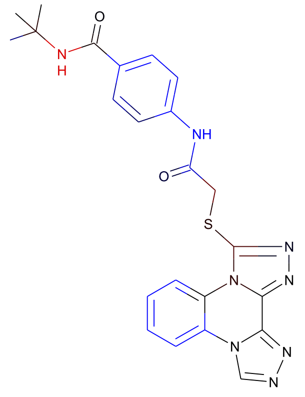

$C_{23}H_{22}N_8O_2S$

Molecular Weight: 474.53818

ALogP: 3.025

Rotatable Bonds: 6

Acceptors: 7

Donors: 2

Model Prediction

Prediction: 0.084

Unit: g/kg_body_weight

Mahalanobis Distance: 43.932

Mahalanobis Distance p-value: 1.66e-048

Mahalanobis Distance: The Mahalanobis distance (MD) is a generalization of the Euclidean distance that accounts for correlations among the X properties. It is calculated as the distance to the center of the training data. The larger the MD, the less trustworthy the prediction.
Mahalanobis Distance p-value: The p-value gives the fraction of training data with an MD greater than or equal to the one for the given sample, assuming normally distributed data. The smaller the p-value, the less trustworthy the prediciton. For highly non-normal X properties (e.g., fingerprints), the MD p-value is wildly inaccurate.

Structural Similar Compounds

Name	C.I.PIGMENT RED 23	C.I. ACID RED 14	C.I. ACID ORANGE 3
Structure	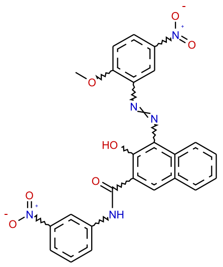	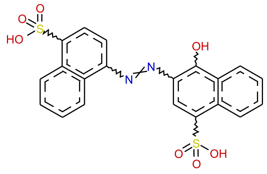	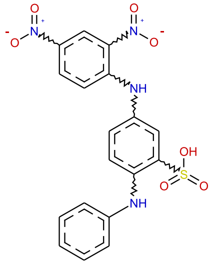
Actual Endpoint (-log C)	2.28997	2.8654	3.20573
Predicted Endpoint (-log C)	3.52921	3.29295	3.55956
Distance	0.753	0.779	0.889
Reference	NTP 411 146	NTP REPORT # 220	NTP REPORT # 335

Model Applicability

Unknown features are fingerprint features in the query molecule, but not found in the training set.

1. OPS PC35 out of range. Value: -4.1147. Training min, max, SD, explained variance: -3.6414, 3.2579, 1.162, 0.0059.
2. Unknown ECFP_6 feature: -830332112: [*]S[*]
3. Unknown ECFP_6 feature: 672362763: [*]:n(:[*]):[*]
4. Unknown ECFP_6 feature: 1430169877: [*]NC(=O)[c](:[*]):[*]
5. Unknown ECFP_6 feature: -175146122: [*]C(=[*])[c](:[cH]:[*]):[cH]:[*]
6. Unknown ECFP_6 feature: -177077903: [*]N[c](:[cH]:[*]):[cH]:[*]
7. Unknown ECFP_6 feature: 16744893: [*]C(=[*])NC([*])([*])[*]
8. Unknown ECFP_6 feature: -1623733623: [*]NC(C)(C)C
9. Unknown ECFP_6 feature: 865857320: [*]C([*])([*])C
10. Unknown ECFP_6 feature: 1731843802: [*]CC(=O)N[*]
11. Unknown ECFP_6 feature: -955816473: [*]SCC(=[*])[*]
12. Unknown ECFP_6 feature: 1427820655: [*]CS[c](:[*]):[*]
13. Unknown ECFP_6 feature: 77911192: [*][c]1:[*]:[*]:[c](:[*]):n:1:[c](:[*]):[*]
14. Unknown ECFP_6 feature: 1049075205: [*]:n1:[*]:[*]:n:[c]:1:[c](:[*]):[*]
15. Unknown ECFP_6 feature: -1221742244: [*]:[c](:[*]):n1:[cH]:[*]:[*]:[c]:1:[*]
16. Unknown ECFP_6 feature: 1334415134: [*]:[cH]:[c](:n(:[*]):[*]):[c](:[*]):[*]
17. Unknown ECFP_6 feature: 1997021792: [*]:[cH]:[cH]:[cH]:[*]
18. Unknown ECFP_6 feature: 911256832: [*][c]1:[*]:[*]:n:n:1
19. Unknown ECFP_6 feature: 1986731747: [*]S[c]1:n:[*]:[*]:n:1:[*]
20. Unknown ECFP_6 feature: -677914300: [*]1:[*]:n:n:[cH]:1

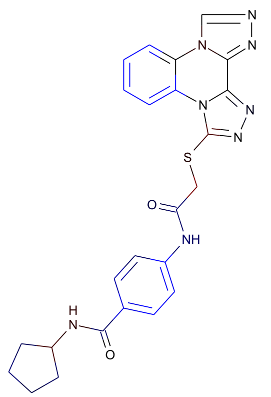

$C_{24}H_{22}N_8O_2S$
Molecular Weight: 486.54888
ALogP: 3.496
Rotatable Bonds: 6
Acceptors: 7
Donors: 2

Structural Similar Compounds

Name	C.I.PIGMENT RED 23	C.I. ACID RED 14	C.I. ACID ORANGE 3
Structure	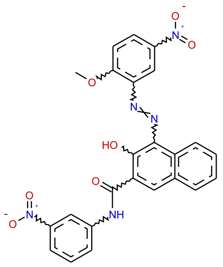	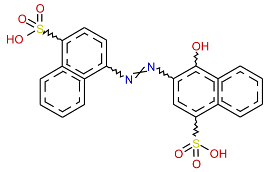	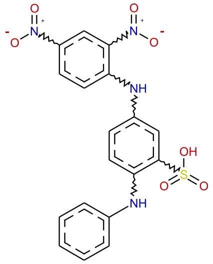
Actual Endpoint (-log C)	2.28997	2.8654	3.20573
Predicted Endpoint (-log C)	3.52921	3.29295	3.55956
Distance	0.737	0.779	0.894
Reference	NTP 411 146	NTP REPORT # 220	NTP REPORT # 335

Model Prediction

Prediction: 0.221
Unit: g/kg_body_weight
Mahalanobis Distance: 44.610
Mahalanobis Distance p-value: 1.45e-049

Mahalanobis Distance: The Mahalanobis distance (MD) is a generalization of the Euclidean distance that accounts for correlations among the X properties. It is calculated as the distance to the center of the training data. The larger the MD, the less trustworthy the prediction.
Mahalanobis Distance p-value: The p-value gives the fraction of training data with an MD greater than or equal to the one for the given sample, assuming normally distributed data. The smaller the p-value, the less trustworthy the prediciton. For highly non-normal X properties (e.g., fingerprints), the MD p-value is wildly inaccurate.

Model Applicability

Unknown features are fingerprint features in the query molecule, but not found in the training set.

1. OPS PC35 out of range. Value: -4.5348. Training min, max, SD, explained variance: -3.6414, 3.2579, 1.162, 0.0059.
2. Unknown ECFP_6 feature: 672362763: [*]:n(:[*]):[*]
3. Unknown ECFP_6 feature: -830332112: [*]S[*]
4. Unknown ECFP_6 feature: -955816473: [*]SCC(=[*])[*]
5. Unknown ECFP_6 feature: 1731843802: [*]CC(=O)N[*]
6. Unknown ECFP_6 feature: -177077903: [*]N[c](:[cH]:[*]):[cH]:[*]
7. Unknown ECFP_6 feature: -175146122: [*]C(=[*])[c](:[cH]:[*]):[cH]:[*]
8. Unknown ECFP_6 feature: 1430169877: [*]NC(=O)[c](:[*]):[*]
9. Unknown ECFP_6 feature: -2091181441: [*]C([*])NC(=[*])[*]
10. Unknown ECFP_6 feature: -859078569: [*]NC1C[*][*]C1
11. Unknown ECFP_6 feature: 1997021792: [*]:[cH]:[cH]:[cH]:[*]
12. Unknown ECFP_6 feature: 1334415134: [*]:[cH]:[c](:n(:[*]):[*]):[c](:[*]):[*]
13. Unknown ECFP_6 feature: -1221742244: [*]:[c](:[*]):n1:[cH]:[*]:[*]:[c]:1:[*]
14. Unknown ECFP_6 feature: 1049075205: [*]:n1:[*]:[*]:n:[c]:1:[c](:[*]):[*]
15. Unknown ECFP_6 feature: 77911192: [*][c]1:[*]:[*]:[c](:[*]):n:1:[c](:[*]):[*]
16. Unknown ECFP_6 feature: 911256832: [*]:[c]1:[*]:[*]:n:n:1
17. Unknown ECFP_6 feature: 1986731747: [*]S[c]1:n:[*]:[*]:n:1:[*]
18. Unknown ECFP_6 feature: -708878603: [*]:n1:[*]:[*]:n:[cH]:1
19. Unknown ECFP_6 feature: -677914300: [*]1:[*]:n:n:[cH]:1
20. Unknown ECFP_6 feature: 1427820655: [*]CS[c](:[*]):[*]

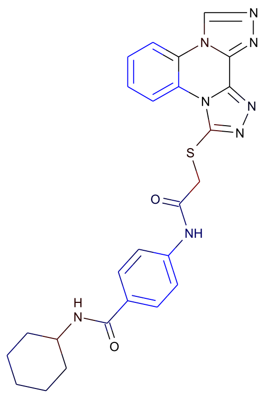

$C_{25}H_{24}N_8O_2S$

Molecular Weight: 500.57546

ALogP: 3.952

Rotatable Bonds: 6

Acceptors: 7

Donors: 2

Model Prediction

Prediction: 0.192

Unit: g/kg_body_weight

Mahalanobis Distance: 44.354

Mahalanobis Distance p-value: 3.62e-049

Mahalanobis Distance: The Mahalanobis distance (MD) is a generalization of the Euclidean distance that accounts for correlations among the X properties. It is calculated as the distance to the center of the training data. The larger the MD, the less trustworthy the prediction.
Mahalanobis Distance p-value: The p-value gives the fraction of training data with an MD greater than or equal to the one for the given sample, assuming normally distributed data. The smaller the p-value, the less trustworthy the prediciton. For highly non-normal X properties (e.g., fingerprints), the MD p-value is wildly inaccurate.

Structural Similar Compounds

Name	C.I.PIGMENT RED 23	C.I. ACID RED 14	DIARYLANILIDE YELLOW
Structure	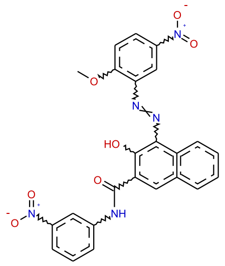	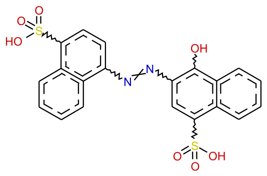	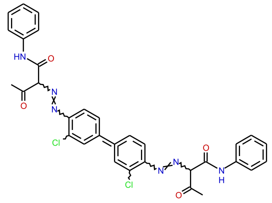
Actual Endpoint (-log C)	2.28997	2.8654	2.70208
Predicted Endpoint (-log C)	3.52921	3.29295	3.76154
Distance	0.721	0.780	0.880
Reference	NTP 411 146	NTP REPORT # 220	NTP 30 C-4

Model Applicability

Unknown features are fingerprint features in the query molecule, but not found in the training set.

1. OPS PC35 out of range. Value: -4.574. Training min, max, SD, explained variance: -3.6414, 3.2579, 1.162, 0.0059.
2. Unknown ECFP_6 feature: 672362763: [*]:n(:[*]):[*]
3. Unknown ECFP_6 feature: -830332112: [*]S[*]
4. Unknown ECFP_6 feature: -955816473: [*]SCC(=[*])[*]
5. Unknown ECFP_6 feature: 1731843802: [*]CC(=O)N[*]
6. Unknown ECFP_6 feature: -177077903: [*]N[c](:[cH]:[*]):[cH]:[*]
7. Unknown ECFP_6 feature: -175146122: [*]C(=[*])[c](:[cH]:[*]):[cH]:[*]
8. Unknown ECFP_6 feature: 1430169877: [*]NC(=O)[c](:[*]):[*]
9. Unknown ECFP_6 feature: -2091181441: [*]C([*])NC(=[*])[*]
10. Unknown ECFP_6 feature: -859078569: [*]NC1C[*][*]C1
11. Unknown ECFP_6 feature: 1997021792: [*]:[cH]:[cH]:[cH]:[*]
12. Unknown ECFP_6 feature: 1334415134: [*]:[cH]:[c](:n(:[*]):[*]):[c](:[*]):[*]
13. Unknown ECFP_6 feature: -1221742244: [*]:[c](:[*]):n1:[cH]:[*]:[*]:[c]:1:[*]
14. Unknown ECFP_6 feature: 1049075205: [*]:n1:[*]:[*]:n:[c]:1:[c](:[*]):[*]
15. Unknown ECFP_6 feature: 77911192: [*][c]1:[*]:[*]:[c](:[*]):n:1:[c](:[*]):[*]
16. Unknown ECFP_6 feature: 911256832: [*]:[c]1:[*]:[*]:n:n:1
17. Unknown ECFP_6 feature: 1986731747: [*]S[c]1:n:[*]:[*]:n:1:[*]
18. Unknown ECFP_6 feature: -708878603: [*]:n1:[*]:[*]:n:[cH]:1
19. Unknown ECFP_6 feature: -677914300: [*]1:[*]:n:n:[cH]:1
20. Unknown ECFP_6 feature: 1427820655: [*]CS[c](:[*]):[*]

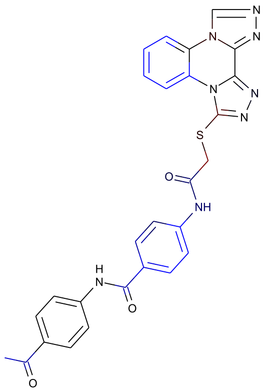

$C_{27}H_{20}N_8O_3S$
Molecular Weight: 536.56449
ALogP: 3.41
Rotatable Bonds: 7
Acceptors: 8
Donors: 2

Model Prediction

Prediction: 0.094
Unit: g/kg_body_weight
Mahalanobis Distance: 41.830
Mahalanobis Distance p-value: 3.71e-045

Mahalanobis Distance: The Mahalanobis distance (MD) is a generalization of the Euclidean distance that accounts for correlations among the X properties. It is calculated as the distance to the center of the training data. The larger the MD, the less trustworthy the prediction.
Mahalanobis Distance p-value: The p-value gives the fraction of training data with an MD greater than or equal to the one for the given sample, assuming normally distributed data. The smaller the p-value, the less trustworthy the prediciton. For highly non-normal X properties (e.g., fingerprints), the MD p-value is wildly inaccurate.

Structural Similar Compounds

Name	C.I.PIGMENT RED 23	C.I. ACID RED 14	DIARYLANILIDE YELLOW
Structure	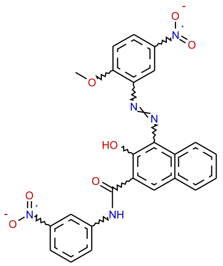	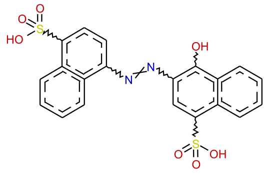	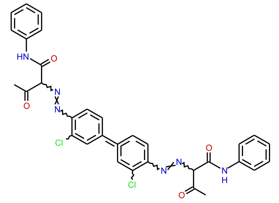
Actual Endpoint (-log C)	2.28997	2.8654	2.70208
Predicted Endpoint (-log C)	3.52921	3.29295	3.76154
Distance	0.793	0.881	0.924
Reference	NTP 411 146	NTP REPORT # 220	NTP 30 C-4

Model Applicability

Unknown features are fingerprint features in the query molecule, but not found in the training set.

1. OPS PC35 out of range. Value: -4.4005. Training min, max, SD, explained variance: -3.6414, 3.2579, 1.162, 0.0059.
2. Unknown ECFP_6 feature: 672362763: [*]:n(:[*]):[*]
3. Unknown ECFP_6 feature: -830332112: [*]S[*]
4. Unknown ECFP_6 feature: -955816473: [*]SCC(=[*])[*]
5. Unknown ECFP_6 feature: 1731843802: [*]CC(=O)N[*]
6. Unknown ECFP_6 feature: -177077903: [*]N[c](:[cH]:[*]):[cH]:[*]
7. Unknown ECFP_6 feature: -175146122: [*]C(=[*])[c](:[cH]:[*]):[cH]:[*]
8. Unknown ECFP_6 feature: 1430169877: [*]NC(=O)[c](:[*]):[*]
9. Unknown ECFP_6 feature: 1997021792: [*]:[cH]:[cH]:[cH]:[*]
10. Unknown ECFP_6 feature: 1334415134: [*]:[cH]:[c](:n(:[*]):[*]):[c](:[*]):[*]
11. Unknown ECFP_6 feature: -1221742244: [*]:[c](:[*]):n1:[cH]:[*]:[*]:[c]:1:[*]
12. Unknown ECFP_6 feature: 1049075205: [*]:n1:[*]:[*]:n:[c]:1:[c](:[*]):[*]
13. Unknown ECFP_6 feature: 77911192: [*][c]1:[*]:[*]:[c](:[*]):n:1:[c](:[*]):[*]
14. Unknown ECFP_6 feature: 911256832: [*]:[c]1:[*]:[*]:n:n:1
15. Unknown ECFP_6 feature: 1986731747: [*]S[c]1:n:[*]:[*]:n:1:[*]
16. Unknown ECFP_6 feature: -708878603: [*]:n1:[*]:[*]:n:[cH]:1
17. Unknown ECFP_6 feature: -677914300: [*]1:[*]:n:n:[cH]:1
18. Unknown ECFP_6 feature: 1427820655: [*]CS[c](:[*]):[*]
19. Unknown ECFP_6 feature: 129482634: [*]:[c](:[*])C(=O)C

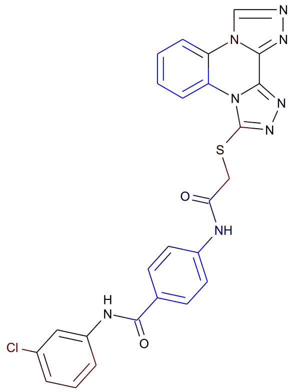

$C_{25}H_{17}ClN_8O_2S$
Molecular Weight: 528.97287
ALogP: 4.334
Rotatable Bonds: 6
Acceptors: 7
Donors: 2

Model Prediction

Prediction: 0.072
Unit: g/kg_body_weight
Mahalanobis Distance: 40.944
Mahalanobis Distance p-value: 1.02e-043

Mahalanobis Distance: The Mahalanobis distance (MD) is a generalization of the Euclidean distance that accounts for correlations among the X properties. It is calculated as the distance to the center of the training data. The larger the MD, the less trustworthy the prediction.
Mahalanobis Distance p-value: The p-value gives the fraction of training data with an MD greater than or equal to the one for the given sample, assuming normally distributed data. The smaller the p-value, the less trustworthy the prediciton. For highly non-normal X properties (e.g., fingerprints), the MD p-value is wildly inaccurate.

Structural Similar Compounds

Name	C.I.PIGMENT RED 23	C.I. ACID RED 14	DIARYLANILIDE YELLOW
Structure	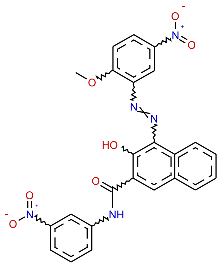	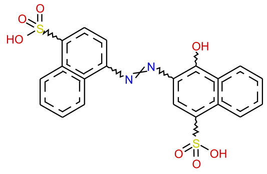	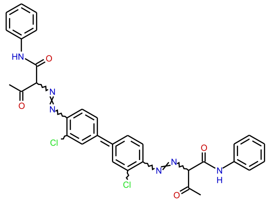
Actual Endpoint (-log C)	2.28997	2.8654	2.70208
Predicted Endpoint (-log C)	3.52921	3.29295	3.76154
Distance	0.819	0.892	0.903
Reference	NTP 411 146	NTP REPORT # 220	NTP 30 C-4

Model Applicability

Unknown features are fingerprint features in the query molecule, but not found in the training set.

1. All properties and OPS components are within expected ranges.
2. Unknown ECFP_6 feature: 672362763: [*]:n(:[*]):[*]
3. Unknown ECFP_6 feature: -830332112: [*]S[*]
4. Unknown ECFP_6 feature: -955816473: [*]SCC(=[*])[*]
5. Unknown ECFP_6 feature: 1731843802: [*]CC(=O)N[*]
6. Unknown ECFP_6 feature: -177077903: [*]N[c](:[cH]:[*]):[cH]:[*]
7. Unknown ECFP_6 feature: -175146122: [*]C(=[*])[c](:[cH]:[*]):[cH]:[*]
8. Unknown ECFP_6 feature: 1430169877: [*]NC(=O)[c](:[*]):[*]
9. Unknown ECFP_6 feature: 1997021792: [*]:[cH]:[cH]:[cH]:[*]
10. Unknown ECFP_6 feature: 1334415134: [*]:[cH]:[c](:n(:[*]):[*]):[c](:[*]):[*]
11. Unknown ECFP_6 feature: -1221742244: [*]:[c](:[*]):n1:[cH]:[*]:[*]:[c]:1:[*]
12. Unknown ECFP_6 feature: 1049075205: [*]:n1:[*]:[*]:n:[c]:1:[c](:[*]):[*]
13. Unknown ECFP_6 feature: 77911192: [*][c]1:[*]:[*]:[c](:[*]):n:1:[c](:[*]):[*]
14. Unknown ECFP_6 feature: 911256832: [*]:[c]1:[*]:[*]:n:n:1
15. Unknown ECFP_6 feature: 1986731747: [*]S[c]1:n:[*]:[*]:n:1:[*]
16. Unknown ECFP_6 feature: -708878603: [*]:n1:[*]:[*]:n:[cH]:1
17. Unknown ECFP_6 feature: -677914300: [*]1:[*]:n:n:[cH]:1
18. Unknown ECFP_6 feature: 1427820655: [*]CS[c](:[*]):[*]
19. Unknown ECFP_6 feature: -176494269: [*]:[cH]:[c](Cl):[cH]:[*]
20. Unknown ECFP_6 feature: 99947387: [*]:[c](:[*])Cl

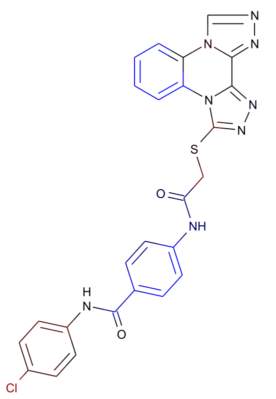

$C_{25}H_{17}ClN_8O_2S$
Molecular Weight: 528.97287
ALogP: 4.334
Rotatable Bonds: 6
Acceptors: 7
Donors: 2

Model Prediction

Prediction: 0.067
Unit: g/kg_body_weight
Mahalanobis Distance: 40.944
Mahalanobis Distance p-value: 1.02e-043

Mahalanobis Distance: The Mahalanobis distance (MD) is a generalization of the Euclidean distance that accounts for correlations among the X properties. It is calculated as the distance to the center of the training data. The larger the MD, the less trustworthy the prediction.
Mahalanobis Distance p-value: The p-value gives the fraction of training data with an MD greater than or equal to the one for the given sample, assuming normally distributed data. The smaller the p-value, the less trustworthy the prediciton. For highly non-normal X properties (e.g., fingerprints), the MD p-value is wildly inaccurate.

Structural Similar Compounds

Name	C.I.PIGMENT RED 23	C.I. ACID RED 14	DIARYLANILIDE YELLOW
Structure	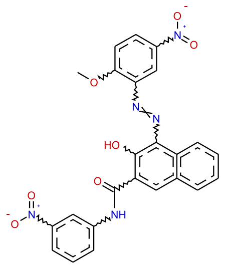	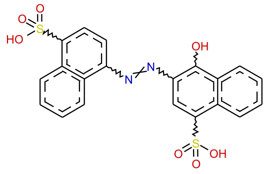	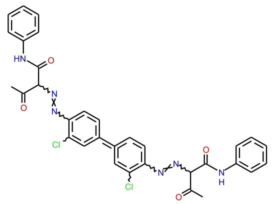
Actual Endpoint (-log C)	2.28997	2.8654	2.70208
Predicted Endpoint (-log C)	3.52921	3.29295	3.76154
Distance	0.825	0.893	0.907
Reference	NTP 411 146	NTP REPORT # 220	NTP 30 C-4

Model Applicability

Unknown features are fingerprint features in the query molecule, but not found in the training set.

1. All properties and OPS components are within expected ranges.
2. Unknown ECFP_6 feature: 672362763: [*]:n(:[*]):[*]
3. Unknown ECFP_6 feature: -830332112: [*]S[*]
4. Unknown ECFP_6 feature: -955816473: [*]SCC(=[*])[*]
5. Unknown ECFP_6 feature: 1731843802: [*]CC(=O)N[*]
6. Unknown ECFP_6 feature: -177077903: [*]N[c](:[cH]:[*]):[cH]:[*]
7. Unknown ECFP_6 feature: -175146122: [*]C(=[*])[c](:[cH]:[*]):[cH]:[*]
8. Unknown ECFP_6 feature: 1430169877: [*]NC(=O)[c](:[*]):[*]
9. Unknown ECFP_6 feature: 1997021792: [*]:[cH]:[cH]:[cH]:[*]
10. Unknown ECFP_6 feature: 1334415134: [*]:[cH]:[c](:n(:[*]):[*]):[c](:[*]):[*]
11. Unknown ECFP_6 feature: -1221742244: [*]:[c](:[*]):n1:[cH]:[*]:[*]:[c]:1:[*]
12. Unknown ECFP_6 feature: 1049075205: [*]:n1:[*]:[*]:n:[c]:1:[c](:[*]):[*]
13. Unknown ECFP_6 feature: 77911192: [*][c]1:[*]:[*]:[c](:[*]):n:1:[c](:[*]):[*]
14. Unknown ECFP_6 feature: 911256832: [*]:[c]1:[*]:[*]:n:n:1
15. Unknown ECFP_6 feature: 1986731747: [*]S[c]1:n:[*]:[*]:n:1:[*]
16. Unknown ECFP_6 feature: -708878603: [*]:n1:[*]:[*]:n:[cH]:1
17. Unknown ECFP_6 feature: -677914300: [*]1:[*]:n:n:[cH]:1
18. Unknown ECFP_6 feature: 1427820655: [*]CS[c](:[*]):[*]
19. Unknown ECFP_6 feature: -176494269: [*]:[cH]:[c](Cl):[cH]:[*]
20. Unknown ECFP_6 feature: 99947387: [*]:[c](:[*])Cl

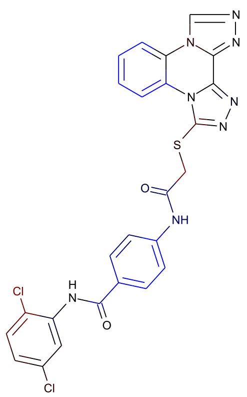

$C_{25}H_{16}Cl_2N_8O_2S$
Molecular Weight: 563.41793
ALogP: 4.999
Rotatable Bonds: 6
Acceptors: 7
Donors: 2

Model Prediction

Prediction: 0.064
Unit: g/kg_body_weight
Mahalanobis Distance: 40.555
Mahalanobis Distance p-value: 4.43e-043

Mahalanobis Distance: The Mahalanobis distance (MD) is a generalization of the Euclidean distance that accounts for correlations among the X properties. It is calculated as the distance to the center of the training data. The larger the MD, the less trustworthy the prediction.
Mahalanobis Distance p-value: The p-value gives the fraction of training data with an MD greater than or equal to the one for the given sample, assuming normally distributed data. The smaller the p-value, the less trustworthy the prediciton. For highly non-normal X properties (e.g., fingerprints), the MD p-value is wildly inaccurate.

Structural Similar Compounds

Name	C.I.PIGMENT RED 23	DIARYLANILIDE YELLOW	C.I. ACID RED 14
Structure	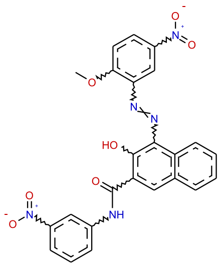	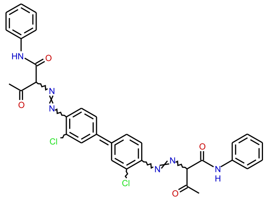	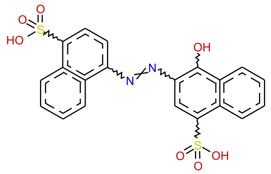
Actual Endpoint (-log C)	2.28997	2.70208	2.8654
Predicted Endpoint (-log C)	3.52921	3.76154	3.29295
Distance	0.828	0.865	0.910
Reference	NTP 411 146	NTP 30 C-4	NTP REPORT # 220

Model Applicability

Unknown features are fingerprint features in the query molecule, but not found in the training set.

1. All properties and OPS components are within expected ranges.
2. Unknown ECFP_6 feature: 672362763: [*]:n(:[*]):[*]
3. Unknown ECFP_6 feature: -830332112: [*]S[*]
4. Unknown ECFP_6 feature: -955816473: [*]SCC(=[*])[*]
5. Unknown ECFP_6 feature: 1731843802: [*]CC(=O)N[*]
6. Unknown ECFP_6 feature: -177077903: [*]N[c](:[cH]:[*]):[cH]:[*]
7. Unknown ECFP_6 feature: -175146122: [*]C(=[*])[c](:[cH]:[*]):[cH]:[*]
8. Unknown ECFP_6 feature: 1430169877: [*]NC(=O)[c](:[*]):[*]
9. Unknown ECFP_6 feature: 1335108269: [*]N[c](:[cH]:[*]):[c]([*]):[*]
10. Unknown ECFP_6 feature: 1997021792: [*]:[cH]:[cH]:[cH]:[*]
11. Unknown ECFP_6 feature: 1334415134: [*]:[cH]:[c](:n(:[*]):[*]):[c](:[*]):[*]
12. Unknown ECFP_6 feature: -1221742244: [*]:[c](:[*]):n1:[cH]:[*]:[*]:[c]:1:[*]
13. Unknown ECFP_6 feature: 1049075205: [*]:n1:[*]:[*]:n:[c]:1:[c](:[*]):[*]
14. Unknown ECFP_6 feature: 77911192: [*][c]1:[*]:[*]:[c](:[*]):n:1:[c](:[*]):[*]
15. Unknown ECFP_6 feature: 911256832: [*]:[c]1:[*]:[*]:n:n:1
16. Unknown ECFP_6 feature: 1986731747: [*]S[c]1:n:[*]:[*]:n:1:[*]
17. Unknown ECFP_6 feature: -708878603: [*]:n1:[*]:[*]:n:[cH]:1
18. Unknown ECFP_6 feature: -677914300: [*]1:[*]:n:n:[cH]:1
19. Unknown ECFP_6 feature: 1427820655: [*]CS[c](:[*]):[*]
20. Unknown ECFP_6 feature: -176494269: [*]:[cH]:[c](Cl):[cH]:[*]
21. Unknown ECFP_6 feature: 99947387: [*]:[c](:[*])Cl

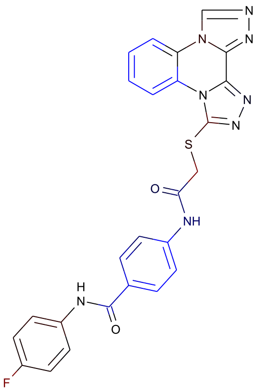

$C_{25}H_{17}FN_8O_2S$
Molecular Weight: 512.51828
ALogP: 3.875
Rotatable Bonds: 6
Acceptors: 7
Donors: 2

Model Prediction

Prediction: 0.073
Unit: g/kg_body_weight
Mahalanobis Distance: 40.988
Mahalanobis Distance p-value: 8.64e-044

Mahalanobis Distance: The Mahalanobis distance (MD) is a generalization of the Euclidean distance that accounts for correlations among the X properties. It is calculated as the distance to the center of the training data. The larger the MD, the less trustworthy the prediction.
Mahalanobis Distance p-value: The p-value gives the fraction of training data with an MD greater than or equal to the one for the given sample, assuming normally distributed data. The smaller the p-value, the less trustworthy the prediciton. For highly non-normal X properties (e.g., fingerprints), the MD p-value is wildly inaccurate.

Structural Similar Compounds

Name	C.I.PIGMENT RED 23	C.I. ACID RED 14	DIARYLANILIDE YELLOW
Structure	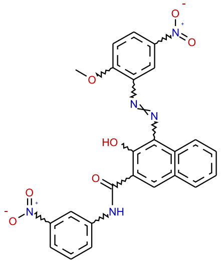	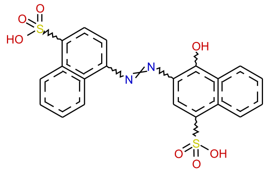	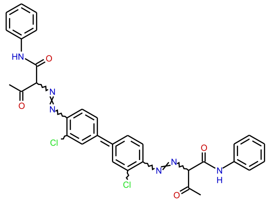
Actual Endpoint (-log C)	2.28997	2.8654	2.70208
Predicted Endpoint (-log C)	3.52921	3.29295	3.76154
Distance	0.832	0.891	0.938
Reference	NTP 411 146	NTP REPORT # 220	NTP 30 C-4

Model Applicability

Unknown features are fingerprint features in the query molecule, but not found in the training set.

1. OPS PC35 out of range. Value: -3.7174. Training min, max, SD, explained variance: -3.6414, 3.2579, 1.162, 0.0059.
2. Unknown ECFP_6 feature: 672362763: [*]:n(:[*]):[*]
3. Unknown ECFP_6 feature: -830332112: [*]S[*]
4. Unknown ECFP_6 feature: -1046436026: [*]F
5. Unknown ECFP_6 feature: -955816473: [*]SCC(=[*])[*]
6. Unknown ECFP_6 feature: 1731843802: [*]CC(=O)N[*]
7. Unknown ECFP_6 feature: -177077903: [*]N[c](:[cH]:[*]):[cH]:[*]
8. Unknown ECFP_6 feature: -175146122: [*]C(=[*])[c](:[cH]:[*]):[cH]:[*]
9. Unknown ECFP_6 feature: 1430169877: [*]NC(=O)[c](:[*]):[*]
10. Unknown ECFP_6 feature: 1997021792: [*]:[cH]:[cH]:[cH]:[*]
11. Unknown ECFP_6 feature: 1334415134: [*]:[cH]:[c](:n(:[*]):[*]):[c](:[*]):[*]
12. Unknown ECFP_6 feature: -1221742244: [*]:[c](:[*]):n1:[cH]:[*]:[*]:[c]:1:[*]
13. Unknown ECFP_6 feature: 1049075205: [*]:n1:[*]:[*]:n:[c]:1:[c](:[*]):[*]
14. Unknown ECFP_6 feature: 77911192: [*][c]1:[*]:[*]:[c](:[*]):n:1:[c](:[*]):[*]
15. Unknown ECFP_6 feature: 911256832: [*]:[c]1:[*]:[*]:n:n:1
16. Unknown ECFP_6 feature: 1986731747: [*]S[c]1:n:[*]:[*]:n:1:[*]
17. Unknown ECFP_6 feature: -708878603: [*]:n1:[*]:[*]:n:[cH]:1
18. Unknown ECFP_6 feature: -677914300: [*]1:[*]:n:n:[cH]:1
19. Unknown ECFP_6 feature: 1427820655: [*]CS[c](:[*]):[*]
20. Unknown ECFP_6 feature: -176686665: [*]:[cH]:[c](F):[cH]:[*]

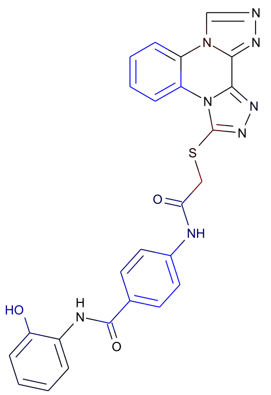

$C_{25}H_{18}N_8O_3S$

Molecular Weight: 510.52722

ALogP: 3.428

Rotatable Bonds: 6

Acceptors: 8

Donors: 3

Model Prediction

Prediction: 0.134

Unit: g/kg_body_weight

Mahalanobis Distance: 41.953

Mahalanobis Distance p-value: 2.35e-045

Mahalanobis Distance: The Mahalanobis distance (MD) is a generalization of the Euclidean distance that accounts for correlations among the X properties. It is calculated as the distance to the center of the training data. The larger the MD, the less trustworthy the prediction.

Mahalanobis Distance p-value: The p-value gives the fraction of training data with an MD greater than or equal to the one for the given sample, assuming normally distributed data. The smaller the p-value, the less trustworthy the prediciton. For highly non-normal X properties (e.g., fingerprints), the MD p-value is wildly inaccurate.

Structural Similar Compounds

Name	C.I.PIGMENT RED 23	C.I. ACID RED 14	C.I. ACID ORANGE 3
Structure	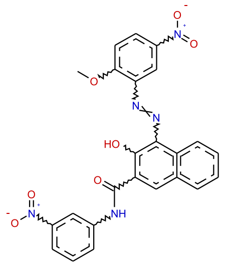	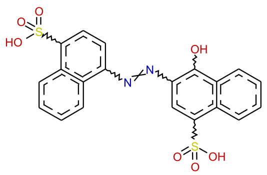	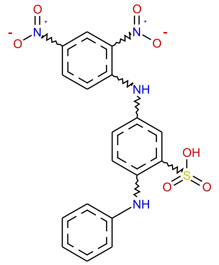
Actual Endpoint (-log C)	2.28997	2.8654	3.20573
Predicted Endpoint (-log C)	3.52921	3.29295	3.55956
Distance	0.790	0.803	0.970
Reference	NTP 411 146	NTP REPORT # 220	NTP REPORT # 335

Model Applicability

Unknown features are fingerprint features in the query molecule, but not found in the training set.

1. OPS PC35 out of range. Value: -3.9689. Training min, max, SD, explained variance: -3.6414, 3.2579, 1.162, 0.0059.
2. Unknown ECFP_6 feature: 672362763: [*]:n(:[*]):[*]
3. Unknown ECFP_6 feature: -830332112: [*]S[*]
4. Unknown ECFP_6 feature: -955816473: [*]SCC(=[*])[*]
5. Unknown ECFP_6 feature: 1731843802: [*]CC(=O)N[*]
6. Unknown ECFP_6 feature: -177077903: [*]N[c](:[cH]:[*]):[cH]:[*]
7. Unknown ECFP_6 feature: -175146122: [*]C(=[*])[c](:[cH]:[*]):[cH]:[*]
8. Unknown ECFP_6 feature: 1430169877: [*]NC(=O)[c](:[*]):[*]
9. Unknown ECFP_6 feature: 1335108269: [*]N[c](:[cH]:[*]):[c]([*]):[*]
10. Unknown ECFP_6 feature: 1997021792: [*]:[cH]:[cH]:[cH]:[*]
11. Unknown ECFP_6 feature: 1334415134: [*]:[cH]:[c](:n(:[*]):[*]):[c](:[*]):[*]
12. Unknown ECFP_6 feature: -1221742244: [*]:[c](:[*]):n1:[cH]:[*]:[*]:[c]:1:[*]
13. Unknown ECFP_6 feature: 1049075205: [*]:n1:[*]:[*]:n:[c]:1:[c](:[*]):[*]
14. Unknown ECFP_6 feature: 77911192: [*][c]1:[*]:[*]:[c](:[*]):n:1:[c](:[*]):[*]
15. Unknown ECFP_6 feature: 911256832: [*]:[c]1:[*]:[*]:n:n:1
16. Unknown ECFP_6 feature: 1986731747: [*]S[c]1:n:[*]:[*]:n:1:[*]
17. Unknown ECFP_6 feature: -708878603: [*]:n1:[*]:[*]:n:[cH]:1
18. Unknown ECFP_6 feature: -677914300: [*]1:[*]:n:n:[cH]:1
19. Unknown ECFP_6 feature: 1427820655: [*]CS[c](:[*]):[*]
20. Unknown ECFP_6 feature: 2019062761: [*]:[c](:[*])O

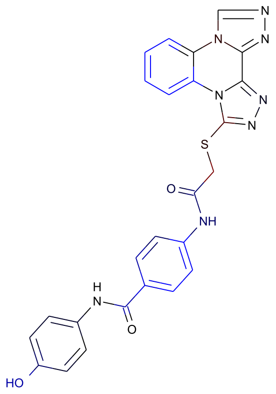

$C_{25}H_{18}N_8O_3S$
Molecular Weight: 510.52722
ALogP: 3.428
Rotatable Bonds: 6
Acceptors: 8
Donors: 3

Model Prediction

Prediction: 0.115
Unit: g/kg_body_weight
Mahalanobis Distance: 41.171
Mahalanobis Distance p-value: 4.35e-044

Mahalanobis Distance: The Mahalanobis distance (MD) is a generalization of the Euclidean distance that accounts for correlations among the X properties. It is calculated as the distance to the center of the training data. The larger the MD, the less trustworthy the prediction.
Mahalanobis Distance p-value: The p-value gives the fraction of training data with an MD greater than or equal to the one for the given sample, assuming normally distributed data. The smaller the p-value, the less trustworthy the prediciton. For highly non-normal X properties (e.g., fingerprints), the MD p-value is wildly inaccurate.

Structural Similar Compounds

Name	C.I.PIGMENT RED 23	C.I. ACID RED 14	C.I. ACID ORANGE 3
Structure	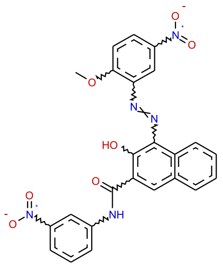	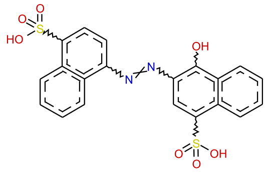	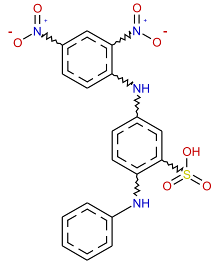
Actual Endpoint (-log C)	2.28997	2.8654	3.20573
Predicted Endpoint (-log C)	3.52921	3.29295	3.55956
Distance	0.790	0.805	0.973
Reference	NTP 411 146	NTP REPORT # 220	NTP REPORT # 335

Model Applicability

Unknown features are fingerprint features in the query molecule, but not found in the training set.

1. OPS PC35 out of range. Value: -4.3198. Training min, max, SD, explained variance: -3.6414, 3.2579, 1.162, 0.0059.
2. Unknown ECFP_6 feature: 672362763: [*]:n(:[*]):[*]
3. Unknown ECFP_6 feature: -830332112: [*]S[*]
4. Unknown ECFP_6 feature: -955816473: [*]SCC(=[*])[*]
5. Unknown ECFP_6 feature: 1731843802: [*]CC(=O)N[*]
6. Unknown ECFP_6 feature: -177077903: [*]N[c](:[cH]:[*]):[cH]:[*]
7. Unknown ECFP_6 feature: -175146122: [*]C(=[*])[c](:[cH]:[*]):[cH]:[*]
8. Unknown ECFP_6 feature: 1430169877: [*]NC(=O)[c](:[*]):[*]
9. Unknown ECFP_6 feature: 1997021792: [*]:[cH]:[cH]:[cH]:[*]
10. Unknown ECFP_6 feature: 1334415134: [*]:[cH]:[c](:n(:[*]):[*]):[c](:[*]):[*]
11. Unknown ECFP_6 feature: -1221742244: [*]:[c](:[*]):n1:[cH]:[*]:[*]:[c]:1:[*]
12. Unknown ECFP_6 feature: 1049075205: [*]:n1:[*]:[*]:n:[c]:1:[c](:[*]):[*]
13. Unknown ECFP_6 feature: 77911192: [*][c]1:[*]:[*]:[c](:[*]):n:1:[c](:[*]):[*]
14. Unknown ECFP_6 feature: 911256832: [*]:[c]1:[*]:[*]:n:n:1
15. Unknown ECFP_6 feature: 1986731747: [*]S[c]1:n:[*]:[*]:n:1:[*]
16. Unknown ECFP_6 feature: -708878603: [*]:n1:[*]:[*]:n:[cH]:1
17. Unknown ECFP_6 feature: -677914300: [*]1:[*]:n:n:[cH]:1
18. Unknown ECFP_6 feature: 1427820655: [*]CS[c](:[*]):[*]
19. Unknown ECFP_6 feature: -177786161: [*]:[cH]:[c](O):[cH]:[*]
20. Unknown ECFP_6 feature: 2019062761: [*]:[c](:[*])O

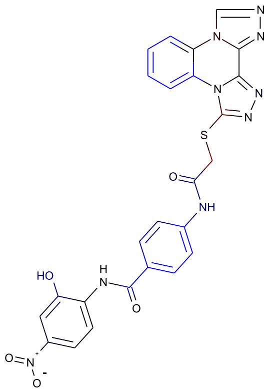

$C_{25}H_{17}N_9O_5S$
Molecular Weight: 555.52477
ALogP: 3.322
Rotatable Bonds: 7
Acceptors: 10
Donors: 3

Model Prediction

Prediction: 0.094
Unit: g/kg_body_weight
Mahalanobis Distance: 42.996
Mahalanobis Distance p-value: 5.02e-047

Mahalanobis Distance: The Mahalanobis distance (MD) is a generalization of the Euclidean distance that accounts for correlations among the X properties. It is calculated as the distance to the center of the training data. The larger the MD, the less trustworthy the prediction.
Mahalanobis Distance p-value: The p-value gives the fraction of training data with an MD greater than or equal to the one for the given sample, assuming normally distributed data. The smaller the p-value, the less trustworthy the prediciton. For highly non-normal X properties (e.g., fingerprints), the MD p-value is wildly inaccurate.

Structural Similar Compounds

Name	C.I.PIGMENT RED 23	C.I. ACID RED 14	C.I. ACID ORANGE 3
Structure	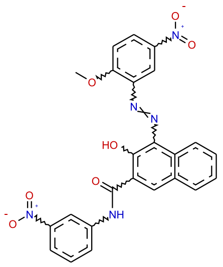	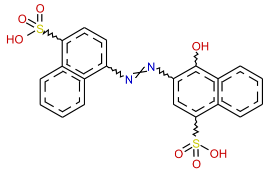	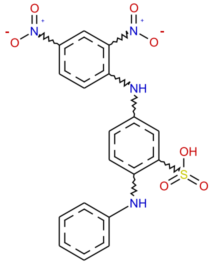
Actual Endpoint (-log C)	2.28997	2.8654	3.20573
Predicted Endpoint (-log C)	3.52921	3.29295	3.55956
Distance	0.811	0.868	0.981
Reference	NTP 411 146	NTP REPORT # 220	NTP REPORT # 335

Model Applicability

Unknown features are fingerprint features in the query molecule, but not found in the training set.

1. OPS PC35 out of range. Value: -3.8928. Training min, max, SD, explained variance: -3.6414, 3.2579, 1.162, 0.0059.
2. Unknown FCFP_2 feature: 5: [*][O-]
3. Unknown FCFP_2 feature: -828984032: [*][N+](=[*])[c](:c:[*]):c:[*]
4. Unknown FCFP_2 feature: -1338588315: [*]:[c](:[*])[N+](=O)[O-]
5. Unknown FCFP_2 feature: 1872392852: [*][N+](=O)[*]
6. Unknown FCFP_2 feature: 260476081: [*][N+](=[*])[O-]
7. Unknown ECFP_6 feature: 672362763: [*]:n(:[*]):[*]
8. Unknown ECFP_6 feature: -830332112: [*]S[*]
9. Unknown ECFP_6 feature: 1043790491: [*][N+](=[*])[*]
10. Unknown ECFP_6 feature: 781519895: [*][O-]
11. Unknown ECFP_6 feature: -955816473: [*]SCC(=[*])[*]
12. Unknown ECFP_6 feature: 1731843802: [*]CC(=O)N[*]
13. Unknown ECFP_6 feature: -177077903: [*]N[c](:[cH]:[*]):[cH]:[*]
14. Unknown ECFP_6 feature: -175146122: [*]C(=[*])[c](:[cH]:[*]):[cH]:[*]
15. Unknown ECFP_6 feature: 1430169877: [*]NC(=O)[c](:[*]):[*]
16. Unknown ECFP_6 feature: 1335108269: [*]N[c](:[cH]:[*]):[c]([*]):[*]
17. Unknown ECFP_6 feature: 1997021792: [*]:[cH]:[cH]:[cH]:[*]
18. Unknown ECFP_6 feature: 1334415134: [*]:[cH]:[c](:n(:[*]):[*]):[c](:[*]):[*]
19. Unknown ECFP_6 feature: -1221742244: [*]:[c](:[*]):n1:[cH]:[*]:[*]:[c]:1:[*]
20. Unknown ECFP_6 feature: 1049075205: [*]:n1:[*]:[*]:n:[c]:1:[c](:[*]):[*]

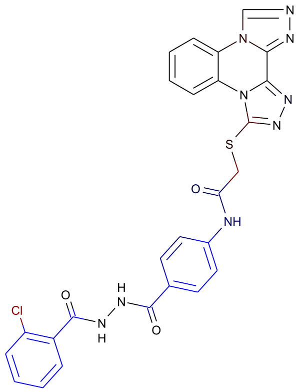

$C_{26}H_{18}ClN_9O_3S$
Molecular Weight: 571.99761
ALogP: 3.591
Rotatable Bonds: 7
Acceptors: 8
Donors: 3

Model Prediction

Prediction: 0.155
Unit: g/kg_body_weight
Mahalanobis Distance: 39.534
Mahalanobis Distance p-value: 2.16e-041

Mahalanobis Distance: The Mahalanobis distance (MD) is a generalization of the Euclidean distance that accounts for correlations among the X properties. It is calculated as the distance to the center of the training data. The larger the MD, the less trustworthy the prediction.
Mahalanobis Distance p-value: The p-value gives the fraction of training data with an MD greater than or equal to the one for the given sample, assuming normally distributed data. The smaller the p-value, the less trustworthy the prediciton. For highly non-normal X properties (e.g., fingerprints), the MD p-value is wildly inaccurate.

Structural Similar Compounds

Name	C.I.PIGMENT RED 23	C.I. ACID RED 14	DIARYLANILIDE YELLOW
Structure	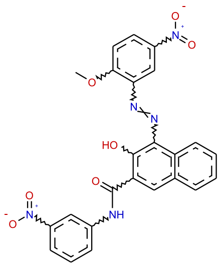	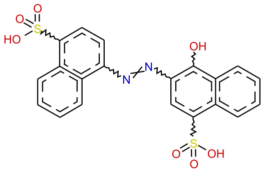	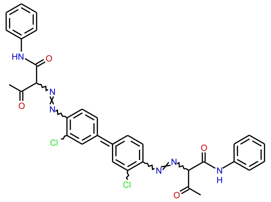
Actual Endpoint (-log C)	2.28997	2.8654	2.70208
Predicted Endpoint (-log C)	3.52921	3.29295	3.76154
Distance	0.823	0.873	0.919
Reference	NTP 411 146	NTP REPORT # 220	NTP 30 C-4

Model Applicability

Unknown features are fingerprint features in the query molecule, but not found in the training set.

1. All properties and OPS components are within expected ranges.
2. Unknown ECFP_6 feature: 672362763: [*]:n(:[*]):[*]
3. Unknown ECFP_6 feature: -830332112: [*]S[*]
4. Unknown ECFP_6 feature: -955816473: [*]SCC(=[*])[*]
5. Unknown ECFP_6 feature: 1731843802: [*]CC(=O)N[*]
6. Unknown ECFP_6 feature: -177077903: [*]N[c](:[cH]:[*]):[cH]:[*]
7. Unknown ECFP_6 feature: -175146122: [*]C(=[*])[c](:[cH]:[*]):[cH]:[*]
8. Unknown ECFP_6 feature: 1430169877: [*]NC(=O)[c](:[*]):[*]
9. Unknown ECFP_6 feature: 1635339976: [*]NNC(=[*])[*]
10. Unknown ECFP_6 feature: 1997021792: [*]:[cH]:[cH]:[cH]:[*]
11. Unknown ECFP_6 feature: 1334415134: [*]:[cH]:[c](:n(:[*]):[*]):[c](:[*]):[*]
12. Unknown ECFP_6 feature: -1221742244: [*]:[c](:[*]):n1:[cH]:[*]:[*]:[c]:1:[*]
13. Unknown ECFP_6 feature: 1049075205: [*]:n1:[*]:[*]:n:[c]:1:[c](:[*]):[*]
14. Unknown ECFP_6 feature: 77911192: [*][c]1:[*]:[*]:[c](:[*]):n:1:[c](:[*]):[*]
15. Unknown ECFP_6 feature: 911256832: [*]:[c]1:[*]:[*]:n:n:1
16. Unknown ECFP_6 feature: 1986731747: [*]S[c]1:n:[*]:[*]:n:1:[*]
17. Unknown ECFP_6 feature: -708878603: [*]:n1:[*]:[*]:n:[cH]:1
18. Unknown ECFP_6 feature: -677914300: [*]1:[*]:n:n:[cH]:1
19. Unknown ECFP_6 feature: 1427820655: [*]CS[c](:[*]):[*]
20. Unknown ECFP_6 feature: 99947387: [*]:[c](:[*])Cl

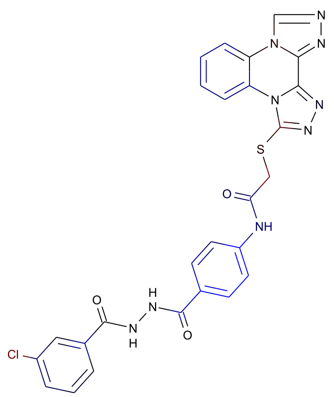

$C_{26}H_{18}ClN_9O_3S$
Molecular Weight: 571.99761
ALogP: 3.591
Rotatable Bonds: 7
Acceptors: 8
Donors: 3

Model Prediction

Prediction: 0.089
Unit: g/kg_body_weight
Mahalanobis Distance: 40.488
Mahalanobis Distance p-value: 5.71e-043

Mahalanobis Distance: The Mahalanobis distance (MD) is a generalization of the Euclidean distance that accounts for correlations among the X properties. It is calculated as the distance to the center of the training data. The larger the MD, the less trustworthy the prediction.
Mahalanobis Distance p-value: The p-value gives the fraction of training data with an MD greater than or equal to the one for the given sample, assuming normally distributed data. The smaller the p-value, the less trustworthy the prediciton. For highly non-normal X properties (e.g., fingerprints), the MD p-value is wildly inaccurate.

Structural Similar Compounds

Name	C.I.PIGMENT RED 23	C.I. ACID RED 14	DIARYLANILIDE YELLOW
Structure	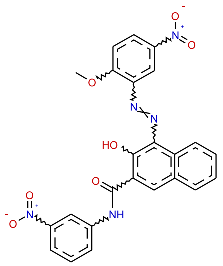	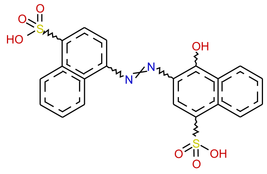	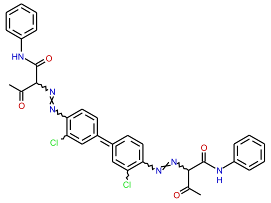
Actual Endpoint (-log C)	2.28997	2.8654	2.70208
Predicted Endpoint (-log C)	3.52921	3.29295	3.76154
Distance	0.824	0.873	0.921
Reference	NTP 411 146	NTP REPORT # 220	NTP 30 C-4

Model Applicability

Unknown features are fingerprint features in the query molecule, but not found in the training set.

1. All properties and OPS components are within expected ranges.
2. Unknown ECFP_6 feature: 672362763: [*]:n(:[*]):[*]
3. Unknown ECFP_6 feature: -830332112: [*]S[*]
4. Unknown ECFP_6 feature: -955816473: [*]SCC(=[*])[*]
5. Unknown ECFP_6 feature: 1731843802: [*]CC(=O)N[*]
6. Unknown ECFP_6 feature: -177077903: [*]N[c](:[cH]:[*]):[cH]:[*]
7. Unknown ECFP_6 feature: -175146122: [*]C(=[*])[c](:[cH]:[*]):[cH]:[*]
8. Unknown ECFP_6 feature: 1430169877: [*]NC(=O)[c](:[*]):[*]
9. Unknown ECFP_6 feature: 1635339976: [*]NNC(=[*])[*]
10. Unknown ECFP_6 feature: -176494269: [*]:[cH]:[c](Cl):[cH]:[*]
11. Unknown ECFP_6 feature: 1997021792: [*]:[cH]:[cH]:[cH]:[*]
12. Unknown ECFP_6 feature: 1334415134: [*]:[cH]:[c](:n(:[*]):[*]):[c](:[*]):[*]
13. Unknown ECFP_6 feature: -1221742244: [*]:[c](:[*]):n1:[cH]:[*]:[*]:[c]:1:[*]
14. Unknown ECFP_6 feature: 1049075205: [*]:n1:[*]:[*]:n:[c]:1:[c](:[*]):[*]
15. Unknown ECFP_6 feature: 77911192: [*][c]1:[*]:[*]:[c](:[*]):n:1:[c](:[*]):[*]
16. Unknown ECFP_6 feature: 911256832: [*]:[c]1:[*]:[*]:n:n:1
17. Unknown ECFP_6 feature: 1986731747: [*]S[c]1:n:[*]:[*]:n:1:[*]
18. Unknown ECFP_6 feature: -708878603: [*]:n1:[*]:[*]:n:[cH]:1
19. Unknown ECFP_6 feature: -677914300: [*]1:[*]:n:n:[cH]:1
20. Unknown ECFP_6 feature: 1427820655: [*]CS[c](:[*]):[*]
21. Unknown ECFP_6 feature: 99947387: [*]:[c](:[*])Cl

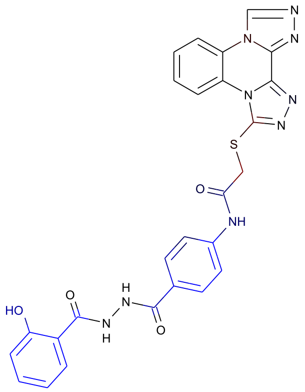

$C_{26}H_{19}N_9O_4S$
Molecular Weight: 553.55195
ALogP: 2.685
Rotatable Bonds: 7
Acceptors: 9
Donors: 4

Model Prediction

Prediction: 0.211
Unit: g/kg_body_weight
Mahalanobis Distance: 39.052
Mahalanobis Distance p-value: 1.38e-040

Mahalanobis Distance: The Mahalanobis distance (MD) is a generalization of the Euclidean distance that accounts for correlations among the X properties. It is calculated as the distance to the center of the training data. The larger the MD, the less trustworthy the prediction.
Mahalanobis Distance p-value: The p-value gives the fraction of training data with an MD greater than or equal to the one for the given sample, assuming normally distributed data. The smaller the p-value, the less trustworthy the prediciton. For highly non-normal X properties (e.g., fingerprints), the MD p-value is wildly inaccurate.

Structural Similar Compounds

Name	C.I.PIGMENT RED 23	C.I. ACID RED 14	C.I. ACID ORANGE 3
Structure	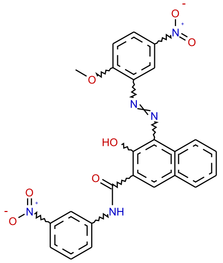	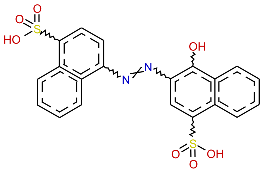	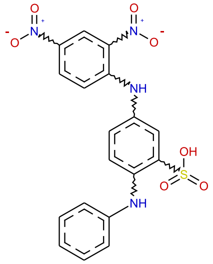
Actual Endpoint (-log C)	2.28997	2.8654	3.20573
Predicted Endpoint (-log C)	3.52921	3.29295	3.55956
Distance	0.866	0.872	1.000
Reference	NTP 411 146	NTP REPORT # 220	NTP REPORT # 335

Model Applicability

Unknown features are fingerprint features in the query molecule, but not found in the training set.

1. OPS PC35 out of range. Value: -3.842. Training min, max, SD, explained variance: -3.6414, 3.2579, 1.162, 0.0059.
2. Unknown ECFP_6 feature: 672362763: [*]:n(:[*]):[*]
3. Unknown ECFP_6 feature: -830332112: [*]S[*]
4. Unknown ECFP_6 feature: -955816473: [*]SCC(=[*])[*]
5. Unknown ECFP_6 feature: 1731843802: [*]CC(=O)N[*]
6. Unknown ECFP_6 feature: -177077903: [*]N[c](:[cH]:[*]):[cH]:[*]
7. Unknown ECFP_6 feature: -175146122: [*]C(=[*])[c](:[cH]:[*]):[cH]:[*]
8. Unknown ECFP_6 feature: 1430169877: [*]NC(=O)[c](:[*]):[*]
9. Unknown ECFP_6 feature: 1635339976: [*]NNC(=[*])[*]
10. Unknown ECFP_6 feature: 1997021792: [*]:[cH]:[cH]:[cH]:[*]
11. Unknown ECFP_6 feature: 1334415134: [*]:[cH]:[c](:n(:[*]):[*]):[c](:[*]):[*]
12. Unknown ECFP_6 feature: -1221742244: [*]:[c](:[*]):n1:[cH]:[*]:[*]:[c]:1:[*]
13. Unknown ECFP_6 feature: 1049075205: [*]:n1:[*]:[*]:n:[c]:1:[c](:[*]):[*]
14. Unknown ECFP_6 feature: 77911192: [*][c]1:[*]:[*]:[c](:[*]):n:1:[c](:[*]):[*]
15. Unknown ECFP_6 feature: 911256832: [*]:[c]1:[*]:[*]:n:n:1
16. Unknown ECFP_6 feature: 1986731747: [*]S[c]1:n:[*]:[*]:n:1:[*]
17. Unknown ECFP_6 feature: -708878603: [*]:n1:[*]:[*]:n:[cH]:1
18. Unknown ECFP_6 feature: -677914300: [*]1:[*]:n:n:[cH]:1
19. Unknown ECFP_6 feature: 1427820655: [*]CS[c](:[*]):[*]
20. Unknown ECFP_6 feature: 2019062761: [*]:[c](:[*])O

Sorafenib

TOPKAT_Chronic_LOAEL

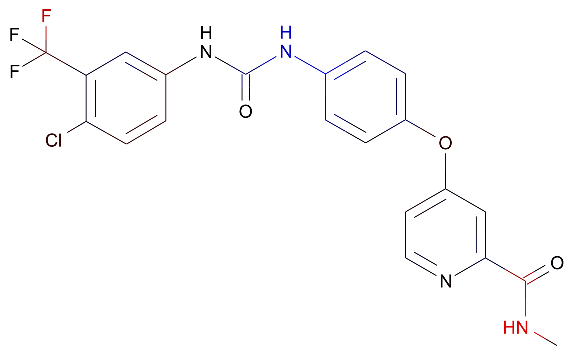

$C_{21}H_{16}ClF_3N_4O_3$
Molecular Weight: 464.82494
ALogP: 4.175
Rotatable Bonds: 6
Acceptors: 4
Donors: 3

Model Prediction

Prediction: 0.005
Unit: g/kg_body_weight
Mahalanobis Distance: 29.988
Mahalanobis Distance p-value: 1.21e-024

Mahalanobis Distance: The Mahalanobis distance (MD) is a generalization of the Euclidean distance that accounts for correlations among the X properties. It is calculated as the distance to the center of the training data. The larger the MD, the less trustworthy the prediction.
Mahalanobis Distance p-value: The p-value gives the fraction of training data with an MD greater than or equal to the one for the given sample, assuming normally distributed data. The smaller the p-value, the less trustworthy the prediciton. For highly non-normal X properties (e.g., fingerprints), the MD p-value is wildly inaccurate.

Structural Similar Compounds

Name	GLYBURIDE	D & C RED 9	SODIUM ACIFLUORFEN
Structure	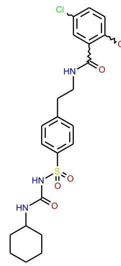	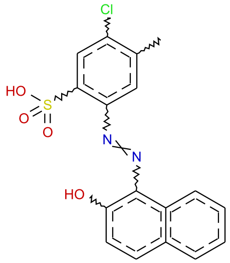	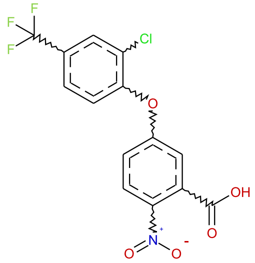
Actual Endpoint (-log C)	4.21661	3.87715	4.16036
Predicted Endpoint (-log C)	4.21035	3.6546	4.65915
Distance	0.636	0.722	0.736
Reference	UPJ-26452	NTP REPORT # 225	EPA COVER SHEET 0192;891101;(1)

Model Applicability

Unknown features are fingerprint features in the query molecule, but not found in the training set.

1. All properties and OPS components are within expected ranges.
2. Unknown ECFP_6 feature: -1046436026: [*]F
3. Unknown ECFP_6 feature: 1413420509: [*]C(=[*])[c](:[cH]:[*]):n:[*]
4. Unknown ECFP_6 feature: 1996163143: [*]:[cH]:[cH]:n:[*]
5. Unknown ECFP_6 feature: -677309799: [*][c](:[*]):n:[cH]:[*]
6. Unknown ECFP_6 feature: 1430169877: [*]NC(=O)[c](:[*]):[*]
7. Unknown ECFP_6 feature: 1338334141: [*]C(=[*])NC
8. Unknown ECFP_6 feature: 864287155: [*]NC
9. Unknown ECFP_6 feature: 1305253718: [*]:[c](:[*])O[c](:[*]):[*]
10. Unknown ECFP_6 feature: -177077903: [*]N[c](:[cH]:[*]):[cH]:[*]
11. Unknown ECFP_6 feature: -649580166: [*]NC(=O)N[*]
12. Unknown ECFP_6 feature: 1336678434: [*][c](:[*]):[c](:[cH]:[*])C([*])([*])[*]
13. Unknown ECFP_6 feature: -1952889961: [*]:[c](:[*])C(F)(F)F
14. Unknown ECFP_6 feature: 226796801: [*]C([*])([*])F
15. Unknown ECFP_6 feature: 99947387: [*]:[c](:[*])Cl

Feature Contribution

Top features for positive contribution			
Fingerprint	Bit/Smiles	Feature Structure	Score

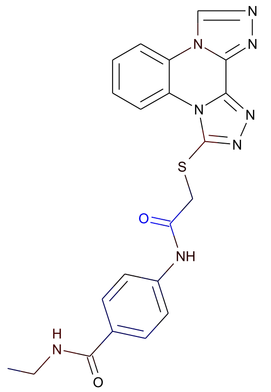

$C_{21}H_{18}N_8O_2S$

Molecular Weight: 446.48502

ALogP: 2.442

Rotatable Bonds: 6

Acceptors: 7

Donors: 2

Model Prediction

Prediction: 0.099

Unit: g/kg_body_weight

Mahalanobis Distance: 13.449

Mahalanobis Distance p-value: 1.74e-011

Mahalanobis Distance: The Mahalanobis distance (MD) is a generalization of the Euclidean distance that accounts for correlations among the X properties. It is calculated as the distance to the center of the training data. The larger the MD, the less trustworthy the prediction.
Mahalanobis Distance p-value: The p-value gives the fraction of training data with an MD greater than or equal to the one for the given sample, assuming normally distributed data. The smaller the p-value, the less trustworthy the prediciton. For highly non-normal X properties (e.g., fingerprints), the MD p-value is wildly inaccurate.

Structural Similar Compounds

Name	C.I.PIGMENT RED 23	SALICYLAZOSULFAPYRIDINE	TRIAMTERENE
Structure	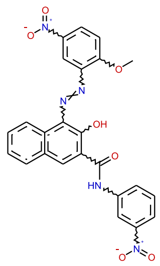	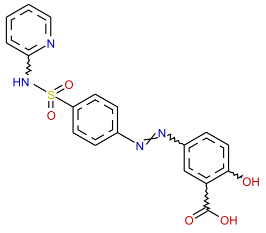	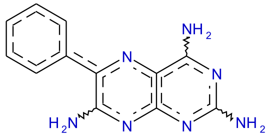
Actual Endpoint (-log C)	2.30052	3.375	4.00564
Predicted Endpoint (-log C)	3.55333	2.80292	3.1992
Distance	0.775	0.845	1.035
Reference	NCI/NTP TR-411	NCI/NTP TR-457	NCI/NTP TR-420

Model Applicability

Unknown features are fingerprint features in the query molecule, but not found in the training set.

1. Num_AromaticRings out of range. Value: 5. Training min, max, mean, SD: 0, 4, 1.1685, 0.8469.
2. OPS PC9 out of range. Value: 4.1105. Training min, max, SD, explained variance: -2.8548, 3.3954, 1.263, 0.0360.
3. Unknown FCFP_2 feature: -203115083: [*]:[c](:[*]):n1:[c](:[*]):[*]:[*]:c:1
4. Unknown FCFP_2 feature: -1410079687: [*]S[c]1:n:[*]:[*]:n:1:[*]
5. Unknown FCFP_2 feature: -124685461: [*]:n1:[*]:[*]:n:c:1

Feature Contribution

Top features for positive contribution

Fingerprint	Bit/Smiles	Feature Structure	Score
FCFP_2	-885550502	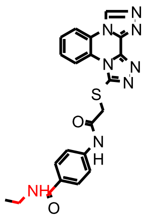 [*]CNC(=[*])[*]	0.115

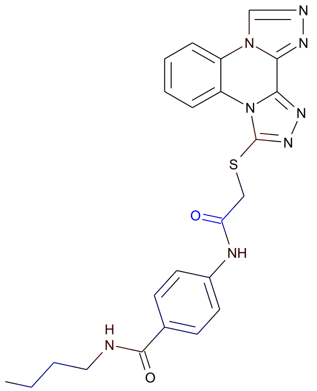

$C_{23}H_{22}N_8O_2S$

Molecular Weight: 474.53818

ALogP: 3.422

Rotatable Bonds: 8

Acceptors: 7

Donors: 2

Model Prediction

Prediction: 0.145

Unit: g/kg_body_weight

Mahalanobis Distance: 13.671

Mahalanobis Distance p-value: 6.16e-012

Mahalanobis Distance: The Mahalanobis distance (MD) is a generalization of the Euclidean distance that accounts for correlations among the X properties. It is calculated as the distance to the center of the training data. The larger the MD, the less trustworthy the prediction.
Mahalanobis Distance p-value: The p-value gives the fraction of training data with an MD greater than or equal to the one for the given sample, assuming normally distributed data. The smaller the p-value, the less trustworthy the prediciton. For highly non-normal X properties (e.g., fingerprints), the MD p-value is wildly inaccurate.

Structural Similar Compounds

Name	C.I.PIGMENT RED 23	SALICYLAZOSULFAPYRIDINE	RESERPINE
Structure	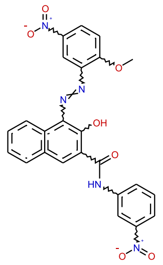	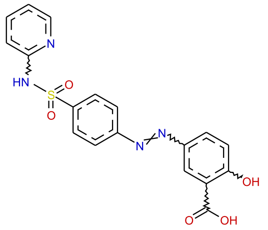	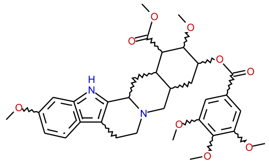
Actual Endpoint (-log C)	2.30052	3.375	6.13118
Predicted Endpoint (-log C)	3.55333	2.80292	4.38304
Distance	0.730	0.863	1.007
Reference	NCI/NTP TR-411	NCI/NTP TR-457	NCI/NTP TR-193

Model Applicability

Unknown features are fingerprint features in the query molecule, but not found in the training set.

1. Num_AromaticRings out of range. Value: 5. Training min, max, mean, SD: 0, 4, 1.1685, 0.8469.
2. OPS PC9 out of range. Value: 4.6803. Training min, max, SD, explained variance: -2.8548, 3.3954, 1.263, 0.0360.
3. Unknown FCFP_2 feature: -203115083: [*]:[c](:[*]):n1:[c](:[*]):[*]:[*]:c:1
4. Unknown FCFP_2 feature: -1410079687: [*]S[c]1:n:[*]:[*]:n:1:[*]
5. Unknown FCFP_2 feature: -124685461: [*]:n1:[*]:[*]:n:c:1

Feature Contribution

Top features for positive contribution

Fingerprint	Bit/Smiles	Feature Structure	Score
FCFP_2	-885550502	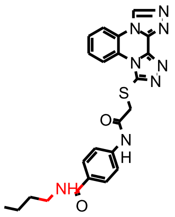 [*]CNC(=[*])[*]	0.115

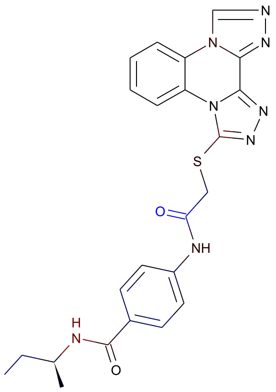

$C_{23}H_{22}N_8O_2S$

Molecular Weight: 474.53818

ALogP: 3.343

Rotatable Bonds: 7

Acceptors: 7

Donors: 2

Model Prediction

Prediction: 0.114

Unit: g/kg_body_weight

Mahalanobis Distance: 12.738

Mahalanobis Distance p-value: 4.58e-010

Mahalanobis Distance: The Mahalanobis distance (MD) is a generalization of the Euclidean distance that accounts for correlations among the X properties. It is calculated as the distance to the center of the training data. The larger the MD, the less trustworthy the prediction.
Mahalanobis Distance p-value: The p-value gives the fraction of training data with an MD greater than or equal to the one for the given sample, assuming normally distributed data. The smaller the p-value, the less trustworthy the prediciton. For highly non-normal X properties (e.g., fingerprints), the MD p-value is wildly inaccurate.

Structural Similar Compounds

Name	C.I.PIGMENT RED 23	SALICYLAZOSULFAPYRIDINE	RESERPINE
Structure	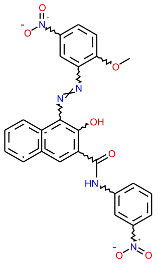	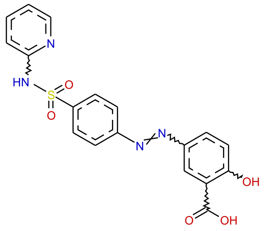	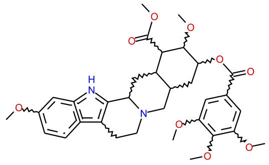
Actual Endpoint (-log C)	2.30052	3.375	6.13118
Predicted Endpoint (-log C)	3.55333	2.80292	4.38304
Distance	0.730	0.854	1.020
Reference	NCI/NTP TR-411	NCI/NTP TR-457	NCI/NTP TR-193

Model Applicability

Unknown features are fingerprint features in the query molecule, but not found in the training set.

1. Num_AromaticRings out of range. Value: 5. Training min, max, mean, SD: 0, 4, 1.1685, 0.8469.
2. OPS PC9 out of range. Value: 4.4755. Training min, max, SD, explained variance: -2.8548, 3.3954, 1.263, 0.0360.
3. Unknown FCFP_2 feature: -203115083: [*]:[c](:[*]):n1:[c](:[*]):[*]:[*]:c:1
4. Unknown FCFP_2 feature: -1410079687: [*]S[c]1:n:[*]:[*]:n:1:[*]
5. Unknown FCFP_2 feature: -124685461: [*]:n1:[*]:[*]:n:c:1

Feature Contribution

Top features for positive contribution

Fingerprint	Bit/Smiles	Feature Structure	Score
FCFP_2	-885550502	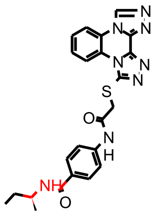 [*]CNC(=[*])[*]	0.115

ter. butyl.cdx

TOPKAT_Rat_Maximum_Tolerated_Dose_Feed

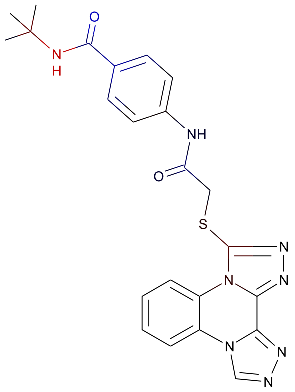

$C_{23}H_{22}N_8O_2S$
Molecular Weight: 474.53818
ALogP: 3.025
Rotatable Bonds: 6
Acceptors: 7
Donors: 2

Model Prediction

Prediction: 0.075
Unit: g/kg_body_weight
Mahalanobis Distance: 12.190
Mahalanobis Distance p-value: 5.38e-009

Mahalanobis Distance: The Mahalanobis distance (MD) is a generalization of the Euclidean distance that accounts for correlations among the X properties. It is calculated as the distance to the center of the training data. The larger the MD, the less trustworthy the prediction.
Mahalanobis Distance p-value: The p-value gives the fraction of training data with an MD greater than or equal to the one for the given sample, assuming normally distributed data. The smaller the p-value, the less trustworthy the prediciton. For highly non-normal X properties (e.g., fingerprints), the MD p-value is wildly inaccurate.

Structural Similar Compounds

Name	C.I.PIGMENT RED 23	SALICYLAZOSULFAPYRIDINE	RESERPINE
Structure	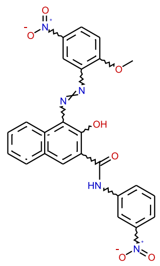	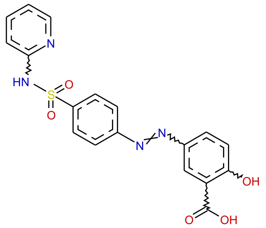	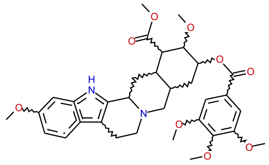
Actual Endpoint (-log C)	2.30052	3.375	6.13118
Predicted Endpoint (-log C)	3.55333	2.80292	4.38304
Distance	0.742	0.848	1.054
Reference	NCI/NTP TR-411	NCI/NTP TR-457	NCI/NTP TR-193

Model Applicability

Unknown features are fingerprint features in the query molecule, but not found in the training set.

1. Num_AromaticRings out of range. Value: 5. Training min, max, mean, SD: 0, 4, 1.1685, 0.8469.
2. OPS PC9 out of range. Value: 3.9849. Training min, max, SD, explained variance: -2.8548, 3.3954, 1.263, 0.0360.
3. Unknown FCFP_2 feature: -203115083: [*][c]1:[*]:[*]:[c](:[*]):n:1:[c](:[*]):[*]
4. Unknown FCFP_2 feature: -1410079687: [*]S[c]1:n:[*]:[*]:n:1:[*]
5. Unknown FCFP_2 feature: -124685461: [*]:n1:[*]:[*]:n:c:1

Feature Contribution

Top features for positive contribution

Fingerprint	Bit/Smiles	Feature Structure	Score
FCFP_2	-885550502	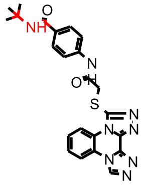 [*]C(=[*])NC([*])([*])[*]	0.115

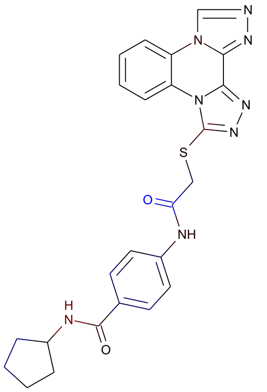

$C_{24}H_{22}N_8O_2S$

Molecular Weight: 486.54888

ALogP: 3.496

Rotatable Bonds: 6

Acceptors: 7

Donors: 2

Model Prediction

Prediction: 0.088

Unit: g/kg_body_weight

Mahalanobis Distance: 12.535

Mahalanobis Distance p-value: 1.15e-009

Mahalanobis Distance: The Mahalanobis distance (MD) is a generalization of the Euclidean distance that accounts for correlations among the X properties. It is calculated as the distance to the center of the training data. The larger the MD, the less trustworthy the prediction.
Mahalanobis Distance p-value: The p-value gives the fraction of training data with an MD greater than or equal to the one for the given sample, assuming normally distributed data. The smaller the p-value, the less trustworthy the prediciton. For highly non-normal X properties (e.g., fingerprints), the MD p-value is wildly inaccurate.

Structural Similar Compounds

Name	C.I.PIGMENT RED 23	SALICYLAZOSULFAPYRIDINE	RESERPINE
Structure	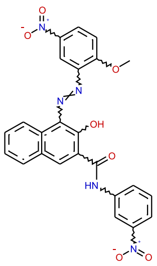	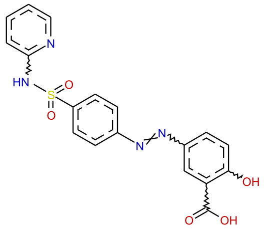	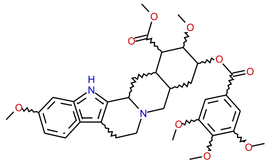
Actual Endpoint (-log C)	2.30052	3.375	6.13118
Predicted Endpoint (-log C)	3.55333	2.80292	4.38304
Distance	0.721	0.852	1.026
Reference	NCI/NTP TR-411	NCI/NTP TR-457	NCI/NTP TR-193

Model Applicability

Unknown features are fingerprint features in the query molecule, but not found in the training set.

1. Num_AromaticRings out of range. Value: 5. Training min, max, mean, SD: 0, 4, 1.1685, 0.8469.
2. OPS PC9 out of range. Value: 4.3767. Training min, max, SD, explained variance: -2.8548, 3.3954, 1.263, 0.0360.
3. Unknown FCFP_2 feature: -203115083: [*]:[c](:[*]):n1:[c](:[*]):[*]:[*]:c:1
4. Unknown FCFP_2 feature: -1410079687: [*]S[c]1:n:[*]:[*]:n:1:[*]
5. Unknown FCFP_2 feature: -124685461: [*]:n1:[*]:[*]:n:c:1

Feature Contribution

Top features for positive contribution

Fingerprint	Bit/Smiles	Feature Structure	Score
FCFP_2	-885550502	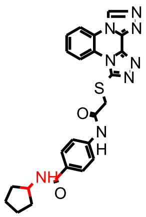 [*]CNC(=[*])[*]	0.115

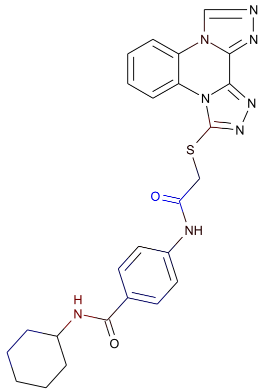

$C_{25}H_{24}N_8O_2S$
Molecular Weight: 500.57546
ALogP: 3.952
Rotatable Bonds: 6
Acceptors: 7
Donors: 2

Model Prediction

Prediction: 0.083
Unit: g/kg_body_weight
Mahalanobis Distance: 12.487
Mahalanobis Distance p-value: 1.43e-009

Mahalanobis Distance: The Mahalanobis distance (MD) is a generalization of the Euclidean distance that accounts for correlations among the X properties. It is calculated as the distance to the center of the training data. The larger the MD, the less trustworthy the prediction.
Mahalanobis Distance p-value: The p-value gives the fraction of training data with an MD greater than or equal to the one for the given sample, assuming normally distributed data. The smaller the p-value, the less trustworthy the prediciton. For highly non-normal X properties (e.g., fingerprints), the MD p-value is wildly inaccurate.

Structural Similar Compounds

Name	C.I.PIGMENT RED 23	SALICYLAZOSULFAPYRIDINE	RESERPINE
Structure	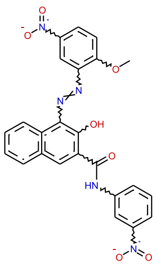	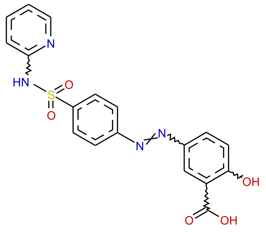	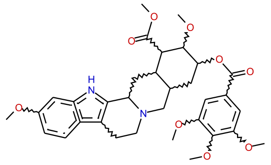
Actual Endpoint (-log C)	2.30052	3.375	6.13118
Predicted Endpoint (-log C)	3.55333	2.80292	4.38304
Distance	0.706	0.862	1.016
Reference	NCI/NTP TR-411	NCI/NTP TR-457	NCI/NTP TR-193

Model Applicability

Unknown features are fingerprint features in the query molecule, but not found in the training set.

1. Num_AromaticRings out of range. Value: 5. Training min, max, mean, SD: 0, 4, 1.1685, 0.8469.
2. OPS PC9 out of range. Value: 4.4089. Training min, max, SD, explained variance: -2.8548, 3.3954, 1.263, 0.0360.
3. Unknown FCFP_2 feature: -203115083: [*]:[c](:[*]):n1:[c](:[*]):[*]:[*]:c:1
4. Unknown FCFP_2 feature: -1410079687: [*]S[c]1:n:[*]:[*]:n:1:[*]
5. Unknown FCFP_2 feature: -124685461: [*]:n1:[*]:[*]:n:c:1

Feature Contribution

Top features for positive contribution

Fingerprint	Bit/Smiles	Feature Structure	Score
FCFP_2	-885550502	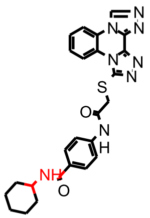 [*]CNC(=[*])[*]	0.115

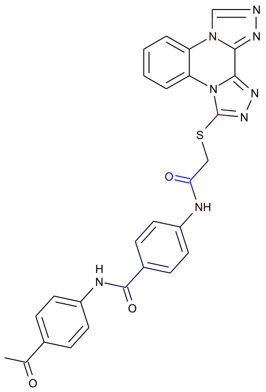

$C_{27}H_{20}N_8O_3S$

Molecular Weight: 536.56449

ALogP: 3.41

Rotatable Bonds: 7

Acceptors: 8

Donors: 2

Model Prediction

Prediction: 0.087

Unit: g/kg_body_weight

Mahalanobis Distance: 13.550

Mahalanobis Distance p-value: 1.08e-011

Mahalanobis Distance: The Mahalanobis distance (MD) is a generalization of the Euclidean distance that accounts for correlations among the X properties. It is calculated as the distance to the center of the training data. The larger the MD, the less trustworthy the prediction.
Mahalanobis Distance p-value: The p-value gives the fraction of training data with an MD greater than or equal to the one for the given sample, assuming normally distributed data. The smaller the p-value, the less trustworthy the prediciton. For highly non-normal X properties (e.g., fingerprints), the MD p-value is wildly inaccurate.

Structural Similar Compounds

Name	C.I.PIGMENT RED 23	SALICYLAZOSULFAPYRIDINE	RESERPINE
Structure	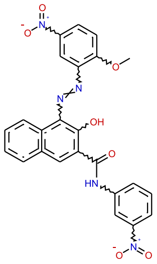	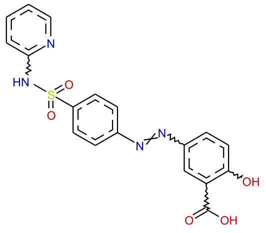	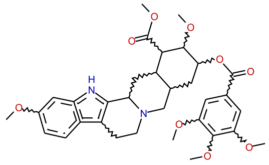
Actual Endpoint (-log C)	2.30052	3.375	6.13118
Predicted Endpoint (-log C)	3.55333	2.80292	4.38304
Distance	0.806	1.053	1.177
Reference	NCI/NTP TR-411	NCI/NTP TR-457	NCI/NTP TR-193

Model Applicability

Unknown features are fingerprint features in the query molecule, but not found in the training set.

1. Num_AromaticRings out of range. Value: 6. Training min, max, mean, SD: 0, 4, 1.1685, 0.8469.
2. OPS PC9 out of range. Value: 3.8948. Training min, max, SD, explained variance: -2.8548, 3.3954, 1.263, 0.0360.
3. Unknown FCFP_2 feature: -203115083: [*]:[c](:[*]):n1:[c](:[*]):[*]:[*]:c:1
4. Unknown FCFP_2 feature: -1410079687: [*]S[c]1:n:[*]:[*]:n:1:[*]
5. Unknown FCFP_2 feature: -124685461: [*]:n1:[*]:[*]:n:c:1

Feature Contribution

Top features for positive contribution

Fingerprint	Bit/Smiles	Feature Structure	Score
FCFP_2	1036089772	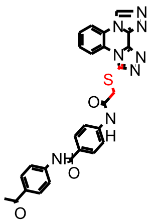 [*]CS[c](:[*]):[*]	0.075

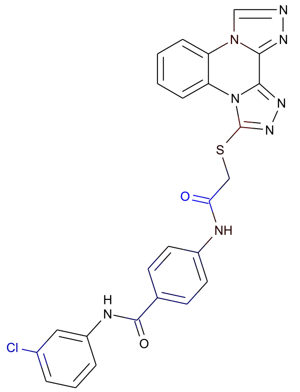

$C_{25}H_{17}ClN_8O_2S$

Molecular Weight: 528.97287

ALogP: 4.334

Rotatable Bonds: 6

Acceptors: 7

Donors: 2

Model Prediction

Prediction: 0.133

Unit: g/kg_body_weight

Mahalanobis Distance: 13.535

Mahalanobis Distance p-value: 1.16e-011

Mahalanobis Distance: The Mahalanobis distance (MD) is a generalization of the Euclidean distance that accounts for correlations among the X properties. It is calculated as the distance to the center of the training data. The larger the MD, the less trustworthy the prediction.
Mahalanobis Distance p-value: The p-value gives the fraction of training data with an MD greater than or equal to the one for the given sample, assuming normally distributed data. The smaller the p-value, the less trustworthy the prediciton. For highly non-normal X properties (e.g., fingerprints), the MD p-value is wildly inaccurate.

Structural Similar Compounds

Name	C.I.PIGMENT RED 23	SALICYLAZOSULFAPYRIDINE	RESERPINE
Structure	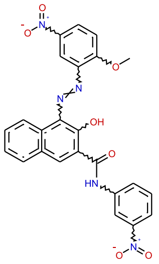	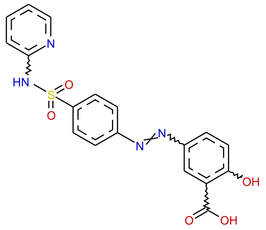	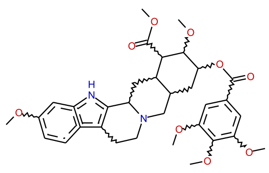
Actual Endpoint (-log C)	2.30052	3.375	6.13118
Predicted Endpoint (-log C)	3.55333	2.80292	4.38304
Distance	0.847	1.065	1.178
Reference	NCI/NTP TR-411	NCI/NTP TR-457	NCI/NTP TR-193

Model Applicability

Unknown features are fingerprint features in the query molecule, but not found in the training set.

1. Num_AromaticRings out of range. Value: 6. Training min, max, mean, SD: 0, 4, 1.1685, 0.8469.
2. OPS PC5 out of range. Value: 5.3168. Training min, max, SD, explained variance: -3.3892, 5.0834, 1.644, 0.0611.
3. OPS PC9 out of range. Value: 3.7133. Training min, max, SD, explained variance: -2.8548, 3.3954, 1.263, 0.0360.
4. Unknown FCFP_2 feature: -203115083: [*]:[c](:[*]):n1:[c](:[*]):[*]:[*]:c:1
5. Unknown FCFP_2 feature: -1410079687: [*]S[c]1:n:[*]:[*]:n:1:[*]
6. Unknown FCFP_2 feature: -124685461: [*]:n1:[*]:[*]:n:c:1

Feature Contribution

Top features for positive contribution

Fingerprint	Bit/Smiles	Feature Structure	Score

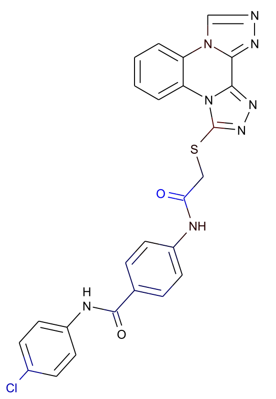

$C_{25}H_{17}ClN_8O_2S$

Molecular Weight: 528.97287

ALogP: 4.334

Rotatable Bonds: 6

Acceptors: 7

Donors: 2

Model Prediction

Prediction: 0.133

Unit: g/kg_body_weight

Mahalanobis Distance: 13.535

Mahalanobis Distance p-value: 1.16e-011

Mahalanobis Distance: The Mahalanobis distance (MD) is a generalization of the Euclidean distance that accounts for correlations among the X properties. It is calculated as the distance to the center of the training data. The larger the MD, the less trustworthy the prediction.
Mahalanobis Distance p-value: The p-value gives the fraction of training data with an MD greater than or equal to the one for the given sample, assuming normally distributed data. The smaller the p-value, the less trustworthy the prediciton. For highly non-normal X properties (e.g., fingerprints), the MD p-value is wildly inaccurate.

Structural Similar Compounds

Name	C.I.PIGMENT RED 23	SALICYLAZOSULFAPYRIDINE	RESERPINE
Structure	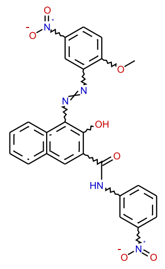	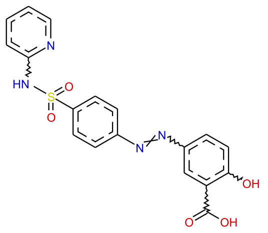	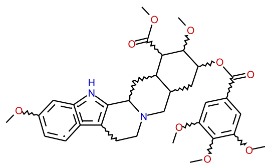
Actual Endpoint (-log C)	2.30052	3.375	6.13118
Predicted Endpoint (-log C)	3.55333	2.80292	4.38304
Distance	0.847	1.065	1.178
Reference	NCI/NTP TR-411	NCI/NTP TR-457	NCI/NTP TR-193

Model Applicability

Unknown features are fingerprint features in the query molecule, but not found in the training set.

1. Num_AromaticRings out of range. Value: 6. Training min, max, mean, SD: 0, 4, 1.1685, 0.8469.
2. OPS PC5 out of range. Value: 5.3168. Training min, max, SD, explained variance: -3.3892, 5.0834, 1.644, 0.0611.
3. OPS PC9 out of range. Value: 3.7133. Training min, max, SD, explained variance: -2.8548, 3.3954, 1.263, 0.0360.
4. Unknown FCFP_2 feature: -203115083: [*]:[c](:[*]):n1:[c](:[*]):[*]:[*]:c:1
5. Unknown FCFP_2 feature: -1410079687: [*]S[c]1:n:[*]:[*]:n:1:[*]
6. Unknown FCFP_2 feature: -124685461: [*]:n1:[*]:[*]:n:c:1

Feature Contribution

Top features for positive contribution

Fingerprint	Bit/Smiles	Feature Structure	Score

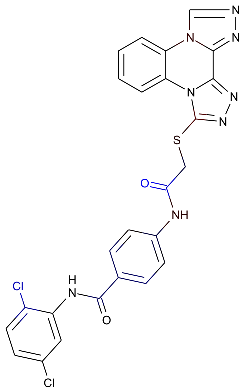

$C_{25}H_{16}Cl_2N_8O_2S$

Molecular Weight: 563.41793

ALogP: 4.999

Rotatable Bonds: 6

Acceptors: 7

Donors: 2

Model Prediction

Prediction: 0.105

Unit: g/kg_body_weight

Mahalanobis Distance: 13.351

Mahalanobis Distance p-value: 2.74e-011

Mahalanobis Distance: The Mahalanobis distance (MD) is a generalization of the Euclidean distance that accounts for correlations among the X properties. It is calculated as the distance to the center of the training data. The larger the MD, the less trustworthy the prediction.

Mahalanobis Distance p-value: The p-value gives the fraction of training data with an MD greater than or equal to the one for the given sample, assuming normally distributed data. The smaller the p-value, the less trustworthy the prediciton. For highly non-normal X properties (e.g., fingerprints), the MD p-value is wildly inaccurate.

Structural Similar Compounds

Name	C.I.PIGMENT RED 23	SALICYLAZOSULFAPYRIDINE	RESERPINE
Structure	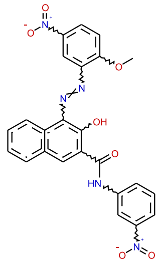	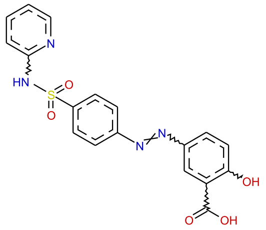	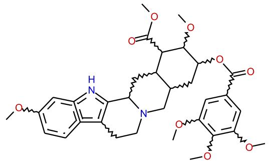
Actual Endpoint (-log C)	2.30052	3.375	6.13118
Predicted Endpoint (-log C)	3.55333	2.80292	4.38304
Distance	0.850	1.094	1.173
Reference	NCI/NTP TR-411	NCI/NTP TR-457	NCI/NTP TR-193

Model Applicability

Unknown features are fingerprint features in the query molecule, but not found in the training set.

1. Num_AromaticRings out of range. Value: 6. Training min, max, mean, SD: 0, 4, 1.1685, 0.8469.
2. OPS PC5 out of range. Value: 5.4347. Training min, max, SD, explained variance: -3.3892, 5.0834, 1.644, 0.0611.
3. OPS PC9 out of range. Value: 3.7797. Training min, max, SD, explained variance: -2.8548, 3.3954, 1.263, 0.0360.
4. Unknown FCFP_2 feature: -203115083: [*]:[c](:[*]):n1:[c](:[*]):[*]:[*]:c:1
5. Unknown FCFP_2 feature: -1410079687: [*]S[c]1:n:[*]:[*]:n:1:[*]
6. Unknown FCFP_2 feature: -124685461: [*]:n1:[*]:[*]:n:c:1

Feature Contribution

Top features for positive contribution			
Fingerprint	Bit/Smiles	Feature Structure	Score

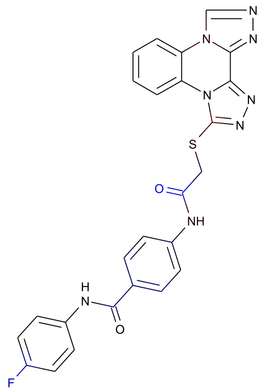

$C_{25}H_{17}FN_8O_2S$

Molecular Weight: 512.51828

ALogP: 3.875

Rotatable Bonds: 6

Acceptors: 7

Donors: 2

Model Prediction

Prediction: 0.144

Unit: g/kg_body_weight

Mahalanobis Distance: 13.697

Mahalanobis Distance p-value: 5.45e-012

Mahalanobis Distance: The Mahalanobis distance (MD) is a generalization of the Euclidean distance that accounts for correlations among the X properties. It is calculated as the distance to the center of the training data. The larger the MD, the less trustworthy the prediction.

Mahalanobis Distance p-value: The p-value gives the fraction of training data with an MD greater than or equal to the one for the given sample, assuming normally distributed data. The smaller the p-value, the less trustworthy the prediciton. For highly non-normal X properties (e.g., fingerprints), the MD p-value is wildly inaccurate.

Structural Similar Compounds

Name	C.I.PIGMENT RED 23	SALICYLAZOSULFAPYRIDINE	RESERPINE
Structure	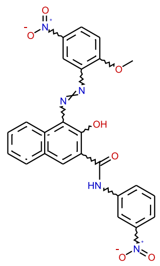	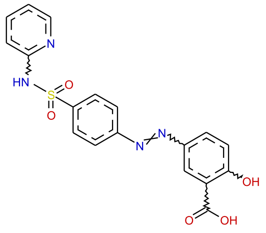	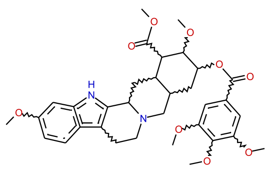
Actual Endpoint (-log C)	2.30052	3.375	6.13118
Predicted Endpoint (-log C)	3.55333	2.80292	4.38304
Distance	0.853	1.053	1.184
Reference	NCI/NTP TR-411	NCI/NTP TR-457	NCI/NTP TR-193

Model Applicability

Unknown features are fingerprint features in the query molecule, but not found in the training set.

1. Num_AromaticRings out of range. Value: 6. Training min, max, mean, SD: 0, 4, 1.1685, 0.8469.
2. OPS PC5 out of range. Value: 5.2548. Training min, max, SD, explained variance: -3.3892, 5.0834, 1.644, 0.0611.
3. OPS PC9 out of range. Value: 3.6777. Training min, max, SD, explained variance: -2.8548, 3.3954, 1.263, 0.0360.
4. Unknown FCFP_2 feature: -203115083: [*]:[c](:[*]):n1:[c](:[*]):[*]:[*]:c:1
5. Unknown FCFP_2 feature: -1410079687: [*]S[c]1:n:[*]:[*]:n:1:[*]
6. Unknown FCFP_2 feature: -124685461: [*]:n1:[*]:[*]:n:c:1

Feature Contribution

Top features for positive contribution

Fingerprint	Bit/Smiles	Feature Structure	Score

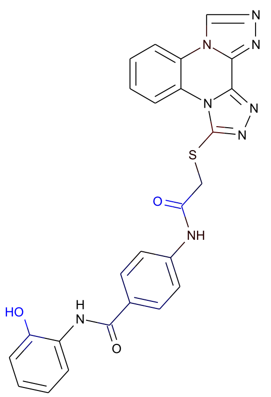

$C_{25}H_{18}N_8O_3S$

Molecular Weight: 510.52722

ALogP: 3.428

Rotatable Bonds: 6

Acceptors: 8

Donors: 3

Model Prediction

Prediction: 0.362

Unit: g/kg_body_weight

Mahalanobis Distance: 13.216

Mahalanobis Distance p-value: 5.13e-011

Mahalanobis Distance: The Mahalanobis distance (MD) is a generalization of the Euclidean distance that accounts for correlations among the X properties. It is calculated as the distance to the center of the training data. The larger the MD, the less trustworthy the prediction.
Mahalanobis Distance p-value: The p-value gives the fraction of training data with an MD greater than or equal to the one for the given sample, assuming normally distributed data. The smaller the p-value, the less trustworthy the prediciton. For highly non-normal X properties (e.g., fingerprints), the MD p-value is wildly inaccurate.

Structural Similar Compounds

Name	C.I.PIGMENT RED 23	SALICYLAZOSULFAPYRIDINE	RESERPINE
Structure	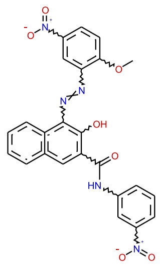	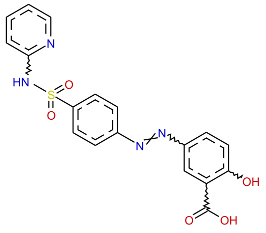	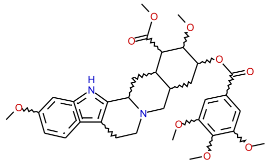
Actual Endpoint (-log C)	2.30052	3.375	6.13118
Predicted Endpoint (-log C)	3.55333	2.80292	4.38304
Distance	0.751	0.965	1.251
Reference	NCI/NTP TR-411	NCI/NTP TR-457	NCI/NTP TR-193

Model Applicability

Unknown features are fingerprint features in the query molecule, but not found in the training set.

1. Num_AromaticRings out of range. Value: 6. Training min, max, mean, SD: 0, 4, 1.1685, 0.8469.
2. Unknown FCFP_2 feature: -203115083: [*]:[c](:[*]):n1:[c](:[*]):[*]:[*]:c:1
3. Unknown FCFP_2 feature: -1410079687: [*]S[c]1:n:[*]:[*]:n:1:[*]
4. Unknown FCFP_2 feature: -124685461: [*]:n1:[*]:[*]:n:c:1

Feature Contribution

Top features for positive contribution			
**Fingerprint**	**Bit/Smiles**	**Feature Structure**	**Score**
FCFP_2	1036089772	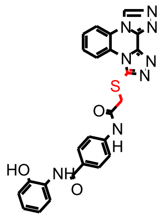 [*]CS[c](:[*]):[*]	0.075

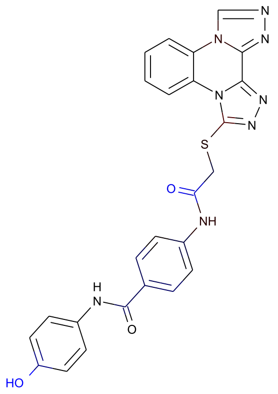

$C_{25}H_{18}N_{8}O_{3}S$
Molecular Weight: 510.52722
ALogP: 3.428
Rotatable Bonds: 6
Acceptors: 8
Donors: 3

Model Prediction

Prediction: 0.362
Unit: g/kg_body_weight
Mahalanobis Distance: 13.216
Mahalanobis Distance p-value: 5.13e-011

Mahalanobis Distance: The Mahalanobis distance (MD) is a generalization of the Euclidean distance that accounts for correlations among the X properties. It is calculated as the distance to the center of the training data. The larger the MD, the less trustworthy the prediction.
Mahalanobis Distance p-value: The p-value gives the fraction of training data with an MD greater than or equal to the one for the given sample, assuming normally distributed data. The smaller the p-value, the less trustworthy the prediciton. For highly non-normal X properties (e.g., fingerprints), the MD p-value is wildly inaccurate.

Structural Similar Compounds

Name	C.I.PIGMENT RED 23	SALICYLAZOSULFAPYRIDINE	RESERPINE
Structure	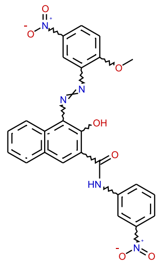	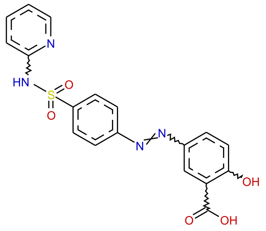	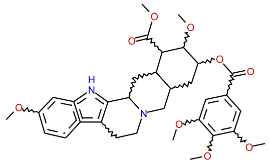
Actual Endpoint (-log C)	2.30052	3.375	6.13118
Predicted Endpoint (-log C)	3.55333	2.80292	4.38304
Distance	0.751	0.965	1.251
Reference	NCI/NTP TR-411	NCI/NTP TR-457	NCI/NTP TR-193

Model Applicability

Unknown features are fingerprint features in the query molecule, but not found in the training set.

1. Num_AromaticRings out of range. Value: 6. Training min, max, mean, SD: 0, 4, 1.1685, 0.8469.
2. Unknown FCFP_2 feature: -203115083: [*]:[c](:[*]):n1:[c](:[*]):[*]:[*]:c:1
3. Unknown FCFP_2 feature: -1410079687: [*]S[c]1:n:[*]:[*]:n:1:[*]
4. Unknown FCFP_2 feature: -124685461: [*]:n1:[*]:[*]:n:c:1

Feature Contribution

Top features for positive contribution

Fingerprint	Bit/Smiles	Feature Structure	Score
FCFP_2	1036089772	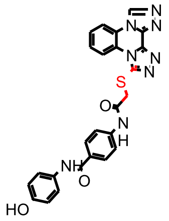 [*]CS[c](:[*]):[*]	0.075

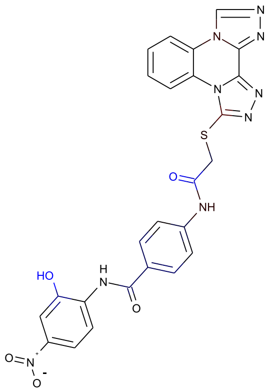

$C_{25}H_{17}N_{9}O_{5}S$

Molecular Weight: 555.52477

ALogP: 3.322

Rotatable Bonds: 7

Acceptors: 10

Donors: 3

Model Prediction

Prediction: 0.262

Unit: g/kg_body_weight

Mahalanobis Distance: 14.033

Mahalanobis Distance p-value: 1.12e-012

Mahalanobis Distance: The Mahalanobis distance (MD) is a generalization of the Euclidean distance that accounts for correlations among the X properties. It is calculated as the distance to the center of the training data. The larger the MD, the less trustworthy the prediction.
Mahalanobis Distance p-value: The p-value gives the fraction of training data with an MD greater than or equal to the one for the given sample, assuming normally distributed data. The smaller the p-value, the less trustworthy the prediciton. For highly non-normal X properties (e.g., fingerprints), the MD p-value is wildly inaccurate.

Structural Similar Compounds

Name	C.I.PIGMENT RED 23	SALICYLAZOSULFAPYRIDINE	4,4'-DIAMINO-2,2'-STILBENEDISULFONIC ACID.2NaSALT
Structure	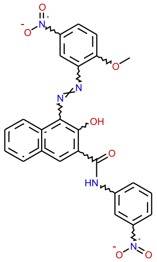	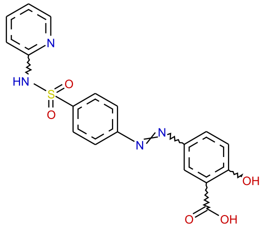	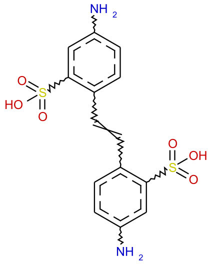
Actual Endpoint (-log C)	2.30052	3.375	2.50759
Predicted Endpoint (-log C)	3.55333	2.80292	3.26068
Distance	0.827	1.112	1.348
Reference	NCI/NTP TR-411	NCI/NTP TR-457	NCI/NTP TR-412

Model Applicability

Unknown features are fingerprint features in the query molecule, but not found in the training set.

1. Num_AromaticRings out of range. Value: 6. Training min, max, mean, SD: 0, 4, 1.1685, 0.8469.
2. Molecular_PolarSurfaceArea out of range. Value: 209.92. Training min, max, mean, SD: 0, 201.84, 63.052, 40.7.
3. OPS PC9 out of range. Value: 3.4258. Training min, max, SD, explained variance: -2.8548, 3.3954, 1.263, 0.0360.
4. Unknown FCFP_2 feature: 8: [*][N+](=[*])[*]
5. Unknown FCFP_2 feature: 5: [*][O-]
6. Unknown FCFP_2 feature: -203115083: [*]:[c](:[*]):n1:[c](:[*]):[*]:[*]:c:1
7. Unknown FCFP_2 feature: -1410079687: [*]S[c]1:n:[*]:[*]:n:1:[*]
8. Unknown FCFP_2 feature: -124685461: [*]:n1:[*]:[*]:n:c:1
9. Unknown FCFP_2 feature: -828984032: [*][N+](=[*])[c](:c:[*]):c:[*]
10. Unknown FCFP_2 feature: -1338588315: [*]:[c](:[*])[N+](=O)[O-]
11. Unknown FCFP_2 feature: 1872392852: [*][N+](=O)[*]
12. Unknown FCFP_2 feature: 260476081: [*][N+](=[*])[O-]

Feature Contribution

Top features for positive contribution

Fingerprint	Bit/Smiles	Feature Structure	Score

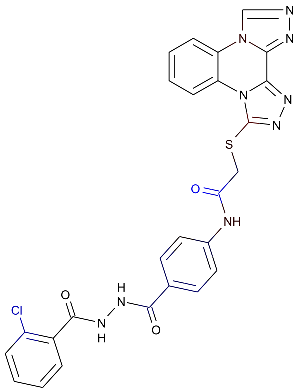

$C_{26}H_{18}ClN_9O_3S$

Molecular Weight: 571.99761

ALogP: 3.591

Rotatable Bonds: 7

Acceptors: 8

Donors: 3

Structural Similar Compounds

Name	C.I.PIGMENT RED 23	SALICYLAZOSULFAPYRIDINE	RESERPINE
Structure	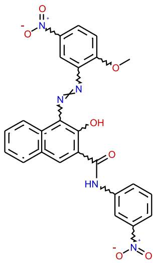	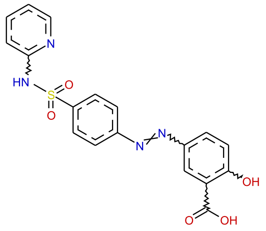	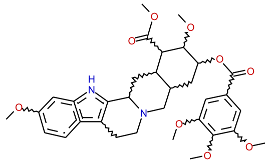
Actual Endpoint (-log C)	2.30052	3.375	6.13118
Predicted Endpoint (-log C)	3.55333	2.80292	4.38304
Distance	0.835	1.073	1.241
Reference	NCI/NTP TR-411	NCI/NTP TR-457	NCI/NTP TR-193

Model Prediction

Prediction: 0.143

Unit: g/kg_body_weight

Mahalanobis Distance: 14.058

Mahalanobis Distance p-value: 9.99e-013

Mahalanobis Distance: The Mahalanobis distance (MD) is a generalization of the Euclidean distance that accounts for correlations among the X properties. It is calculated as the distance to the center of the training data. The larger the MD, the less trustworthy the prediction.
Mahalanobis Distance p-value: The p-value gives the fraction of training data with an MD greater than or equal to the one for the given sample, assuming normally distributed data. The smaller the p-value, the less trustworthy the prediciton. For highly non-normal X properties (e.g., fingerprints), the MD p-value is wildly inaccurate.

Model Applicability

Unknown features are fingerprint features in the query molecule, but not found in the training set.

1. Num_AromaticRings out of range. Value: 6. Training min, max, mean, SD: 0, 4, 1.1685, 0.8469.
2. OPS PC5 out of range. Value: 5.7778. Training min, max, SD, explained variance: -3.3892, 5.0834, 1.644, 0.0611.
3. OPS PC9 out of range. Value: 3.86. Training min, max, SD, explained variance: -2.8548, 3.3954, 1.263, 0.0360.
4. Unknown FCFP_2 feature: -885461129: [*]NNC(=[*])[*]
5. Unknown FCFP_2 feature: -203115083: [*]:[c](:[*]):n1:[c](:[*]):[*]:[*]:c:1
6. Unknown FCFP_2 feature: -1410079687: [*]S[c]1:n:[*]:[*]:n:1:[*]
7. Unknown FCFP_2 feature: -124685461: [*]:n1:[*]:[*]:n:c:1

Feature Contribution

Top features for positive contribution

Fingerprint	Bit/Smiles	Feature Structure	Score

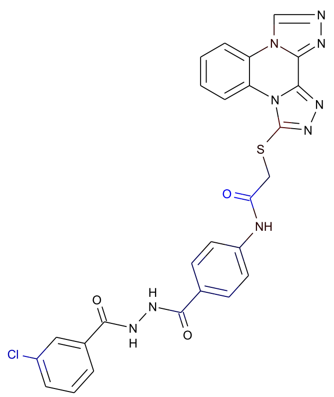

$C_{26}H_{18}ClN_9O_3S$
Molecular Weight: 571.99761
ALogP: 3.591
Rotatable Bonds: 7
Acceptors: 8
Donors: 3

Model Prediction

Prediction: 0.143
Unit: g/kg_body_weight
Mahalanobis Distance: 14.058
Mahalanobis Distance p-value: 9.99e-013

Mahalanobis Distance: The Mahalanobis distance (MD) is a generalization of the Euclidean distance that accounts for correlations among the X properties. It is calculated as the distance to the center of the training data. The larger the MD, the less trustworthy the prediction.
Mahalanobis Distance p-value: The p-value gives the fraction of training data with an MD greater than or equal to the one for the given sample, assuming normally distributed data. The smaller the p-value, the less trustworthy the prediciton. For highly non-normal X properties (e.g., fingerprints), the MD p-value is wildly inaccurate.

Structural Similar Compounds

Name	C.I.PIGMENT RED 23	SALICYLAZOSULFAPYRIDINE	RESERPINE
Structure	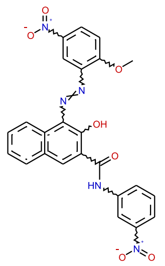	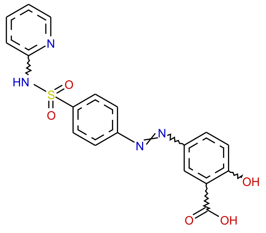	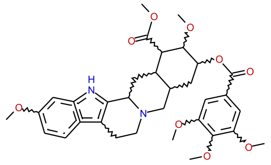
Actual Endpoint (-log C)	2.30052	3.375	6.13118
Predicted Endpoint (-log C)	3.55333	2.80292	4.38304
Distance	0.835	1.073	1.241
Reference	NCI/NTP TR-411	NCI/NTP TR-457	NCI/NTP TR-193

Model Applicability

Unknown features are fingerprint features in the query molecule, but not found in the training set.

1. Num_AromaticRings out of range. Value: 6. Training min, max, mean, SD: 0, 4, 1.1685, 0.8469.
2. OPS PC5 out of range. Value: 5.7778. Training min, max, SD, explained variance: -3.3892, 5.0834, 1.644, 0.0611.
3. OPS PC9 out of range. Value: 3.86. Training min, max, SD, explained variance: -2.8548, 3.3954, 1.263, 0.0360.
4. Unknown FCFP_2 feature: -885461129: [*]NNC(=[*])[*]
5. Unknown FCFP_2 feature: -203115083: [*]:[c](:[*]):n1:[c](:[*]):[*]:[*]:c:1
6. Unknown FCFP_2 feature: -1410079687: [*]S[c]1:n:[*]:[*]:n:1:[*]
7. Unknown FCFP_2 feature: -124685461: [*]:n1:[*]:[*]:n:c:1

Feature Contribution

Top features for positive contribution

Fingerprint	Bit/Smiles	Feature Structure	Score

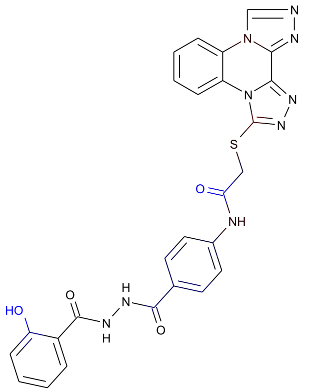

$C_{26}H_{19}N_9O_4S$
Molecular Weight: 553.55195
ALogP: 2.685
Rotatable Bonds: 7
Acceptors: 9
Donors: 4

Structural Similar Compounds

Name	C.I.PIGMENT RED 23	SALICYLAZOSULFAPYRIDINE	4,4'-DIAMINO-2,2'-STILBENEDISULFONIC ACID.2NaSALT
Structure	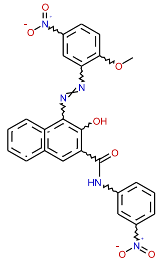	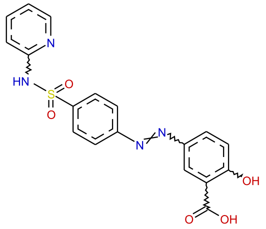	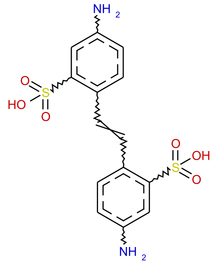
Actual Endpoint (-log C)	2.30052	3.375	2.50759
Predicted Endpoint (-log C)	3.55333	2.80292	3.26068
Distance	0.841	1.051	1.295
Reference	NCI/NTP TR-411	NCI/NTP TR-457	NCI/NTP TR-412

Model Prediction

Prediction: 0.391
Unit: g/kg_body_weight
Mahalanobis Distance: 13.669
Mahalanobis Distance p-value: 6.23e-012

Mahalanobis Distance: The Mahalanobis distance (MD) is a generalization of the Euclidean distance that accounts for correlations among the X properties. It is calculated as the distance to the center of the training data. The larger the MD, the less trustworthy the prediction.
Mahalanobis Distance p-value: The p-value gives the fraction of training data with an MD greater than or equal to the one for the given sample, assuming normally distributed data. The smaller the p-value, the less trustworthy the prediciton. For highly non-normal X properties (e.g., fingerprints), the MD p-value is wildly inaccurate.

Model Applicability

Unknown features are fingerprint features in the query molecule, but not found in the training set.

1. Num_AromaticRings out of range. Value: 6. Training min, max, mean, SD: 0, 4, 1.1685, 0.8469.
2. OPS PC9 out of range. Value: 3.5069. Training min, max, SD, explained variance: -2.8548, 3.3954, 1.263, 0.0360.
3. Unknown FCFP_2 feature: -885461129: [*]NNC(=[*])[*]
4. Unknown FCFP_2 feature: -203115083: [*]:[c](:[*]):n1:[c](:[*]):[*]:[*]:c:1
5. Unknown FCFP_2 feature: -1410079687: [*]S[c]1:n:[*]:[*]:n:1:[*]
6. Unknown FCFP_2 feature: -124685461: [*]:n1:[*]:[*]:n:c:1

Feature Contribution

Top features for positive contribution			
Fingerprint	Bit/Smiles	Feature Structure	Score

Sorafenib

TOPKAT_Rat_Maximum_Tolerated_Dose_Feed

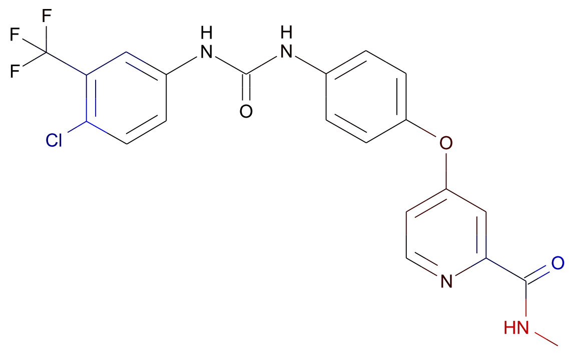

$C_{21}H_{16}ClF_3N_4O_3$
Molecular Weight: 464.82494
ALogP: 4.175
Rotatable Bonds: 6
Acceptors: 4
Donors: 3

Model Prediction

Prediction: 0.089
Unit: g/kg_body_weight
Mahalanobis Distance: 12.441
Mahalanobis Distance p-value: 1.76e-009

Mahalanobis Distance: The Mahalanobis distance (MD) is a generalization of the Euclidean distance that accounts for correlations among the X properties. It is calculated as the distance to the center of the training data. The larger the MD, the less trustworthy the prediction.
Mahalanobis Distance p-value: The p-value gives the fraction of training data with an MD greater than or equal to the one for the given sample, assuming normally distributed data. The smaller the p-value, the less trustworthy the prediciton. For highly non-normal X properties (e.g., fingerprints), the MD p-value is wildly inaccurate.

Structural Similar Compounds

Name	FUROSEMIDE	PHENOLPHTHALEIN	DISPERSE YELLOW 3
Structure	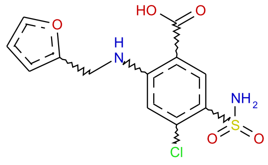	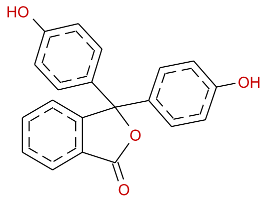	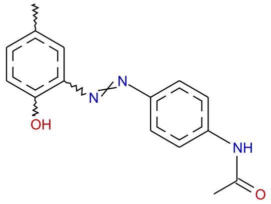
Actual Endpoint (-log C)	4.04236	2.20184	2.77703
Predicted Endpoint (-log C)	2.8614	2.8857	2.80195
Distance	0.741	0.780	0.799
Reference	NCI/NTP TR-356	NCI/NTP TR-465	NCI/NTP TR-222

Model Applicability

Unknown features are fingerprint features in the query molecule, but not found in the training set.

1. All properties and OPS components are within expected ranges.

Feature Contribution

Top features for positive contribution

Fingerprint	Bit/Smiles	Feature Structure	Score
FCFP_2	-885550502	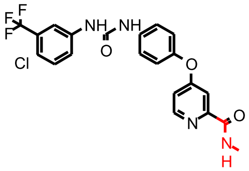 [*]CNC(=[*])[*]	0.115

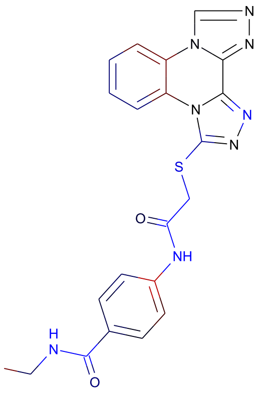

$C_{21}H_{18}N_8O_2S$

Molecular Weight: 446.48502

ALogP: 2.442

Rotatable Bonds: 6

Acceptors: 7

Donors: 2

Structural Similar Compounds

Name	BENZENESULFONIC ACID; 2;2'-(4;4'-BIPHENYLYLENEDIVINYLENE)DI-; DISODIUM SALT (Na STRIPPED)	PRASOZIN .HCl (HCl STRIPPED)	BENZOTHIAZOLE; 6-NITRO-2-(p-NITROBENZOYLAMINO)-
Structure	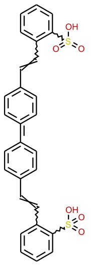	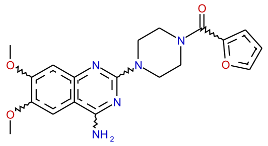	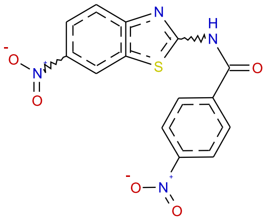
Actual Endpoint (-log C)	1.968	2.294	2.361
Predicted Endpoint (-log C)	1.72109	3.00765	2.96257
Distance	0.895	0.931	0.936
Reference	MVCRB3 2;193;73	NIIRDN 6;688;82	JPETAB 90;260;47

Model Prediction

Prediction: 2.209

Unit: g/kg_body_weight

Mahalanobis Distance: 28.199

Mahalanobis Distance p-value: 3.24e-051

Mahalanobis Distance: The Mahalanobis distance (MD) is a generalization of the Euclidean distance that accounts for correlations among the X properties. It is calculated as the distance to the center of the training data. The larger the MD, the less trustworthy the prediction.
Mahalanobis Distance p-value: The p-value gives the fraction of training data with an MD greater than or equal to the one for the given sample, assuming normally distributed data. The smaller the p-value, the less trustworthy the prediciton. For highly non-normal X properties (e.g., fingerprints), the MD p-value is wildly inaccurate.

Model Applicability

Unknown features are fingerprint features in the query molecule, but not found in the training set.

1. All properties and OPS components are within expected ranges.
2. Unknown ECFP_2 feature: 1312166648: [*]:[c](:[*]):n1:[c](:[*]):[*]:[*]:c:1
3. Unknown ECFP_2 feature: 1986731747: [*]S[c]1:n:[*]:[*]:n:1:[*]
4. Unknown FCFP_6 feature: 16: [*][c](:[*]):[*]
5. Unknown FCFP_6 feature: 1618154665: [*][c](:[*]):[cH]:[cH]:[*]
6. Unknown FCFP_6 feature: -203115083: [*]:[c](:[*]):n1:[cH]:[*]:[*]:[c]:1:[*]
7. Unknown FCFP_6 feature: -1564473960: [*]:n1:[*]:[*]:n:[c]:1:[c](:[*]):[*]
8. Unknown FCFP_6 feature: 4427049: [*]:[c]1:[*]:[*]:n:n:1
9. Unknown FCFP_6 feature: -1410079687: [*]S[c]1:n:[*]:[*]:n:1:[*]
10. Unknown FCFP_6 feature: -124685461: [*]:n1:[*]:[*]:n:[cH]:1

Feature Contribution

Top features for positive contribution

Fingerprint	Bit/Smiles	Feature Structure	Score

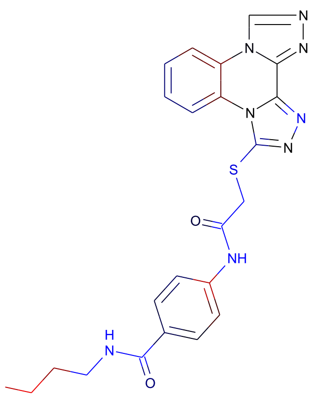

$C_{23}H_{22}N_8O_2S$
Molecular Weight: 474.53818
ALogP: 3.422
Rotatable Bonds: 8
Acceptors: 7
Donors: 2

Structural Similar Compounds

Name	BENZENESULFONIC ACID; 2;2'-(4;4'-BIPHENYLYLENEDIVINYLENE)DI-; DISODIUM SALT (Na STRIPPED)	ACEMETACIN	PRASOZIN .HCl (HCl STRIPPED)
Structure	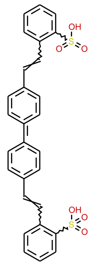	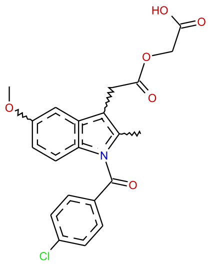	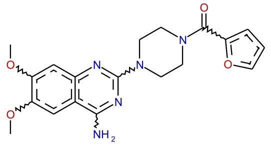
Actual Endpoint (-log C)	1.968	4.235	2.294
Predicted Endpoint (-log C)	1.72109	3.39415	3.00765
Distance	0.816	0.955	0.985
Reference	MVCRB3 2;193;73	ARZNAD 30;1398;80	NIIRDN 6;688;82

Model Prediction

Prediction: 1.900
Unit: g/kg_body_weight
Mahalanobis Distance: 29.788
Mahalanobis Distance p-value: 1.26e-062

Mahalanobis Distance: The Mahalanobis distance (MD) is a generalization of the Euclidean distance that accounts for correlations among the X properties. It is calculated as the distance to the center of the training data. The larger the MD, the less trustworthy the prediction.
Mahalanobis Distance p-value: The p-value gives the fraction of training data with an MD greater than or equal to the one for the given sample, assuming normally distributed data. The smaller the p-value, the less trustworthy the prediciton. For highly non-normal X properties (e.g., fingerprints), the MD p-value is wildly inaccurate.

Model Applicability

Unknown features are fingerprint features in the query molecule, but not found in the training set.

1. All properties and OPS components are within expected ranges.
2. Unknown ECFP_2 feature: 1312166648: [*]:[c](:[*]):n1:[c](:[*]):[*]:[*]:c:1
3. Unknown ECFP_2 feature: 1986731747: [*]S[c]1:n:[*]:[*]:n:1:[*]
4. Unknown FCFP_6 feature: 16: [*][c](:[*]):[*]
5. Unknown FCFP_6 feature: 1618154665: [*][c](:[*]):[cH]:[cH]:[*]
6. Unknown FCFP_6 feature: -203115083: [*]:[c](:[*]):n1:[cH]:[*]:[*]:[c]:1:[*]
7. Unknown FCFP_6 feature: -1564473960: [*]:n1:[*]:[*]:n:[c]:1:[c](:[*]):[*]
8. Unknown FCFP_6 feature: 4427049: [*]:[c]1:[*]:[*]:n:n:1
9. Unknown FCFP_6 feature: -1410079687: [*]S[c]1:n:[*]:[*]:n:1:[*]
10. Unknown FCFP_6 feature: -124685461: [*]:n1:[*]:[*]:n:[cH]:1

Feature Contribution

Top features for positive contribution			
Fingerprint	Bit/Smiles	Feature Structure	Score

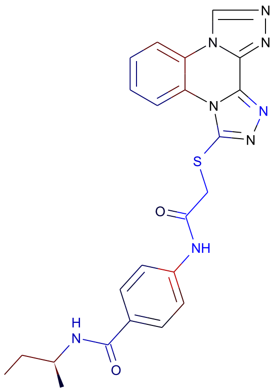

$C_{23}H_{22}N_8O_2S$

Molecular Weight: 474.53818

ALogP: 3.343

Rotatable Bonds: 7

Acceptors: 7

Donors: 2

Structural Similar Compounds

Name	BENZENESULFONIC ACID; 2;2'-(4;4'-BIPHENYLYLENEDIVINYLENE)DI-; DISODIUM SALT (Na STRIPPED)	ACEMETACIN	BENZOTHIAZOLE; 6-NITRO-2-(p-NITROBENZOYLAMINO)-
Structure	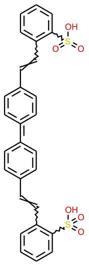	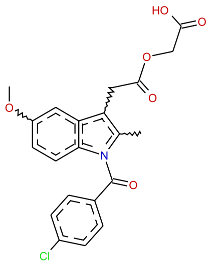	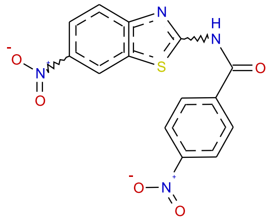
Actual Endpoint (-log C)	1.968	4.235	2.361
Predicted Endpoint (-log C)	1.72109	3.39415	2.96257
Distance	0.814	0.949	0.965
Reference	MVCRB3 2;193;73	ARZNAD 30;1398;80	JPETAB 90;260;47

Model Prediction

Prediction: 1.899

Unit: g/kg_body_weight

Mahalanobis Distance: 29.713

Mahalanobis Distance p-value: 4.57e-062

Mahalanobis Distance: The Mahalanobis distance (MD) is a generalization of the Euclidean distance that accounts for correlations among the X properties. It is calculated as the distance to the center of the training data. The larger the MD, the less trustworthy the prediction.
Mahalanobis Distance p-value: The p-value gives the fraction of training data with an MD greater than or equal to the one for the given sample, assuming normally distributed data. The smaller the p-value, the less trustworthy the prediciton. For highly non-normal X properties (e.g., fingerprints), the MD p-value is wildly inaccurate.

Model Applicability

Unknown features are fingerprint features in the query molecule, but not found in the training set.

1. All properties and OPS components are within expected ranges.
2. Unknown ECFP_2 feature: 1312166648: [*]:[c](:[*]):n1:[c](:[*]):[*]:[*]:c:1
3. Unknown ECFP_2 feature: 1986731747: [*]S[c]1:n:[*]:[*]:n:1:[*]
4. Unknown FCFP_6 feature: 16: [*][c](:[*]):[*]
5. Unknown FCFP_6 feature: 1618154665: [*][c](:[*]):[cH]:[cH]:[*]
6. Unknown FCFP_6 feature: -203115083: [*]:[c](:[*]):n1:[cH]:[*]:[*]:[c]:1:[*]
7. Unknown FCFP_6 feature: -1564473960: [*]:n1:[*]:[*]:n:[c]:1:[c](:[*]):[*]
8. Unknown FCFP_6 feature: 4427049: [*]:[c]1:[*]:[*]:n:n:1
9. Unknown FCFP_6 feature: -1410079687: [*]S[c]1:n:[*]:[*]:n:1:[*]
10. Unknown FCFP_6 feature: -124685461: [*]:n1:[*]:[*]:n:[cH]:1

Feature Contribution

Top features for positive contribution			
**Fingerprint**	**Bit/Smiles**	**Feature Structure**	**Score**

ter. butyl.cdx

TOPKAT_Rat_Oral_LD50

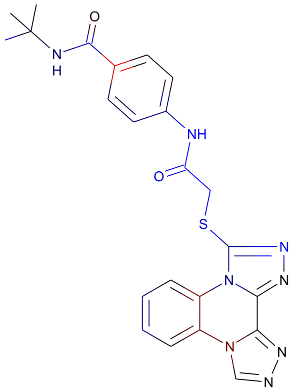

$C_{23}H_{22}N_8O_2S$
Molecular Weight: 474.53818
ALogP: 3.025
Rotatable Bonds: 6
Acceptors: 7
Donors: 2

Model Prediction

Prediction: 1.978
Unit: g/kg_body_weight
Mahalanobis Distance: 28.686
Mahalanobis Distance p-value: 1.29e-054

Mahalanobis Distance: The Mahalanobis distance (MD) is a generalization of the Euclidean distance that accounts for correlations among the X properties. It is calculated as the distance to the center of the training data. The larger the MD, the less trustworthy the prediction.
Mahalanobis Distance p-value: The p-value gives the fraction of training data with an MD greater than or equal to the one for the given sample, assuming normally distributed data. The smaller the p-value, the less trustworthy the prediciton. For highly non-normal X properties (e.g., fingerprints), the MD p-value is wildly inaccurate.

Structural Similar Compounds

Name	BENZENESULFONIC ACID; 2;2'-(4;4'-BIPHENYLYLENEDIVINYLENE)DI-; DISODIUM SALT (Na STRIPPED)	PRASOZIN .HCl (HCl STRIPPED)	BENZOTHIAZOLE; 6-NITRO-2-(p-NITROBENZOYLAMINO)-
Structure	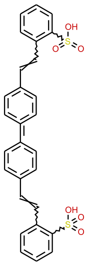	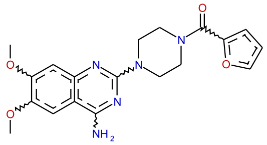	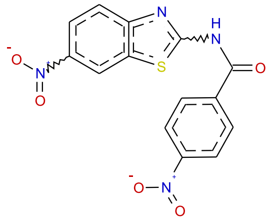
Actual Endpoint (-log C)	1.968	2.294	2.361
Predicted Endpoint (-log C)	1.72109	3.00765	2.96257
Distance	0.837	0.951	0.953
Reference	MVCRB3 2;193;73	NIIRDN 6;688;82	JPETAB 90;260;47

Model Applicability

Unknown features are fingerprint features in the query molecule, but not found in the training set.

1. All properties and OPS components are within expected ranges.
2. Unknown ECFP_2 feature: 1312166648: [*]:[c](:[*]):n1:[c](:[*]):[*]:[*]:c:1
3. Unknown ECFP_2 feature: 1986731747: [*]S[c]1:n:[*]:[*]:n:1:[*]
4. Unknown FCFP_6 feature: 16: [*][c](:[*]):[*]
5. Unknown FCFP_6 feature: 1618154665: [*][c](:[*]):[cH]:[cH]:[*]
6. Unknown FCFP_6 feature: -203115083: [*][c]1:[*]:[*]:[c](:[*]):n:1:[c](:[*]):[*]
7. Unknown FCFP_6 feature: -1564473960: [*]:n1:[*]:[*]:n:[c]:1:[c](:[*]):[*]
8. Unknown FCFP_6 feature: 4427049: [*][c]1:[*]:[*]:n:n:1
9. Unknown FCFP_6 feature: -1410079687: [*]S[c]1:n:[*]:[*]:n:1:[*]
10. Unknown FCFP_6 feature: -124685461: [*]:n1:[*]:[*]:n:[cH]:1

Feature Contribution

Top features for positive contribution			
**Fingerprint**	**Bit/Smiles**	**Feature Structure**	**Score**

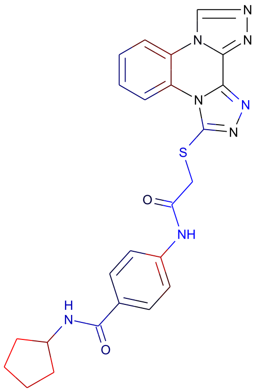

$C_{24}H_{22}N_8O_2S$

Molecular Weight: 486.54888

ALogP: 3.496

Rotatable Bonds: 6

Acceptors: 7

Donors: 2

Model Prediction

Prediction: 0.729

Unit: g/kg_body_weight

Mahalanobis Distance: 28.396

Mahalanobis Distance p-value: 1.4e-052

Mahalanobis Distance: The Mahalanobis distance (MD) is a generalization of the Euclidean distance that accounts for correlations among the X properties. It is calculated as the distance to the center of the training data. The larger the MD, the less trustworthy the prediction.
Mahalanobis Distance p-value: The p-value gives the fraction of training data with an MD greater than or equal to the one for the given sample, assuming normally distributed data. The smaller the p-value, the less trustworthy the prediciton. For highly non-normal X properties (e.g., fingerprints), the MD p-value is wildly inaccurate.

Structural Similar Compounds

Name	BENZENESULFONIC ACID; 2;2'-(4;4'-BIPHENYLYLENEDIVINYLENE)DI-; DISODIUM SALT (Na STRIPPED)	ACEMETACIN	PRASOZIN .HCl (HCl STRIPPED)
Structure	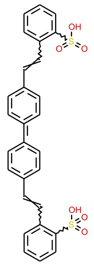	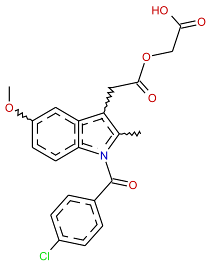	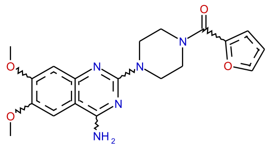
Actual Endpoint (-log C)	1.968	4.235	2.294
Predicted Endpoint (-log C)	1.72109	3.39415	3.00765
Distance	0.805	0.961	0.963
Reference	MVCRB3 2;193;73	ARZNAD 30;1398;80	NIIRDN 6;688;82

Model Applicability

Unknown features are fingerprint features in the query molecule, but not found in the training set.

1. All properties and OPS components are within expected ranges.
2. Unknown ECFP_2 feature: 1312166648: [*]:[c](:[*]):n1:[c](:[*]):[*]:[*]:c:1
3. Unknown ECFP_2 feature: 1986731747: [*]S[c]1:n:[*]:[*]:n:1:[*]
4. Unknown FCFP_6 feature: 16: [*][c](:[*]):[*]
5. Unknown FCFP_6 feature: 1618154665: [*][c](:[*]):[cH]:[cH]:[*]
6. Unknown FCFP_6 feature: -203115083: [*]:[c](:[*]):n1:[cH]:[*]:[*]:[c]:1:[*]
7. Unknown FCFP_6 feature: -1564473960: [*]:n1:[*]:[*]:n:[c]:1:[c](:[*]):[*]
8. Unknown FCFP_6 feature: 4427049: [*]:[c]1:[*]:[*]:n:n:1
9. Unknown FCFP_6 feature: -1410079687: [*]S[c]1:n:[*]:[*]:n:1:[*]
10. Unknown FCFP_6 feature: -124685461: [*]:n1:[*]:[*]:n:[cH]:1

Feature Contribution

Top features for positive contribution			
Fingerprint	Bit/Smiles	Feature Structure	Score

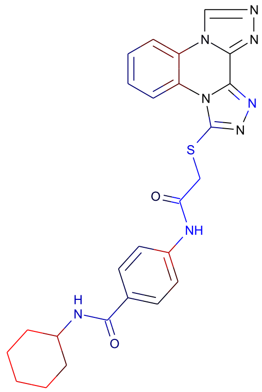

$C_{25}H_{24}N_8O_2S$
Molecular Weight: 500.57546
ALogP: 3.952
Rotatable Bonds: 6
Acceptors: 7
Donors: 2

Structural Similar Compounds

Name	BENZENESULFONIC ACID; 2;2'-(4;4'-BIPHENYLYLENEDIVINYLENE)DI-; DISODIUM SALT (Na STRIPPED)	ACEMETACIN	BENZOTHIAZOLE; 6-NITRO-2-(p-NITROBENZOYLAMINO)-
Structure	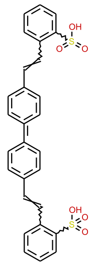	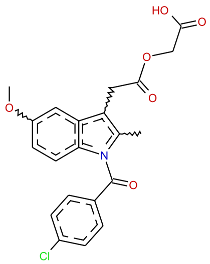	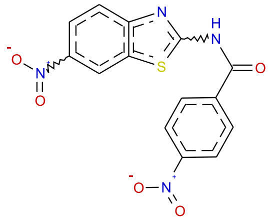
Actual Endpoint (-log C)	1.968	4.235	2.361
Predicted Endpoint (-log C)	1.72109	3.39415	2.96257
Distance	0.773	0.965	0.984
Reference	MVCRB3 2;193;73	ARZNAD 30;1398;80	JPETAB 90;260;47

Model Prediction

Prediction: 0.619
Unit: g/kg_body_weight
Mahalanobis Distance: 28.720
Mahalanobis Distance p-value: 7.39e-055

Mahalanobis Distance: The Mahalanobis distance (MD) is a generalization of the Euclidean distance that accounts for correlations among the X properties. It is calculated as the distance to the center of the training data. The larger the MD, the less trustworthy the prediction.
Mahalanobis Distance p-value: The p-value gives the fraction of training data with an MD greater than or equal to the one for the given sample, assuming normally distributed data. The smaller the p-value, the less trustworthy the prediciton. For highly non-normal X properties (e.g., fingerprints), the MD p-value is wildly inaccurate.

Model Applicability

Unknown features are fingerprint features in the query molecule, but not found in the training set.

1. All properties and OPS components are within expected ranges.
2. Unknown ECFP_2 feature: 1312166648: [*]:[c](:[*]):n1:[c](:[*]):[*]:[*]:c:1
3. Unknown ECFP_2 feature: 1986731747: [*]S[c]1:n:[*]:[*]:n:1:[*]
4. Unknown FCFP_6 feature: 16: [*][c](:[*]):[*]
5. Unknown FCFP_6 feature: 1618154665: [*][c](:[*]):[cH]:[cH]:[*]
6. Unknown FCFP_6 feature: -203115083: [*]:[c](:[*]):n1:[cH]:[*]:[*]:[c]:1:[*]
7. Unknown FCFP_6 feature: -1564473960: [*]:n1:[*]:[*]:n:[c]:1:[c](:[*]):[*]
8. Unknown FCFP_6 feature: 4427049: [*]:[c]1:[*]:[*]:n:n:1
9. Unknown FCFP_6 feature: -1410079687: [*]S[c]1:n:[*]:[*]:n:1:[*]
10. Unknown FCFP_6 feature: -124685461: [*]:n1:[*]:[*]:n:[cH]:1

Feature Contribution

Top features for positive contribution			
**Fingerprint**	**Bit/Smiles**	**Feature Structure**	**Score**

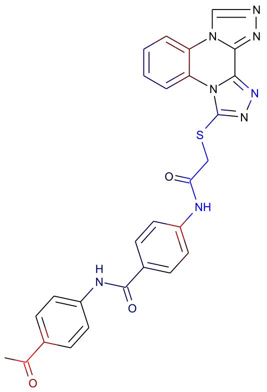

$C_{27}H_{20}N_8O_3S$
Molecular Weight: 536.56449
ALogP: 3.41
Rotatable Bonds: 7
Acceptors: 8
Donors: 2

Structural Similar Compounds

Name	BENZENESULFONIC ACID; 2;2'-(4;4'-BIPHENYLYLENEDIVINYLENE)DI-; DISODIUM SALT (Na STRIPPED)	BENZOTHIAZOLE; 6-NITRO-2-(p-NITROBENZOYLAMINO)-	AZOSEMIDE
Structure	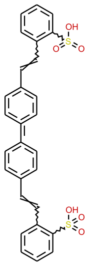	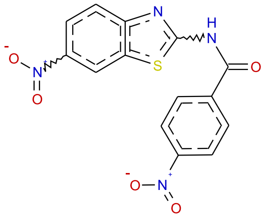	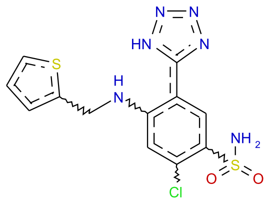
Actual Endpoint (-log C)	1.968	2.361	2.163
Predicted Endpoint (-log C)	1.72109	2.96257	2.21052
Distance	0.991	1.157	1.189
Reference	MVCRB3 2;193;73	JPETAB 90;260;47	IYKEDH 18;666;87

Model Prediction

Prediction: 1.071
Unit: g/kg_body_weight
Mahalanobis Distance: 30.866
Mahalanobis Distance p-value: 7.64e-071

Mahalanobis Distance: The Mahalanobis distance (MD) is a generalization of the Euclidean distance that accounts for correlations among the X properties. It is calculated as the distance to the center of the training data. The larger the MD, the less trustworthy the prediction.
Mahalanobis Distance p-value: The p-value gives the fraction of training data with an MD greater than or equal to the one for the given sample, assuming normally distributed data. The smaller the p-value, the less trustworthy the prediciton. For highly non-normal X properties (e.g., fingerprints), the MD p-value is wildly inaccurate.

Model Applicability

Unknown features are fingerprint features in the query molecule, but not found in the training set.

1. All properties and OPS components are within expected ranges.
2. Unknown ECFP_2 feature: 1312166648: [*]:[c](:[*]):n1:[c](:[*]):[*]:[*]:c:1
3. Unknown ECFP_2 feature: 1986731747: [*]S[c]1:n:[*]:[*]:n:1:[*]
4. Unknown FCFP_6 feature: 16: [*][c](:[*]):[*]
5. Unknown FCFP_6 feature: 1618154665: [*][c](:[*]):[cH]:[cH]:[*]
6. Unknown FCFP_6 feature: -203115083: [*]:[c](:[*]):n1:[cH]:[*]:[*]:[c]:1:[*]
7. Unknown FCFP_6 feature: -1564473960: [*]:n1:[*]:[*]:n:[c]:1:[c](:[*]):[*]
8. Unknown FCFP_6 feature: 4427049: [*]:[c]1:[*]:[*]:n:n:1
9. Unknown FCFP_6 feature: -1410079687: [*]S[c]1:n:[*]:[*]:n:1:[*]
10. Unknown FCFP_6 feature: -124685461: [*]:n1:[*]:[*]:n:[cH]:1

Feature Contribution

Top features for positive contribution			
Fingerprint	Bit/Smiles	Feature Structure	Score

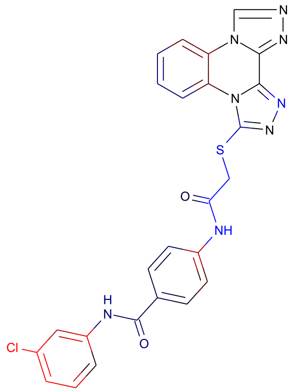

$C_{25}H_{17}ClN_8O_2S$
Molecular Weight: 528.97287
ALogP: 4.334
Rotatable Bonds: 6
Acceptors: 7
Donors: 2

Structural Similar Compounds

Name	BENZENESULFONIC ACID; 2;2'-(4;4'-BIPHENYLYLENEDIVINYLENE)DI-; DISODIUM SALT (Na STRIPPED)	ACEMETACIN	BENZOTHIAZOLE; 6-NITRO-2-(p-NITROBENZOYLAMINO)-
Structure	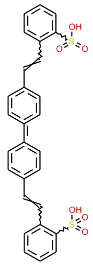	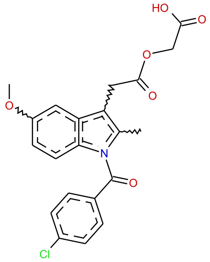	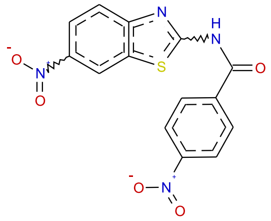
Actual Endpoint (-log C)	1.968	4.235	2.361
Predicted Endpoint (-log C)	1.72109	3.39415	2.96257
Distance	0.925	1.136	1.167
Reference	MVCRB3 2;193;73	ARZNAD 30;1398;80	JPETAB 90;260;47

Model Prediction

Prediction: 0.720
Unit: g/kg_body_weight
Mahalanobis Distance: 30.263
Mahalanobis Distance p-value: 3.39e-066

Mahalanobis Distance: The Mahalanobis distance (MD) is a generalization of the Euclidean distance that accounts for correlations among the X properties. It is calculated as the distance to the center of the training data. The larger the MD, the less trustworthy the prediction.
Mahalanobis Distance p-value: The p-value gives the fraction of training data with an MD greater than or equal to the one for the given sample, assuming normally distributed data. The smaller the p-value, the less trustworthy the prediciton. For highly non-normal X properties (e.g., fingerprints), the MD p-value is wildly inaccurate.

Model Applicability

Unknown features are fingerprint features in the query molecule, but not found in the training set.

1. All properties and OPS components are within expected ranges.
2. Unknown ECFP_2 feature: 1312166648: [*]:[c](:[*]):n1:[c](:[*]):[*]:[*]:c:1
3. Unknown ECFP_2 feature: 1986731747: [*]S[c]1:n:[*]:[*]:n:1:[*]
4. Unknown FCFP_6 feature: 16: [*][c](:[*]):[*]
5. Unknown FCFP_6 feature: 1618154665: [*][c](:[*]):[cH]:[cH]:[*]
6. Unknown FCFP_6 feature: -203115083: [*]:[c](:[*]):n1:[cH]:[*]:[*]:[c]:1:[*]
7. Unknown FCFP_6 feature: -1564473960: [*]:n1:[*]:[*]:n:[c]:1:[c](:[*]):[*]
8. Unknown FCFP_6 feature: 4427049: [*]:[c]1:[*]:[*]:n:n:1
9. Unknown FCFP_6 feature: -1410079687: [*]S[c]1:n:[*]:[*]:n:1:[*]
10. Unknown FCFP_6 feature: -124685461: [*]:n1:[*]:[*]:n:[cH]:1
11. Unknown FCFP_6 feature: 71476542: [*]:[c](:[*])Cl

Feature Contribution

Top features for positive contribution			
Fingerprint	Bit/Smiles	Feature Structure	Score

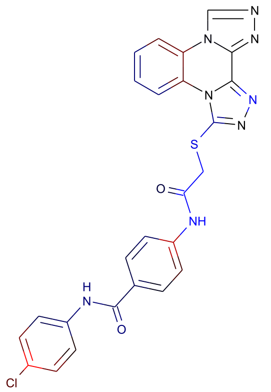

$C_{25}H_{17}ClN_{8}O_{2}S$
Molecular Weight: 528.97287
ALogP: 4.334
Rotatable Bonds: 6
Acceptors: 7
Donors: 2

Structural Similar Compounds

Name	BENZENESULFONIC ACID; 2;2'-(4;4'-BIPHENYLYLENEDIVINYLENE)DI-; DISODIUM SALT (Na STRIPPED)	ACEMETACIN	BENZOTHIAZOLE; 6-NITRO-2-(p-NITROBENZOYLAMINO)-
Structure	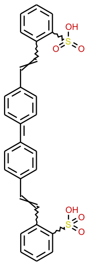	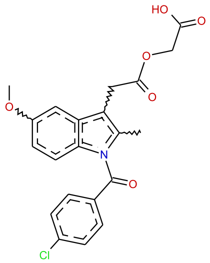	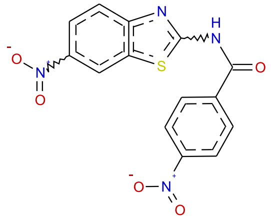
Actual Endpoint (-log C)	1.968	4.235	2.361
Predicted Endpoint (-log C)	1.72109	3.39415	2.96257
Distance	0.924	1.130	1.167
Reference	MVCRB3 2;193;73	ARZNAD 30;1398;80	JPETAB 90;260;47

Model Prediction

Prediction: 1.140
Unit: g/kg_body_weight
Mahalanobis Distance: 30.263
Mahalanobis Distance p-value: 3.39e-066

Mahalanobis Distance: The Mahalanobis distance (MD) is a generalization of the Euclidean distance that accounts for correlations among the X properties. It is calculated as the distance to the center of the training data. The larger the MD, the less trustworthy the prediction.
Mahalanobis Distance p-value: The p-value gives the fraction of training data with an MD greater than or equal to the one for the given sample, assuming normally distributed data. The smaller the p-value, the less trustworthy the prediciton. For highly non-normal X properties (e.g., fingerprints), the MD p-value is wildly inaccurate.

Model Applicability

Unknown features are fingerprint features in the query molecule, but not found in the training set.

1. All properties and OPS components are within expected ranges.
2. Unknown ECFP_2 feature: 1312166648: [*]:[c](:[*]):n1:[c](:[*]):[*]:[*]:c:1
3. Unknown ECFP_2 feature: 1986731747: [*]S[c]1:n:[*]:[*]:n:1:[*]
4. Unknown FCFP_6 feature: 16: [*][c](:[*]):[*]
5. Unknown FCFP_6 feature: 1618154665: [*][c](:[*]):[cH]:[cH]:[*]
6. Unknown FCFP_6 feature: -203115083: [*]:[c](:[*]):n1:[cH]:[*]:[*]:[c]:1:[*]
7. Unknown FCFP_6 feature: -1564473960: [*]:n1:[*]:[*]:n:[c]:1:[c](:[*]):[*]
8. Unknown FCFP_6 feature: 4427049: [*]:[c]1:[*]:[*]:n:n:1
9. Unknown FCFP_6 feature: -1410079687: [*]S[c]1:n:[*]:[*]:n:1:[*]
10. Unknown FCFP_6 feature: -124685461: [*]:n1:[*]:[*]:n:[cH]:1
11. Unknown FCFP_6 feature: 71476542: [*]:[c](:[*])Cl

Feature Contribution

Top features for positive contribution			
Fingerprint	Bit/Smiles	Feature Structure	Score

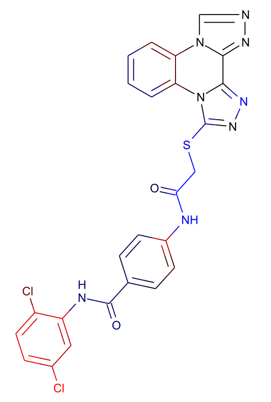

$C_{25}H_{16}Cl_2N_8O_2S$

Molecular Weight: 563.41793

ALogP: 4.999

Rotatable Bonds: 6

Acceptors: 7

Donors: 2

Structural Similar Compounds

Name	BENZENESULFONIC ACID; 2;2'-(4;4'-BIPHENYLYLENEDIVINYLENE)DI-; DISODIUM SALT (Na STRIPPED)	ACEMETACIN	ANTHRAQUINONE; 1;4-bis-(p-TOLYLAMINO)-
Structure	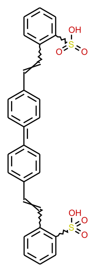	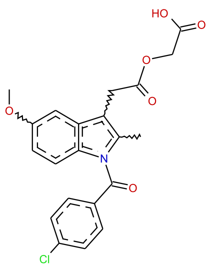	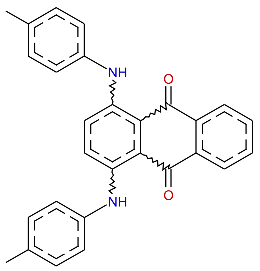
Actual Endpoint (-log C)	1.968	4.235	2.058
Predicted Endpoint (-log C)	1.72109	3.39415	1.57464
Distance	0.911	1.159	1.203
Reference	MVCRB3 2;193;73	ARZNAD 30;1398;80	85JCAE -;1330;86

Model Prediction

Prediction: 0.546

Unit: g/kg_body_weight

Mahalanobis Distance: 30.065

Mahalanobis Distance p-value: 1.06e-064

Mahalanobis Distance: The Mahalanobis distance (MD) is a generalization of the Euclidean distance that accounts for correlations among the X properties. It is calculated as the distance to the center of the training data. The larger the MD, the less trustworthy the prediction.
Mahalanobis Distance p-value: The p-value gives the fraction of training data with an MD greater than or equal to the one for the given sample, assuming normally distributed data. The smaller the p-value, the less trustworthy the prediciton. For highly non-normal X properties (e.g., fingerprints), the MD p-value is wildly inaccurate.

Model Applicability

Unknown features are fingerprint features in the query molecule, but not found in the training set.

1. All properties and OPS components are within expected ranges.
2. Unknown ECFP_2 feature: 1312166648: [*]:[c](:[*]):n1:[c](:[*]):[*]:[*]:c:1
3. Unknown ECFP_2 feature: 1986731747: [*]S[c]1:n:[*]:[*]:n:1:[*]
4. Unknown FCFP_6 feature: 16: [*][c](:[*]):[*]
5. Unknown FCFP_6 feature: 1618154665: [*][c](:[*]):[cH]:[cH]:[*]
6. Unknown FCFP_6 feature: -203115083: [*]:[c](:[*]):n1:[cH]:[*]:[*]:[c]:1:[*]
7. Unknown FCFP_6 feature: -1564473960: [*]:n1:[*]:[*]:n:[c]:1:[c](:[*]):[*]
8. Unknown FCFP_6 feature: 4427049: [*]:[c]1:[*]:[*]:n:n:1
9. Unknown FCFP_6 feature: -1410079687: [*]S[c]1:n:[*]:[*]:n:1:[*]
10. Unknown FCFP_6 feature: -124685461: [*]:n1:[*]:[*]:n:[cH]:1
11. Unknown FCFP_6 feature: 71476542: [*]:[c](:[*])Cl

Feature Contribution

Top features for positive contribution			
**Fingerprint**	**Bit/Smiles**	**Feature Structure**	**Score**

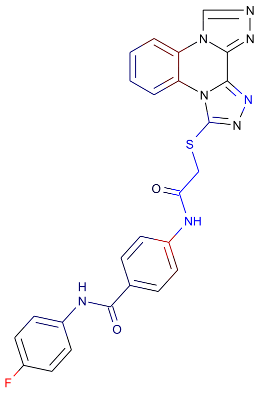

$C_{25}H_{17}FN_8O_2S$
Molecular Weight: 512.51828
ALogP: 3.875
Rotatable Bonds: 6
Acceptors: 7
Donors: 2

Model Prediction

Prediction: 0.592
Unit: g/kg_body_weight
Mahalanobis Distance: 31.063
Mahalanobis Distance p-value: 2.19e-072

Mahalanobis Distance: The Mahalanobis distance (MD) is a generalization of the Euclidean distance that accounts for correlations among the X properties. It is calculated as the distance to the center of the training data. The larger the MD, the less trustworthy the prediction.
Mahalanobis Distance p-value: The p-value gives the fraction of training data with an MD greater than or equal to the one for the given sample, assuming normally distributed data. The smaller the p-value, the less trustworthy the prediciton. For highly non-normal X properties (e.g., fingerprints), the MD p-value is wildly inaccurate.

Structural Similar Compounds

Name	BENZENESULFONIC ACID; 2;2'-(4;4'-BIPHENYLYLENEDIVINYLENE)DI-; DISODIUM SALT (Na STRIPPED)	ACEMETACIN	BENZOTHIAZOLE; 6-NITRO-2-(p-NITROBENZOYLAMINO)-
Structure	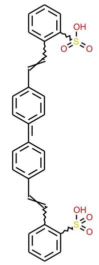	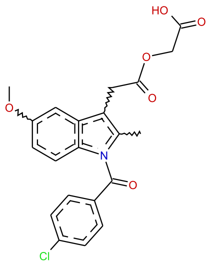	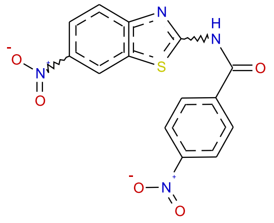
Actual Endpoint (-log C)	1.968	4.235	2.361
Predicted Endpoint (-log C)	1.72109	3.39415	2.96257
Distance	0.941	1.133	1.149
Reference	MVCRB3 2;193;73	ARZNAD 30;1398;80	JPETAB 90;260;47

Model Applicability

Unknown features are fingerprint features in the query molecule, but not found in the training set.

1. All properties and OPS components are within expected ranges.
2. Unknown ECFP_2 feature: 1312166648: [*]:[c](:[*]):n1:[c](:[*]):[*]:[*]:c:1
3. Unknown ECFP_2 feature: 1986731747: [*]S[c]1:n:[*]:[*]:n:1:[*]
4. Unknown FCFP_6 feature: 16: [*][c](:[*]):[*]
5. Unknown FCFP_6 feature: 1618154665: [*][c](:[*]):[cH]:[cH]:[*]
6. Unknown FCFP_6 feature: -203115083: [*]:[c](:[*]):n1:[cH]:[*]:[*]:[c]:1:[*]
7. Unknown FCFP_6 feature: -1564473960: [*]:n1:[*]:[*]:n:[c]:1:[c](:[*]):[*]
8. Unknown FCFP_6 feature: 4427049: [*]:[c]1:[*]:[*]:n:n:1
9. Unknown FCFP_6 feature: -1410079687: [*]S[c]1:n:[*]:[*]:n:1:[*]
10. Unknown FCFP_6 feature: -124685461: [*]:n1:[*]:[*]:n:[cH]:1
11. Unknown FCFP_6 feature: 71476542: [*]:[c](:[*])Cl

Feature Contribution

Top features for positive contribution			
Fingerprint	Bit/Smiles	Feature Structure	Score

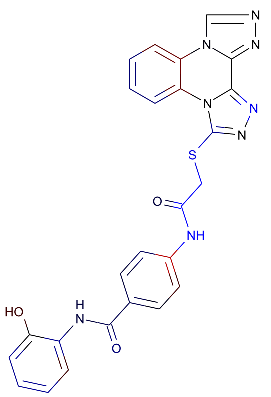

$C_{25}H_{18}N_8O_3S$
Molecular Weight: 510.52722
ALogP: 3.428
Rotatable Bonds: 6
Acceptors: 8
Donors: 3

Model Prediction

Prediction: 0.663
Unit: g/kg_body_weight
Mahalanobis Distance: 31.716
Mahalanobis Distance p-value: 1.4e-077

Mahalanobis Distance: The Mahalanobis distance (MD) is a generalization of the Euclidean distance that accounts for correlations among the X properties. It is calculated as the distance to the center of the training data. The larger the MD, the less trustworthy the prediction.
Mahalanobis Distance p-value: The p-value gives the fraction of training data with an MD greater than or equal to the one for the given sample, assuming normally distributed data. The smaller the p-value, the less trustworthy the prediciton. For highly non-normal X properties (e.g., fingerprints), the MD p-value is wildly inaccurate.

Structural Similar Compounds

Name	BENZENESULFONIC ACID; 2;2'-(4;4'-BIPHENYLYLENEDIVINYLENE)DI-; DISODIUM SALT (Na STRIPPED)	AZOSEMIDE	ANTHRAQUINONE; 1;5-DIAMINO-4;8-DIHYDROXY-3-(p-METHOXYPHENYL)-
Structure	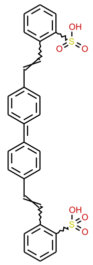	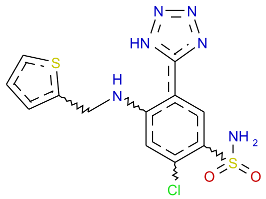	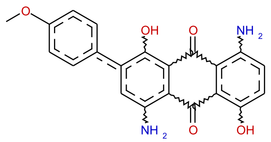
Actual Endpoint (-log C)	1.968	2.163	1.771
Predicted Endpoint (-log C)	1.72109	2.21052	2.1122
Distance	1.021	1.147	1.179
Reference	MVCRB3 2;193;73	IYKEDH 18;666;87	28ZPAK -;245;72

Model Applicability

Unknown features are fingerprint features in the query molecule, but not found in the training set.

1. All properties and OPS components are within expected ranges.
2. Unknown ECFP_2 feature: 1312166648: [*]:[c](:[*]):n1:[c](:[*]):[*]:[*]:c:1
3. Unknown ECFP_2 feature: 1986731747: [*]S[c]1:n:[*]:[*]:n:1:[*]
4. Unknown FCFP_6 feature: 16: [*][c](:[*]):[*]
5. Unknown FCFP_6 feature: 1618154665: [*][c](:[*]):[cH]:[cH]:[*]
6. Unknown FCFP_6 feature: -203115083: [*]:[c](:[*]):n1:[cH]:[*]:[*]:[c]:1:[*]
7. Unknown FCFP_6 feature: -1564473960: [*]:n1:[*]:[*]:n:[c]:1:[c](:[*]):[*]
8. Unknown FCFP_6 feature: 4427049: [*]:[c]1:[*]:[*]:n:n:1
9. Unknown FCFP_6 feature: -1410079687: [*]S[c]1:n:[*]:[*]:n:1:[*]
10. Unknown FCFP_6 feature: -124685461: [*]:n1:[*]:[*]:n:[cH]:1
11. Unknown FCFP_6 feature: 74595001: [*][c](:[*]):[c](O):[cH]:[*]
12. Unknown FCFP_6 feature: -549108873: [*]:[c](:[*])O

Feature Contribution

Top features for positive contribution			
Fingerprint	Bit/Smiles	Feature Structure	Score

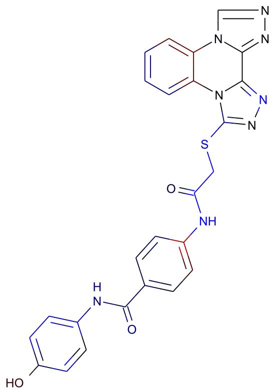

$C_{25}H_{18}N_8O_3S$
Molecular Weight: 510.52722
ALogP: 3.428
Rotatable Bonds: 6
Acceptors: 8
Donors: 3

Structural Similar Compounds

Name	BENZENESULFONIC ACID; 2;2'-(4;4'-BIPHENYLYLENEDIVINYLENE)DI-; DISODIUM SALT (Na STRIPPED)	AZOSEMIDE	ANTHRAQUINONE; 1;5-DIAMINO-4;8-DIHYDROXY-3-(p-METHOXYPHENYL)-
Structure	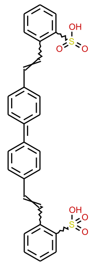	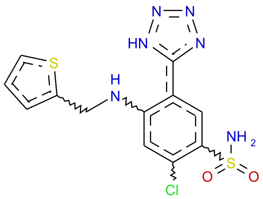	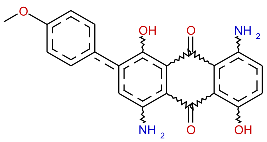
Actual Endpoint (-log C)	1.968	2.163	1.771
Predicted Endpoint (-log C)	1.72109	2.21052	2.1122
Distance	1.021	1.148	1.180
Reference	MVCRB3 2;193;73	IYKEDH 18;666;87	28ZPAK -;245;72

Model Prediction

Prediction: 0.864
Unit: g/kg_body_weight
Mahalanobis Distance: 30.775
Mahalanobis Distance p-value: 3.93e-070

Mahalanobis Distance: The Mahalanobis distance (MD) is a generalization of the Euclidean distance that accounts for correlations among the X properties. It is calculated as the distance to the center of the training data. The larger the MD, the less trustworthy the prediction.
Mahalanobis Distance p-value: The p-value gives the fraction of training data with an MD greater than or equal to the one for the given sample, assuming normally distributed data. The smaller the p-value, the less trustworthy the prediciton. For highly non-normal X properties (e.g., fingerprints), the MD p-value is wildly inaccurate.

Model Applicability

Unknown features are fingerprint features in the query molecule, but not found in the training set.

1. All properties and OPS components are within expected ranges.
2. Unknown ECFP_2 feature: 1312166648: [*]:[c](:[*]):n1:[c](:[*]):[*]:[*]:c:1
3. Unknown ECFP_2 feature: 1986731747: [*]S[c]1:n:[*]:[*]:n:1:[*]
4. Unknown FCFP_6 feature: 16: [*][c](:[*]):[*]
5. Unknown FCFP_6 feature: 1618154665: [*][c](:[*]):[cH]:[cH]:[*]
6. Unknown FCFP_6 feature: -203115083: [*]:[c](:[*]):n1:[cH]:[*]:[*]:[c]:1:[*]
7. Unknown FCFP_6 feature: -1564473960: [*]:n1:[*]:[*]:n:[c]:1:[c](:[*]):[*]
8. Unknown FCFP_6 feature: 4427049: [*]:[c]1:[*]:[*]:n:n:1
9. Unknown FCFP_6 feature: -1410079687: [*]S[c]1:n:[*]:[*]:n:1:[*]
10. Unknown FCFP_6 feature: -124685461: [*]:n1:[*]:[*]:n:[cH]:1
11. Unknown FCFP_6 feature: 74595001: [*][c](:[*]):[c](O):[cH]:[*]
12. Unknown FCFP_6 feature: -549108873: [*]:[c](:[*])O

Feature Contribution

Top features for positive contribution			
Fingerprint	Bit/Smiles	Feature Structure	Score

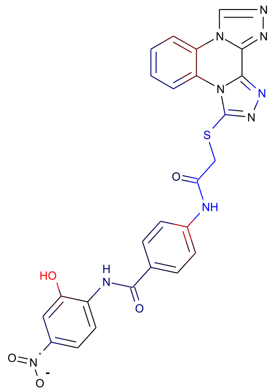

$C_{25}H_{17}N_9O_5S$
Molecular Weight: 555.52477
ALogP: 3.322
Rotatable Bonds: 7
Acceptors: 10
Donors: 3

Model Prediction

Prediction: 0.769
Unit: g/kg_body_weight
Mahalanobis Distance: 32.693
Mahalanobis Distance p-value: 1.37e-085

Mahalanobis Distance: The Mahalanobis distance (MD) is a generalization of the Euclidean distance that accounts for correlations among the X properties. It is calculated as the distance to the center of the training data. The larger the MD, the less trustworthy the prediction.
Mahalanobis Distance p-value: The p-value gives the fraction of training data with an MD greater than or equal to the one for the given sample, assuming normally distributed data. The smaller the p-value, the less trustworthy the prediciton. For highly non-normal X properties (e.g., fingerprints), the MD p-value is wildly inaccurate.

Structural Similar Compounds

Name	2;7-NAPHTHALENE DISULFONIC ACID; 4-AMINO-5-HYDROXY-; p-TOLUENE SULFONATE ESTER	BENZENESULFONIC ACID; 2;2'-(4;4'-BIPHENYLYLENEDIVINYLENE)DI-; DISODIUM SALT (Na STRIPPED)	METHOTREXATE
Structure	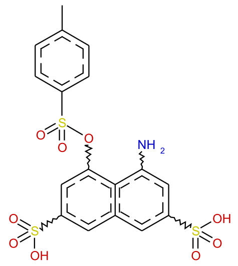	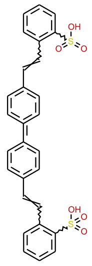	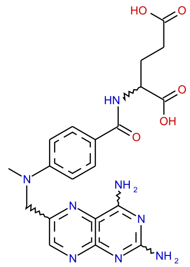
Actual Endpoint (-log C)	1.615	1.968	3.527
Predicted Endpoint (-log C)	1.79606	1.72109	2.39978
Distance	1.113	1.236	1.237
Reference	85JCAE -;1063;86	MVCRB3 2;193;73	NIIRDN 6;841;82

Model Applicability

Unknown features are fingerprint features in the query molecule, but not found in the training set.

1. All properties and OPS components are within expected ranges.
2. Unknown ECFP_2 feature: 1043790491: [*][N+](=[*])[*]
3. Unknown ECFP_2 feature: 781519895: [*][O-]
4. Unknown ECFP_2 feature: 1312166648: [*]:[c](:[*]):n1:[c](:[*]):[*]:[*]:c:1
5. Unknown ECFP_2 feature: 1986731747: [*]S[c]1:n:[*]:[*]:n:1:[*]
6. Unknown ECFP_2 feature: -179073144: [*][N+](=[*])[c](:c:[*]):c:[*]
7. Unknown ECFP_2 feature: -215026467: [*]:[c](:[*])[N+](=O)[O-]
8. Unknown ECFP_2 feature: 2104376220: [*][N+](=O)[*]
9. Unknown ECFP_2 feature: -659271057: [*][N+](=[*])[O-]
10. Unknown FCFP_6 feature: 16: [*][c](:[*]):[*]
11. Unknown FCFP_6 feature: 8: [*][N+](=[*])[*]
12. Unknown FCFP_6 feature: 5: [*][O-]
13. Unknown FCFP_6 feature: 1618154665: [*][c](:[*]):[cH]:[cH]:[*]
14. Unknown FCFP_6 feature: -203115083: [*]:[c](:[*]):n1:[cH]:[*]:[*]:[c]:1:[*]
15. Unknown FCFP_6 feature: -1564473960: [*]:n1:[*]:[*]:n:[c]:1:[c](:[*]):[*]
16. Unknown FCFP_6 feature: 4427049: [*]:[c]1:[*]:[*]:n:n:1
17. Unknown FCFP_6 feature: -1410079687: [*]S[c]1:n:[*]:[*]:n:1:[*]
18. Unknown FCFP_6 feature: -124685461: [*]:n1:[*]:[*]:n:[cH]:1

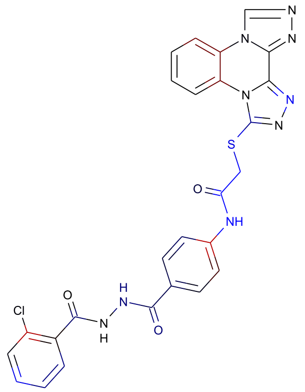

$C_{26}H_{18}ClN_9O_3S$
Molecular Weight: 571.99761
ALogP: 3.591
Rotatable Bonds: 7
Acceptors: 8
Donors: 3

Model Prediction

Prediction: 2.477
Unit: g/kg_body_weight
Mahalanobis Distance: 30.225
Mahalanobis Distance p-value: 6.53e-066

Mahalanobis Distance: The Mahalanobis distance (MD) is a generalization of the Euclidean distance that accounts for correlations among the X properties. It is calculated as the distance to the center of the training data. The larger the MD, the less trustworthy the prediction.
Mahalanobis Distance p-value: The p-value gives the fraction of training data with an MD greater than or equal to the one for the given sample, assuming normally distributed data. The smaller the p-value, the less trustworthy the prediciton. For highly non-normal X properties (e.g., fingerprints), the MD p-value is wildly inaccurate.

Structural Similar Compounds

Name	BENZENESULFONIC ACID; 2;2'-(4;4'-BIPHENYLYLENEDIVINYLENE)DI-; DISODIUM SALT (Na STRIPPED)	AZOSEMIDE	2;7-NAPHTHALENE DISULFONIC ACID; 4-AMINO-5-HYDROXY-; p-TOLUENE SULFONATE ESTER
Structure	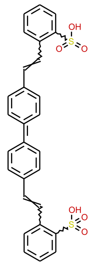	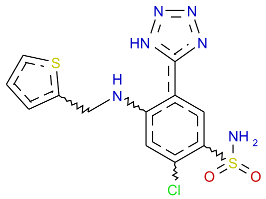	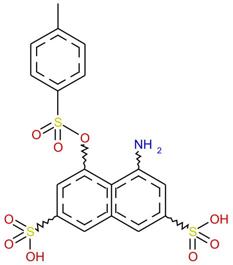
Actual Endpoint (-log C)	1.968	2.163	1.615
Predicted Endpoint (-log C)	1.72109	2.21052	1.79606
Distance	1.034	1.175	1.236
Reference	MVCRB3 2;193;73	IYKEDH 18;666;87	85JCAE -;1063;86

Model Applicability

Unknown features are fingerprint features in the query molecule, but not found in the training set.

1. All properties and OPS components are within expected ranges.
2. Unknown ECFP_2 feature: 1312166648: [*]:[c](:[*]):n1:[c](:[*]):[*]:[*]:c:1
3. Unknown ECFP_2 feature: 1986731747: [*]S[c]1:n:[*]:[*]:n:1:[*]
4. Unknown FCFP_6 feature: 16: [*][c](:[*]):[*]
5. Unknown FCFP_6 feature: 1618154665: [*][c](:[*]):[cH]:[cH]:[*]
6. Unknown FCFP_6 feature: -885461129: [*]NNC(=[*])[*]
7. Unknown FCFP_6 feature: -203115083: [*]:[c](:[*]):n1:[cH]:[*]:[*]:[c]:1:[*]
8. Unknown FCFP_6 feature: -1564473960: [*]:n1:[*]:[*]:n:[c]:1:[c](:[*]):[*]
9. Unknown FCFP_6 feature: 4427049: [*]:[c]1:[*]:[*]:n:n:1
10. Unknown FCFP_6 feature: -1410079687: [*]S[c]1:n:[*]:[*]:n:1:[*]
11. Unknown FCFP_6 feature: -124685461: [*]:n1:[*]:[*]:n:[cH]:1
12. Unknown FCFP_6 feature: 71476542: [*]:[c](:[*])Cl

Feature Contribution

Top features for positive contribution			
Fingerprint	Bit/Smiles	Feature Structure	Score

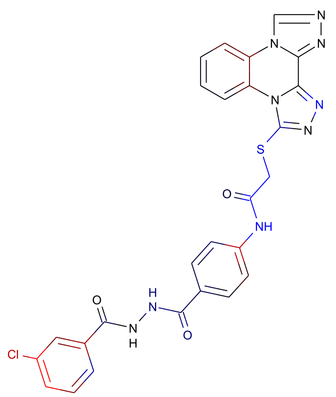

$C_{26}H_{18}ClN_9O_3S$

Molecular Weight: 571.99761

ALogP: 3.591

Rotatable Bonds: 7

Acceptors: 8

Donors: 3

Model Prediction

Prediction: 1.754

Unit: g/kg_body_weight

Mahalanobis Distance: 30.370

Mahalanobis Distance p-value: 5.12e-067

Mahalanobis Distance: The Mahalanobis distance (MD) is a generalization of the Euclidean distance that accounts for correlations among the X properties. It is calculated as the distance to the center of the training data. The larger the MD, the less trustworthy the prediction.
Mahalanobis Distance p-value: The p-value gives the fraction of training data with an MD greater than or equal to the one for the given sample, assuming normally distributed data. The smaller the p-value, the less trustworthy the prediciton. For highly non-normal X properties (e.g., fingerprints), the MD p-value is wildly inaccurate.

Structural Similar Compounds

Name	BENZENESULFONIC ACID; 2;2'-(4;4'-BIPHENYLYLENEDIVINYLENE)DI-; DISODIUM SALT (Na STRIPPED)	AZOSEMIDE	2;7-NAPHTHALENE DISULFONIC ACID; 4-AMINO-5-HYDROXY-; p-TOLUENE SULFONATE ESTER
Structure	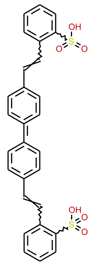	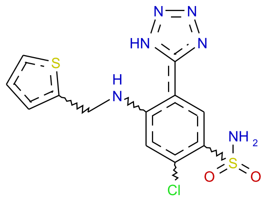	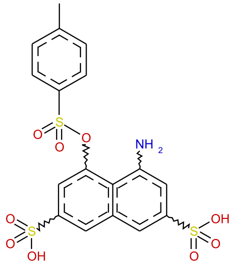
Actual Endpoint (-log C)	1.968	2.163	1.615
Predicted Endpoint (-log C)	1.72109	2.21052	1.79606
Distance	1.038	1.179	1.234
Reference	MVCRB3 2;193;73	IYKEDH 18;666;87	85JCAE -;1063;86

Model Applicability

Unknown features are fingerprint features in the query molecule, but not found in the training set.

1. All properties and OPS components are within expected ranges.
2. Unknown ECFP_2 feature: 1312166648: [*]:[c](:[*]):n1:[c](:[*]):[*]:[*]:c:1
3. Unknown ECFP_2 feature: 1986731747: [*]S[c]1:n:[*]:[*]:n:1:[*]
4. Unknown FCFP_6 feature: 16: [*][c](:[*]):[*]
5. Unknown FCFP_6 feature: 1618154665: [*][c](:[*]):[cH]:[cH]:[*]
6. Unknown FCFP_6 feature: -885461129: [*]NNC(=[*])[*]
7. Unknown FCFP_6 feature: -203115083: [*]:[c](:[*]):n1:[cH]:[*]:[*]:[c]:1:[*]
8. Unknown FCFP_6 feature: -1564473960: [*]:n1:[*]:[*]:n:[c]:1:[c](:[*]):[*]
9. Unknown FCFP_6 feature: 4427049: [*]:[c]1:[*]:[*]:n:n:1
10. Unknown FCFP_6 feature: -1410079687: [*]S[c]1:n:[*]:[*]:n:1:[*]
11. Unknown FCFP_6 feature: -124685461: [*]:n1:[*]:[*]:n:[cH]:1
12. Unknown FCFP_6 feature: 71476542: [*]:[c](:[*])Cl

Feature Contribution

Top features for positive contribution			
Fingerprint	Bit/Smiles	Feature Structure	Score

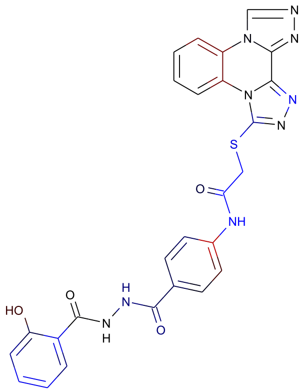

$C_{26}H_{19}N_9O_4S$

Molecular Weight: 553.55195

ALogP: 2.685

Rotatable Bonds: 7

Acceptors: 9

Donors: 4

Structural Similar Compounds

Name	2;7-NAPHTHALENE DISULFONIC ACID; 4-AMINO-5-HYDROXY-; p-TOLUENE SULFONATE ESTER	METHOTREXATE	BENZENESULFONIC ACID; 2;2'-(4;4'-BIPHENYLYLENEDIVINYLENE)DI-; DISODIUM SALT (Na STRIPPED)
Structure	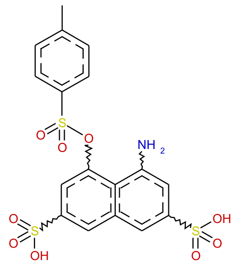	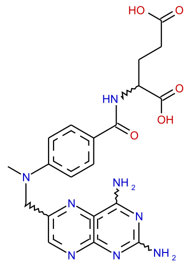	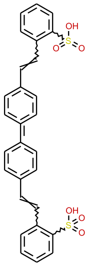
Actual Endpoint (-log C)	1.615	3.527	1.968
Predicted Endpoint (-log C)	1.79606	2.39978	1.72109
Distance	1.155	1.200	1.214
Reference	85JCAE -;1063;86	NIIRDN 6;841;82	MVCRB3 2;193;73

Model Prediction

Prediction: 1.350

Unit: g/kg_body_weight

Mahalanobis Distance: 30.426

Mahalanobis Distance p-value: 1.92e-067

Mahalanobis Distance: The Mahalanobis distance (MD) is a generalization of the Euclidean distance that accounts for correlations among the X properties. It is calculated as the distance to the center of the training data. The larger the MD, the less trustworthy the prediction.
Mahalanobis Distance p-value: The p-value gives the fraction of training data with an MD greater than or equal to the one for the given sample, assuming normally distributed data. The smaller the p-value, the less trustworthy the prediciton. For highly non-normal X properties (e.g., fingerprints), the MD p-value is wildly inaccurate.

Model Applicability

Unknown features are fingerprint features in the query molecule, but not found in the training set.

1. All properties and OPS components are within expected ranges.
2. Unknown ECFP_2 feature: 1312166648: [*]:[c](:[*]):n1:[c](:[*]):[*]:[*]:c:1
3. Unknown ECFP_2 feature: 1986731747: [*]S[c]1:n:[*]:[*]:n:1:[*]
4. Unknown FCFP_6 feature: 16: [*][c](:[*]):[*]
5. Unknown FCFP_6 feature: 1618154665: [*][c](:[*]):[cH]:[cH]:[*]
6. Unknown FCFP_6 feature: -885461129: [*]NNC(=[*])[*]
7. Unknown FCFP_6 feature: 74595001: [*][c](:[*]):[c](O):[cH]:[*]
8. Unknown FCFP_6 feature: -203115083: [*]:[c](:[*]):n1:[cH]:[*]:[*]:[c]:1:[*]
9. Unknown FCFP_6 feature: -1564473960: [*]:n1:[*]:[*]:n:[c]:1:[c](:[*]):[*]
10. Unknown FCFP_6 feature: 4427049: [*]:[c]1:[*]:[*]:n:n:1
11. Unknown FCFP_6 feature: -1410079687: [*]S[c]1:n:[*]:[*]:n:1:[*]
12. Unknown FCFP_6 feature: -124685461: [*]:n1:[*]:[*]:n:[cH]:1
13. Unknown FCFP_6 feature: -549108873: [*]:[c](:[*])O

Feature Contribution

Top features for positive contribution

Fingerprint	Bit/Smiles	Feature Structure	Score

Sorafenib

TOPKAT_Rat_Oral_LD50

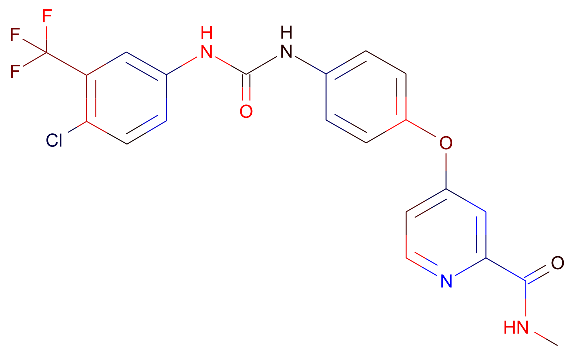

$C_{21}H_{16}ClF_3N_4O_3$
Molecular Weight: 464.82494
ALogP: 4.175
Rotatable Bonds: 6
Acceptors: 4
Donors: 3

Structural Similar Compounds

Name	FLUBENDAZOLE	PHOSPHORAMIDOTHIOIC ACID; ACETIMIDOYL-; O;O-bis-(p-CHLOROPHENYL)ESTER	BEZAFIBRATE
Structure	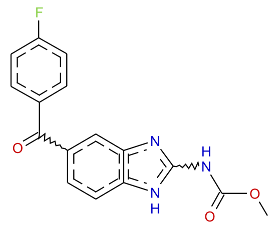	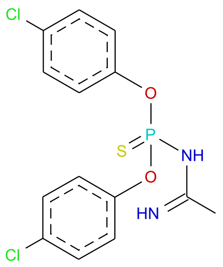	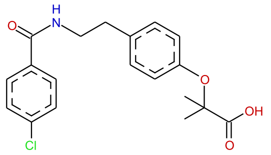
Actual Endpoint (-log C)	2.088	5.006	1.946
Predicted Endpoint (-log C)	2.69288	3.23989	2.54395
Distance	0.697	0.703	0.721
Reference	YRTMA6 9;11;78	FMCHA2 -;C149;89	ARZNAD 30;2023;80

Model Prediction

Prediction: 0.823
Unit: g/kg_body_weight
Mahalanobis Distance: 21.029
Mahalanobis Distance p-value: 1.93e-012

Mahalanobis Distance: The Mahalanobis distance (MD) is a generalization of the Euclidean distance that accounts for correlations among the X properties. It is calculated as the distance to the center of the training data. The larger the MD, the less trustworthy the prediction.
Mahalanobis Distance p-value: The p-value gives the fraction of training data with an MD greater than or equal to the one for the given sample, assuming normally distributed data. The smaller the p-value, the less trustworthy the prediciton. For highly non-normal X properties (e.g., fingerprints), the MD p-value is wildly inaccurate.

Model Applicability

Unknown features are fingerprint features in the query molecule, but not found in the training set.

1. All properties and OPS components are within expected ranges.
2. Unknown FCFP_6 feature: 16: [*][c](:[*]):[*]
3. Unknown FCFP_6 feature: 1618154665: [*][c](:[*]):[cH]:[cH]:[*]
4. Unknown FCFP_6 feature: 1747237384: [*][c](:[*]):n:[cH]:[*]
5. Unknown FCFP_6 feature: 136686699: [*]NC
6. Unknown FCFP_6 feature: 71476542: [*]:[c](:[*])Cl

Feature Contribution

Top features for positive contribution			
**Fingerprint**	**Bit/Smiles**	**Feature Structure**	**Score**
